# Base-Promoted Conversion of Propargylic Alcohols to 1,3-Enynes

**DOI:** 10.1021/acs.orglett.6c00196

**Published:** 2026-02-04

**Authors:** Morgane Delattre, Milena Wiegand, Qian Wang, Jieping Zhu

**Affiliations:** Laboratory of Synthesis and Natural Products (LSPN), Institute of Chemical Sciences and Engineering, 27218 Ecole Polytechnique Fédérale de Lausanne, EPFL-SB-ISIC-LSPN, BCH 5304, 1015 Lausanne, Switzerland

## Abstract

Under acidic conditions, propargylic alcohols undergo Meyer–Schuster or Rupe rearrangements to afford two isomeric α,β-unsaturated ketones. Herein, we disclose a mechanistically distinct base-mediated regioselective conversion of TMS ethers of propargylic alcohols to 1,3-enynes in high yields with broad functional-group compatibility. Alternatively, trapping of the in situ-generated lithium acetylide with electrophiles enables access to functionalized internal 1,3-enynes. Owing to the ready accessibility of propargylic alcohols, this method provides a practical and attractive entry to synthetically valuable 1,3-enynes.

## Graphical abstract

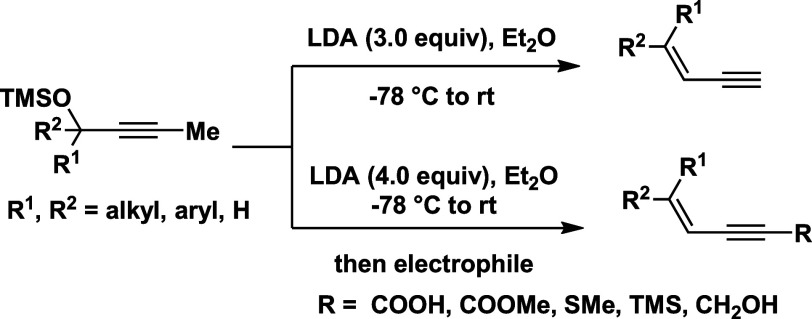

Propargylic alcohols **1**, readily accessible from terminal alkynes and carbonyl compounds, are versatile and widely used building blocks in organic synthesis.^1–3^ The combination of a C≡C bond with a vicinal hydroxyl group makes these substrates particularly susceptible to activation by Brønsted acids, Lewis acids, and transition metals, thereby enabling the numerous powerful synthetic transformations. They also serve as precursors to other important synthons. For instance, two competing reactions, namely, the Meyer–Schuster rearrangement (Scheme 1a)^4^ and the Rupe rearrangement (Scheme 1b),^5^ convert **1** into two isomeric α,β-unsaturated carbonyl compounds **2** and **3**, respectively, depending on the reaction conditions and the nature of substituents on the propargylic alcohols. Under classical acidic conditions, the Meyer–Schuster reaction has mainly been limited to substrates lacking β-hydrogens, rendering the Rupe pathway inaccessible.^6,7^

**Scheme 1:**
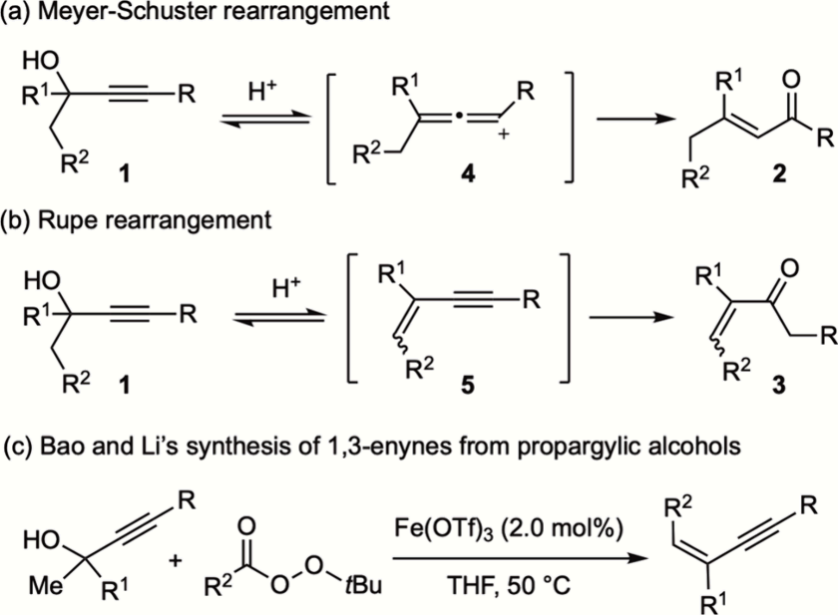
Meyer–Schuster Rearrangement, Rupe Rearrangement, and Variations

Mechanistically, allenic cation **4** has been proposed as the key intermediate in the Meyer–Schuster reaction. Trapping this highly reactive cationic species with a variety of nucleophiles has enabled the development of numerous synthetically powerful transformations.^8^ On the other hand, reaction conditions that halt the Rupe rearrangement at intermediate 1,3-enyne **5**, which is another class of important building blocks in organic synthesis, have been identified.^9–13^ Exploiting the reactivity of the enyne toward radical addition, Bao and Li developed an elegant iron-catalyzed domino dehydrative alkylation of propargylic alcohols to access functionalized 1,3-enynes (Scheme 1c).^14^

The conversion of propargylic ethers to enynes under basic conditions has been only sporadically reported. Notably, Arens and co-workers showed that the treatment of propargylic ethers **6** with sodium amide in liquid ammonia afforded enynes **7** in good yields.^15,16^ However, the scope of this reaction has not been systematically investigated.^17^ In connection with our ongoing project,^18^ we had occasion to examine the carbocyclization of **8** via its zinc enolate^19^ and observed the formation of 1,3-enyne **9** as a minor product, rather than the anticipated cyclization product. This unexpected outcome, combined with the facile accessibility of propargylic ethers and the synthetic value of 1,3-enynes,^20–23^ motivated us to investigate this transformation in detail. Herein, we report that exposure of a diethyl ether solution of propargylic ethers **10** to LDA (3.0 equiv) affords terminal 1,3-enynes **11** in high yields with good *E* selectivity. When 4 equiv of LDA was employed, the in situ-formed lithium acetylide can be trapped by electrophiles to generate functionalized 1,3-enynes **12** (Scheme 2c). Importantly, the relative positions of the alkene and alkyne units in 1,3-enynes **11** and **12** differ from those obtained in the transformation reported by Arens and co-workers (cf. compound **7**).^15^

**Scheme 2:**
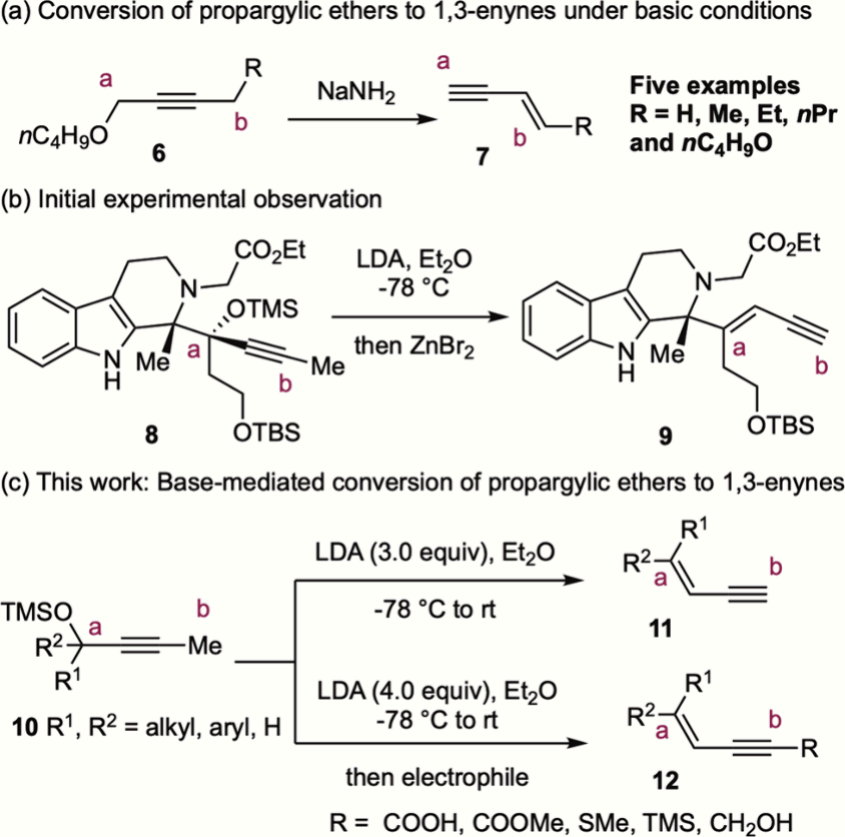
Base-Promoted Conversion of Propargylic Ethers to 1,3-Enynes (literature precedent and this work)

Trimethyl­[(2-phenylpent-3-yn-2yl)­oxy]­silane (**10a**) (R^1^ = Me; R^2^ = Ph) was selected for reaction optimization (Supporting Information). Some key experimental observations emerged. (a) Three equivalents of LDA proved to be optimal. LTMP (lithium 2,2,6,6-tetramethylpiperidide) gave a slightly lower yield of **11a** under otherwise identical conditions, whereas no product was formed when *n*BuLi and NaH were employed as bases. (b) The presence of ZnBr_2_ and InBr_3_ was detrimental to the desired reaction. (c) Among the hydroxyl protective groups examined (OPiv, OBz, and OBoc), the OTMS group provided superior results. (d) Comparable outcomes were obtained in diethyl ether and tetrahydrofuran (THF), with an optimal substrate concentration of 0.15 M in Et_2_O. Overall, addition of LDA (3.0 equiv) to a diethyl ether solution of **10a** (*c* = 0.15 M) at −78 °C, followed by warming to room temperature and stirring for 45 min, afforded (*E*)-4-phenyl-pent-3-en-1-yne (**11a**) in 74% yield. The *E* geometry of the double bond was assigned to the major product on the basis of the NOE experiment.

With the optimized conditions in hand, the reaction scope was examined starting with the TMS ether of the tertiary propargylic alcohols (Scheme 3). The TMS ether of 2-(4-hydroxyphenyl)­pent-3-yn-2-ol (**10b**) was converted into **11b** in 95% yield. The phenolic hydroxyl group was tolerated, although 4.5 equiv of LDA was required to ensure complete conversion. The TMS ether of 2-(pyridin-4-yl)­pent-3-yn-2-ol (**10c**) was similarly transformed into **11c**, demonstrating compatibility with a basic heteroarene. The presence of an aryl substituent in **10** was not a prerequisite, since 1,1-dialkyl-substituted but-2-yn-1-ol derivatives were converted into the corresponding 1,3-enynes (**11d**–**11i**) in excellent yields. A diyne substrate (**10j**) was highly regioselectively converted into enediyne **11j**. Cyclic ketone-derived propargylic alcohols, including those originating from cyclohexanones (**11k**–**11n**), tetrahydro-4*H*-pyran-4-one (**11o**), *N*-Boc-piperidin-4-one (**11p**), *N*-Boc-2-azaspiro­[3,5]­nonan-7-one (**11q**), and adamantan-2-ones (**11r** and **11s**), were smoothly converted into the corresponding enynes. Chiral substrates delivered enynes **11t** and **11u** without detectable epimerization. In addition, a range of prop-2-yn-1-ylidenecycloalkanes (**11v**–**11z**) were obtained in good yields. Importantly, diverse functional groups, including silyl ether, alkyl azide, chloride, methyl ether, acetal, and carbamate, were well tolerated under these reaction conditions.

**Scheme 3:**
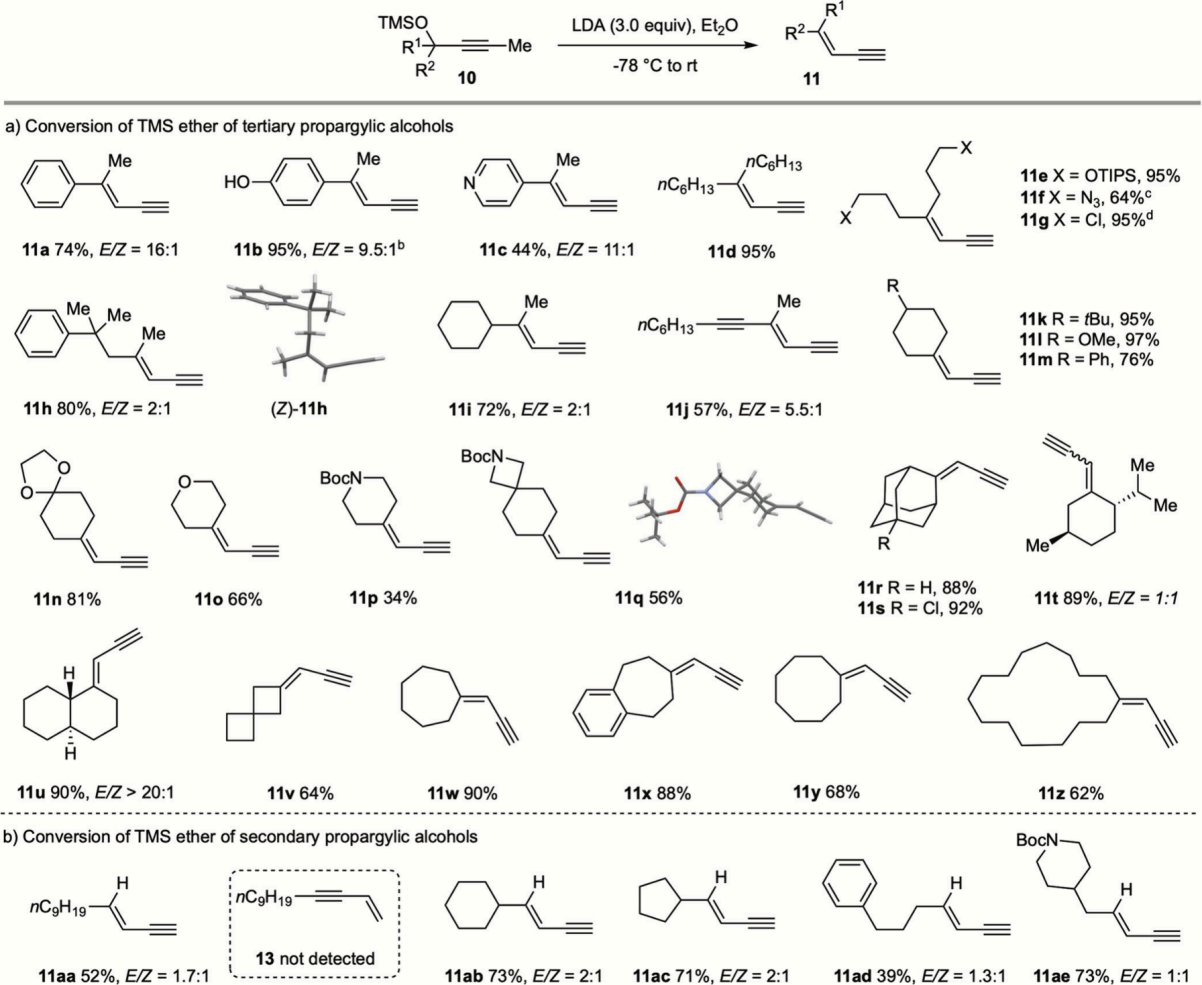
Conversion of TMS Ether of Propargylic Alcohols into 1,3-Enynes^a^

a Standard conditions: **10** (0.2 mmol), LDA (3.0 equiv), Et_2_O (1.3 mL; *c* = 0.15 M), −78 °C to rt.
b LDA (4.5 equiv).
c LDA (6.0 equiv), Et_2_O (*c* 0.08 M).
d LDA (2.3 equiv).

We next investigated the conversion of the TMS ethers derived from secondary propargylic alcohols (Scheme 3b). Treatment of TMS ether of tridec-2-yn-4-ol (**10aa**) with LDA (3.0 equiv) in Et_2_O afforded tridec-3-en-1-yne (**11aa**) in 52% yield (1.7:1 *E*:*Z*). Notably, the regioisomeric tridec-1-en-3-yne (**13** (inset of Scheme 3b)) was not detected. Enynes **11ab**–**11ae** were similarly prepared as a mixture of *E* and *Z* isomers.

A gram-scale experiment converted **10d** (1.0 g, 3.23 mmol) into **11d** in 90% yield, highlighting the practicality and scalability of this protocol.

We note that no reaction occurred when the TMS ether of 2-phenylhept-3-yn-2-ol was subjected to the standard conditions. Therefore, the protocol can be applied only to the synthesis of terminal alkynes. To circumvent this limitation, functionalization of the terminal alkyne generated in situ was subsequently explored. As shown in Scheme 4, increasing the LDA loading to 4 equiv enabled trapping of the resulting alkynyllithium with a variety of electrophiles, including carbon dioxide, dimethyl carbonate, gaseous formaldehyde, trimethylsilyl chloride, and *S*-methylmethanethiosulfonate,^24^ to afford functionalized internal enynes **12a**–**12g** in excellent yields.^25^

**Scheme 4:**
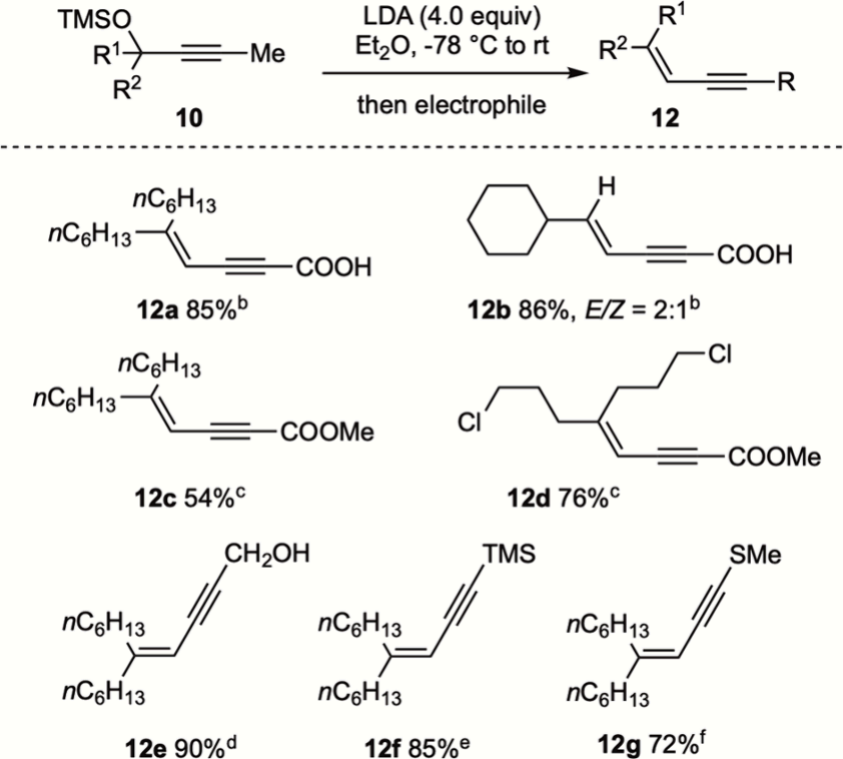
Functionalization of In Situ-Generated Terminal Alkynes^a^

a Standard conditions: **10** (0.2 mmol), LDA (4.0 equiv), Et_2_O (1.3 mL; *c* = 0.15 M), −78 °C to rt, 2 h, then an electrophile.
b Using CO_2_ as an electrophile.
c Using dimethyl carbonate as an electrophile.
d Using gaseous formaldehyde as an electrophile.
e Using TMSCl as an electrophile.
f Using *S*-methylmethanethiosulfonate as an electrophile.

Control experiments were conducted to gain insight into the reaction mechanism. When propargylic alcohol **10d** was subjected to the standard conditions and the reaction was quenched with D_2_O, C1-deuterated product **11d-D** and nondeuterated **11d** were obtained in 80% and 15% yields, respectively (Scheme 5a). No C3-deuterated products were detected in the reaction mixture. In contrast, exposure of **10d-D**_3_ to the standard conditions afforded a mixture of four identifiable compounds, consisting of nondeuterated **11d** together with deuterated products **14–16** (Scheme 5b). Notably, the C3 vinylic atom was deuterated in products **14–16**. For the success of this deuterium labeling experiment, it is important to generate LDA with an excess of *n*BuLi to ensure that the diisopropylamine (*i*Pr_2_NH) formed in situ was fully reconverted into LDA, thereby minimizing competitive protonation of organolithium intermediates. Reaction of **11g**, obtained from **10g** under the standard conditions, with LDA furnished cyclopropane derivative **17** in 80% yield. By contrast, subjecting **10g** to a large excess of LDA (9.0 equiv) led to a mixture of two inseparable compounds, biscyclopropane **18** and 1,2-disubstituted cyclopentene **19**, in a 54% combined yield (1.4:1). Finally, reaction of **10d** with 1.0 equiv of LDA, followed by quenching at approximately 30% conversion, allowed the isolation of cumulene **20** in ca. 1% yield.^26^ Subsequent reaction of **20** with 1.3 equiv of LDA delivered expected 1,3-enyne **11d** in 80% yield. Collectively, these control experiments support the involvement of a cumulene intermediate as well as both C1-lithiated and C3-lithiated species in the conversion of **10** into **11**.

**Scheme 5:**
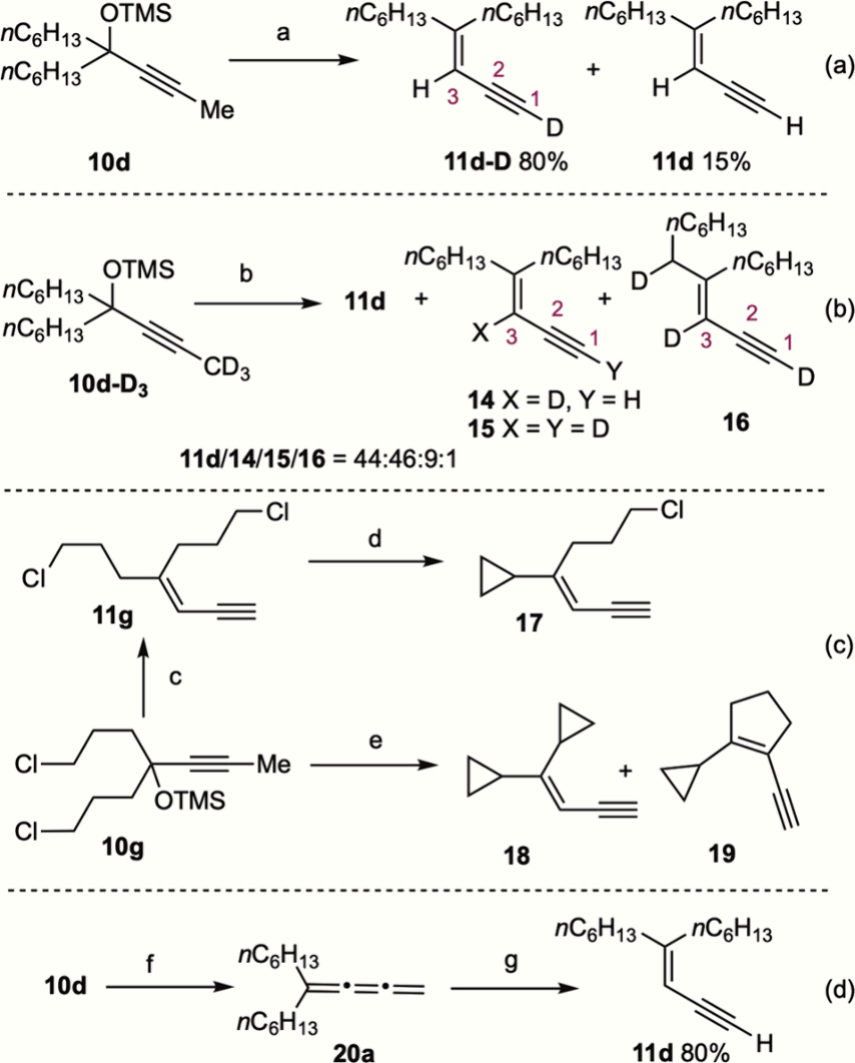
Mechanistic Studies^a^

a Reagents and conditions: (a) LDA (3.5 equiv), Et_2_O, −78 °C to rt, 2 h, then D_2_O; (b) LDA (2.3 + 1.0 equiv), Et_2_O, −78 °C to rt, 2 h, 44:46:9:1 **11d**:**14**:**15**:**16**, ratio estimated based on ^1^H NMR and HRMS analysis; (c) LDA (2.3 equiv), Et_2_O, −78 °C to rt, 105 min, 95%; (d) LDA (2.6 equiv), Et_2_O, −78 °C to rt, 2 h, 80%; (e) LDA (9.0 equiv), Et_2_O, −78 °C to rt, 20 h, 54%, 1.4:1 **18**:**19**; (f) LDA (1.0 equiv), Et_2_O, −78 °C to rt, 25 min, 29% conversion, 0.9%; (g) LDA (1.3 equiv), Et_2_O, −78 °C to rt, 1 h, 80%.

Building on the control experiments presented above, a plausible reaction mechanism is proposed in Scheme 6. Deprotonation of **10**, followed by elimination of lithium trimethylsilyl oxide, would generate cumulene **20**, which would then undergo further deprotonation to furnish **21**. Given that the acidity of allene C­(sp^2^)–H bond is comparable to that of an acetylene C–H bond (p*K*_a_ ∼ 25),^27^ resulting organolithium species **21** could not be protonated by *i*Pr_2_NH (p*K*_a_ ∼ 35). Instead, it underwent isomerization to give vinyl lithium species **22** (p*K*_aH_ ∼ 42), which could subsequently be protonated by *i*Pr_2_NH to afford 1,3-enyne **11**. Once formed, **11** would be immediately deprotonated by LDA leading to lithium acetylide **23**. Quenching the reaction mixture with water would then deliver product **11**, whereas trapping with an electrophile would lead to the formation of internal alkyne **12**. Note that the acidity of the C­(sp)–H bond in **11** is significantly higher than that of the propargylic C­(sp^3^)–H bond in starting material **10**. As a result, **11** is deprotonated before the complete consumption of **10**. Consequently, 3 equiv of base is required to drive the reaction to completion. This mechanistic scenario is consistent with all experimental observations shown in Scheme 5.

**Scheme 6:**
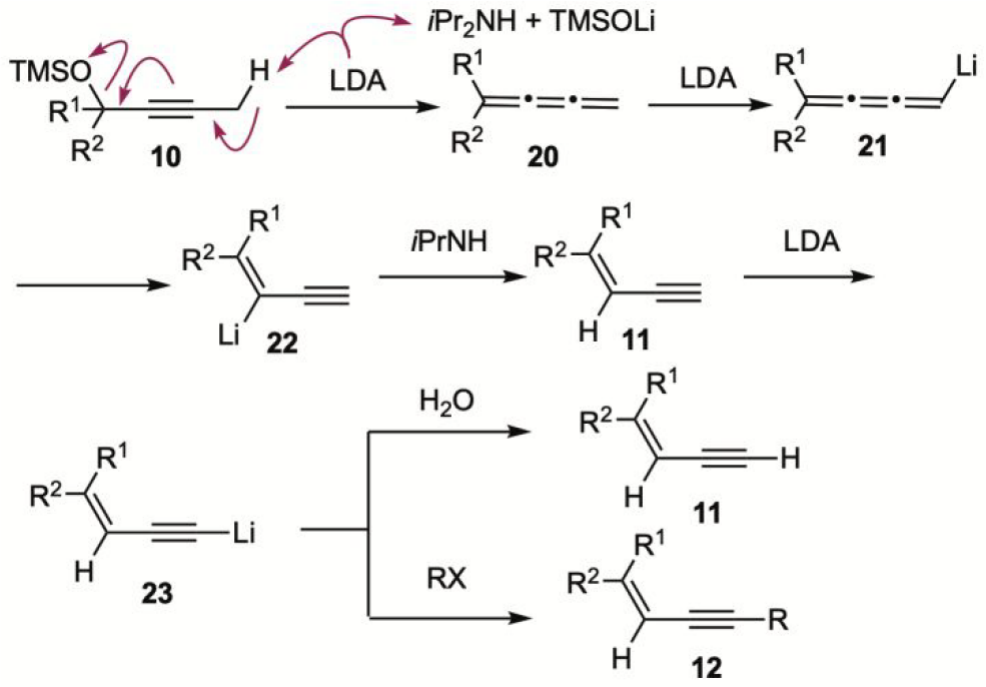
Plausible Reaction Mechanism

In summary, we have developed a base-mediated conversion of the TMS ether of propargylic alcohols into 1,3-enynes. The transformation proceeds under mild conditions and tolerates a wide range of functional groups. Given the ready accessibility of the starting materials, this method provides a practical and attractive alternative to 1,3-enynes of significant synthetic importance.

## Supplementary Material



## Data Availability

The data underlying this study are available in the published article and its Supporting Information.

## Notes

Experimental procedures and characterization data, additional experimental details, and copies of ^1^H and ^13^C NMR spectra (PDF)
