# Supplementary material for: Base-Promoted Conversion of Propargylic Alcohols to 1,3-Enynes
Source: Org Lett. 2026 Feb 4;28(7):2500–4. doi: 10.1021/acs.orglett.6c00196 (PMC12930487; doi:10.1021/acs.orglett.6c00196)
Supplement: Supplementary file 1 [file ol6c00196_si_001.pdf]

Base-Promoted Conversion of Propargylic Alcohols to 1,3-Enynes

Morgane Delattre, Milena Wiegand, Qian Wang, and Jieping Zhu*

Email: jieping.zhu@epfl.ch

Supporting information

Table of contents

1) **General information** S3
2) **Initial experimental observation** S3
3) **Synthesis and characterization data of the starting materials** S4
4) **Optimization of the reaction conditions** S30
5) **Synthesis and characterization data of 1,3-enyne derivatives** S32
6) **Mechanistic studies** S48
7) **Product modifications** S51
8) **References** S52
9) **X-Ray crystallographic data** S53
10) **Copies of the NMR spectra** S73

1) General information

Reagents and solvents were purchased from commercial sources and preserved under argon. More sensitive compounds were stored in a desiccator or in the glove-box if required. Reagents were used as received without further purification. All reactions were performed under argon (or nitrogen) and stirring unless otherwise noted. When needed, glassware was dried at least overnight in an oven (170 °C) or under vacuum with a heat gun (650 °C).

Solvents indicated as dry were either purchased as such, distilled prior to use or dried by a passage through a column of anhydrous alumina or copper using a Puresolv MD 5 from Innovative Technology Inc., based on the Grubbs' design. Flash column chromatography was performed using Silicycle SiliaFlash® P60 230-400 mesh. Reactions were monitored using Merck Kieselgel $60F_{254}$ aluminum or glass backed plates. TLC's were revealed by UV fluorescence (254 nm) then one of the following: $KMnO_4$, phosphomolybdic acid, ninhydrin, pancaldi, *p*-anisaldehyde, vanillin.

NMR spectra were recorded on a Brüker AvanceIII-400, Brüker Avance-400 Brüker DPX-400 spectrometer at room temperature, ^1H frequency is at 400.13 MHz, ^{13}C frequency is at 100.62 MHz. Chemical shifts (δ) were reported in parts per million (ppm) relative to residual solvent peaks rounded to the nearest 0.01 for proton and 0.1 for carbon (*ref: $CHCl_3$ [^1H: 7.26, ^{13}C: 77.2] and CD_2HOD [^1H: 3.34, ^{13}C: 49.9]).* Coupling constants (J) were reported in Hz to the nearest 0.1 Hz. Peak multiplicity was indicated as follows: s (singlet), d (doublet), t (triplet), q (quartet), p (quintet), m (multiplet) and br (broad). Attribution of peaks was done using the multiplicities and integrals of the peaks. COSY, HSQC, HMBC and NOESY experiments were performed when needed to confirm the assignment.

IR spectra were recorded in a Jasco FT/IR-4100 spectrometer outfitted with a PIKE technology MIRacle™ ATR accessory as neat films compressed onto a Zinc Selenide window or a Perkin Elmer Spectrum BX FT-IR. The spectra are reported in cm^{-1}. Abbreviations used are: w (weak), m (medium), s (strong) and br (broad).

Mass spectra were determined with a Waters ACQUITY H-class UPLC/MS ACQ-SQD by electron ionization (EI positive and negative) or a Finnigan TSQ7000 by electrospray ionization (ESI^+). The accurate masses were done by the mass spectrometry service of the EPFL by ESI-TOF using a QTOF Ultima from Waters.

Melting points were determined using a Stuart SMP30 or Büchi B-540.

Optical rotations α_D were measured with a Jasco P-2000 polarimeter (589 nm).

Cautionary note: diisopropylamine is highly flammable and corrosive. *n*-BuLi is pyrophoric and reacts violently with air and moisture. Both must be handled using appropriate inert atmosphere techniques and standard safety precautions.

2) Initial experimental observation

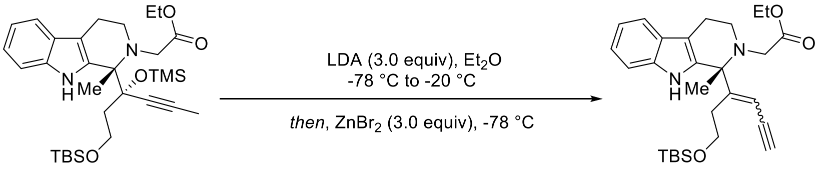

8 9

Diisopropylamine was distilled over CaH_2. In an oven-dried flask containing dry THF (0.12 mL) at -78 °C and diisopropylamine (10 µL, 0.07 mmol, 3.0 equiv) was added dropwise *n*BuLi (2.5 M in hexane) (28 µL, 0.07 mmol, 3.0 equiv). The reaction mixture was stirred at -78 °C for 20 min. A solution of **8** (12.5 mg, 0.02 mmol, 1.0 equiv) in Et_2O (0.2 mL) was added dropwise at -78 °C. The reaction mixture was warmed to -20 °C and stirred for 30 min. The reaction mixture was cooled to -78 °C and a solution of $ZnBr_2$ (14.9 mg, 0.07 mmol, 3.0 equiv) in Et_2O (0.1 mL) was added dropwise. The reaction mixture was stirred at room temperature for 3 h. The reaction was quenched at 0 °C by addition of a saturated solution of NH_4Cl. The reaction mixture was stirred at room temperature for 3 h. Then, a saturated solution of Na_2CO_3 was added and the aqueous phase was extracted with EtOAc (3 x). The combined organic phases were dried over anhydrous Na_2SO_4, filtered and concentrated under reduced pressure. The reaction mixture was then purified by prepTLC (hexane/EtOAc 8/2) to afford **9** (<5% yield) as a white oil.

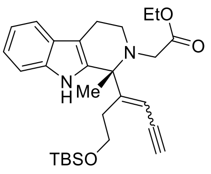

9

ethyl (*R*)-2-(1-(1-((*tert*-butyldimethylsilyl)oxy)hex-3-en-5-yn-3-yl)-1-methyl-1,3,4,9-tetrahydro-2*H*-pyrido[3,4-*b*]indol-2-yl)acetate **9**:

Rf = 0.51 (8/2 Hexane/EtOAc)

^{1}H NMR (800 MHz, $CDCl_3$) δ 7.58 (s, 1H), 7.47 (d, *J* = 7.7 Hz, 1H), 7.27-7.25 (m, 1H), 7.14 (t, *J* = 7.5 Hz, 1H), 7.08 (t, *J* = 7.4 Hz, 1H), 5.87 (s, 1H), 4.18 (q, *J* = 7.1 Hz, 2H), 3.54 (td, *J* = 9.8, 6.6 Hz, 1H), 3.30 (d, J = 16.5 Hz, 1H), 3.24 (td, *J* = 9.8, 5.3 Hz, 1H), 3.21 (s, 1H), 3.14 (d, *J* = 16.5 Hz, 1H), 3.16 – 3.12 (m, 1H), 2.99 – 2.93 (m, 2H), 2.77 – 2.70 (m, 2H), 2.46 (td, *J* = 11.1, 6.2 Hz, 1H), 1.47 (s, 3H), 1.28 (t, *J* = 7.1 Hz, 3H), 0.76 (s, 9H), -0.10 (s, 3H), -0.14 (s, 3H).

^{13}C NMR (201 MHz, $CDCl_3$) δ 171.6, 158.0, 136.5, 136.0, 127.4, 122.0, 119.6, 118.5, 111.0, 109.8, 109.4, 83.4, 81.0, 62.7, 62.0, 60.7, 52.0, 45.3, 34.4, 26.1, 21.6, 18.5, 18.3, 14.4, -5.2, -5.3.

HRMS (APCI/QTOF) m/z: $[M + H]^+$ Calcd for $C_{28}H_{41}N_2O_3Si^+$ 481.2881; Found 481.2872.

IR (ν_{max}, cm^{-1}) 3854 (s), 3744 (s), 3734 (s), 3726 (s), 3628 (s), 2952 (s), 2927 (s), 2852 (s), 1749 (s), 1733 (s), 1716 (s), 1655 (m), 1558 (s), 1471 (s), 1457 (s), 1254 (s), 1189 (s), 1175 (s), 1093 (s), 1080 (s), 913 (s), 835 (s), 778 (s), 742 (s), 672 (s).

3) Synthesis and characterization data of the starting materials

General procedure 1 for the synthesis of starting materials **1a-1d, 1g-1i, 1k-1ae**:

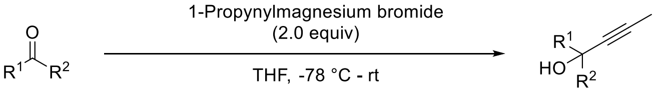

To a solution of the ketone (1.0 equiv) in dry THF [0.1 M] at -78 °C was added 1-propynylmagnesium bromide (2.0 equiv, [0.5M]) dropwise. The reaction mixture was slowly warmed to room temperature and stirred for 2 hours. The reaction mixture was poured into a cold saturated solution of NH_4Cl. The aqueous phase was extracted three times with Et_2O. The combined organic layers were washed with brine, dried over Na_2SO_4, filtered, and concentrated *in vacuo.* The crude product was used in the subsequent step without further purification.

General procedure 2 for the synthesis of starting materials **10a-10e, 10g, 10i-10j, 10r-10u, 10x-10y, 10aa-10ae**:

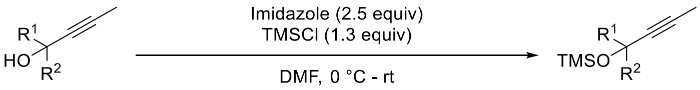

To a solution of the crude propargylic alcohol (1.0 equiv) in dry DMF [0.2 M] was added imidazole (2.5 equiv). The reaction mixture was cooled to 0 °C and TMSCl (1.3 equiv) was added dropwise. The reaction mixture was then warmed to room temperature and stirred for the indicated time. The reaction mixture was then quenched with water. The reaction mixture was extracted three times with EtOAc. The combined organic layers were washed with brine, dried over Na_2SO_4, filtered, and evaporated *in vacuo*. The crude product was then purified by flash column chromatography on silica gel (pentane 100% or pentane/Et_2O 9/1 or pentane/EtOAc 1/1 depending on the polarity) to afford the desired protected alcohol.

General procedure 3 for the synthesis of starting materials **10h, 10k-10q, 10v-10w, 10z**:

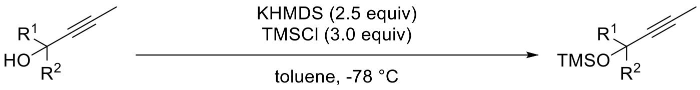

To a solution of the crude propargylic alcohol (1.0 equiv) in dry toluene [0.1 M] was added dropwise KHMDS (2.5 equiv, [1 M] in THF) at -78 °C. The reaction mixture was stirred at this temperature for 30 minutes. TMSCl (3.0 equiv) was added dropwise and the reaction mixture was stirred at -78 °C for the indicated time. The reaction mixture was then warmed to room temperature and stirred for the indicated time. The reaction mixture was then quenched with NH_4Cl at -78 °C and warmed to room temperature. The reaction mixture was extracted three times with EtOAc. The combined organic layers were washed with brine, dried over Na_2SO_4, filtered, and evaporated *in vacuo*. The crude product was then purified by flash column chromatography on silica gel (pentane 100% or pentane/Et_2O 9/1 or pentane/EtOAc 1/1 depending on the polarity) to afford the desired protected alcohol.

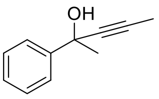

1a

2-phenylpent-3-yn-2-ol **1a**:

Compound **1a** was prepared according to general procedure 1. The crude product was used in the subsequent step without further purification.

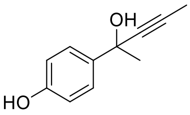

1b

4-(2-hydroxypent-3-yn-2-yl)phenol **1b**:

Compound **1b** was prepared according to general procedure 1 with 3.0 equivalents of 1-propynylmagnesium bromide and pH was adjusted to 4 with 1M HCl during workup. The crude product was used in the subsequent step without further purification.

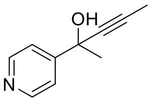

1c

2-(pyridin-4-yl)pent-3-yn-2-ol **1c**:

Compound **1c** was prepared according to general procedure 1. The crude product was used in the subsequent step without further purification.

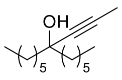

1d

7-(prop-1-yn-1-yl)tridecan-7-ol **1d**:

Compound **1d** was prepared according to general procedure 1. The crude product was used in the subsequent step without further purification.

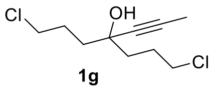

1g

7-chloro-4-(3-chloropropyl)hept-2-yn-4-ol **1g**:

Compound **1g** was prepared according to general procedure 1. The crude product was used in the subsequent step without further purification.

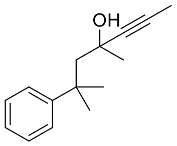

1h

4,6-dimethyl-6-phenylhept-2-yn-4-ol **1h**:

Compound **1h** was prepared according to general procedure 1. The crude product was used in the subsequent step without further purification.

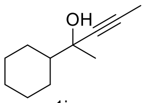

1i

2-cyclohexylpent-3-yn-2-ol **1i**:

Compound **1i** was prepared according to general procedure 1. The crude product was used in the subsequent step without further purification.

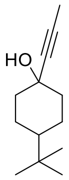

1k

4-(*tert*-butyl)-1-(prop-1-yn-1-yl)cyclohexan-1-ol **1k**:

Compound **1k** was prepared according to general procedure 1. The crude product was used in the subsequent step without further purification.

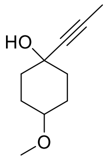

1l

4-methoxy-1-(prop-1-yn-1-yl)cyclohexan-1-ol **1l**:

Compound **1l** was prepared according to general procedure 1. The crude product was used in the subsequent step without further purification.

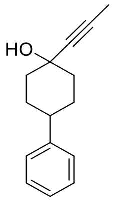

1m

4-phenyl-1-(prop-1-yn-1-yl)cyclohexan-1-ol **1m**:

Compound **1m** was prepared according to general procedure 1. The crude product was used in the subsequent step without further purification.

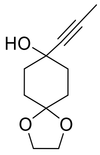

1n

8-(prop-1-yn-1-yl)-1,4-dioxaspiro[4.5]decan-8-ol **1n**:

Compound **1n** was prepared according to general procedure 1. The crude product was used in the subsequent step without further purification.

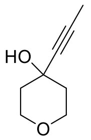

1o

4-(prop-1-yn-1-yl)tetrahydro-2*H*-pyran-4-ol **1o**:

Compound **1o** was prepared according to general procedure 1. The crude product was used in the subsequent step without further purification.

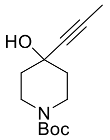

1p

tert-butyl 4-hydroxy-4-(prop-1-yn-1-yl)piperidine-1-carboxylate **1p**:

Compound **1p** was prepared according to general procedure 1. The crude product was used in the subsequent step without further purification.

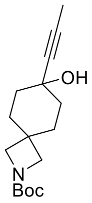

1q

tert-butyl 7-hydroxy-7-(prop-1-yn-1-yl)-2-azaspiro[3.5]nonane-2-carboxylate **1q**:

Compound **1q** was prepared according to general procedure 1. The crude product was used in the subsequent step without further purification.

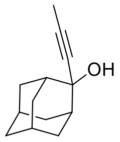

1r

(1*r*,3*r*,5*r*,7*r*)-2-(prop-1-yn-1-yl)adamantan-2-ol **1r**:

Compound **1r** was prepared according to general procedure 1. The crude product was used in the subsequent step without further purification.

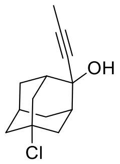

1s

(1*R*,3*S*,5*s*,7*s*)-5-chloro-2-(prop-1-yn-1-yl)adamantan-2-ol **1s**:

Compound **1s** was prepared according to general procedure 1. The crude product was used in the subsequent step without further purification.

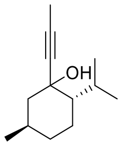

1t

(2*S*,5*R*)-2-isopropyl-5-methyl-1-(prop-1-yn-1-yl)cyclohexan-1-ol **1t**:

Compound **1t** was prepared according to general procedure 1. The crude product was used in the subsequent step without further purification.

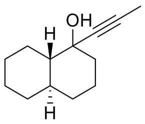

1u

(4a*R*,8a*S*)-1-(prop-1-yn-1-yl)decahydronaphthalen-1-ol **1u**:

Compound **1u** was prepared according to general procedure 1. The crude product was used in the subsequent step without further purification.

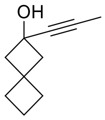

1v

2-(prop-1-yn-1-yl)spiro[3.3]heptan-2-ol **1v**:

Compound **1v** was prepared according to general procedure 1. The crude product was used in the subsequent step without further purification.

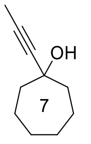

1w

1-(prop-1-yn-1-yl)cycloheptan-1-ol **1w**:

Compound **1w** was prepared according to general procedure 1. The crude product was used in the subsequent step without further purification.

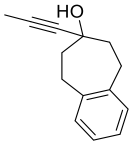

1x

7-(prop-1-yn-1-yl)-6,7,8,9-tetrahydro-5H-benzo[7]annulen-7-ol **1x**:

Compound **1x** was prepared according to general procedure 1. The crude product was used in the subsequent step without further purification.

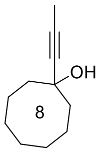

1y

1-(prop-1-yn-1-yl)cyclooctan-1-ol **1y**:

Compound **1y** was prepared according to general procedure 1. The crude product was used in the subsequent step without further purification.

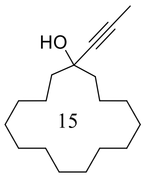

1z

1-(prop-1-yn-1-yl)cyclopentadecan-1-ol **1z**:

Compound **1z** was prepared according to general procedure 1. The crude product was used in the subsequent step without further purification.

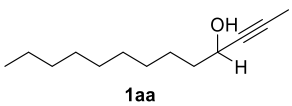

1aa

tridec-2-yn-4-ol **1aa**:

Compound **1aa** was prepared according to general procedure 1. The crude product was used in the subsequent step without further purification.

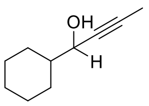

1ab

1-cyclohexylbut-2-yn-1-ol **1ab**:

Compound **1ab** was prepared according to general procedure 1. The crude product was used in the subsequent step without further purification.

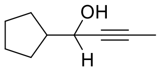

1ac

1-cyclopentylbut-2-yn-1-ol **1ac**:

Compound **1ac** was prepared according to general procedure 1. The crude product was used in the subsequent step without further purification.

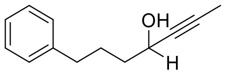

1ad

7-phenylhept-2-yn-4-ol **1ad**:

Compound **1ad** was prepared according to general procedure 1. The crude product was used in the subsequent step without further purification.

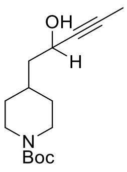

1ae

tert-butyl 4-(2-hydroxypent-3-yn-1-yl)piperidine-1-carboxylate **1ae**:

Compound **1ae** was prepared according to general procedure 1. The crude product was used in the subsequent step without further purification.

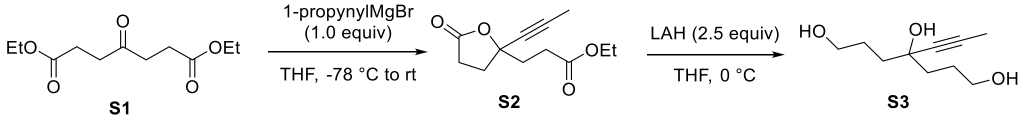

To a solution of diethyl 4-oxoheptanedioate (0.643 mL, 3 mmol, 1.0 equiv) in dry THF (30 mL) at -78 °C was added 1-propynylmagnesium bromide (6 mL, 3 mmol, 1.0 equiv, [0.5M] in THF) dropwise. The reaction mixture was slowly warmed to room temperature and stirred for 2 hours. The reaction mixture was poured into a cold saturated solution of NH_4Cl. The aqueous phase was extracted three times with EtOAc. The combined organic layers were washed with brine, dried over Na_2SO_4, filtered, and concentrated *in vacuo.* The crude **S2** was used in the subsequent step without further purification.

To a solution of $LiAlH_4$ (2 mL, 2.0 mmol, 2.5 equiv, [1 M] in THF) in dry THF (4 mL) was added a solution of **S2** (179 mg, 0.8 mmol, 1.0 equiv) in Et_2O (minimum amount) dropwise at 0 °C. The reaction mixture was stirred at room temperature for 2 hours. The reaction mixture was diluted with Et_2O and a Fieser workup was done. The crude **S3** was used in the subsequent step without further purification.

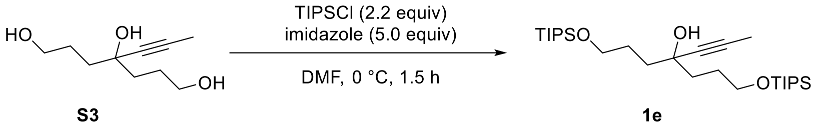

To a solution of the crude **S3** (182.5 mg, 0.98 mmol, 1.0 equiv) in dry DMF (4.9 mL) was added imidazole (320 mg, 4.9 mmol, 5.0 equiv). The reaction mixture was cooled to 0 °C and TIPSCl (0.46 mL, 2.16 mmol, 2.2 equiv) was added dropwise. The reaction mixture was then warmed to room temperature and stirred for 1.5 hours. The reaction mixture was then quenched with water. The reaction mixture was extracted three times with EtOAc. The combined organic layers were washed with brine, dried over Na_2SO_4, filtered, and evaporated *in vacuo*. The crude product was then purified by flash column chromatography on silica gel (pentane/EtOAc 7/3) to afford the desired product **1e** (325 mg, 0.74 mmol, 67% yield over 2 steps) as a colorless oil.

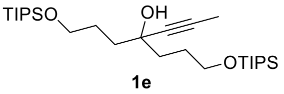

3,3,13,13-tetraisopropyl-2,14-dimethyl-8-(prop-1-yn-1-yl)-4,12-dioxa-3,13-disilapentadecan-8-ol **1e**:

Rf = 0.76 (4/6 EtOAc/hexane).

^{1}H NMR (400 MHz, $CDCl_3$) δ 3.80 (s, 1H), 3.80 – 3.68 (m, 4H), 1.81 (s, 3H), 1.91 – 1.62 (m, 8H), 1.17 – 0.95 (m, 42H).

^{13}C NMR (101 MHz, $CDCl_3$) δ 82.6, 80.1, 70.5, 64.0, 39.8, 28.4, 18.1, 12.1, 3.7.

HRMS (APCI/QTOF) m/z: $[M + Na]^+$ Calcd for $C_{28}H_{58}NaO_3Si_2^+$ 521.3817; Found 521.3821.

IR (ν_{max}, cm^{-1}) 2956 (m), 2945 (s), 2923 (s), 2891 (m), 2868 (s), 1464 (m), 1387 (w), 1248 (w), 1102 (s), 1069 (m), 1010 (m), 996 (m), 882 (s), 791 (m), 725 (m), 677 (s).

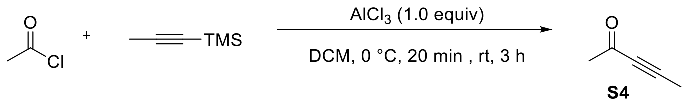

Compound **S4** (443 mg, 5.4 mmol, 54% yield) was prepared according to a literature procedure.[1] All characterization data were in full agreement with those reported in the literature.

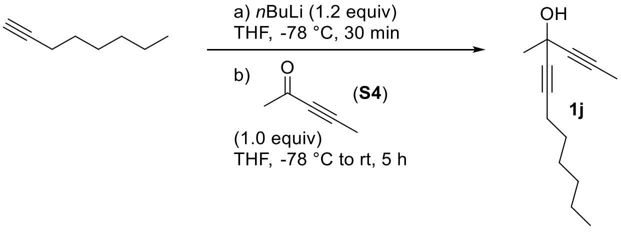

To a solution of 1-octyne (0.43 mL, 2.96 mmol, 1.2 equiv) in dry THF (5 mL) at – 78 °C was added dropwise *n*BuLi (1.17 mL, 2.96 mmol, 1.2 equiv). The mixture was stirred at -78 °C for 30 minutes. A solution of **S4** (200 mg, 2.43 mmol, 1.0 equiv) in THF (3 mL) was added dropwise. The reaction mixture was then allowed to warm to room temperature and stirred for an additional 5 hours. The reaction mixture was quenched with

water and extracted three times with EtOAc. The combined organic layers were washed with brine, dried over Na_2SO_4, filtered, and evaporated *in vacuo*. The crude product was purified by flash column chromatography on silica gel (hexane/EtOAc 8/2) to afford the desired product **1j** (283.5 mg, 1.48 mmol, 61% yield over 2 steps) as a yellow liquid.

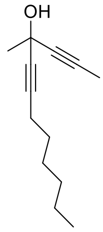

1j

4-methyldodeca-2,5-diyn-4-ol **1j:**

Rf = 0.19 (9/1 Hexane/EtOAc).

^{1}H NMR (400 MHz, $CDCl_3$) δ 2.32 (s, 1H), 2.21 (t, J = 7.2 Hz, 2H), 1.85 (s, 3H), 1.72 (s, 3H), 1.56 – 1.47 (m, 2H), 1.37 (ddt, J = 12.6, 9.8, 5.8 Hz, 3H), 1.33 – 1.20 (m, 5H), 0.89 (t, J = 6.8 Hz, 3H).

^{13}C NMR (151 MHz, $CDCl_3$) δ 83.3, 82.2, 81.4, 78.7, 60.3, 32.6, 31.4, 28.7, 28.5, 22.7, 18.8, 14.2, 3.8.

HRMS (Sicrit plasma/LTQ-Orbitrap) m/z: $[M + H]^+$ Calcd for $C_{13}H_{21}O^+$ 193.1587; Found 193.1588.

IR (ν_{max}, cm^{-1}) 3445 (w), 2989 (w), 2955 (m), 2925 (s), 2858 (m), 1467 (w), 1366 (w), 1229 (m), 1159 (w), 1080 (m), 1017 (w), 906 (m).

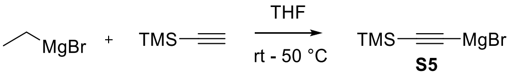

Alkynyl Grignard reagent **S5** was prepared according to the literature procedure.[2] An oven-dried 30 mL re-sealable screw-cap test tube was charged with ethynyltrimethylsilane (0.97 mL, 7 mmol, 1.0 equiv) and THF (7 mL), followed by the slow addition of ethylmagnesium bromide (2.65 mL, 7 mmol, 1.0 equiv) (Caution: exothermic reaction; ethane gas was evolved). The reaction mixture was stirred at room temperature for 15 min to form a clear solution. The reaction mixture was then stirred at 50 °C for 1 hour to afford **S5.** The alkynyl Grignard reagent was titrated before using it in the next steps.

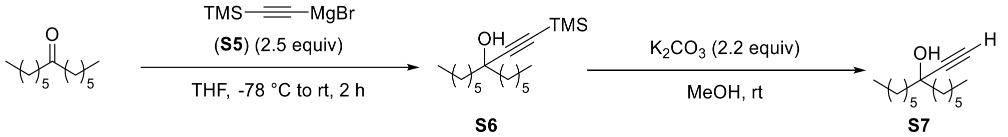

To a solution of the dihexylketone (397 mg, 2 mmol, 1.0 equiv) in dry THF (20 mL) at -78 °C was added **S5** (7.6 mL, 5 mmol, 2.5 equiv, [0.66M]) dropwise. The reaction mixture was slowly warmed to room temperature and stirred for 2 hours. The reaction mixture was poured into a cold saturated solution of NH_4Cl. The aqueous phase was extracted three times with EtOAc. The combined organic layers were washed with brine, dried over Na_2SO_4, filtered, and concentrated *in vacuo.* The crude product **S6** was used in the subsequent step without further purification.

To a solution of the **S6** (585 mg, 1.97 mmol, 1.0 equiv) in MeOH (9.8 mL) at room temperature was added K_2CO_3 (599 mg, 4.33 mmol, 2.2 equiv). The reaction mixture was stirred for 1.5 hours. The reaction mixture was evaporated and diluted with water and Et_2O. The aqueous phase was extracted three times with Et_2O. The combined organic layers were washed with brine, dried over Na_2SO_4, filtered, and concentrated *in vacuo.* The crude product **S7** was used in the subsequent step without further purification.

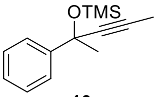

10a

trimethyl((2-phenylpent-3-yn-2-yl)oxy)silane **10a**:

Compound **10a** (171 mg, 0.74 mmol, 98% yield over 2 steps) was prepared according to general procedure 2 for 2 hours.

Yellow liquid.

Rf = 0.65 (8/2 Hexane/EtOAc).

^{1}H NMR (400 MHz, $CDCl_3$) δ 7.60 – 7.57 (m, 2H), 7.34-7.30 (m, 2H), 7.26 – 7.21 (m, 1H), 1.91 (s, 3H), 1.68 (s, 3H), 0.13 (s, 9H).

^{13}C NMR (201 MHz, $CDCl_3$) δ 147.7, 128.0, 127.1, 125.2, 83.3, 81.9, 71.4, 36.1, 3.8, 1.8.

HRMS (APPI/LTQ-Orbitrap) m/z: $[M]^+$ Calcd for $C_{14}H_{20}OSi^+$ 232.1278; Found 232.1281.

IR (ν_{max}, cm^{-1}) 2986 (s), 2970 (s), 2910 (m), 1653 (s), 1559 (s), 1507 (s), 1457 (s), 1251 (m), 1074 (s), 1071 (s), 1057 (s), 844 (m), 698 (s), 695 (s), 671 (s).

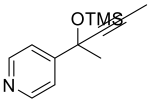

10c

4-(2-((trimethylsilyl)oxy)pent-3-yn-2-yl)pyridine **10c**:

Compound **10c** (165.8 mg, 0.71 mmol, 59% yield over 2 steps) was prepared according to general procedure 2 for 4 hours.

Yellow liquid.

Rf = 0.63 (6/4 Hexane/EtOAc).

^{1}H NMR (400 MHz, $CDCl_3$) δ 8.57 – 8.54 (m, 2H), 7.49 – 7.47 (m, 2H), 1.91 (s, 3H), 1.64 (s, 3H), 0.16 (s, 9H).

^{13}C NMR (126 MHz, $CDCl_3$) δ 156.6, 149.8, 120.2, 82.8, 82.0, 70.5, 35.6, 3.7, 1.7.

HRMS (ESI/QTOF) m/z: $[M + H]^+$ Calcd for $C_{13}H_{20}NOSi^+$ 234.1309; Found 234.1310.

IR (ν_{max}, cm^{-1}) 2985 (w), 2958 (w), 2930 (w), 1594 (w), 1559 (w), 1408 (w), 1367 (w), 1249 (s), 1190 (w), 1105 (m), 1082 (m), 1036 (w), 993 (s), 860 (s), 843 (s), 823 (m), 755 (w).

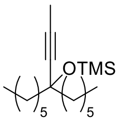

10d

trimethyl((7-(prop-1-yn-1-yl)tridecan-7-yl)oxy)silane **10d**:

Compound **10d** (294 mg, 0.95 mmol, 95% yield over 2 steps) was prepared according to general procedure 2 for 2 hours.

Colorless oil.

Rf = 0.82 (98/2 Hexane/Et_2O).

^{1}H NMR (400 MHz, $CDCl_3$) δ 1.83 (s, 3H), 1.57-1.52 (m, 4H), 1.43-1.35 (m, 4H), 1.34-1.24 (m, 12H), 0.90 – 0.87 (m, 6H), 0.15 (s, 9H).

^{13}C NMR (101 MHz, $CDCl_3$) δ 83.2, 80.8, 72.9, 43.3, 32.1, 29.7, 24.5, 22.8, 14.3, 3.6, 2.1.

HRMS (nanochip-ESI/LTQ-Orbitrap) m/z: $[M + Ag]^+$ Calcd for $C_{19}H_{38}AgOSi^+$ 417.1737; Found 417.1729.

IR (ν_{max}, cm^{-1}) 2952 (m), 2918 (w), 2865 (w), 1248 (w), 1072 (w), 840 (w).

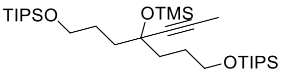

10e

3,3,13,13-tetraisopropyl-2,14-dimethyl-8-(prop-1-yn-1-yl)-8-((trimethylsilyl)oxy)-4,12-dioxa-3,13-disilapentadecane **10e**:

Compound **10e** (159 mg, 0.28 mmol, 70% yield over 2 steps) was prepared according to general procedure 2 for 4 hours.

Light yellow oil.

Rf = 0.59 (0.5/9.5 EtOAc/hexane).

^{1}H NMR (400 MHz, $CDCl_3$) δ 3.69 (t, J = 6.0 Hz, 4H), 1.81 (s, 3H), 1.73-1.61 (m, 8H), 1.13-1.02 (m, 42H), 0.15 (s, 9H).

^{13}C NMR (101 MHz, $CDCl_3$) δ 82.8, 81.1, 72.6, 63.8, 39.6, 28.4, 18.2, 12.2, 3.6, 2.0.

HRMS (ESI/QTOF) m/z: $[M + Na]^+$ Calcd for $C_{31}H_{66}NaO_3Si_3^+$ 593.4212; Found 593.4222.

IR (ν_{max}, cm^{-1}) 2945 (s), 2926 (s), 2897 (m), 2866 (s), 1463 (m), 1247 (m), 1103 (s), 1060 (s), 885 (m), 837 (m), 741 (m), 681 (s), 663 (m).

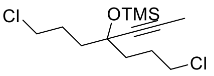

10g

((7-chloro-4-(3-chloropropyl)hept-2-yn-4-yl)oxy)trimethylsilane **10g**:

Compound **10g** (1158 mg, 3.92 mmol, 98% yield over 2 steps) was prepared according to general procedure 2 for 2 hours.

Yellow oil.

Rf = 0.73 (2/8 EtOAc/hexane).

^{1}H NMR (400 MHz, $CDCl_3$) δ 3.62 – 3.52 (m, 4H), 1.97-1.90 (m, 4H), 1.84 (s, 3H), 1.76 – 1.67 (m, 4H), 0.16 (s, 9H).

^{13}C NMR (101 MHz, $CDCl_3$) δ 82.1, 81.8, 71.7, 45.6, 40.7, 28.1, 3.6, 2.0.

HRMS (Sicrit plasma/LTQ-Orbitrap) m/z: $[M + H]^+$ Calcd for $C_{13}H_{25}Cl_2OSi^+$ 295.1046; Found 295.1044.

IR (ν_{max}, cm^{-1}) 2959 (m), 2920 (w), 2875 (w), 1445 (w), 1307 (w), 1277 (w), 1248 (m), 1091 (m), 1039 (m), 875 (w), 841 (s), 747 (w).

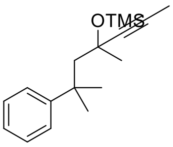

10h

((4,6-dimethyl-6-phenylhept-2-yn-4-yl)oxy)trimethylsilane **10h**:

Compound **10h** (212 mg, 0.74 mmol, 74% yield over 2 steps) was prepared according to general procedure 3 for 1.5 hours.

Yellow liquid.

Rf = 0.66 (2/8 EtOAc/hexane).

^{1}H NMR (400 MHz, $CDCl_3$) δ 7.39 – 7.36 (m, 2H), 7.29 – 7.25 (m, 2H), 7.14 – 7.10 (m, 1H), 2.12 (d, *J* = 14.4 Hz, 1H), 2.04 (d, *J* = 14.4 Hz, 1H), 1.63 (s, 3H), 1.44 (s, 3H), 1.42 (s, 3H), 1.20 (s, 3H), 0.09 (s, 9H).

^{13}C NMR (151 MHz, $CDCl_3$) δ 150.5, 127.8, 126.3, 125.2, 84.3, 81.0, 69.2, 57.0, 37.9, 33.7, 30.8, 30.2, 3.6, 2.0.

HRMS (Sicrit plasma/LTQ-Orbitrap) m/z: $[M + H]^+$ Calcd for $C_{18}H_{29}OSi^+$ 289.1982; Found 289.1983.

IR (ν_{max}, cm^{-1}) 2960 (m), 2916 (w), 1442 (w), 1248 (s), 1073 (m), 1017 (m), 838 (s), 762 (m), 698 (m).

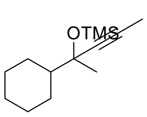

10i

((2-cyclohexylpent-3-yn-2-yl)oxy)trimethylsilane **10i**:

Compound **10i** (206 mg, 0.86 mmol, 86% yield over 2 steps) was prepared according to general procedure 2 for 4.5 hours.

Pale yellow liquid.

Rf = 0.68 (98/2 Hexane/EtOAc).

^{1}H NMR (400 MHz, $CDCl_3$) δ 1.96-1.89 (m, 1H), 1.83 (s, 3H), 1.85 – 1.72 (m, 3H), 1.67 – 1.60 (m, 1H), 1.37 – 1.29 (m, 1H), 1.35 (s, 3H), 1.26-0.99 (m, 5H), 0.15 (s, 9H).

^{13}C NMR (201 MHz, $CDCl_3$) δ 83.0, 80.7, 72.7, 50.1, 28.9, 27.9, 27.7, 26.6, 26.6, 3.6, 2.0.

HRMS (ESI/QTOF) m/z: $[M + Ag]^+$ Calcd for $C_{14}H_{26}AgOSi^+$ 345.0798; Found 345.0794.

IR (ν_{max}, cm^{-1}) 2925 (m), 2843 (w), 2347 (m), 917 (s), 745 (s).

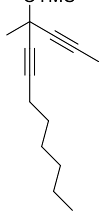

10j

trimethyl((4-methyldodeca-2,5-diyn-4-yl)oxy)silane **10j**:

Compound **10j** (288 mg, 1.09 mmol, 74% yield) was prepared according to general procedure 2 for 3 hours.

Yellow liquid.

Rf = 0.63 (9/1 Hexane/EtOAc).

^{1}H NMR (400 MHz, $CDCl_3$) δ 2.20 (t, *J* = 7.1 Hz, 2H), 1.84 (s, 3H), 1.70 (s, 3H), 1.53 – 1.47 (m, 2H), 1.43-1.23 (m, 6H), 0.89 (t, *J* = 6.8 Hz, 3H), 0.23 (s, 9H).

^{13}C NMR (151 MHz, $CDCl_3$) δ 83.1, 82.9, 82.2, 78.4, 61.3, 35.1, 31.5, 28.7, 28.5, 22.7, 18.9, 14.2, 3.8, 1.7.

HRMS (Sicrit plasma/LTQ-Orbitrap) m/z: $[M + H]^+$ Calcd for $C_{16}H_{29}OSi^+$ 265.1982; Found 265.1982.

IR (ν_{max}, cm^{-1}) 2992 (w), 2959 (m), 2931 (m), 2866 (w), 1734 (w), 1249 (m), 1233 (m), 1170 (w), 1092 (s), 1036 (w), 988 (m), 860 (m), 843 (s), 754 (w).

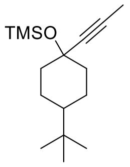

10k

((4-(*tert*-butyl)-1-(prop-1-yn-1-yl)cyclohexyl)oxy)trimethylsilane **10k**:

Compound **10k** (213 mg, 0.8 mmol, 80% yield over 2 steps) was prepared according to general procedure 3 for 30 minutes.

Pale yellow oil.

Rf = 0.5 (Hexane 100%).

^{1}H NMR (400 MHz, $CDCl_3$) δ 1.97 – 1.91 (m, 2H), 1.85 (s, 3H), 1.69-1.62 (m, 2H), 1.45 (td, *J* = 12.5, 3.1 Hz, 2H), 1.34 (td, *J* = 12.5, 2.8 Hz, 2H), 0.98 – 0.90 (m, 1H), 0.85 (s, 9H), 0.17 (s, 9H).

^{13}C NMR (101 MHz, $CDCl_3$) δ 82.8, 81.8, 71.4, 47.2, 42.0, 32.4, 27.8, 24.9, 3.7, 2.2.

HRMS (ESI/QTOF) m/z: $[M + Ag]^+$ Calcd for $C_{16}H_{30}AgOSi^+$ 373.1111; Found 373.1107.

IR (ν_{max}, cm^{-1}) 2956 (m), 2941 (m), 2908 (w), 2861 (w), 1449 (w), 1365 (w), 1260 (w), 1252 (m), 1248 (m), 1091 (s), 1019 (w), 999 (w), 895 (m), 876 (s), 840 (s), 816 (w), 754 (w).

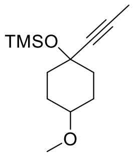

10l

((4-methoxy-1-(prop-1-yn-1-yl)cyclohexyl)oxy)trimethylsilane **10l**:

Compound **10l** (156 mg, 0.65 mmol, 65% yield over 2 steps) was prepared according to general procedure 3 for 1 hour.

Colorless liquid.

d.r. 1.5:1.

Rf = 0.76 (2/8 EtOAc/hexane).

^{1}H NMR (400 MHz, $CDCl_3$) δ 3.33, 3.31 (two s, 3H), 3.24-3.12 (m, 1H), 1.94 – 1.85 (m, 3H), 1.84, 1.83 (two s, 3H), 1.74-1.67 (m, 2H), 1.64-1.52 (m, 3H), 0.17, 0.16 (two s, 9H).

^{13}C NMR (101 MHz, $CDCl_3$) δ 83.4 (major), 82.7 (minor), 81.9 (minor), 80.3 (major), 77.6, 76.4, 69.8, 68.6, 56.0 (minor), 55.7 (major), 38.5 (minor), 37.5 (major), 28.2 (minor), 27.1 (major), 3.7 (minor), 3.6 (major), 2.0 (minor), 2.0 (major).

HRMS (Sicrit plasma/LTQ-Orbitrap) m/z: $[M + H]^+$ Calcd for $C_{13}H_{25}O_2Si^+$ 241.1618; Found 241.1611.

IR (ν_{max}, cm^{-1}) 2944 (w), 1248 (m), 1090 (s), 1049 (m), 1017 (m), 881 (m), 837 (s), 754 (m).

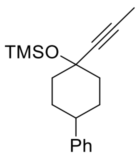

10m

trimethyl((4-phenyl-1-(prop-1-yn-1-yl)cyclohexyl)oxy)silane **10m**:

Compound **10m** (73 mg, 0.26 mmol, 51% yield over 2 steps) was prepared according to general procedure 3 for 2 hours.

Yellow oil.

Rf = 0.82 (2/8 EtOAc/Hexane).

^{1}H NMR (400 MHz, $CDCl_3$) δ 7.32 – 7.27 (m, 2H), 7.25 – 7.21 (m, 2H), 7.21-7.16 (m, 1H), 2.52 – 2.43 (m, 1H), 2.06-1.99 (m, 2H), 1.91 (s, 3H), 1.86 – 1.79 (m, 4H), 1.69 – 1.61 (m, 2H), 0.21 (s, 9H).

^{13}C NMR (101 MHz, $CDCl_3$) δ 147.0, 128.5, 127.0, 126.1, 82.6, 82.3, 70.8, 43.5, 41.9, 31.7, 3.8, 2.2.

HRMS (Sicrit plasma/LTQ-Orbitrap) m/z: $[M + H]^+$ Calcd for $C_{18}H_{27}OSi^+$ 287.1826; Found 287.1825.

IR (ν_{max}, cm^{-1}) 2937 (m), 2858 (w), 1446 (w), 1249 (m), 1089 (s), 879 (s), 840 (s), 755 (m), 699 (m).

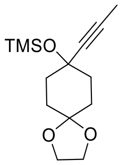

10n

trimethyl((8-(prop-1-yn-1-yl)-1,4-dioxaspiro[4.5]decan-8-yl)oxy)silane **10n**:

Compound **10n** (129 mg, 0.48 mmol, 96% yield over 2 steps) was prepared according to general procedure 3 for 2.5 hours.

Yellow oil.

Rf = 0.64 (2/8 EtOAc/Hexane).

^{1}H NMR (400 MHz, $CDCl_3$) δ 3.93 (s, 4H), 1.91 – 1.80 (m, 4H), 1.83 (s, 3H), 1.83 – 1.66 (m, 4H), 0.17 (s, 9H).

^{13}C NMR (101 MHz, $CDCl_3$) δ 108.4, 82.8, 80.9, 68.6 (br), 64.4, 64.3, 38.6, 31.4, 3.6, 2.0.

HRMS (Sicrit plasma/LTQ-Orbitrap) m/z: $[M + H]^+$ Calcd for $C_{14}H_{25}O_3Si^+$ 269.1567; Found 269.1568.

IR (ν_{max}, cm^{-1}) 2956 (m), 2932 (m), 1249 (m), 1104 (s), 1044 (m), 1017 (m), 929 (m), 885 (m), 841 (s).

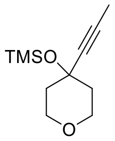

10o

trimethyl((4-(prop-1-yn-1-yl)tetrahydro-2*H*-pyran-4-yl)oxy)silane **10o**:

Compound **10o** (156 mg, 0.74 mmol, 74% yield over 2 steps) was prepared according to general procedure 3 for 1 hour.

Pale yellow liquid.

Rf = 0.72 (2/8 EtOAc/hexane).

^{1}H NMR (400 MHz, $CDCl_3$) δ 3.84 – 3.79 (m, 2H), 3.63 (ddd, *J* = 11.5, 8.4, 3.1 Hz, 2H), 1.86 (s, 3H), 1.85-1.79 (m, 2H), 1.73 (ddd, *J* = 12.7, 8.7, 4.0 Hz, 2H), 0.18 (s, 9H).

^{13}C NMR (101 MHz, $CDCl_3$) δ 82.3, 81.9, 67.2, 64.9, 41.6, 3.6, 2.0.

HRMS (Sicrit plasma/LTQ-Orbitrap) m/z: $[M + H]^+$ Calcd for $C_{11}H_{21}O_2Si^+$ 213.1305; Found 213.1298.

IR (ν_{max}, cm^{-1}) 2957 (w), 2856 (w), 1249 (m), 1098 (s), 1045 (m), 1007 (m), 900 (m), 869 (m), 837 (s), 754 (m).

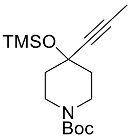

10p

tert-butyl 4-(prop-1-yn-1-yl)-4-((trimethylsilyl)oxy)piperidine-1-carboxylate **10p**:

Compound **10p** (251.4 mg, 0.81 mmol, 81% yield over 2 steps) was prepared according to general procedure 3 for 1 hour.

Yellow liquid.

Rf = 0.78 (3/7 EtOAc/hexane).

^{1}H NMR (400 MHz, $CDCl_3$) δ 3.66-3.56 (m, 2H), 3.29 (ddd, *J* = 13.3, 8.6, 3.6 Hz, 2H), 1.85 (s, 3H), 1.80 – 1.69 (m, 2H), 1.69 – 1.59 (m, 2H), 1.45 (s, 9H), 0.17 (s, 9H).

^{13}C NMR (101 MHz, $CDCl_3$) δ 154.9, 82.1, 82.1, 79.5, 68.0, 40.5, 28.6, 3.6, 2.0.

HRMS (Sicrit plasma/LTQ-Orbitrap) m/z: $[M + H]^+$ Calcd for $C_{16}H_{30}NO_3Si^+$ 312.1989; Found 312.1974.

IR (ν_{max}, cm^{-1}) 1696 (s), 1417 (m), 1245 (s), 1161 (s), 1092 (s), 1041 (m), 890 (m), 866 (m), 839 (s), 755 (m).

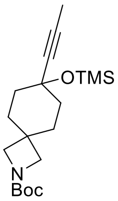

10q

tert-butyl 7-(prop-1-yn-1-yl)-7-((trimethylsilyl)oxy)-2-azaspiro[3.5]nonane-2-carboxylate **10q**:

Compound **10q** (319 mg, 0.91 mmol, 91% yield over 2 steps) was prepared according to general procedure 3 for 2 hours.

Yellow solid.

Rf = 0.75 (1/1 EtOAc/hexane).

m.p.: 85.9 – 86.4 °C.

^{1}H NMR (400 MHz, $CDCl_3$) δ 3.57 (s, 4H), 1.84 (s, 3H), 1.81 – 1.64 (m, 6H), 1.62-1.51 (m, 2H), 1.43 (s, 9H), 0.16 (s, 9H).

^{13}C NMR (151 MHz, $CDCl_3$) δ 156.7, 82.6 (by HMBC), 81.5, 79.3, 68.8 (by HMBC), 59.9 (by HMBC), 58.7 (by HMBC), 37.8, 34.2, 32.6, 28.6, 3.6, 2.0.

HRMS (Sicrit plasma/LTQ-Orbitrap) m/z: $[M + H]^+$ Calcd for $C_{19}H_{34}NO_3Si^+$ 352.2302; Found 352.2302.

IR (ν_{max}, cm^{-1}) 2960 (w), 2930 (w), 2871 (w), 1705 (s), 1402 (s), 1358 (w), 1248 (m), 1137 (m), 1096 (m), 1039 (w), 886 (m), 853 (m), 841 (m).

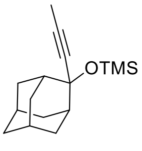

10r

trimethyl(((1*r*,3*r*,5*r*,7*r*)-2-(prop-1-yn-1-yl)adamantan-2-yl)oxy)silane **10r**:

Compound **10r** (231 mg, 0.89 mmol, 89% yield over 2 steps) was prepared according to general procedure 2 for 4.5 hours.

Colorless liquid.

Rf = 0.77 (9/1 Hexane/EtOAc).

^{1}H NMR (600 MHz, $CDCl_3$) δ 2.16-2.08 (m, 4H), 1.86 (s, 3H), 1.82 (t, *J* = 3.1 Hz, 2H), 1.76-1.73 (m, 1H), 1.71 – 1.64 (m, 5H), 1.48 – 1.44 (m, 2H), 0.17 (s, 9H).

^{13}C NMR (151 MHz, $CDCl_3$) δ 84.1, 81.6, 74.0, 40.0, 38.1, 35.3, 32.0, 27.1, 27.0, 3.7, 2.0.

HRMS (Sicrit plasma/LTQ-Orbitrap) m/z: $[M]^+$ Calcd for $C_{16}H_{26}OSi^+$ 262.1747; Found 262.1747.

IR (ν_{max}, cm^{-1}) 2941 (w), 2934 (m), 2901 (s), 2854 (m), 1468 (w), 1449 (w), 1256 (w), 1244 (m), 1105 (m), 1103 (s), 1082 (s), 1061 (s), 1058 (m), 1031 (m), 883 (s), 853 (m), 838 (s), 754 (w).

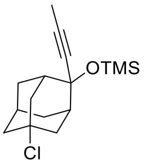

10s

(((1*R*,3*S*,5s,7s)-5-chloro-2-(prop-1-yn-1-yl)adamantan-2-yl)oxy)trimethylsilane **10s**:

Compound **10s** (329 mg, 1.1 mmol, 92% yield over 2 steps) was prepared according to general procedure 2 for 2 hours.

White solid.

d.r. 3.7:1.

Rf = 0.4 (98/2 Hexane/EtOAc).

m.p.: 56.4 – 57.2 °C.

^{1}H NMR (600 MHz, $CDCl_3$) δ 2.60 – 2.53 (m, 2H major + 2H minor), 2.13 – 2.05 (m, 4H major + 4H minor), 2.04 – 1.97 (m, 5H major + 3H minor), 1.88 (s, 3H, major), 1.86 (s, 3H, minor), 1.85 – 1.81 (m, 2H minor), 1.64 – 1.60 (m, 2H, minor), 1.42-1.38 (m, 2H, major), 0.17 (s, 9H, minor), 0.16 (s, 9H, major).

^{13}C NMR (151 MHz, $CDCl_3$) δ 83.0 (major), 82.9 (minor), 82.8 (major), 82.5 (minor), 72.2 (major), 72.0 (minor), 67.7 (minor), 67.5 (major), 48.1 (major), 48.0 (minor), 44.9 (major), 43.5 (minor), 43.0 (major), 42.0 (minor), 33.3 (major), 30.3 (minor), 30.3 (minor), 30.1 (major), 3.7 (major), 3.7 (minor), 1.9 (minor) 1.9 (major).

HRMS (ESI/QTOF) m/z: $[M + H]^+$ Calcd for $C_{16}H_{26}ClOSi^+$ 297.1436; Found 297.1430.

IR (ν_{max}, cm^{-1}) 2948 (m), 2944 (m), 2917 (m), 2855 (w), 1739 (w), 1448 (w), 1248 (m), 1110 (m), 1107 (m), 1085 (s), 1068 (m), 882 (s), 864 (m), 843 (s), 839 (s).

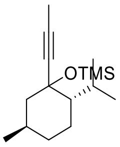

10t

(((2*S*,5*R*)-2-isopropyl-5-methyl-1-(prop-1-yn-1-yl)cyclohexyl)oxy)trimethylsilane **10t**:

Compound **10t** (167 mg, 0.65 mmol, 63% yield over 2 steps) was prepared according to general procedure 2 for 4.5 hours.

d.r. 3.9:1.

Diastereomer major: **10t-1**

Colorless liquid.

Rf = 0.76 (98/2 Hexane/EtOAc).

^{1}H NMR (400 MHz, $CDCl_3$) δ 2.38-2.27 (m, 1H), 1.89 (dt, *J* = 13.4, 2.9 Hz, 1H), 1.83 (s, 3H), 1.74-1.62 (m, 2H), 1.49 – 1.31 (m, 2H), 1.20 (dd, *J* = 13.5, 11.9 Hz, 1H), 1.09 (ddd, *J* = 12.2, 3.8, 1.6 Hz, 1H), 0.90 (d, *J* = 7.0 Hz, 3H), 0.85 (d, *J* = 6.7 Hz, 3H), 0.85 – 0.79 (m, 1H), 0.82 (d, *J* = 6.7 Hz, 3H), 0.14 (s,I9H).

^{13}C NMR (201 MHz, $CDCl_3$) δ 84.6, 80.0, 73.3, 52.1, 51.0, 35.2, 28.4, 27.4, 24.2, 22.2, 20.6, 18.4, 3.7, 1.9.

HRMS (Sicrit plasma/LTQ-Orbitrap) m/z: $[M]^+$ Calcd for $C_{16}H_{30}OSi^+$ 266.2060; Found 266.2059.

IR (ν_{max}, cm^{-1}) 3746 (m), 3628 (w), 2959 (w), 2881 (w), 2362 (m), 2342 (m), 1735 (w), 1360 (w), 1217 (w), 913 (m), 838 (m), 746 (m), 620 (s), 606 (s).

Diastereomer minor: **10t-2**

Pale yellow liquid.

^{1}H NMR (400 MHz, $CDCl_3$) δ 2.23-2.15 (m, 1H), 1.92 – 1.85 (m, 1H), 1.83 (s, 3H), 1.75-1.62 (m, 2H), 1.58 – 1.52 (m, 1H), 1.28 (qd, *J* = 12.9, 3.5 Hz, 1H), 1.18 – 1.11 (m, 2H), 0.90 (d, *J* = 6.7 Hz, 6H), 0.87 (d, *J* = 6.7 Hz, 3H), 0.86 – 0.79 (m, 1H), 0.17 (s, 9H).

^{13}C NMR (201 MHz, $CDCl_3$) δ 83.4, 82.1, j73.7, 54.0, 52.6, 35.0, 30.5, 25.9, 24.3, 23.5, 22.1, 17.8, 3.7, 2.2.

Rf = 0.58 (98/2 Hexane/EtOAc).

HRMS (Sicrit plasma/LTQ-Orbitrap) m/z: $[M]^+$ Calcd for $C_{16}H_{30}OSi^+$ 266.2060; Found 266.2059.

IR (ν_{max}, cm^{-1}) 3626 (m), 3052 (w), 2914 (w), 2347 (m), 1738 (s), 1728 (m), 1356 (m), 1217 (m), 909 (w), 741 (m), 674 (s), 620 (s).

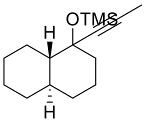

10u

trimethyl(((4aR,8aS)-1-(prop-1-yn-1-yl)decahydronaphthalen-1-yl)oxy)silane **10u**:

Compound **10u** (247 mg, 0.94 mmol, 94% yield over 2 steps) was prepared according to general procedure 2 for 4.5 hours.

Colorless liquid.

dr: 1.1:1.

Diastereomer 1: (**10u-1**)

Rf = 0.79 (9/1 Hexane/EtOAc).

^{1}H NMR (600 MHz, $CDCl_3$) δ 1.95-1.90 (m, 2H), 1.82 (s, 3H), 1.78 – 1.73 (m, 1H), 1.64 – 1.55 (m, 3H), 1.55 – 1.47 (m, 2H), 1.42 – 1.38 (m, 1H), 1.36 – 1.27 (m, 1H), 1.27 – 1.13 (m, 3H), 0.97 – 0.86 (m, 3H), 0.14 (s, 9H).

^{13}C NMR (151 MHz, $CDCl_3$) δ 84.2, 79.8, 70.8, 52.7, 41.8, 36.3, 34.6, 34.0, 27.2, 26.9, 26.6, 20.9, 3.6, 1.8.

HRMS (Sicrit plasma/LTQ-Orbitrap) m/z: $[M + H]^+$ Calcd for $C_{16}H_{29}OSi^+$ 265.1982; Found 265.1982.

IR (ν_{max}, cm^{-1}) 2927 (m), 2854 (w), 1453 (w), 1247 (m), 1189 (w), 1100 (w), 1083 (m), 1061 (s), 1031 (m), 988 (m), 872 (m), 838 (s), 836 (s), 753 (m).

Diastereomer 2: (**10u-2**)

Rf = 0.72 (9/1 Hexane/EtOAc).

^{1}H NMR (400 MHz, $CDCl_3$) δ 1.97 – 1.91 (m, 2H), 1.86 (s, 3H), 1.77-1.72 (m, 1H), 1.65 – 1.56 (m, 3H), 1.52 – 1.45 (m, 2H), 1.30 – 1.09 (m, 4H), 1.04 – 0.84 (m, 4H), 0.16 (s, 9H).

^{13}C NMR (151 MHz, $CDCl_3$) δ 82.9, 81.2, 74.2, 53.7, 42.8, 39.0, 34.2, 33.8, 26.6, 26.4, 26.4, 23.3, 3.7, 2.3.

HRMS (Sicrit plasma/LTQ-Orbitrap) m/z: $[M + H]^+$ Calcd for $C_{16}H_{29}OSi^+$ 265.1982; Found 265.1982.

IR (ν_{max}, cm^{-1}) 2941 (m), 2926 (s), 2857 (m), 1449 (w), 1252 (m), 1248 (m), 1098 (w), 1076 (s), 1075 (s), 1054 (w), 971 (m), 966 (w), 904 (s), 867 (m), 838 (s), 754 (w).

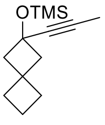

10v

trimethyl((2-(prop-1-yn-1-yl)spiro[3.3]heptan-2-yl)oxy)silane **10v**:

Compound **10v** (220 mg, 0.99 mmol, 99% yield over 2 steps) was prepared according to general procedure 3 for 3 hours.

Yellow oil.

Rf = 0.80 (EtOAc/hexane 1/9).

^{1}H NMR (400 MHz, $CDCl_3$) δ 2.48 – 2.43 (m, 2H), 2.22 – 2.16 (m, 2H), 2.13 – 2.09 (m, 2H), 1.97 – 1.90 (m, 2H), 1.84-1.76 (m, 2H), 1.82 (s, 3H), 0.15 (s, 9H).

^{13}C NMR (101 MHz, $CDCl_3$) δ 84.3, 79.3, 64.8, 52.5, 35.5, 34.6, 17.1, 3.7, 1.5.

HRMS (Sicrit plasma/LTQ-Orbitrap) m/z: $[M + H]^+$ Calcd for $C_{13}H_{23}OSi^+$ 223.1513; Found 223.1513.

IR (ν_{max}, cm^{-1}) 3741 (s), 3692 (m), 2968 (s), 2933 (s), 2904 (s), 1520 (m), 1415 (m), 1250 (m), 1084 (s), 1052 (s), 909 (m), 840 (s), 746 (m).

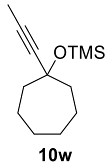

10w

trimethyl((1-(prop-1-yn-1-yl)cycloheptyl)oxy)silane **10w**:

Compound **10w** (104 mg, 0.46 mmol, 66% yield over 2 steps) was prepared according to general procedure 3 for 45 minutes.

Colorless liquid.

Rf = 0.88 (1/9 EtOAc/hexane).

^{1}H NMR (400 MHz, $CDCl_3$) δ 1.92 – 1.85 (m, 2H), 1.84 (s, 3H), 1.82 – 1.75 (m, 2H), 1.63 – 1.43 (m, 8H), 0.16 (s, 9H).

^{13}C NMR (101 MHz, $CDCl_3$) δ 84.7, 80.1, 73.2, 44.7, 28.1, 22.0, 3.7, 2.1.

HRMS (Sicrit plasma/LTQ-Orbitrap) m/z: $[M + H]^+$ Calcd for $C_{13}H_{25}OSi^+$ 225.1669; Found 225.1670.

IR (ν_{max}, cm^{-1}) 2924 (m), 2856 (m), 2361 (s), 2337 (s), 1736 (w), 1062 (w), 841 (w).

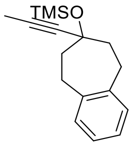

10x

trimethyl((7-(prop-1-yn-1-yl)-6,7,8,9-tetrahydro-5*H*-benzo[7]annulen-7-yl)oxy)silane **10x**:

Compound **10x** (244 mg, 0.9 mmol, 90% yield over 2 steps) was prepared according to general procedure 2 for 2 hours.

White solid.

Rf = 0.57 (9.5/0.5 Hexane/EtOAc).

m.p.: 78.9 – 80.4 °C.

^{1}H NMR (600 MHz, $CDCl_3$) δ 7.08 (brs, 4H), 2.94-2.68 (m, 4H), 2.02-1.90 (m, 2H), 1.87 (s, 3H), 1.87-1.73 (m, 2H), 0.21 (s, 9H). (NMR was performed at 50 °C)

^{13}C NMR (151 MHz, $CDCl_3$) δ 142.9, 129.0, 126.2, 84.0, 81.4, 73.1, 43.0, 30.5, 3.5, 2.1. (NMR was performed at 50 °C)

HRMS (Sicrit plasma/LTQ-Orbitrap) m/z: $[M + H]^+$ Calcd for $C_{17}H_{25}OSi^+$ 273.1669; Found 273.1668.

IR (ν_{max}, cm^{-1}) 3011 (w), 2942 (m), 2922 (w), 2855 (w), 1730 (w), 1456 (w), 1251 (m), 1208 (w), 1077 (s), 1041 (m), 995 (w), 947 (w), 875 (m), 864 (m), 839 (s), 750 (s).

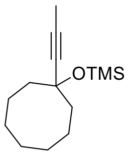

10y

trimethyl((1-(prop-1-yn-1-yl)cyclooctyl)oxy)silane **10y**:

Compound **10y** (203 mg, 0.85 mmol, 85% yield over 2 steps) was prepared according to general procedure 2 for 4.5 hours.

Pale yellow liquid.

Rf = 0.54 (98/2 Hexane/EtOAc).

^{1}H NMR (400 MHz, $CDCl_3$) δ 1.95 – 1.74 (m, 4H), 1.83 (s, 3H), 1.67 – 1.44 (m, 10H), 0.15 (s, 9H).

^{13}C NMR (201 MHz, $CDCl_3$) δ 84.5, 79.7, 72.7, 39.4, 28.1, 24.4, 21.8, 3.5, 1.9.

HRMS (Sicrit plasma/LTQ-Orbitrap) m/z: $[M - H]^+$ Calcd for $C_{14}H_{25}OSi^+$ 237.1669; Found 237.1668.

IR (ν_{max}, cm^{-1}) 2955 (w), 2921 (m), 2844 (w), 1452 (w), 1248 (s), 1088 (m), 1060 (s), 1027 (m), 876 (m), 839 (s), 754 (w).

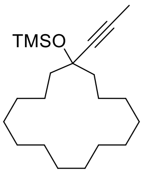

10z

trimethyl((1-(prop-1-yn-1-yl)cyclopentadecyl)oxy)silane **10z**:

Compound **10z** (298 mg, 0.89 mmol, 89% yield over 2 steps) was prepared according to general procedure 3 for 3 hours.

Slightly yellow liquid.

Rf = 0.81 (1/9 EtOAc/pentane).

^{1}H NMR (400 MHz, $CDCl_3$) δ 1.83 (s, 3H), 1.71 – 1.56 (m, 4H), 1.41 – 1.23 (m, 24H), 0.15 (s, 9H).

^{13}C NMR (101 MHz, $CDCl_3$) δ 84.3, 80.2, 72.4, 41.1, 27.6, 27.1, 26.9, 26.9, 26.6, 22.2, 3.6, 2.0.

HRMS (Sicrit plasma/LTQ-Orbitrap) m/z: $[M + H]^+$ Calcd for $C_{21}H_{41}OSi^+$ 337.2921; Found 337.2920.

IR (ν_{max}, cm^{-1}) 2925 (m), 2856 (w), 1457 (w), 1249 (m), 1049 (m), 917 (s), 838 (s), 754 (m), 683 (w).

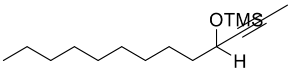

10aa

trimethyl(tridec-2-yn-4-yloxy)silane **10aa**:

Compound **10aa** (160 mg, 0.6 mmol, 60% yield over 2 steps) was prepared according to general procedure 2 for 3 hours.

Pale yellow liquid.

Rf = 0.33 (9.5/0.5 Hexane/EtOAc).

^{1}H NMR (400 MHz, $CDCl_3$) δ 4.28 (tq, J = 6.5, 2.1 Hz, 1H), 1.82 (d, J = 2.1 Hz, 3H), 1.64 – 1.59 (m, 2H), 1.43-1.22 (m, 14H), 0.87 (t, J = 7.0 Hz, 3H), 0.16 (s, 9H).

^{13}C NMR (101 MHz, $CDCl_3$) δ 81.0, 80.2, 63.1, 39.1, 32.1, 29.7, 29.7, 29.5, 29.4, 25.4, 22.8, 14.3, 3.7, 0.3.

HRMS (Sicrit plasma/LTQ-Orbitrap) m/z: $[M + H]^+$ Calcd for $C_{16}H_{33}OSi^+$ 269.2295; Found 269.2294.

IR (ν_{max}, cm^{-1}) 3679 (w), 3651 (w), 2975 (m), 2963 (s), 2923 (s), 2858 (m), 2361 (m), 2164 (m), 1399 (w), 1240 (w), 1092 (m), 1074 (s), 1051 (s), 841 (s).

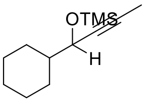

10ab

((1-cyclohexylbut-2-yn-1-yl)oxy)trimethylsilane **10ab**:

Compound **10ab** (206 mg, 0.92 mmol, 99% yield over 2 steps) was prepared according to general procedure 2 for 1 hour.

Colorless oil.

Rf = 0.4 (96/4 Hexane/EtOAc).

^{1}H NMR (400 MHz, $CDCl_3$) δ 4.03 (dq, *J* = 6.4, 2.2 Hz, 1H), 1.89 – 1.78 (m, 2H), 1.83 (d, *J* = 2.1 Hz, 3H), 1.78 – 1.69 (m, 2H), 1.69 – 1.61 (m, 1H), 1.49 – 1.38 (m, 1H), 1.30 – 1.10 (m, 3H), 1.10-0.92 (m, 2H), 0.15 (s, 9H).

^{13}C NMR (101 MHz, $CDCl_3$) δ 80.8, 80.0, 67.8, 45.0, 28.9, 28.5, 26.7, 26.2, 3.7, 0.3.

HRMS (Sicrit plasma/LTQ-Orbitrap) m/z: $[M + H]^+$ Calcd for $C_{13}H_{25}OSi^+$ 225.1669; Found 225.1669.

IR (ν_{max}, cm^{-1}) 2953 (m), 2924 (s), 2853 (m), 1731 (w), 1449 (w), 1369 (w), 1250 (s), 1212 (w), 1102 (m), 1068 (m), 1054 (m), 901 (m), 871 (s), 841 (s), 747 (m).

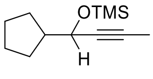

10ac

((1-cyclopentylbut-2-yn-1-yl)oxy)trimethylsilane **10ac**:

Compound **10ac** (189.3 mg, 0.9 mmol, 90% yield over 2 steps) was prepared according to general procedure 2 for 3 hours.

Colorless oil.

Rf = 0.4 (98/2 Hexane/EtOAc).

^{1}H NMR (400 MHz, $CDCl_3$) δ 4.12 (dq, *J* = 6.8, 2.1 Hz, 1H), 2.09 (h, *J* = 7.9 Hz, 1H), 1.82 (d, *J* = 2.1 Hz, 3H), 1.78 – 1.66 (m, 2H), 1.64 – 1.45 (m, 4H), 1.43-1.31 (m, 2H), 0.15 (s, 9H).

^{13}C NMR (101 MHz, $CDCl_3$) δ 80.5, 80.0, 66.8, 47.3, 29.1, 28.6, 25.7, 25.7, 3.7, 0.3.

HRMS (Sicrit plasma/LTQ-Orbitrap) m/z: $[M - H]^+$ Calcd for $C_{12}H_{21}OSi^+$ 209.1356; Found 209.1356.

IR (ν_{max}, cm^{-1}) 2951 (m), 2941 (m), 2867 (w), 2353 (m), 1735 (s), 1717 (s), 1558 (m), 1515 (m), 1365 (m), 1216 (m), 913 (m), 745 (s), 672 (s).

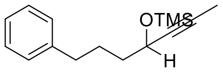

10ad

trimethyl((7-phenylhept-2-yn-4-yl)oxy)silane **10ad**:

Compound **10ad** (202 mg, 0.76 mmol, 76% yield over 2 steps) was prepared according to general procedure 2 for 1.5 hours.

Colorless oil.

Rf = 0.4 (96/4 Hexane/EtOAc).

^{1}H NMR (400 MHz, $CDCl_3$) δ 7.31 – 7.26 (m, 2H), 7.20 – 7.15 (m, 3H), 4.34-4.29 (m, 1H), 2.63 (t, *J* = 7.3 Hz, 2H), 1.82 (d, *J* = 2.1 Hz, 3H), 1.84 – 1.63 (m, 4H), 0.15 (s, 9H).

^{13}C NMR (101 MHz, $CDCl_3$) δ 142.5, 128.6, 128.4, 125.8, 80.8, 80.4, 62.9, 38.6, 35.7, 27.2, 3.7, 0.3.

HRMS (Sicrit plasma/LTQ-Orbitrap) m/z: $[M + H]^+$ Calcd for $C_{16}H_{25}OSi^+$ 261.1669; Found 261.1669.

IR (ν_{max}, cm^{-1}) 3063 (w), 3029 (w), 2948 (w), 2920 (w), 2861 (w), 1496 (w), 1453 (w), 1347 (w), 1250 (m), 1091 (m), 1079 (m), 1025 (w), 877 (m), 838 (s), 750 (m), 699 (m).

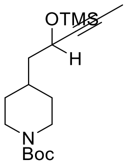

10ae

tert-butyl 4-(2-((trimethylsilyl)oxy)pent-3-yn-1-yl)piperidine-1-carboxylate **10ae**:

Compound **10ae** (152.5 mg, 0.45 mmol, 60% yield over 2 steps) was prepared according to general procedure 2 for 3 hours.

Colorless gum.

Rf = 0.5 (8/2 Hexane/EtOAc).

The NMR spectra are obtained at 45 °C.

^{1}H NMR (400 MHz, $CDCl_3$) δ 4.39 (tq, *J* = 6.6, 2.1 Hz, 1H), 4.04 (s, 2H), 2.70 (t, *J* = 12.3 Hz, 2H), 1.82 (d, *J* = 2.1 Hz, 3H), 1.71 – 1.51 (m, 5H), 1.45 (s, 9H), 1.17 – 1.03 (m, 2H), 0.16 (s, 9H).

^{13}C NMR (101 MHz, $CDCl_3$) δ 155.1, 81.0, 80.6, 79.3, 60.9, 45.7, 44.2 (2C), 32.7, 32.5, 32.3, 28.7, 3.6, 0.4.

HRMS (ESI/QTOF) m/z: $[M + Na]^+$ Calcd for $C_{18}H_{33}NNaO_3Si^+$ 362.2122; Found 362.2108.

IR (ν_{max}, cm^{-1}) 2959 (w), 2922 (w), 2857 (w), 1694 (s), 1420 (m), 1361 (w), 1279 (m), 1249 (m), 1172 (m), 1151 (m), 1087 (m), 1072 (m), 864 (m), 842 (s), 761 (w).

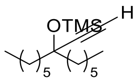

S8

((7-ethynyltridecan-7-yl)oxy)trimethylsilane **S8**:

Compound **S8** (525 mg, 1.78 mmol, 90% yield over 3 steps) was prepared according to general procedure 2 for 1.5 hours.

Colorless liquid.

Rf = 0.66 (98/2 Hexane/EtOAc).

^{1}H NMR (400 MHz, $CDCl_3$) δ 2.42 (s, 1H), 1.62-1.58 (m, 4H), 1.47 – 1.38 (m, 4H), 1.35-1.24 (m, 12H), 0.91 – 0.87 (m, 6H), 0.17 (s, 9H).

^{13}C NMR (151 MHz, $CDCl_3$) δ 87.8, 73.2, 72.6, 43.0, 32.0, 29.6, 24.3, 22.8, 14.2, 2.2.

HRMS (Sicrit plasma/LTQ-Orbitrap) m/z: $[M + H]^+$ Calcd for $C_{18}H_{37}OSi^+$ 297.2608; Found 297.2600.

IR (ν_{max}, cm^{-1}) 3693 (w), 3600 (w), 3026 (w), 2966 (w), 2947 (w), 2346 (w), 1738 (s), 1451 (m), 1374 (m), 1229 (m), 1211 (m), 910 (m), 743 (m).

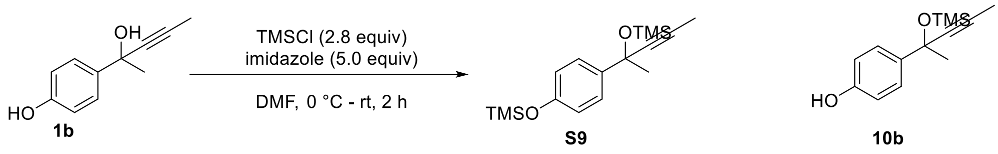

To a solution of **1b** (353 mg, 2 mmol, 1.0 equiv) in dry DMF (10 mL) was added imidazole (650.7 mg, 10 mmol, 2.0 equiv). The reaction mixture was cooled to 0 °C and TMSCl (0.71 mL, 5.6 mmol, 2.8 equiv) was added dropwise. The reaction mixture was then warmed to room temperature and stirred for 2 hours. The reaction mixture was then quenched with water. The reaction mixture was extracted three times with EtOAc. The combined organic layers were washed with brine, dried over Na_2SO_4, filtered, and evaporated *in vacuo*.

The crude NMR showed only formation of **S9** which was converted to **10b** upon purification. The crude product was then purified by flash column chromatography on silica gel (pentane/EtOAc 98/2 to pentane/EtOAc 7.5/2.5) to afford **S9** (315 mg, 0.98 mmol, 49% yield over 2 steps) as a pale-yellow liquid and **10b** (216 mg, 0.86 mmol, 43% yield over 2 steps) as a bright yellow gum.

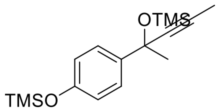

S9

trimethyl(4-(2-((trimethylsilyl)oxy)pent-3-yn-2-yl)phenoxy)silane **S9**:

Rf = 0.68 (9/1 Hexane/EtOAc).

^{1}H NMR (400 MHz, $CDCl_3$) δ 7.48 – 7.41 (m, 2H), 6.80 – 6.74 (m, 2H), 1.90 (s, 3H), 1.67 (s, 3H), 0.26 (s, 9H), 0.11 (s, 9H).

^{13}C NMR (151 MHz, $CDCl_3$) δ 154.3, 140.6, 126.5, 119.4, 83.5, 81.6, 71.1, 36.0, 3.8, 1.8, 0.4.

IR (ν_{max}, cm^{-1}) 2992 (w), 2964 (m), 1738 (m), 1728 (m), 1506 (m), 1367 (m), 1247 (s), 1092 (m), 993 (m), 913 (s), 842 (s), 747 (m), 680 (m).

Product not stable upon HRMS ionization.

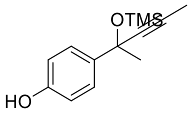

10b

4-(2-((trimethylsilyl)oxy)pent-3-yn-2-yl)phenol **10b**:

Rf = 0.31 (9/2 Hexane/EtOAc).

^{1}H NMR (400 MHz, $CDCl_3$) δ 7.48 – 7.44 (m, 2H), 6.79 – 6.75 (m, 2H), 4.74 (s, 1H, -OH), 1.90 (s, 3H), 1.67 (s, 3H), 0.12 (s, 9H).

^{13}C NMR (151 MHz, $CDCl_3$) δ 154.6, 140.2, 126.7, 114.7, 83.4, 81.8, 71.1, 36.1, 3.8, 1.8.

HRMS (Sicrit plasma/LTQ-Orbitrap) m/z: $[M - OH]^+$ Calcd for $C_{14}H_{19}OSi^+$ 231.1200; Found 231.1199.

IR (ν_{max}, cm^{-1}) 3339 (w), 2985 (w), 2957 (w), 2922 (w), 1612 (w), 1596 (w), 1510 (m), 1441 (w), 1365 (w), 1248 (m), 1226 (m), 1170 (m), 1092 (m), 1069 (w), 992 (m), 965 (w), 839 (s), 755 (w).

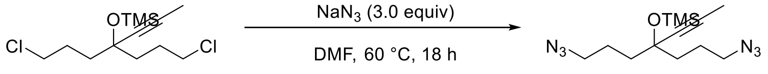

10g **10f**

To a solution of the crude **10g** (207 mg, 0.7 mmol, 1.0 equiv) in dry DMF (1.4 mL) was added sodium azide (137 mg, 2.1 mmol, 3.0 equiv). The reaction mixture was heated to 60 °C and stirred for 18 hours. The reaction mixture was then poured into a saturated solution of Na_2CO_3 with ice. The reaction mixture was extracted three times with EtOAc. The combined organic layers were washed with brine, dried over Na_2SO_4, filtered,

and evaporated *in vacuo*. The crude product was then purified by flash column chromatography on silica gel (pentane/EtOAc 8.5/1.5) to afford the desired product **10f** (136 mg, 0.45 mmol, 64% yield) as a colorless oil.

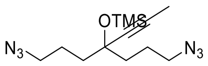

10f

((7-azido-4-(3-azidopropyl)hept-2-yn-4-yl)oxy)trimethylsilane **10f**:

Rf = 0.5 (9/1 Hexane/EtOAc).

^{1}H NMR (400 MHz, $CDCl_3$) δ 3.35 – 3.24 (m, 4H), 1.84 (s, 3H), 1.79-1.70 (m, 4H), 1.67 – 1.62 (m, 4H), 0.17 (s, 9H).

^{13}C NMR (151 MHz, $CDCl_3$) δ 82.3, 81.7, 71.9, 51.8, 40.4, 24.3, 3.6, 2.0.

HRMS (ESI/QTOF) m/z: $[M + Ag]^+$ Calcd for $C_{13}H_{24}AgN_6OSi^+$ 415.0826; Found 415.0826.

IR (ν_{max}, cm^{-1}) 2956 (w), 2922 (w), 2851 (w), 2092 (s), 1452 (w), 1344 (w), 1255 (m), 1248 (s), 1110 (w), 1056 (m), 1043 (m), 839 (s), 754 (m), 694 (w).

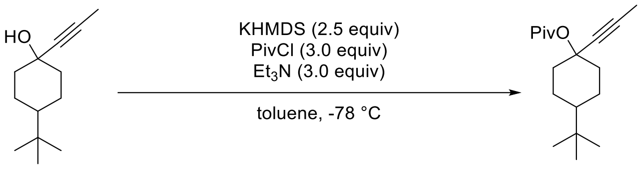

1k **S10**

The synthesis of **S10** was adapted from a literature procedure[3] using 4-(*tert*-butyl)-1-(prop-1-yn-1-yl)cyclohexan-1-ol (**1k**). To a solution of **1k** (58 mg, 0.3 mmol, 1.0 equiv) in dry toluene (3 mL) at -78 °C was added dropwise KHMDS (0.7 mL, 0.7 mmol, 2.5 equiv, [1 M] in THF). The mixture was stirred at -78 °C for 30 minutes before pivaloyl chloride (0.11 mL, 0.9 mmol, 3.0 equiv) and triethylamine (0.12 mL, 0.9 mmol, 3.0 equiv) were added. The reaction mixture was stirred at -78 °C for 1 hour. The reaction mixture was quenched with water and sodium hydroxide [1 M] and extracted three times with EtOAc. The combined organic layers were washed with sodium hydroxide [1 M] and with brine, dried over Na_2SO_4, filtered, and evaporated *in vacuo*. The crude product was purified by flash column chromatography on silica gel (hexane/Et_2O 9/1) to afford the desired product **S10** (78.6 mg, 0.2 mmol, 66% yield over 2 steps) as a yellow oil.

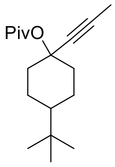

S10

4-(*tert*-butyl)-1-(prop-1-yn-1-yl)cyclohexyl pivalate **S10**:

Rf = 0.71 (3/7 Et_2O/pentane).

^{1}H NMR (400 MHz, $CDCl_3$) *δ* 2.43-2.35 (m, 2H), 1.87 (s, 3H), 1.73-1.65 (m, 2H), 1.53 – 1.37 (m, 4H), 1.16 (s, 9H), 1.05-0.96 (m, 1H), 0.86 (s, 9H).

^{13}C NMR (101 MHz, $CDCl_3$) *δ* 176.8, 82.8, 78.6, 76.6, 47.5, 39.2, 37.7, 32.4, 27.7, 27.3, 24.4, 3.9.

HRMS (ESI/QTOF) *m/z*: $[M + Na]^+$ Calcd for $C_{18}H_{30}NaO_2{}^+$ 301.2138; Found 301.2142.

IR (ν_{max}, cm^{-1}) 2953 (s), 2863 (m), 1736 (s), 1478 (w), 1366 (w), 1284 (m), 1159 (s), 1143 (s), 1026 (m).

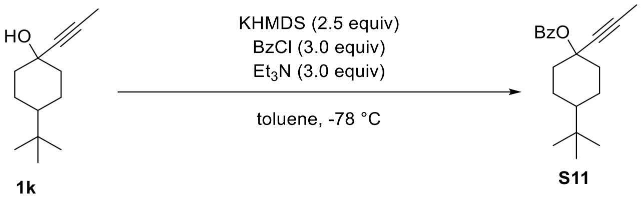

The synthesis of **S11** was adapted from a literature procedure[3] using 4-(*tert*-butyl)-1-(prop-1-yn-1-yl)cyclohexan-1-ol (**1k**). To a solution of **1k** (58 mg, 0.3 mmol, 1.0 equiv) in dry toluene (3 mL) at -78 °C was added dropwise KHMDS (0.7 mL, 0.7 mmol, 2.5 equiv, [1 M] in THF). The mixture was stirred at -78 °C for 30 minutes before benzoyl chloride (0.10 mL, 0.9 mmol, 3.0 equiv) and triethylamine (0.12 mL, 0.9 mmol, 3.0 equiv) were added. The reaction mixture was stirred at -78 °C for 1 hour. The reaction mixture was quenched with water and sodium hydroxide [1 M] and extracted three times with EtOAc. The combined organic layers were washed with sodium hydroxide [1 M] and with brine, dried over Na_2SO_4, filtered, and evaporated *in vacuo*. The crude product was purified by flash column chromatography on silica gel (hexane/Et_2O 9/1) to afford the desired product **S11** (78.6 mg, 0.21 mmol, 72% yield over 2 steps) as a colorless gum.

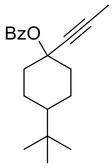

S11

4-(*tert*-butyl)-1-(prop-1-yn-1-yl)cyclohexyl benzoate **S11**:

Rf = 0.57 (3/7 Et_2O/pentane).

^{1}H NMR (400 MHz, $CDCl_3$) δ 8.04-8.00 (m, 2H), 7.55-7.50 (m, 1H), 7.41 (t, J = 7.6 Hz, 2H), 2.60-2.54 (m, 2H), 1.90 (s, 3H), 1.81-1.73 (m, 2H), 1.71-1.62 (m, 2H), 1.47 (td, J = 12.7, 3.0 Hz, 2H), 1.06 (tt, J = 12.0, 3.4 Hz, 1H), 0.89 (s, 9H).

^{13}C NMR (101 MHz, $CDCl_3$) δ 165.0, 132.7, 131.5, 129.8, 128.3, 83.6, 78.5, 77.9, 47.4, 38.0, 32.5, 27.7, 24.5, 3.9.

HRMS (ESI/QTOF) *m/z*: $[M + Na]^+$ Calcd for $C_{20}H_{26}NaO_2^+$ 321.1825; Found 321.1827.

IR (ν_{max}, cm^{-1}) 2949 (m), 2862 (w), 1725 (s), 1281 (s), 1261 (s), 1109 (m), 1024 (m), 711 (m).

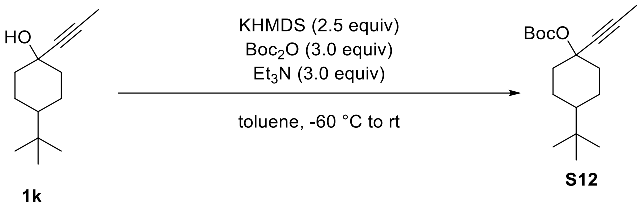

To a solution of **1k** (38.5 mg, 0.2 mmol, 1.0 equiv) in dry THF (2 mL) at -60 °C was added dropwise KHMDS (0.5 mL, 0.5 mmol, 2.5 equiv, [1 M] in THF). The mixture was stirred at -60 °C for 30 minutes before Boc_2O (109.12 mg, 0.5 mmol, 2.5 equiv) was added in one portion. The reaction mixture was then allowed to warm to 0 °C and stirred for an additional 30 minutes, followed by warming to room temperature and stirring for 2,5 hours. The reaction mixture was quenched with water and extracted three times with EtOAc. The combined organic layers were washed with sodium hydroxide [1 M] and with brine, dried over Na_2SO_4, filtered, and evaporated *in vacuo*. The crude product was purified by flash column chromatography on silica gel

(hexane/EtOAc 96/4) to afford the desired product **S12** (44.35 mg, 0.15 mmol, 75% yield over 2 steps) as a colorless oil.

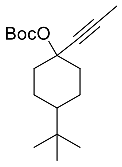

S12

(4-(*tert*-butyl)-1-(prop-1-yn-1-yl)cyclohexyl) carbonate **S12**:

Rf = 0.68 (pentane 100%).

^{1}H NMR (400 MHz, $CDCl_3$) δ 2.43-2.36 (m, 2H), 1.89 (s, 3H), 1.76-1.68 (m, 2H), 1.62 – 1.53 (m, 2H), 1.49 (s, 9H), 1.46 – 1.37 (m, 2H), 1.00 (tt, J = 11.8, 3.4 Hz, 1H), 0.86 (s, 9H).

^{13}C NMR (101 MHz, $CDCl_3$) δ 151.6, 83.5, 81.9, 78.8, 47.3, 37.8, 32.4, 31.1, 28.0, 27.7, 24.5, 3.9.

HRMS (APCI/QTOF) *m/z*: $[M + Na]^+$ Calcd for $C_{18}H_{30}NaO_3^+$ 317.2087; Found 317.2081.

IR (ν_{max}, cm^{-1}) 2950 (s), 2867 (m), 2361 (m), 2338 (m), 1747 (s), 1368 (m), 1283 (s), 1252 (s), 1165 (s), 1024 (m), 864 (m).

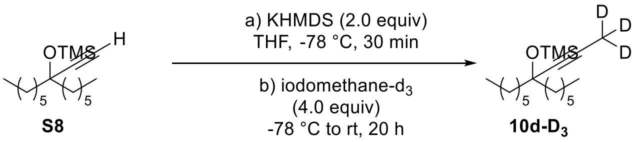

To a solution of **S8** (2524 mg, 1.77 mmol, 1.0 equiv) in dry THF (17.7 mL) at -78 °C was added dropwise KHMDS (5.3 mL, 5.31 mmol, 3.0 equiv, [1 M] in THF). The mixture was stirred at -78 °C for 30 minutes. Iodomethane-d_3 (0.12 mL, 17.7 mmol, 10.0 equiv) was added dropwise. The reaction mixture was then allowed to warm to room temperature and stirred for an additional 15 hours. The reaction mixture was quenched with NH_4Cl and extracted three times with Et_2O. The combined organic layers were washed with brine, dried over Na_2SO_4, filtered, and evaporated *in vacuo*. The crude product was purified by flash column chromatography on silica gel (hexane/Et_2O 97/3) to afford the desired product **10d-D3** (347 mg, 1.12 mmol, 63% yield) as a pale-yellow liquid.

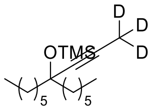

10d-D$_3$

trimethyl((7-(prop-1-yn-1-yl-d$_3$)tridecan-7-yl)oxy)silane **10d-D$_3$**:

Rf = 0.51 (98/2 Hexane/EtOAc).

^{1}H NMR (500 MHz, $CDCl_3$) δ 1.58-1.51 (m, 4H), 1.43 – 1.35 (m, 4H), 1.34 – 1.25 (m, 12H), 0.89 (t, J = 6.7 Hz, 6H), 0.15 (s, 9H).

^{13}C NMR (126 MHz, $CDCl_3$) δ 83.2, 80.7 (m), 72.9, 43.3, 32.1, 29.7, 24.5, 22.8, 14.3, 3.0 (sept, J = 20.0 Hz), 2.1.

HRMS (Sicrit plasma/LTQ-Orbitrap) m/z: $[M + H]^+$ Calcd for $C_{19}H_{36}[^2H]_3OSi^+$ 314.2953; Found 314.2953.

IR (ν_{max}, cm^{-1}) 2952 (s), 2932 (s), 2861 (m), 1735 (m), 1461 (m), 1373 (m), 1249 (m), 1206 (m), 1072 (m), 1056 (m), 913 (m), 842 (s), 744 (m), 680 (m), 654 (m).

4) Optimization of the reaction conditions

Procedure A:

To a solution of **10a** (1.0 equiv) in dry Et_2O [0.1 M] was added base (1.5 to 3.0 equiv) at -78 °C. The reaction mixture was then slowly warmed to the indicated temperature and stirred for 35 minutes. A solution of additive (1.5 to 3.0 equiv) in dry Et_2O [0.2 M] was then added dropwise at -78 °C and the reaction mixture was stirred for 18 hours at room temperature. The reaction mixture was poured into a cold saturated solution of NH_4Cl. The aqueous phase was extracted three times with Et_2O. The combined organic layers were washed with brine, dried over Na_2SO_4, filtered, and concentrated *in vacuo.* The crude product was then purified by preparative TLC (pentane/Et_2O 98/2) to afford the desired product.

Procedure B:

Diisopropylamine was distilled over CaH_2. In an oven-dried flask containing dry THF [2.1 M] at -78 °C and diisopropylamine (1.2 equiv) was added dropwise *n*BuLi (1.0 equiv). The reaction mixture was stirred at -78 °C for 30 min. In a second vial was added the protected propargylic alcohol (1.0 equiv) and Et_2O [0.15 M]. The reaction mixture was cooled to -78 °C and the freshly prepared LDA (3.0 equiv, [1 M]) was added dropwise. The reaction mixture was then stirred for 45 minutes. The reaction mixture was poured into a saturated solution of NH_4Cl and ice. The mixture was extracted three times with Et_2O. The combined organic layers were washed with brine, dried over Na_2SO_4, filtered, and concentrated *in vacuo.* The reaction mixture was then purified by flash column chromatography on silica gel (pentane 100%) to afford the pure desired product.

Table S1. Initial Screening Using Procedure A

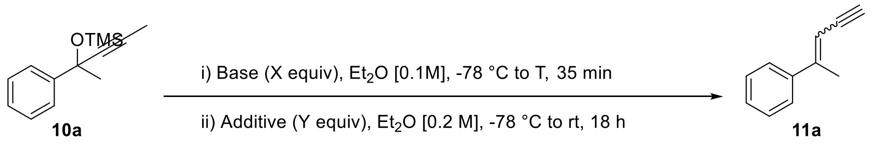

Entry	Additive	X equiv	Y equiv	Base	T	**10a/11a**
1	$ZnBr_2$	1.5	1.5	LDA	-20 °C	5/1
2	ZnI_2	1.5	1.5	LDA	-20 °C	12/1
3	$ZnCl_2$	1.5	1.5	LDA	-20 °C	12/1
4	$InBr_3$	1.5	1.5	LDA	-20 °C	4.5/1
5	$ZnBr_2$	3.0	1.5	LDA	-20 °C	18/1
6	$InBr_3$	3.0	1.5	LDA	-20 °C	7.6/1
7	$InBr_3$	1.5	1.5	*n*BuLi	-20 °C	no conv
8	$InBr_3$	1.5	1.5	*n*BuLi	-10 °C	no conv
9	$InBr_3$	1.5	1.5	LiHMDS	-20 °C	no conv
10	$InBr_3$	1.5	1.5	KHMDS	-20 °C	no conv
11	$InBr_3$	1.5	1.5	LDA	rt	2/1
12	HBr	1.5	1.5	LDA	rt	2.2/1
13	$InBr_3$	2.0	2.0	LDA	rt	1/1
14	HBr	2.0	2.0	LDA	rt	1/1
15	$InBr_3$	2.5	2.5	LDA	rt	1/15
16	HBr	2.5	2.5	LDA	rt	1/18
17	none	-	2.5	LDA	rt	1/18
18	none	-	2.5	LDA	rt	1/18[(a)]
19	none	-	3.0	LDA	rt	1/19
20	none	-	3.0	LDA	rt	74%[(b)]
21	none	-	3.0	*n*BuLi	rt	no conv
22	none	-	3.0	LiTMP	rt	71%[(b)]
23	none	-	3.0	NaH	rt	no conv[(a)]

Reactions were performed on 15 mg scale. (a) THF was used instead of Et_2O. (b) [0.15 M] instead of [0.1 M].

Table S2. Influence of the Protecting Group Using Procedure B

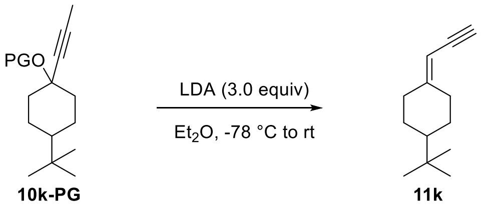

Entry	PG	Yield
1	TMS	95%
2	Piv	84%
3	Bz	51%
4	Boc	no conv.

Reactions were performed on 0.2 mmol scale.

5) Synthesis and characterization data of 1,3-enyne derivatives

General procedure 1 for the 1,3-enyne synthesis:

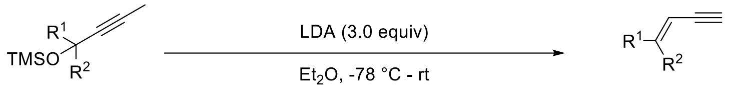

Diisopropylamine was distilled over CaH_2. In an oven-dried flask containing dry THF [2.1 M] at -78 °C and diisopropylamine (1.2 equiv) was added dropwise *n*BuLi (1.0 equiv). The reaction mixture was stirred at -78 °C for 30 min. In a second vial was added the protected propargylic alcohol (1.0 equiv) and Et_2O [0.15 M]. The reaction mixture was cooled to -78 °C and the freshly prepared LDA (3.0 equiv, [1 M]) was added dropwise. The reaction mixture was then stirred at room temperature for the indicated time. The reaction mixture was poured into a saturated solution of NH_4Cl and ice. The mixture was extracted three times with Et_2O. The combined organic layers were washed with brine, dried over Na_2SO_4, filtered, and concentrated *in vacuo.* The reaction mixture was then purified by flash column chromatography on silica gel (pentane 100% or pentane/Et_2O 98/2 or pentane/Et_2O 94/6 or pentane/EtOAc 7/3 depending on the product polarity) to afford the pure desired product.

General procedure 2 for the one pot alkylation/acylation:

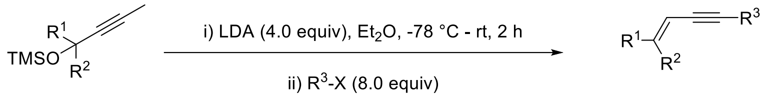

Diisopropylamine was distilled over CaH_2. In an oven-dried flask containing dry THF [2.1 M] at -78 °C and diisopropylamine (1.0 equiv) was added dropwise *n*BuLi (1.0 equiv). The reaction mixture was stirred at -78 °C for 30 min. In a second vial was added the protected propargylic alcohol (1.0 equiv) and Et_2O [0.15 M]. The reaction mixture was cooled to -78 °C and the freshly prepared LDA (4.0 equiv, [1 M]) was added dropwise. The reaction mixture was then stirred at room temperature for 2 hours. Alkylating agent (8.0 equiv) was then added dropwise and the reaction mixture was stirred for the indicated time. The reaction mixture was poured into water and ice. The mixture was extracted three times with Et_2O. The combined organic layers were washed with brine, dried over Na_2SO_4, filtered, and concentrated *in vacuo.* The reaction mixture was then purified by flash column chromatography on silica gel (pentane 100% or pentane/Et_2O 96/4 or pentane/Et_2O 1.5/8.5 depending on the product polarity) to afford the pure desired product.

General procedure 3:

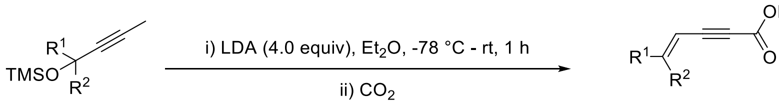

Diisopropylamine was distilled over CaH_2. In an oven-dried flask containing dry THF [2.1 M] at -78 °C and diisopropylamine (1.0 equiv) was added dropwise *n*BuLi (1.0 equiv). The reaction mixture was stirred at -78 °C for 30 min. In a second vial was added the protected propargylic alcohol (1.0 equiv) and Et_2O [0.15 M]. The reaction mixture was cooled to -78 °C and the freshly prepared LDA (4.0 equiv, [1 M]) was added dropwise. The reaction mixture was then stirred at room temperature for 1 hour. A balloon of CO_2 was then bubbled into the flask for 3 minutes and the reaction mixture was stirred under CO_2 atmosphere for an additional 15 minutes. The reaction mixture was poured into water and ice. 1 M HCl was added until PH <3. The mixture was extracted three times with EtOAc. The combined organic layers were washed with brine, dried over Na_2SO_4, filtered, and concentrated *in vacuo.* The reaction mixture was then purified by flash column chromatography on silica gel (pentane/Et_2O 8/2 followed by MeOH/EtOAc/AcOH 1/9/1%) to afford the pure desired product.

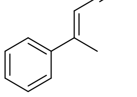

11a

(*E*)-pent-2-en-4-yn-2-ylbenzene **11a**:

Obtained as a 16:1 mixture of E/Z isomers.

Compound **11a** (21 mg, 0.15 mmol, 74% yield) was prepared according to general procedure 1 for 45 minutes. All characterization data were in full agreement with those reported in the literature.[4]

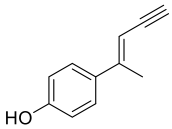

11b

(*E*)-4-(pent-2-en-4-yn-2-yl)phenol **11b**:

Compound **11b** (30 mg, 0.19 mmol, 95% yield) was prepared according to general procedure 1 for 1 hour with 4.5 equivalents of LDA.

Beige solid obtained as a 9.5:1 mixture of E/Z isomers.

Rf = 0.22 (8/2 Hexane/EtOAc).

m.p.: 67.7 – 68.9 °C.

^{1}H NMR (400 MHz, $CDCl_3$) δ 7.37 – 7.32 (m, 2H), 6.83 – 6.79 (m, 2H), 5.79-5.77 (m, 1H), 4.76 (s, 1H), 3.24 (d, *J* = 2.3 Hz, 1H), 2.31 (s, 3H).

^{13}C NMR (151 MHz, $CDCl_3$) δ 155.9, 149.8, 133.5, 127.1, 115.4, 103.9, 82.6, 82.4, 18.7.

HRMS (Sicrit plasma/LTQ-Orbitrap) m/z: $[M + H]^+$ Calcd for $C_{11}H_{11}O^+$ 159.0804; Found 159.0804.

IR (ν_{max}, cm^{-1}) 3426 (w), 3385 (m), 3302 (w), 3269 (s), 3260 (s), 1608 (w), 1597 (m), 1519 (m), 1441 (m), 1363 (w), 1252 (s), 1181 (m), 824 (s), 821 (s).

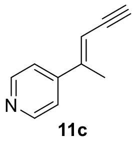

11c

(*E*)-4-(pent-2-en-4-yn-2-yl)pyridine **11c**:

Compound **11c** (12.6 mg, 0.088 mmol, 44% yield) was prepared according to general procedure 1 for 45 minutes.

Colorless liquid obtained as a 11:1 mixture of E/Z isomers.

Rf = 0.34 (4/6 Hexane/Et_2O).

Major:

^{1}H NMR (400 MHz, $CDCl_3$) δ 8.59 – 8.58 (m, 2H), 7.31 – 7.29 (m, 2H), 6.03 (dq, *J* = 2.5, 1.3 Hz, 1H), 3.37 (d, *J* = 2.3 Hz, 1H), 2.33 – 2.32 (m, 3H).

^{13}C NMR (151 MHz, $CDCl_3$) δ 150.3, 147.7, 147.7, 120.0, 109.0, 85.1, 81.3, 18.0.

HRMS (ESI/QTOF) m/z: $[M + H]^+$ Calcd for $C_{10}H_{10}N^+$ 144.0808; Found 144.0806.

IR ($ν_{max}$, cm^{-1}) 3296 (m), 3291 (m), 3037 (w), 2919 (w), 2856 (w), 1593 (s), 1539 (w), 1493 (w), 1411 (m), 1381 (w), 1224 (w), 994 (w), 812 (s), 777 (w).

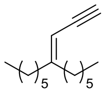

11d

7-(prop-2-yn-1-ylidene)tridecane **11d**:

Compound **11d** (41.7 mg, 0.19 mmol, 95% yield) was prepared according to general procedure 1 for 2.5 hours.

Compound **11d** (640 mg, 2.90 mmol, 90% yield) was prepared according to general procedure 1 for 2 hours.

Colorless oil.

Rf = 0.82 (98/2 Hexane/Et_2O).

^{1}H NMR (400 MHz, $CDCl_3$) δ 5.24 (q, J = 1.6 Hz, 1H), 2.99 (d, J = 2.3 Hz, 1H), 2.33 – 2.29 (m, 2H), 2.09 – 2.05 (m, 2H), 1.49 – 1.37 (m, 4H), 1.34-1.24 (m, 12H), 0.89 (t, J = 6.7 Hz, 3H), 0.89 (t, J = 6.7 Hz, 3H).

^{13}C NMR (101 MHz, $CDCl_3$) δ 159.3, 103.4, 82.0, 79.5, 36.1, 33.2, 31.84, 31.82, 29.33, 29.17, 27.9, 27.7, 22.7 (2C), 14.25, 14.23.

HRMS (Sicrit plasma/LTQ-Orbitrap) m/z: [M + H]$^+$ Calcd for $C_{16}H_{29}^+$ 221.2264; Found 221.2263.

IR ($ν_{max}$, cm^{-1}) 3312 (w), 2955 (m), 2926 (s), 2856 (m), 1592 (m), 1493 (w), 1454 (s), 1442 (m), 1378 (m), 735 (m), 702 (s).

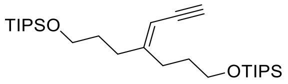

11e

3,3,13,13-tetraisopropyl-2,14-dimethyl-8-(prop-2-yn-1-ylidene)-4,12-dioxa-3,13-disilapentadecane **11e**:

Compound **11e** (91 mg, 0.19 mmol, 95% yield) was prepared according to general procedure 1 for 1.5 hours.

Yellow liquid.

Rf = 0.32 (9.5/0.5 Hexane/EtOAc).

^{1}H NMR (400 MHz, $CDCl_3$) δ 5.29-5.27 (m, 1H), 3.73 – 3.65 (m, 4H), 2.98 (d, J = 2.2 Hz, 1H), 2.42 – 2.38 (m, 2H), 2.23 – 2.17 (m, 2H), 1.74-1.62 (m, 4H), 1.13 – 1.02 (m, 42H).

^{13}C NMR (101 MHz, $CDCl_3$) δ 158.4, 103.9, 81.7, 79.9, 63.5, 62.9, 32.6, 31.5, 31.2, 30.0, 18.2, 18.2, 12.2, 12.1.

HRMS (ESI/QTOF) m/z: [M + Na]$^+$ Calcd for $C_{28}H_{56}NaO_2Si_2^+$ 503.3711; Found 503.3716.

IR ($ν_{max}$, cm^{-1}) 3318 (w), 2956 (m), 2942 (s), 2930 (s), 2894 (m), 2865 (s), 1463 (m), 1380 (w), 1244 (w), 1107 (s), 1069 (m), 1013 (w), 996 (w), 878 (m), 725 (w), 681 (s).

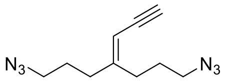

11f

7-azido-4-(3-azidopropyl)hept-3-en-1-yne **11f**:

Compound **11f** (14 mg, 0.13 mmol, 64% yield) was prepared according to general procedure 1 for 5 hours with 6 equivalents of LDA and Et_2O [0.08 M] because of solubility issue.

Colorless liquid.

Rf = 0.38 (9/1 Hexane/EtOAc).

^{1}H NMR (400 MHz, $CDCl_3$) δ 5.35 (q, *J* = 1.7 Hz, 1H), 3.32 (t, *J* = 6.7 Hz, 2H), 3.30 (t, *J* = 6.7 Hz, 2H), 3.07 (d, *J* = 2.3 Hz, 1H), 2.41 (dd, *J* = 8.8, 6.7 Hz, 2H), 2.19 (td, *J* = 7.5, 1.3 Hz, 2H), 1.40-1.69 (m, 4H).

^{13}C NMR (151 MHz, $CDCl_3$) δ 155.0, 106.0, 81.0, 80.9, 51.2, 50.9, 33.0, 30.3, 27.2, 27.0.

HRMS (Sicrit plasma/LTQ-Orbitrap) m/z: $[M + H]^+$ Calcd for $C_{10}H_{15}N_6^+$ 219.1353; Found 219.1352.

IR (ν_{max}, cm^{-1}) 3293 (w), 2938 (w), 2871 (w), 2098 (s), 1452 (w), 1355 (w), 1296 (w), 1255 (w).

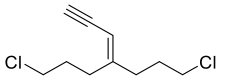

11g

7-chloro-4-(3-chloropropyl)hept-3-en-1-yne **11g**:

Compound **11g** (38.9 mg, 0.19 mmol, 95% yield) was prepared according to general procedure 1 for 1.75 hours with 2.3 equivalents of LDA to avoid side reactions.

colorless liquid.

Rf = 0.18 (98.5/1.5 Hexane/EtOAc).

^{1}H NMR (400 MHz, $CDCl_3$) δ 5.37-5.35 (m, 1H), 3.56 (t, *J* = 6.6 Hz, 2H), 3.53 (t, *J* = 6.6 Hz, 2H), 3.06 (d, *J* = 2.3 Hz, 1H), 2.49-2.45 (m, 2H), 2.29 – 2.25 (m, 2H), 1.99 – 1.87 (m, 4H).

^{13}C NMR (101 MHz, $CDCl_3$) δ 154.8, 106.1, 81.0, 80.9, 44.7, 44.3, 33.1, 31.0, 30.7, 30.5.

HRMS (Sicrit plasma/LTQ-Orbitrap) m/z: $[M + H]^+$ Calcd for $C_{10}H_{15}Cl_2^+$ 205.0545; Found 205.0541.

IR (ν_{max}, cm^{-1}) 3293 (s), 3000 (w), 2956 (s), 2923 (m), 2859 (w), 1622 (w), 1443 (m), 1310 (m), 1288 (m), 1277 (m), 1231 (w), 840 (w), 772 (w), 725 (m), 655 (s).

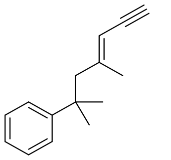

11h

(*E*)-(2,4-dimethylhept-4-en-6-yn-2-yl)benzene **11h**:

Compound **11h** (31.6 mg, 0.16 mmol, 80% yield) was prepared according to general procedure 1 for 3 hours.

Yellow solid obtained as a 2:1 mixture of E/Z isomers.

m.p.: 55.7 – 56.3 °C.

Rf = 0.56 (9.5/0.5 Hexane/EtOAc).

Major:

^{1}H NMR (400 MHz, $CDCl_3$) δ 7.42 – 7.38 (m, 2H), 7.33 – 7.28 (m, 2H), 7.21 – 7.16 (m, 1H), 5.30 (p, *J* = 1.7 Hz, 1H), 3.00 (d, *J* = 2.3 Hz, 2H), 2.72 (s, 3H), 1.41 (s, 6H), 1.26 (d, *J* = 1.7 Hz, 3H).

^{13}C NMR (151 MHz, $CDCl_3$) δ 152.8, 149.6, 128.2, 126.1, 125.8, 107.4, 82.5, 79.6, 49.3, 39.2, 29.3, 24.5.

Minor:

^{1}**H NMR** (400 MHz, $CDCl_3$) δ 7.37 – 7.28 (m, 4H), 7.21 – 7.16 (m, 1H), 5.10 (dq, *J* = 2.3, 1.2 Hz, 1H), 3.02 (d, *J* = 2.2 Hz, 1H), 2.42 (s, 3H), 1.50 (d, *J* = 1.2 Hz, 3H), 1.34 (s, 6H).

^{13}**C NMR** (151 MHz, $CDCl_3$) δ 152.3, 148.9, 128.2, 126.0, 125.9, 107.4, 81.9, 80.4, 53.6, 38.4, 29.2, 21.3.

HRMS (Sicrit plasma/LTQ-Orbitrap) m/z: $[M + H]^+$ Calcd for $C_{15}H_{19}^+$ 199.1481; Found 199.1482.

IR (ν_{max}, cm^{-1}) 3307 (m), 3292 (w), 2967 (s), 2925 (m), 2875 (w), 1496 (m), 1456 (w), 1445 (m), 1383 (w), 1031 (w), 765 (m), 700 (s).

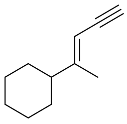

11i

(*E*)-pent-2-en-4-yn-2-ylcyclohexane **11i**:

Compound **11i** (21.3 mg, 0.14 mmol, 72% yield) was prepared according to general procedure 1 for 1 hour.

Colorless liquid obtained as a 2:1 mixture of E/Z isomers.

Rf = 0.57 (98/2 Hexane/EtOAc).

^{1}**H NMR** (400 MHz, $CDCl_3$) δ 5.27-5.26 (m, 1H, major), 5.19 (p, *J* = 1.7 Hz, 1H, minor), 3.02 (d, *J* = 2.2 Hz, 1H, major), 3.00 (d, *J* = 2.3 Hz, 1H, minor), 2.84 (tt, *J* = 11.3, 3.4 Hz, 1H, minor), 1.96 (tt, *J* = 11.5, 3.5 Hz, 1H, major), 1.90 (d, *J* = 1.1 Hz, 3H, major), 1.71 (d, *J* = 1.6 Hz, 3H, minor), 1.81-1.55 (m, 5H major + 5H minor), 1.41-1.09 (m, 5H major + 5H minor).

^{13}**C NMR** (126 MHz, $CDCl_3$) δ 159.6 (major), 159.5 (minor), 103.0 (minor), 102.1 (major), 82.3 (major), 81.5 (minor), 79.9 (major), 79.7 (minor), 46.7 (major), 42.9 (minor), 31.6 (major), 30.7 (minor), 26.6 (major), 26.5 (minor), 26.3 (major), 26.2 (minor), 19.2 (minor), 17.9 (major).

HRMS (Sicrit plasma/LTQ-Orbitrap) m/z: $[M + H]^+$ Calcd for $C_{11}H_{17}^+$ 149.1325; Found 149.1324.

IR (ν_{max}, cm^{-1}) 3311 (w), 3297 (w), 2922 (s), 2853 (m), 1719 (w), 1448 (m), 1372 (w), 1201 (w), 888 (w), 828 (w), 632 (m).

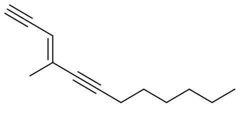

11j

(*E*)-4-methyldodeca-3-en-1,5-diyne **11j**:

Compound **11j** (20 mg, 0.11 mmol, 57% yield) was prepared according to general procedure 1 for 1 hour.

Yellow gum obtained as a 5.5:1 mixture of E/Z isomers.

Rf = 0.46 (99/1 Hexane/EtOAc).

^{1}**H NMR** (800 MHz, $CDCl_3$) δ 5.66 (s, 1H), 3.34 (d, *J* = 2.4 Hz, 1H), 2.32 (t, *J* = 7.2 Hz, 2H), 2.02 (d, *J* = 1.5 Hz, 3H), 1.53 (p, *J* = 7.3 Hz, 2H), 1.39 (p, *J* = 7.4 Hz, 2H), 1.34 – 1.26 (m, 4H), 0.89 (t, *J* = 7.1 Hz, 3H).

^{13}**C NMR** (201 MHz, $CDCl_3$) δ 134.3, 113.0, 94.9, 85.3, 82.1, 81.0, 31.5, 28.7, 22.7, 20.9, 19.6, 14.2.

HRMS (Sicrit plasma/LTQ-Orbitrap) m/z: $[M + H]^+$ Calcd for $C_{13}H_{19}^+$ 175.1481; Found 175.1481.

IR (ν_{max}, cm^{-1}) 3308 (m), 3301 (m), 2955 (s), 2929 (s), 2866 (m), 2858 (m), 2216 (w), 1467 (w), 1441 (w), 1376 (m), 1237 (w), 1185 (w), 884 (w), 847 (w), 646 (m)

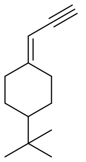

11k

1-(tert-butyl)-4-(prop-2-yn-1-ylidene)cyclohexane **11k**:

Compound **11k** (33 mg, 0.19 mmol, 95% yield) was prepared according to general procedure 1 for 45 minutes.

Colorless oil.

Rf = 0.74 (Hexane 100%).

^{1}H NMR (400 MHz, $CDCl_3$) δ 5.19 (q, *J* = 2.0 Hz, 1H), 3.04 (ddt, *J* = 13.5, 4.8, 2.3 Hz, 1H), 2.95 (d, *J* = 2.2 Hz, 1H), 2.32 (ddt, *J* = 13.3, 4.0, 2.4 Hz, 1H), 2.15 – 2.02 (m, 1H), 1.95-1.82 (m, 3H), 1.19 (dt, *J* = 11.7, 2.7 Hz, 1H), 1.15 – 1.01 (m, 2H), 0.86 (s, 9H).

^{13}C NMR (151 MHz, $CDCl_3$) δ 158.3, 100.3, 81.7, 78.9, 48.0, 36.0, 32.6, 31.6, 28.3, 27.8.

HRMS (Sicrit plasma/LTQ-Orbitrap) m/z: $[M + H]^+$ Calcd for $C_{13}H_{21}^+$ 177.1638; Found 177.1635.

IR (ν_{max}, cm^{-1}) 3311 (m), 2952 (s), 2926 (s), 2864 (m), 1456 (m), 1367 (m), 1116 (w).

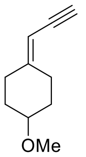

11l

1-methoxy-4-(prop-2-yn-1-ylidene)cyclohexane **11l**:

Compound **11l** (29 mg, 0.19 mmol, 97% yield) was prepared according to general procedure 1 for 1 hour.

Pale yellow liquid obtained as a >20:1 mixture of E/Z isomers.

Rf = 0.43 (9/1 Hexane/EtOAc).

^{1}H NMR (400 MHz, $CDCl_3$) δ 5.24 (dt, *J* = 2.5, 1.3 Hz, 1H), 3.42 – 3.36 (m, 1H), 3.36 (s, 3H), 2.96 (d, *J* = 2.2 Hz, 1H), 2.75 – 2.64 (m, 1H), 2.37 (ddd, *J* = 13.0, 7.5, 4.5 Hz, 1H), 2.28 (dddd, *J* = 13.9, 9.1, 4.7, 1.2 Hz, 1H), 2.10 (dddd, *J* = 13.7, 9.0, 4.6, 1.4 Hz, 1H), 1.92 – 1.80 (m, 2H), 1.65 – 1.55 (m, 2H).

^{13}C NMR (151 MHz, $CDCl_3$) δ 156.2, 101.5, 81.4, 79.3, 77.1, 56.0, 32.0, 31.9, 31.3, 27.6.

HRMS (Sicrit plasma/LTQ-Orbitrap) m/z: $[M + H]^+$ Calcd for $C_{10}H_{15}O^+$ 151.1117; Found 151.1117.

IR (ν_{max}, cm^{-1}) 3735 (w), 3625 (w), 3314 (w), 3240 (w), 2935 (s), 2856 (m), 2826 (m), 2360 (m), 1442 (m), 1247 (w), 1225 (w), 1192 (w), 1158 (w), 1100 (s), 1022 (w).

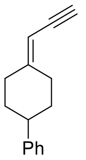

11m

(4-(prop-2-yn-1-ylidene)cyclohexyl)benzene **11m**:

Compound **11m** (29.9 mg, 0.15 mmol, 76% yield) was prepared according to general procedure 1 for 4.5 hours.

Orange liquid.

Rf = 0.86 (2/8 EtOAc /hexane).

^{1}H NMR (400 MHz, $CDCl_3$) δ 7.32 – 7.27 (m, 2H), 7.22 – 7.17 (m, 3H), 5.29 (d, *J* = 2.0 Hz, 1H), 3.18 – 3.08 (m, 1H), 2.99 (d, *J* = 2.2 Hz, 1H), 2.72 (tt, *J* = 12.0, 3.2 Hz, 1H), 2.45 – 2.38 (m, 1H), 2.28 (t, *J* = 13.4 Hz, 1H), 2.13 – 1.96 (m, 3H), 1.64 – 1.51 (m, 2H).

^{13}C NMR (101 MHz, $CDCl_3$) δ 156.7, 146.4, 128.6, 127.0, 126.3, 101.5, 81.4, 79.3, 44.3, 35.8, 35.5, 34.7, 31.5.

HRMS (Sicrit plasma/LTQ-Orbitrap) m/z: $[M + H]^+$ Calcd for $C_{15}H_{17}^+$ 197.1325; Found 197.1325.

IR (ν_{max}, cm^{-1}) 3306 (m), 3027 (m), 2926 (s), 2855 (m), 1494 (m), 1444 (m), 836 (m), 754 (m), 700 (s).

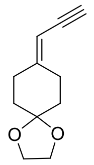

11n

8-(prop-2-yn-1-ylidene)-1,4-dioxaspiro[4.5]decane **11n**:

Compound **11n** (28.7 mg, 0.16 mmol, 81% yield) was prepared according to general procedure 1 for 30 minutes.

Colorless oil.

Rf = 0.37 (1/9 EtOAc/Hexane).

^{1}H NMR (400 MHz, $CDCl_3$) δ 5.27 (s, 1H), 3.97 (s, 4H), 2.98 (d, *J* = 2.2 Hz, 1H), 2.59 (t, *J* = 6.6 Hz, 2H), 2.34 (t, *J* = 6.5 Hz, 2H), 1.72 (q, *J* = 7.4 Hz, 4H).

^{13}C NMR (101 MHz, $CDCl_3$) δ 155.1, 108.5, 102.2, 81.3, 79.5, 64.6, 35.7, 35.0, 32.6, 28.3.

HRMS (Sicrit plasma/LTQ-Orbitrap) m/z: $[M + H]^+$ Calcd for $C_{11}H_{15}O_2^+$ 179.1067; Found 179.1066.

IR (ν_{max}, cm^{-1}) 2952 (m), 2878 (m), 1440 (w), 1271 (w), 1120 (s), 1087 (s), 1034 (m), 946 (m), 904 (m), 686 (m).

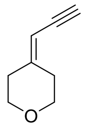

11o

4-(prop-2-yn-1-ylidene)tetrahydro-2*H*-pyran **11o**:

Compound **11o** (16 mg, 0.13 mmol, 66% yield) was prepared according to general procedure 1 for 1 hour.

Colorless oil.

Rf = 0.37 (9/1 Hexane/EtOAc).

^{1}H NMR (400 MHz, $CDCl_3$) δ 5.31 (dt, *J* = 2.4, 1.3 Hz, 1H), 3.71 (dt, *J* = 9.2, 5.5 Hz, 4H), 3.00 (d, *J* = 2.2 Hz, 1H), 2.58 – 2.55 (m, 2H), 2.31 (td, *J* = 5.5, 1.3 Hz, 2H).

^{13}C NMR (101 MHz, $CDCl_3$) δ 152.1, 102.5, 80.7, 80.2, 69.0, 68.6, 36.0, 32.5.

HRMS (Sicrit plasma/LTQ-Orbitrap) m/z: $[M + H]^+$ Calcd for $C_8H_{11}O^+$ 123.0804; Found 123.0804.

IR (ν_{max}, cm^{-1}) 3710 (m), 2967 (s), 2920 (s), 2852 (s), 2338 (m), 1736 (m), 1369 (m), 1247 (m), 1040 (m).

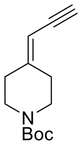

11p

tert-butyl 4-(prop-2-yn-1-ylidene)piperidine-1-carboxylate **11p**:

Compound **11p** (15 mg, 0.068 mmol, 34% yield) was prepared according to general procedure 1 for 1 hour.

Pale yellow liquid.

Rf = 0.43 (9/1 Hexane/EtOAc).

^{1}H NMR (400 MHz, $CDCl_3$) δ 5.33 (dt, *J* = 2.4, 1.3 Hz, 1H), 3.45 (dt, *J* = 8.1, 5.7 Hz, 4H), 3.01 (d, *J* = 2.2 Hz, 1H), 2.49 (t, *J* = 5.9 Hz, 2H), 2.25 (t, *J* = 5.8 Hz, 2H), 1.47 (s, 9H).

^{13}C NMR (151 MHz, $CDCl_3$) δ 154.8, 152.9, 103.2, 80.7, 80.3, 79.9, 45.9, 35.0, 31.2, 28.6.

HRMS (Sicrit plasma/LTQ-Orbitrap) m/z: $[M + H]^+$ Calcd for $C_{13}H_{20}NO_2^+$ 222.1489; Found 222.1488.

IR (ν_{max}, cm^{-1}) 3285 (w), 3250 (w), 2974 (w), 2935 (w), 2905 (w), 2872 (w), 1691 (s), 1420 (s), 1365 (m), 1306 (w), 1276 (m), 1244 (m), 1228 (m), 1163 (s), 1114 (m), 1021 (w), 984 (w), 848 (w), 770 (w).

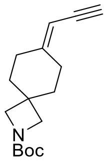

11q

tert-butyl 7-(prop-2-yn-1-ylidene)-2-azaspiro[3.5]nonane-2-carboxylate **11q**:

Compound **11q** (29 mg, 0.11 mmol, 56% yield) was prepared according to general procedure 1 for 1 hour.

Pale yellow solid.

m.p.: 69.2 – 71.4 °C.

Rf = 0.54 (8/2 Hexane/EtOAc).

^{1}H NMR (400 MHz, $CDCl_3$) δ 5.25 (dt, *J* = 2.3, 1.2 Hz, 1H), 3.65 (d, *J* = 8.2 Hz, 2H), 3.60 (d, *J* = 8.2 Hz, 2H), 2.97 (d, *J* = 2.2 Hz, 1H), 2.41 (t, *J* = 6.3 Hz, 2H), 2.17 – 2.14 (m, 2H), 1.78 – 1.72 (m, 4H), 1.45 (s, 9H).

^{13}C NMR (101 MHz, $CDCl_3$) δ 156.7, 155.0, 102.1, 81.1, 79.6, 79.5, 59.5 (by HSQC), 37.0, 36.3, 35.0, 31.9, 28.6, 27.7.

HRMS (ESI/QTOF) m/z: $[M + Na]^+$ Calcd for $C_{16}H_{23}NNaO_2^+$ 284.1621; Found 284.1625.

IR (ν_{max}, cm^{-1}) 3311 (w), 3252 (w), 2975 (w), 2934 (w), 2868 (w), 1700 (s), 1405 (s), 1366 (m), 1348 (w), 1171 (w), 1146 (m), 1080 (w).

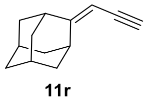

11r

(1*r*,3*r*,5*R*,7*S*)-2-(prop-2-yn-1-ylidene)adamantane **11r**:

Compound **11r** (30 mg, 0.18 mmol, 88% yield) was prepared according to general procedure 1 for 2 hours.

Colorless liquid.

Rf = 0.5 (98/2 Hexane/EtOAc).

^{1}H NMR (400 MHz, $CDCl_3$) δ 5.17 (d, *J* = 2.2 Hz, 1H), 3.18 (apparent t, *J* = 3.3 Hz, 1H), 2.91 (d, *J* = 2.2 Hz, 1H), 2.47 (apparent t, *J* = 3.3 Hz, 1H), 1.99 – 1.87 (m, 6H), 1.86 – 1.74 (m, 6H).

^{13}C NMR (201 MHz, $CDCl_3$) δ 166.5, 95.4, 81.6, 78.5, 39.8, 39.0, 37.1, 35.3, 28.3.

HRMS (APPI/LTQ-Orbitrap) m/z: $[M + H]^+$ Calcd for $C_{13}H_{17}^+$ 173.1325; Found 173.1321.

IR (ν_{max}, cm^{-1}) 3308 (m), 2902 (s), 2851 (m), 1738 (w), 1444 (w), 1355 (w), 1211 (w), 816 (w), 632 (w).

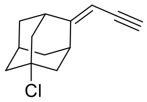

11s

(1*S*,3*R*,5*S*,7*S*,*Z*)-1-chloro-4-(prop-2-yn-1-ylidene)adamantane **11s**:

Compound **11s** (38 mg, 0.18 mmol, 92% yield) was prepared according to general procedure 1 for 2 hours.

Colorless liquid.

Rf = 0.32 (98/2 Hexane/EtOAc).

^{1}H NMR (400 MHz, $CDCl_3$) δ 5.22 (d, *J* = 2.2 Hz, 1H), 3.34 (apparent t, *J* = 3.4 Hz, 1H), 2.95 (d, *J* = 2.2 Hz, 1H), 2.67-2.62 (m, 1H), 2.29 – 2.12 (m, 7H), 1.89-1.80 (m, 2H), 1.79 – 1.68 (m, 2H).

^{13}C NMR (126 MHz, $CDCl_3$) δ 161.3, 97.9, 80.7, 79.6, 67.1, 48.7, 48.0, 47.2, 41.8, 37.9, 37.5, 37.1, 31.5.

HRMS (Sicrit plasma/LTQ-Orbitrap) m/z: $[M + H]^+$ Calcd for $C_{13}H_{16}Cl^+$ 207.0935; Found 207.0935.

IR (ν_{max}, cm^{-1}) 3291 (m), 2929 (s), 2918 (s), 2855 (m), 2098 (w), 1626 (w), 1448 (m), 1340 (w), 1285 (m), 1266 (w), 1103 (w), 1032 (s), 940 (w), 917 (w), 830 (s), 798 (w), 735 (w), 646 (s), 620 (s).

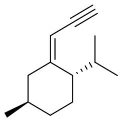

11t

(1*S*,4*R*)-1-isopropyl-4-methyl-2-(prop-2-yn-1-ylidene)cyclohexane **11t**:

Compound **11t** (31 mg, 0.18 mmol, 89% yield) was prepared according to general procedure 1 for 1 hour.

Colorless liquid obtained as a 1:1 mixture of E/Z isomers.

Rf = 0.48 (98/2 Hexane/EtOAc).

^{1}H NMR (500 MHz, $CDCl_3$) δ 5.23 (brs, 2H), 2.99 (d, *J* = 2.3 Hz, 1H), 2.93 (d, *J* = 2.3 Hz, 1H), 2.75 (dd, *J* = 13.0, 4.3 Hz, 1H), 2.56 (ddd, *J* = 10.7, 4.8, 2.4 Hz, 1H), 2.43 (ddd, *J* = 13.2, 5.6, 1.9 Hz, 1H), 2.12-2.03 (m, 1H), 1.96-1.86 (m, 2H), 1.89 (dd, *J* = 13.1, 8.1 Hz, 1H), 1.84 – 1.66 (m, 7H), 1.63 (dt, *J* = 13.9, 4.3 Hz, 1H), 1.28 (q, *J* = 8.9 Hz, 1H), 1.23 (dt, *J* = 13.4, 3.0 Hz, 1H), 1.13 (q, *J* = 8.8 Hz, 1H), 0.97 (d, *J* = 6.2 Hz, 3H), 0.96 (d, *J* = 6.2 Hz, 3H), 0.89 (d, *J* = 6.7 Hz, 3H), 0.89 (d, *J* = 6.7 Hz, 3H), 0.88 (d, *J* = 6.7 Hz, 3H), 0.84 (d, *J* = 6.6 Hz, 3H).

^{13}C NMR (126 MHz, $CDCl_3$) δ 159.4, 158.5, 103.6, 100.7, 82.1, 79.5, 78.8, 50.6, 46.9, 39.1, 38.7, 33.4, 32.4, 30.1, 27.2, 27.0, 26.9, 26.5, 23.3, 22.1, 21.0, 20.9, 19.4, 18.2.

HRMS (Sicrit plasma/LTQ-Orbitrap) m/z: $[M + H]^+$ Calcd for $C_{13}H_{21}^+$ 177.1638; Found 177.1638.

IR (νmax, cm-1) 3315 (m), 2955 (s), 2928 (s), 2870 (m), 2097 (w), 1734 (w), 1622 (w), 1456 (m), 1383 (m), 1366 (m), 1065 (w), 843 (m), 646 (m), 635 (m).

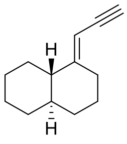

11u

(4a*R*,8a*S*,*E*)-1-(prop-2-yn-1-ylidene)decahydronaphthalene **11u**:

Compound **11u** (31 mg, 0.18 mmol, 90% yield) was prepared according to general procedure 1 for 2 hours.

Colorless liquid obtained as a >20:1 mixture of E/Z isomers.

Rf = 0.54 (98/2 Hexane/EtOAc).

^{1}H NMR (400 MHz, $CDCl_3$) δ 5.14 (q, *J* = 1.8 Hz, 1H), 3.15 – 3.08 (m, 1H), 2.99 (d, *J* = 2.2 Hz, 1H), 1.90 – 1.73 (m, 4H), 1.73 – 1.59 (m, 4H), 1.44-1.11 (m, 5H), 1.10 – 0.99 (m, 2H).

^{13}C NMR (201 MHz, $CDCl_3$) δ 161.3, 98.0, 82.2, 79.6, 48.0, 45.0, 34.8, 34.4, 32.9, 28.5, 27.4, 26.4, 26.2.

HRMS (APPI/LTQ-Orbitrap) m/z: $[M]^+$ Calcd for $C_{13}H_{18}^+$ 174.1403; Found 174.1402.

IR (νmax, cm-1) 3311 (w), 2918 (s), 2851 (m), 1626 (w), 1444 (m), 900 (w), 828 (w), 631 (m).

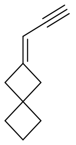

11v

2-(prop-2-yn-1-ylidene)spiro[3.3]heptane **11v**:

Compound **11v** (16.6 mg, 0.13 mmol, 64% yield) was prepared according to general procedure 1 for 1 hour.

Pale yellow liquid.

Rf = 0.68 (9.5/0.5 Hexane/Et_2O).

^{1}H NMR (400 MHz, $CDCl_3$) δ 5.24 (h, *J* = 2.5 Hz, 1H), 2.96 (dt, *J* = 2.0, 0.9 Hz, 1H), 2.79 (d, *J* = 2.7 Hz, 2H), 2.72 (d, *J* = 2.4 Hz, 2H), 2.06-1.99 (m, 4H), 1.87 – 1.81 (m, 2H).

^{13}C NMR (101 MHz, $CDCl_3$) δ 156.1, 101.0, 80.9, 79.1, 44.6, 44.2, 39.1, 34.7, 16.4.

HRMS (Sicrit plasma/LTQ-Orbitrap) m/z: $[M + H]^+$ Calcd for $C_{10}H_{13}^+$ 133.1012; Found 133.1012.

IR (νmax, cm-1) 3303 (m), 2960 (s), 2919 (s), 2850 (m), 1436 (m), 1247 (m), 1051 (m), 912 (m), 843 (m), 748 (m), 677 (m).

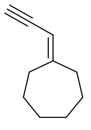

11w

prop-2-yn-1-ylidenecycloheptane **11w**:

Compound **11w** (24.2 mg, 0.18 mmol, 90% yield) was prepared according to general procedure 1 for 45 minutes.

Yellow oil.

Rf = 0.64 (hexane 100%).

^{1}H NMR (400 MHz, $CDCl_3$) δ 5.28-5.26 (m, 1H), 3.04 (d, *J* = 2.2 Hz, 1H), 2.55 (apparent t, *J* = 5.5 Hz, 2H), 2.32 (apparent t, *J* = 5.4 Hz, 2H), 1.68 – 1.56 (m, 4H), 1.56 – 1.46 (m, 4H).

^{13}C NMR (101 MHz, $CDCl_3$) δ 161.0, 103.9, 82.0, 80.2, 37.2, 33.2, 30.1, 29.6, 28.4, 26.9.

HRMS (Sicrit plasma/LTQ-Orbitrap) m/z: $[M + H]^+$ Calcd for $C_{10}H_{15}^+$ 135.1168; Found 135.1165.

IR (ν_{max}, cm^{-1}) 2923 (s), 2854 (m), 2365 (w), 2340 (w), 1461 (w), 1377 (w).

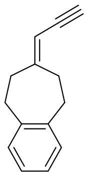

11x

7-(prop-2-yn-1-ylidene)-6,7,8,9-tetrahydro-5*H*-benzo[7]annulene **11x**:

Compound **11x** (32 mg, 0.18 mmol, 88% yield) was prepared according to general procedure 1 for 1 hour.

Beige gum.

Rf = 0.34 (98/2 Hexane/EtOAc).

^{1}H NMR (400 MHz, $CDCl_3$) δ 7.21 – 7.12 (m, 4H), 5.33 (d, *J* = 2.1 Hz, 1H), 3.04 (d, *J* = 2.2 Hz, 1H), 2.84 – 2.79 (m, 4H), 2.67 – 2.64 (m, 2H), 2.38 – 2.35 (m, 2H).

^{13}C NMR (126 MHz, $CDCl_3$) δ 159.6, 142.4, 142.1, 129.3, 129.2, 126.6, 126.5, 103.8, 81.3, 80.1, 38.3, 35.6, 34.8, 33.4.

HRMS (Sicrit plasma/LTQ-Orbitrap) m/z: $[M]^+$ Calcd for $C_{14}H_{14}^+$ 182.1090; Found 182.1090.

IR (ν_{max}, cm^{-1}) 3291 (m), 3019 (w), 2929 (m), 2911 (m), 2840 (w), 1619 (w), 1493 (m), 1451 (m), 943 (m), 836 (w), 754 (s), 663 (s), 644 (m), 609 (m).

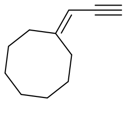

11y

prop-2-yn-1-ylidenecyclooctane **11y**:

Compound **11y** (20.1 mg, 0.14 mmol, 68% yield) was prepared according to general procedure 1 for 1 hour.

Colorless liquid.

Rf = 0.54 (98/2 Hexane/EtOAc).

^{1}H NMR (400 MHz, $CDCl_3$) δ 5.31 (d, *J* = 2.2 Hz, 1H), 3.07 (d, *J* = 2.3 Hz, 1H), 2.47 – 2.44 (m, 2H), 2.26 – 2.23 (m, 2H), 1.79-1.73 (m, 2H), 1.72-1.65 (m, 2H), 1.54-1.44 (m, 6H).

^{13}C NMR (126 MHz, $CDCl_3$) δ 161.9, 104.0, 82.1, 80.4, 36.6, 32.1, 27.4, 26.8, 26.6, 26.5, 25.7.

HRMS (Sicrit plasma/LTQ-Orbitrap) m/z: $[M + H]^+$ Calcd for $C_{11}H_{17}^+$ 149.1325; Found 149.1324.

IR (ν_{max}, cm^{-1}) 3309 (m), 2925 (s), 2854 (m), 1739 (w), 1608 (w), 1467 (m), 1444 (m), 1426 (w), 869 (w), 829 (w), 632 (m).

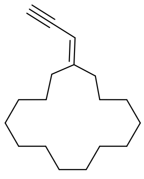

11z

prop-2-yn-1-ylidenecyclopentadecane **11z**:

Compound **11z** (30.7 mg, 0.12 mmol, 62% yield) was prepared according to general procedure 1 for 45 minutes.

Yellow oil.

Rf = 0.66 (hexane 100%).

^{1}H NMR (400 MHz, $CDCl_3$) δ 5.28 (brs, 1H), 3.00 (d, *J* = 2.3 Hz, 1H), 2.33 (t, *J* = 7.5 Hz, 2H), 2.10 (t, *J* = 7.5 Hz, 2H), 1.53 – 1.43 (m, 4H), 1.41 – 1.28 (m, 20H).

^{13}C NMR (101 MHz, $CDCl_3$) δ 159.5, 104.0, 82.1, 79.7, 36.4, 33.2, 27.7, 27.6, 26.9, 26.8, 26.8, 26.7, 26.7, 26.6, 26.6, 26.5.

HRMS (Sicrit plasma/LTQ-Orbitrap) m/z: $[M + H]^+$ Calcd for $C_{18}H_{31}^+$ 247.2420; Found 247.2411.

IR (ν_{max}, cm^{-1}) 3311 (w), 2925 (s), 2855 (m), 1457 (m), 1254 (w), 918 (s), 842 (m).

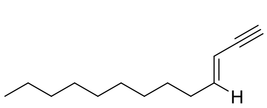

11aa

(*E*)-tridec-3-en-1-yne **11aa**:

Compound **11aa** (18 mg, 0.104 mmol, 52% yield) was prepared according to general procedure 1 for 1 hour.

Colorless liquid obtained as a 1.7:1 mixture of E/Z isomers.

Rf = 0.52 (97/3 Hexane/EtOAc).

^{1}H NMR (400 MHz, $CDCl_3$) δ 6.25 (dt, *J* = 15.9, 7.0 Hz, 1H, major), 6.00 (dtd, *J* = 10.9, 7.5, 0.9 Hz, 1H, minor), 5.49 – 5.40 (m, 1H major + 1H minor), 3.06 (d, *J* = 2.3 Hz, 1H, minor), 2.77 (d, *J* = 2.2 Hz, 1H, major), 2.33 (qd, *J* = 7.3, 1.5 Hz, 2H, minor), 2.10 (qd, *J* = 7.1, 1.7 Hz, 2H, major), 1.45-1.34 (m, 2H major + 2H minor), 1.34-1.20 (m, 12H major + 12H minor), 0.88 (t, *J* = 6.8 Hz, 3H major + 3H minor).

^{13}C NMR (151 MHz, $CDCl_3$) δ 147.2 (major), 146.5 (minor), 108.5 (major), 108.0 (minor), 82.8 (major), 81.3 (minor), 80.7 (minor), 75.6 (major), 33.2 (major), 32.0 (major+minor), 30.4 (minor), 29.7 (minor), 29.7 (major), 29.6 (minor), 29.6 (major), 29.5 (minor), 29.5 (major), 29.3 (minor), 29.2 (major), 28.9 (minor), 28.7 (major), 22.8 (major+minor), 14.3 (major+minor).

HRMS (Sicrit plasma/LTQ-Orbitrap) m/z: $[M - H]^+$ Calcd for $C_{13}H_{21}^+$ 177.1638; Found 177.1637.

IR (ν_{max}, cm^{-1}) 3314 (w), 3296 (w), 2956 (m), 2925 (s), 2875 (w), 2854 (m), 1465 (w), 959 (w).

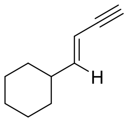

11ab

(*E*)-but-1-en-3-yn-1-ylcyclohexane **11ab**:

Compound **11ab** (19 mg, 0.15 mmol, 73% yield) was prepared according to general procedure 1 for 1 hour.

Colorless liquid obtained as a 2:1 mixture of E/Z isomers.

Rf = 0.69 (96/4 Hexane/EtOAc).

^{1}H NMR (400 MHz, $CDCl_3$) δ 6.21 (dd, *J* = 16.1, 7.0 Hz, 2H), 5.83 (ddd, *J* = 10.6, 9.4, 0.9 Hz, 1H), 5.41 (ddd, *J* = 16.1, 2.2, 1.5 Hz, 2H), 5.35 (ddd, *J* = 10.9, 2.3, 0.8 Hz, 1H), 3.05 (dd, *J* = 2.4, 0.9 Hz, 1H), 2.78 (d, *J* = 2.2 Hz, 2H), 2.69 – 2.56 (m, 1H), 2.08-1.99 (m, 2H), 1.77 – 1.60 (m, 12H), 1.38 – 1.03 (m, 18H).

^{13}C NMR (101 MHz, $CDCl_3$) δ 152.4 (major), 151.8 (minor), 106.3 (major), 106.1 (minor), 83.0 (major), 80.9 (minor), 80.8 (minor), 75.9 (major), 41.3 (major), 39.5 (minor), 32.3 (minor), 32.2 (major), 26.1, 26.1, 25.9, 25.8.

HRMS (Sicrit plasma/LTQ-Orbitrap) m/z: $[M + H]^+$ Calcd for $C_{10}H_{15}^+$ 135.1168; Found 135.1168.

IR (ν_{max}, cm^{-1}) 2957 (s), 2920 (s), 2852 (s), 1738 (w), 1650 (s), 1563 (s), 1540 (s), 1508 (s), 1456 (s), 1350 (m), 1197 (w), 1087 (w), 915 (m), 745 (m).

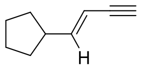

11ac

(*E*)-but-1-en-3-yn-1-ylcyclopentane **11ac**:

Compound **11ac** (17 mg, 0.14 mmol, 71% yield) was prepared according to general procedure 1 for 45 minutes.

Colorless liquid obtained as a 2:1 mixture of E/Z isomers.

Rf = 0.5 (Pentane 100%).

^{1}H NMR (400 MHz, $CDCl_3$) δ 6.24 (dd, *J* = 15.9, 8.0 Hz, 2H), 5.91 (t, *J* = 10.2 Hz, 1H), 5.44 (ddt, *J* = 15.8, 2.2, 1.1 Hz, 2H), 5.36 (dd, *J* = 10.7, 2.3 Hz, 1H), 3.10 – 3.00 (m, 2H), 2.77 (d, *J* = 2.2 Hz, 2H), 2.49 (h, *J* = 8.0 Hz, 2H), 1.95 – 1.84 (m, 2H), 1.84 – 1.73 (m, 3H), 1.73 – 1.49 (m, 10H), 1.38 – 1.20 (m, 9H).

^{13}C NMR (101 MHz, $CDCl_3$) δ 151.5 (major), 151.4 (minor), 106.7 (major), 106.3 (minor), 82.9 (major), 80.9 (minor), 80.9 (minor), 75.8 (major), 43.9 (major), 41.3 (minor), 33.1 (minor), 32.8 (major), 25.6 (minor), 25.3 (major).

HRMS (Sicrit plasma/LTQ-Orbitrap) m/z: $[M]^+$ Calcd for $C_9H_{12}^+$ 120.0934; Found 120.0934.

IR (ν_{max}, cm^{-1}) 2952 (s), 2923 (s), 2858 (s), 1453 (m), 1167 (w), 1096 (w), 886 (w), 740 (w).

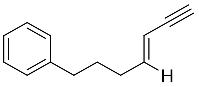

11ad

(*E*)-hept-4-en-6-yn-1-ylbenzene **11ad**:

Compound **11ad** (13.3 mg, 0.078 mmol, 39% yield) was prepared according to general procedure 1 for 1 hour.

Colorless liquid obtained as a 1.3:1 mixture of E/Z isomers.

Rf = 0.52 (96/4 Hexane/EtOAc).

^{1}H NMR (400 MHz, $CDCl_3$) δ 7.31 – 7.26 (m, 2H major + minor), 7.21 – 7.14 (m, 3H, major + minor), 6.27 (dt, *J* = 15.9, 7.0 Hz, 0.6H, major), 6.03 (dt, *J* = 10.8, 7.4 Hz, 0.4H, minor), 5.52 – 5.43 (m, 1H, major + minor), 3.07 (d, *J* = 2.3 Hz, 0.4H, minor), 2.79 (d, *J* = 2.2 Hz, 0.6H, major), 2.64 (dt, *J* = 9.9, 7.7 Hz, 2H, major + minor), 2.41 – 2.36 (m, 0.8H, minor), 2.18 – 2.12 (m, 1.2H, major), 1.79 – 1.70 (m, 2H, major + minor).

^{13}**C NMR** (101 MHz, $CDCl_3$) δ 146.4, 145.7, 142.3, 142.1, 128.6, 128.6, 128.5, 128.4, 126.0, 125.9, 109.1, 108.7, 82.6, 81.6, 80.6, 75.9, 35.6, 35.3, 32.6, 30.7, 30.3, 30.1.

HRMS (Sicrit plasma/LTQ-Orbitrap) m/z: $[M + H]^+$ Calcd for $C_{13}H_{15}^+$ 171.1168; Found 171.1168.

IR (ν_{max}, cm^{-1}) 3315 (w), 3292 (m), 3026 (m), 2952 (m), 2925 (s), 2854 (m), 1749 (w), 1496 (m), 1453 (m), 1361 (w), 1226 (w), 957 (m), 744 (m), 699 (s).

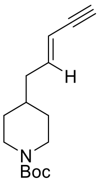

11ae

tert-butyl (*E*)-4-(pent-2-en-4-yn-1-yl)piperidine-1-carboxylate **11ae**:

Compound **11ae** (36.2 mg, 0.15 mmol, 73% yield) was prepared according to general procedure 1 for 45 minutes.

Colorless liquid obtained as a 1:1 mixture of E/Z isomers.

Rf = 0.44 (8/2 Hexane/EtOAc).

^{1}**H NMR** (400 MHz, $CDCl_3$) δ 6.21 (dt, *J* = 15.4, 7.5 Hz, 1H), 6.03 – 5.97 (m, 1H), 5.52 (ddt, *J* = 10.9, 2.5, 1.3 Hz, 1H), 5.46 (dq, *J* = 15.8, 1.7 Hz, 1H), 4.07 (brs, 4H), 3.07 (d, *J* = 2.3 Hz, 1H), 2.79 (d, *J* = 2.2 Hz, 1H), 2.75 – 2.60 (m, 4H), 2.31 – 2.25 (m, 2H), 2.06 (td, *J* = 7.2, 1.5 Hz, 2H), 1.70-1.41 (m, 5H), 1.45 (brs, 18H), 1.30-1.04 (m, 5H).

^{13}**C NMR** (101 MHz, $CDCl_3$) δ 155.0, 144.4, 143.6, 110.3, 109.7, 82.4, 81.7, 80.6, 79.4, 79.4, 76.1, 44.0, 40.1, 37.0, 36.2, 36.1, 32.0, 28.6.

HRMS (ESI/QTOF) m/z: $[M + Na]^+$ Calcd for $C_{15}H_{23}NNaO_2^+$ 272.1621; Found 272.1614.

IR (ν_{max}, cm^{-1}) 3285 (w), 3238 (w), 2971 (w), 2930 (w), 2851 (w), 1449 (w), 1422 (m), 1365 (m), 1277 (m), 1240 (m), 1171 (s), 1130 (m), 1079 (w), 965 (w), 864 (w), 765 (w).

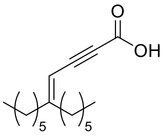

12a

5-hexylundec-4-en-2-ynoic acid **12a**:

Compound **12a** (44 mg, 0.18 mmol, 85% yield) was prepared according to general procedure 3.

Yellow liquid.

Rf = 0.39 (2/8 MeOH/EtOAc).

^{1}**H NMR** (400 MHz, CD_3OD) δ 5.43 (apparent t, *J* = 1.5 Hz, 1H), 2.42-2.38 (m, 2H), 2.22 – 2.17 (m, 2H), 1.53 – 1.43 (m, 4H), 1.38-1.27 (m, 12H), 0.94 – 0.88 (m, 6H).

^{13}**C NMR** (151 MHz, CD_3OD) δ 166.9, 157.2, 103.2, 85.7, 85.1, 37.3, 34.4, 32.8, 32.7, 30.1, 30.1, 29.0, 28.7, 23.7, 23.6, 14.4.

HRMS (ESI/QTOF) m/z: $[M - H]^-$ Calcd for $C_{17}H_{27}O_2^-$ 263.2017; Found 263.2022.

IR (ν_{max}, cm^{-1}) 2926 (s), 2856 (m), 2196 (w), 1691 (m), 1461 (m), 1368 (m), 1263 (s).

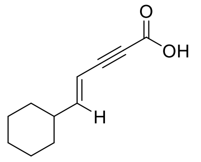

12b

(*E*)-5-cyclohexylpent-4-en-2-ynoic acid **12b**:

Compound **12b** (30 mg, 0.18 mmol, 86% yield) was prepared according to general procedure 3.

Orange liquid obtained as a 2:1 mixture of E/Z isomers.

Rf = 0.71 (8/2 EtOAc/MeOH+1%AcOH).

^{1}H NMR (600 MHz, CD_3OD) δ 6.38 (dd, *J* = 16.1, 7.1 Hz, 1H), 6.04 (dd, *J* = 10.9, 9.7 Hz, 0.5H), 5.59 (dd, *J* = 16.1, 1.5 Hz, 1H), 5.49 (dd, *J* = 10.9, 0.8 Hz, 0.5H), 2.70 – 2.62 (m, 0.5H), 2.12 (qdd, *J* = 10.1, 3.4, 1.6 Hz, 1H), 1.78-1.65 (m, 6.5H), 1.72-1.10 (m, 8.5H).

^{13}C NMR (151 MHz, CD_3OD) δ 158.9, 158.8, 157.0, 155.8, 106.4, 105.9, 87.6, 84.4, 82.5, 81.6, 42.8, 41.0, 33.2, 33.0, 27.0, 26.9, 26.8, 26.6.

HRMS (Sicrit plasma/LTQ-Orbitrap) m/z: $[M + H]^+$ Calcd for $C_{11}H_{15}O_2^+$ 179.1067; Found 179.1066.

IR (ν_{max}, cm^{-1}) 2926 (s), 2851 (m), 2204 (m), 1683 (m), 1621 (m), 1568 (m), 1449 (m), 1390 (m), 1369 (m), 1273 (m), 1255 (m), 960 (m).

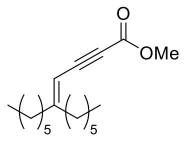

12c

methyl 5-hexylundec-4-en-2-ynoate **12c**:

Compound **12c** (28 mg, 0.11 mmol, 54% yield) was prepared according to general procedure 2 with dimethylcarbonate for 4 hours.

Orange gum.

Rf = 0.33 (9.5/0.5 Hexane/EtOAc).

^{1}H NMR (400 MHz, $CDCl_3$) δ 5.35 (apparent t, *J* = 1.4 Hz, 1H), 3.77 (s, 3H), 2.36 (dd, *J* = 8.6, 6.7 Hz, 2H), 2.13 (td, *J* = 7.6, 1.3 Hz, 2H), 1.47 – 1.39 (m, 4H), 1.34 – 1.25 (m, 12H), 0.90-0.85 (m, 6H).

^{13}C NMR (151 MHz, $CDCl_3$) δ 166.4, 155.0, 102.1, 85.9, 83.4, 52.6, 36.5, 33.9, 31.8, 31.7, 29.2, 29.1, 28.1, 27.6, 22.7, 14.2, 14.2.

HRMS (Sicrit plasma/LTQ-Orbitrap) m/z: $[M + H]^+$ Calcd for $C_{18}H_{31}O_2^+$ 279.2319; Found 279.2318.

IR (ν_{max}, cm^{-1}) 2959 (w), 2929 (m), 2899 (w), 2851 (w), 2202 (m), 1712 (s), 1615 (w), 1467 (w), 1433 (m), 1262 (s), 1124 (w), 1084 (w), 828 (w), 748 (w).

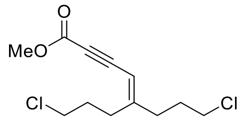

12d

methyl 8-chloro-5-(3-chloropropyl)oct-4-en-2-ynoate **12d**:

Compound **12d** (100 mg, 0.38 mmol, 76% yield) was prepared according to general procedure 2 with dimethylcarbonate for 4 hours.

Yellow liquid.

Rf = 0.31 (8.5/1.5 Hexane/EtOAc).

^{1}H NMR (500 MHz, $CDCl_3$) δ 5.47 (apparent t, *J* = 1.5 Hz, 1H), 3.79 (s, 3H), 3.56 (t, *J* = 6.5 Hz, 2H), 3.54 (t, *J* = 6.5 Hz, 2H), 2.54-2.51 (m, 2H), 2.34 (td, *J* = 7.5, 1.4 Hz, 2H), 2.01 – 1.88 (m, 4H).

^{13}C NMR (126 MHz, $CDCl_3$) δ 161.6, 154.7, 104.5, 84.4, 84.1, 52.8, 44.4, 44.1, 33.5, 31.3, 31.1, 30.3.

HRMS (Sicrit plasma/LTQ-Orbitrap) m/z: $[M + H]^+$ Calcd for $C_{12}H_{17}Cl_2O_2^+$ 263.0600; Found 263.0595.

IR (ν_{max}, cm^{-1}) 2955 (w), 2914 (w), 2209 (m), 2198 (m), 1708 (s), 1616 (w), 1434 (m), 1260 (s), 1103 (m), 832 (w), 748 (w), 665 (w).

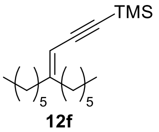

12f

(4-hexyldec-3-en-1-yn-1-yl)trimethylsilane **12f**:

Compound **12f** (49 mg, 0.18 mmol, 85% yield) was prepared according to general procedure 2 with TMSCl for 4 hours.

Colorless liquid.

Rf = 0.57 (Hexane 100%).

^{1}H NMR (400 MHz, $CDCl_3$) δ 5.28 (apparent t, *J* = 1.4 Hz, 1H), 2.32 – 2.28 (m, 2H), 2.08 – 2.03 (m, 2H), 1.48-1.35 (m, 4H), 1.33 – 1.21 (m, 12H), 0.91-0.86 (m, 6H) 0.18 (s, 9H).

^{13}C NMR (151 MHz, $CDCl_3$) δ 159.2, 104.6, 103.9, 96.5, 36.3, 33.4, 31.9, 31.8, 29.3, 29.2, 27.9, 27.8, 22.8, 22.8, 14.25, 14.23, 0.3.

HRMS (Sicrit plasma/LTQ-Orbitrap) m/z: $[M + H]^+$ Calcd for $C_{19}H_{37}Si^+$ 293.2659; Found 293.2659.

IR (ν_{max}, cm^{-1}) 3728 (m), 3694 (m), 3626 (m), 2956 (s), 2921 (s), 2854 (m), 1739 (s), 1458 (m), 1369 (s), 1213 (m), 909 (m), 842 (s), 746 (m), 673 (m), 655 (m), 635 (s).

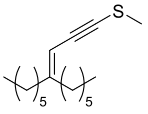

12g

(4-hexyldec-3-en-1-yn-1-yl)(methyl)sulfane **12g**:

Compound **12g** (38 mg, 0.14 mmol, 72% yield) was prepared according to general procedure 2 with *S*-methyl methanethiosulfonate for 4 hours.

Colorless liquid.

Rf = 0.50 (Hexane 100%).

^{1}H NMR (400 MHz, $CDCl_3$) δ 5.32 (apparent t, *J* = 1.4 Hz, 1H), 2.40 (s, 3H), 2.28 (dd, *J* = 8.8, 6.5 Hz, 2H), 2.09 – 2.03 (m, 2H), 1.44 – 1.37 (m, 4H), 1.32 – 1.24 (m, 12H), 0.91 – 0.86 (m, 6H).

^{13}C NMR (151 MHz, $CDCl_3$) δ 157.5, 104.7, 90.4, 82.0, 36.1, 33.3, 31.9, 31.8, 29.3, 29.2, 28.0, 27.9, 22.8, 22.8, 19.9, 14.3, 14.2.

HRMS (Sicrit plasma/LTQ-Orbitrap) m/z: $[M + H]^+$ Calcd for $C_{17}H_{31}S^+$ 267.2141; Found 267.2141.

IR (ν_{max}, cm^{-1}) 2955 (m), 2927 (s), 2855 (m), 2372 (w), 1461 (w), 1433 (w), 1083 (w), 822 (w), 750 (w), 627 (w).

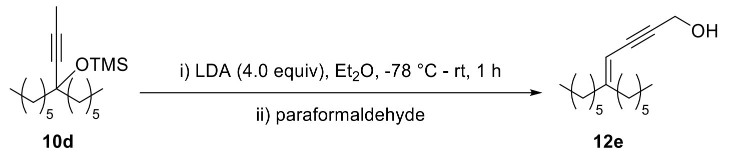

Diisopropylamine was distilled over CaH_2. In an oven-dried flask containing dry THF (0.95 mL) at -78 °C and diisopropylamine (0.28 mL, 2 mmol, 1.0 equiv) was added dropwise *n*BuLi (0.8 mL, 2 mmol, 1.0 equiv). The reaction mixture was stirred at -78 °C for 30 min. In a second vial was added the protected propargylic alcohol (62 mg, 0.2 mmol, 1.0 equiv) and Et_2O (1.33 mL). The reaction mixture was cooled to -78 °C and the freshly prepared LDA (0.8 mL, 0.8 mmol, 4.0 equiv, [1 M]) was added dropwise. The reaction mixture was then stirred at room temperature for 1 hour. In an oven dried flask, paraformaldehyde was heated and cannuled into the reaction mixture for 5 minutes. Then, the reaction mixture was stirred for an additional 3 hours. The reaction mixture was poured into water and ice. The mixture was extracted three times with EtOAc. The combined organic layers were washed with brine, dried over Na_2SO_4, filtered, and concentrated *in vacuo.* The reaction mixture was then purified by flash column chromatography on silica gel (pentane/Et_2O 8/2) to afford the pure desired product **12e** (45 mg, 0.18 mmol, 90% yield) as a colorless liquid.

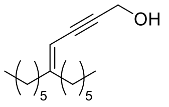

12e

5-hexylundec-4-en-2-yn-1-ol **12e**:

Rf = 0.48 (8/2 Hexane/EtOAc).

^{1}H NMR (400 MHz, $CDCl_3$) δ 5.28 (p, *J* = 1.7 Hz, 1H), 4.41 (dd, *J* = 6.1, 2.1 Hz, 2H), 2.30-2.26 (m, 2H), 2.06 (td, *J* = 7.6, 1.3 Hz, 2H), 1.52 (t, *J* = 6.1 Hz, 1H, OH), 1.46 – 1.36 (m, 4H), 1.34 – 1.25 (m, 12H), 0.91-0.85 (m, 6H).

^{13}C NMR (151 MHz, $CDCl_3$) δ 158.0, 103.8, 89.5, 84.1, 52.0, 36.1, 33.1, 31.8, 31.8, 29.3, 29.2, 27.9, 27.8, 22.7, 14.3, 14.2.

HRMS (Sicrit plasma/LTQ-Orbitrap) m/z: $[M + H]^+$ Calcd for $C_{17}H_{31}O^+$ 251.2369; Found 251.2369.

IR (ν_{max}, cm^{-1}) 3339 (w), 2955 (m), 2922 (s), 2870 (m), 2856 (m), 1458 (w), 1381 (w), 1355 (w), 1162 (w), 1010 (m).

6) Mechanistic studies

Procedure of the control experiment:

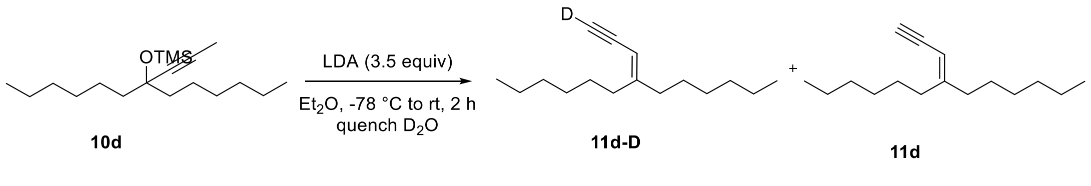

Diisopropylamine was distilled over CaH_2. In an oven-dried flask containing dry THF (0.95 mL) at -78 °C and diisopropylamine (0.28 mL, 2 mmol, 1.0 equiv) was added dropwise *n*BuLi (0.8 mL, 2 mmol, 1.0 equiv). The reaction mixture was stirred at -78 °C for 30 min. In a second vial was added **10d** (62 mg, 0.2 mmol, 1.0

equiv) and Et_2O (1.33 mL). The reaction mixture was cooled to -78 °C and the freshly prepared LDA (0.7 mL, 0.7 mmol, 3.0 equiv, [1 M]) was added dropwise. The reaction mixture was then stirred at room temperature for 2 hours. Water-d_2 (1.0 mL) was added to the reaction mixture. The mixture was extracted three times with Et_2O. The combined organic layers were washed with brine, dried over Na_2SO_4, filtered, and concentrated *in vacuo* to afford a mixture of **11d-D** and **11d** in a 5.5/1 ratio.

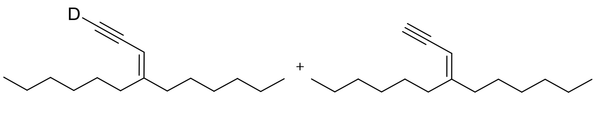

11d-D 11d

Crude containing 7-(prop-2-yn-1-ylidene-3-*d*)tridecane/7-(prop-2-yn-1-ylidene)tridecane in 5.5/1 ratio.

Colorless liquid.

Rf = 0.54 (hexane 100%).

^{1}H NMR (400 MHz, $CDCl_3$) δ 5.29 (brs, 0.2H), 5.23 (apparent t, J = 1.5 Hz, 1H), 2.98 (d, J = 2.3 Hz, 0.2H), 2.32 – 2.28 (m, 2H + 0.4H), 2.08-2.04 (m, 2H + 0.4H), 1.47 – 1.23 (m, 16H + 3.2H), 0.90-0.86 (m, 6H + 1.2H).

^{13}C NMR (151 MHz, $CDCl_3$) δ 159.22, 159.18, 103.4, 82.0, 81.5 (t, J = 7.3 Hz), 79.4, 79.2 (t, J = 38.1 Hz), 36.1, 33.2, 31.8, 31.8, 29.3, 29.1, 27.9, 27.7, 22.7, 22.7, 14.2, 14.2.

HRMS (Sicrit plasma/LTQ-Orbitrap) m/z: $[M + H]^+$ Calcd for $C_{16}H_{28}[_2H]^+$ 222.2327; Found 222.2325.

IR (ν_{max}, cm^{-1}) 3313 (w), 2955 (m), 2929 (s), 2856 (m), 2585 (w), 1621 (w), 1461 (m), 1377 (w), 830 (w), 725 (w).

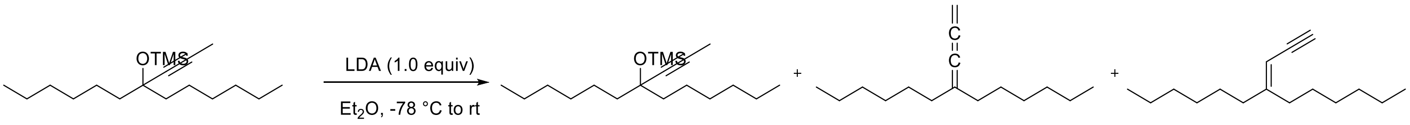

10d → 10d 71% + 20a 0.9% + 11d 15%

Diisopropylamine was distilled over CaH_2. In an oven-dried flask containing dry THF (1.9 mL) at -78 °C and diisopropylamine (0.56 mL, 4 mmol, 1.0 equiv) was added dropwise *n*BuLi (1.6 mL, 4 mmol, 1.0 equiv). The reaction mixture was stirred at -78 °C for 30 min. In a second vial was added **10d** (620 mg, 2 mmol, 1.0 equiv) and Et_2O (13.3 mL). The reaction mixture was cooled to -78 °C and the freshly prepared LDA (2 mL, 2 mmol, 1.0 equiv, [1 M]) was added dropwise. The reaction mixture was then stirred at room temperature for 25 minutes. The reaction mixture was poured into a saturated solution of NH_4Cl and ice. The mixture was extracted three times with Et_2O. The combined organic layers were washed with brine, dried over Na_2SO_4, filtered, and concentrated *in vacuo.* The reaction mixture was then purified by flash column chromatography on silica gel (pentane 100%) to afford the pure desired product **10d** as a colorless oil (440 mg, 1.42 mmol, 71% yield), **20a** as a colorless oil (3.96 mg, 0.018 mmol, 0.9% yield) and **11d** as a colorless oil (67 mg, 0.3 mmol, 15% yield).

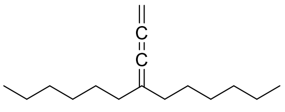

20a

7-(propa-1,2-dien-1-ylidene)tridecane **20a**:

Colorless oil.

^{1}H NMR (800 MHz, $CDCl_3$) δ 4.93 (p, J = 1.4 Hz, 2H), 2.20 (tt, J = 7.7, 1.3 Hz, 4H), 1.56 – 1.51 (m, 4H), 1.34 – 1.28 (m, 12H), 0.95 – 0.89 (m, 6H).

^{13}C NMR (201 MHz, $CDCl_3$) δ 169.1, 154.5, 129.2, 85.2, 37.0, 31.9, 29.1, 27.7, 22.8, 14.2.

Rf = 0.68 (100% Hexane).

HRMS (Sicrit plasma/LTQ-Orbitrap) m/z: $[M + H]^+$ Calcd for $C_{16}H_{29}^+$ 221.2264; Found 221.2264.

IR (ν_{max}, cm^{-1}) 2955 (m), 2933 (s), 2925 (s), 2873 (m), 2855 (m), 1460 (w), 1377 (w), 1252 (w), 1080 (w), 1040 (w), 914 (m), 843 (w), 817 (w).

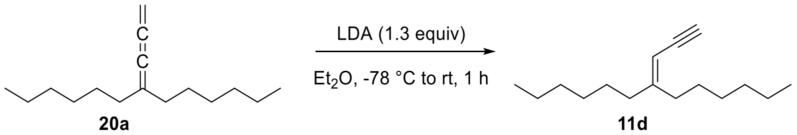

Diisopropylamine was distilled over CaH_2. In an oven-dried flask containing dry THF (0.95 mL) at -78 °C and diisopropylamine (0.28 mL, 2 mmol, 1.0 equiv) was added dropwise *n*BuLi (0.8 mL, 2 mmol, 1.0 equiv). The reaction mixture was stirred at -78 °C for 30 min. In a second vial was added **20a** (5 mg, 0.023 mmol, 1.0 equiv) and Et_2O (0.15 mL). The reaction mixture was cooled to -78 °C and the freshly prepared LDA (0.03 mL, 0.03 mmol, 1.3 equiv, [1 M]) was added dropwise. The reaction mixture was then stirred at room temperature for 1 hour. The reaction mixture was poured into a saturated solution of NH_4Cl and ice. The mixture was extracted three times with Et_2O. The combined organic layers were washed with brine, dried over Na_2SO_4, filtered, and concentrated *in vacuo.* The reaction mixture was then purified by flash column chromatography on silica gel (pentane 100%) to afford the pure desired product **11d** as a colorless oil (4 mg, 0.018 mmol, 80% yield).

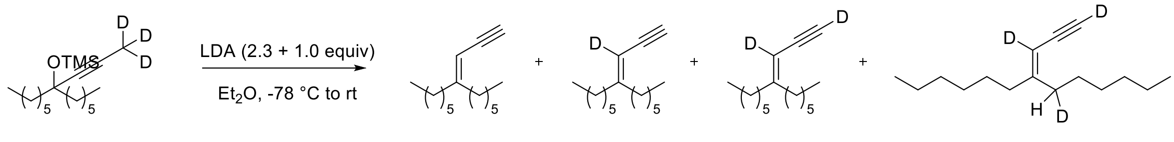

Diisopropylamine was distilled over CaH_2. In an oven-dried flask containing dry THF (0.95 mL) at -78 °C and diisopropylamine (0.27 mL, 1.9 mmol, 0.95 equiv) was added dropwise *n*BuLi (0.91 mL, 2 mmol, 1.0 equiv). The reaction mixture was stirred at -78 °C for 30 min. In a second vial, was added **10d-D₃** (69 mg, 0.22 mmol, 1.0 equiv) and Et_2O (1.5 mL). The reaction mixture was cooled to -78 °C and the freshly prepared LDA (0.55 mL, 0.51 mmol, 2.3 equiv, [0.94 M]) was added dropwise. The reaction mixture was then stirred at room temperature for 1 hour. The reaction mixture was cooled to -78 °C and the freshly prepared LDA (0.24 mL, 0.22 mmol, 1.0 equiv, [0.94 M]) was added dropwise. The reaction mixture was then stirred at room temperature for 1 hour. The reaction mixture was poured into a saturated solution of NH_4Cl and ice. The mixture was extracted three times with Et_2O. The combined organic layers were washed with brine, dried over Na_2SO_4, filtered, and concentrated *in vacuo.* The reaction mixture was then purified by flash column chromatography on silica gel (pentane 100%) to afford an inseparable mixture of product containing 0, 1, 2 and 3 deuterium(s).

^{1}H NMR (400 MHz, $CDCl_3$) δ 5.24 (q, J = 1.8 Hz, 0.45H), 2.98 (d, J = 2.4 Hz) + 2.98 (s) (0.82H), 2.33 – 2.29 (m, 2H), 2.09 – 2.05 (m, 2H), 1.48 – 1.36 (m, 4H), 1.36-1.22 (m, 12H), 0.91-0.85 (m, 6H).

^{13}C NMR (126 MHz, $CDCl_3$) δ 159.22, 159.12, 103.45, 103.17 (t, J = 25.1 Hz), 82.02, 81.99, 79.46, 79.20 (t, J = 37.7 Hz), 36.11, 36.05, 33.25, 33.22, 31.85, 31.83, 29.34, 29.18, 27.95, 27.74, 27.73, 22.76, 22.76, 14.25, 14.23.

Based on the integration of 5.24 (q, J = 1.8 Hz, 0.45H), the deuterated alkene **14** is 55%, corresponding to the alkyne H at 2.98 (s) ppm. The alkyne H at 2.98 (d, J = 2.4 Hz) corresponding to the non-deuterated enyne (37%).

Based on the intensity of HRMS spectra, the mixture contains 44.2% of 0 deuterium incorporation, 46% of 1 deuterium incorporation, 8.8% of 2 deuterium incorporation and 1% of 3 deuterium incorporation.

HRMS (Sicrit plasma/LTQ-Orbitrap) m/z: $[M + H]^+$ Calcd for $C_{16}H_{29}^+$ 221.2264; Found 221.2262.

HRMS (Sicrit plasma/LTQ-Orbitrap) m/z: $[M + H]^+$ Calcd for $C_{16}H_{28}[^2H]^+$ 222.2327; Found 222.2325.

HRMS (Sicrit plasma/LTQ-Orbitrap) m/z: $[M + H]^+$ Calcd for $C_{16}H_{27}[^2H]_2^+$ 223.2389; Found 223.2388.

HRMS (Sicrit plasma/LTQ-Orbitrap) m/z: $[M + H]^+$ Calcd for $C_{16}H_{26}[^2H]_3^+$ 224.2452; Found 224.2451.

7) Product modifications

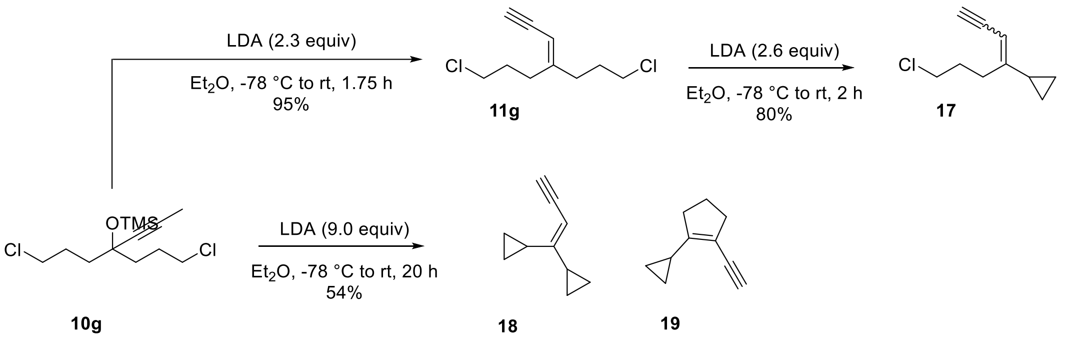

Diisopropylamine was distilled over CaH_2. In an oven-dried flask containing dry THF (0.95 mL) at -78 °C and diisopropylamine (0.28 mL, 2 mmol, 1.0 equiv) was added dropwise *n*BuLi (0.8 mL, 2 mmol, 1.0 equiv). The reaction mixture was stirred at -78 °C for 30 min. In a second vial was added **11g** (30.77 mg, 0.15 mmol, 1.0 equiv) and Et_2O (1 mL). The reaction mixture was cooled to -78 °C and the freshly prepared LDA (0.39 mL, 0.39 mmol, 2.6 equiv, [1 M]) was added dropwise. The reaction mixture was then stirred at room temperature for 2 hours. The reaction mixture was poured into a saturated solution of NH_4Cl and ice. The mixture was extracted three times with Et_2O. The combined organic layers were washed with brine, dried over Na_2SO_4, filtered, and concentrated *in vacuo.* The reaction mixture was then purified by flash column chromatography on silica gel (pentane/Et_2O 96/4) to afford the pure desired product **17** as a colorless liquid (20 mg, 0.12 mmol, 80% yield).

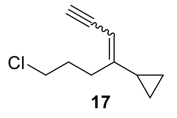

(7-chlorohept-3-en-1-yn-4-yl)cyclopropane **17**:

Rf = 0.29 (98.5/1.5 Hexane/EtOAc).

^{1}H NMR (400 MHz, $CDCl_3$) δ 5.33 (d, J = 2.3 Hz, 1H), 3.56 – 3.51 (m, 2H), 3.10 (d, J = 2.3 Hz, 1H), 2.14 (tt, J = 8.5, 5.3 Hz, 1H), 1.92 – 1.83 (m, 4H), 0.84 – 0.79 (m, 2H), 0.70-0.66 (m, 2H).

^{13}C NMR (101 MHz, $CDCl_3$) δ 156.4, 104.0, 81.6, 80.9, 44.5, 31.5, 28.1, 15.0, 5.9.

HRMS (Sicrit plasma/LTQ-Orbitrap) m/z: $[M + H]^+$ Calcd for $C_{10}H_{14}Cl^+$ 169.0779; Found 169.0778.

IR (ν_{max}, cm^{-1}) 3292 (s), 3080 (w), 3018 (w), 2960 (m), 2934 (m), 2901 (w), 2095 (w), 1603 (m), 1442 (m), 1303 (m), 1067 (w), 1043 (w), 926 (s), 869 (m), 823 (s).

Diisopropylamine was distilled over CaH_2. In an oven-dried flask containing dry THF (0.95 mL) at -78 °C and diisopropylamine (0.28 mL, 2 mmol, 1.0 equiv) was added dropwise *n*BuLi (0.8 mL, 2 mmol, 1.0 equiv). The reaction mixture was stirred at -78 °C for 30 min. In a second vial was added **10g** (59 mg, 0.2 mmol, 1.0 equiv) and Et_2O (1.33 mL). The reaction mixture was cooled to -78° C and the freshly prepared LDA (1.0 mL, 1 mmol, 5.0 equiv, [1 M]) was added dropwise. The reaction mixture was then stirred at room temperature for 3 hours. The reaction mixture was cooled to -78 °C and the freshly prepared LDA (0.8 mL, 0.8 mmol, 4.0 equiv, [1 M]) was added dropwise. The reaction mixture was then stirred at room temperature for 17 hours. The reaction mixture was poured into a saturated solution of NH_4Cl and ice. The mixture was extracted three times with Et_2O. The combined organic layers were washed with brine, dried over Na_2SO_4, filtered, and concentrated *in vacuo.* The reaction mixture was then purified by flash column chromatography on silica gel (pentane 100%) to afford an inseparable mixture of **18** and **19** in a 1.4/1 ratio as a colorless liquid (14 mg, 0.11 mmol, 54% yield).

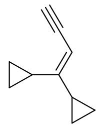

18

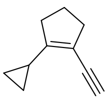

19

but-1-en-3-yne-1,1-diyldicyclopropane **18** (major) and 1-cyclopropyl-2-ethynylcyclopent-1-ene **19** (minor):

ratio: 1.4/1

Rf = 0.57 (97/3 Hexane/EtOAc).

18:

^{1}H NMR (800 MHz, $CDCl_3$) δ 5.10 (dd, J = 2.4, 1.2 Hz, 1H), 3.09 (d, J = 2.3 Hz, 1H), 2.27 (tt, J = 8.5, 5.2 Hz, 1H), 0.93 – 0.82 (m, 3H), 0.82 – 0.79 (m, 2H), 0.66 – 0.62 (m, 2H), 0.45 – 0.42 (m, 2H).

^{13}C NMR (201 MHz, $CDCl_3$) δ 159.2, 100.0, 82.2, 80.8, 15.9, 11.6, 6.3, 6.0.

19:

^{1}H NMR (800 MHz, $CDCl_3$) δ 3.17 (s, 1H), 2.50 (tt, J = 7.5, 2.1 Hz, 2H), 2.05 (tt, J = 7.5, 2.1 Hz, 2H), 2.02 (td, J = 8.6, 4.2 Hz, 1H), 1.82 (p, J = 7.5 Hz, 2H), 0.78 – 0.75 (m, 2H), 0.66 – 0.62 (m, 2H).

^{13}C NMR (201 MHz, $CDCl_3$) δ 155.0, 115.8, 81.5, 80.8, 36.9, 31.4, 22.3, 12.6, 5.7.

HRMS (Sicrit plasma/LTQ-Orbitrap) m/z: $[M + H]^+$ Calcd for $C_{10}H_{13}^+$ 133.1012; Found 133.1013.

IR (ν_{max}, cm^{-1}) 3307 (m), 3086 (w), 3007 (w), 2966 (w), 2923 (m), 2851 (m), 2090 (w), 1604 (w), 1467 (w), 1422 (w), 1236 (w), 1181 (w), 1035 (m), 988 (w), 926 (m), 865 (m), 813 (m), 635 (s), 620 (m).

8) References

(1) Martin, P.; Mueller, M.; Flubacher, D.; Boudier, A.; Blaser, H.-U.; Spielvogel, D. Total Synthesis of Hematoporphyrin and Protoporphyrin: A Conceptually New Approach. *Org. Process Res. Dev.* **2010**, *14*, 799–804.

(2) Vechorkin, O.; Godinat, A.; Scopelliti, R.; Hu, X. Cross-Coupling of Nonactivated Alkyl Halides with Alkynyl Grignard Reagents: A Nickel Pincer Complex as the Catalyst. *Angew. Chem. Int. Ed.* **2011**, *50*, 11777–11781.

(3) Nicolaou, K. C.; Ortiz, A.; Zhang, H.; Guella, G. Total Synthesis and Structural Revision of Vannusals A and B: Synthesis of the True Structures of Vannusals A and B. *J. Am. Chem. Soc.* **2010**, *132*, 7153–7176.

(4) Cabezas, J. A.; Poveda, R. R.; Brenes, J. A. One-Pot Conversion of Aldehydes and Ketones into 1-Substituted and 1,4-Disubstituted 1,3-Enynes. *Synthesis* **2018**, *50*, 3307–3321.

9) X-Ray crystallographic data

X-ray crystallographic data of (*Z*)-**11h** (CCDC 2443046):

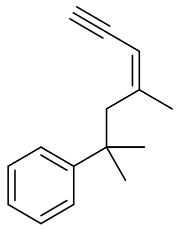

(*Z*)-**11h**

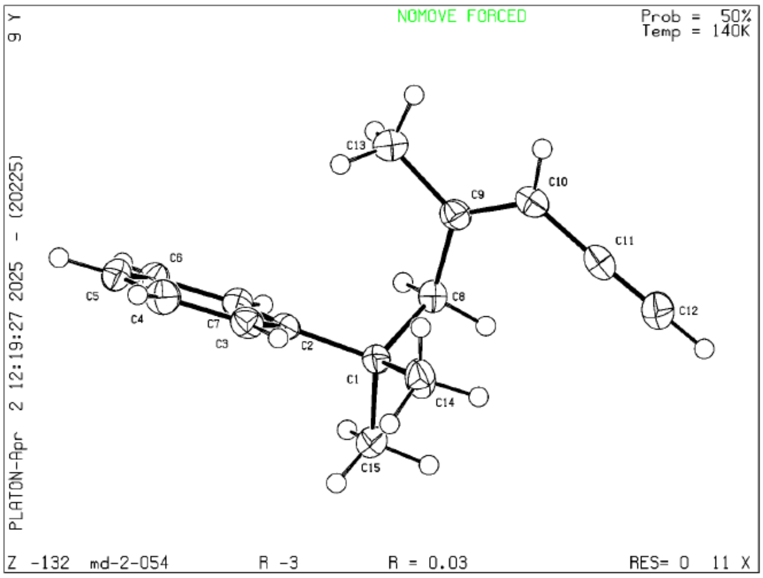

(*Z*)-10h

Report created with ReportPlus

Submitted by: **Morgane Elodie Marie Delattre**

***R*₁=3.43%**

Supervisor: **Professor Jieping Zhu**

Solved by: **Farzaneh Fadaei Tirani**

Sample ID: (*Z*)-**11h**

Crystal Data and Experimental

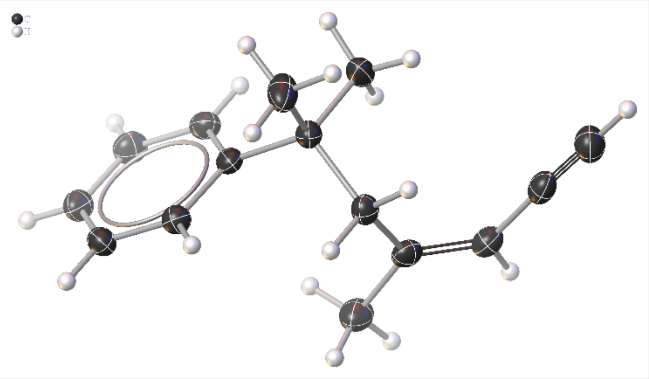

Ortep picture of (*Z*)-11h. The ellipsoids are displayed at 50% probability level.

Experimental. Single colourless prism-shaped crystals of (*Z*)-**11h** were used as supplied. A suitable crystal with dimensions 0.29 × 0.08 × 0.07 mm³ was selected and mounted on an XtaLAB Synergy R, DW system, HyPix-Arc 150 diffractometer. The crystal was kept at a steady T = 140.00(10) K during data collection. The structure was solved with the ShelXT 2018/2 (Sheldrick, 2015) solution program using dual methods and by using Olex2 1.5 (Dolomanov et al., 2009) as the graphical interface. The model was refined with ShelXL 2019/3 (Sheldrick, 2015) using full matrix least squares minimisation on F^2.

Crystal Data. $C_{15}H_{18}$, M_r = 198.29, trigonal, $R\bar{3}$ (No. 148), a = 25.1351(4) Å, b = 25.1351(4) Å, c = 9.9869(2) Å, α = 90°, β = 90°, γ = 120°, V = 5464.2(2) Å³, T = 140.00(10) K, Z = 18, Z' = 1, μ(Cu K$_\alpha$) = 0.448, 8315 reflections measured, 2390 unique (R_{int} = 0.0134) which were used in all calculations. The final wR_2 was 0.0872 (all data) and R_1 was 0.0343 (I≥2σ(I)).

**Compound**	(*Z*)-**11h**
Formula	$C_{15}H_{18}$
$D_{calc.}$/ g cm^{-3}	1.085
μ/mm^{-1}	0.448
Formula Weight	198.29
Colour	colourless
Shape	prism-shaped
Size/mm³	0.29×0.08×0.07
T/K	140.00(10)
Crystal System	trigonal
Space Group	$R\bar{3}$
a/Å	25.1351(4)
b/Å	25.1351(4)
c/Å	9.9869(2)
α/°	90
β/°	90
γ/°	120
V/Å³	5464.2(2)
Z	18
Z'	1
Wavelength/Å	1.54184
Radiation type	Cu$K\alpha$
Θ_{min}/°	3.517
Θ_{max}/°	74.564
Measured Refl's.	8315
Indep't Refl's	2390
Refl's I≥2σ(I)	2159
R_{int}	0.0134
Parameters	140
Restraints	0
Largest Peak/e Å^{-3}	0.239
Deepest Hole/e Å^{-3}	-0.165
GooF	1.066
wR_2 (all data)	0.0872
wR_2	0.0852
R_1 (all data)	0.0381
R_1	0.0343
CCDC number	2443046

Structure Quality Indicators

**Reflections:**	d min (CuKα) 2Θ=149.1° .cif 0.80	I/σ(I) .cif 69.9	Rint m=3.48 .cif 1.34%	Full 135.4° 96% to 149.1° .cif 99.7
**Refinement:**	Shift .cif 0.001	Max Peak .cif 0.2	Min Peak .cif -0.2	GooF .cif 1.066

A colourless prism-shaped crystal with dimensions 0.29 × 0.08 × 0.07 mm³ was mounted. Data were collected using an XtaLAB Synergy R, DW system, HyPix-Arc 150 diffractometer operating at T = 140.00(10) K.

Data were measured using ω scans with CuK_α radiation. The diffraction pattern was indexed and the total number of runs and images was based on the strategy calculation from the program CrysAlisPro system (CCD 44.89a 64-bit (release 14-01-2025)). The maximum resolution achieved was Θ = 74.564° (0.80 Å).

The unit cell was refined using CrysAlisPro 1.171.44.88a (Rigaku OD, 2025) on 2956 reflections, 36% of the observed reflections.

Data reduction, scaling and absorption corrections were performed using CrysAlisPro 1.171.44.88a (Rigaku OD, 2025). The final completeness is 99.70 % out to 74.564° in Θ. A Gaussian absorption correction was performed using CrysAlisPro 1.171.44.88a (Rigaku Oxford Diffraction, 2025) Numerical absorption correction based on Gaussian integration over a multifaceted crystal model. Empirical absorption correction using spherical harmonics as implemented in SCALE3 ABSPACK scaling algorithm. The absorption coefficient μ of this material is 0.448 mm^{-1} at this wavelength (λ = 1.54184Å) and the minimum and maximum transmissions are 0.752 and 1.000.

The structure was solved in the space group $R\bar{3}$ (# 148) by the ShelXT 2018/2 (Sheldrick, 2015) structure solution program using dual methods and refined by full matrix least squares minimisation on $\boldsymbol{F}^2$ using version 2019/3 of ShelXL 2019/3 (Sheldrick, 2015). All non-hydrogen atoms were refined anisotropically. Hydrogen atom positions were calculated geometrically and refined using the riding model.

There is a single formula unit in the asymmetric unit, which is represented by the reported sum formula. In other words: Z is 18 and Z' is 1. The moiety formula is C15 H18.

Citations

CrysAlis^{Pro} Software System, Rigaku Oxford Diffraction, (2025).

Sheldrick, G.M., ShelXT-Integrated space-group and crystal-structure determination, *Acta Cryst.*, (2015), **A71**, 3-8.

Sheldrick, G.M., Crystal structure refinement with ShelXL, *Acta Cryst.*, (2015), **C71**, 3-8.

O.V. Dolomanov and L.J. Bourhis and R.J. Gildea and J.A.K. Howard and H. Puschmann, **Olex2**: A complete structure solution, refinement and analysis program, *J. Appl. Cryst.*, (2009), **42**, 339-341.

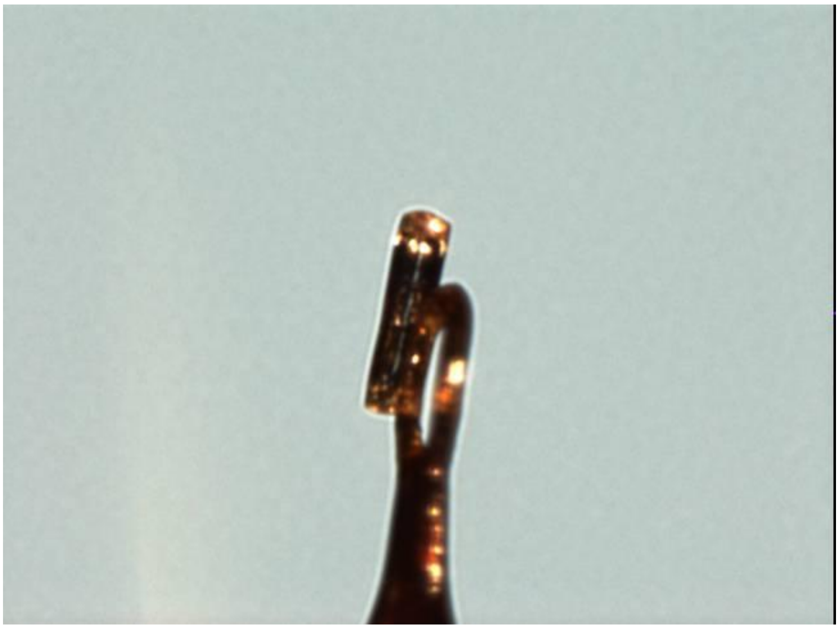

Figure 1 Image of the Crystal on the Diffractometer.

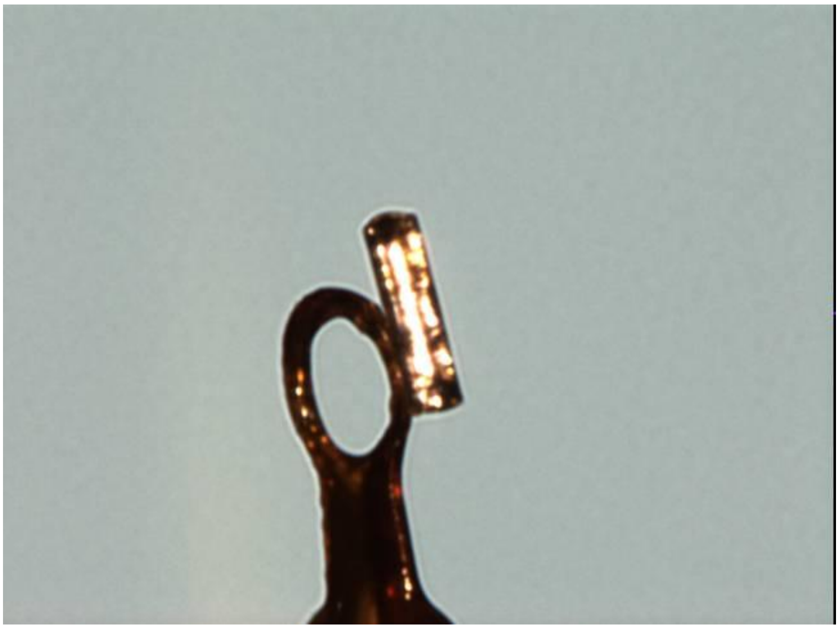

Figure 2 Image of the Crystal on the Diffractometer.

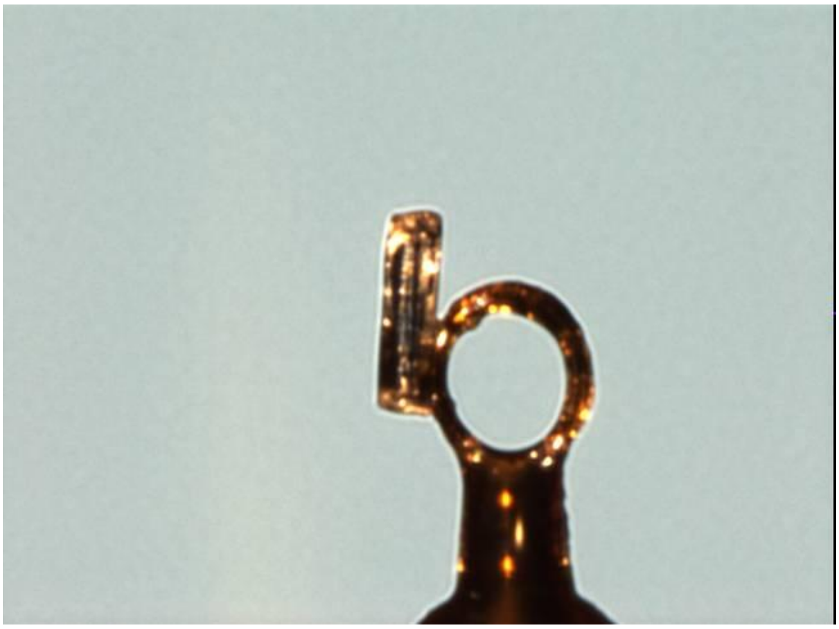

Figure 3 Image of the Crystal on the Diffractometer.

Data Plots: Diffraction Data

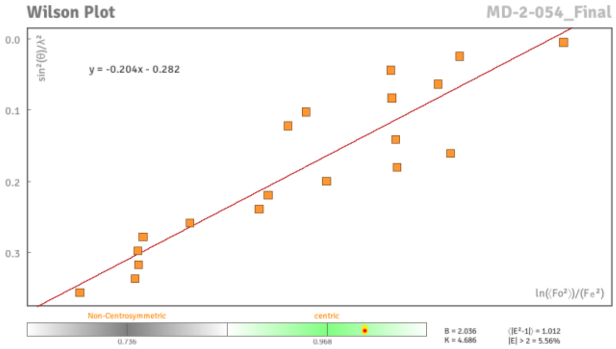

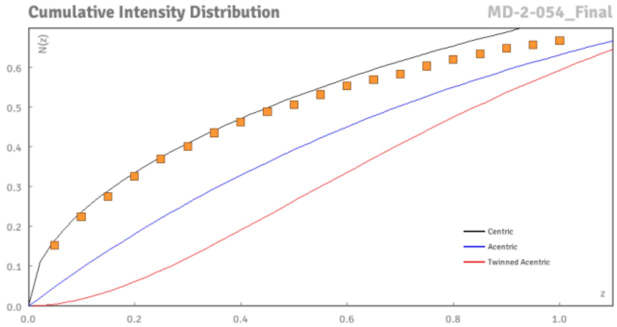

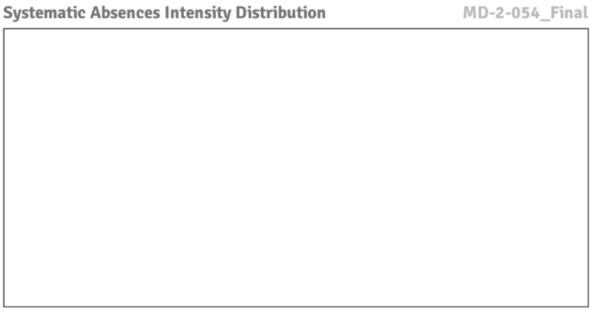

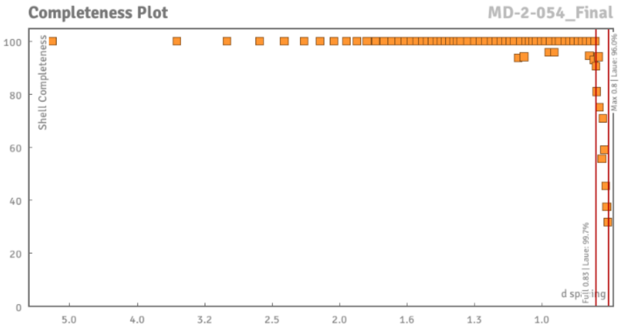

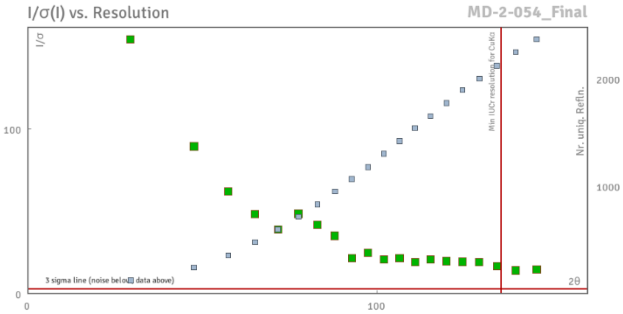

Data Plots: Refinement and Data

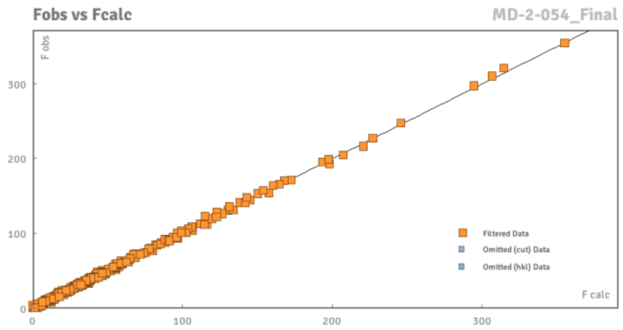

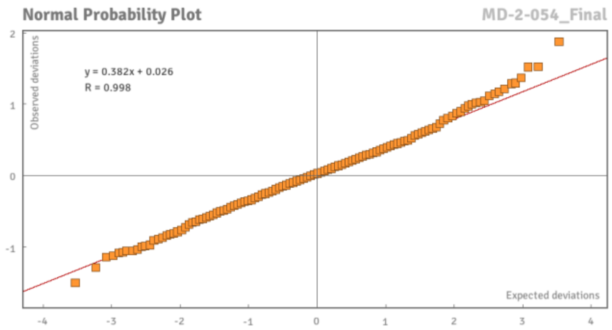

Reflection Statistics

Total reflections (after filtering)	8315	Unique reflections	2390
Completeness	0.96	Mean I/σ	38.53
hkl_{max} collected	(31, 28, 12)	hkl_{min} collected	(-29, -30, -9)
hkl_{max} used	(15, 31, 12)	hkl_{min} used	(-31, 0, 0)
Lim d_{max} collected	100.0	Lim d_{min} collected	0.77
d_{max} used	12.57	d_{min} used	0.8
Friedel pairs	448	Friedel pairs merged	1
Inconsistent equivalents	2	R_{int}	0.0134
R_{sigma}	0.0143	Intensity transformed	0
Omitted reflections	0	Omitted by user (OMIT hkl)	0
Multiplicity	(3157, 1287, 429, 139, 68, 25, 29, 6)	Maximum multiplicity	17
Removed systematic absences	0	Filtered off (Shel/OMIT)	0

Table S3: Fractional Atomic Coordinates (×10^4) and Equivalent Isotropic Displacement Parameters (Å^2×10^3) for (*Z*)-**11h**. U_{eq} is defined as 1/3 of the trace of the orthogonalised U_{ij}.

Atom	x	y	z	U_{eq}
C1	4389.7(4)	6139.8(4)	4173.0(10)	24.3(2)
C2	5090.6(4)	6464.8(4)	4298.4(10)	21.7(2)
C3	5385.2(5)	6549.3(5)	5524.7(10)	26.4(2)
C4	6024.0(5)	6875.7(5)	5617.1(11)	30.5(3)
C5	6380.4(5)	7125.8(5)	4485.9(11)	28.9(2)
C6	6095.5(5)	7045.4(5)	3256.7(10)	26.0(2)
C7	5459.5(5)	6717.8(4)	3165.4(10)	23.7(2)
C8	4136.3(4)	5616.4(4)	3111.1(10)	24.0(2)
C9	4179.3(5)	5056.2(5)	3456.4(10)	24.9(2)
C10	3689.6(5)	4533.2(5)	3879.0(10)	27.4(2)
C11	3085.0(5)	4444.2(5)	4051.5(10)	28.0(2)
C12	2574.1(5)	4349.9(5)	4188.6(12)	34.6(3)
C13	4786.7(5)	5088.8(5)	3252.9(12)	35.0(3)
C14	4063.5(5)	5873.8(5)	5504.0(11)	35.4(3)
C15	4218.9(5)	6617.3(5)	3692.7(14)	38.1(3)

Table S4: Anisotropic Displacement Parameters (×10^4) for (*Z*)-**11h**. The anisotropic displacement factor exponent takes the form: $-2\pi^2[h^2a^{*2} \times U_{11} + ... + 2hka^* \times b^* \times U_{12}]$

Atom	U_{11}	U_{22}	U_{33}	U_{23}	U_{13}	U_{12}
C1	21.2(5)	20.4(5)	29.2(5)	-1.6(4)	3.4(4)	8.8(4)
C2	23.0(5)	17.0(4)	25.2(5)	-1.4(4)	1.6(4)	10.1(4)

Atom	U_{11}	U_{22}	U_{33}	U_{23}	U_{13}	U_{12}
C3	30.4(5)	25.5(5)	23.2(5)	-0.8(4)	2.8(4)	13.8(4)
C4	32.1(6)	34.0(6)	26.5(5)	-4.8(4)	-6.5(4)	17.4(5)
C5	21.5(5)	29.1(5)	35.4(6)	-5.2(4)	-2.3(4)	12.1(4)
C6	24.2(5)	25.1(5)	28.4(5)	1.3(4)	4.9(4)	12.0(4)
C7	24.5(5)	23.6(5)	23.2(5)	0.4(4)	-0.1(4)	12.3(4)
C8	21.8(5)	24.0(5)	23.6(5)	0.3(4)	-0.9(4)	9.4(4)
C9	26.9(5)	25.5(5)	22.3(5)	-5.9(4)	-3.3(4)	13.0(4)
C10	31.9(5)	22.9(5)	27.7(5)	-2.6(4)	-2.5(4)	14.0(4)
C11	31.8(6)	19.9(5)	26.7(5)	-0.3(4)	-1.2(4)	8.6(4)
C12	29.0(6)	29.2(6)	40.5(6)	1.2(5)	0.9(5)	10.6(5)
C13	30.8(6)	31.4(6)	46.2(7)	-5.1(5)	0.0(5)	18.0(5)
C14	28.4(6)	31.4(6)	32.8(6)	-8.1(4)	9.3(4)	4.7(5)
C15	23.4(5)	26.8(6)	65.4(8)	-0.1(5)	2.0(5)	13.4(5)

Table S5: Bond Lengths in Å for (*Z*)-**11h**.

Atom	Atom	Length/Å
C1	C2	1.5321(13)
C1	C8	1.5566(13)
C1	C14	1.5290(14)
C1	C15	1.5395(15)
C2	C3	1.3914(14)
C2	C7	1.3982(13)
C3	C4	1.3938(15)
C4	C5	1.3823(15)

Atom	Atom	Length/Å
C5	C6	1.3841(15)
C6	C7	1.3877(14)
C8	C9	1.5050(14)
C9	C10	1.3429(15)
C9	C13	1.5012(14)
C10	C11	1.4315(15)
C11	C12	1.1916(16)

Table S6: Bond Angles in ° for (*Z*)-**11h**.

Atom	Atom	Atom	Angle/°
C2	C1	C8	112.44(8)
C2	C1	C15	107.72(8)
C14	C1	C2	112.60(9)
C14	C1	C8	108.62(8)
C14	C1	C15	108.31(9)
C15	C1	C8	106.91(9)
C3	C2	C1	122.39(9)
C3	C2	C7	117.49(9)
C7	C2	C1	120.04(9)
C2	C3	C4	121.11(9)

Atom	Atom	Atom	Angle/°
C5	C4	C3	120.50(10)
C4	C5	C6	119.21(9)
C5	C6	C7	120.23(9)
C6	C7	C2	121.46(9)
C9	C8	C1	116.37(8)
C10	C9	C8	121.90(9)
C10	C9	C13	120.35(10)
C13	C9	C8	117.69(9)
C9	C10	C11	124.93(10)
C12	C11	C10	177.84(11)

Table S7: Torsion Angles in ° for (*Z*)-**11h**.

Atom	Atom	Atom	Atom	Angle/˚
C1	C2	C3	C4	-176.50(9)
C1	C2	C7	C6	176.27(9)
C1	C8	C9	C10	102.60(11)
C1	C8	C9	C13	-80.17(11)
C2	C1	C8	C9	69.44(11)
C2	C3	C4	C5	0.26(16)
C3	C2	C7	C6	-0.48(14)
C3	C4	C5	C6	-0.38(16)
C4	C5	C6	C7	0.08(16)
C5	C6	C7	C2	0.37(15)
C7	C2	C3	C4	0.17(14)
C8	C1	C2	C3	-128.49(9)
C8	C1	C2	C7	54.91(12)
C8	C9	C10	C11	-0.09(16)
C13	C9	C10	C11	-177.25(10)
C14	C1	C2	C3	-5.39(13)
C14	C1	C2	C7	178.01(9)
C14	C1	C8	C9	-55.86(11)
C15	C1	C2	C3	113.97(11)
C15	C1	C2	C7	-62.63(12)
C15	C1	C8	C9	-172.54(8)

Table S8: Hydrogen Fractional Atomic Coordinates (×10[4]) and Equivalent Isotropic Displacement Parameters (Å^2×10^3) for (*Z*)-**11h**. U_{eq} is defined as 1/3 of the trace of the orthogonalised U_{ij}.

Atom	x	y	z	U_{eq}
H3	5146.91	6381.76	6312.27	32
H4	6216	6926.59	6463.79	37
H5	6815.75	7350.26	4551.28	35
H6	6336.18	7214.95	2472.95	31
H7	5270.51	6664.37	2314.21	28
H8A	3699.25	5484.8	2946.39	29
H8B	4360.61	5787.99	2261.81	29
H10	3748.88	4196.85	4078.55	33
H12	2166.73	4274.77	4297.91	42
H13A	4914.23	5191.4	2316.92	53
H13B	4747.12	4690.38	3470.69	53
H13C	5095.28	5405.87	3838.35	53
H14A	4186.69	5586.89	5852.9	53
H14B	3617.97	5656.16	5364.03	53
H14C	4176.89	6207.73	6148.04	53
H15A	4373.83	6957.75	4333.67	57
H15B	3771.12	6424.01	3626.58	57
H15C	4403.07	6774.1	2812.25	57

X-ray crystallographic data of **11q** (CCDC 2443047):

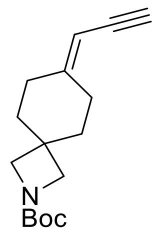

11q

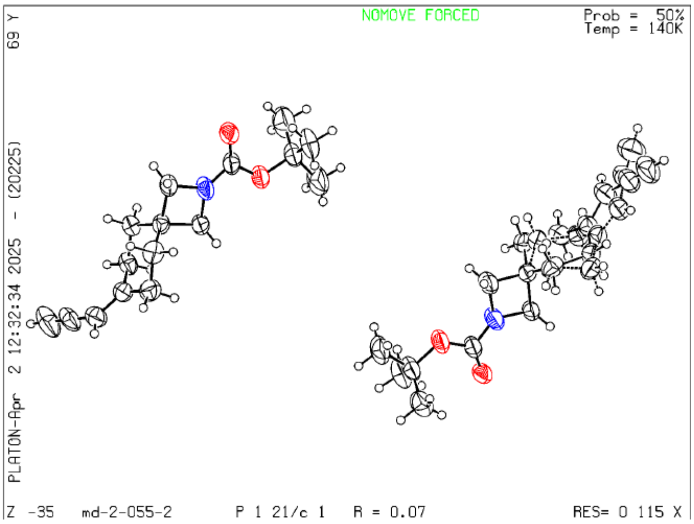
NOMOVE FORCED
Prob = 50%
Temp = 140K
PLATON-Apr 2 12:32:34 2025 - (20225)
69 Y
Z -35 md-2-055-2 P 1 21/c 1 R = 0.07 RES= 0 115 X

11q

Report created with ReportPlus

R_1=7.25%

Submitted by: **Morgane Elodie Marie Delattre**

Supervisor: **Professor Jieping Zhu**

Solved by: **Farzaneh Fadaei Tirani**

Sample ID: **11q**

Crystal Data and Experimental

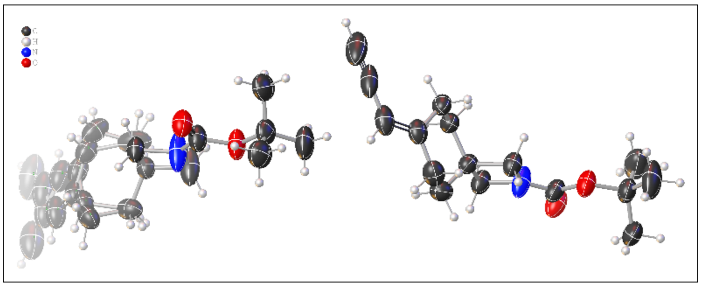

Ortep picture of 11q. The ellipsoids are displayed at 50% probability level.

Experimental. Single colourless irregular-shaped crystals of **11q** were used as supplied. A suitable crystal with dimensions 0.20 × 0.06 × 0.05 mm³ was selected and mounted on an XtaLAB Synergy R, DW system, HyPix-Arc 150 diffractometer. The crystal was kept at a steady T = 140.00(10) K during data collection. The structure was solved with the ShelXT 2018/2 (Sheldrick, 2015) solution program using dual methods and by using Olex2 1.5 (Dolomanov et al., 2009) as the graphical interface. The model was refined with ShelXL 2019/3 (Sheldrick, 2015) using full matrix least squares minimisation on $\boldsymbol{F}^2$.

Crystal Data. $C_{16}H_{23}NO_2$, M_r = 261.35, monoclinic, $P2_1/c$ (No. 14), a = 12.4256(3) Å, b = 23.6346(3) Å, c = 11.5821(4) Å, β = 112.255(3)°, α = γ = 90°, V = 3148.00(15) Å³, T = 140.00(10) K, Z = 8, Z' = 2, μ(Cu K_α) = 0.568, 27879 reflections measured, 6217 unique (R_{int} = 0.0281) which were used in all calculations. The final wR_2 was 0.2275 (all data) and R_1 was 0.0725 (I≥2σ(I)).

Compound	11q
Formula	$C_{16}H_{23}NO_2$
$D_{calc.}$/ g cm^{-3}	1.103
μ/mm^{-1}	0.568
Formula Weight	261.35
Colour	colourless
Shape	irregular-shaped
Size/mm³	0.20×0.06×0.05
T/K	140.00(10)
Crystal System	monoclinic
Space Group	$P2_1/c$
a/Å	12.4256(3)
b/Å	23.6346(3)
c/Å	11.5821(4)
α/°	90
β/°	112.255(3)
γ/°	90
V/Å³	3148.00(15)
Z	8
Z'	2
Wavelength/Å	1.54184
Radiation type	Cu$K\alpha$
Θ_{min}/°	3.740
Θ_{max}/°	74.844
Measured Refl's.	27879
Indep't Refl's	6217
Refl's I≥2σ(I)	3655
R_{int}	0.0281
Parameters	413
Restraints	319
Largest Peak/e Å^{-3}	0.462
Deepest Hole/e Å^{-3}	-0.365
GooF	1.038
wR_2 (all data)	0.2275
wR_2	0.1939
R_1 (all data)	0.1199
R_1	0.0725
CCDC number	2443047

Structure Quality Indicators

**Reflections:**	d min (CuKα) 2Θ=149.7° 0.80	I/σ(I) 32.9	Rint m=4.57 2.81%	Full 135.4° 96% to 149.7° 100
**Refinement:**	Shift 0.000	Max Peak 0.5	Min Peak -0.4	GooF 1.038

A colourless irregular-shaped crystal with dimensions 0.20 × 0.06 × 0.05 mm³ was mounted. Data were collected using an XtaLAB Synergy R, DW system, HyPix-Arc 150 diffractometer operating at T = 140.00(10) K.

Data were measured using ω scans with CuK_α radiation. The diffraction pattern was indexed and the total number of runs and images was based on the strategy calculation from the program CrysAlisPro system (CCD 44.89a 64-bit (release 14-01-2025)). The maximum resolution achieved was Θ = 74.844° (0.80 Å).

The unit cell was refined using CrysAlisPro 1.171.44.88a (Rigaku OD, 2025) on 7467 reflections, 27% of the observed reflections.

Data reduction, scaling and absorption corrections were performed using CrysAlisPro 1.171.44.88a (Rigaku OD, 2025). The final completeness is 100.00 % out to 74.844° in Θ. A Gaussian absorption correction was performed using CrysAlisPro 1.171.44.88a (Rigaku Oxford Diffraction, 2025) Numerical absorption correction based on Gaussian integration over a multifaceted crystal model. Empirical absorption correction using spherical harmonics as implemented in SCALE3 ABSPACK scaling algorithm. The absorption coefficient μ of this material is 0.568 mm^{-1} at this wavelength (λ = 1.54184Å) and the minimum and maximum transmissions are 0.702 and 1.000.

The structure was solved in the space group $P2_1/c$ (# 14) by the ShelXT 2018/2 (Sheldrick, 2015) structure solution program using dual methods and refined by full matrix least squares minimisation on $\boldsymbol{F^2}$ using version 2019/3 of ShelXL 2019/3 (Sheldrick, 2015). All non-hydrogen atoms were refined anisotropically. Hydrogen atom positions were calculated geometrically and refined using the riding model.

The value of Z' is 2. This means that there are two independent molecules in the asymmetric unit. The moiety formula is C16 H23 N O2.

Citations

CrysAlis^{Pro} Software System, Rigaku Oxford Diffraction, (2025).

Sheldrick, G.M., ShelXT-Integrated space-group and crystal-structure determination, *Acta Cryst.*, (2015), **A71**, 3-8.

Sheldrick, G.M., Crystal structure refinement with ShelXL, *Acta Cryst.*, (2015), **C71**, 3-8.

O.V. Dolomanov and L.J. Bourhis and R.J. Gildea and J.A.K. Howard and H. Puschmann, **Olex2**: A complete structure solution, refinement and analysis program, *J. Appl. Cryst.*, (2009), **42**, 339-341.

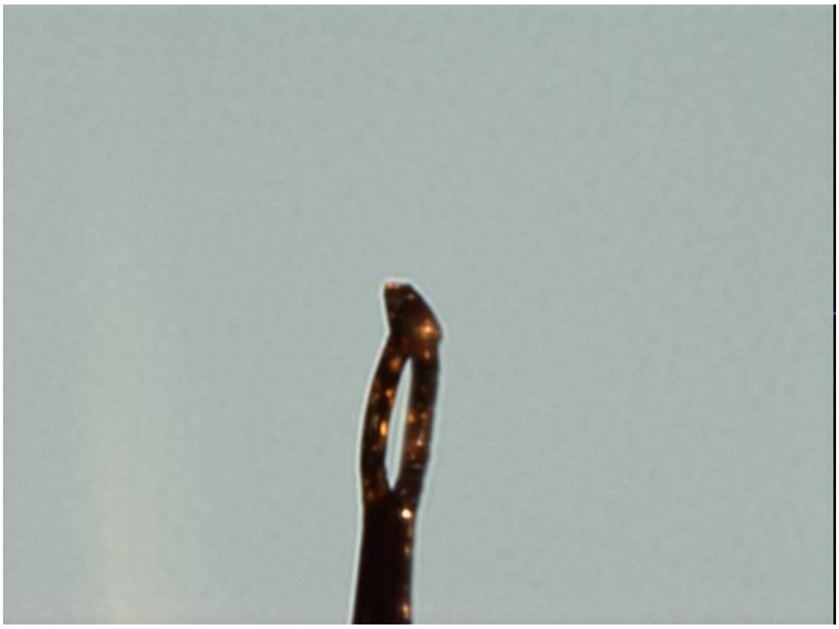

Figure 4 Image of the Crystal on the Diffractometer.

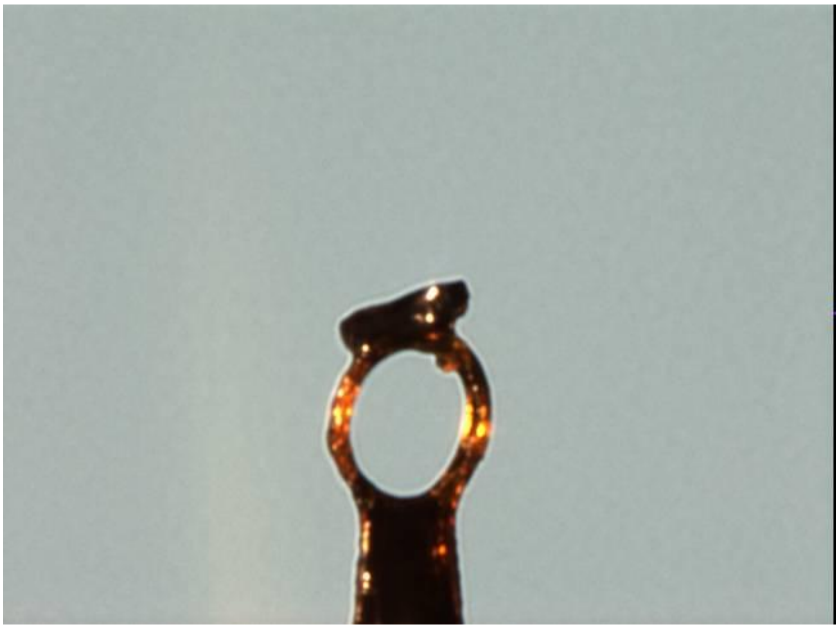

Figure 5 Image of the Crystal on the Diffractometer.

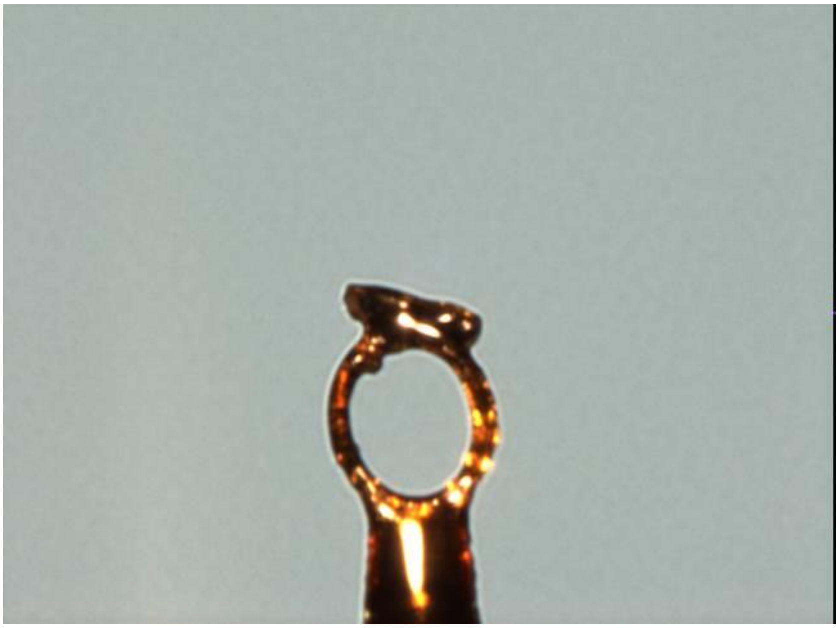

Figure 6 Image of the Crystal on the Diffractometer.

Data Plots: Diffraction Data

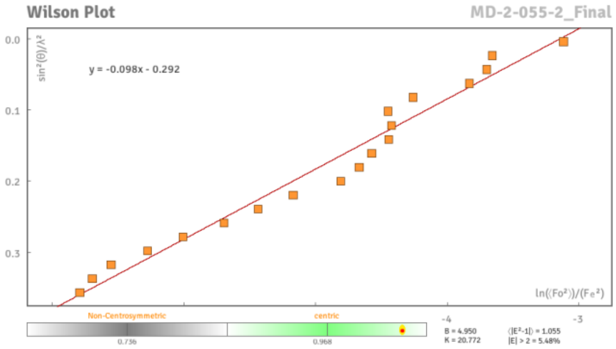

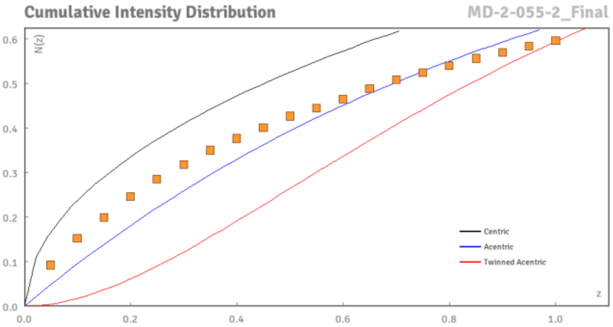

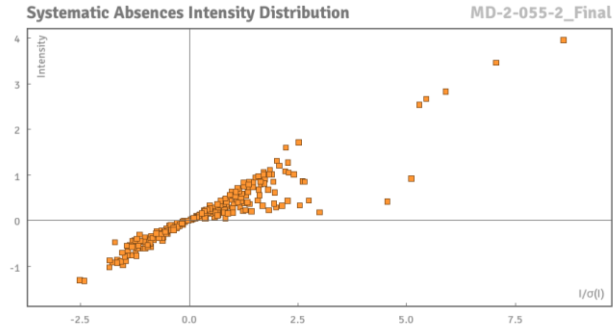

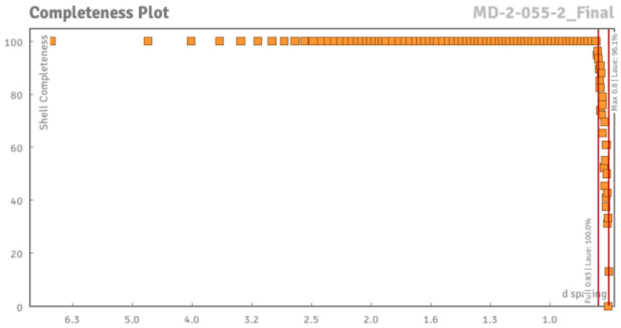

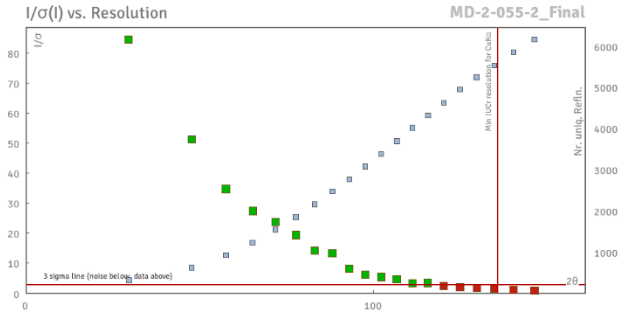

Data Plots: Refinement and Data

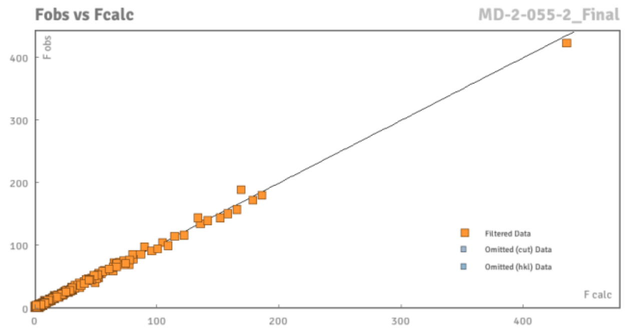

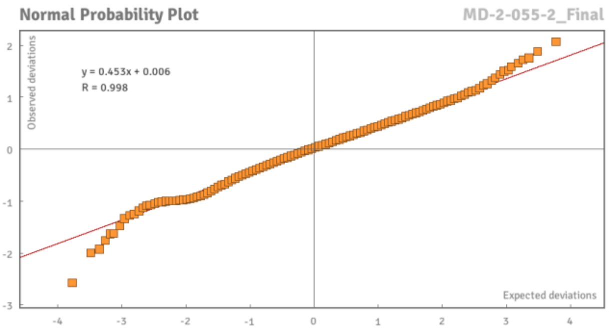

Reflection Statistics

Total reflections (after filtering)	28386	Unique reflections	6217
Completeness	0.961	Mean I/σ	16.1
hkl_{max} collected	(15, 29, 14)	hkl_{min} collected	(-15, -21, -14)
hkl_{max} used	(14, 29, 14)	hkl_{min} used	(-15, 0, 0)
Lim d_{max} collected	100.0	Lim d_{min} collected	0.77
d_{max} used	23.63	d_{min} used	0.8
Friedel pairs	4305	Friedel pairs merged	1
Inconsistent equivalents	0	R_{int}	0.0281
R_{sigma}	0.0304	Intensity transformed	0
Omitted reflections	0	Omitted by user (OMIT hkl)	0
Multiplicity	(8181, 4689, 1719, 462, 237, 150, 77, 65, 45, 13, 13)	Maximum multiplicity	19
Removed systematic absences	507	Filtered off (Shel/OMIT)	0

Table S9: Fractional Atomic Coordinates (×10^4) and Equivalent Isotropic Displacement Parameters (Å^2×10^3) for **11q**. U_{eq} is defined as 1/3 of the trace of the orthogonalised U_{ij}.

Atom	x	y	z	U_{eq}
O1	9498.2(17)	3303.8(8)	12107(2)	74.4(6)
O2	8117.4(16)	3832.3(8)	10626.7(19)	67.6(6)
N1	8067(2)	2905.9(10)	10490(3)	78.9(9)
C1	8639(2)	3347.7(11)	11164(3)	59.6(8)
C2	8255(2)	2306.3(11)	10745(3)	60.7(8)
C3	7139(2)	2194.6(11)	9583(2)	49.4(6)
C4	6178(2)	1904.4(11)	9869(3)	52.5(7)
C5	5054(2)	1865.7(11)	8708(3)	57.0(7)
C6	5257(2)	1591.9(11)	7652(3)	58.3(7)
C7	6229(3)	1842.9(13)	7375(3)	70.1(8)
C8	7340(3)	1880.5(13)	8534(3)	63.9(8)
C9	6997(2)	2845.0(11)	9399(3)	58.2(7)
C10	4620(3)	1154.4(12)	6996(3)	73.9(10)
C11	3675(3)	913.1(13)	7226(4)	86.2(12)
C12	2888(3)	711.2(16)	7404(5)	111.2(17)
C13	8567(3)	4382.3(11)	11186(3)	66.3(8)
C14	8507(4)	4427.2(16)	12453(4)	112.4(15)
C15	9798(3)	4463.1(13)	11265(4)	87.7(11)
C16	7754(4)	4795.0(15)	10295(4)	120.7(18)
O3	5397.1(16)	6813.6(8)	2841.5(19)	67.1(6)
O4	6538.6(18)	6097.8(8)	3988(2)	76.0(7)
N2	7064(2)	6975.5(10)	4453(3)	94.6(10)
C17	6247(3)	6642.7(11)	3685(3)	66.7(8)
C18	7108(2)	7588.9(11)	4564(3)	58.8(7)

Atom	x	y	z	U_{eq}
C19	8242(2)	7517.2(10)	5743(2)	50.5(6)
C20	9324(2)	7730.6(13)	5599(3)	65.2(7)
C21A	9360(6)	8409(2)	5893(7)	64.3(13)
C21B	9448(6)	8309(3)	5340(6)	66.8(15)
C22A	9076(6)	8567(3)	7008(6)	65.9(12)
C22B	9458(6)	8592(3)	6506(7)	66.9(13)
C23A	7996(5)	8288(3)	7036(6)	66.7(13)
C23B	8419(7)	8482(2)	6830(7)	64.6(13)
C24A	8092(7)	7652(3)	6920(6)	56.9(14)
C24B	8242(8)	7850(2)	6904(6)	57.1(13)
C25A	9771(5)	8884(3)	7966(7)	83.8(14)
C25B	10374(6)	8899(3)	7222(6)	84.2(14)
C26A	10856(6)	9116(4)	8103(9)	87.5(18)
C26B	10411(9)	9151(4)	8353(8)	104(2)
C27A	11731(7)	9306(3)	8239(8)	119(3)
C27B	10543(9)	9387(4)	9285(8)	140(3)
C28	8081(3)	6875.3(13)	5593(3)	91.8(12)
C29	5774(3)	5637.6(12)	3295(3)	71.0(8)
C30	4605(3)	5688.4(14)	3405(4)	92.8(12)
C31	6422(4)	5120.7(14)	3994(4)	99.6(13)
C32	5676(3)	5634.9(15)	1962(3)	87.0(10)

Table S10: Anisotropic Displacement Parameters (×10[4]) for **11q**. The anisotropic displacement factor exponent takes the form: $-2\pi^2[h^2a^{*2} \times U_{11}+ ... +2hka^* \times b^* \times U_{12}]$

Atom	U_{11}	U_{22}	U_{33}	U_{23}	U_{13}	U_{12}
O1	60.8(12)	56.4(12)	79.7(14)	9.6(10)	-3.4(11)	-12.0(9)
O2	58.4(12)	42.9(10)	82.4(14)	5.7(9)	5.1(10)	-5.2(8)
N1	59.6(15)	40.1(13)	97(2)	11.8(12)	-15.5(13)	-5.2(10)
C1	47.7(15)	44.9(15)	76(2)	10.3(13)	11.6(14)	-5.1(12)
C2	48.4(15)	44.9(15)	75.2(19)	8.9(13)	8.0(13)	-2.6(11)
C3	42.5(13)	45.1(14)	57.4(15)	6.2(12)	15.5(12)	-1.7(11)
C4	51.9(15)	47.3(14)	60.1(16)	-4.5(12)	23.1(13)	-5.5(11)
C5	45.1(14)	45.9(14)	76.0(19)	-4.1(13)	18.2(13)	-3.6(11)
C6	57.7(16)	44.5(14)	59.1(17)	1.2(12)	6.9(13)	9.0(12)
C7	86(2)	60.8(18)	62.5(19)	0.8(15)	27.6(17)	5.1(16)
C8	59.4(17)	65.5(18)	73(2)	3.0(15)	32.1(15)	3.4(14)
C9	50.2(14)	44.2(14)	65.3(17)	5.5(12)	4.9(13)	-4.1(11)
C10	75(2)	47.3(16)	73(2)	-4.3(14)	-1.5(16)	11.1(15)
C11	68(2)	40.6(16)	106(3)	-11.2(17)	-17.6(19)	4.8(16)
C12	61(2)	59(2)	173(4)	-15(2)	-2(2)	-4.1(17)
C13	66.4(18)	42.3(15)	85(2)	-2.3(14)	23.1(16)	-8.6(13)
C14	163(4)	72(2)	129(4)	-18(2)	86(3)	-24(3)
C15	81(2)	52.3(18)	139(3)	-11.2(19)	52(2)	-21.3(16)
C16	113(3)	45(2)	158(4)	5(2)	0(3)	7.0(19)
O3	57.4(11)	49.7(10)	70.3(13)	-1.8(9)	-2.9(10)	-2.9(9)
O4	71.7(12)	38.2(10)	90.2(14)	0.8(9)	-0.8(11)	-5.9(9)
N2	85.1(16)	40.7(12)	97.2(17)	6.0(12)	-34.2(14)	-6.9(11)
C17	63.5(15)	39.6(13)	75.8(17)	1.2(13)	2.2(14)	-6.9(12)
C18	54.9(14)	38.2(12)	63.2(16)	1.9(11)	-0.2(12)	-2.3(11)
C19	47.4(12)	44.7(12)	50.8(12)	5.0(10)	8.6(10)	-1.4(10)
C20	53.2(14)	77.5(17)	62.0(16)	15.8(14)	18.7(12)	5.8(13)
C21A	58.2(19)	57(2)	67(2)	13(2)	11(2)	-6.5(18)
C21B	58(2)	72(3)	65(3)	21(2)	17(2)	-5(2)
C22A	62(2)	49.9(18)	70(2)	-5.0(18)	6.5(19)	3.1(19)
C22B	64(2)	49(2)	69(2)	9(2)	6(2)	-2.9(19)
C23A	63(2)	64(3)	65(2)	-12(2)	15(2)	-1(2)
C23B	68(2)	51(2)	65(2)	-3.1(19)	14(2)	-2(2)
C24A	55(2)	62(3)	53(2)	5(2)	20.1(19)	-7(2)

Atom	U_{11}	U_{22}	U_{33}	U_{23}	U_{13}	U_{12}
C24B	59(2)	53(2)	55(2)	6(2)	18.3(18)	-4(2)
C25A	76(2)	56(2)	86(2)	-9(2)	-7(2)	1(2)
C25B	78(2)	54(2)	85(2)	7(2)	-9(2)	-8(2)
C26A	77(3)	53(2)	96(3)	-3(2)	-8(3)	-4(3)
C26B	101(3)	59(3)	100(3)	-2(3)	-22(3)	-4(3)
C27A	91(4)	71(4)	137(5)	3(4)	-23(4)	-9(3)
C27B	161(5)	78(4)	107(5)	-12(4)	-31(4)	14(4)
C28	77.9(19)	50.0(16)	93(2)	9.4(15)	-29.6(17)	-7.4(14)
C29	70.4(17)	41.7(14)	93(2)	-11.5(14)	21.7(16)	-11.6(13)
C30	97(3)	56.7(19)	132(3)	-19(2)	52(2)	-19.6(18)
C31	111(3)	43.2(17)	126(3)	0.7(18)	25(3)	-5.3(17)
C32	101(3)	65(2)	96(3)	-19.3(18)	39(2)	-11.3(18)

Table S11: Bond Lengths in Å for **11q**.

Atom	Atom	Length/Å
O1	C1	1.209(3)
O2	C1	1.346(3)
O2	C13	1.466(3)
N1	C1	1.336(4)
N1	C2	1.449(3)
N1	C9	1.453(3)
C2	C3	1.546(4)
C3	C4	1.518(3)
C3	C8	1.523(4)
C3	C9	1.553(4)
C4	C5	1.532(4)
C5	C6	1.487(4)
C6	C7	1.486(4)
C6	C10	1.348(4)
C7	C8	1.521(4)
C10	C11	1.418(6)
C11	C12	1.175(6)
C13	C14	1.501(5)
C13	C15	1.509(4)
C13	C16	1.499(5)
O3	C17	1.205(3)
O4	C17	1.348(3)
O4	C29	1.467(3)
N2	C17	1.325(4)
N2	C18	1.455(3)
N2	C28	1.461(4)
C18	C19	1.556(3)
C19	C20	1.503(3)
C19	C24A	1.479(6)
C19	C24B	1.558(6)
C19	C28	1.532(4)
C20	C21A	1.636(6)
C20	C21B	1.421(6)
C21A	C22A	1.509(7)
C21B	C22B	1.504(7)
C22A	C23A	1.507(7)
C22A	C25A	1.345(7)
C22B	C23B	1.497(7)
C22B	C25B	1.340(7)
C23A	C24A	1.519(7)
C23B	C24B	1.517(6)
C25A	C26A	1.407(8)
C25B	C26B	1.424(8)
C26A	C27A	1.132(7)
C26B	C27B	1.170(8)
C29	C30	1.510(5)
C29	C31	1.517(5)
C29	C32	1.502(5)

Table S12: Bond Angles in ° for **11q**.

Atom	Atom	Atom	Angle/°
C1	O2	C13	120.9(2)
C1	N1	C2	129.5(2)
C1	N1	C9	134.0(2)
C2	N1	C9	96.0(2)
O1	C1	O2	126.6(3)
O1	C1	N1	123.6(3)
N1	C1	O2	109.8(2)
N1	C2	C3	88.10(19)
C2	C3	C9	88.20(19)
C4	C3	C2	114.1(2)
C4	C3	C8	109.5(2)
C4	C3	C9	114.8(2)
C8	C3	C2	114.4(2)
C8	C3	C9	114.7(2)
C3	C4	C5	111.5(2)
C6	C5	C4	111.4(2)
C7	C6	C5	114.1(2)
C10	C6	C5	123.4(3)
C10	C6	C7	122.4(3)
C6	C7	C8	111.9(2)
C7	C8	C3	111.4(2)
N1	C9	C3	87.68(19)
C6	C10	C11	123.6(3)
C12	C11	C10	179.3(4)

Atom	Atom	Atom	Angle/°
O2	C13	C14	110.1(3)
O2	C13	C15	110.3(2)
O2	C13	C16	103.2(3)
C14	C13	C15	110.9(3)
C16	C13	C14	110.9(3)
C16	C13	C15	111.2(3)
C17	O4	C29	120.8(2)
C17	N2	C18	130.1(2)
C17	N2	C28	133.7(3)
C18	N2	C28	95.2(2)
O3	C17	O4	126.8(3)
O3	C17	N2	123.9(3)
N2	C17	O4	109.3(2)
N2	C18	C19	87.73(19)
C18	C19	C24B	114.0(4)
C20	C19	C18	114.7(2)
C20	C19	C24B	102.6(3)
C20	C19	C28	113.8(3)
C24A	C19	C18	113.4(4)
C24A	C19	C20	117.4(3)
C24A	C19	C28	105.1(3)
C28	C19	C18	88.44(19)
C28	C19	C24B	123.7(3)
C19	C20	C21A	105.2(3)
C21B	C20	C19	121.3(4)
C22A	C21A	C20	115.1(4)
C20	C21B	C22B	101.7(5)
C23A	C22A	C21A	114.2(5)
C25A	C22A	C21A	124.0(7)
C25A	C22A	C23A	121.6(7)
C23B	C22B	C21B	115.7(5)
C25B	C22B	C21B	120.8(7)
C25B	C22B	C23B	123.4(8)
C22A	C23A	C24A	109.3(5)
C22B	C23B	C24B	110.0(5)
C19	C24A	C23A	109.6(5)
C23B	C24B	C19	113.7(5)
C22A	C25A	C26A	126.7(8)
C22B	C25B	C26B	121.3(9)
C27A	C26A	C25A	178.5(11)
C27B	C26B	C25B	172.9(13)
N2	C28	C19	88.4(2)
O4	C29	C30	109.8(3)
O4	C29	C31	101.7(3)
O4	C29	C32	110.4(3)
C30	C29	C31	110.6(3)
C32	C29	C30	112.4(3)
C32	C29	C31	111.4(3)

Table S13: Torsion Angles in ° for **11q.**

Atom	Atom	Atom	Atom	Angle/°
N1	C2	C3	C4	-117.4(2)
N1	C2	C3	C8	115.4(3)
N1	C2	C3	C9	-0.9(2)
C1	O2	C13	C14	-62.3(4)
C1	O2	C13	C15	60.3(4)
C1	O2	C13	C16	179.3(3)
C1	N1	C2	C3	173.8(4)
C1	N1	C9	C3	-173.3(4)
C2	N1	C1	O1	5.1(6)
C2	N1	C1	O2	-175.3(3)
C2	N1	C9	C3	-1.0(3)
C2	C3	C4	C5	173.6(2)
C2	C3	C8	C7	-173.8(2)
C2	C3	C9	N1	0.9(2)
C3	C4	C5	C6	54.0(3)
C4	C3	C8	C7	56.7(3)
C4	C3	C9	N1	116.7(3)
C4	C5	C6	C7	-51.4(3)
C4	C5	C6	C10	128.6(3)
C5	C6	C7	C8	51.8(3)
C5	C6	C10	C11	1.1(4)
C6	C7	C8	C3	-54.1(3)
C7	C6	C10	C11	-178.9(3)
C8	C3	C4	C5	-56.7(3)
C8	C3	C9	N1	-115.2(3)
C9	N1	C1	O1	175.1(3)
C9	N1	C1	O2	-5.3(5)
C9	N1	C2	C3	1.0(3)
C9	C3	C4	C5	73.9(3)
C9	C3	C8	C7	-74.0(3)
C10	C6	C7	C8	-128.3(3)

Atom	Atom	Atom	Atom	Angle/°
C13	O2	C1	O1	-0.9(5)
C13	O2	C1	N1	179.5(3)
N2	C18	C19	C20	112.4(3)
N2	C18	C19	C24A	-109.0(4)
N2	C18	C19	C24B	-129.8(3)
N2	C18	C19	C28	-3.2(3)
C17	O4	C29	C30	-60.4(4)
C17	O4	C29	C31	-177.6(3)
C17	O4	C29	C32	64.1(4)
C17	N2	C18	C19	173.3(4)
C17	N2	C28	C19	-172.8(4)
C18	N2	C17	O3	6.2(7)
C18	N2	C17	O4	-175.0(3)
C18	N2	C28	C19	-3.4(3)
C18	C19	C20	C21A	84.2(4)
C18	C19	C20	C21B	61.4(5)
C18	C19	C24A	C23A	-73.9(5)
C18	C19	C24B	C23B	-74.9(7)
C18	C19	C28	N2	3.2(3)
C19	C20	C21A	C22A	43.8(6)
C19	C20	C21B	C22B	65.1(6)
C20	C19	C24A	C23A	63.5(6)
C20	C19	C24B	C23B	49.7(7)
C20	C19	C28	N2	-113.2(3)
C20	C21A	C22A	C23A	-48.0(7)
C20	C21A	C22A	C25A	125.7(7)
C20	C21B	C22B	C23B	-57.0(7)
C20	C21B	C22B	C25B	120.1(6)
C21A	C22A	C23A	C24A	53.8(7)
C21A	C22A	C25A	C26A	-0.2(11)
C21B	C22B	C23B	C24B	54.8(8)
C21B	C22B	C25B	C26B	-177.6(7)
C22A	C23A	C24A	C19	-58.7(7)
C22B	C23B	C24B	C19	-50.6(8)
C23A	C22A	C25A	C26A	173.0(7)
C23B	C22B	C25B	C26B	-0.8(11)
C24A	C19	C20	C21A	-52.7(5)
C24A	C19	C28	N2	117.0(4)
C24B	C19	C20	C21B	-62.8(5)
C24B	C19	C28	N2	121.2(5)
C25A	C22A	C23A	C24A	-120.0(7)
C25B	C22B	C23B	C24B	-122.1(7)
C28	N2	C17	O3	172.3(4)
C28	N2	C17	O4	-8.8(6)
C28	N2	C18	C19	3.3(3)
C28	C19	C20	C21A	-176.0(3)
C28	C19	C20	C21B	161.1(4)
C28	C19	C24A	C23A	-168.9(4)
C28	C19	C24B	C23B	180.0(5)
C29	O4	C17	O3	-2.1(5)
C29	O4	C17	N2	179.1(3)

Table S14: Hydrogen Fractional Atomic Coordinates (×10[4]) and Equivalent Isotropic Displacement Parameters (Å[2]×10[3]) for **11q**. U_{eq} is defined as 1/3 of the trace of the orthogonalised U_{ij}.

Atom	x	y	z	U_{eq}
H2A	8201.13	2186.76	11540.57	73
H2B	8973.25	2159.73	10671.89	73
H4A	6026.78	2118.35	10526.9	63

Atom	x	y	z	U_{eq}
H4B	6433.19	1518.77	10189.82	63
H5A	4469.8	1644.77	8908	68
H5B	4737.32	2250.66	8457.24	68
H7A	6005.13	2226.73	7023.77	84
H7B	6374.05	1609.22	6739.27	84
H8A	7944.63	2079.47	8326.44	77
H8B	7626.67	1494.11	8818.56	77
H9A	7051.07	2982.4	8615.12	70
H9B	6301.3	2999.76	9508.31	70
H10	4814.43	999.24	6343.02	89
H12	2251.24	547.91	7547.59	133
H14A	9043.91	4153.98	13018.32	169
H14B	7712.62	4346.24	12388.11	169
H14C	8724.74	4810.84	12778.77	169
H15A	9820.58	4419.83	10432.75	132
H15B	10303.78	4179.36	11828.33	132
H15C	10068.19	4842.72	11584.1	132
H16A	6957.79	4720.63	10230.34	181
H16B	7797.2	4752.94	9471.32	181
H16C	7976.8	5181.24	10599.52	181
H18A	6452.36	7755.15	4736.69	71
H18B	7220.84	7785.54	3864.01	71
H20A	9286.74	7663.12	4740.99	78
H20B	10021.85	7539.49	6195.54	78
H20C	9986.11	7635.09	6377.89	78
H20D	9423.29	7506.39	4924.06	78
H21A	10147.22	8552.69	6027.45	77
H21B	8803.72	8602.84	5148.51	77
H21C	8786.42	8440.57	4594.56	80
H21D	10183.27	8376.36	5215.74	80
H23A	7303.53	8431.19	6339.51	80
H23B	7906.37	8379.85	7829.32	80
H23C	7719.04	8651.39	6186.64	78
H23D	8532.91	8659.99	7641.35	78
H24A	8765.16	7506.81	7637.21	68
H24B	7380.7	7465.97	6928.3	68
H24C	7492.94	7784.43	6997.98	68
H24D	8868.31	7699.2	7657.7	68
H25A	9501.52	8957.98	8616.7	101
H25B	11011.93	8949.78	6970.32	101
H27A	12465.99	9466.04	8352.57	143
H27B	10651.1	9579.17	10041.68	168
H28A	8724.65	6678.27	5454.4	110
H28B	7898.8	6691.2	6265.44	110
H30A	4193.36	6020.04	2935.96	139
H30B	4716.81	5730.81	4284.73	139
H30C	4145.46	5347.27	3065.27	139
H31A	6489.71	5139.31	4863.54	149
H31B	7200.83	5110.75	3966.21	149
H31C	5995.33	4778.13	3602.01	149
H32A	6456.39	5634.57	1938.27	131
H32B	5253.88	5972.71	1536.1	131
H32C	5255.31	5295.6	1542.54	131

Table S15: Atomic Occupancies for all atoms that are not fully occupied in **11q**.

Atom	Occupancy	Atom	Occupancy	Atom	Occupancy
H20A	0.511(3)	H20C	0.489(3)	C21A	0.511(3)
H20B	0.511(3)	H20D	0.489(3)	H21A	0.511(3)

Atom	Occupancy
H21B	0.511(3)
C21B	0.489(3)
H21C	0.489(3)
H21D	0.489(3)
C22A	0.511(3)
C22B	0.489(3)
C23A	0.511(3)
H23A	0.511(3)
H23B	0.511(3)
C23B	0.489(3)

Atom	Occupancy
H23C	0.489(3)
H23D	0.489(3)
C24A	0.511(3)
H24A	0.511(3)
H24B	0.511(3)
C24B	0.489(3)
H24C	0.489(3)
H24D	0.489(3)
C25A	0.511(3)
H25A	0.511(3)

Atom	Occupancy
C25B	0.489(3)
H25B	0.489(3)
C26A	0.511(3)
C26B	0.489(3)
C27A	0.511(3)
H27A	0.511(3)
C27B	0.489(3)
H27B	0.489(3)

10) Copies of the NMR spectra

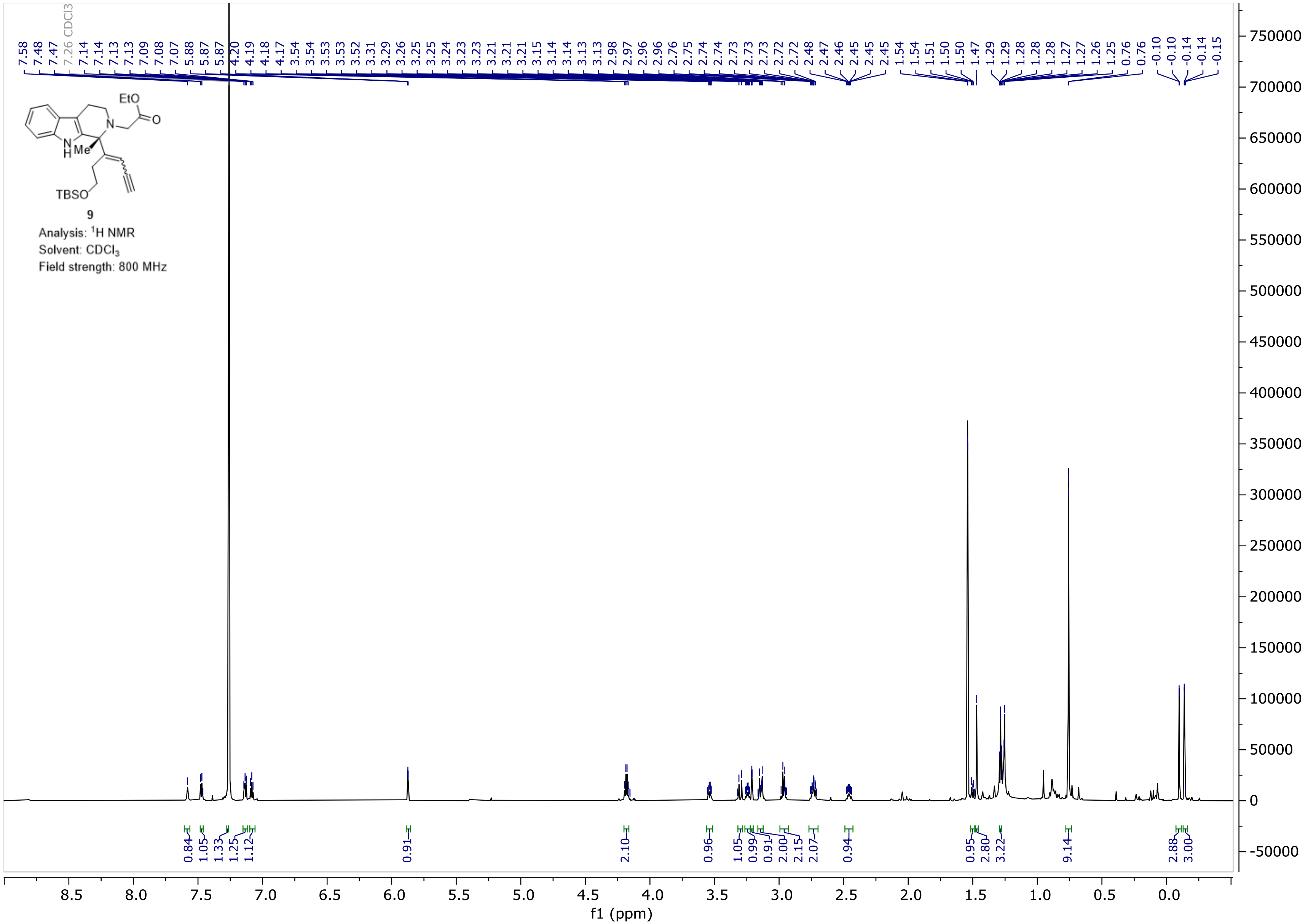
Analysis: ¹H NMR
Solvent: CDCl3
Field strength: 800 MHz
f1 (ppm)

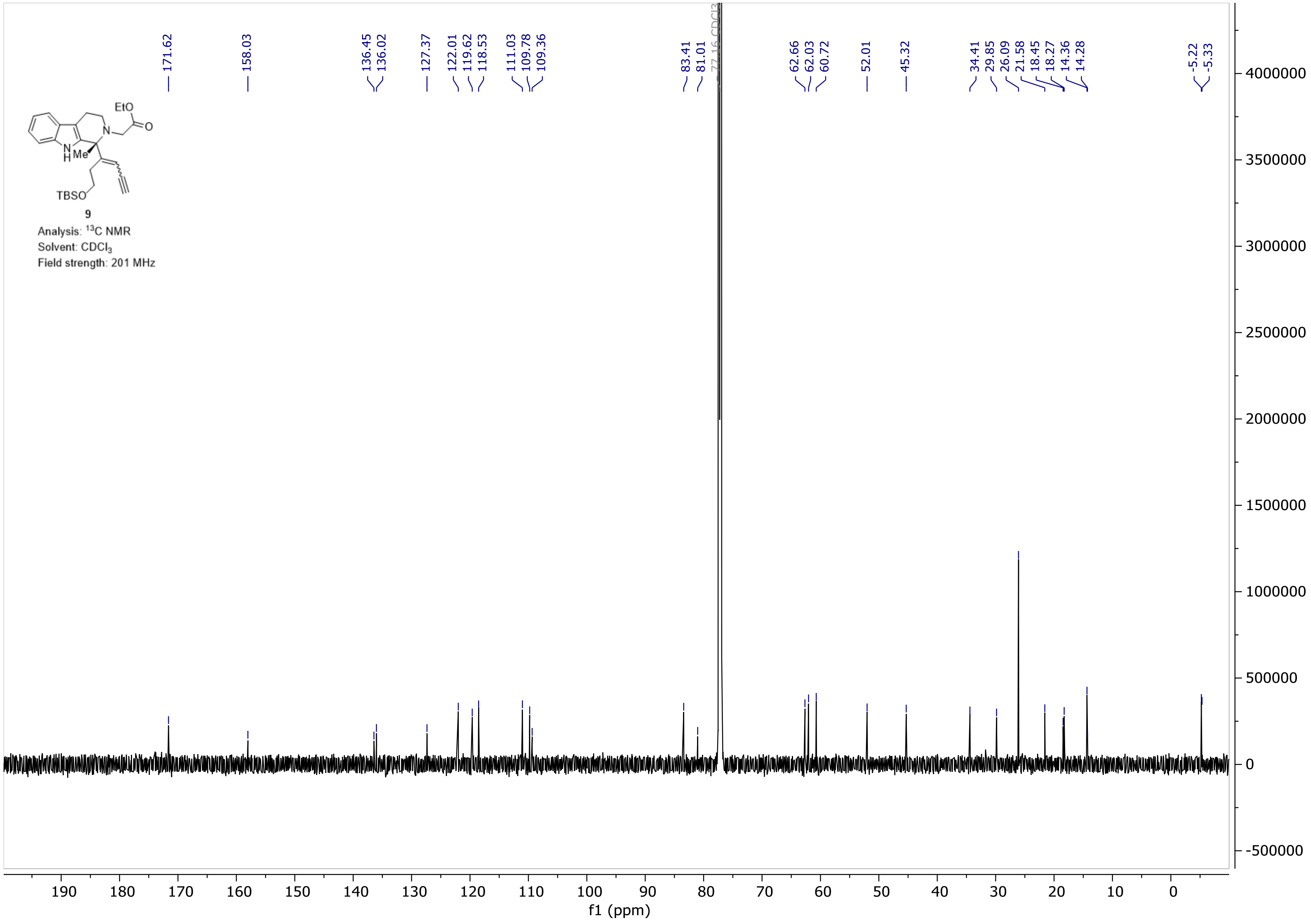

171.62
158.03
136.45
136.02
127.37
122.01
119.62
118.53
111.03
109.78
109.36
83.41
81.01
77.16 CDCl3
62.66
62.03
60.72
52.01
45.32
34.41
29.85
26.09
21.58
18.45
18.27
14.36
14.28
-5.22
-5.33
EtO
O
N
N
H
Me
TBSO
9
Analysis: 13C NMR
Solvent: CDCl3
Field strength: 201 MHz
4000000
3500000
3000000
2500000
2000000
1500000
1000000
500000
0
-500000
190
180
170
160
150
140
130
120
110
100
90
80
70
60
50
40
30
20
10
0
f1 (ppm)

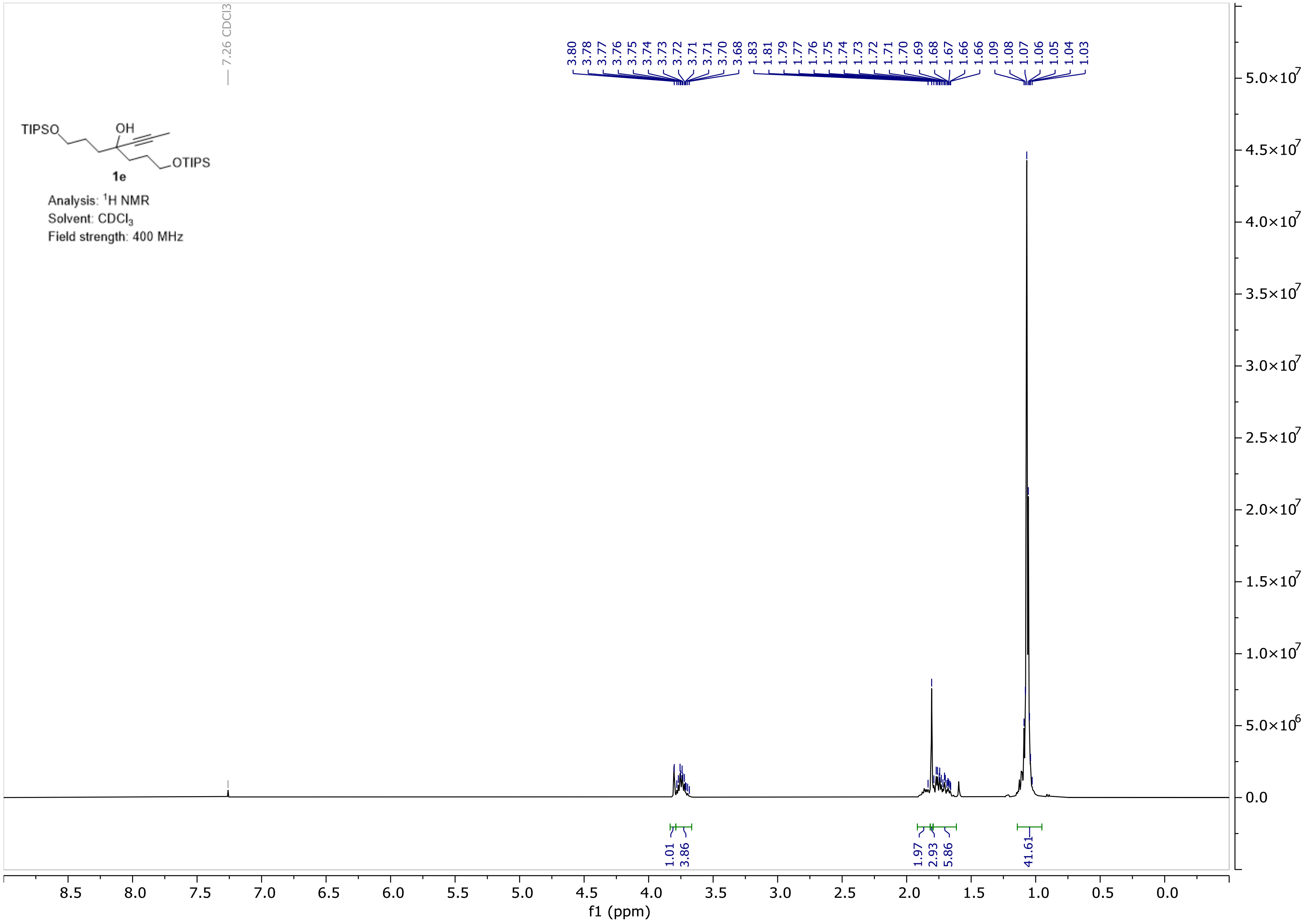

TIPSO
OH
OTIPS
1e
Analysis: ^{1}H NMR
Solvent: $CDCl_3$
Field strength: 400 MHz
7.26 CDCl3
3.80 3.78 3.77 3.76 3.75 3.74 3.73 3.72 3.71 3.71 3.70 3.68 1.83 1.81 1.79 1.77 1.76 1.75 1.74 1.73 1.72 1.71 1.70 1.69 1.68 1.67 1.66 1.66 1.09 1.08 1.07 1.06 1.05 1.04 1.03
1.01
3.86
1.97
2.93
5.86
41.61
f1 (ppm)

1e

Analysis: ^{13}C NMR
Solvent: $CDCl_3$
Field strength: 101 MHz

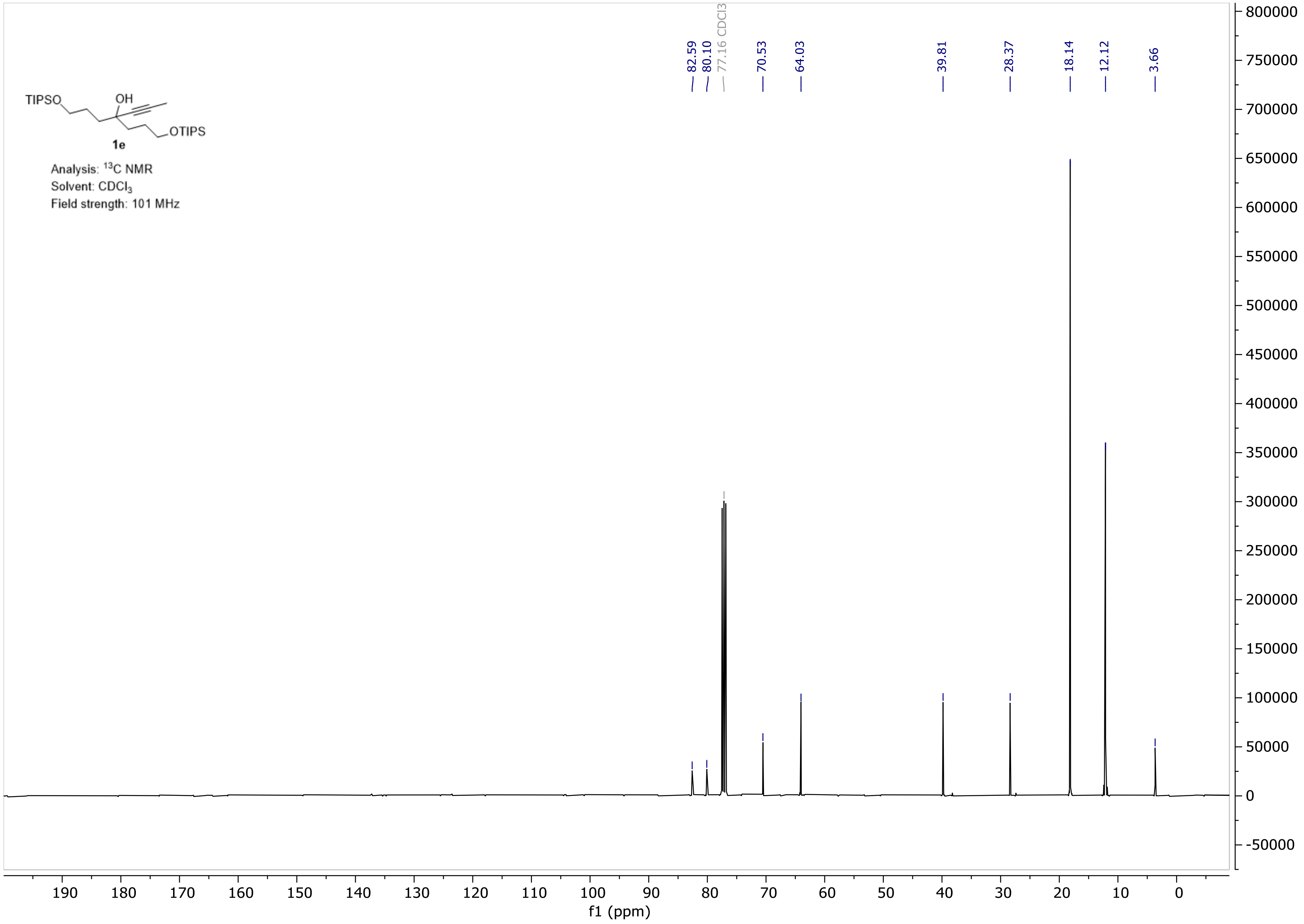

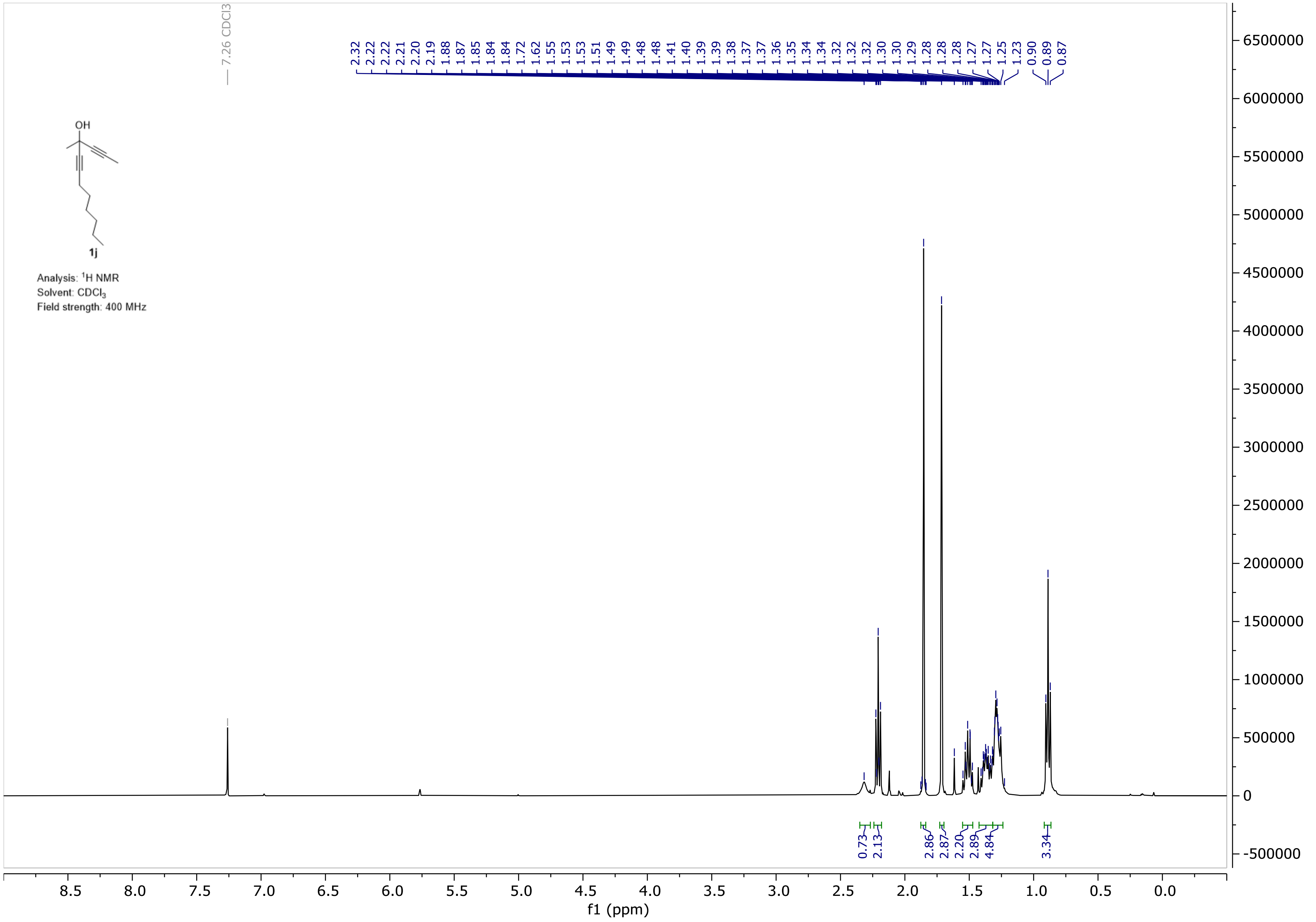
OH
1j
Analysis: ^{1}H NMR
Solvent: $CDCl_3$
Field strength: 400 MHz
7.26 CDCl3
f1 (ppm)

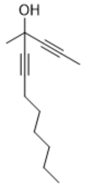

1j

Analysis: ^{13}C NMR
Solvent: $CDCl_3$
Field strength: 151 MHz

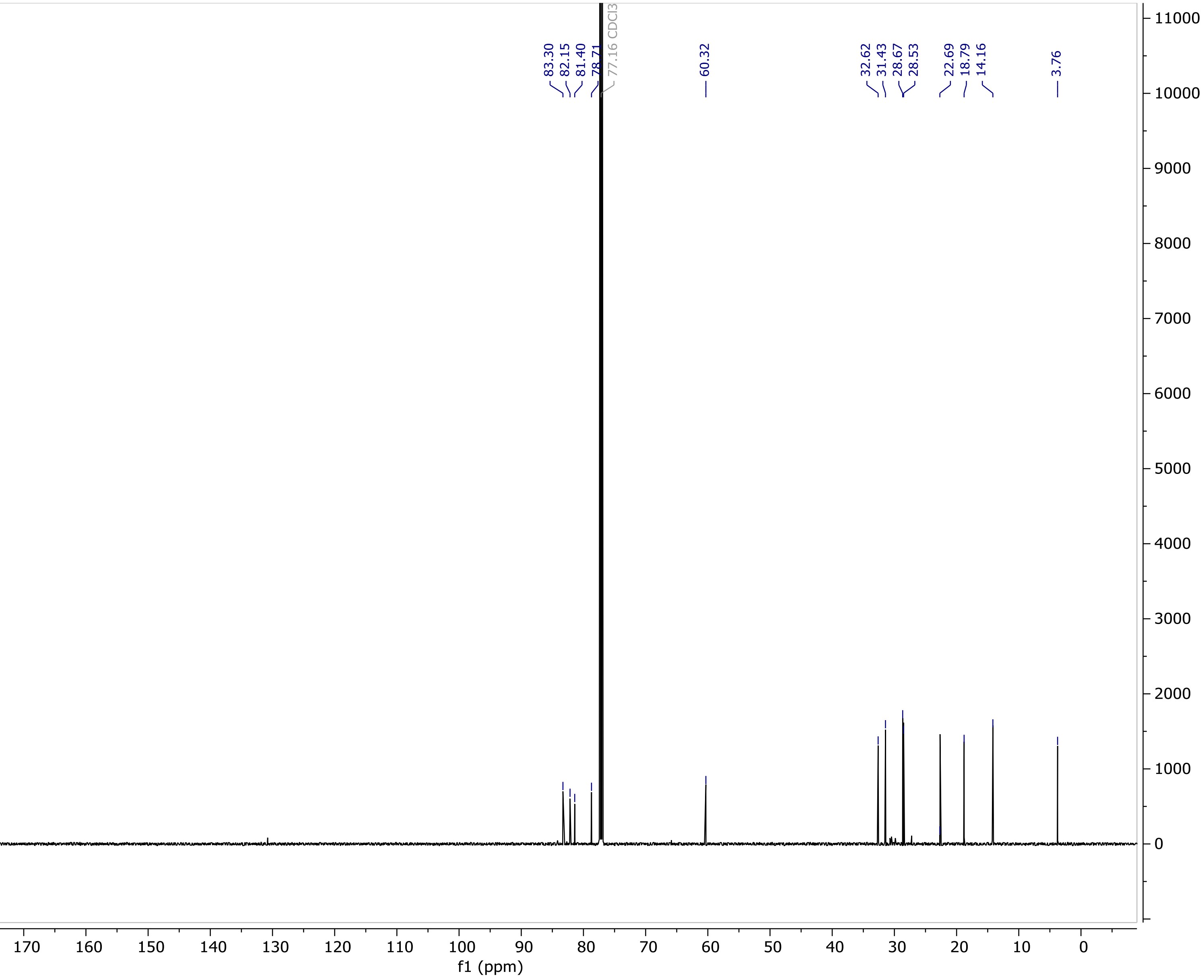

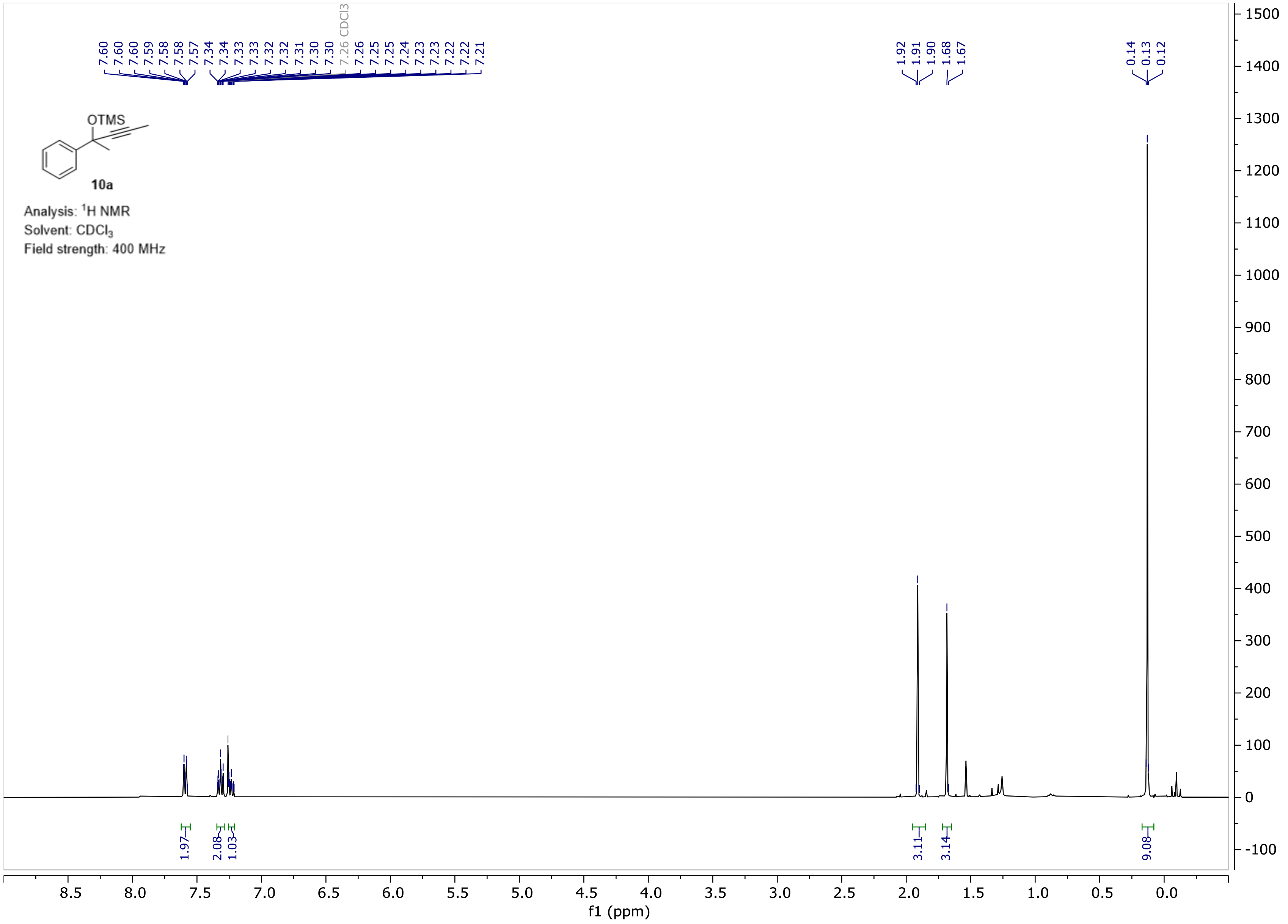

OTMS
10a
Analysis: ¹H NMR
Solvent: CDCl₃
Field strength: 400 MHz
7.60 7.60 7.59 7.58 7.57 7.34 7.34 7.33 7.33 7.32 7.32 7.31 7.30 7.30 7.26 CDCl3 7.26 7.25 7.25 7.24 7.23 7.23 7.22 7.22 7.21
1.92 1.91 1.90 1.68 1.67
0.14 0.13 0.12
1.97 2.08 1.03 3.11 3.14 9.08
f1 (ppm)

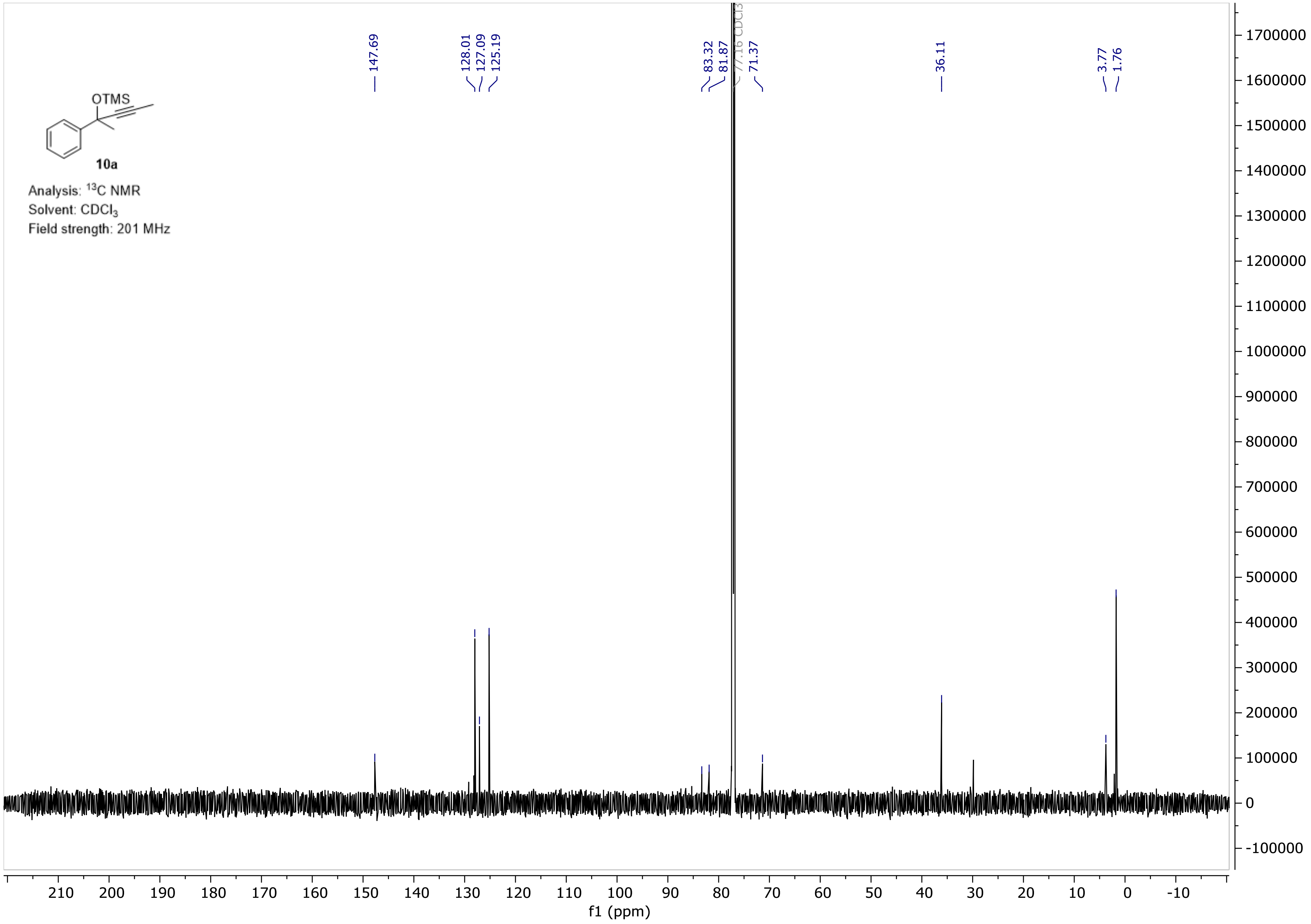
OTMS
10a
Analysis: 13C NMR
Solvent: CDCl3
Field strength: 201 MHz
147.69
128.01
127.09
125.19
83.32
81.87
77.16 CDCl3
71.37
36.11
3.77
1.76
f1 (ppm)

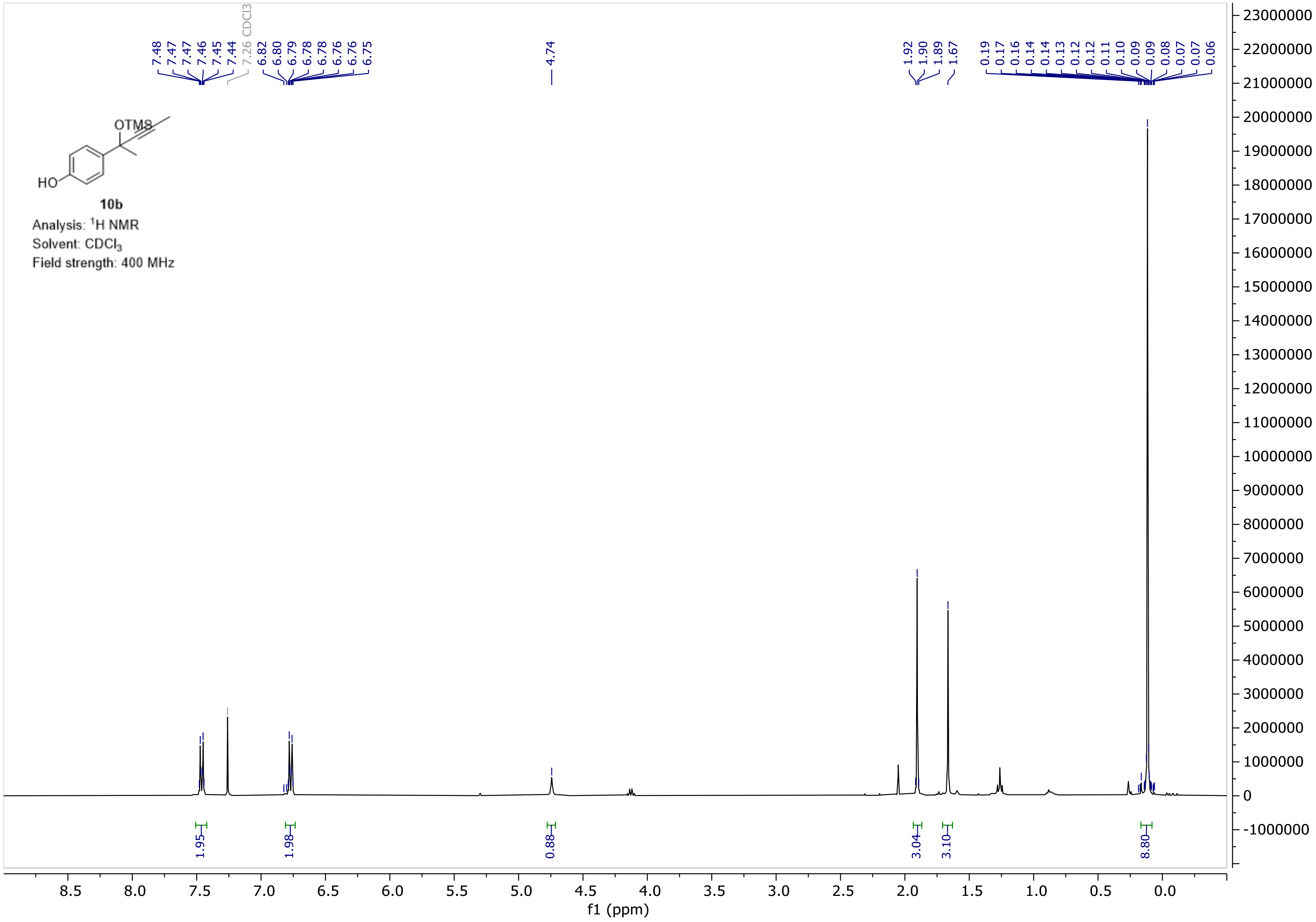

7.48
7.47
7.47
7.46
7.45
7.44
7.26 CDCl3
6.82
6.80
6.79
6.78
6.78
6.76
6.76
6.75
4.74
1.92
1.90
1.89
1.67
0.19
0.17
0.16
0.14
0.14
0.13
0.12
0.12
0.11
0.11
0.10
0.09
0.09
0.08
0.07
0.07
0.06
OTMS
HO
10b
Analysis: 1H NMR
Solvent: CDCl3
Field strength: 400 MHz
1.95
1.98
0.88
3.04
3.10
8.80
f1 (ppm)

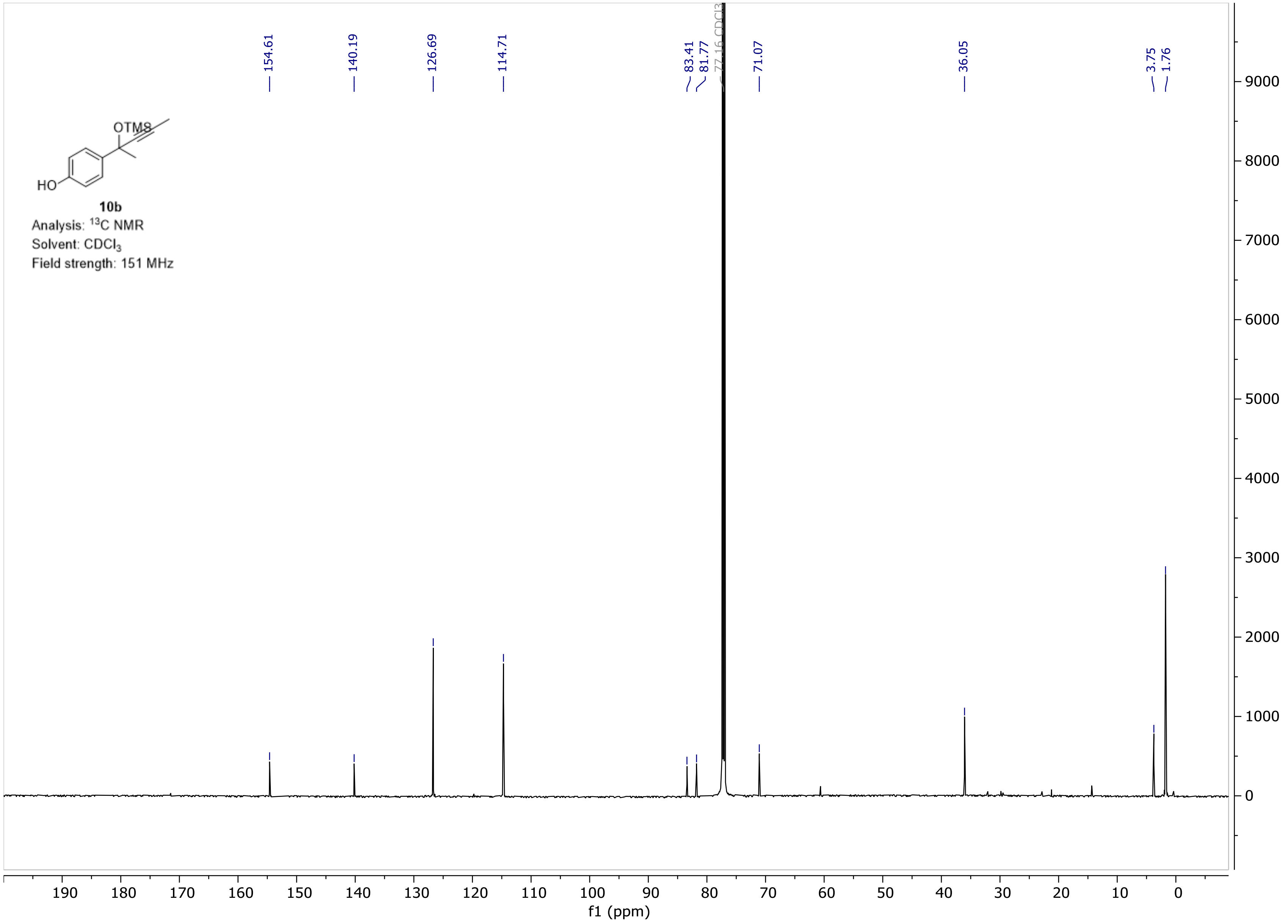
154.61
140.19
126.69
114.71
83.41
81.77
77.16 CDCl3
71.07
36.05
3.75
1.76
OTMS
HO
10b
Analysis: 13C NMR
Solvent: CDCl3
Field strength: 151 MHz
f1 (ppm)

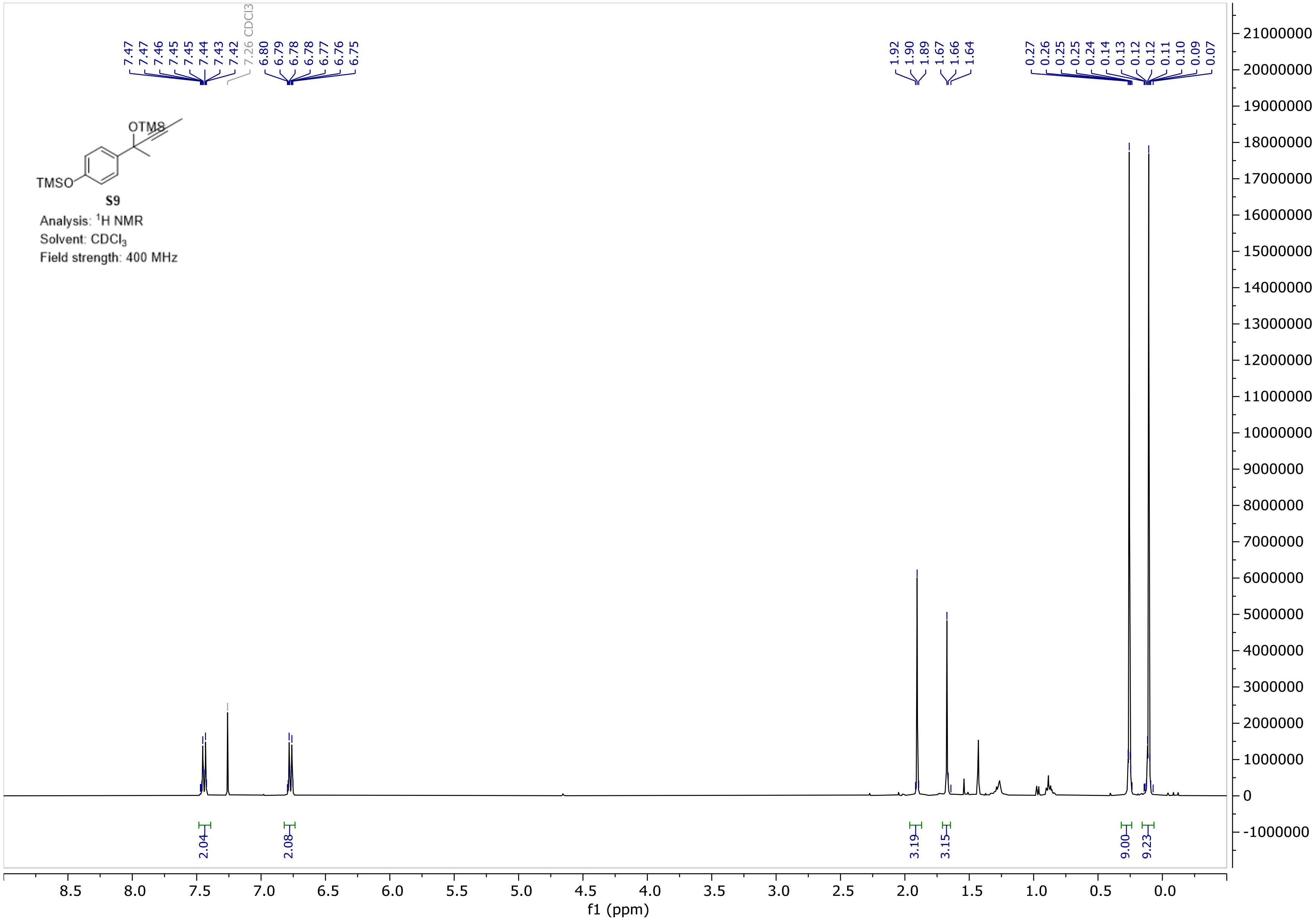

7.47
7.47
7.46
7.45
7.45
7.44
7.43
7.42
7.26 CDCl3
6.80
6.79
6.78
6.78
6.77
6.76
6.75
1.92
1.90
1.89
1.67
1.66
1.64
0.27
0.26
0.25
0.25
0.24
0.14
0.13
0.12
0.12
0.11
0.10
0.09
0.07
OTMS
TMSO
S9
Analysis: ¹H NMR
Solvent: CDCl3
Field strength: 400 MHz
2.04
2.08
3.19
3.15
9.00
9.23
f1 (ppm)

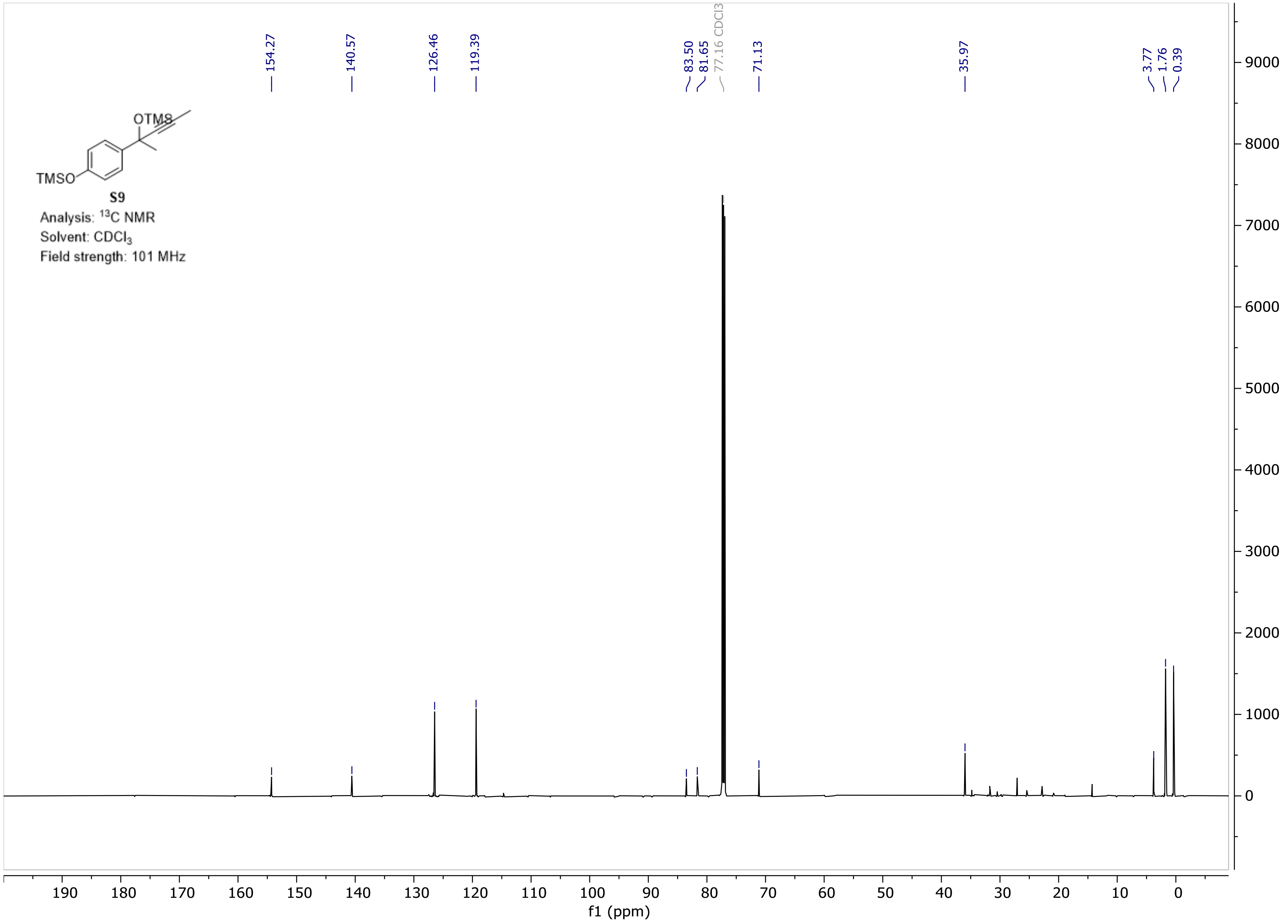
154.27
140.57
126.46
119.39
83.50
81.65
77.16 CDCl3
71.13
35.97
3.77
1.76
0.39
OTMS
TMSO
S9
Analysis: 13C NMR
Solvent: CDCl3
Field strength: 101 MHz
9000
8000
7000
6000
5000
4000
3000
2000
1000
0
190
180
170
160
150
140
130
120
110
100
90
80
70
60
50
40
30
20
10
0
f1 (ppm)

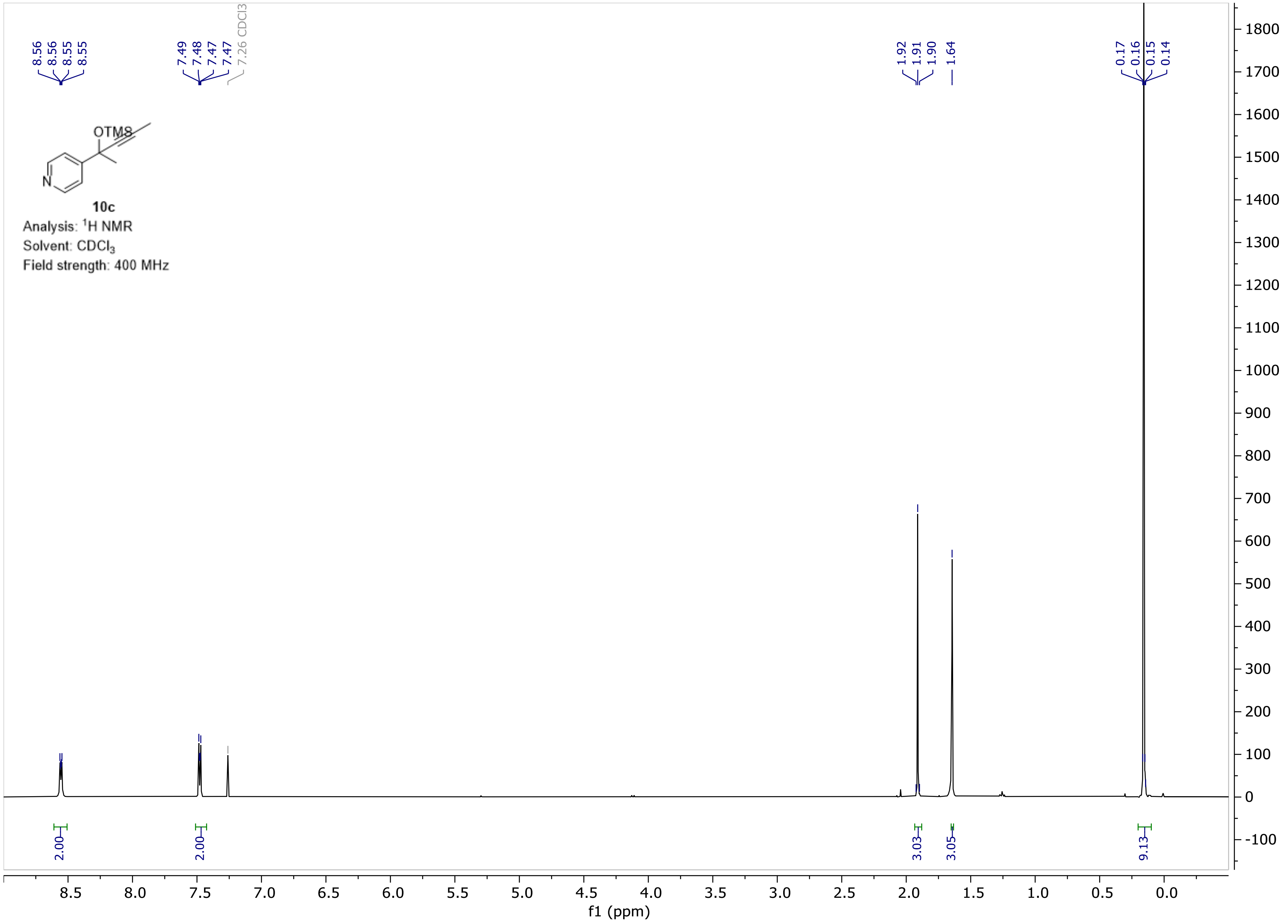

8.56
8.56
8.55
8.55
7.49
7.48
7.47
7.47
7.26 CDCl3
1.92
1.91
1.90
1.64
0.17
0.16
0.15
0.14
OTMS
N
10c
Analysis: 1H NMR
Solvent: CDCl3
Field strength: 400 MHz
2.00
2.00
3.03
3.05
9.13
f1 (ppm)

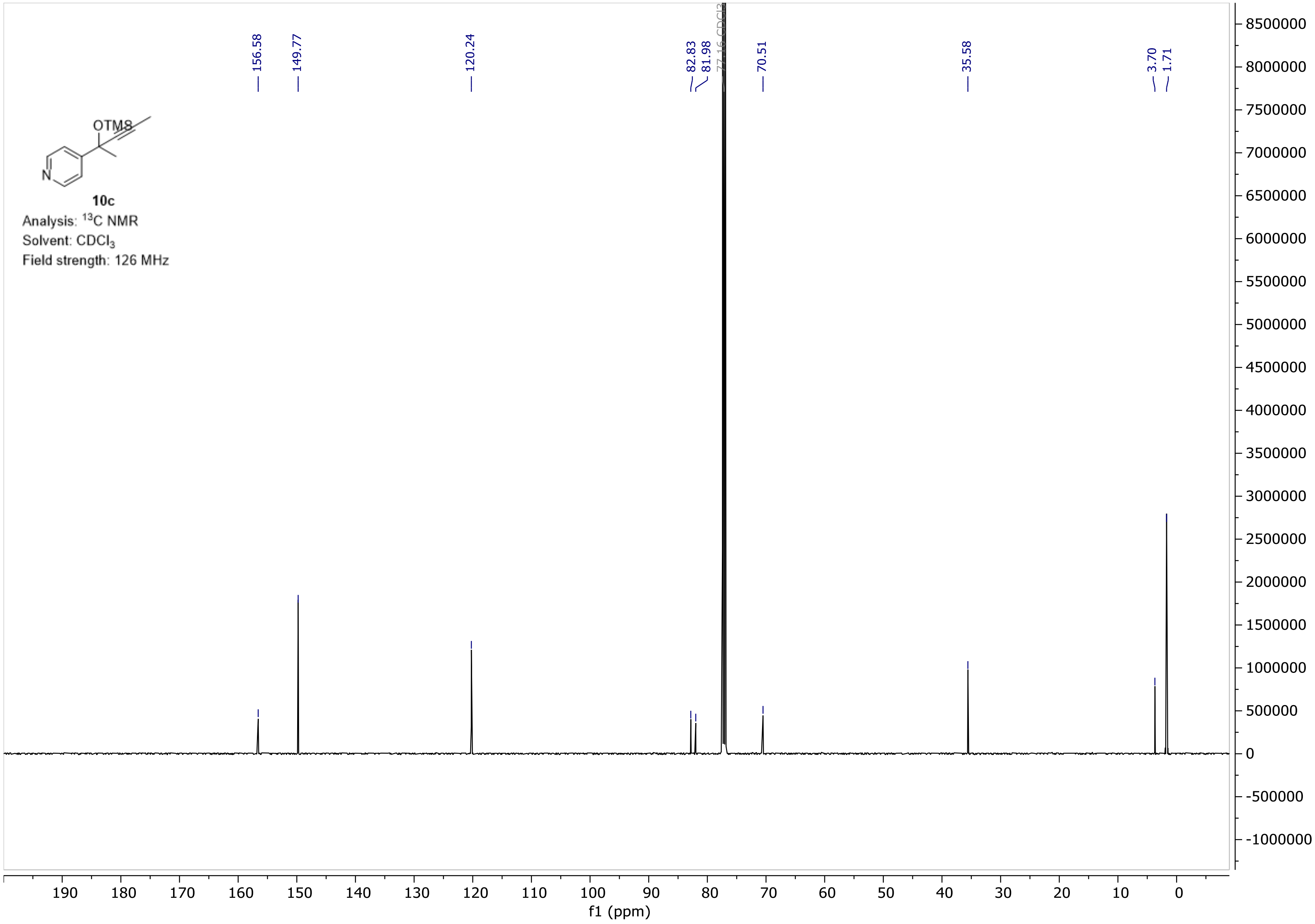
OTMS
N
10c
Analysis: ^{13}C NMR
Solvent: $CDCl_3$
Field strength: 126 MHz
156.58
149.77
120.24
82.83
81.98
77.16 CDCl3
70.51
35.58
3.70
1.71
8500000
8000000
7500000
7000000
6500000
6000000
5500000
5000000
4500000
4000000
3500000
3000000
2500000
2000000
1500000
1000000
500000
0
-500000
-1000000
190 180 170 160 150 140 130 120 110 100 90 80 70 60 50 40 30 20 10 0
f1 (ppm)

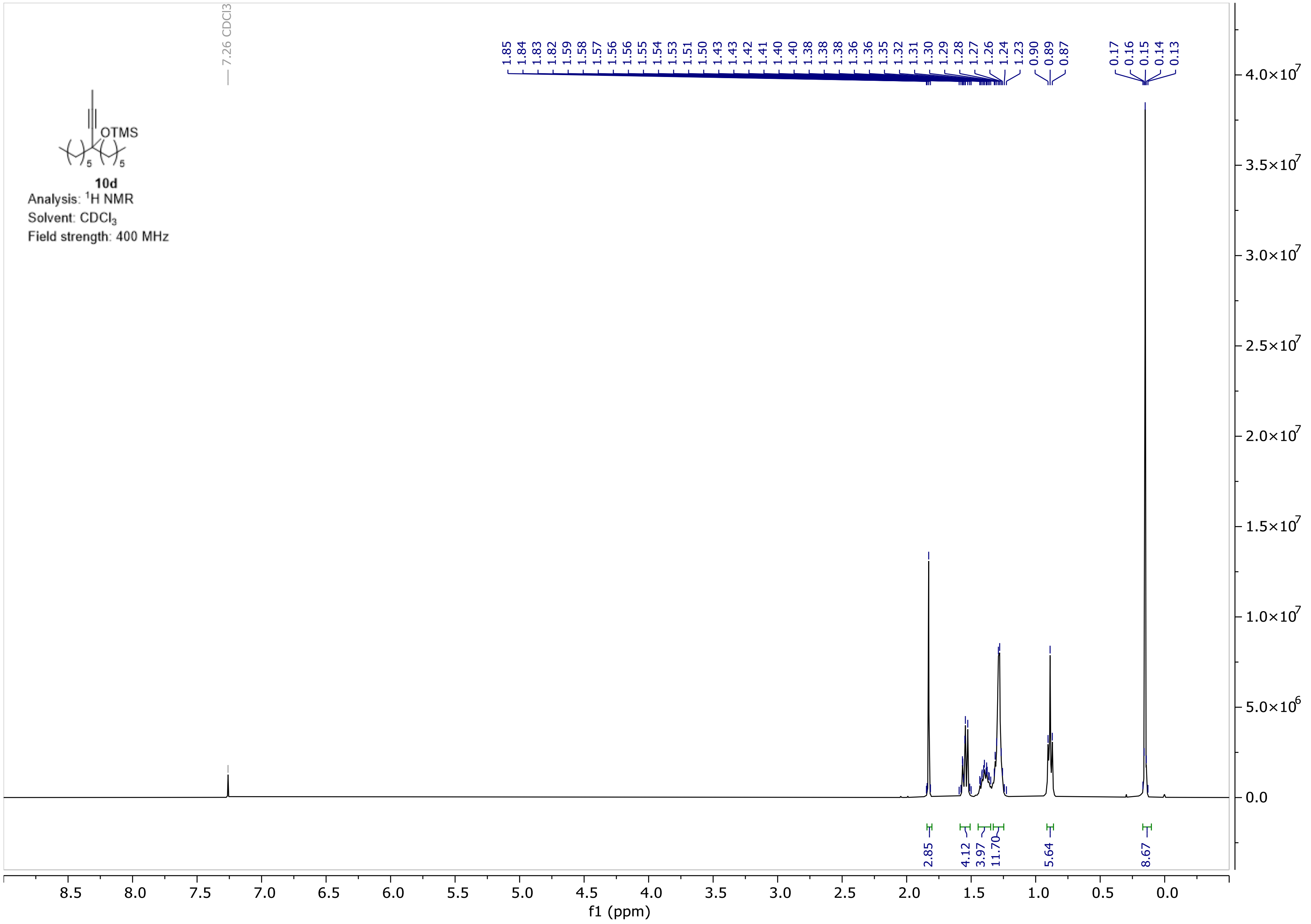

OTMS
10d
Analysis: ^{1}H NMR
Solvent: $CDCl_3$
Field strength: 400 MHz
7.26 CDCl3
1.85 1.84 1.83 1.82 1.59 1.58 1.57 1.56 1.56 1.55 1.54 1.53 1.51 1.50 1.43 1.43 1.42 1.41 1.40 1.40 1.38 1.38 1.38 1.36 1.36 1.35 1.32 1.31 1.30 1.29 1.28 1.27 1.26 1.24 1.23 0.90 0.89 0.87
0.17 0.16 0.15 0.14 0.13
2.85 4.12 3.97 11.70 5.64 8.67
f1 (ppm)

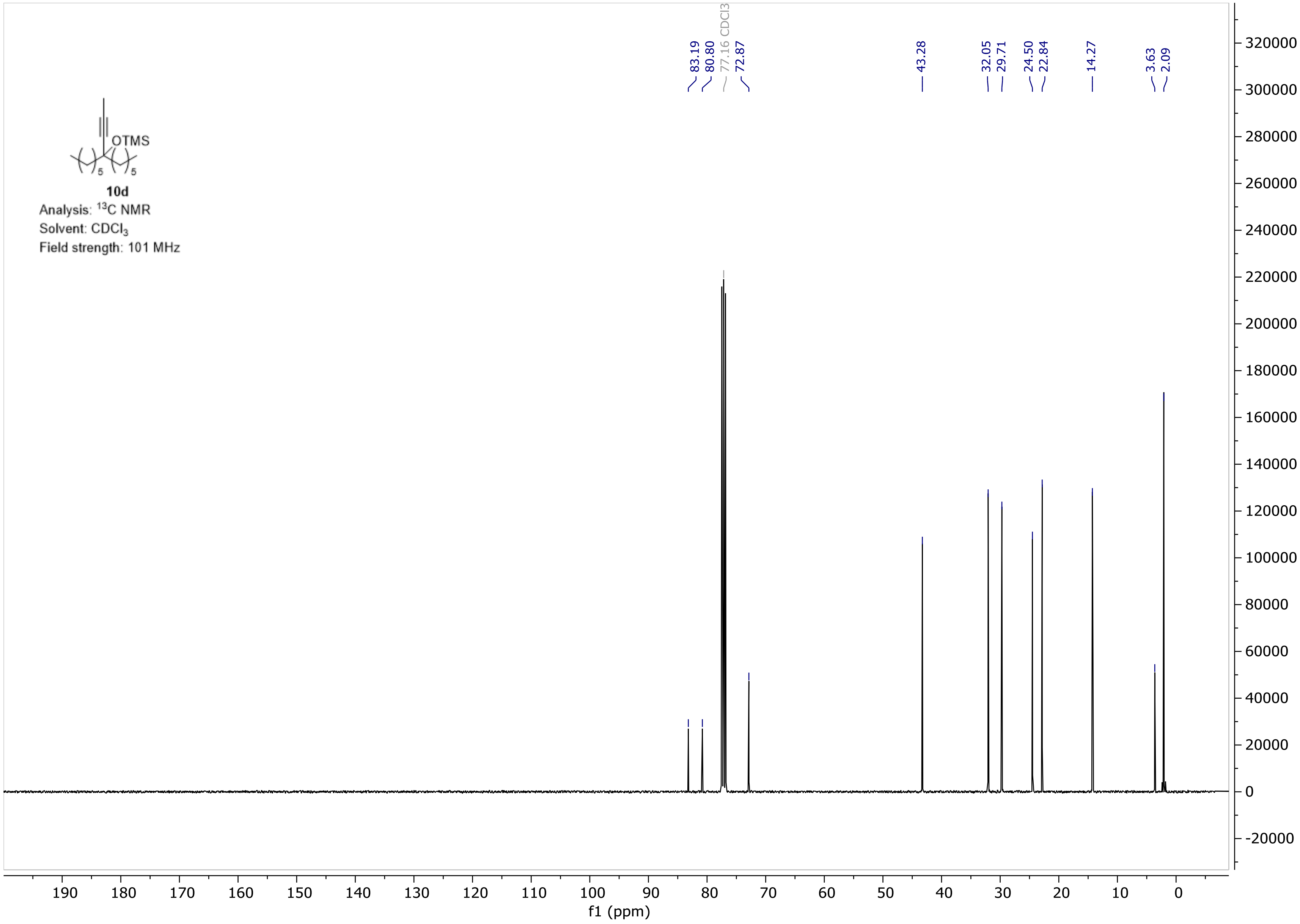

83.19
80.80
77.16 CDCl3
72.87
43.28
32.05
29.71
24.50
22.84
14.27
3.63
2.09
OTMS
5
5
10d
Analysis: 13C NMR
Solvent: CDCl3
Field strength: 101 MHz
320000
300000
280000
260000
240000
220000
200000
180000
160000
140000
120000
100000
80000
60000
40000
20000
0
-20000
190
180
170
160
150
140
130
120
110
100
90
80
70
60
50
40
30
20
10
0
f1 (ppm)

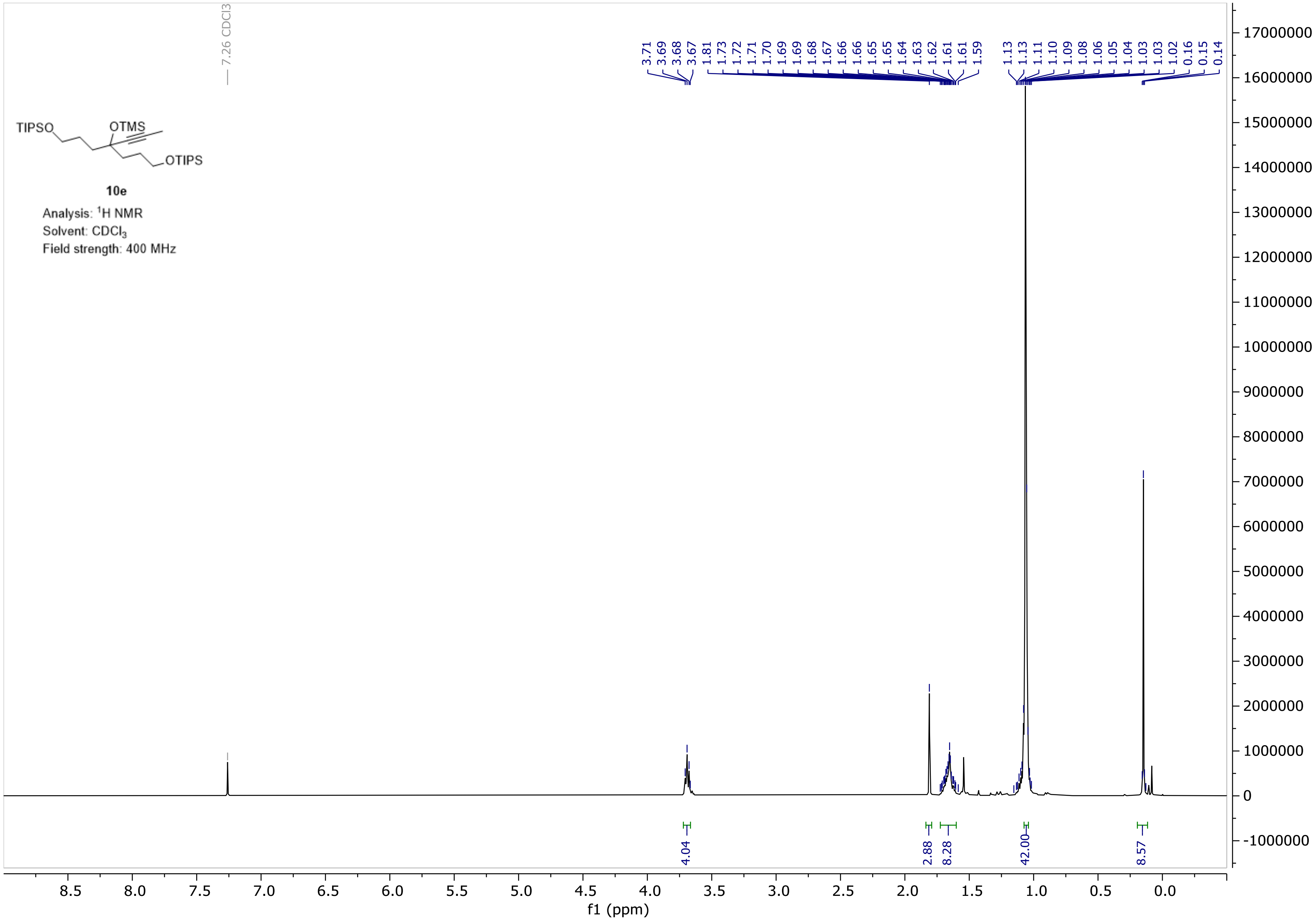

TIPSO
OTMS
OTIPS
10e
Analysis: ¹H NMR
Solvent: CDCl3
Field strength: 400 MHz
7.26 CDCl3
f1 (ppm)

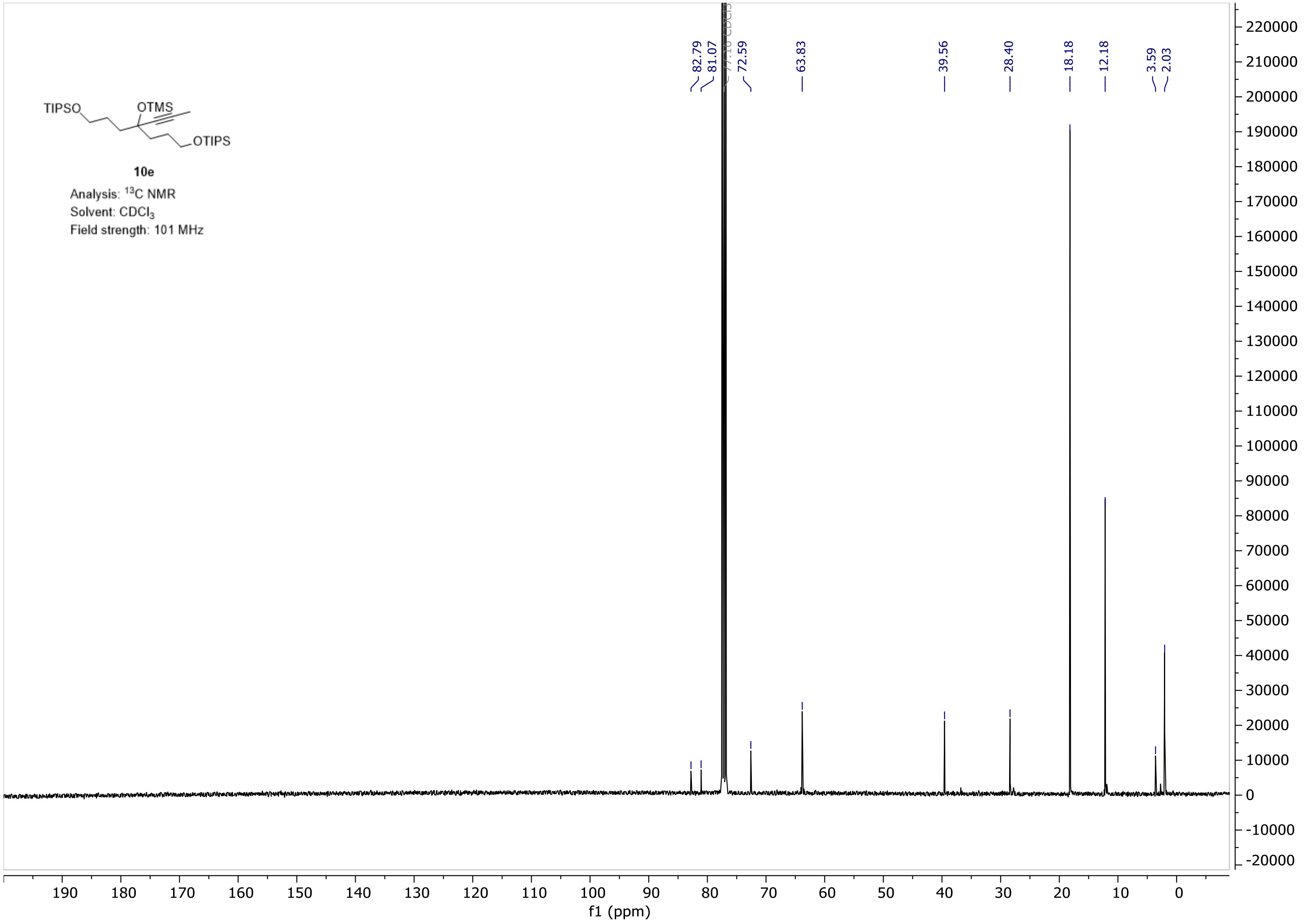
TIPSO
OTMS
OTIPS
10e
Analysis: 13C NMR
Solvent: CDCl3
Field strength: 101 MHz
82.79
81.07
72.59
63.83
39.56
28.40
18.18
12.18
3.59
2.03
f1 (ppm)

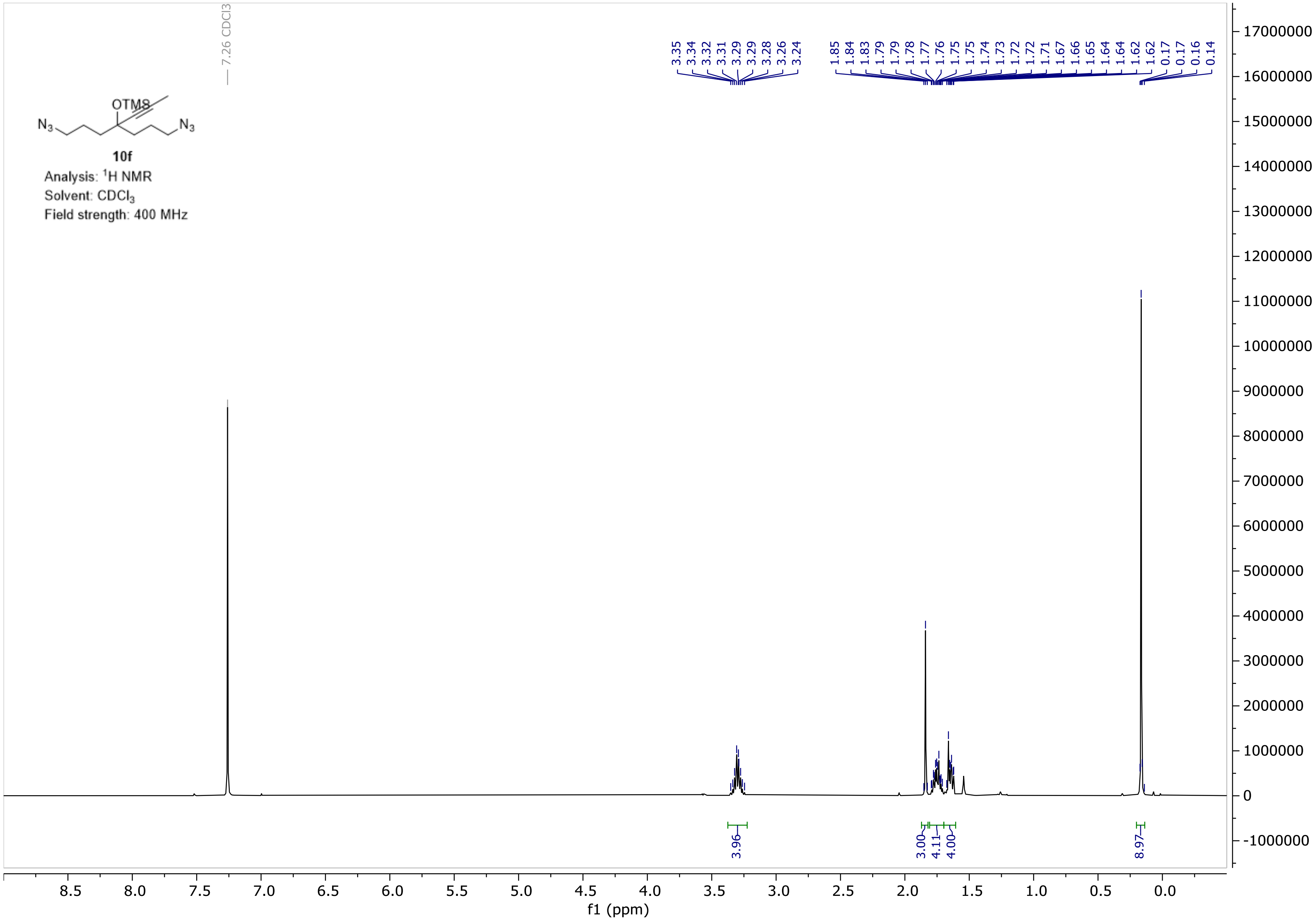

OTMS
N3
N3
10f
Analysis: 1H NMR
Solvent: CDCl3
Field strength: 400 MHz
7.26 CDCl3
3.35 3.34 3.32 3.31 3.29 3.29 3.28 3.26 3.24
1.85 1.84 1.83 1.79 1.78 1.77 1.76 1.75 1.75 1.74 1.73 1.72 1.72 1.71 1.67 1.66 1.65 1.64 1.64 1.62 1.62 0.17 0.17 0.16 0.14
3.96
3.00
4.11
4.00
8.97
f1 (ppm)

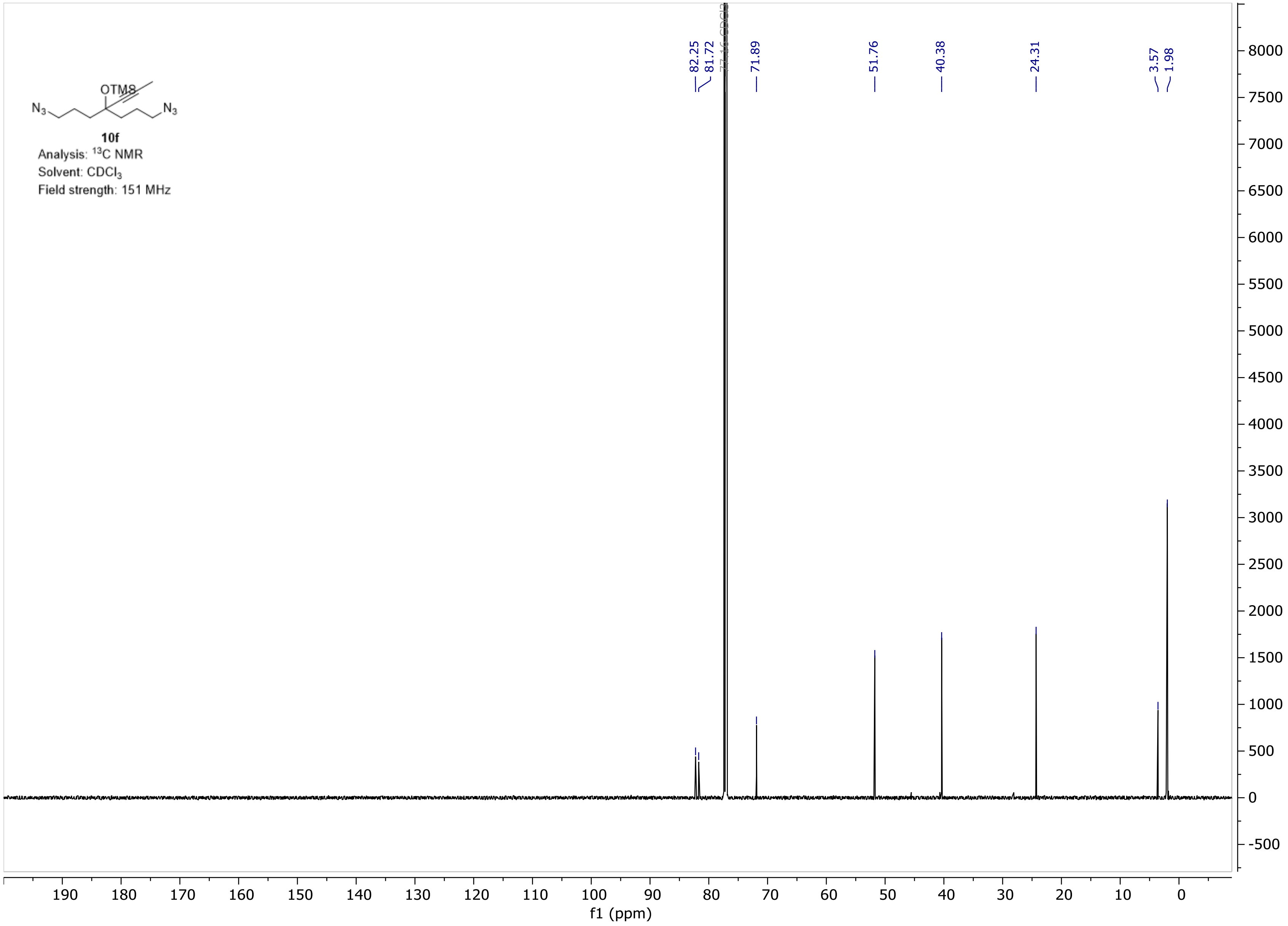

OTMS
N3
N3
10f
Analysis: 13C NMR
Solvent: CDCl3
Field strength: 151 MHz
82.25
81.72
71.89
51.76
40.38
24.31
3.57
1.98
8000
7500
7000
6500
6000
5500
5000
4500
4000
3500
3000
2500
2000
1500
1000
500
0
-500
190 180 170 160 150 140 130 120 110 100 90 80 70 60 50 40 30 20 10 0
f1 (ppm)

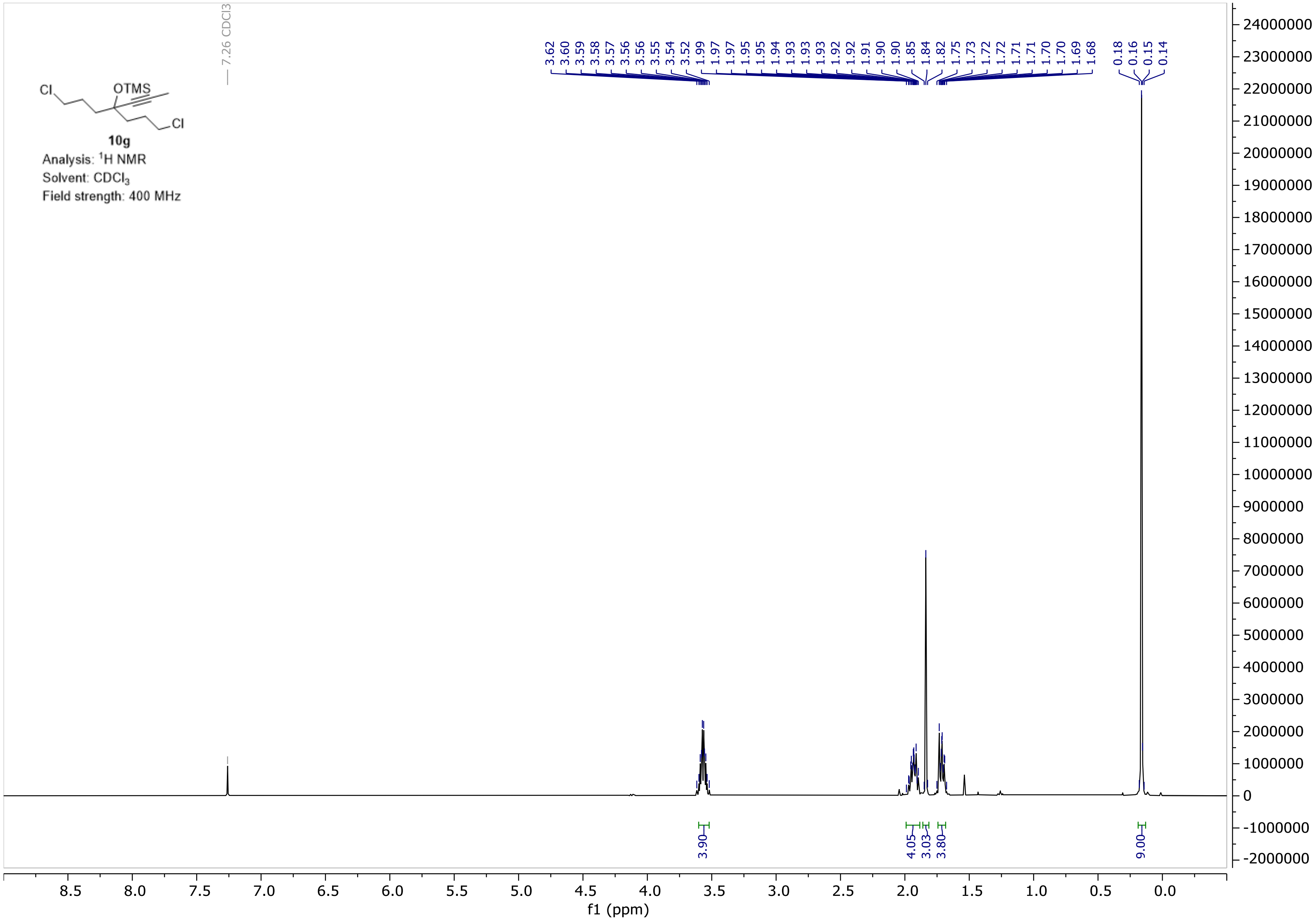

Cl
OTMS
Cl
10g
Analysis: 1H NMR
Solvent: CDCl3
Field strength: 400 MHz
7.26 CDCl3
3.62 3.60 3.59 3.58 3.57 3.56 3.56 3.55 3.54 3.52 1.99 1.97 1.97 1.95 1.95 1.94 1.93 1.93 1.93 1.92 1.92 1.91 1.90 1.90 1.85 1.84 1.82 1.75 1.73 1.72 1.72 1.71 1.71 1.70 1.70 1.69 1.68 0.18 0.16 0.15 0.14
3.90 4.05 3.03 3.80 9.00
f1 (ppm)

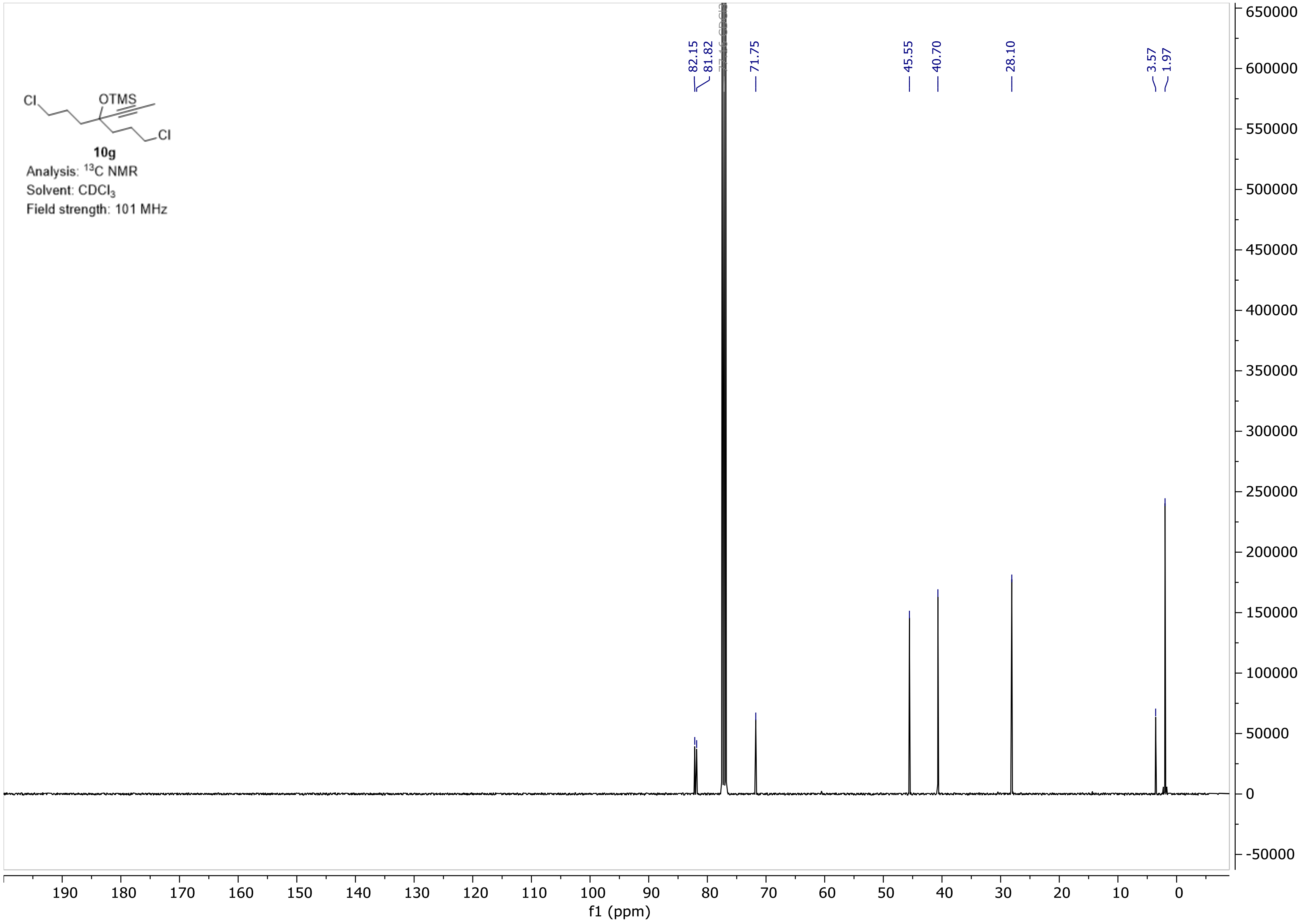

10g
Analysis: 13C NMR
Solvent: CDCl3
Field strength: 101 MHz
82.15
81.82
71.75
45.55
40.70
28.10
3.57
1.97
f1 (ppm)

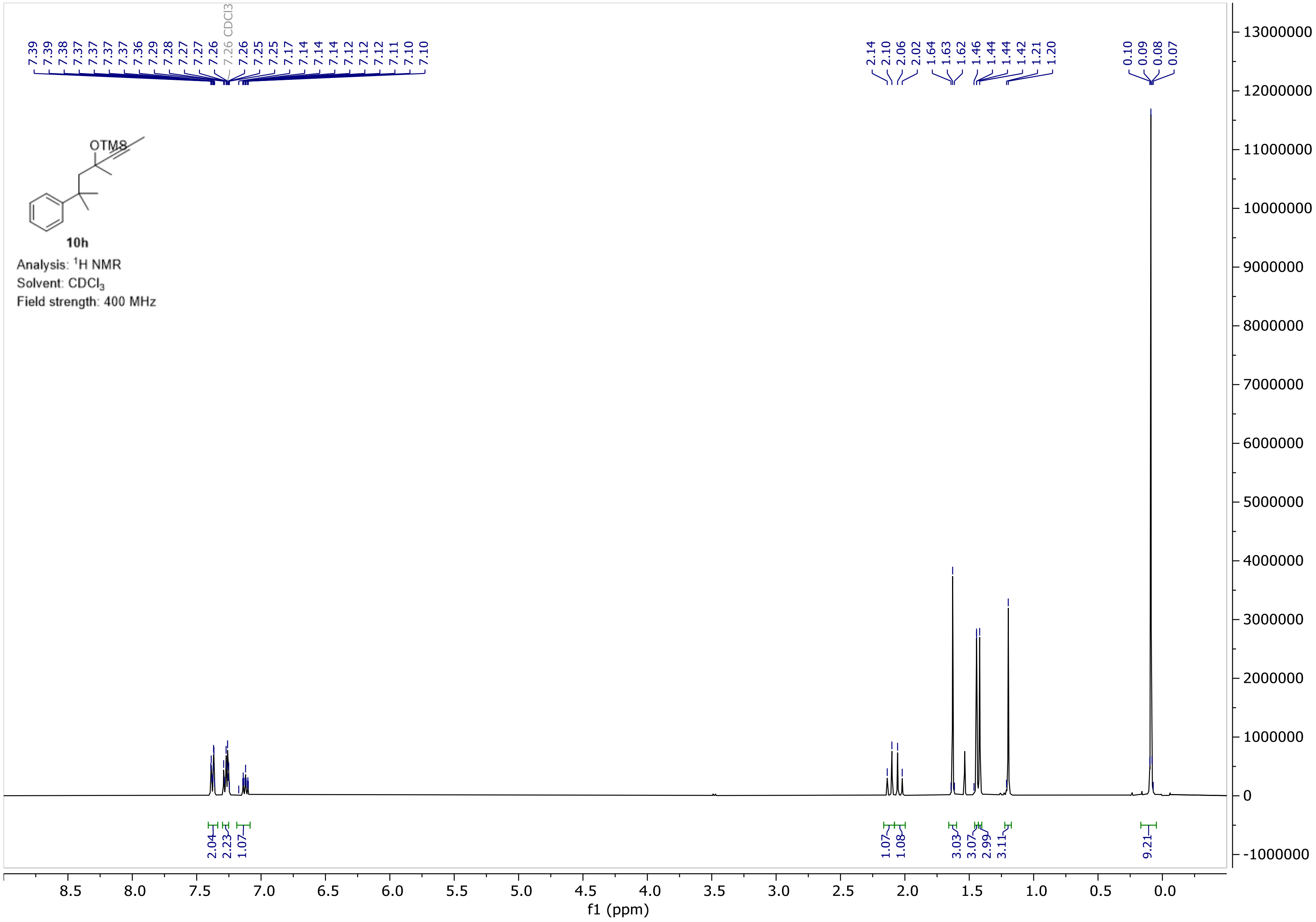

10h
Analysis: ¹H NMR
Solvent: CDCl3
Field strength: 400 MHz
f1 (ppm)

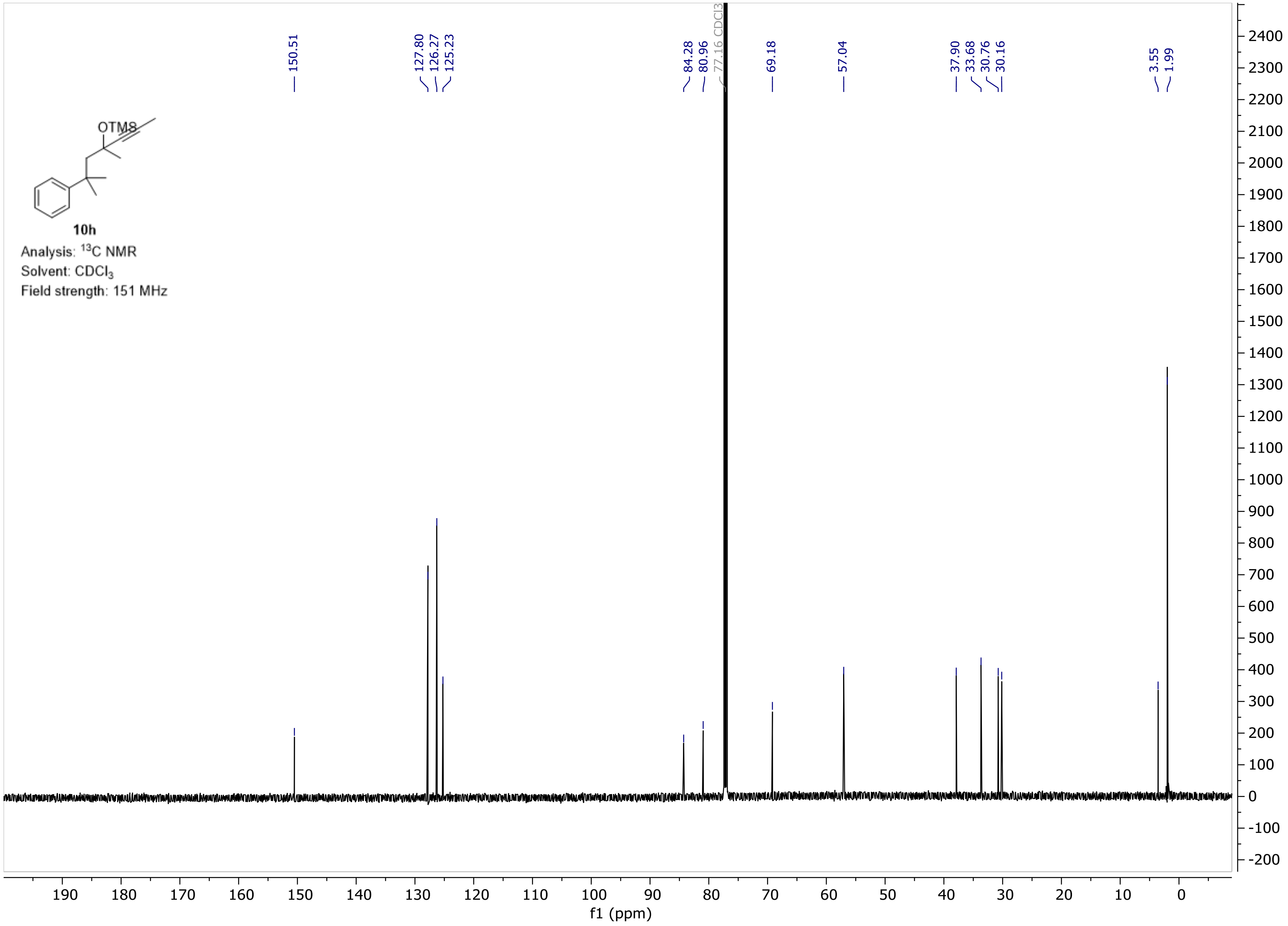

150.51
127.80
126.27
125.23
84.28
80.96
77.16 CDCl3
69.18
57.04
37.90
33.68
30.76
30.16
3.55
1.99
OTMS
10h
Analysis: ^{13}C NMR
Solvent: $CDCl_3$
Field strength: 151 MHz
f1 (ppm)

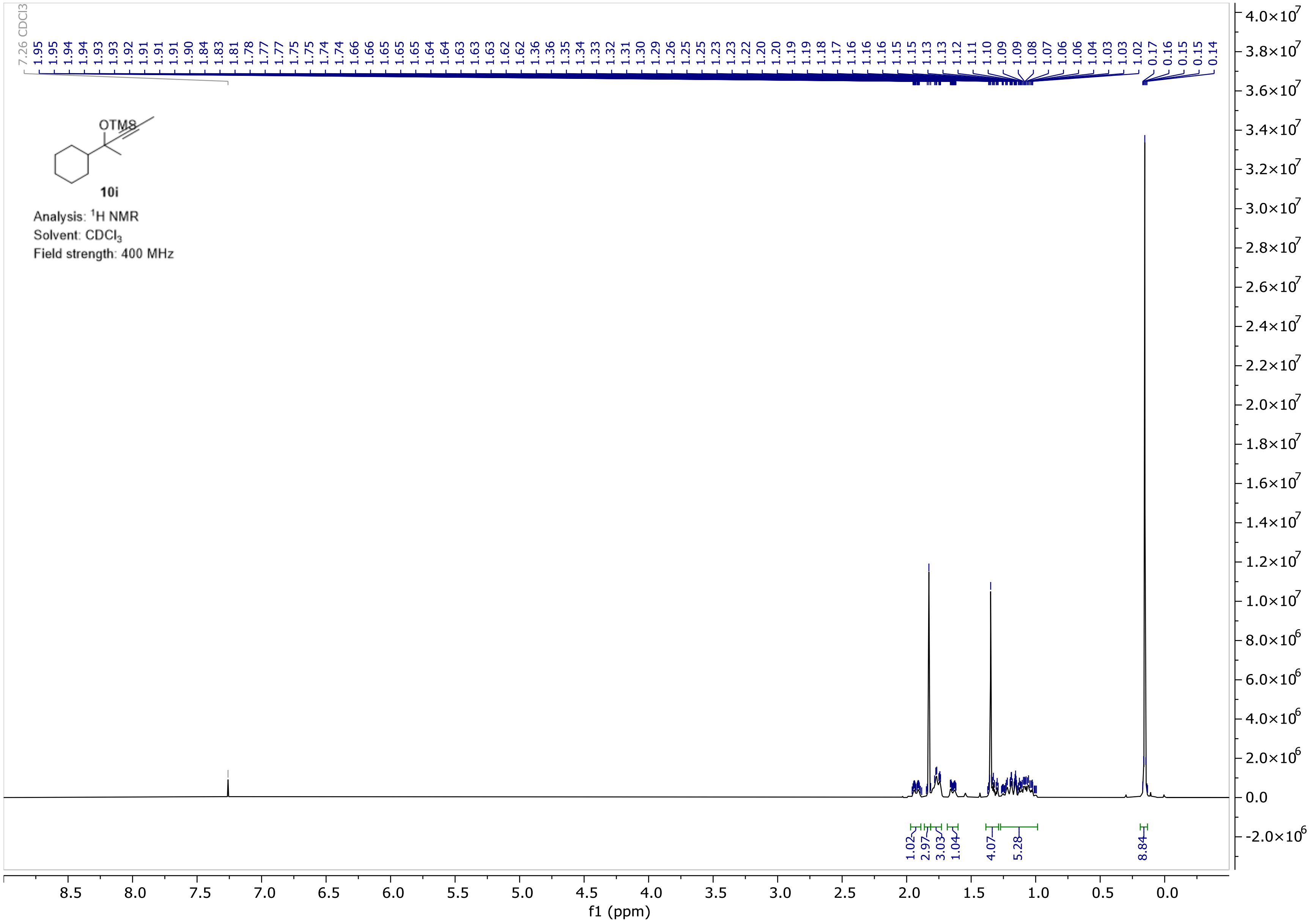

OTMS
10i
Analysis: 1H NMR
Solvent: CDCl3
Field strength: 400 MHz
f1 (ppm)

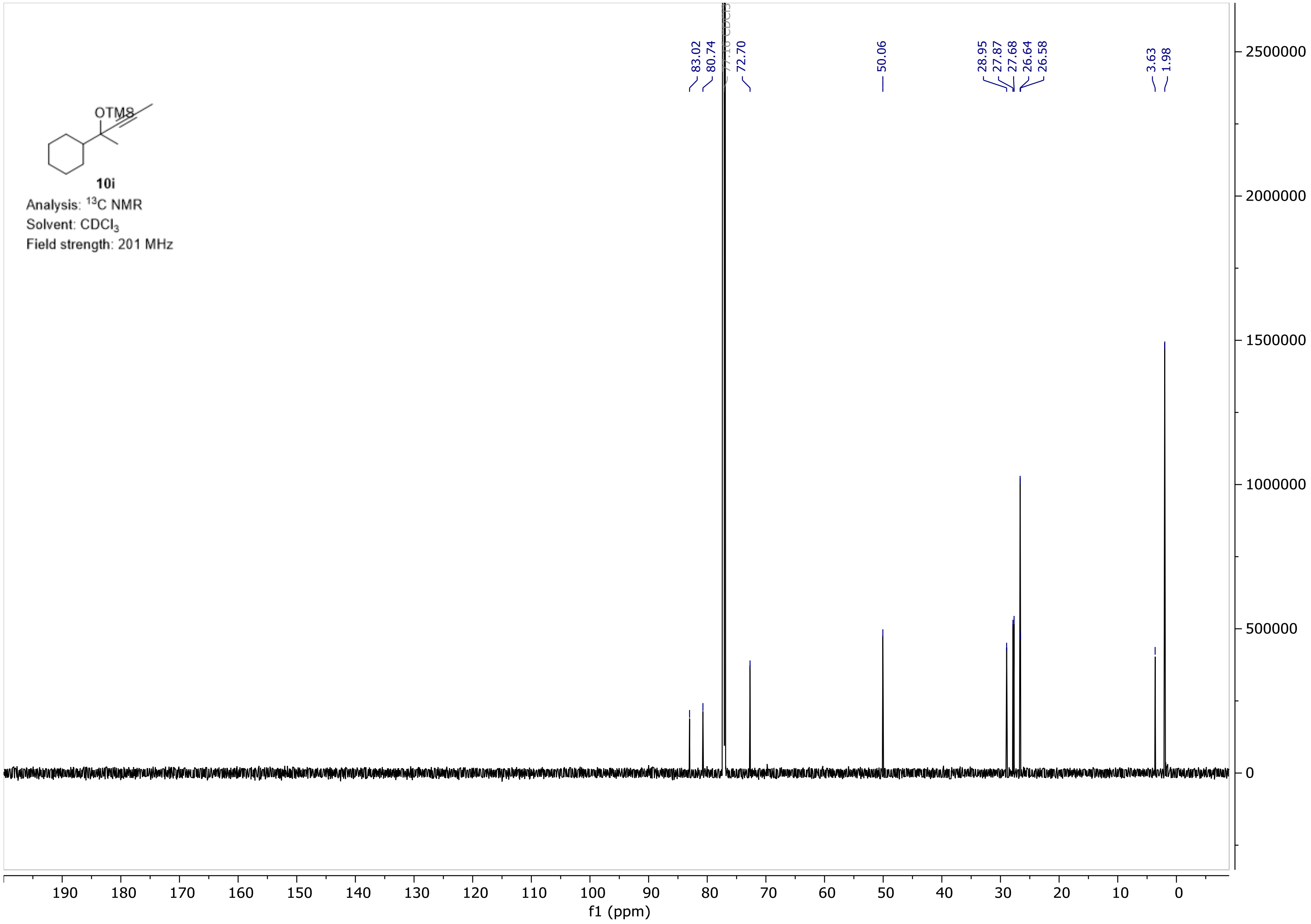
83.02
80.74
77.16 CDCl3
72.70
50.06
28.95
27.87
27.68
26.64
26.58
3.63
1.98
OTMS
10i
Analysis: ^{13}C NMR
Solvent: $CDCl_3$
Field strength: 201 MHz
2500000
2000000
1500000
1000000
500000
0
190 180 170 160 150 140 130 120 110 100 90 80 70 60 50 40 30 20 10 0
f1 (ppm)

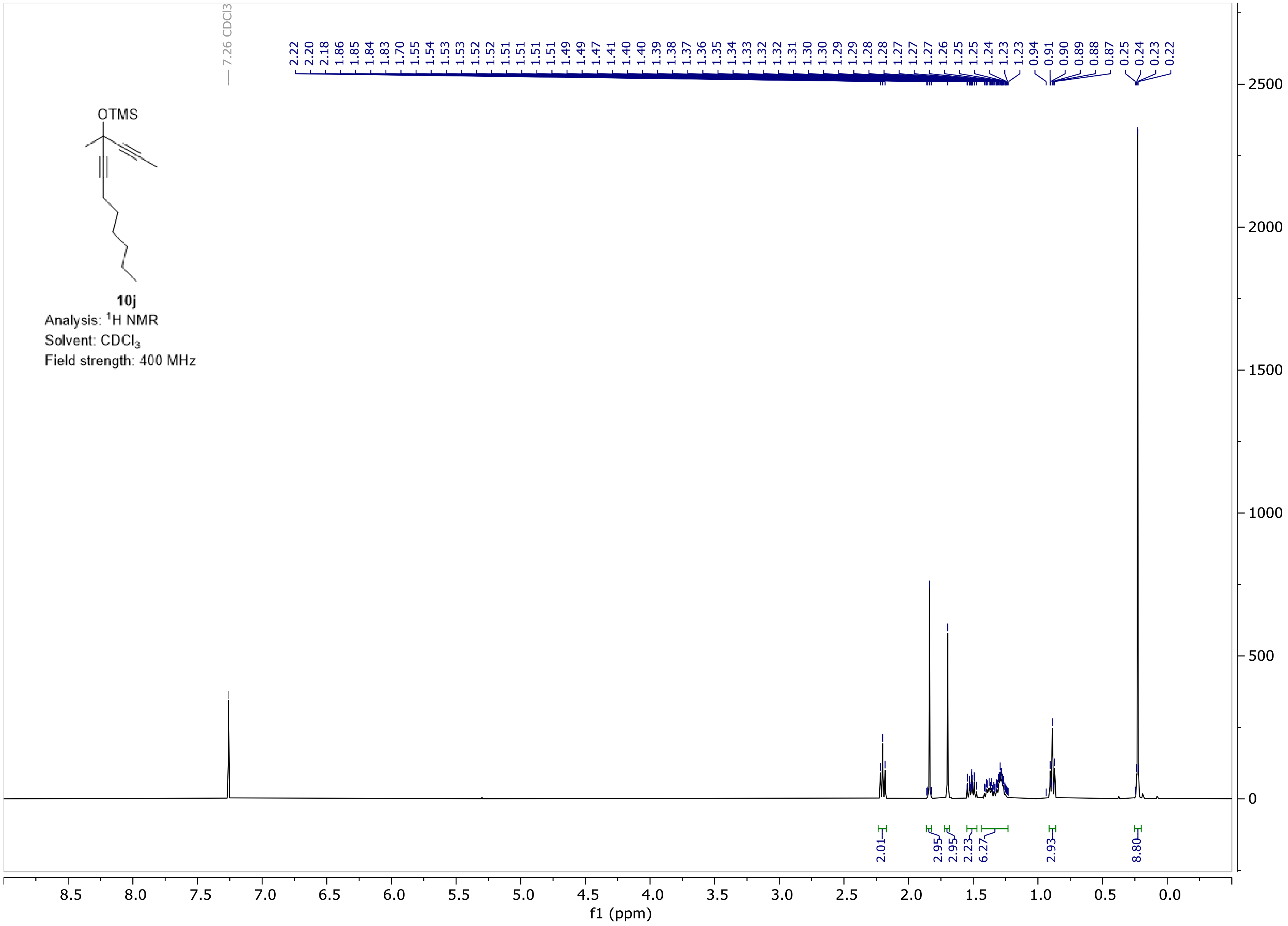

7.26 CDCl3
2.22 2.20 2.18 1.86 1.85 1.84 1.83 1.70 1.55 1.54 1.53 1.53 1.52 1.52 1.51 1.51 1.51 1.51 1.49 1.49 1.47 1.41 1.40 1.40 1.39 1.38 1.37 1.36 1.35 1.34 1.33 1.32 1.32 1.31 1.30 1.30 1.29 1.29 1.28 1.28 1.27 1.27 1.27 1.26 1.25 1.25 1.24 1.23 1.23 0.94 0.91 0.90 0.89 0.88 0.87 0.25 0.24 0.23 0.22
OTMS
10j
Analysis: ¹H NMR
Solvent: CDCl3
Field strength: 400 MHz
2.01 2.95 2.95 2.23 6.27 2.93 8.80
f1 (ppm)

10j
Analysis: ^{13}C NMR
Solvent: $CDCl_3$
Field strength: 151 MHz

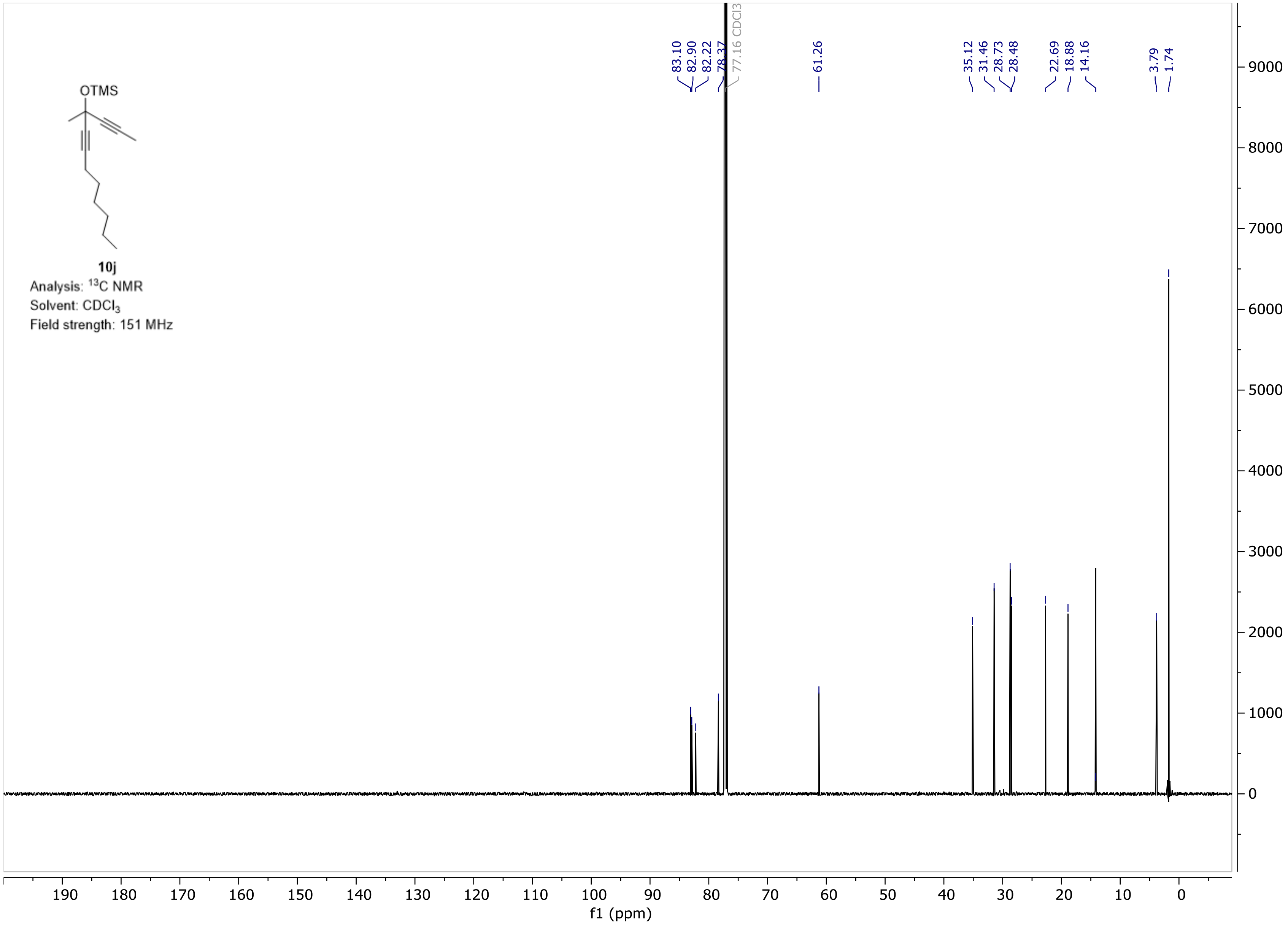

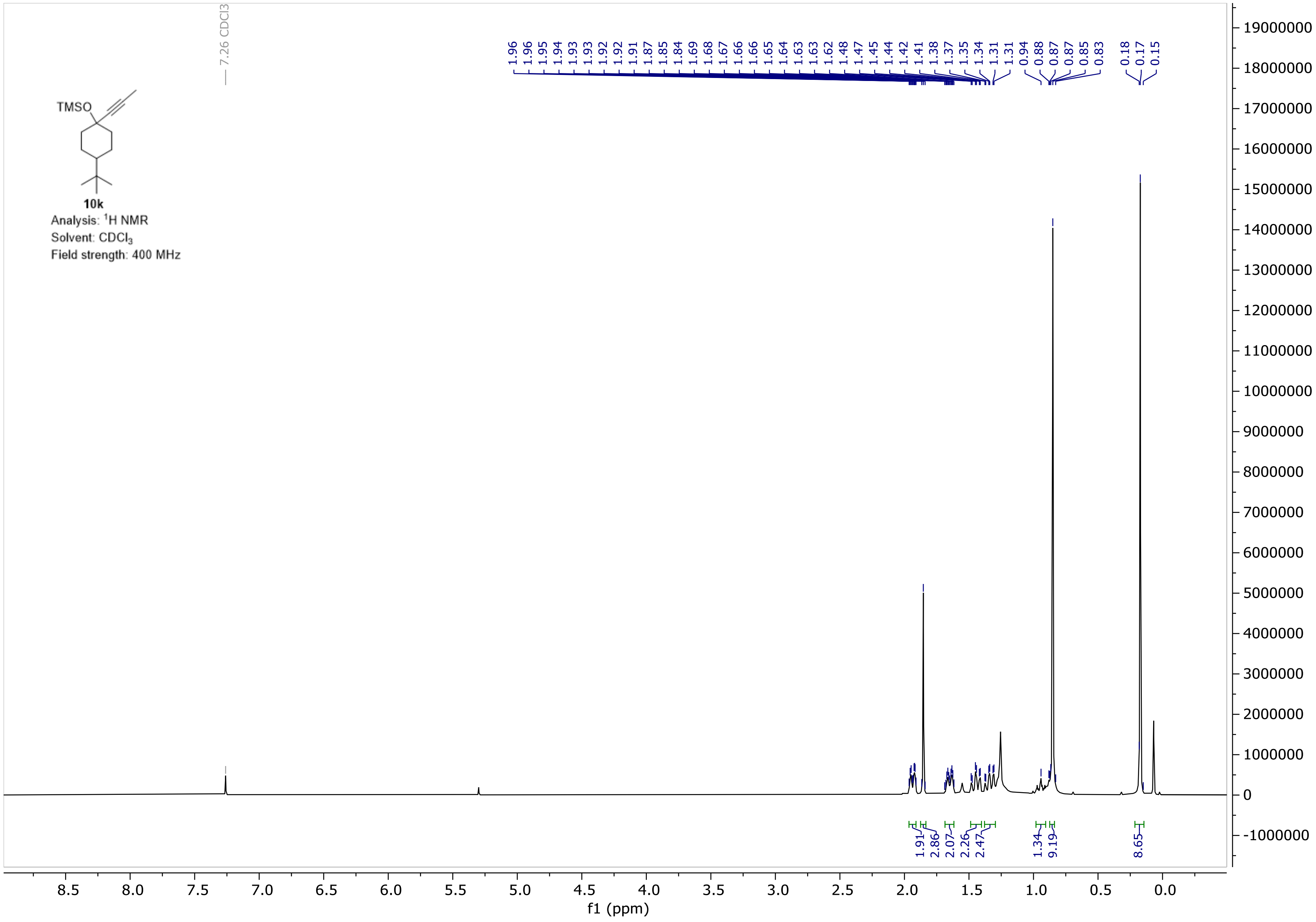

TMSO
10k
Analysis: 1H NMR
Solvent: CDCl3
Field strength: 400 MHz
7.26 CDCl3
f1 (ppm)

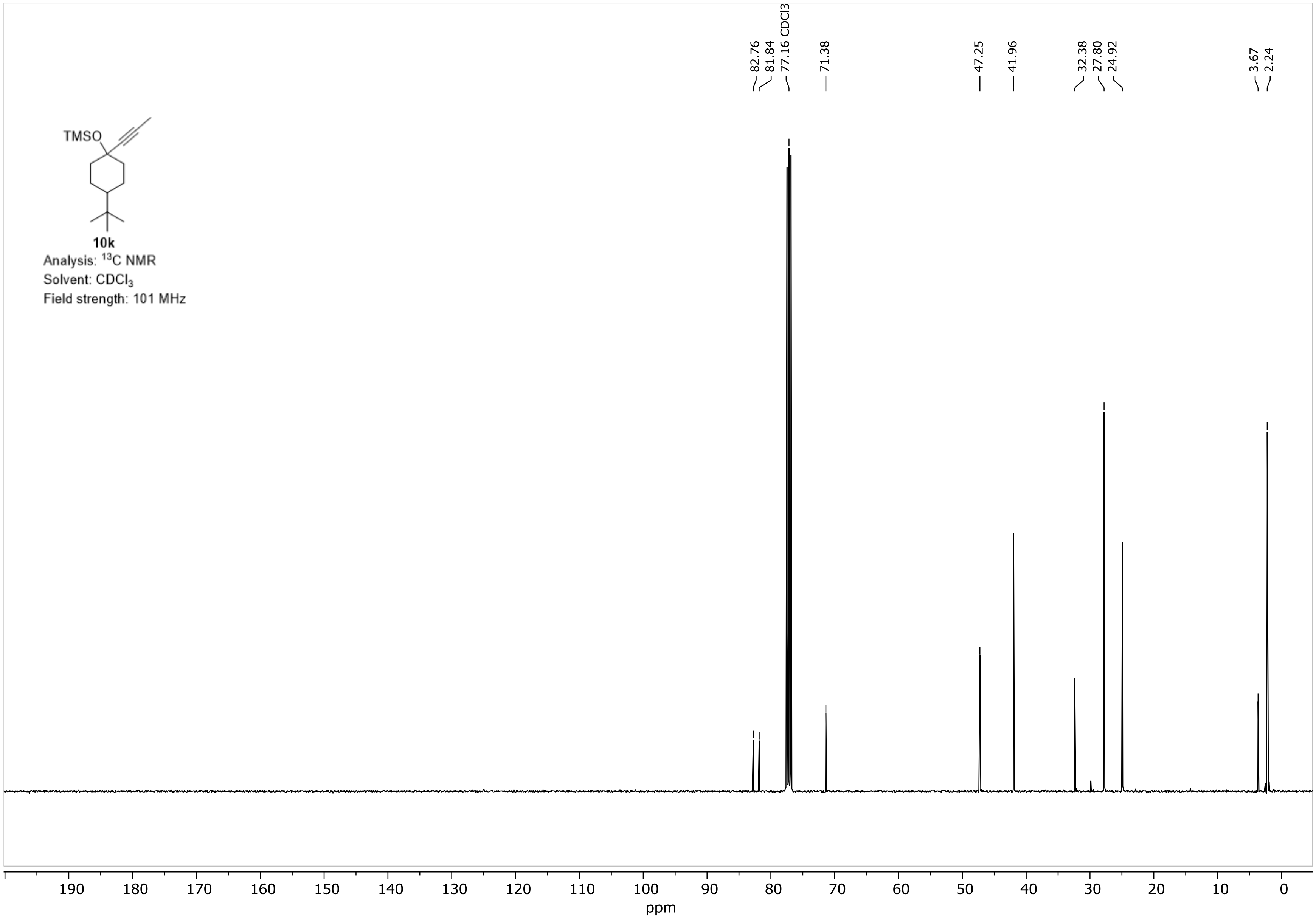

82.76
81.84
77.16 CDCl3
71.38
47.25
41.96
32.38
27.80
24.92
3.67
2.24
TMSO
10k
Analysis: 13C NMR
Solvent: CDCl3
Field strength: 101 MHz
190
180
170
160
150
140
130
120
110
100
90
80
70
60
50
40
30
20
10
0
ppm

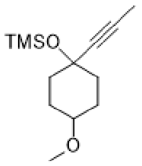

10l

Analysis: ^{1}H NMR

Solvent: $CDCl_3$

Field strength: 400 MHz

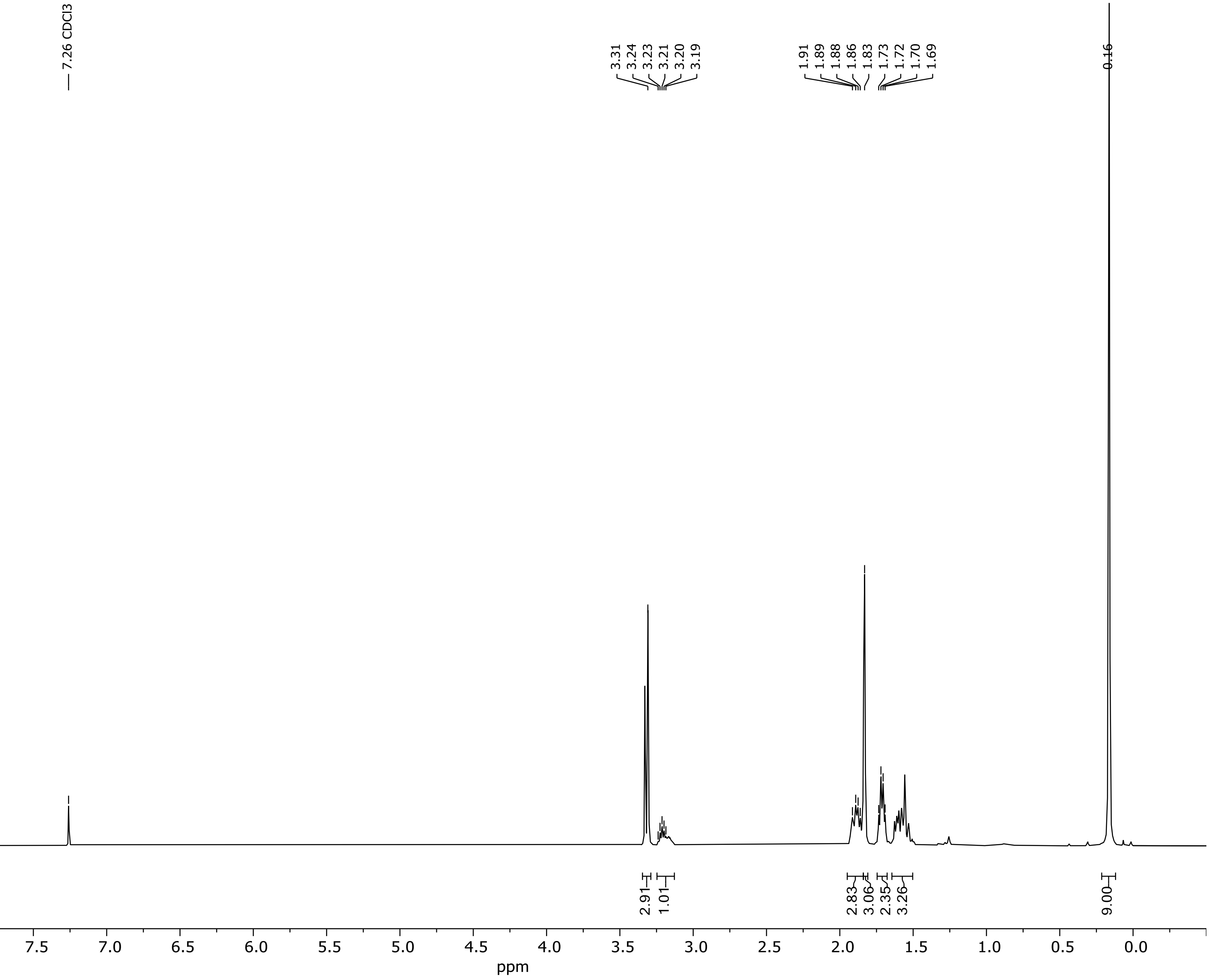

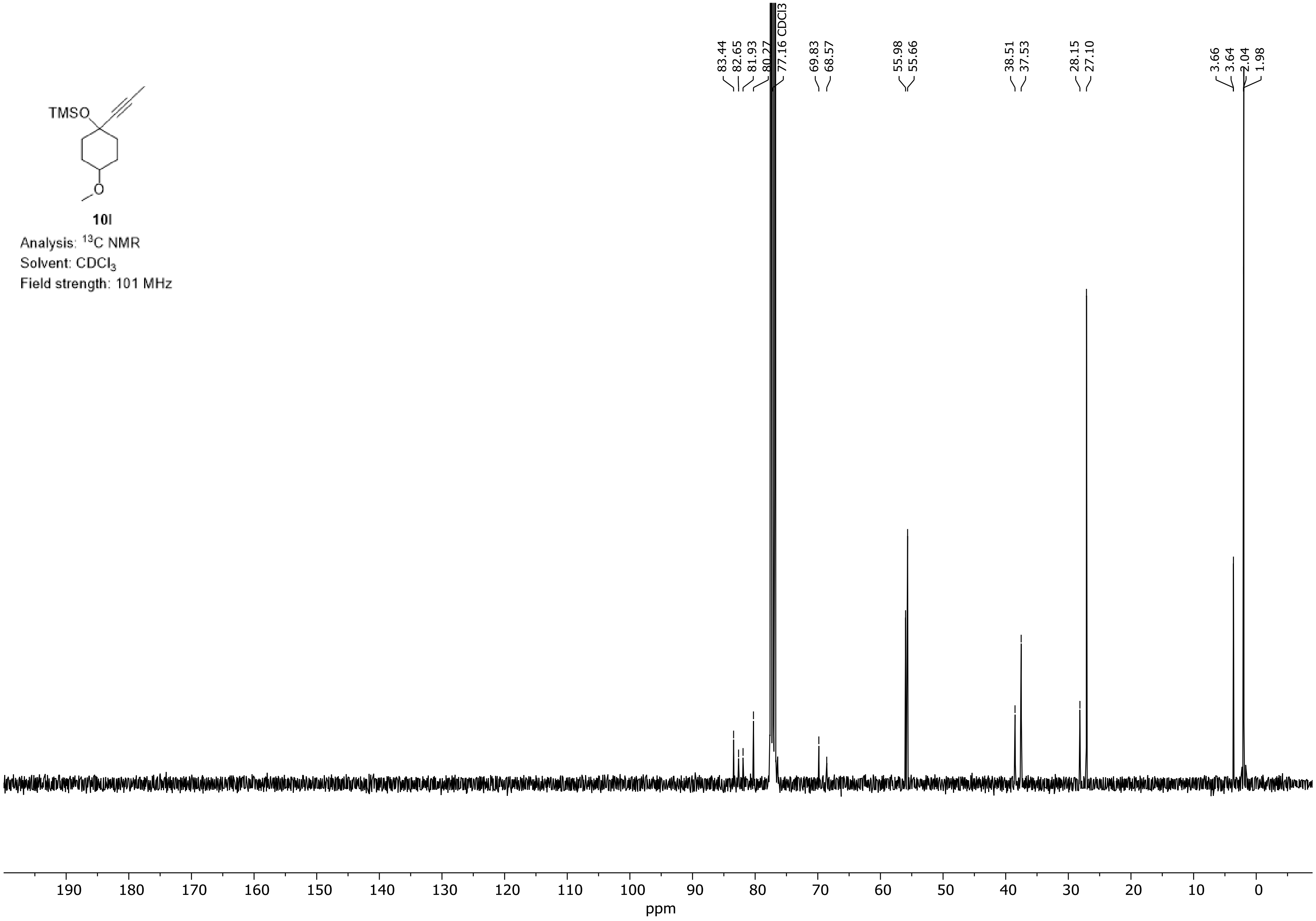

10l

Analysis: ^{13}C NMR

Solvent: $CDCl_3$

Field strength: 101 MHz

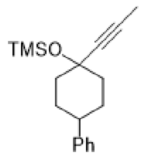

10m

Analysis: ^{1}H NMR
Solvent: $CDCl_3$
Field strength: 400 MHz

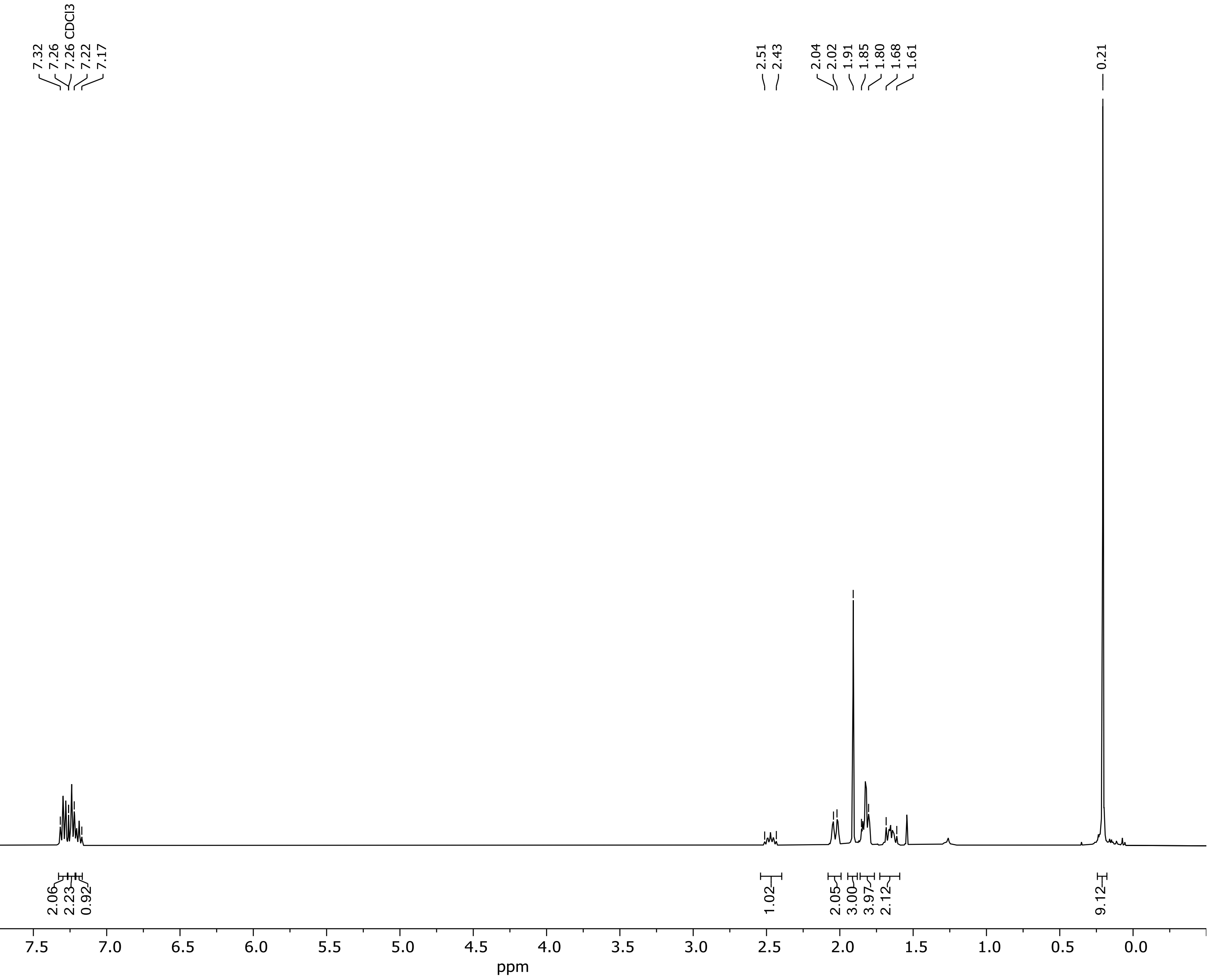

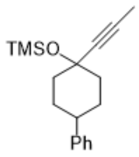

10m

Analysis: ^{13}C NMR
Solvent: $CDCl_3$
Field strength: 101 MHz

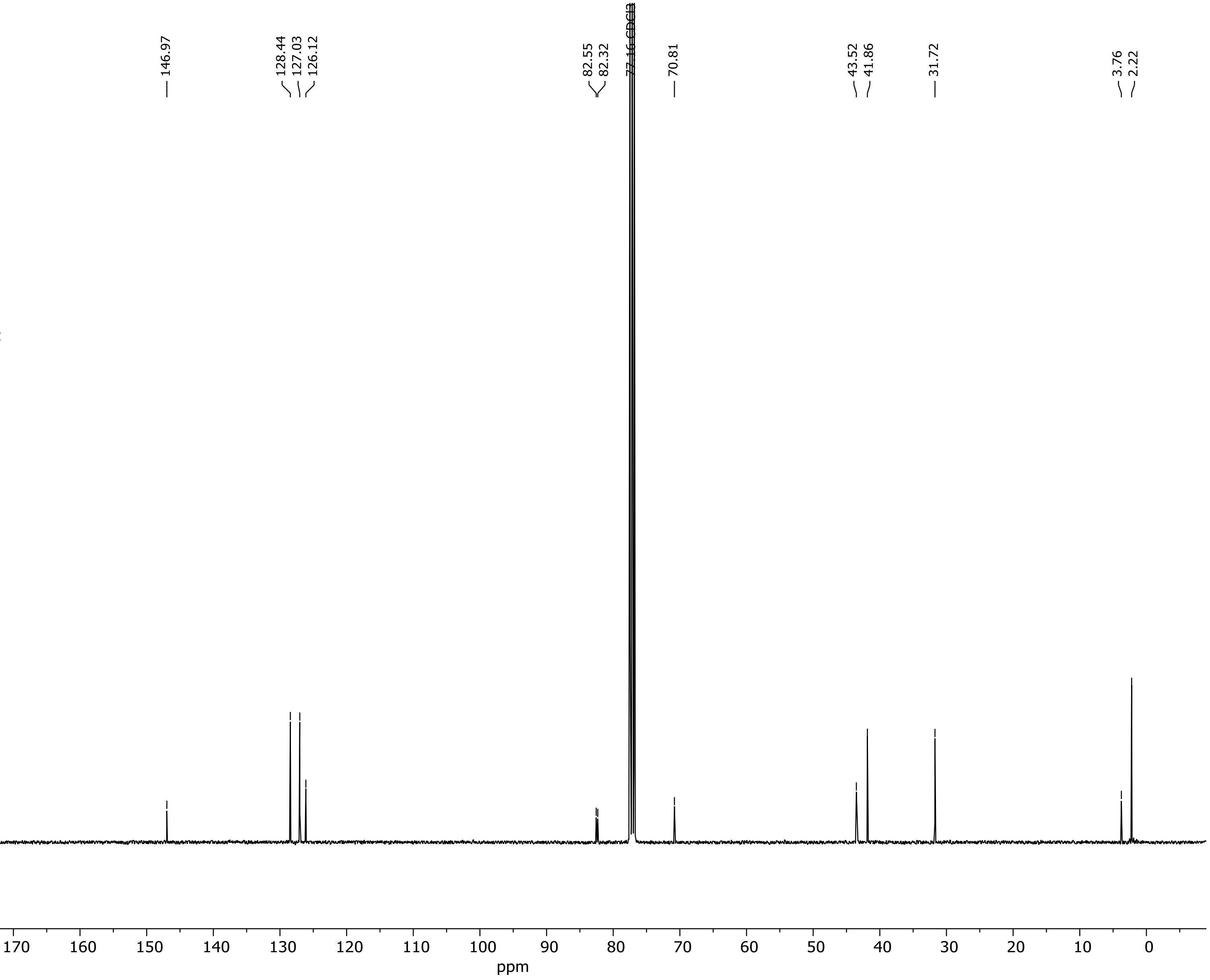

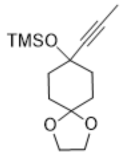

10n

Analysis: ^{1}H NMR

Solvent: $CDCl_3$

Field strength: 400 MHz

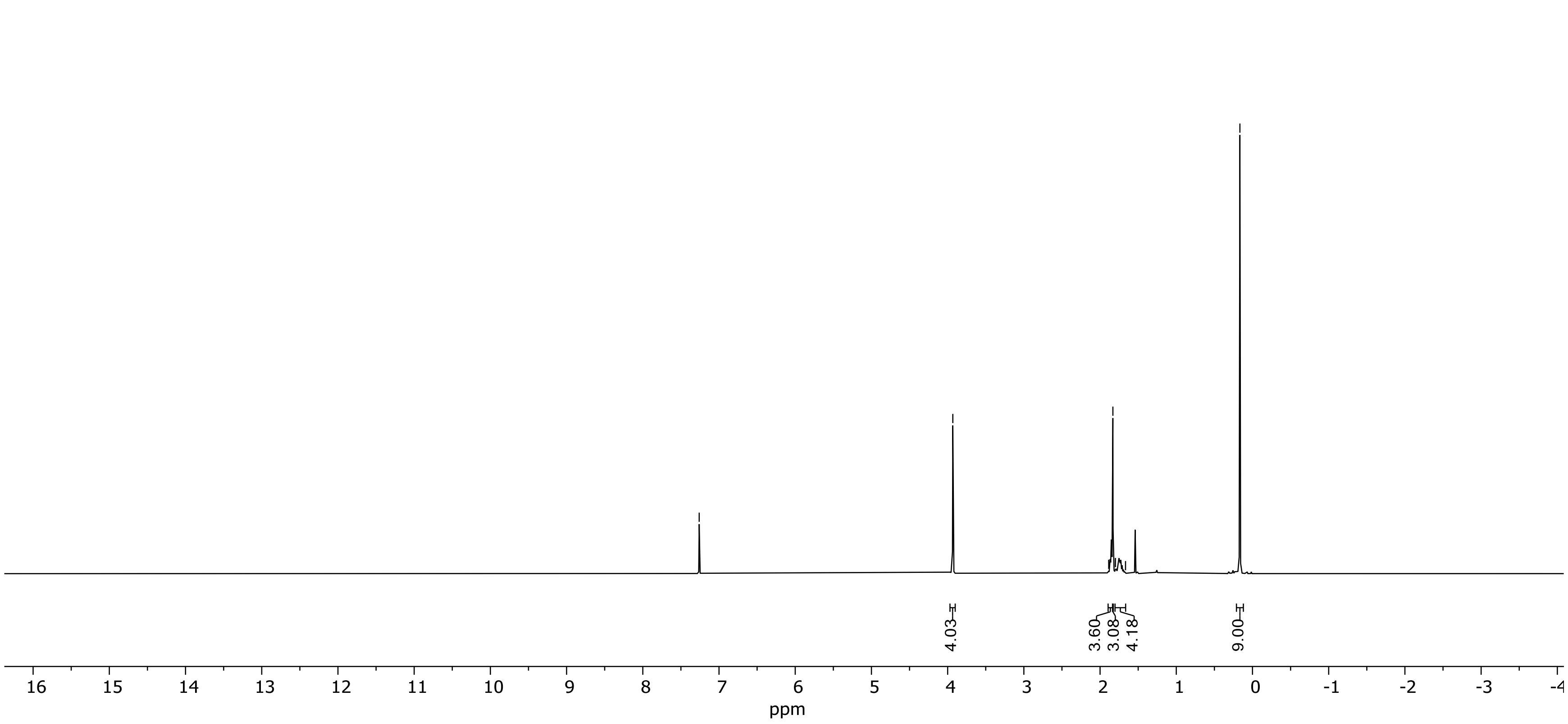

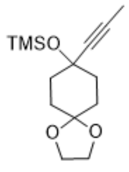

10n

Analysis: ^{13}C NMR
Solvent: $CDCl_3$
Field strength: 101 MHz

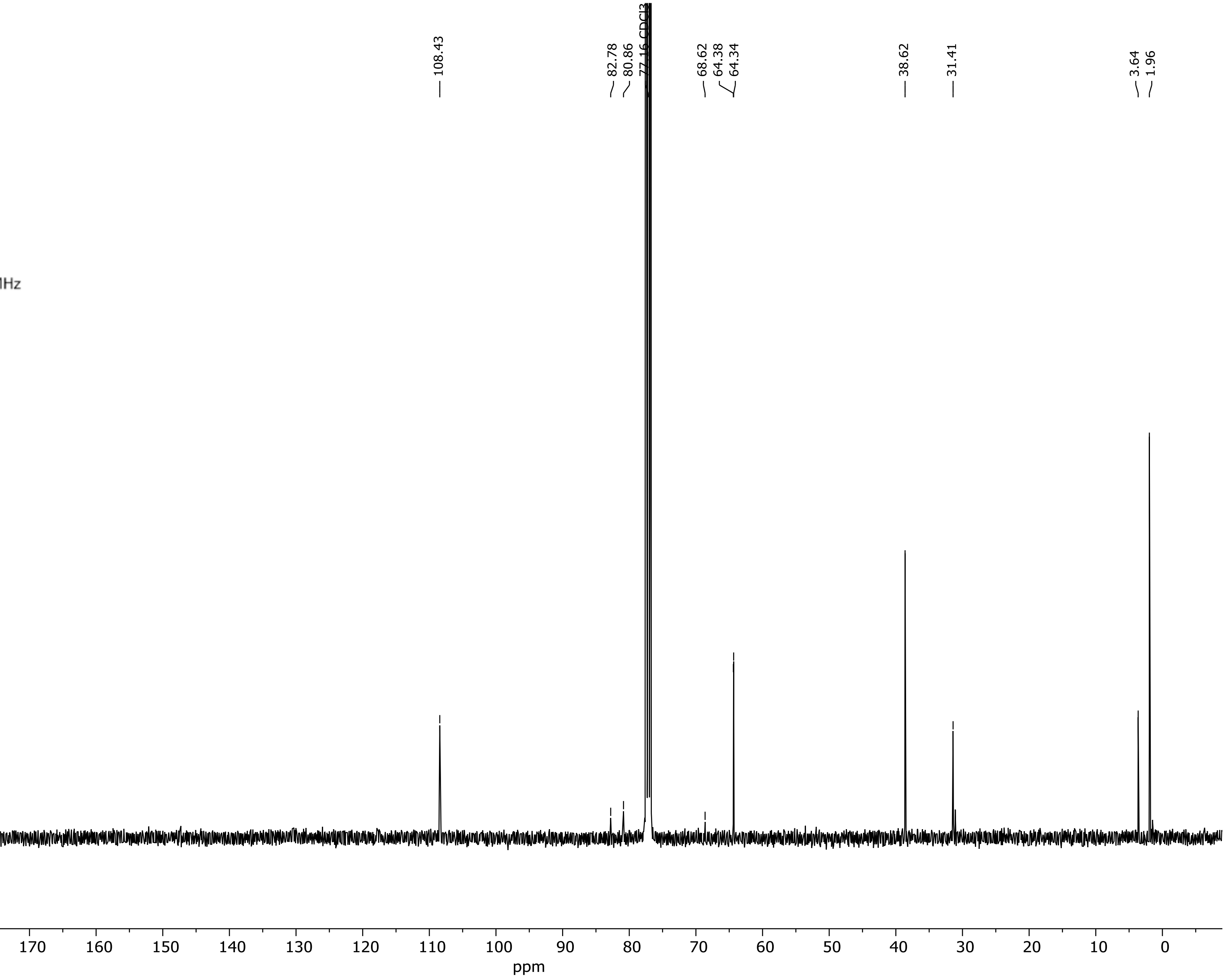

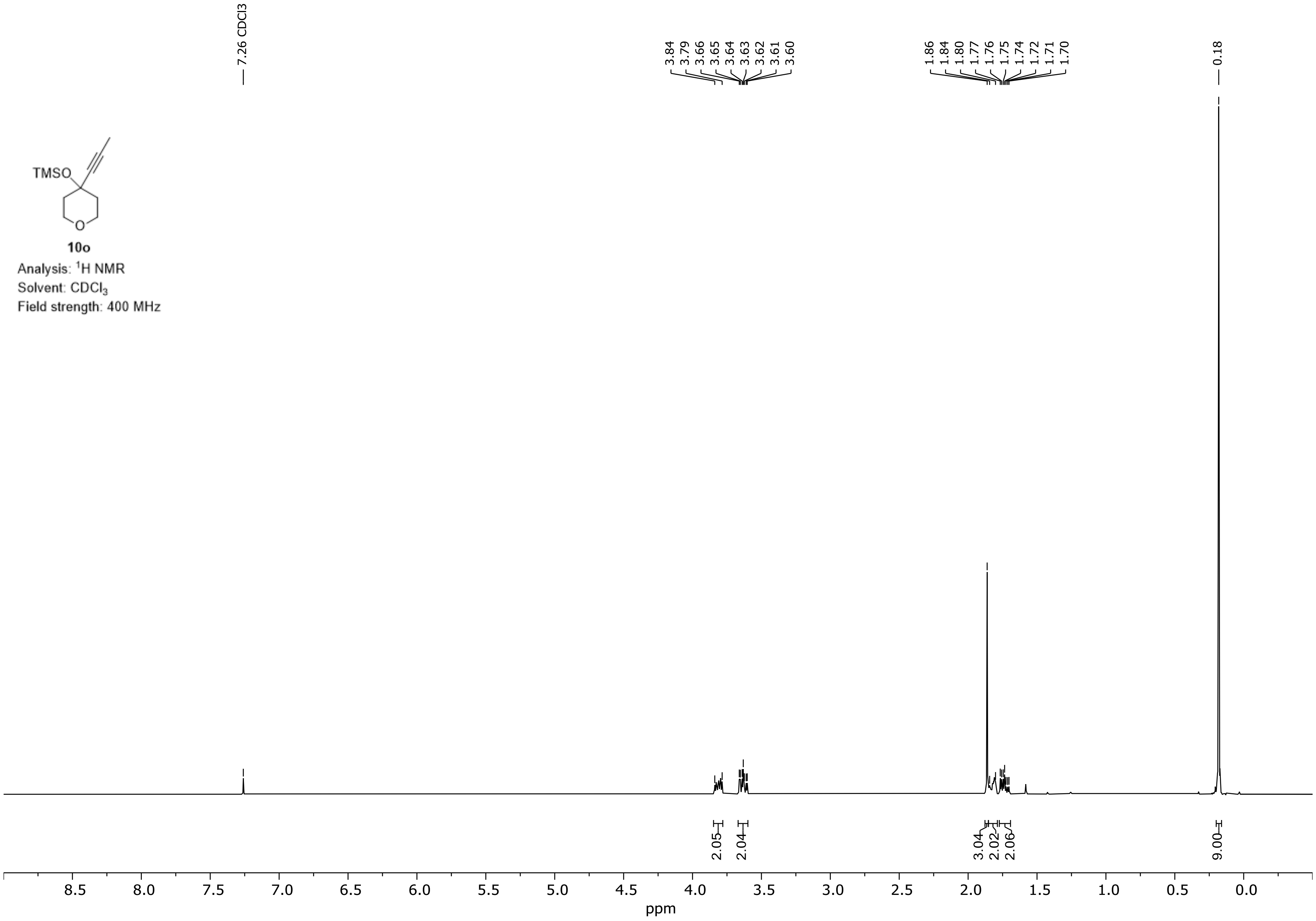
TMSO
O
10o
Analysis: ¹H NMR
Solvent: CDCl3
Field strength: 400 MHz
7.26 CDCl3
3.84
3.79
3.66
3.65
3.64
3.63
3.62
3.61
3.60
1.86
1.84
1.80
1.77
1.76
1.75
1.74
1.72
1.71
1.70
0.18
2.05
2.04
3.04
2.02
2.06
9.00
8.5
8.0
7.5
7.0
6.5
6.0
5.5
5.0
4.5
4.0
3.5
3.0
2.5
2.0
1.5
1.0
0.5
0.0
ppm

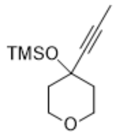

10o

Analysis: ^{13}C NMR
Solvent: $CDCl_3$
Field strength: 101 MHz

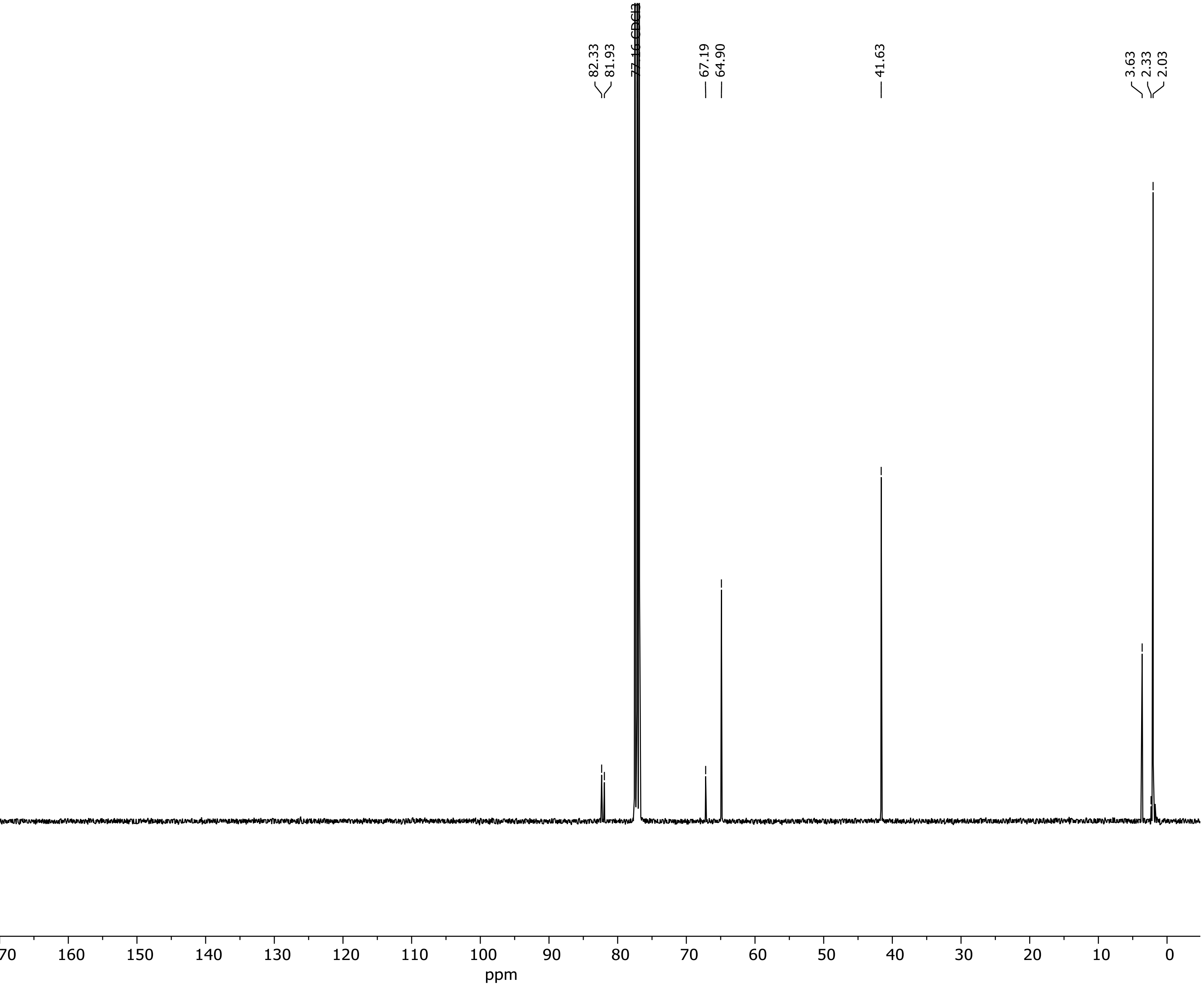

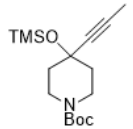

10p

Analysis: ^{1}H NMR

Solvent: $CDCl_3$

Field strength: 400 MHz

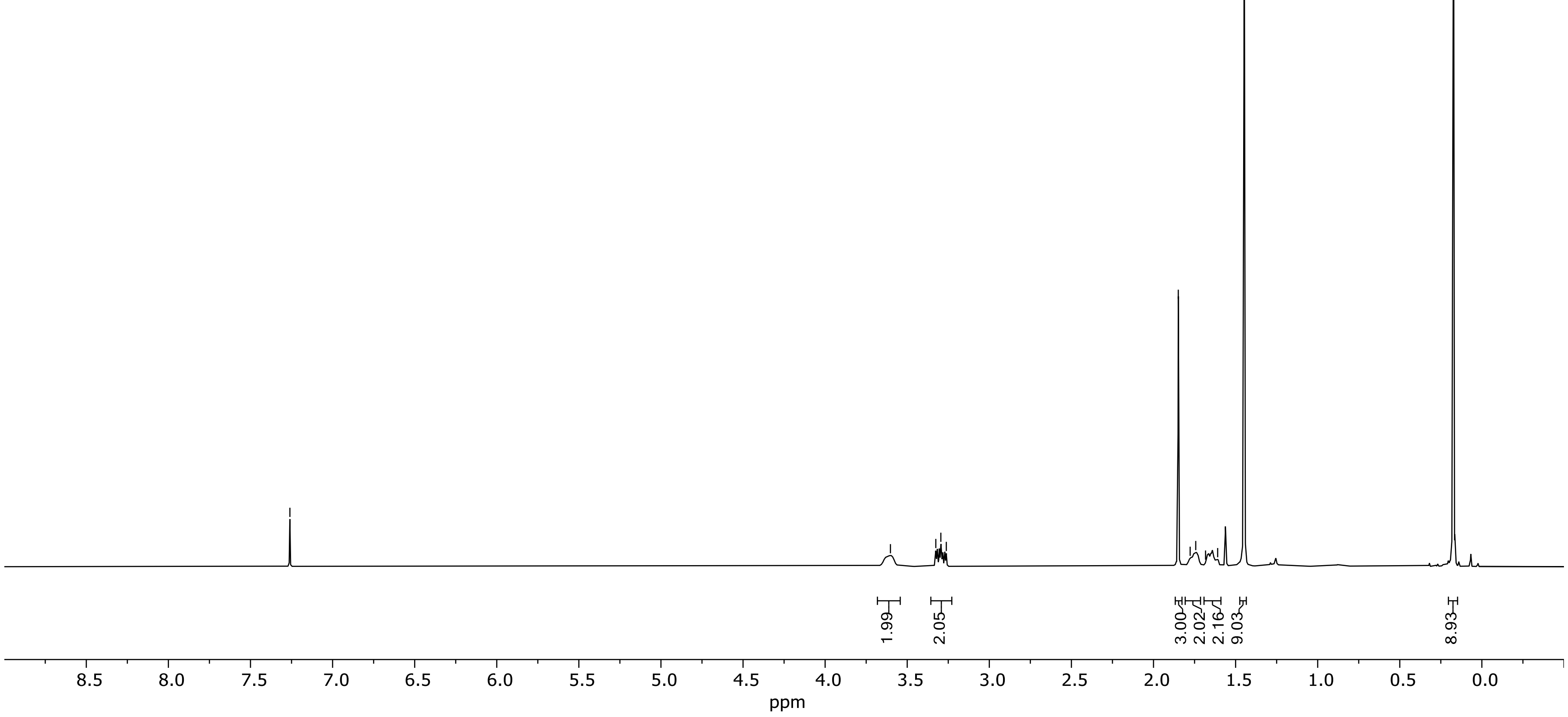

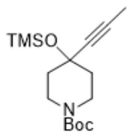

10p

Analysis: ^{13}C NMR
Solvent: $CDCl_3$
Field strength: 101 MHz

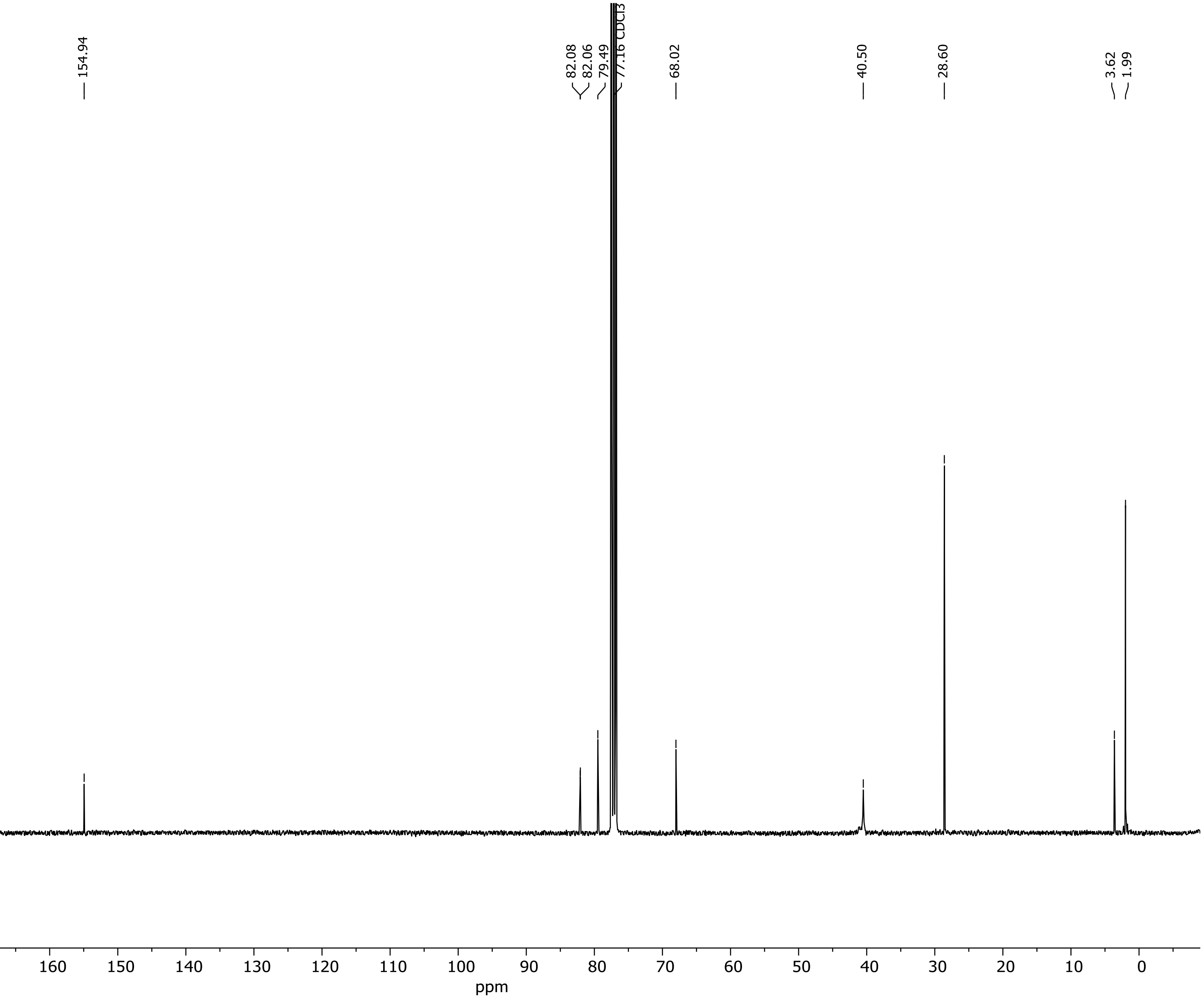

10q

Analysis: ^{1}H NMR

Solvent: $CDCl_3$

Field strength: 400 MHz

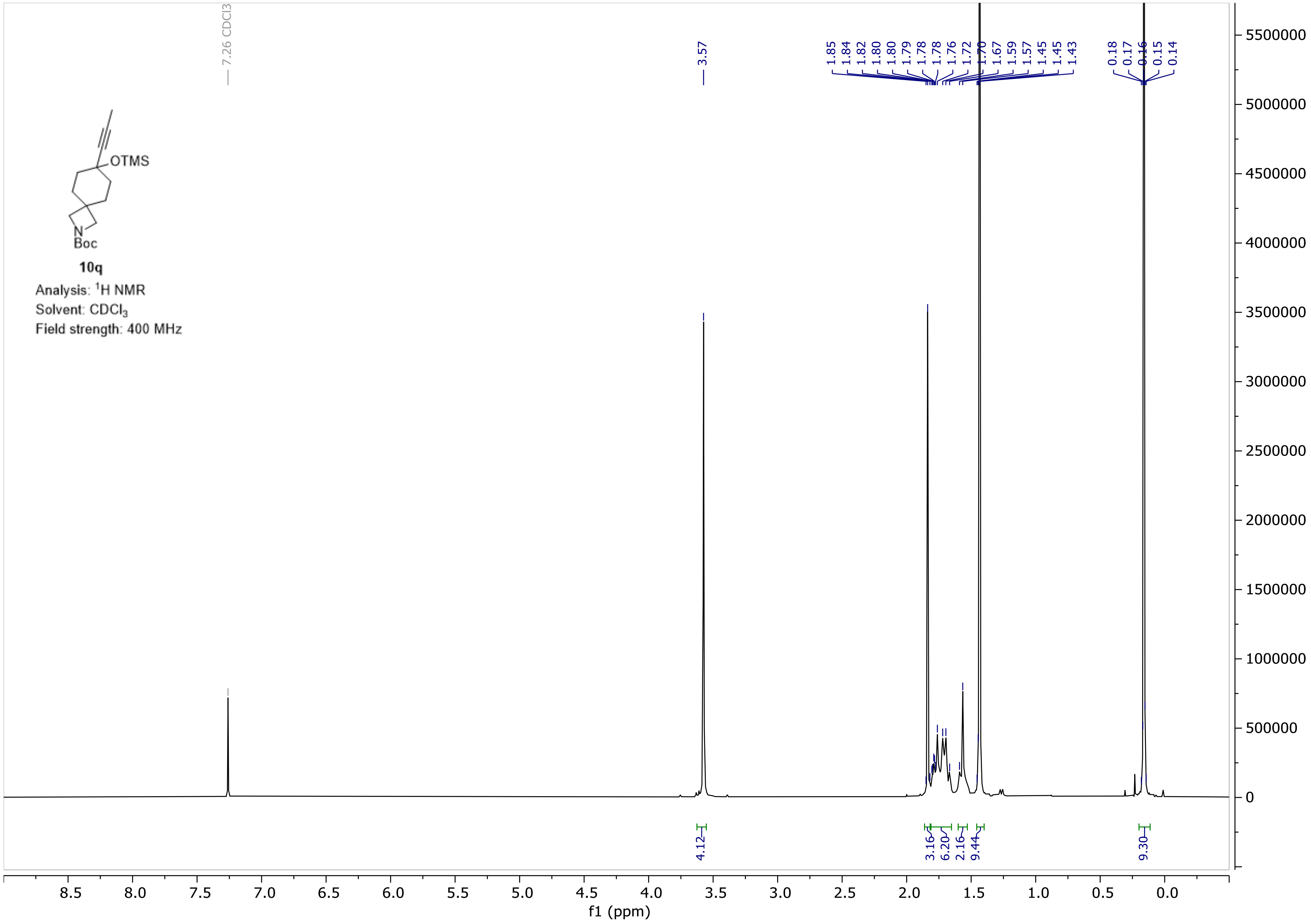

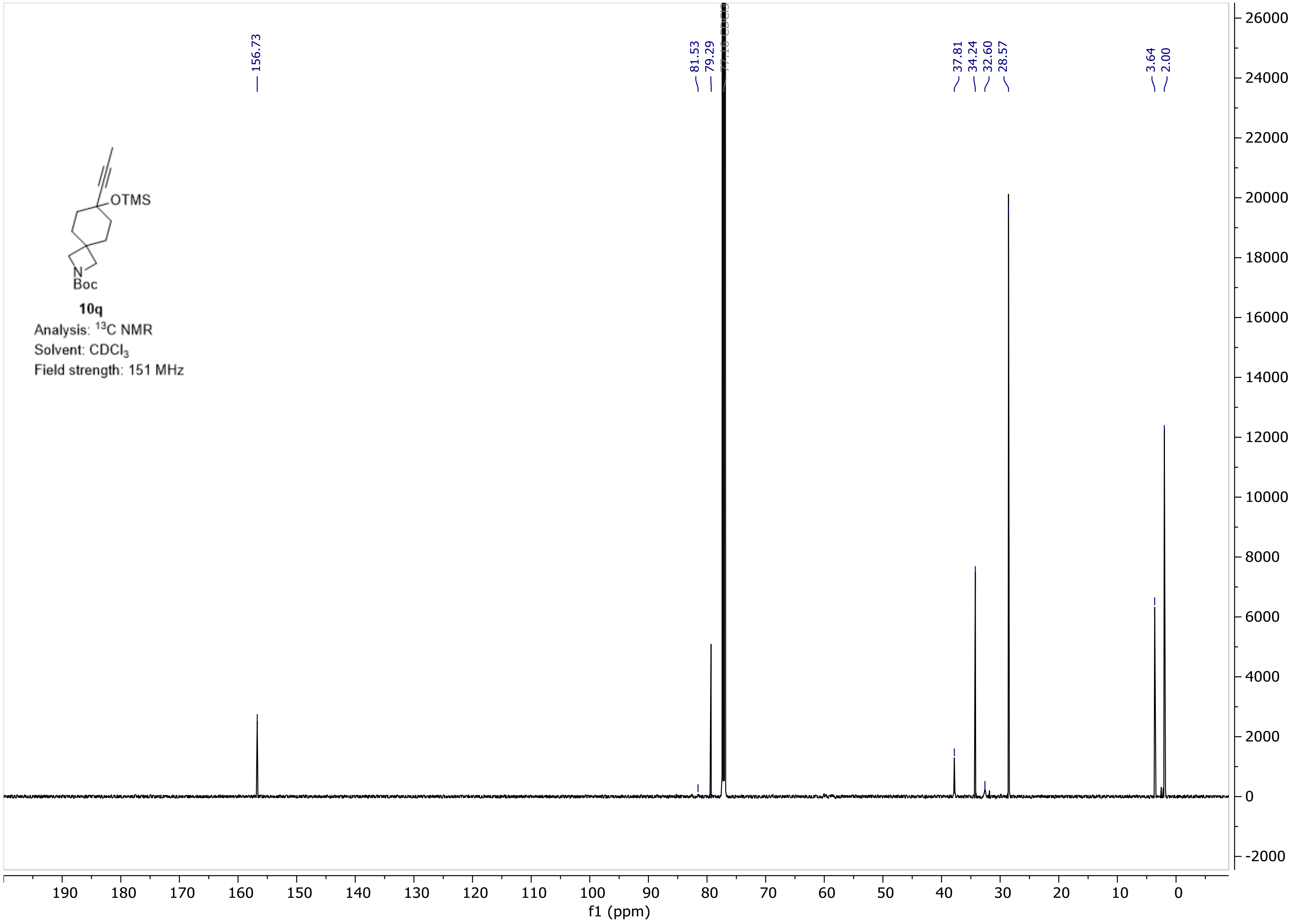

10q
Analysis: ^{13}C NMR
Solvent: $CDCl_3$
Field strength: 151 MHz

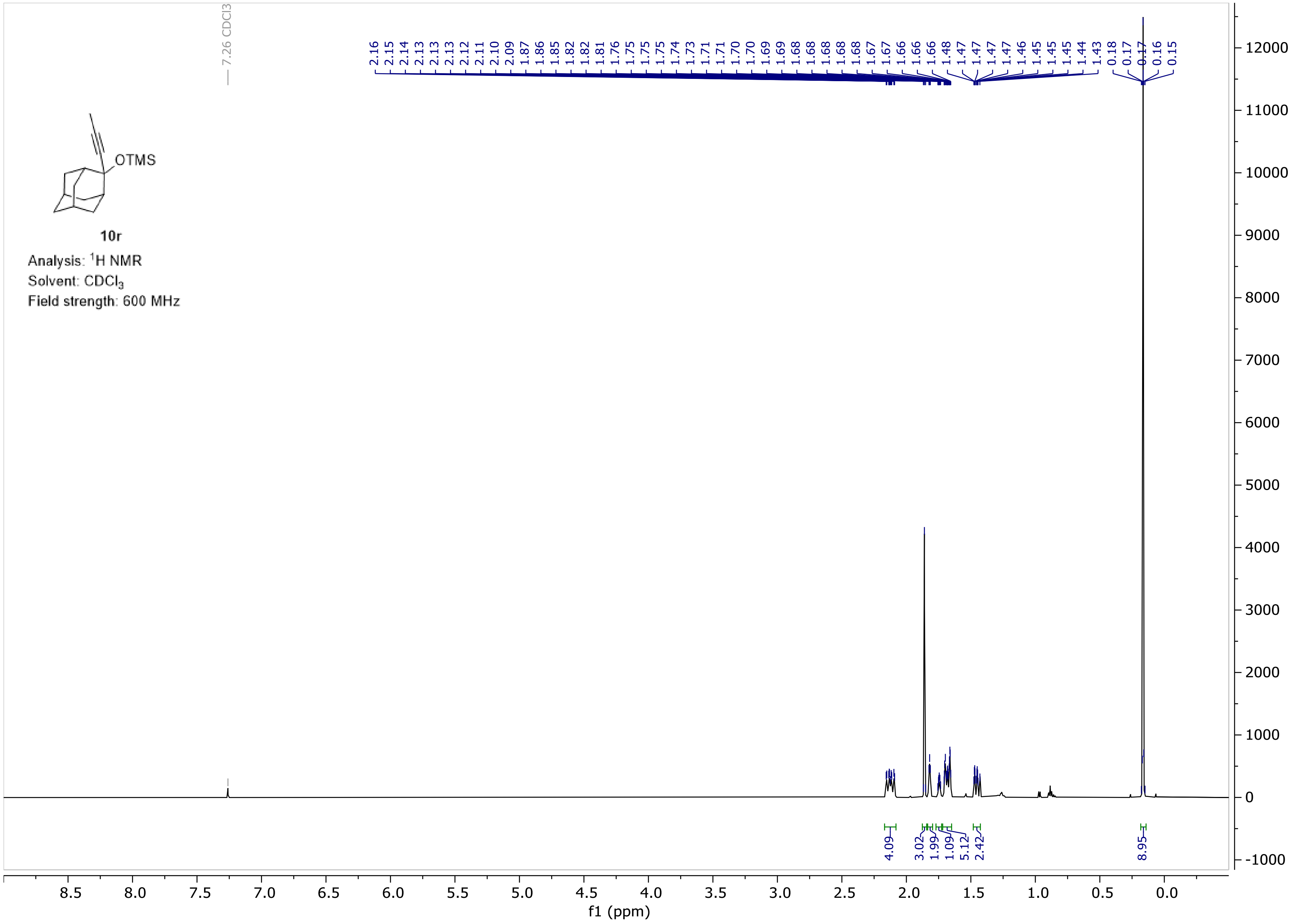

OTMS
10r
Analysis: ¹H NMR
Solvent: CDCl3
Field strength: 600 MHz
7.26 CDCl3
2.16 2.15 2.14 2.13 2.13 2.13 2.12 2.11 2.10 2.09 1.87 1.86 1.85 1.82 1.82 1.81 1.76 1.75 1.75 1.75 1.74 1.73 1.71 1.71 1.70 1.69 1.68 1.68 1.68 1.68 1.67 1.67 1.66 1.66 1.66 1.48 1.47 1.47 1.47 1.46 1.45 1.45 1.45 1.44 1.43 0.18 0.17 0.17 0.16 0.15
4.09 3.02 1.99 1.09 5.12 2.42 8.95
f1 (ppm)

10r

Analysis: ^{13}C NMR
Solvent: $CDCl_3$
Field strength: 151 MHz

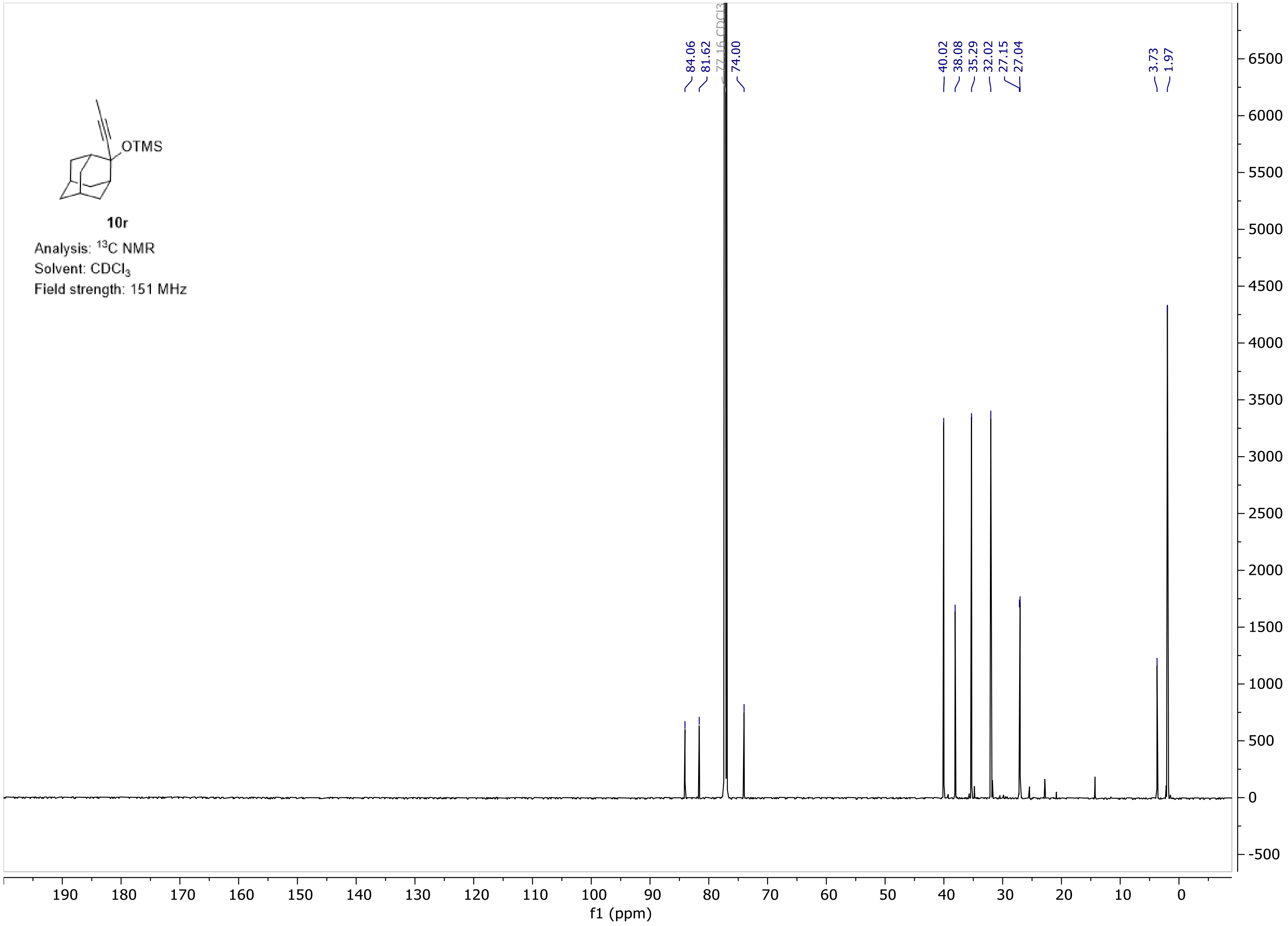

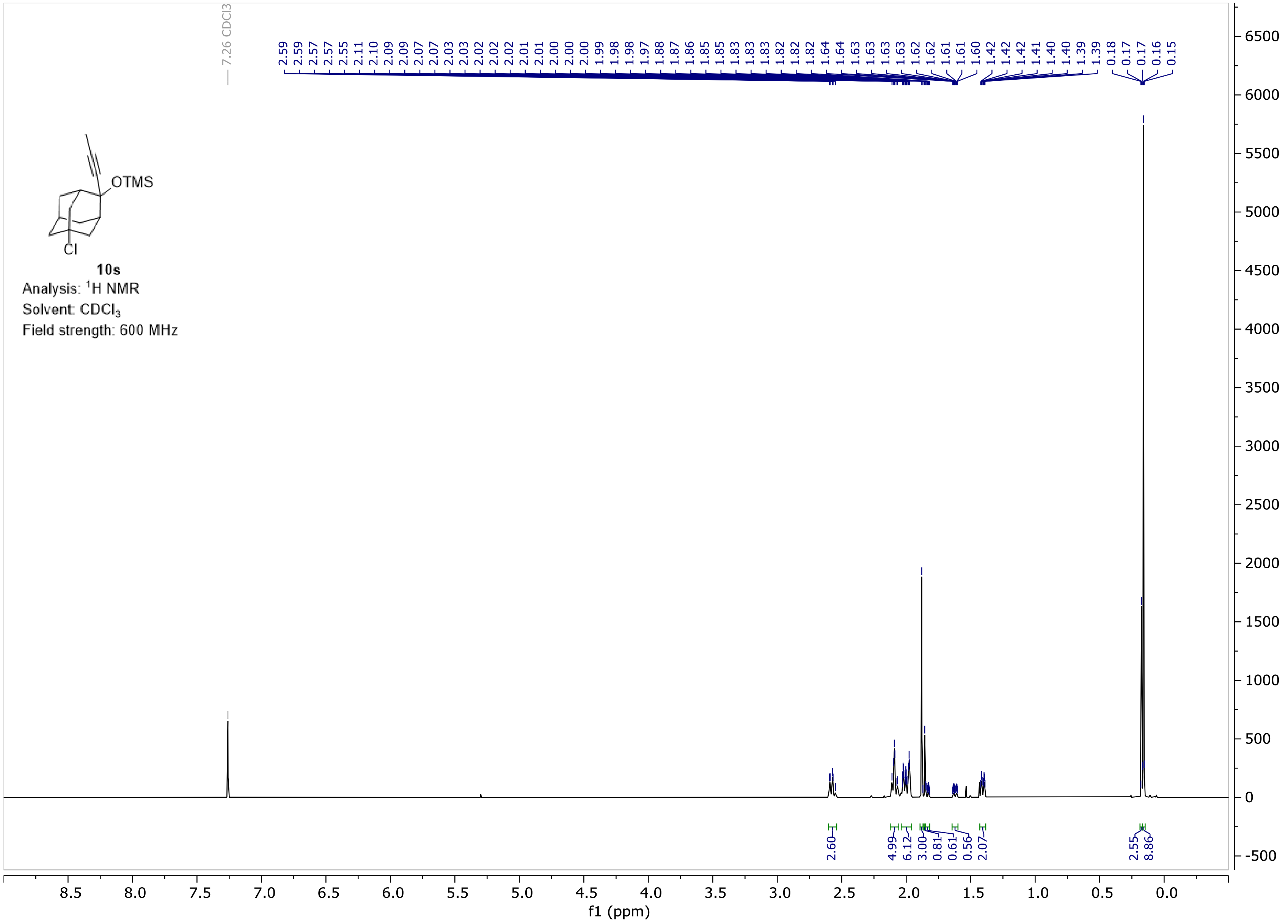

7.26 CDCl3
2.59
2.59
2.57
2.57
2.55
2.11
2.10
2.09
2.09
2.07
2.07
2.03
2.03
2.02
2.02
2.02
2.01
2.01
2.00
2.00
1.99
1.98
1.98
1.97
1.88
1.87
1.86
1.85
1.85
1.83
1.83
1.83
1.82
1.82
1.82
1.64
1.64
1.63
1.63
1.63
1.63
1.62
1.62
1.61
1.61
1.61
1.60
1.42
1.42
1.42
1.41
1.40
1.40
1.39
1.39
0.18
0.17
0.17
0.16
0.15
OTMS
Cl
10s
Analysis: ¹H NMR
Solvent: CDCl3
Field strength: 600 MHz
2.60
4.99
6.12
3.00
0.81
0.61
0.56
2.07
2.55
8.86
f1 (ppm)

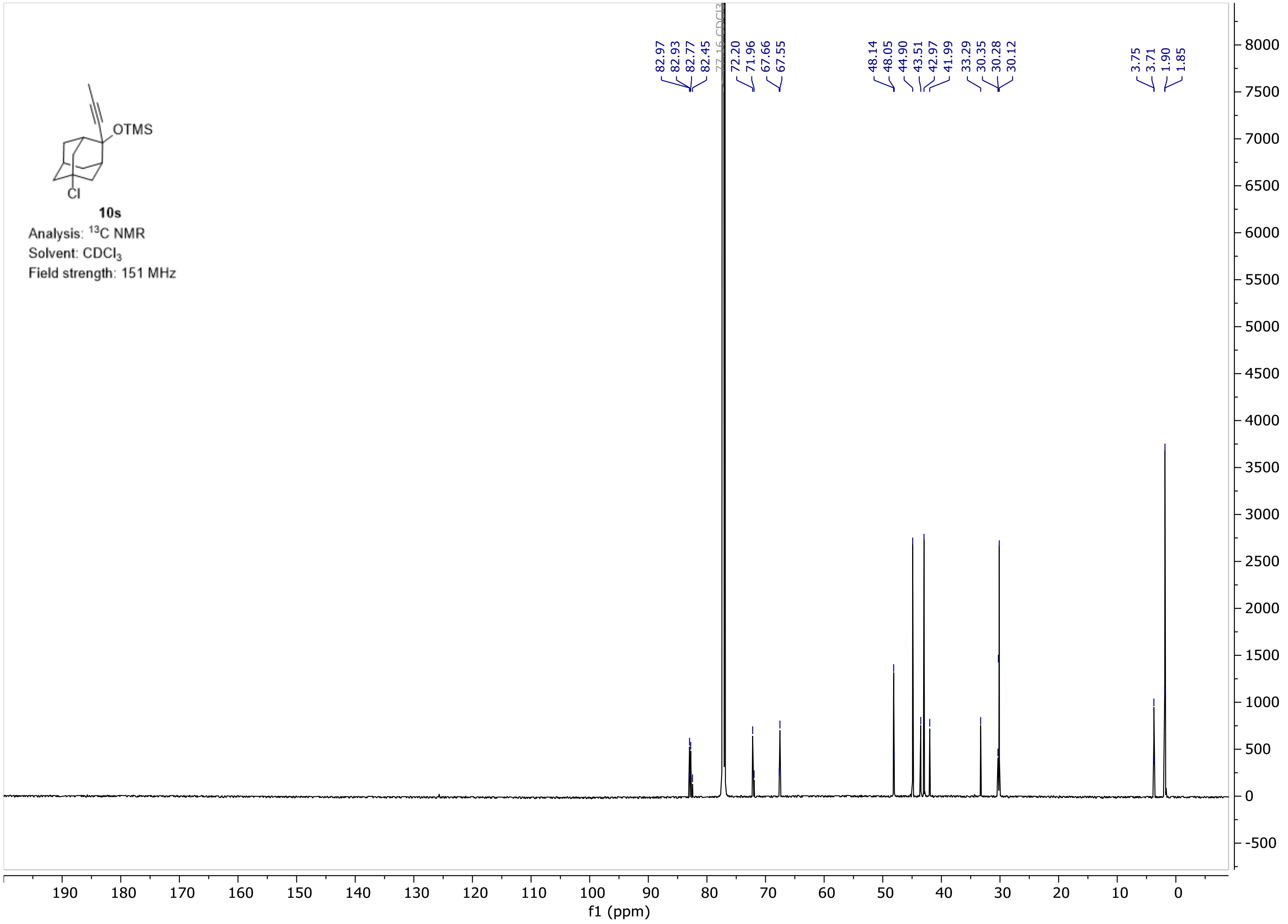
82.97
82.93
82.77
82.45
77.16 CDCl3
72.20
71.96
67.66
67.55
48.14
48.05
44.90
43.51
42.97
41.99
33.29
30.35
30.28
30.12
3.75
3.71
1.90
1.85
OTMS
Cl
10s
Analysis: 13C NMR
Solvent: CDCl3
Field strength: 151 MHz
f1 (ppm)

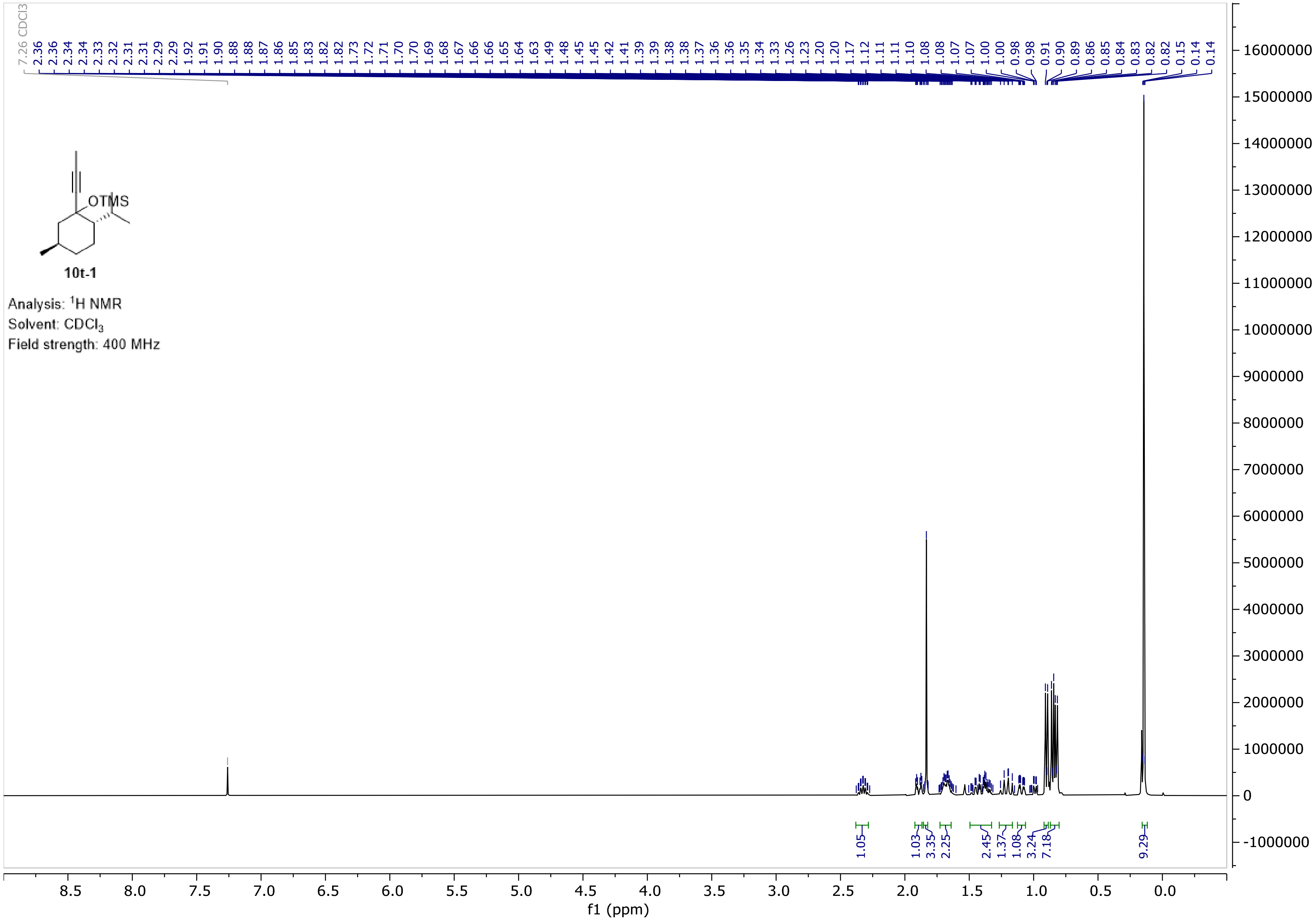

OTMS
10t-1
Analysis: ¹H NMR
Solvent: CDCl3
Field strength: 400 MHz
f1 (ppm)

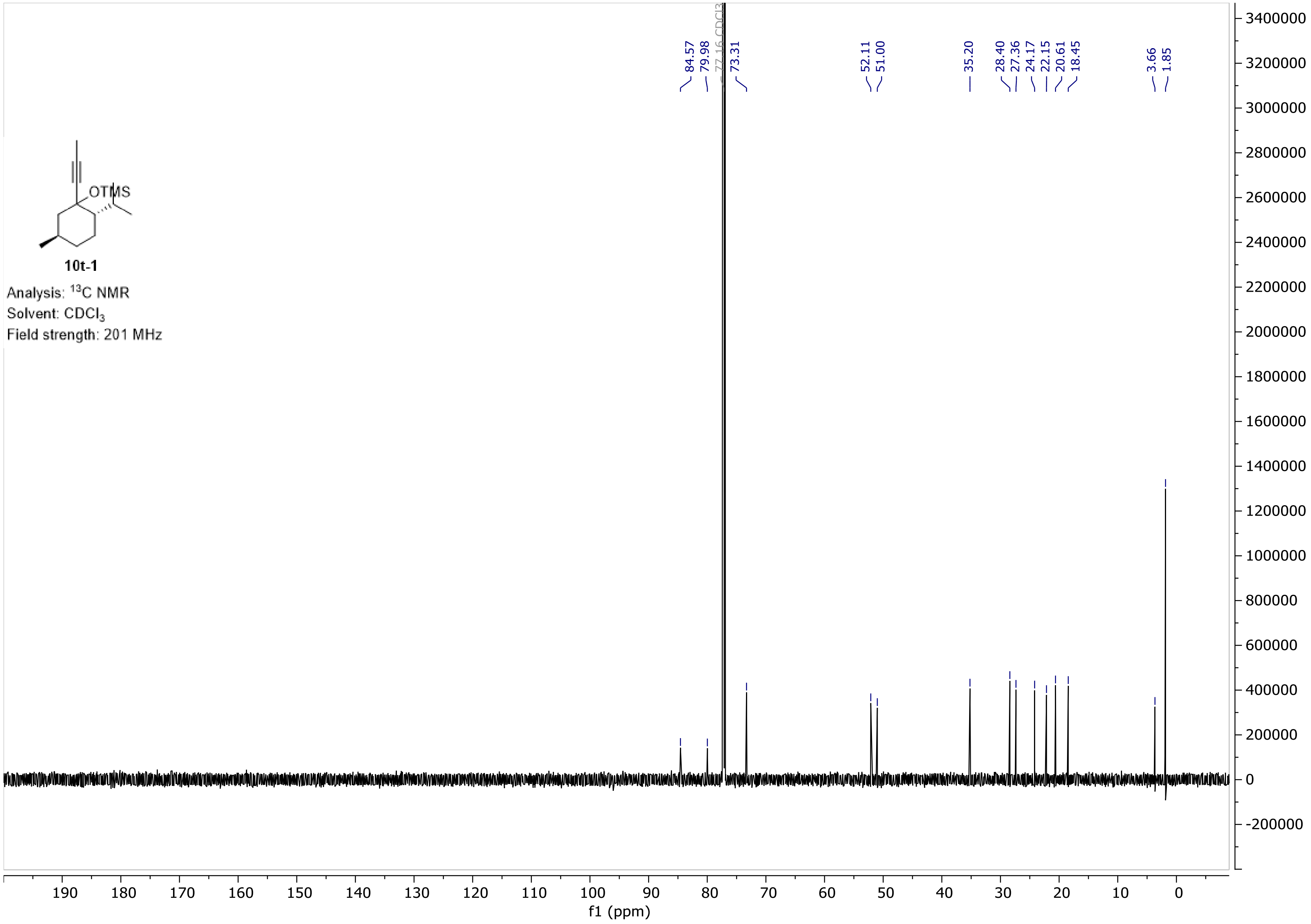

10t-1

Analysis: ^{13}C NMR

Solvent: $CDCl_3$

Field strength: 201 MHz

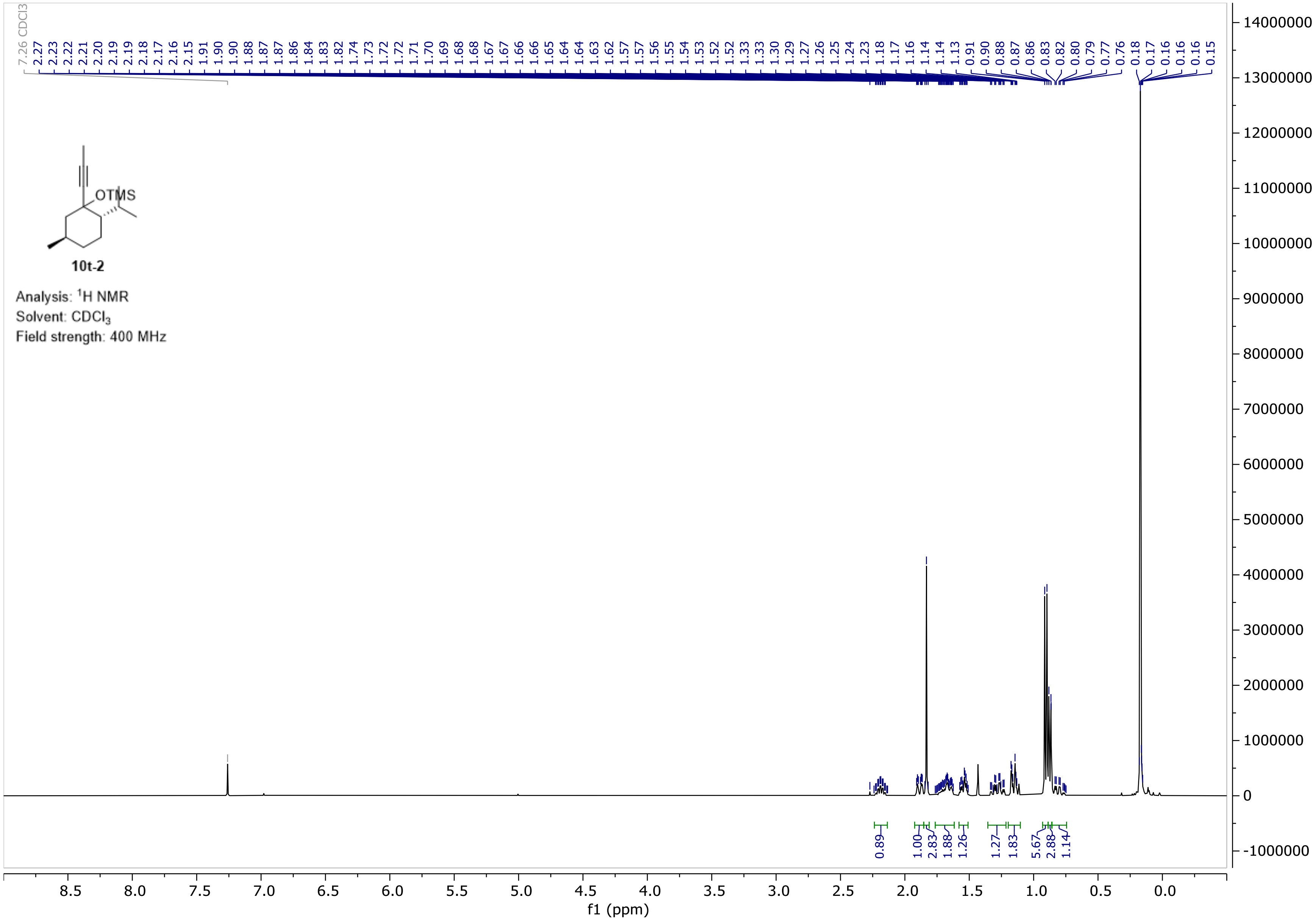
7.26 CDCl3
2.27 2.23 2.22 2.21 2.20 2.19 2.19 2.18 2.17 2.16 2.15 1.91 1.90 1.90 1.88 1.87 1.87 1.86 1.84 1.83 1.82 1.74 1.73 1.72 1.72 1.71 1.70 1.69 1.68 1.68 1.67 1.67 1.66 1.66 1.65 1.64 1.64 1.63 1.62 1.57 1.57 1.56 1.55 1.54 1.53 1.52 1.52 1.33 1.30 1.29 1.27 1.26 1.25 1.24 1.23 1.18 1.17 1.16 1.14 1.14 1.13 0.91 0.90 0.88 0.87 0.86 0.83 0.82 0.80 0.79 0.77 0.76 0.18 0.17 0.16 0.16 0.16 0.15
OTMS
10t-2
Analysis: ^{1}H NMR
Solvent: $CDCl_3$
Field strength: 400 MHz
0.89 1.00 2.83 1.88 1.26 1.27 1.83 5.67 2.88 1.14
f1 (ppm)

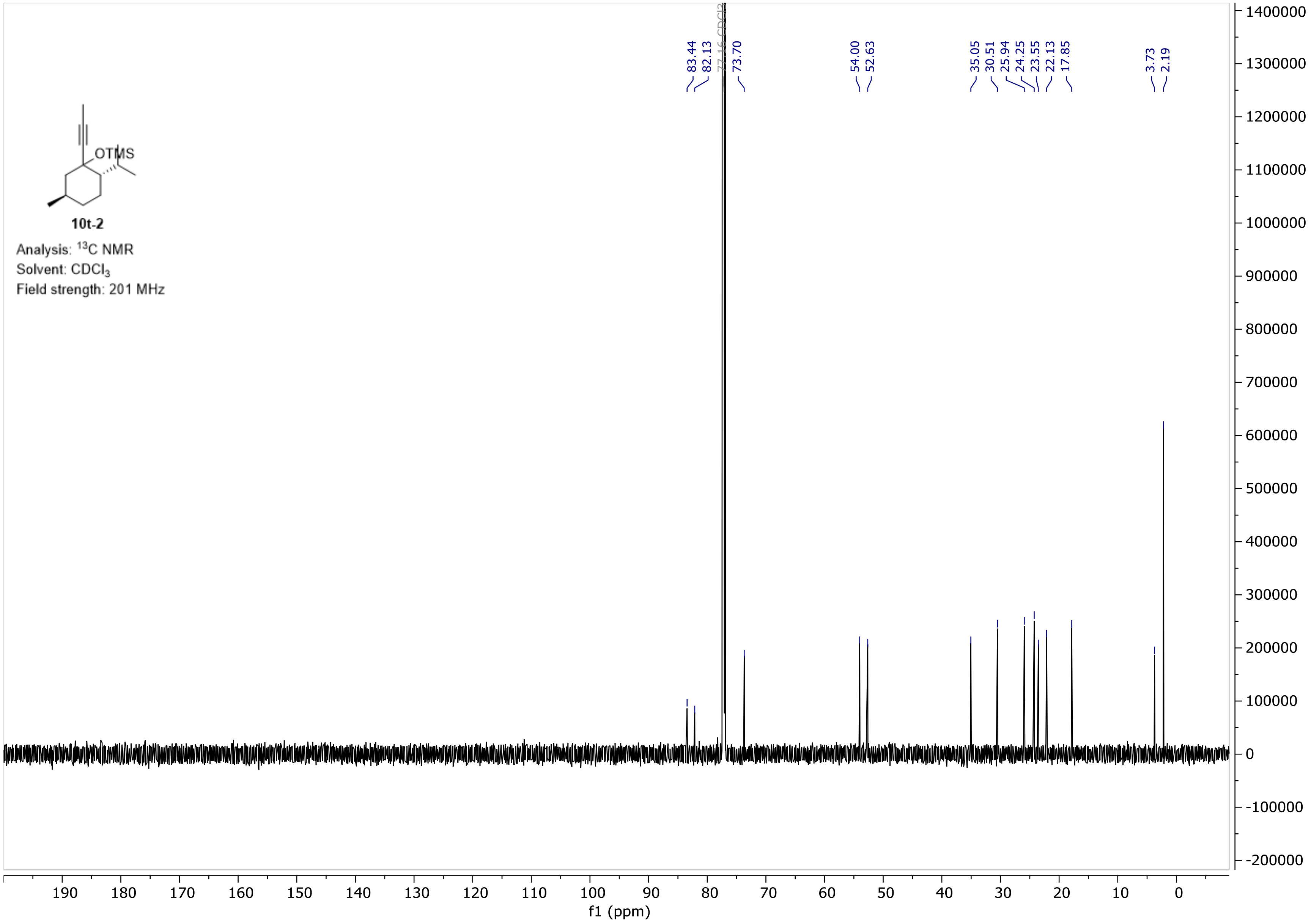

10t-2

Analysis: ^{13}C NMR

Solvent: $CDCl_3$

Field strength: 201 MHz

10u-1
Analysis: ^{1}H NMR
Solvent: $CDCl_3$
Field strength: 600 MHz

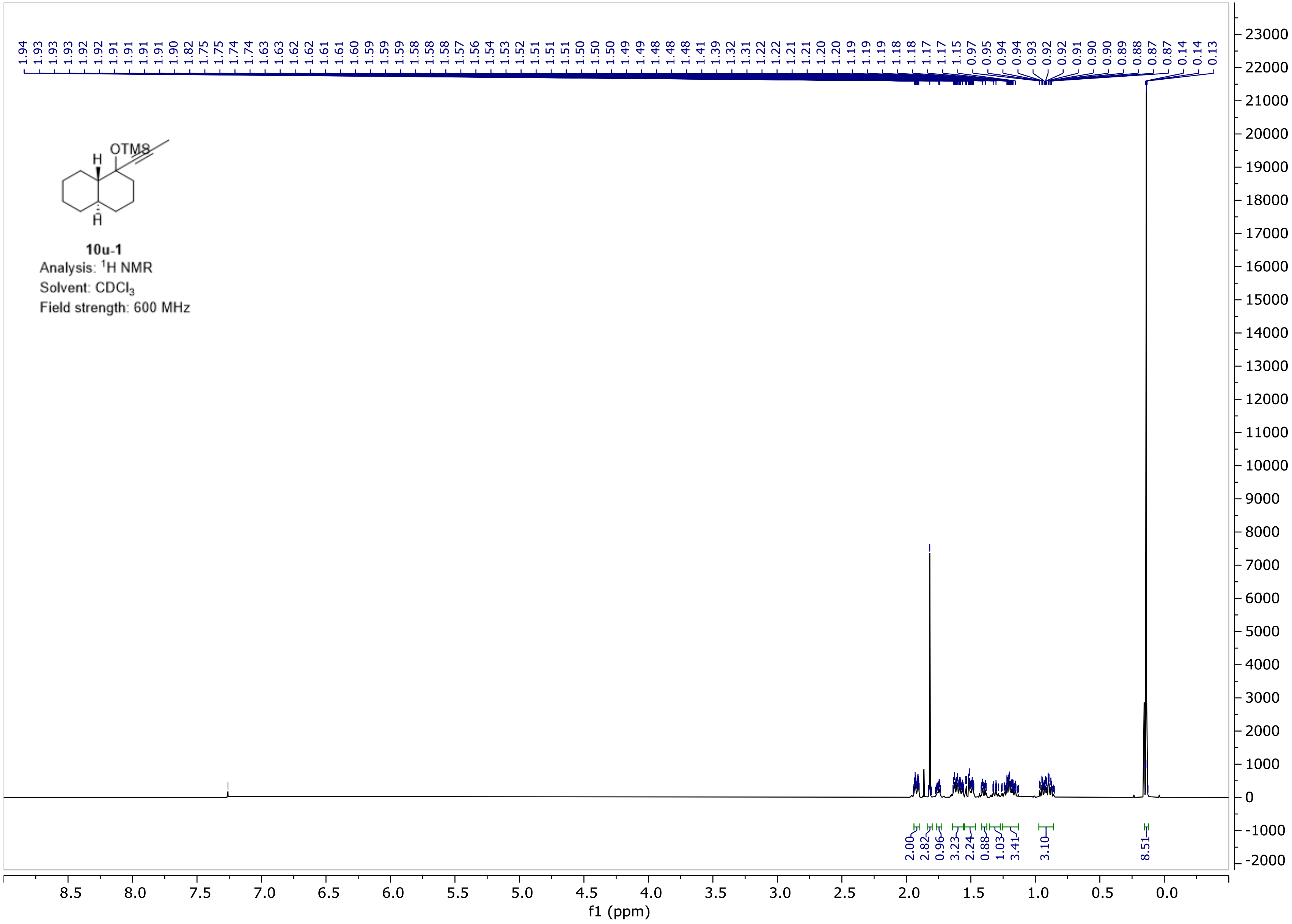

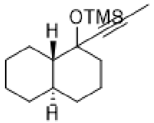

10u-1
Analysis: ^{13}C NMR
Solvent: $CDCl_3$
Field strength: 151 MHz

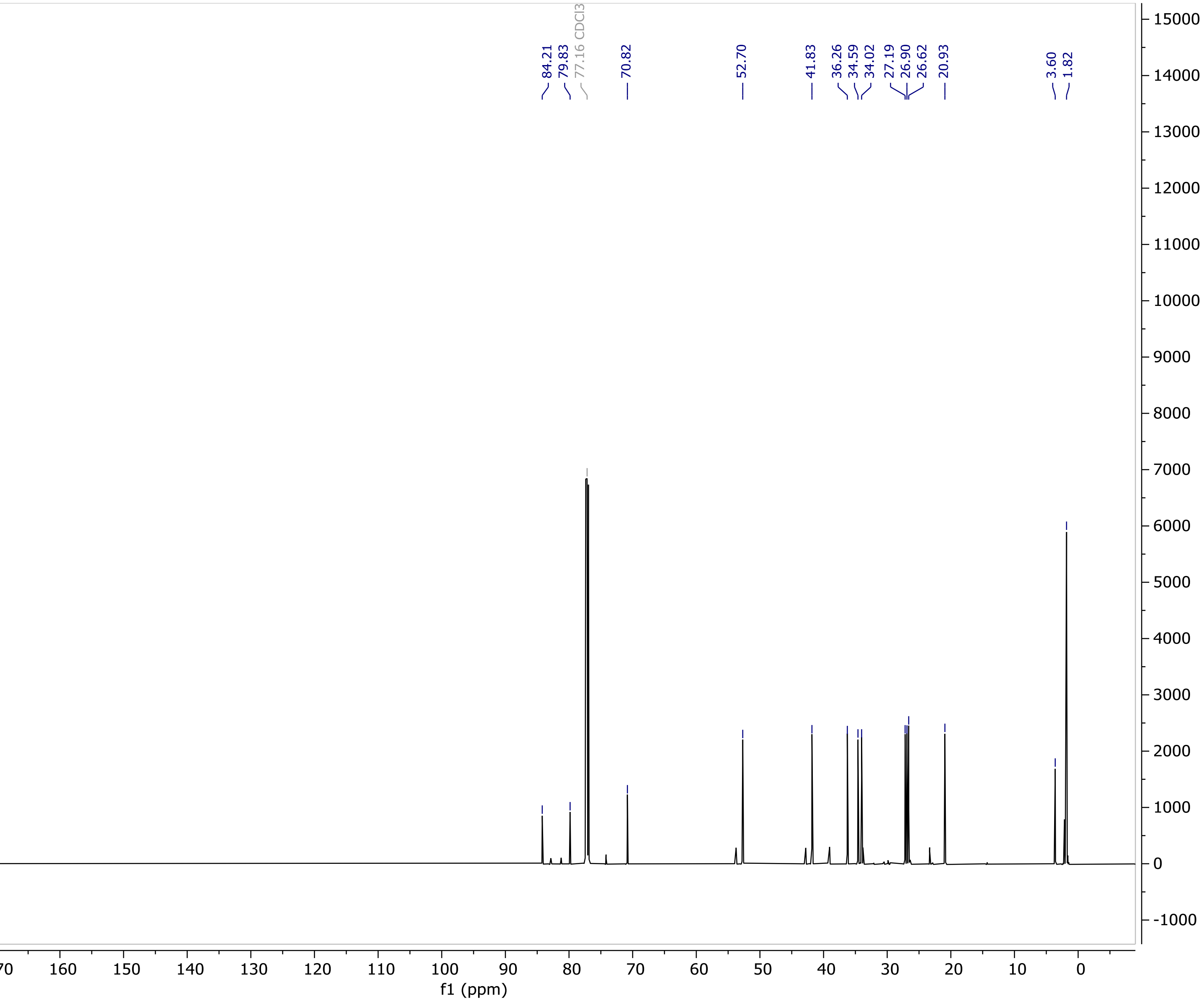

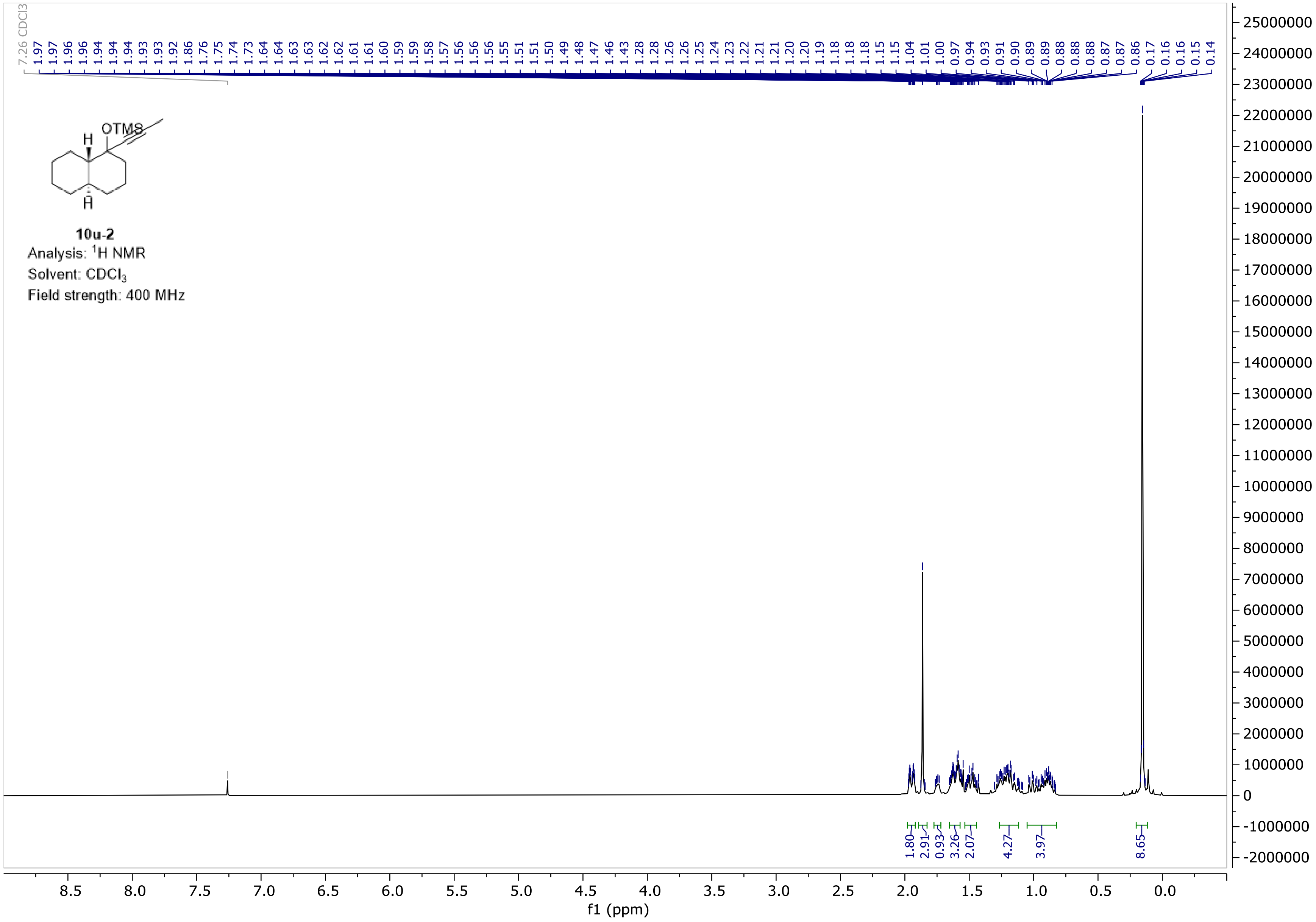
OTMS
H
H
10u-2
Analysis: ¹H NMR
Solvent: CDCl3
Field strength: 400 MHz
f1 (ppm)

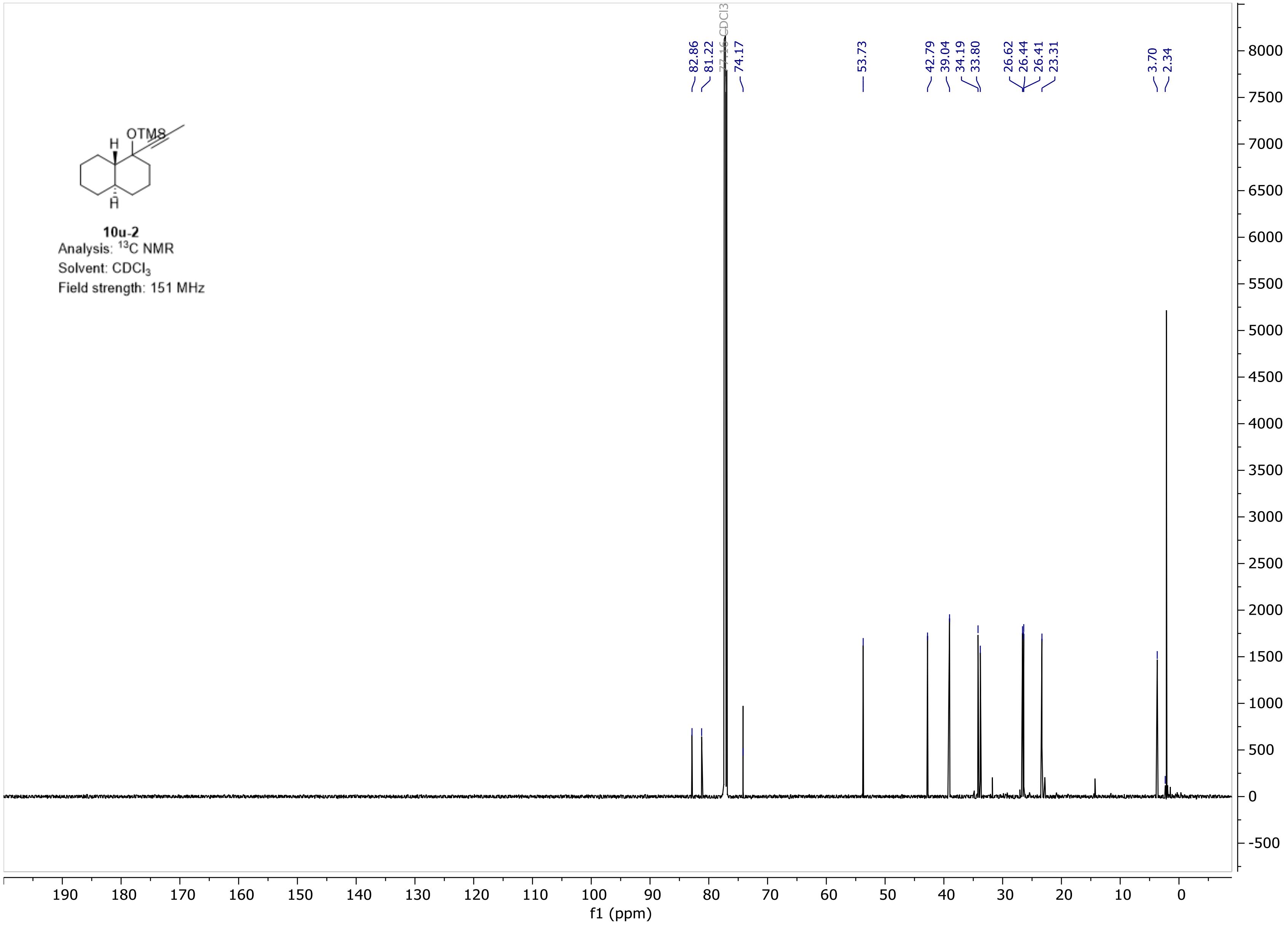
OTMS
10u-2
Analysis: ^{13}C NMR
Solvent: $CDCl_3$
Field strength: 151 MHz
82.86
81.22
77.16 CDCl3
74.17
53.73
42.79
39.04
34.19
33.80
26.62
26.44
26.41
23.31
3.70
2.34
f1 (ppm)

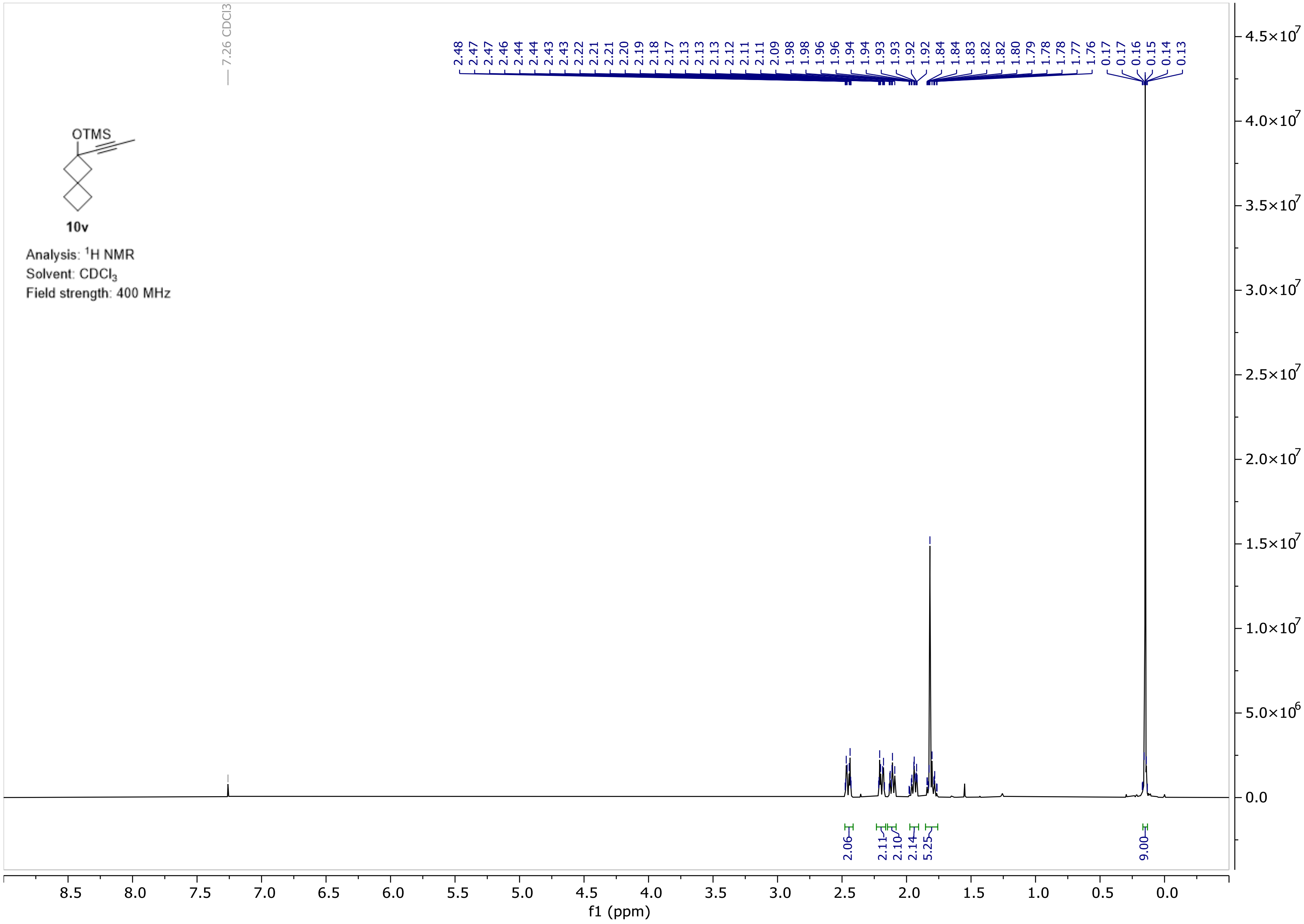
7.26 CDCl3
2.48 2.47 2.47 2.46 2.44 2.44 2.43 2.43 2.22 2.21 2.21 2.20 2.19 2.18 2.17 2.13 2.13 2.13 2.12 2.11 2.11 2.09 1.98 1.98 1.96 1.96 1.94 1.94 1.93 1.93 1.92 1.92 1.84 1.84 1.83 1.82 1.82 1.80 1.79 1.78 1.78 1.77 1.76 0.17 0.17 0.16 0.15 0.14 0.13
OTMS
10v
Analysis: ^{1}H NMR
Solvent: $CDCl_3$
Field strength: 400 MHz
2.06
2.11
2.10
2.14
5.25
9.00
8.5 8.0 7.5 7.0 6.5 6.0 5.5 5.0 4.5 4.0 3.5 3.0 2.5 2.0 1.5 1.0 0.5 0.0
f1 (ppm)
4.5×10^{7}
4.0×10^{7}
3.5×10^{7}
3.0×10^{7}
2.5×10^{7}
2.0×10^{7}
1.5×10^{7}
1.0×10^{7}
5.0×10^{6}
0.0

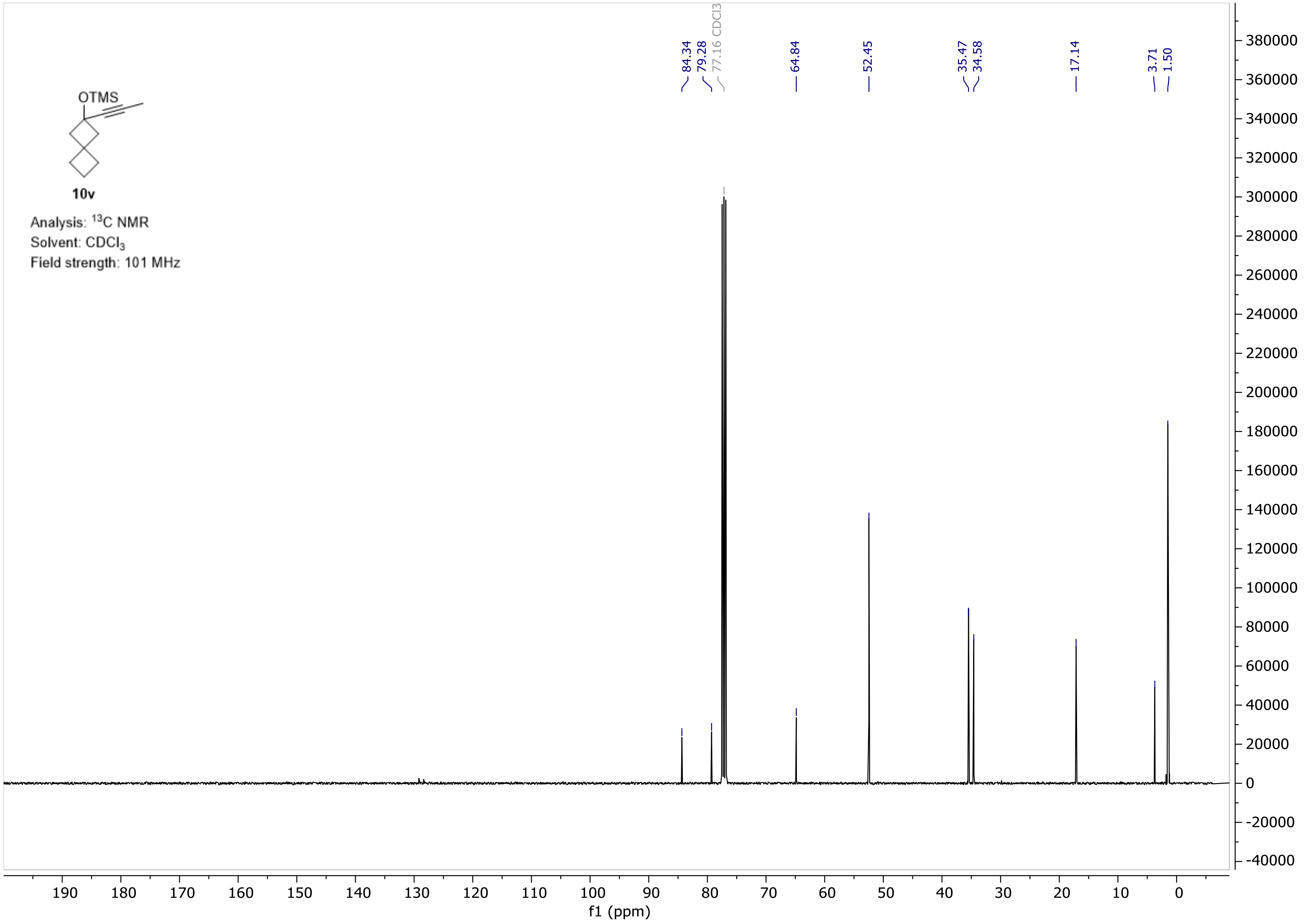
OTMS
10v
Analysis: ^{13}C NMR
Solvent: $CDCl_3$
Field strength: 101 MHz
84.34
79.28
77.16 CDCl3
64.84
52.45
35.47
34.58
17.14
3.71
1.50
f1 (ppm)

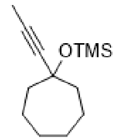

10w

Analysis: ^{1}H NMR
Solvent: $CDCl_3$
Field strength: 400 MHz

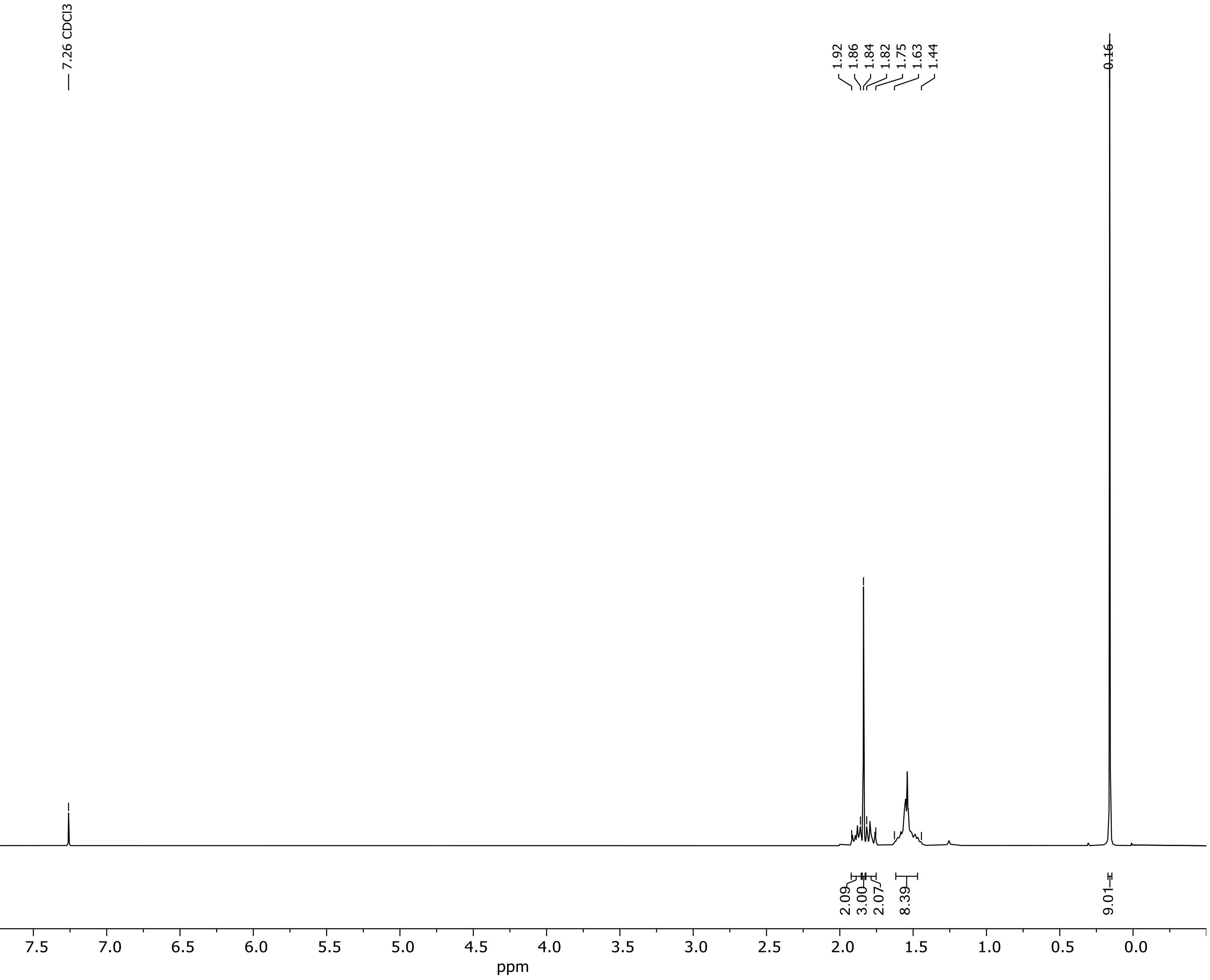

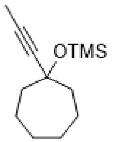

10w

Analysis: ^{13}C NMR
Solvent: $CDCl_3$
Field strength: 101 MHz

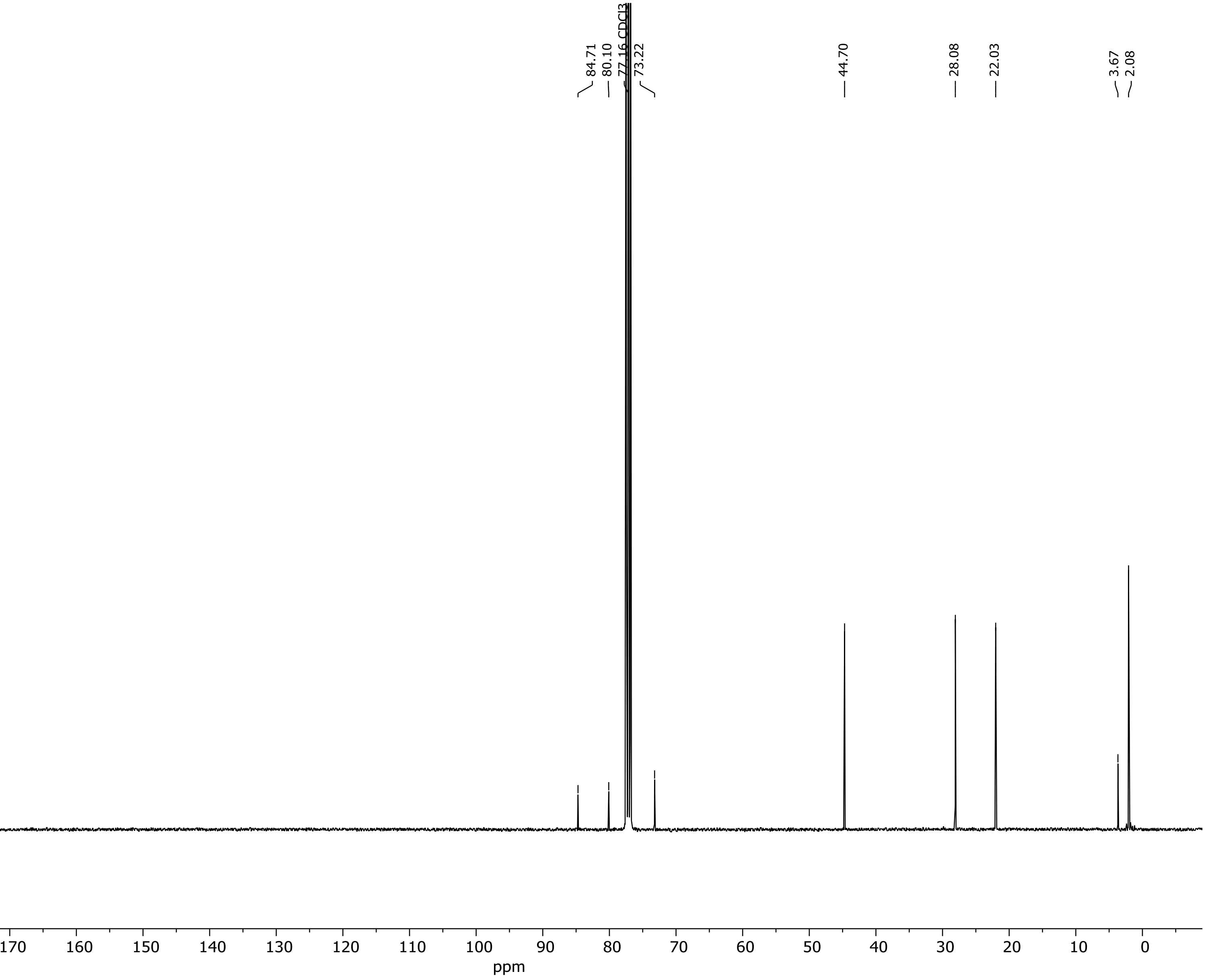

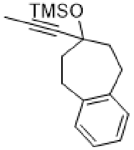

10x

Analysis: ^{1}H NMR
Solvent: $CDCl_3$
Field strength: 600 MHz

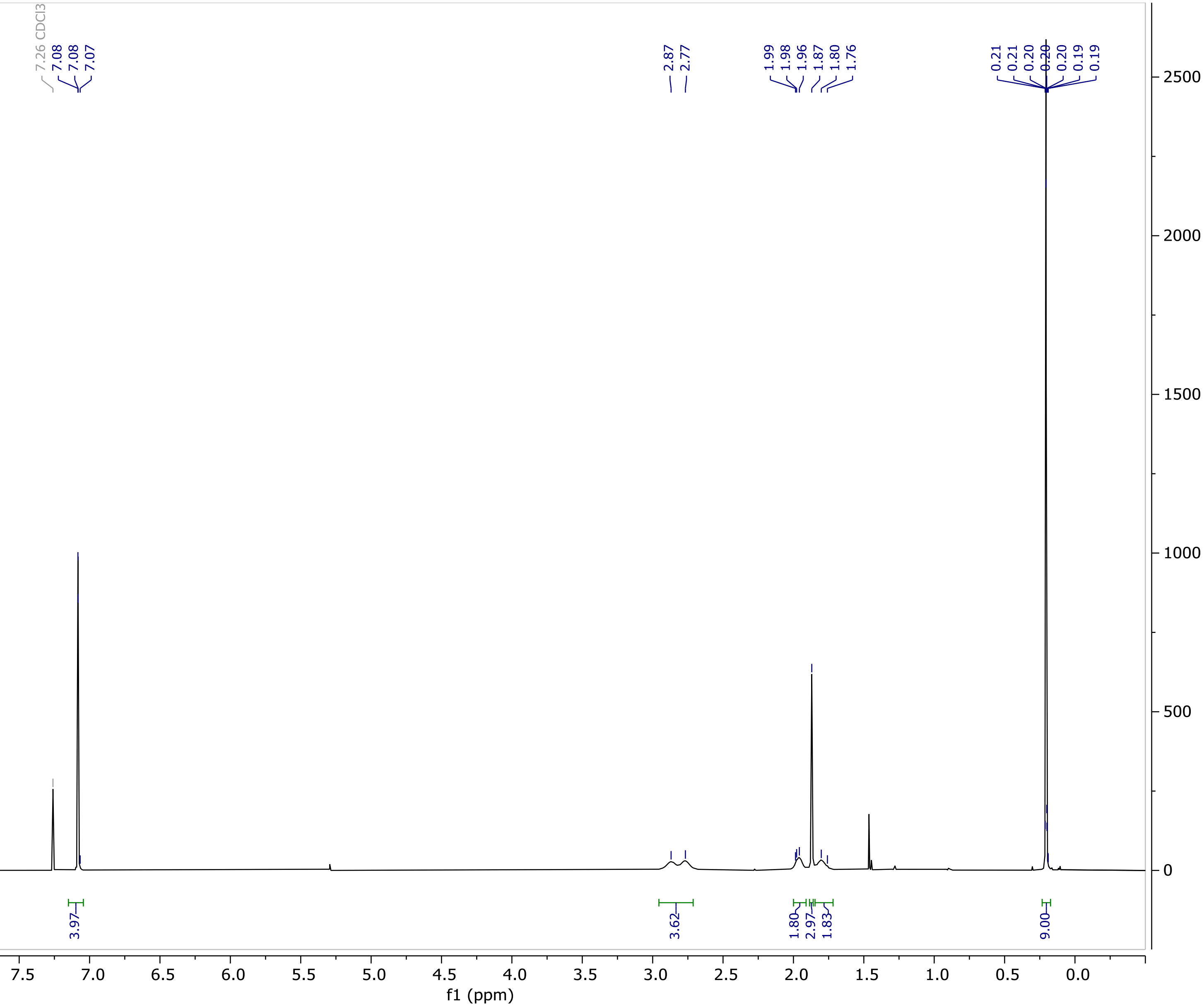

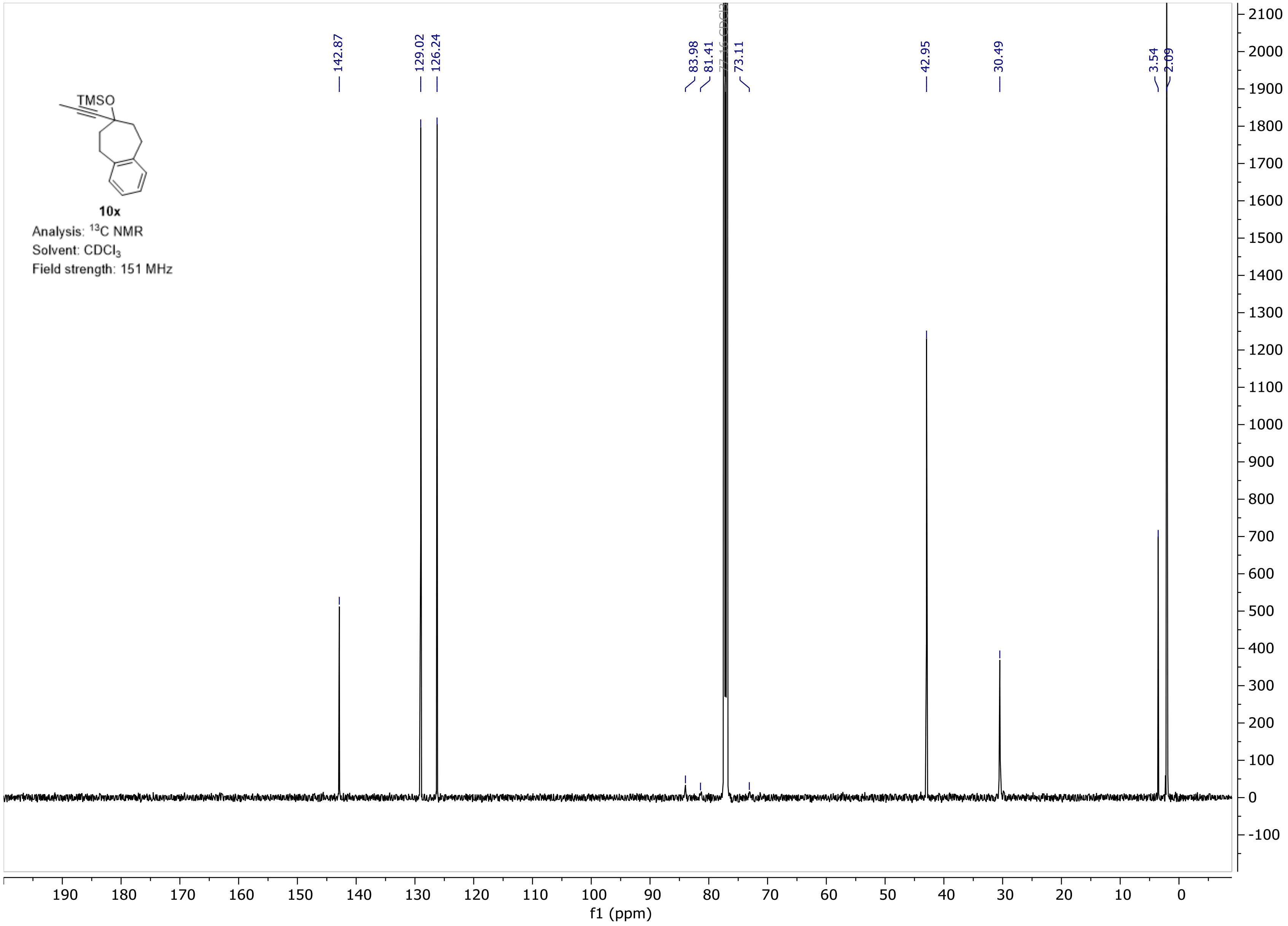
TMSO
10x
Analysis: 13C NMR
Solvent: CDCl3
Field strength: 151 MHz
142.87
129.02
126.24
83.98
81.41
77.16 CDCl3
73.11
42.95
30.49
3.54
2.09
f1 (ppm)

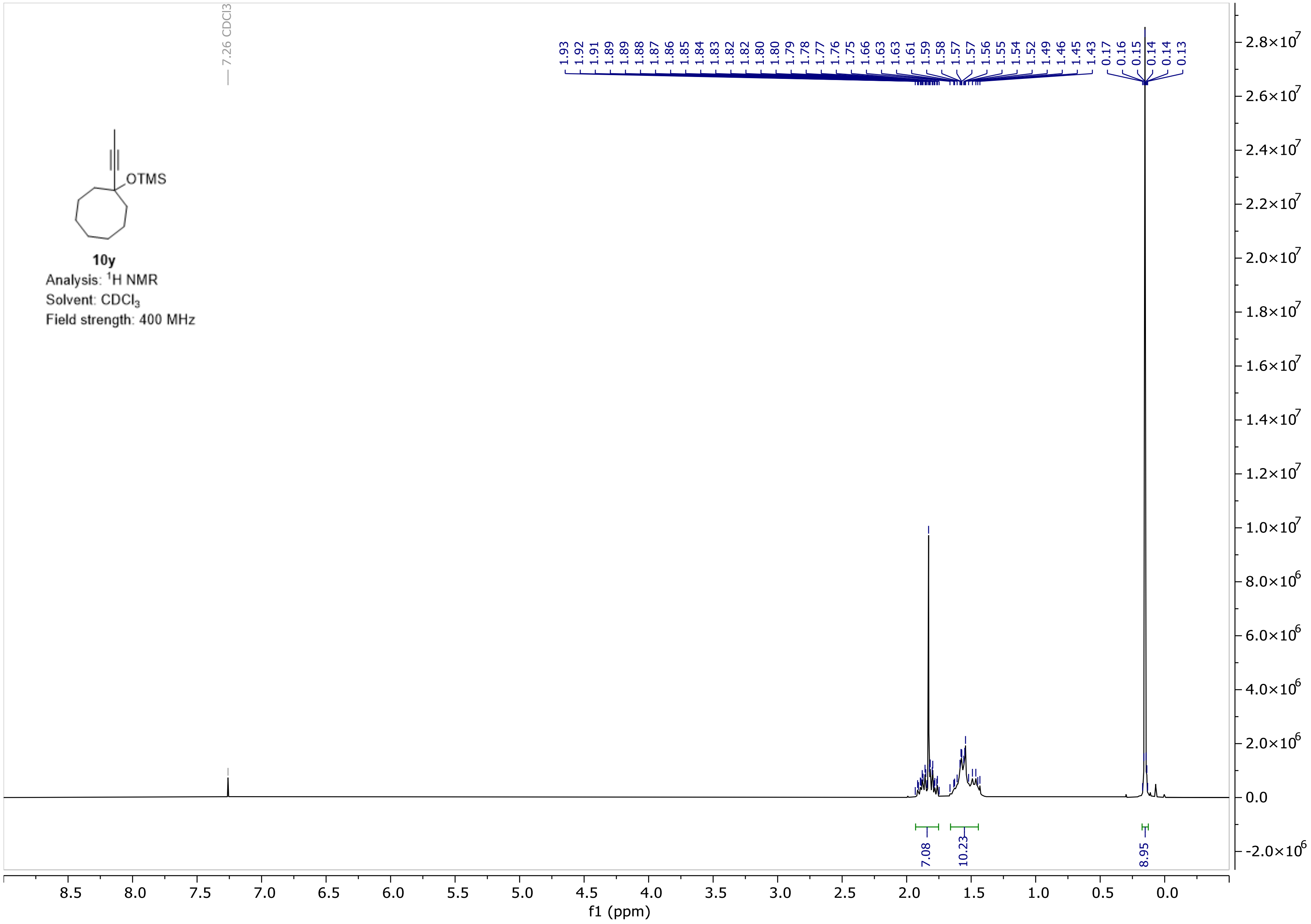

7.26 CDCl3
1.93 1.92 1.91 1.89 1.89 1.88 1.87 1.86 1.85 1.84 1.83 1.82 1.82 1.80 1.79 1.78 1.77 1.76 1.75 1.66 1.63 1.63 1.61 1.59 1.58 1.57 1.57 1.56 1.55 1.54 1.52 1.49 1.46 1.45 1.43
0.17 0.16 0.15 0.14 0.14 0.13
OTMS
10y
Analysis: ^{1}H NMR
Solvent: $CDCl_3$
Field strength: 400 MHz
7.08
10.23
8.95
f1 (ppm)

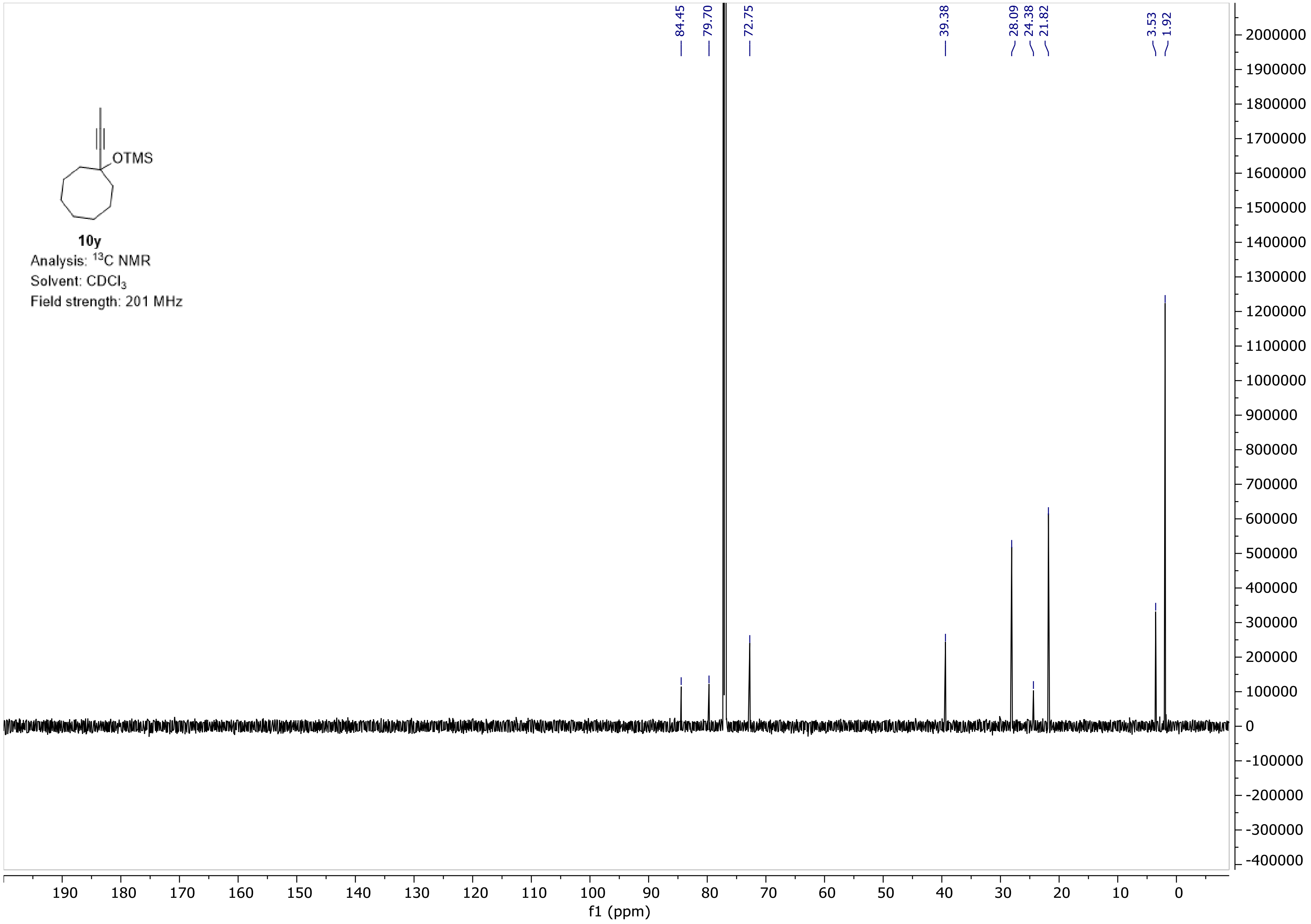

10y

Analysis: ^{13}C NMR
Solvent: $CDCl_3$
Field strength: 201 MHz

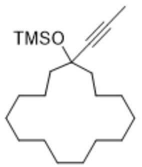

10z

Analysis: ^{1}H NMR
Solvent: $CDCl_3$
Field strength: 400 MHz

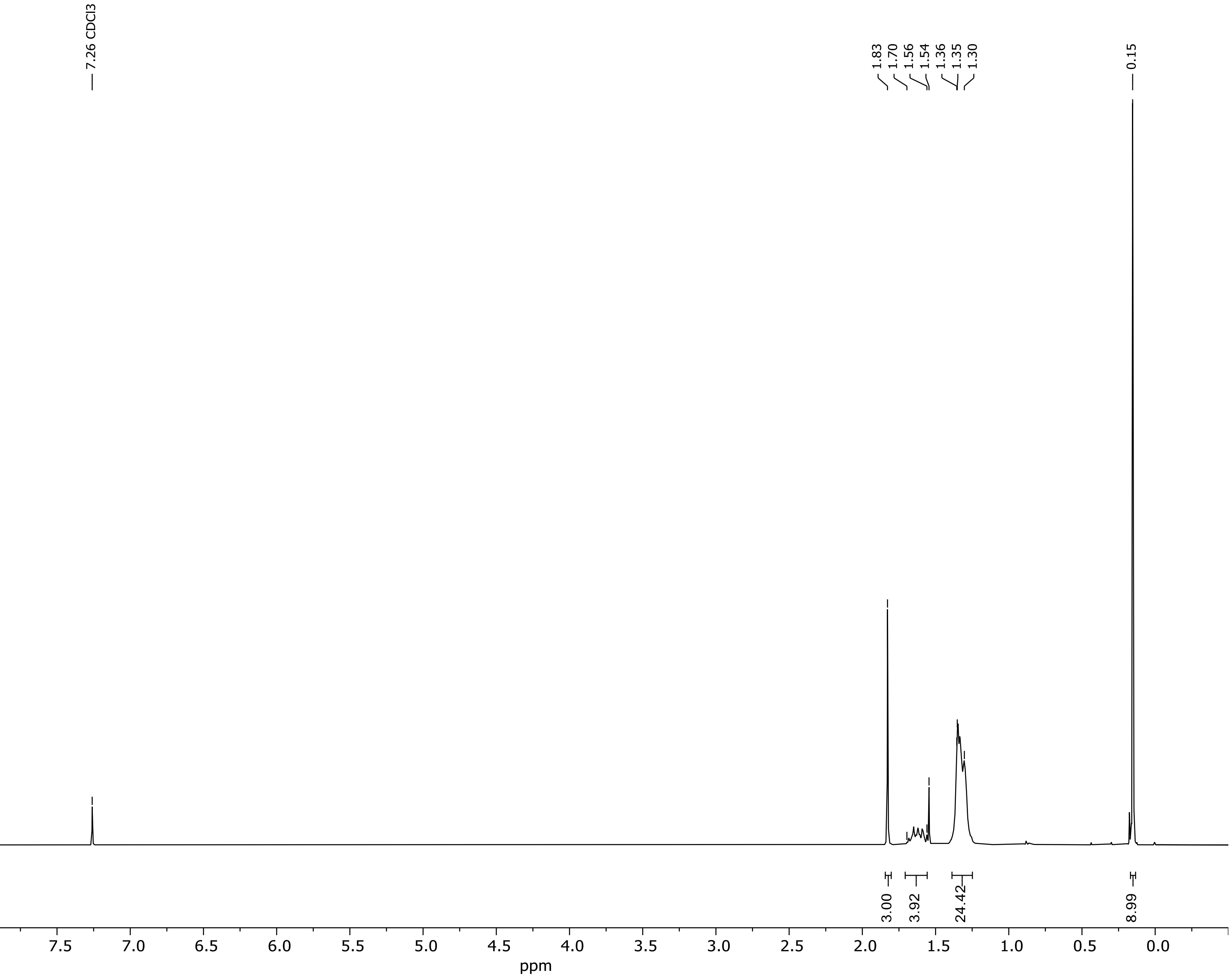

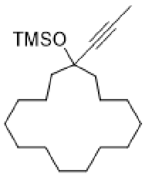

10z

Analysis: ^{13}C NMR

Solvent: $CDCl_3$

Field strength: 101 MHz

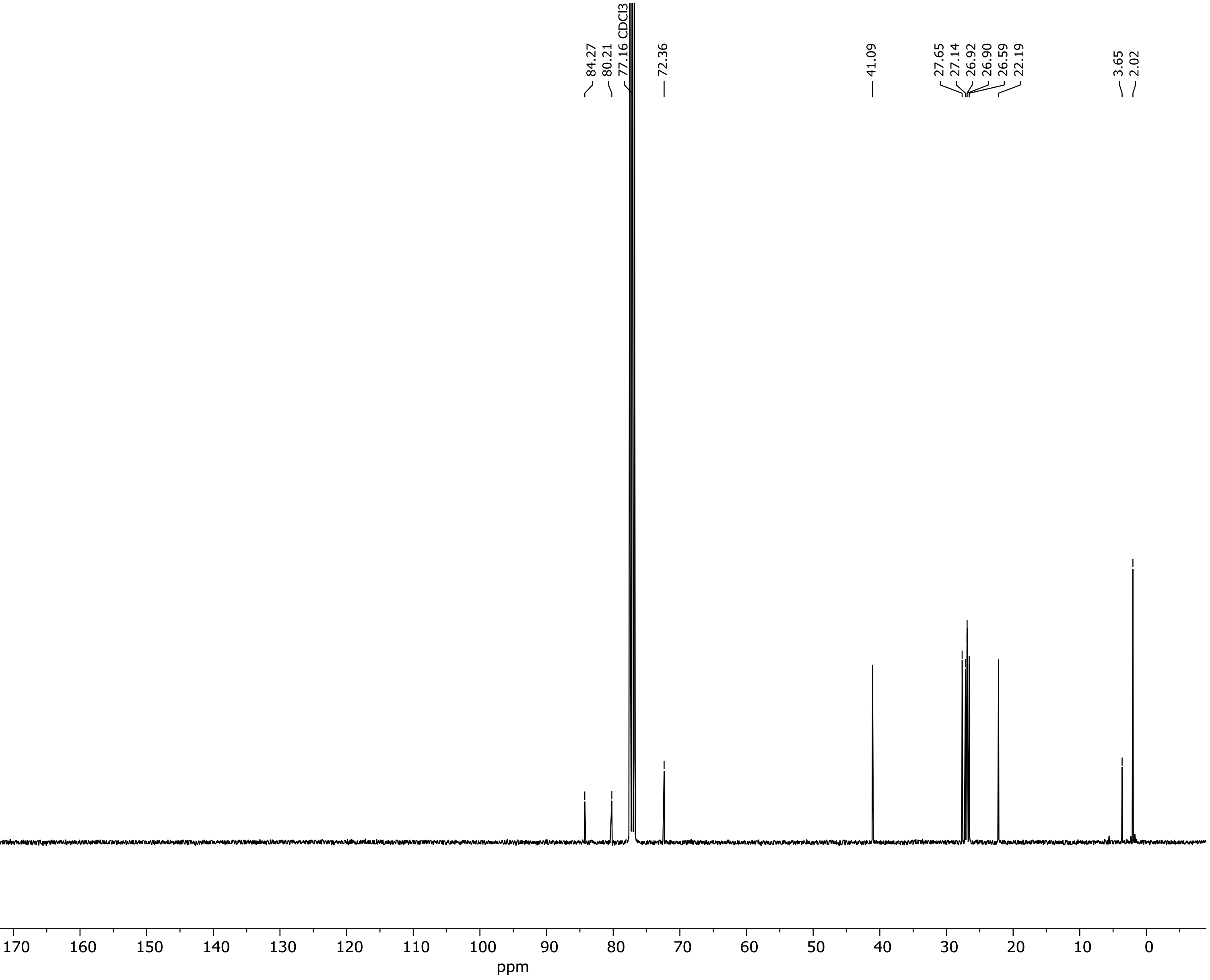

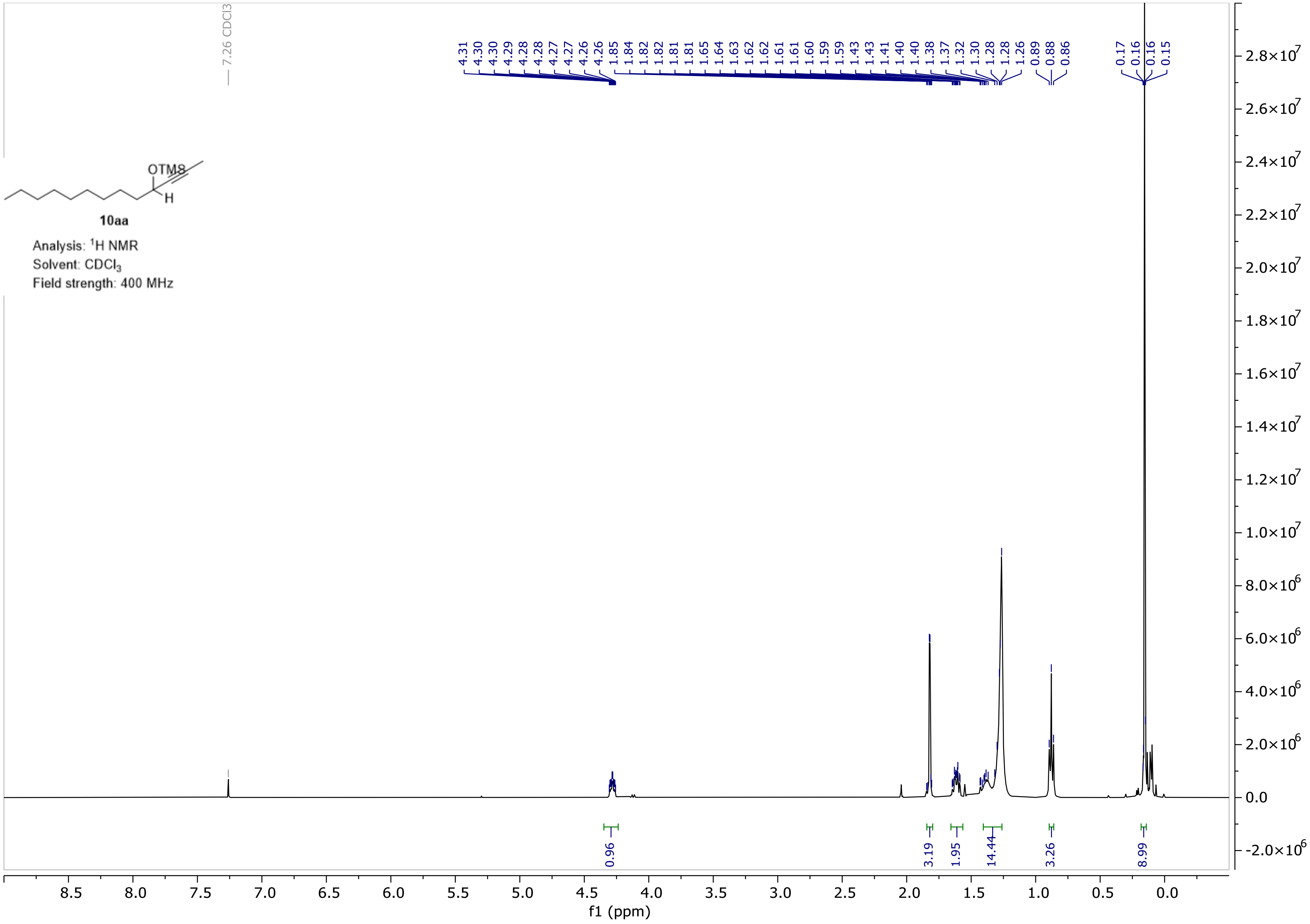
OTMS
H
10aa
Analysis: ^{1}H NMR
Solvent: $CDCl_3$
Field strength: 400 MHz
7.26 CDCl3
4.31 4.30 4.30 4.29 4.28 4.28 4.27 4.27 4.26 4.26 1.85 1.84 1.82 1.82 1.81 1.81 1.65 1.64 1.63 1.62 1.62 1.61 1.61 1.60 1.59 1.59 1.43 1.43 1.41 1.40 1.40 1.38 1.37 1.32 1.30 1.28 1.28 1.26 0.89 0.88 0.86
0.17 0.16 0.16 0.15
0.96
3.19
1.95
14.44
3.26
8.99
f1 (ppm)

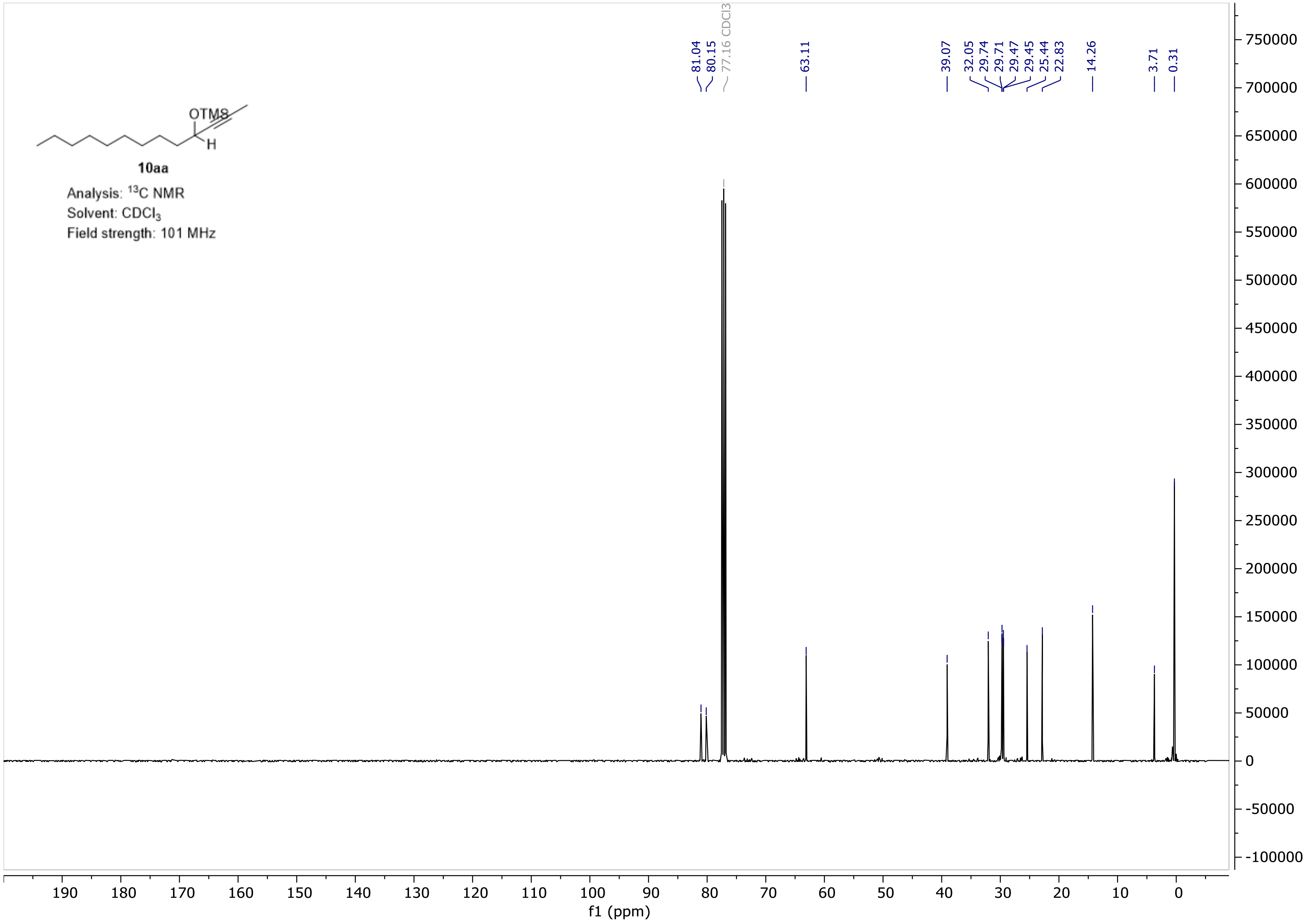

81.04
80.15
77.16 CDCl3
63.11
39.07
32.05
29.74
29.71
29.47
29.45
25.44
22.83
14.26
3.71
0.31
OTMS
H
10aa
Analysis: 13C NMR
Solvent: CDCl3
Field strength: 101 MHz
f1 (ppm)

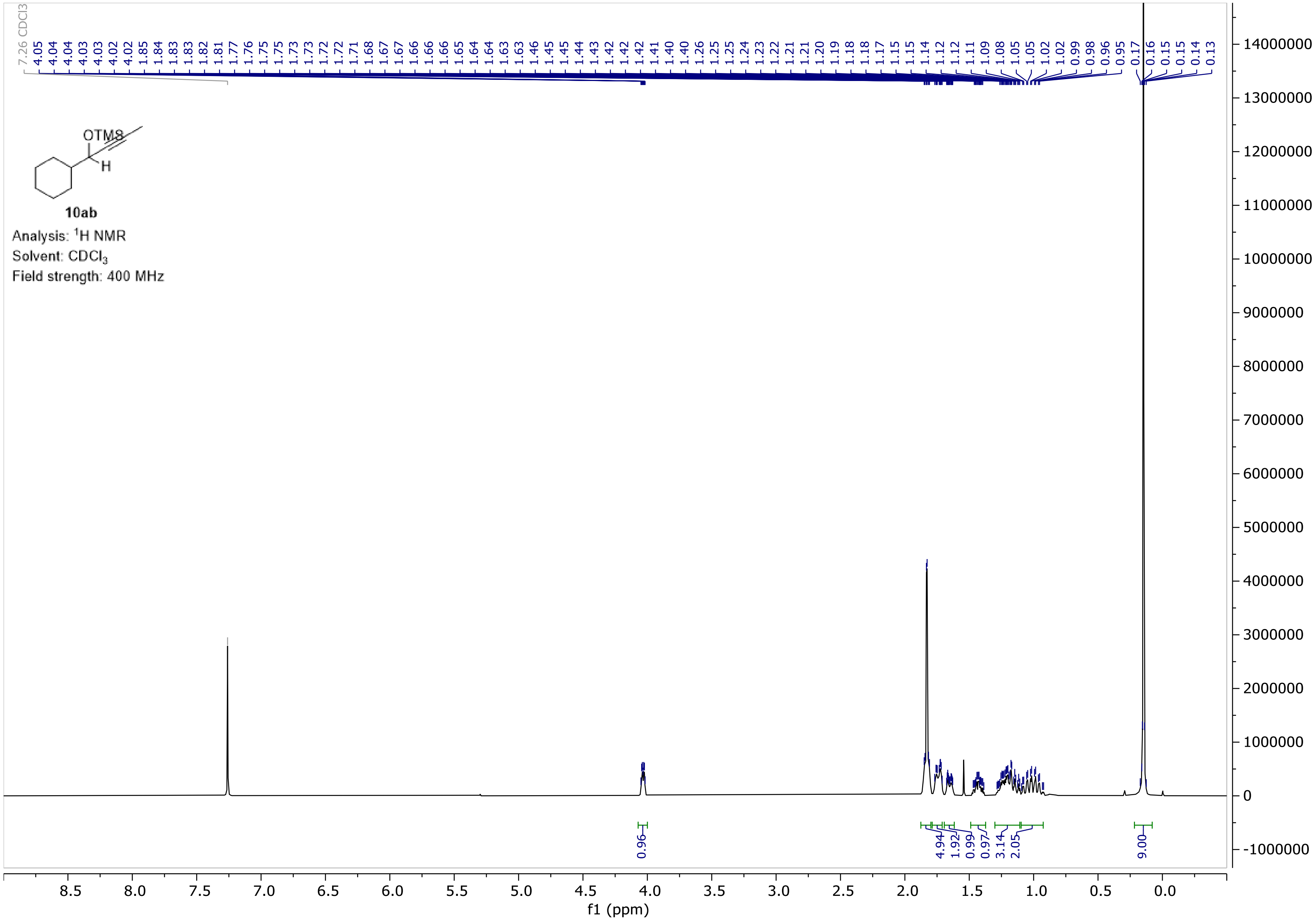
OTMS
H
10ab
Analysis: ^{1}H NMR
Solvent: $CDCl_3$
Field strength: 400 MHz
f1 (ppm)

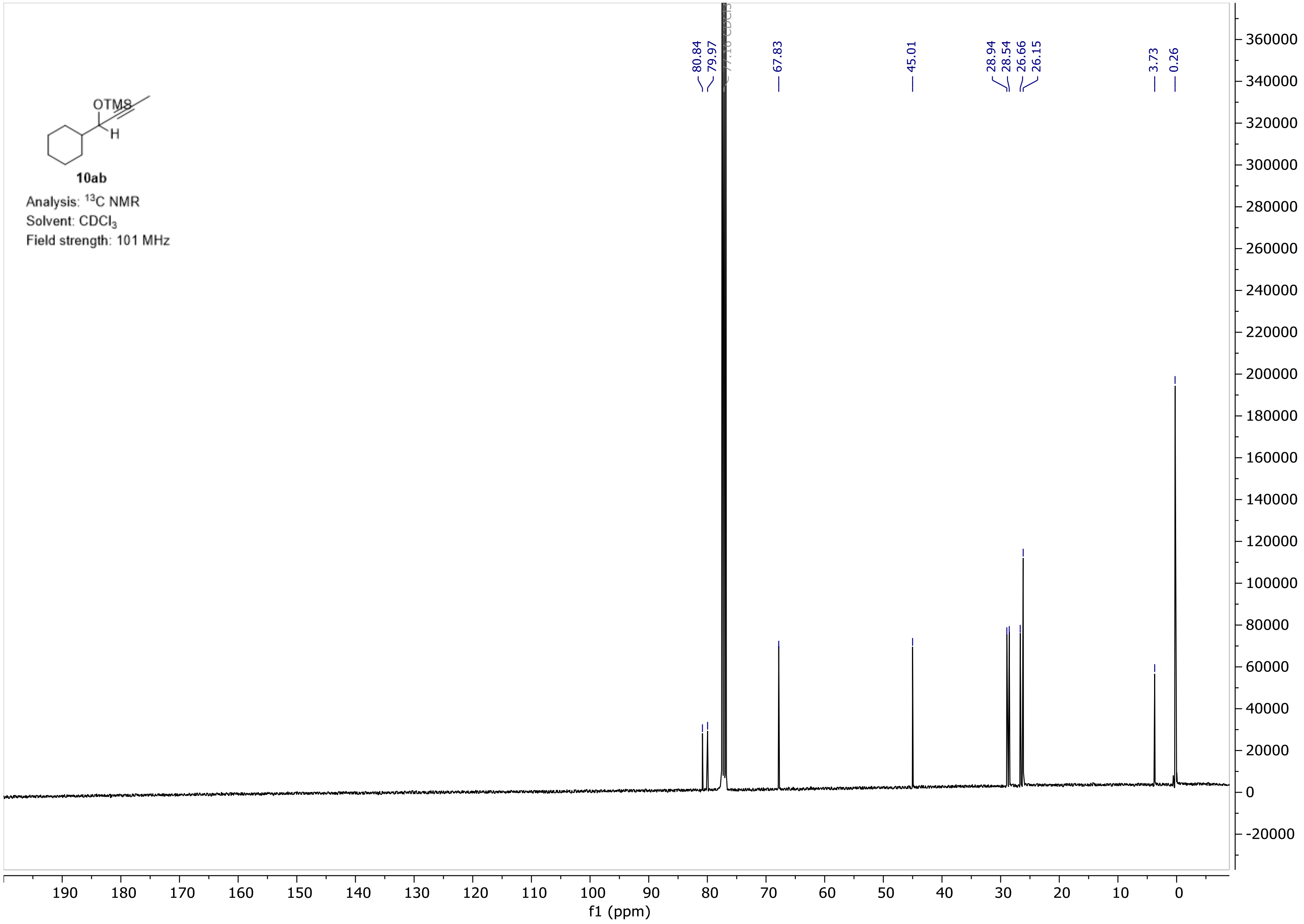
OTMS
H
10ab
Analysis: ¹³C NMR
Solvent: CDCl3
Field strength: 101 MHz
80.84
79.97
77.16 CDCl3
67.83
45.01
28.94
28.54
26.66
26.15
3.73
0.26
f1 (ppm)

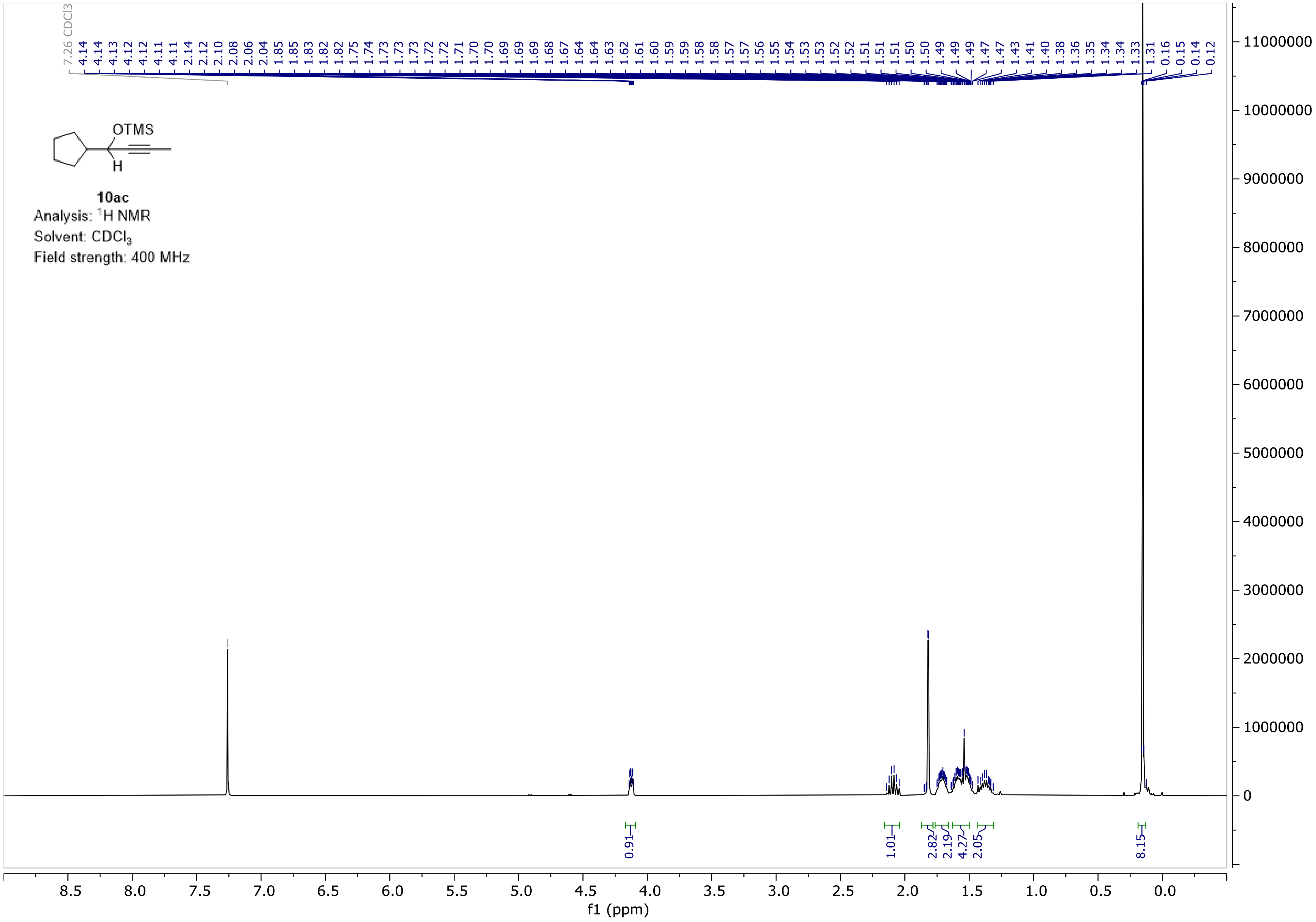

OTMS
H
10ac
Analysis: 1H NMR
Solvent: CDCl3
Field strength: 400 MHz
f1 (ppm)

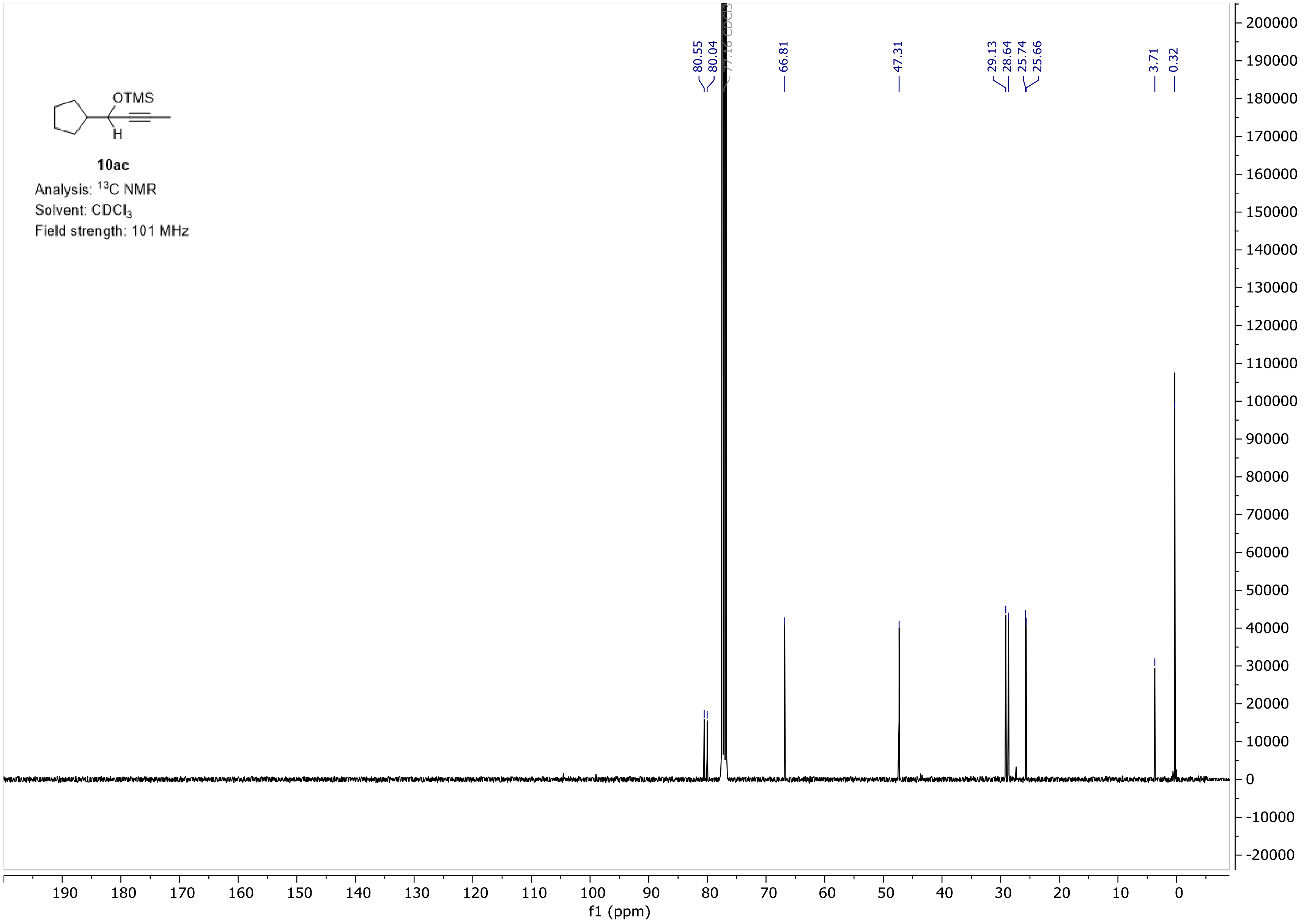
OTMS
H
10ac
Analysis: 13C NMR
Solvent: CDCl3
Field strength: 101 MHz
80.55
80.04
77.16 CDCl3
66.81
47.31
29.13
28.64
25.74
25.66
3.71
0.32
f1 (ppm)

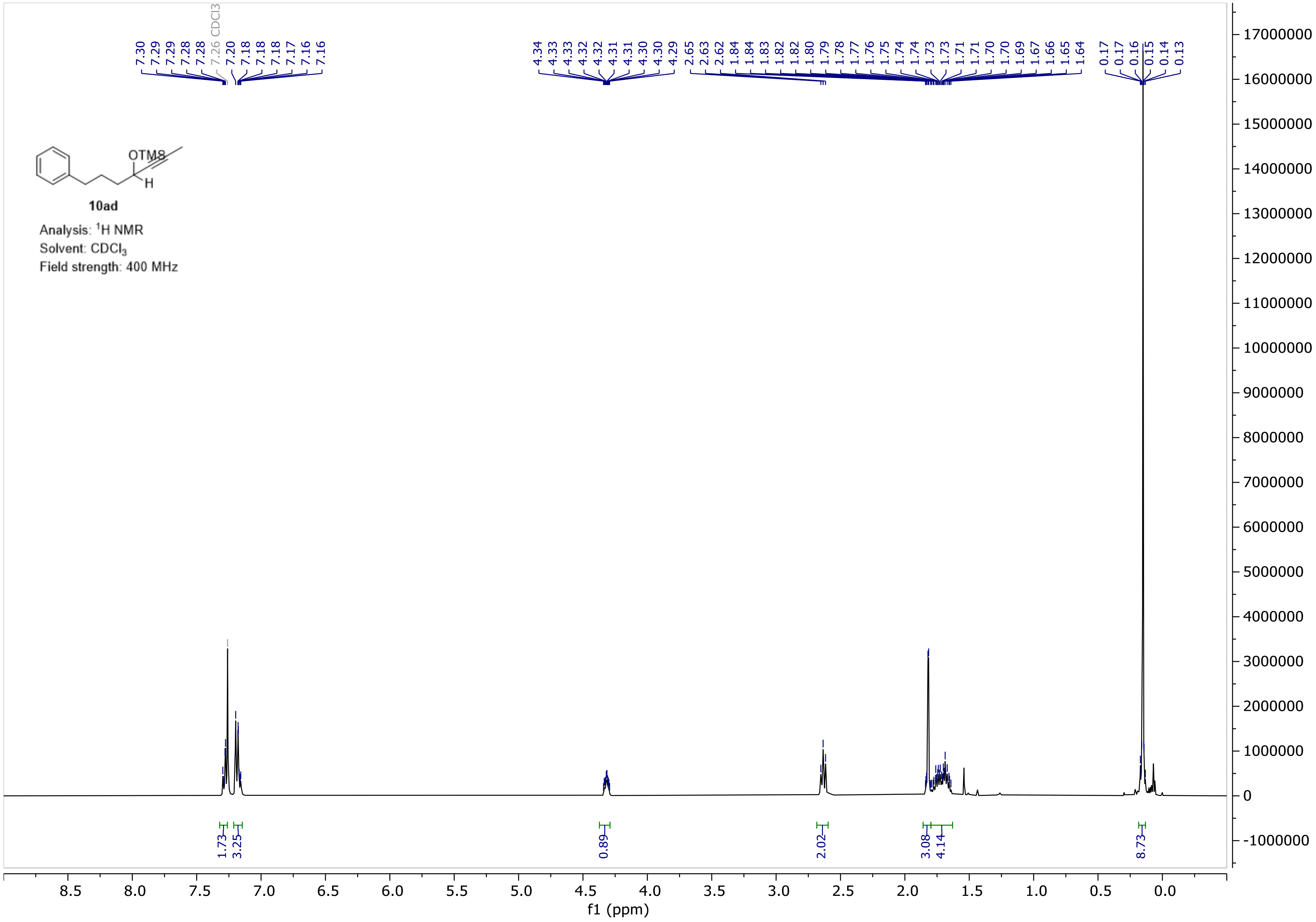

10ad
Analysis: 1H NMR
Solvent: CDCl3
Field strength: 400 MHz
OTMS
H
7.30 7.29 7.29 7.28 7.28 7.26 CDCl3 7.20 7.18 7.18 7.18 7.17 7.16 7.16
4.34 4.33 4.33 4.32 4.32 4.31 4.31 4.30 4.30 4.29
2.65 2.63 2.62 1.84 1.84 1.83 1.82 1.82 1.80 1.79 1.78 1.77 1.76 1.75 1.74 1.74 1.73 1.73 1.71 1.71 1.70 1.69 1.67 1.66 1.65 1.64
0.17 0.17 0.16 0.15 0.14 0.13
1.73 3.25 0.89 2.02 3.08 4.14 8.73
f1 (ppm)

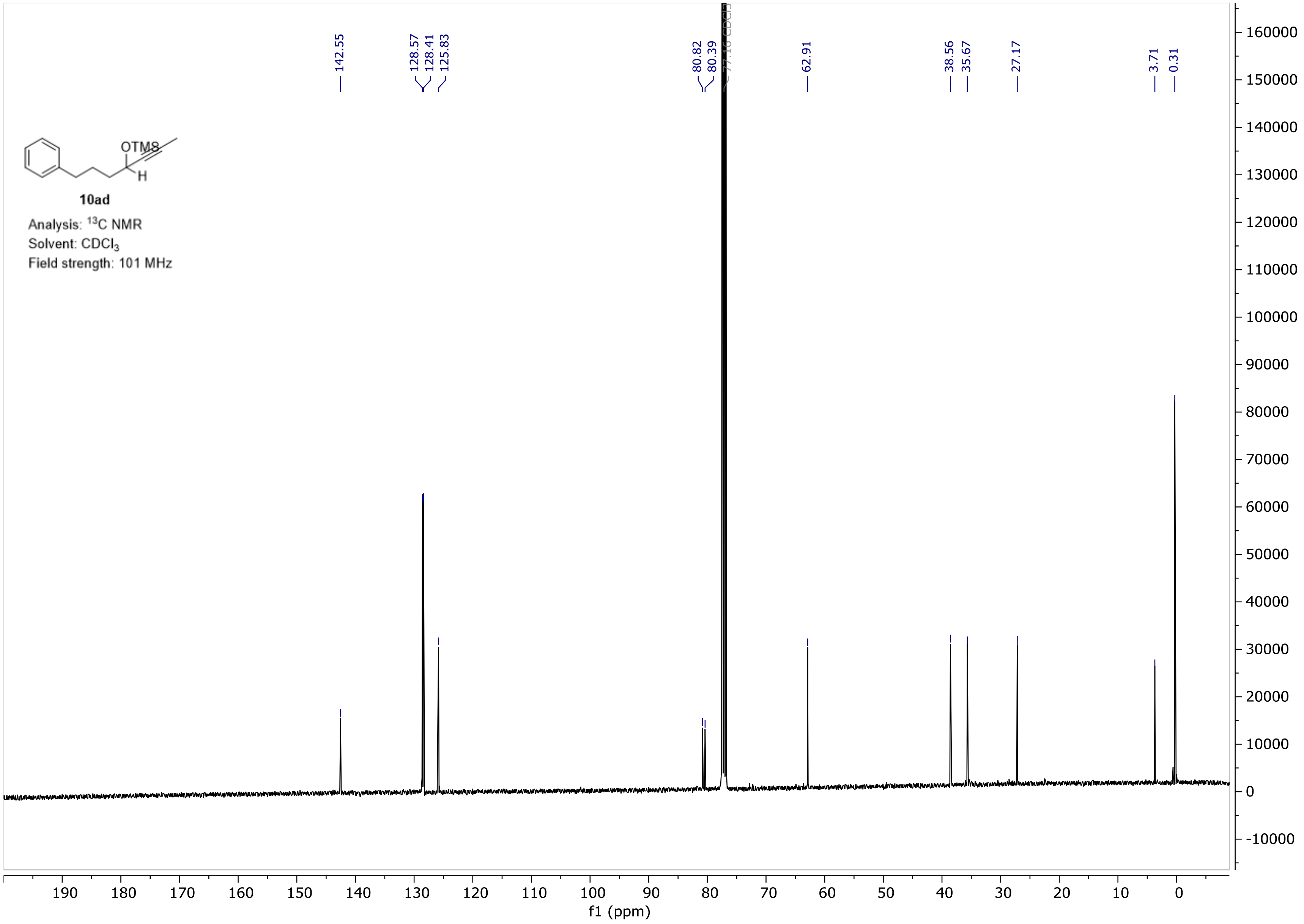

142.55
128.57
128.41
125.83
80.82
80.39
62.91
38.56
35.67
27.17
3.71
0.31
OTMS
H
10ad
Analysis: ¹³C NMR
Solvent: CDCl3
Field strength: 101 MHz
f1 (ppm)

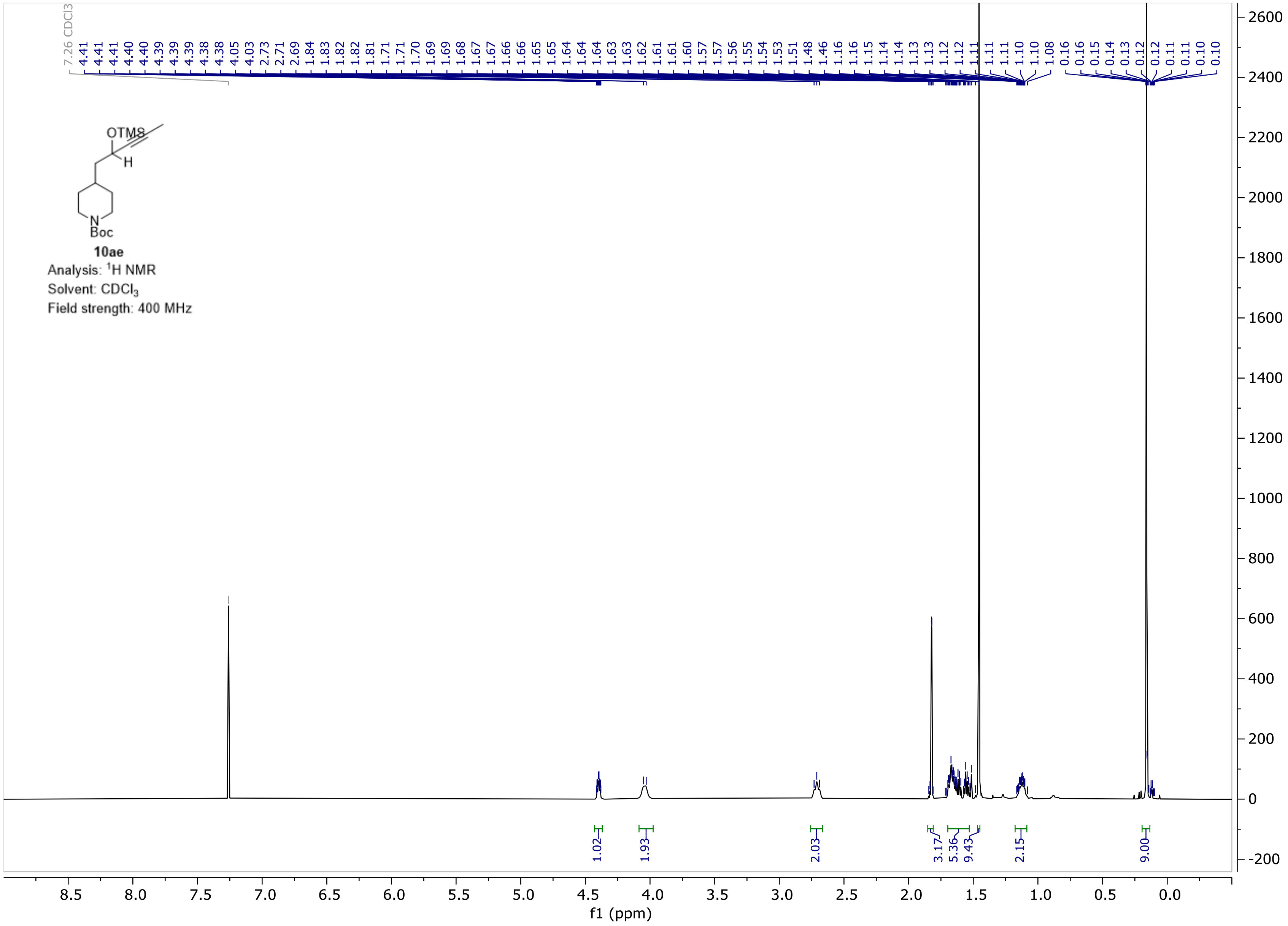

7.26 CDCl3
4.41
4.41
4.41
4.40
4.40
4.39
4.39
4.39
4.38
4.38
4.05
4.03
2.73
2.71
2.69
1.84
1.83
1.82
1.82
1.81
1.71
1.71
1.70
1.69
1.69
1.68
1.67
1.67
1.66
1.66
1.65
1.65
1.64
1.64
1.64
1.63
1.63
1.62
1.61
1.61
1.60
1.57
1.57
1.56
1.55
1.54
1.53
1.51
1.48
1.46
1.16
1.16
1.15
1.14
1.14
1.13
1.13
1.12
1.12
1.11
1.11
1.11
1.10
1.10
1.08
0.16
0.16
0.15
0.14
0.13
0.12
0.12
0.11
0.11
0.10
0.10
OTMS
H
N
Boc
10ae
Analysis: 1H NMR
Solvent: CDCl3
Field strength: 400 MHz
1.02
1.93
2.03
3.17
5.36
9.43
2.15
9.00
f1 (ppm)

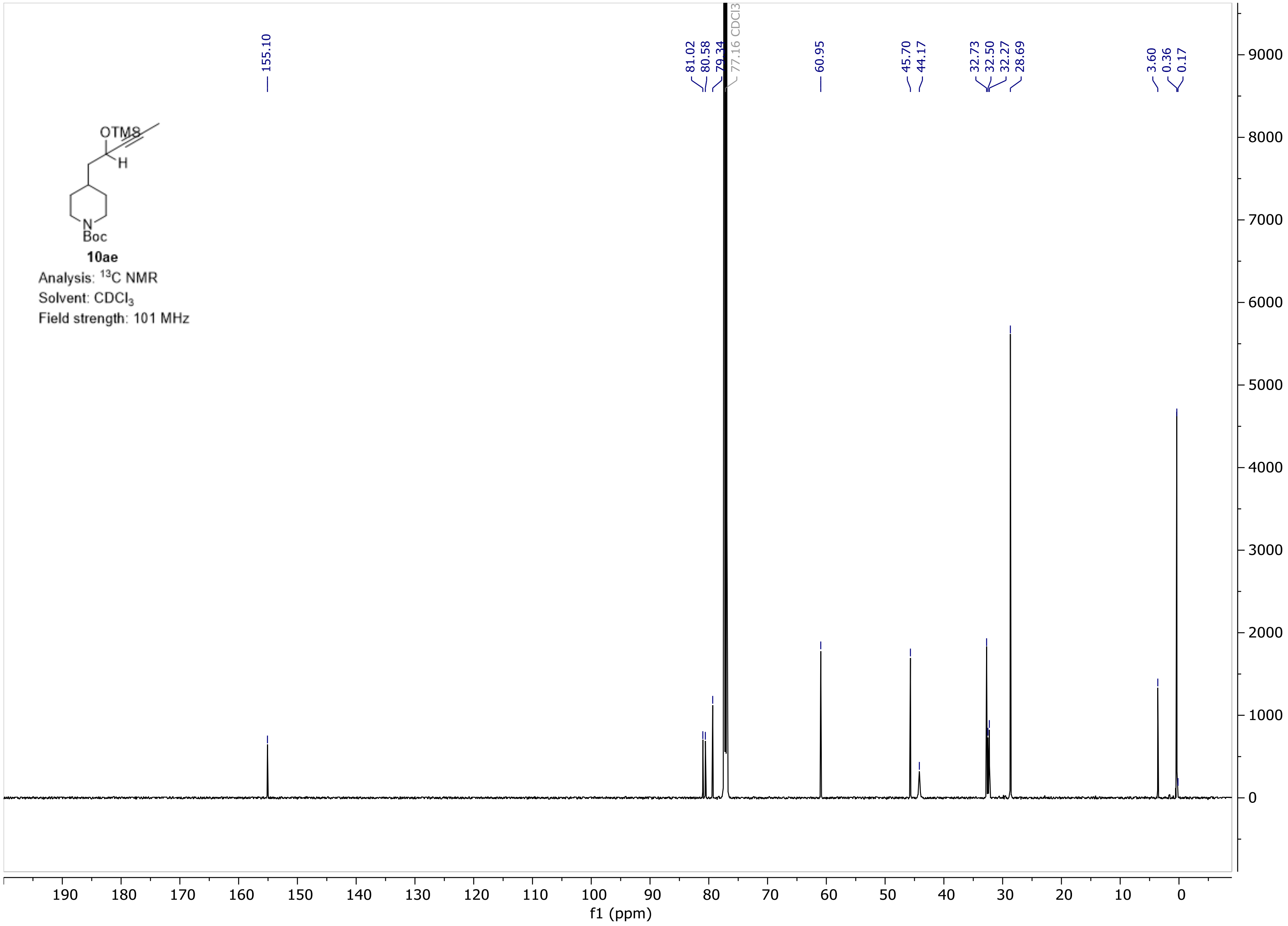

155.10
81.02
80.58
79.34
77.16 CDCl3
60.95
45.70
44.17
32.73
32.50
32.27
28.69
3.60
0.36
0.17
OTMS
H
N
Boc
10ae
Analysis: 13C NMR
Solvent: CDCl3
Field strength: 101 MHz
f1 (ppm)

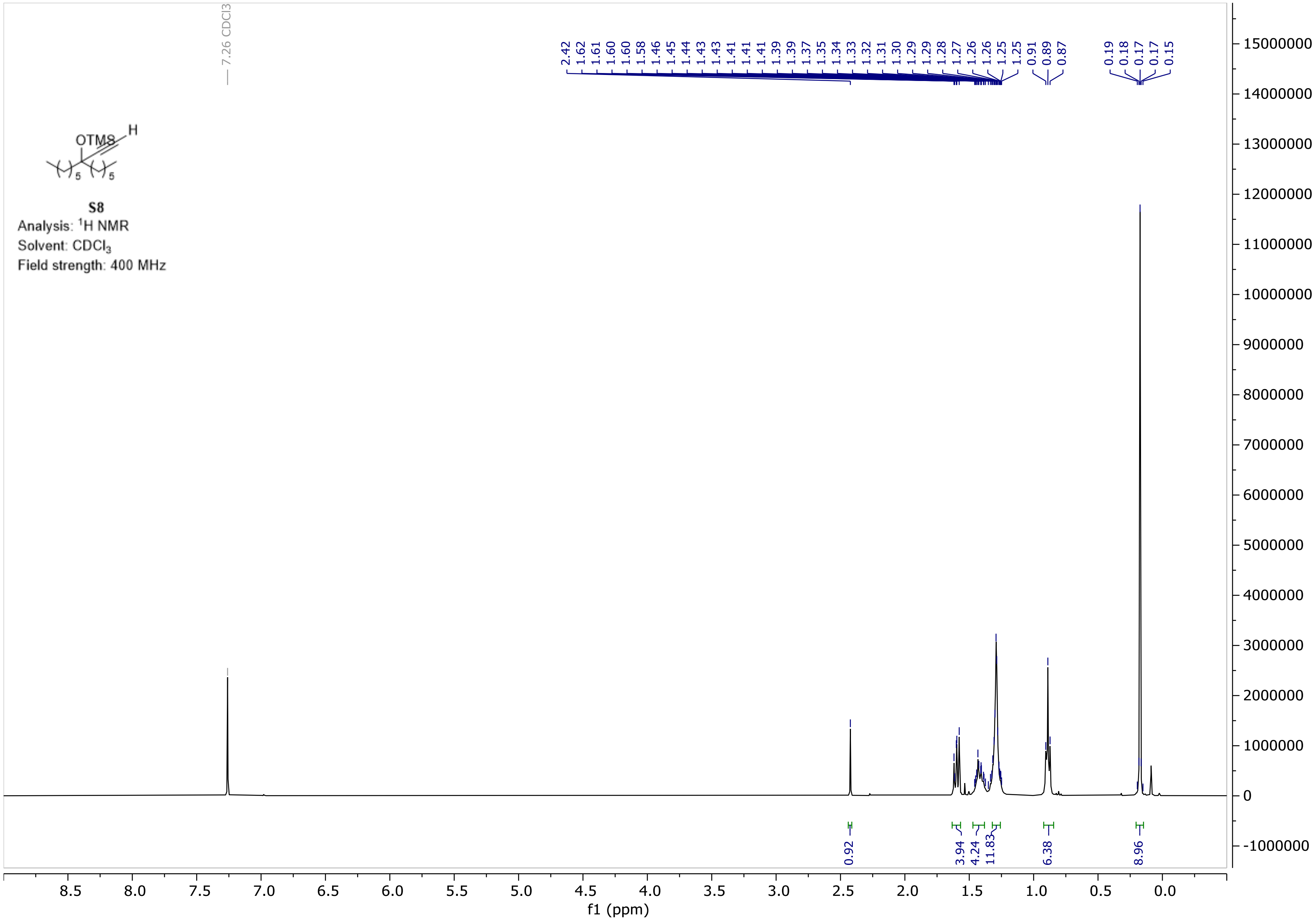
7.26 CDCl3
2.42 1.62 1.61 1.60 1.60 1.58 1.46 1.45 1.44 1.43 1.43 1.41 1.41 1.41 1.39 1.39 1.37 1.35 1.34 1.33 1.32 1.31 1.30 1.29 1.29 1.28 1.27 1.26 1.26 1.25 1.25 0.91 0.89 0.87
0.19 0.18 0.17 0.17 0.15
OTMS
H
5
5
S8
Analysis: 1H NMR
Solvent: CDCl3
Field strength: 400 MHz
0.92
3.94
4.24
11.83
6.38
8.96
8.5 8.0 7.5 7.0 6.5 6.0 5.5 5.0 4.5 4.0 3.5 3.0 2.5 2.0 1.5 1.0 0.5 0.0
f1 (ppm)
15000000 14000000 13000000 12000000 11000000 10000000 9000000 8000000 7000000 6000000 5000000 4000000 3000000 2000000 1000000 0 -1000000

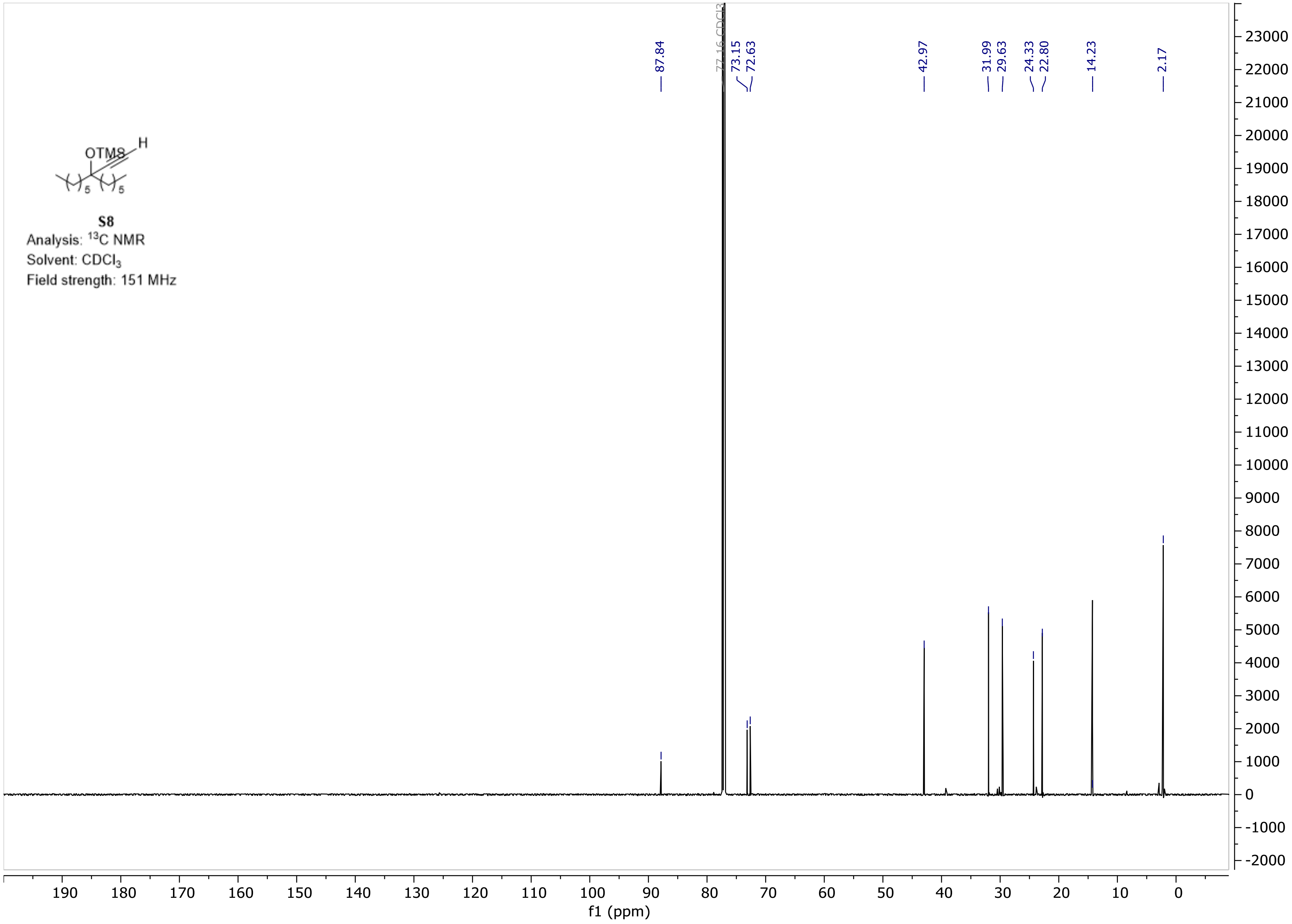

S8
Analysis: ^{13}C NMR
Solvent: $CDCl_3$
Field strength: 151 MHz

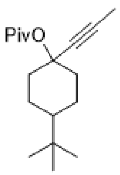

S10

Analysis: ^{1}H NMR
Solvent: $CDCl_3$
Field strength: 400 MHz

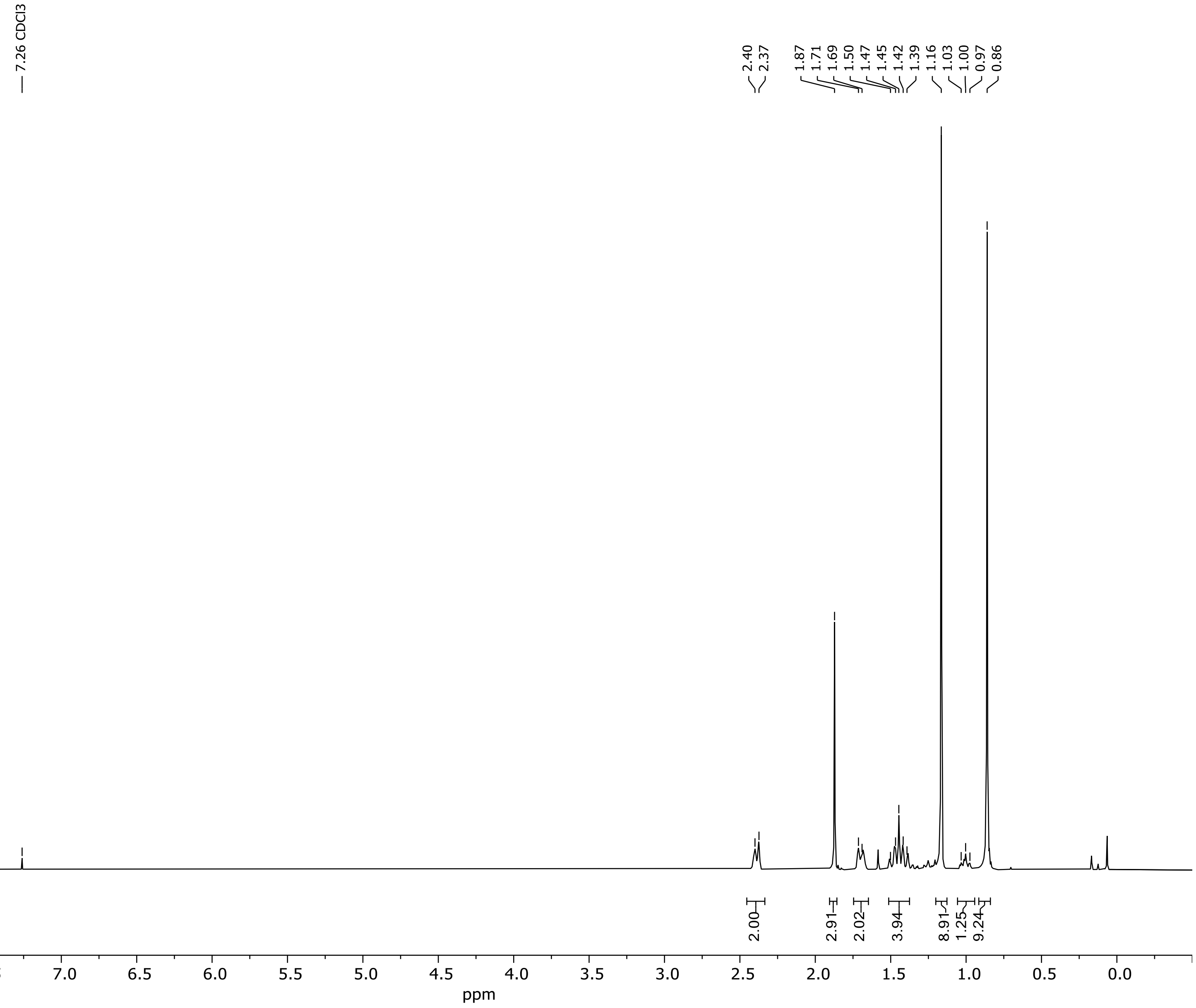

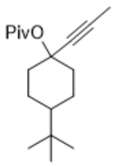

S10

Analysis: ^{13}C NMR
Solvent: $CDCl_3$
Field strength: 101 MHz

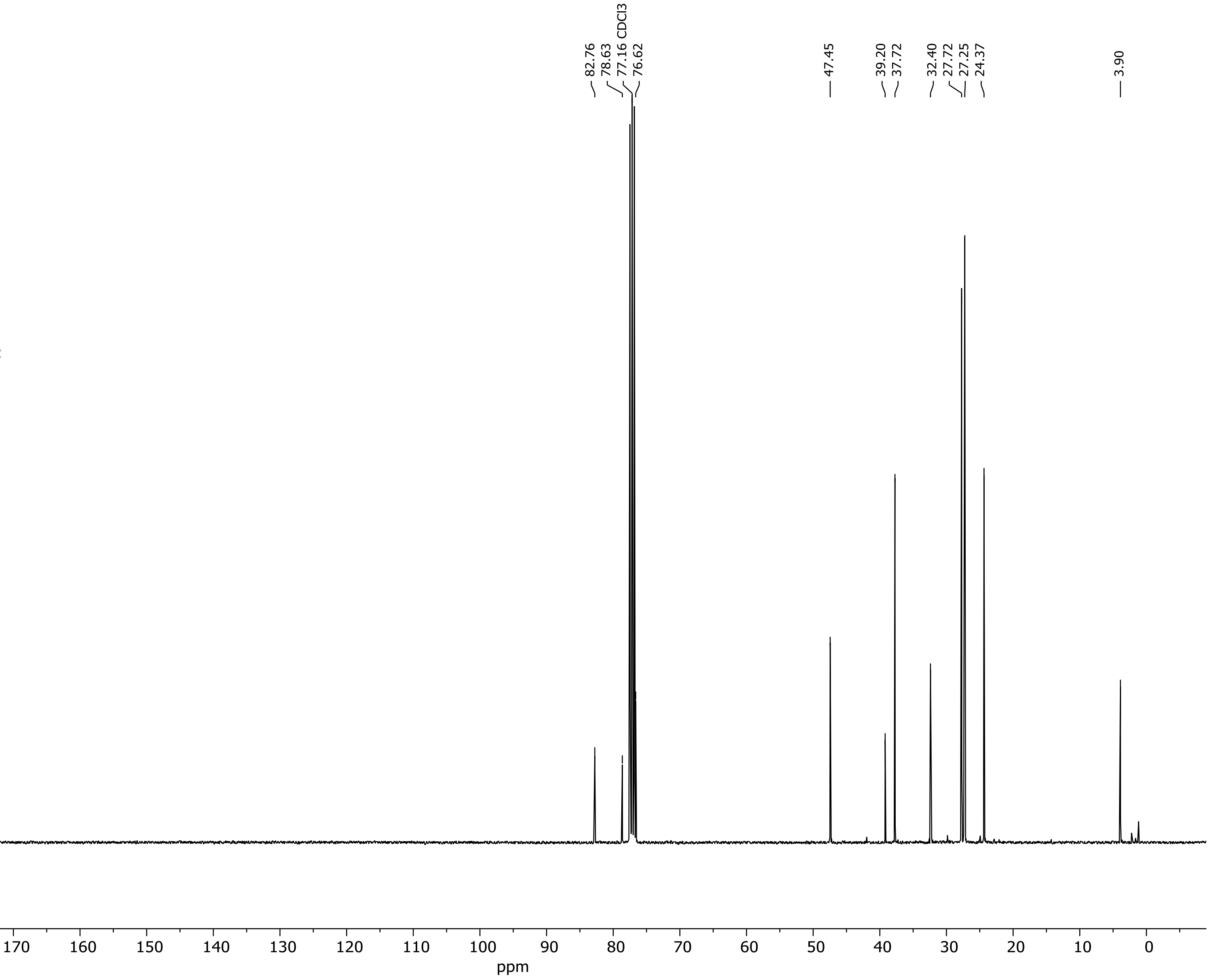

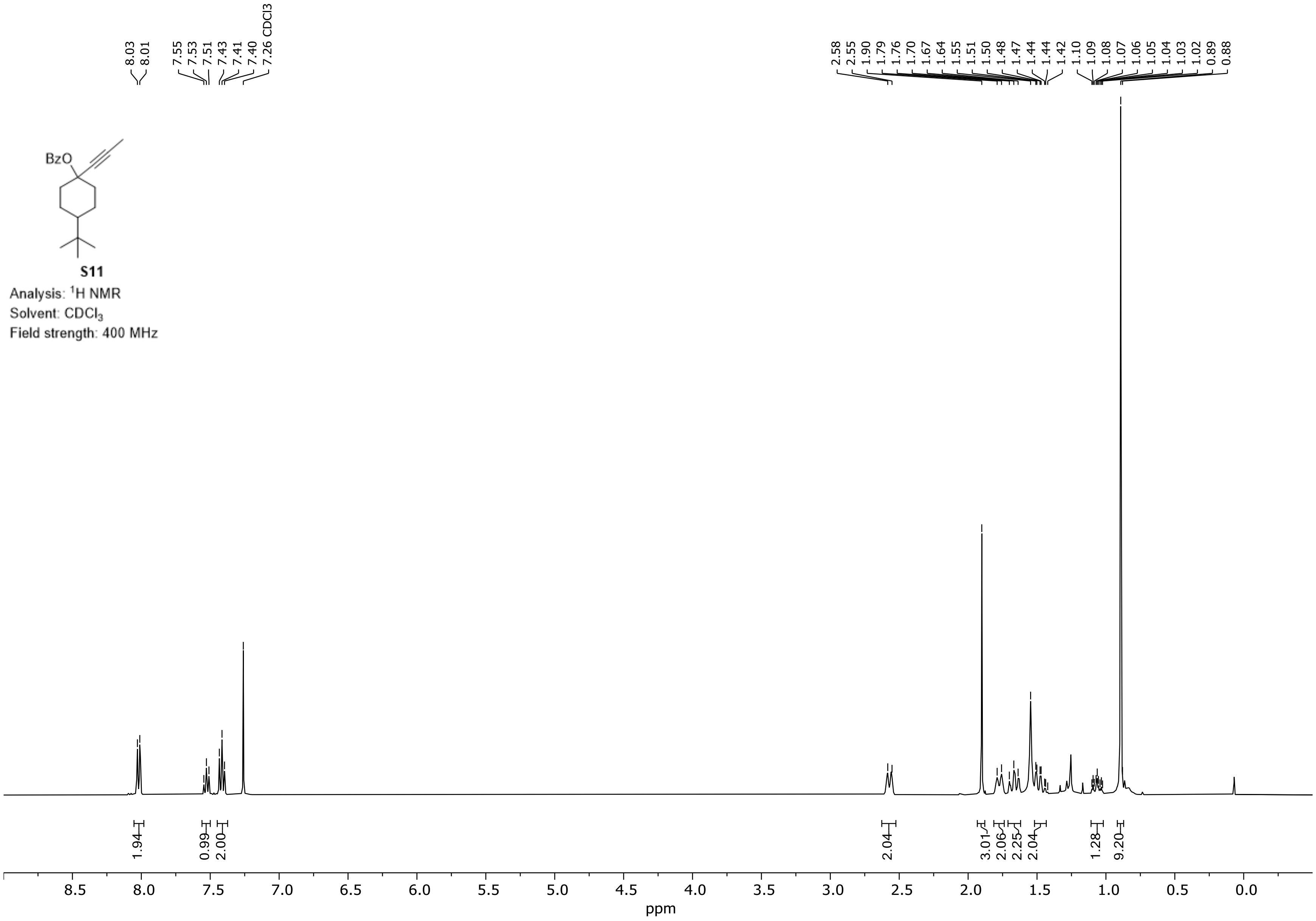
BzO
S11
Analysis: ^{1}H NMR
Solvent: $CDCl_3$
Field strength: 400 MHz
8.03
8.01
7.55
7.53
7.51
7.43
7.41
7.40
7.26 CDCl3
2.58
2.55
1.90
1.79
1.76
1.70
1.67
1.64
1.55
1.51
1.50
1.48
1.47
1.44
1.44
1.42
1.10
1.09
1.08
1.07
1.06
1.05
1.04
1.03
1.02
0.89
0.88
1.94
0.99
2.00
2.04
3.01
2.06
2.25
2.04
1.28
9.20
ppm

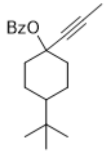

S11

Analysis: ^{13}C NMR

Solvent: $CDCl_3$

Field strength: 101 MHz

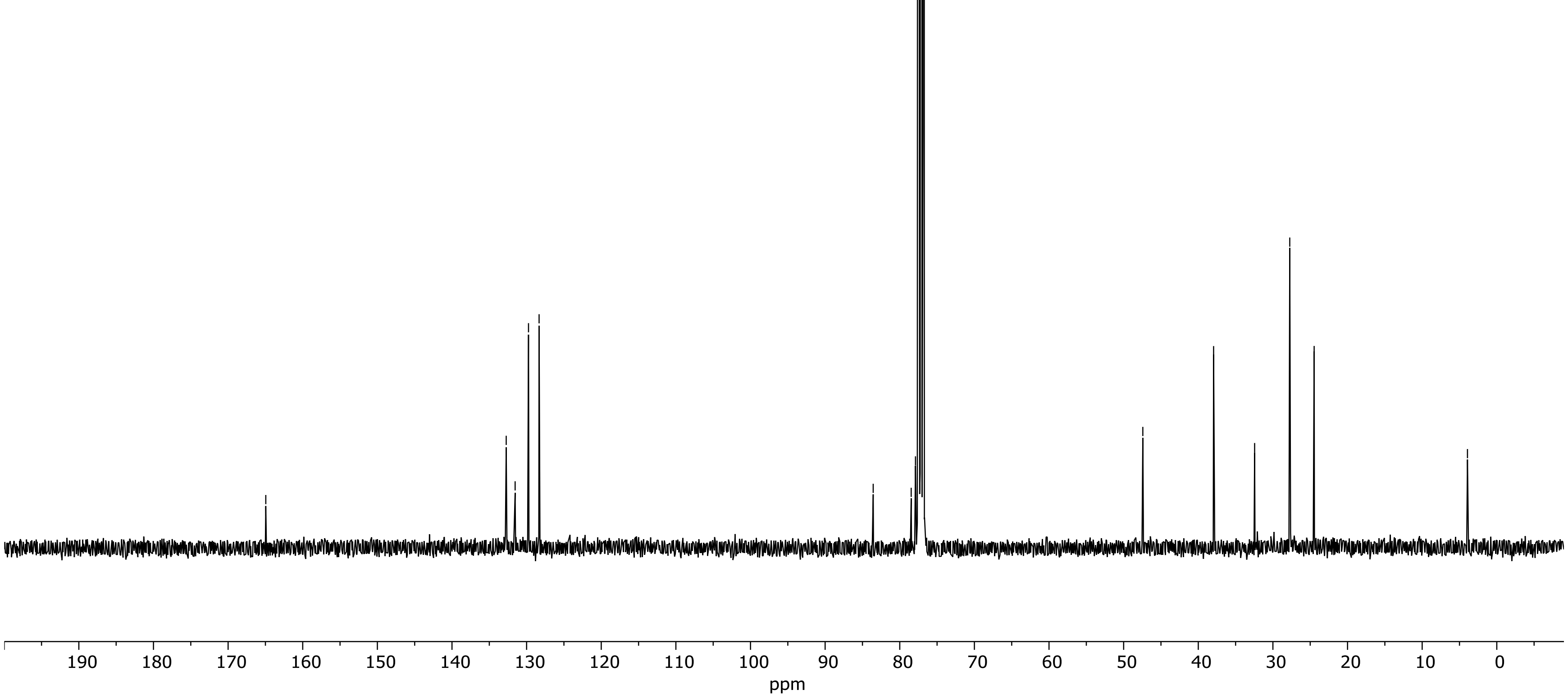

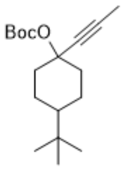

S12

Analysis: ^{1}H NMR
Solvent: $CDCl_3$
Field strength: 400 MHz

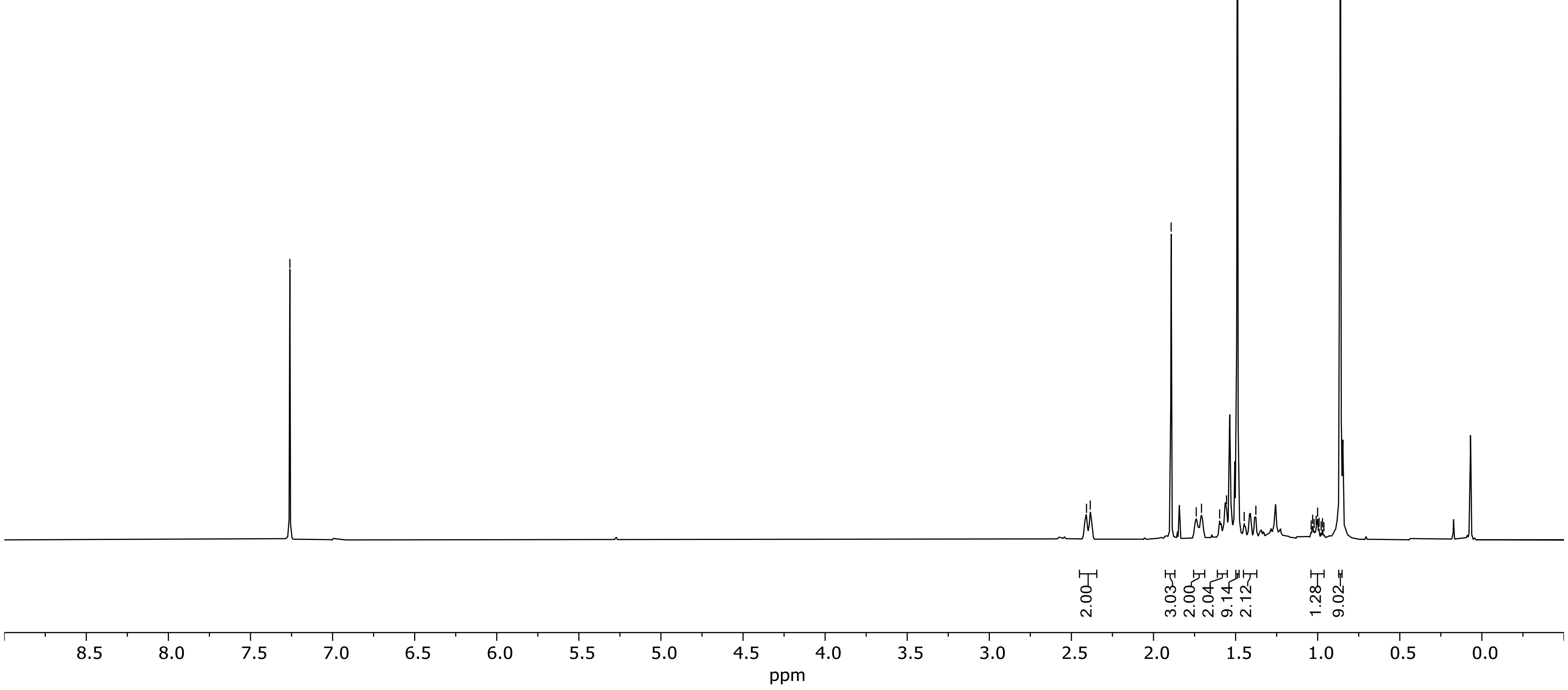

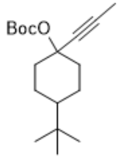

S12

Analysis: ^{13}C NMR

Solvent: $CDCl_3$

Field strength: 101 MHz

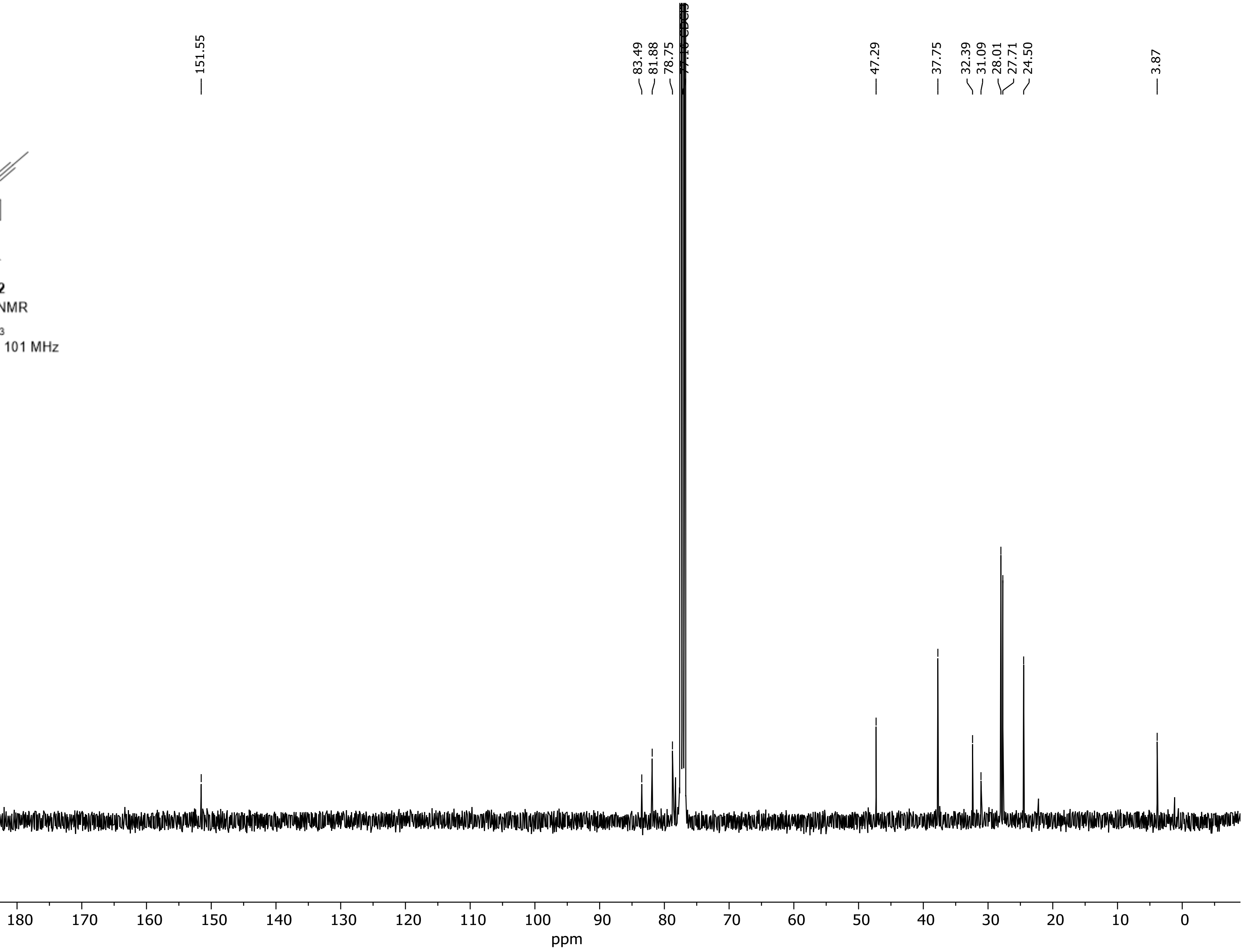

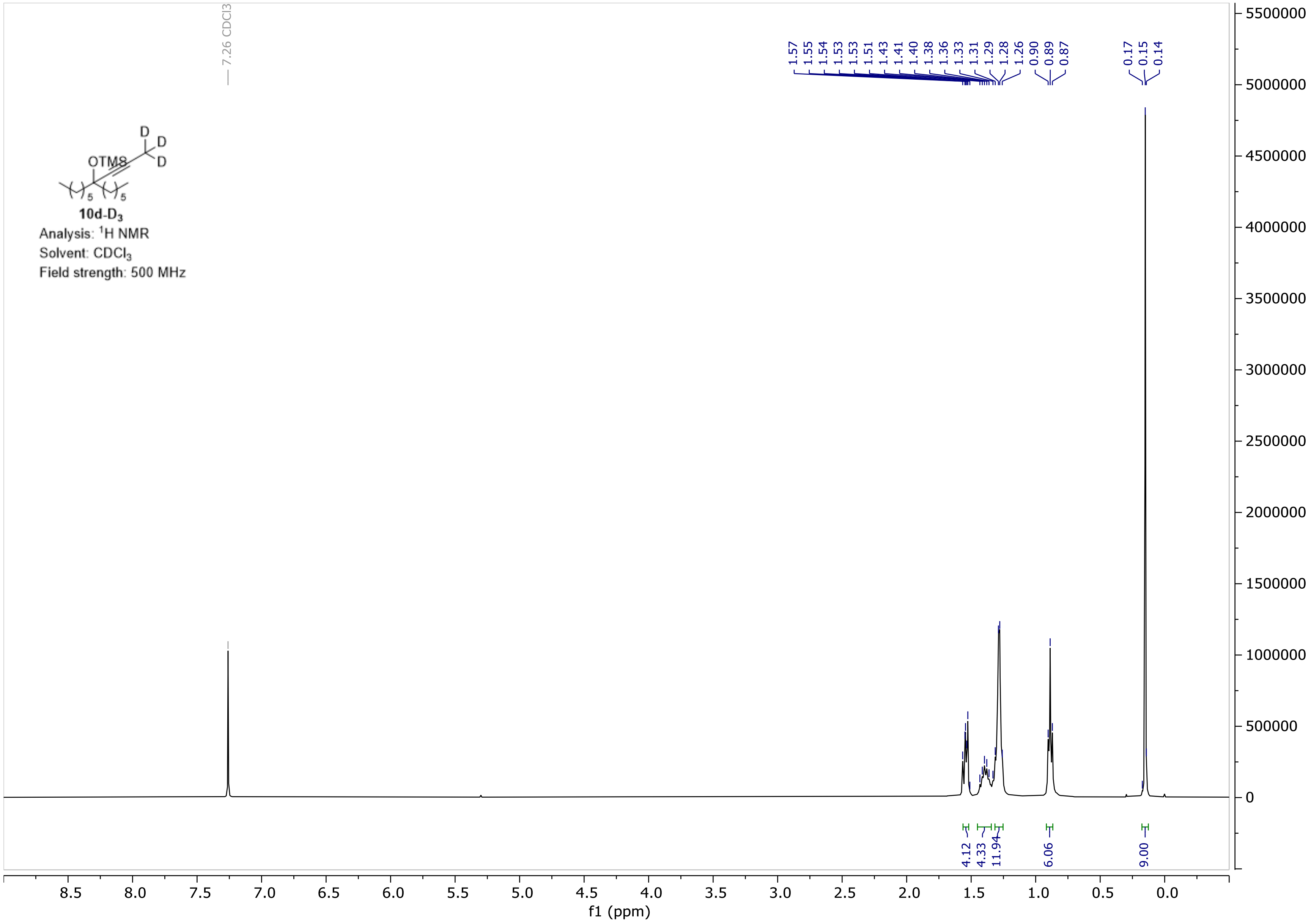

7.26 CDCl3
1.57 1.55 1.54 1.53 1.53 1.51 1.43 1.41 1.40 1.38 1.36 1.33 1.31 1.29 1.28 1.26 0.90 0.89 0.87
0.17 0.15 0.14
OTMS
D D D
5 5
10d-D3
Analysis: 1H NMR
Solvent: CDCl3
Field strength: 500 MHz
4.12 4.33 11.94 6.06 9.00
5500000 5000000 4500000 4000000 3500000 3000000 2500000 2000000 1500000 1000000 500000 0
8.5 8.0 7.5 7.0 6.5 6.0 5.5 5.0 4.5 4.0 3.5 3.0 2.5 2.0 1.5 1.0 0.5 0.0
f1 (ppm)

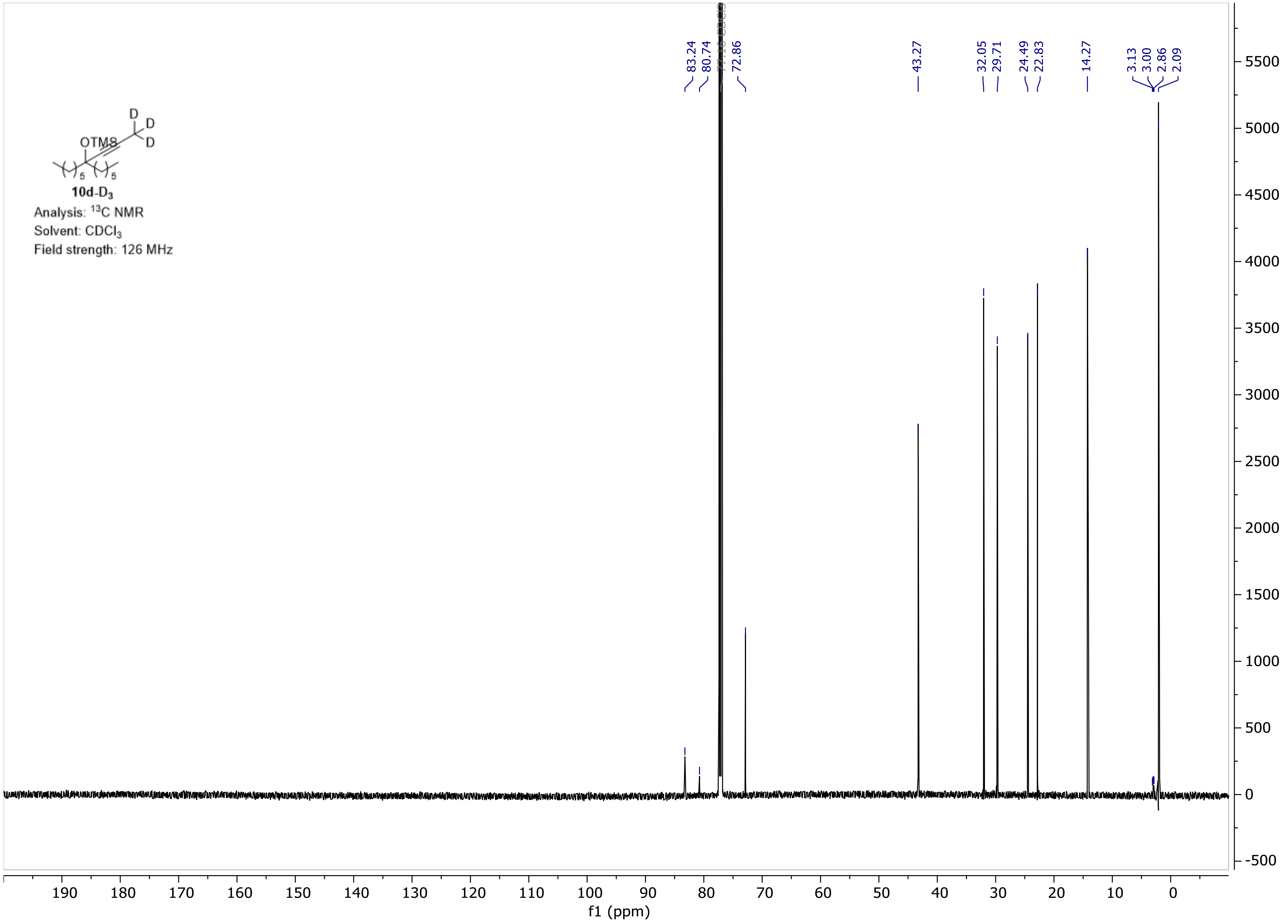
83.24
80.74
72.86
43.27
32.05
29.71
24.49
22.83
14.27
3.13
3.00
2.86
2.09
D
D
D
OTMS
5
5
10d-D3
Analysis: 13C NMR
Solvent: CDCl3
Field strength: 126 MHz
f1 (ppm)

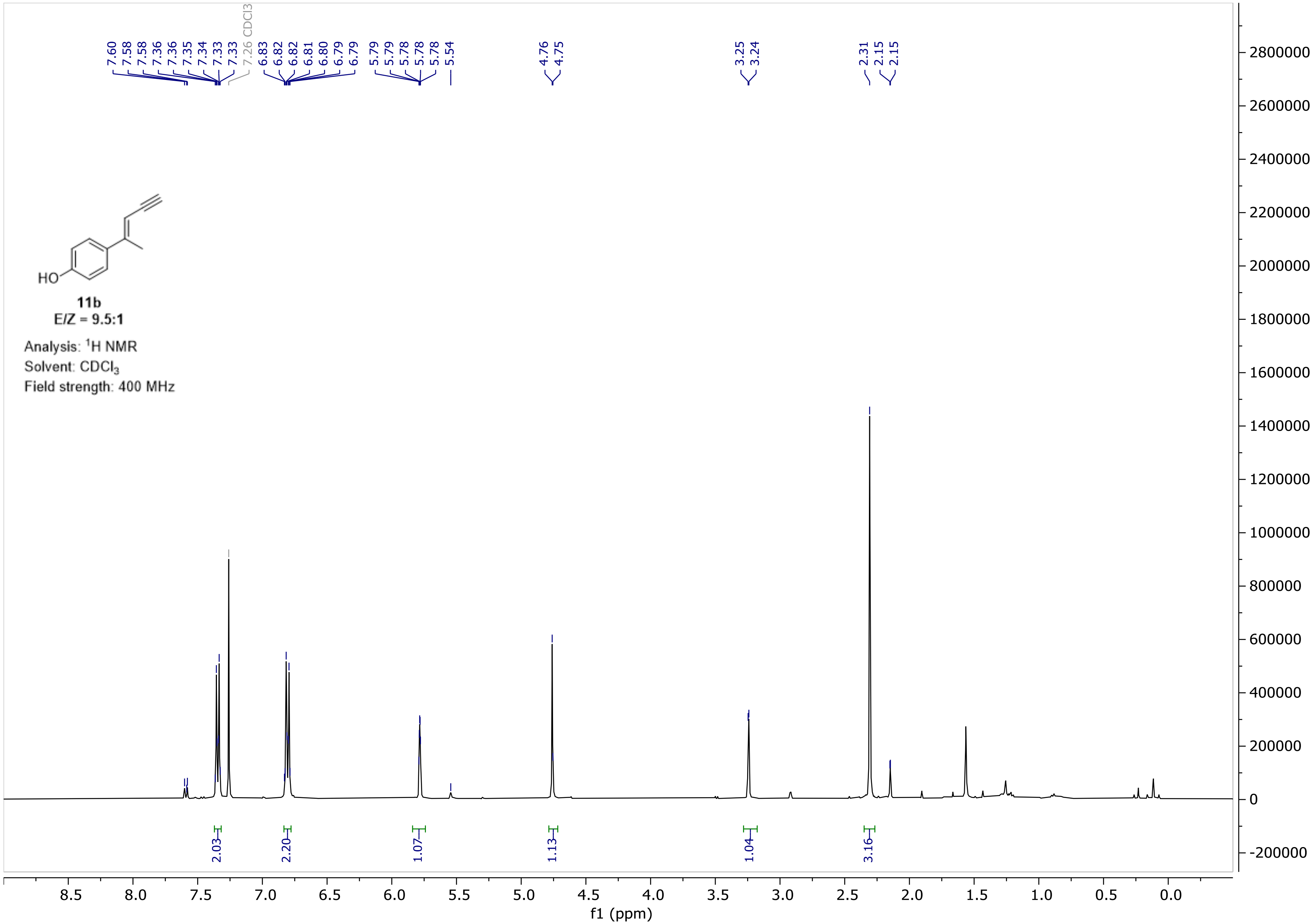

11b
E/Z = 9.5:1

Analysis: ^{1}H NMR
Solvent: $CDCl_3$
Field strength: 400 MHz

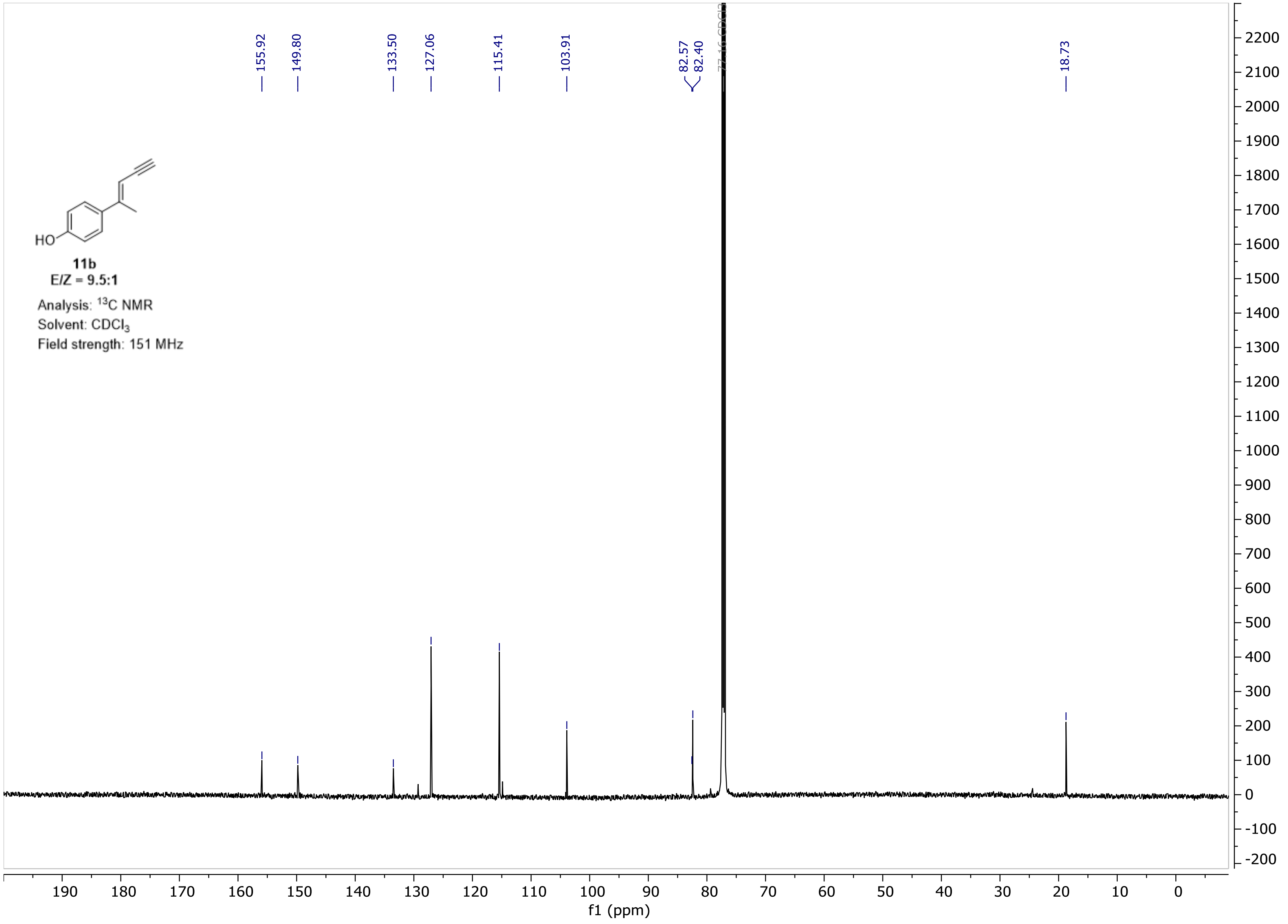
155.92
149.80
133.50
127.06
115.41
103.91
82.57
82.40
77.16 CDCl3
18.73
HO
11b
E/Z = 9.5:1
Analysis: 13C NMR
Solvent: CDCl3
Field strength: 151 MHz
f1 (ppm)

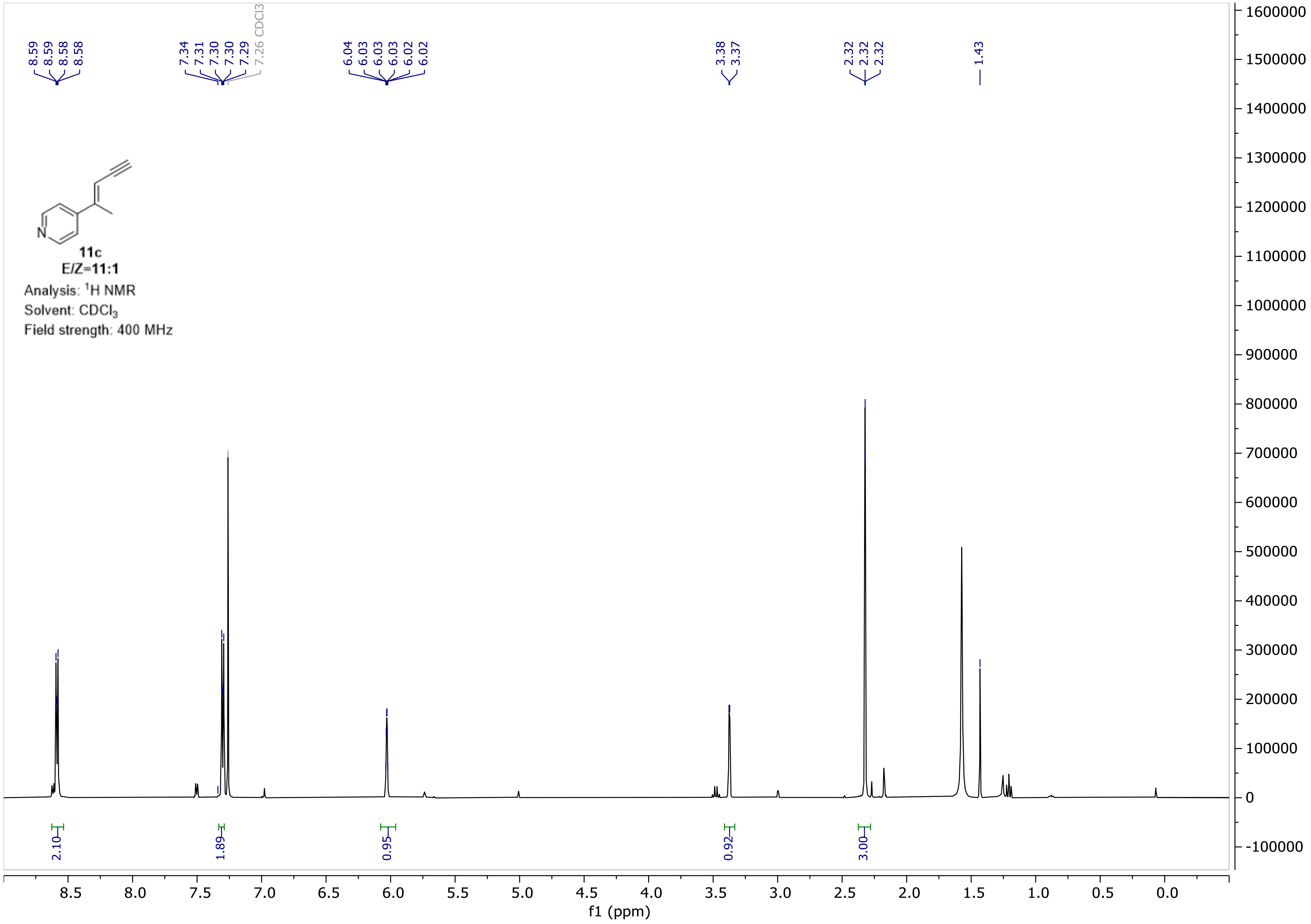

8.59
8.59
8.58
8.58
7.34
7.31
7.30
7.30
7.29
7.26 CDCl3
6.04
6.03
6.03
6.03
6.02
6.02
3.38
3.37
2.32
2.32
2.32
1.43
N
11c
E/Z=11:1
Analysis: 1H NMR
Solvent: CDCl3
Field strength: 400 MHz
2.10
1.89
0.95
0.92
3.00
f1 (ppm)

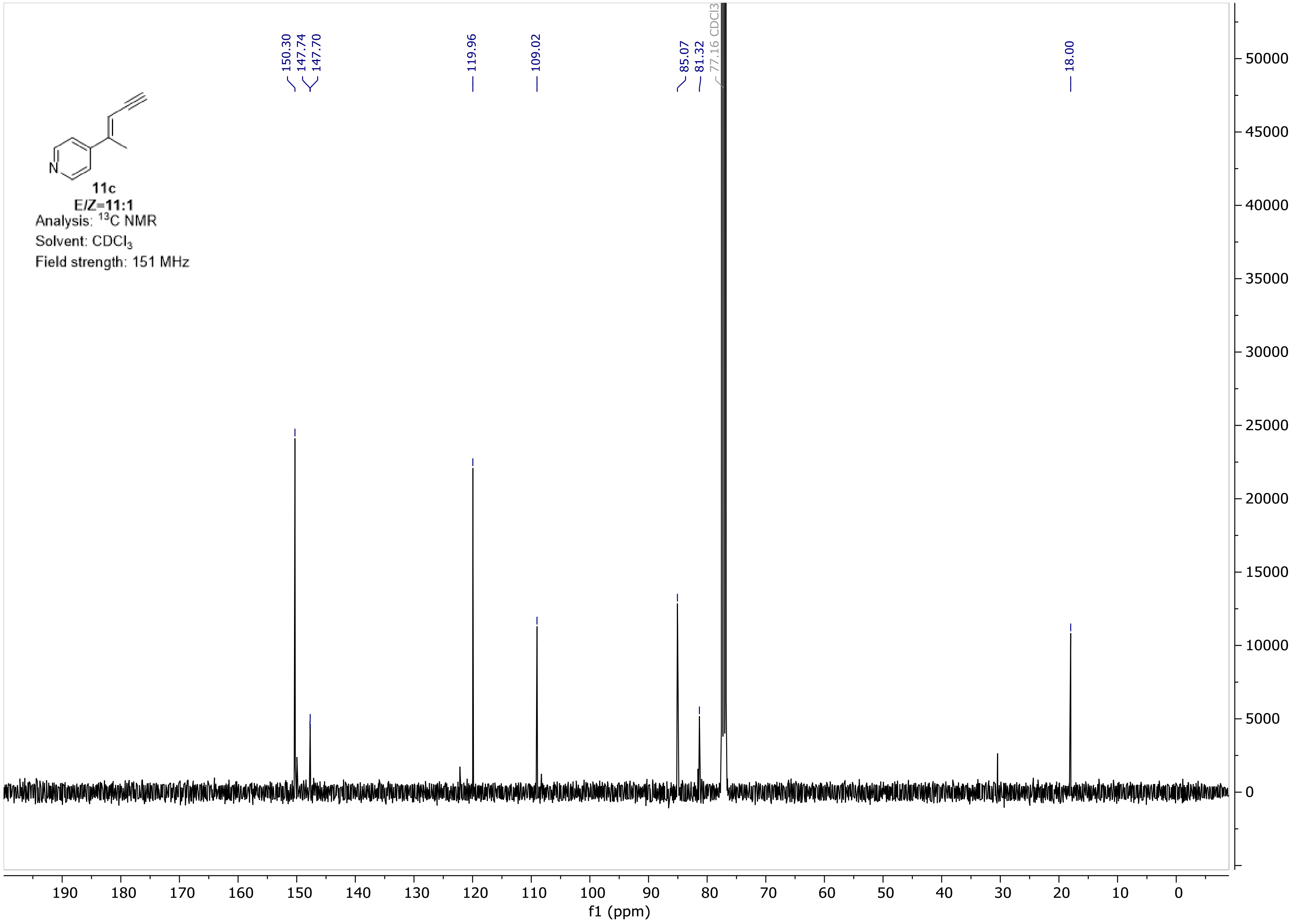
150.30
147.74
147.70
119.96
109.02
85.07
81.32
77.16 CDCl3
18.00
11c
E/Z=11:1
Analysis: ^{13}C NMR
Solvent: $CDCl_3$
Field strength: 151 MHz
N
f1 (ppm)
190 180 170 160 150 140 130 120 110 100 90 80 70 60 50 40 30 20 10 0
50000 45000 40000 35000 30000 25000 20000 15000 10000 5000 0

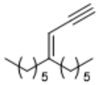

11d

Analysis: ^{1}H NMR
Solvent: $CDCl_3$
Field strength: 400 MHz

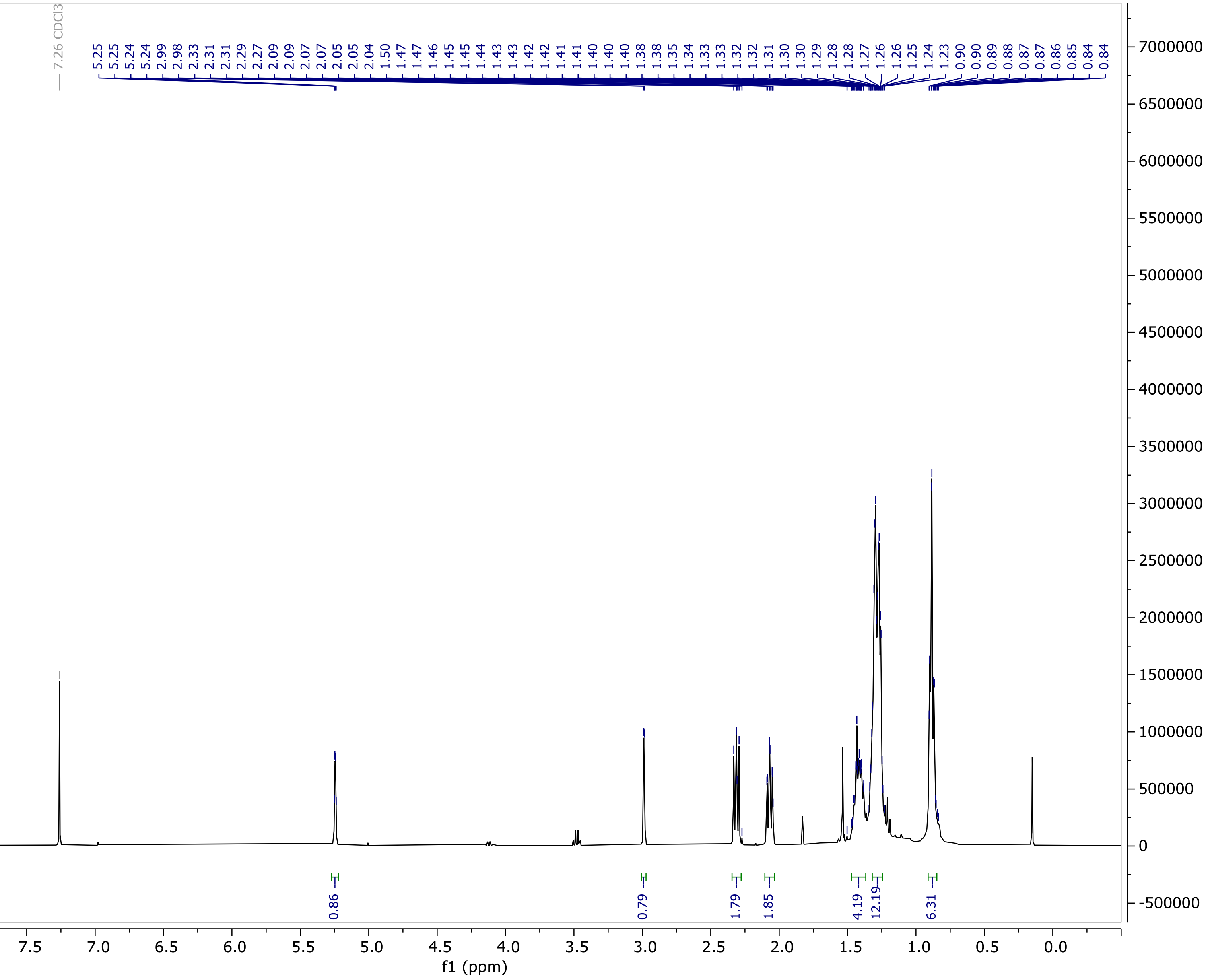

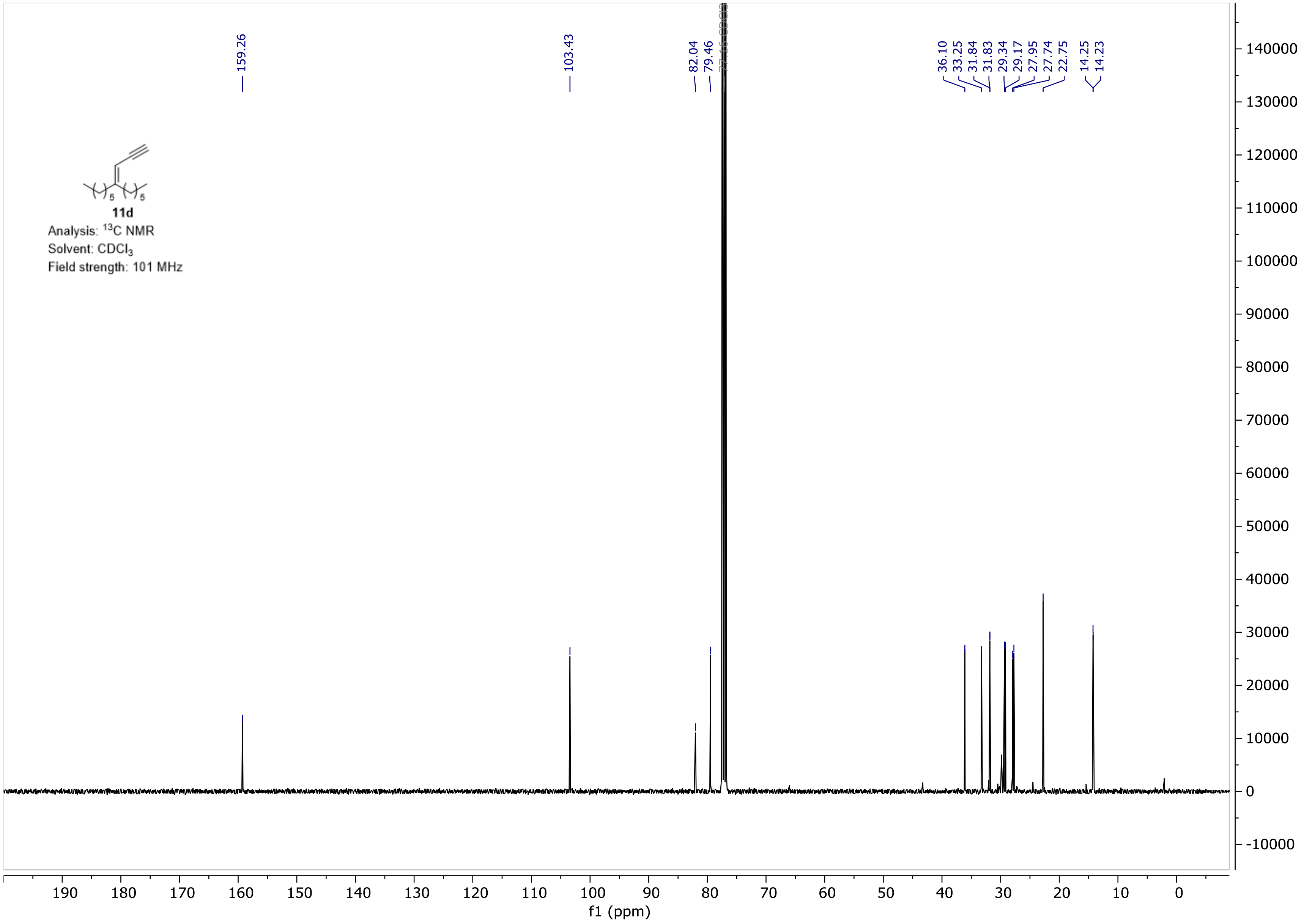
159.26
103.43
82.04
79.46
36.10
33.25
31.84
31.83
29.34
29.17
27.95
27.74
22.75
14.25
14.23
11d
Analysis: ¹³C NMR
Solvent: CDCl3
Field strength: 101 MHz
190 180 170 160 150 140 130 120 110 100 90 80 70 60 50 40 30 20 10 0
f1 (ppm)
140000 130000 120000 110000 100000 90000 80000 70000 60000 50000 40000 30000 20000 10000 0 -10000

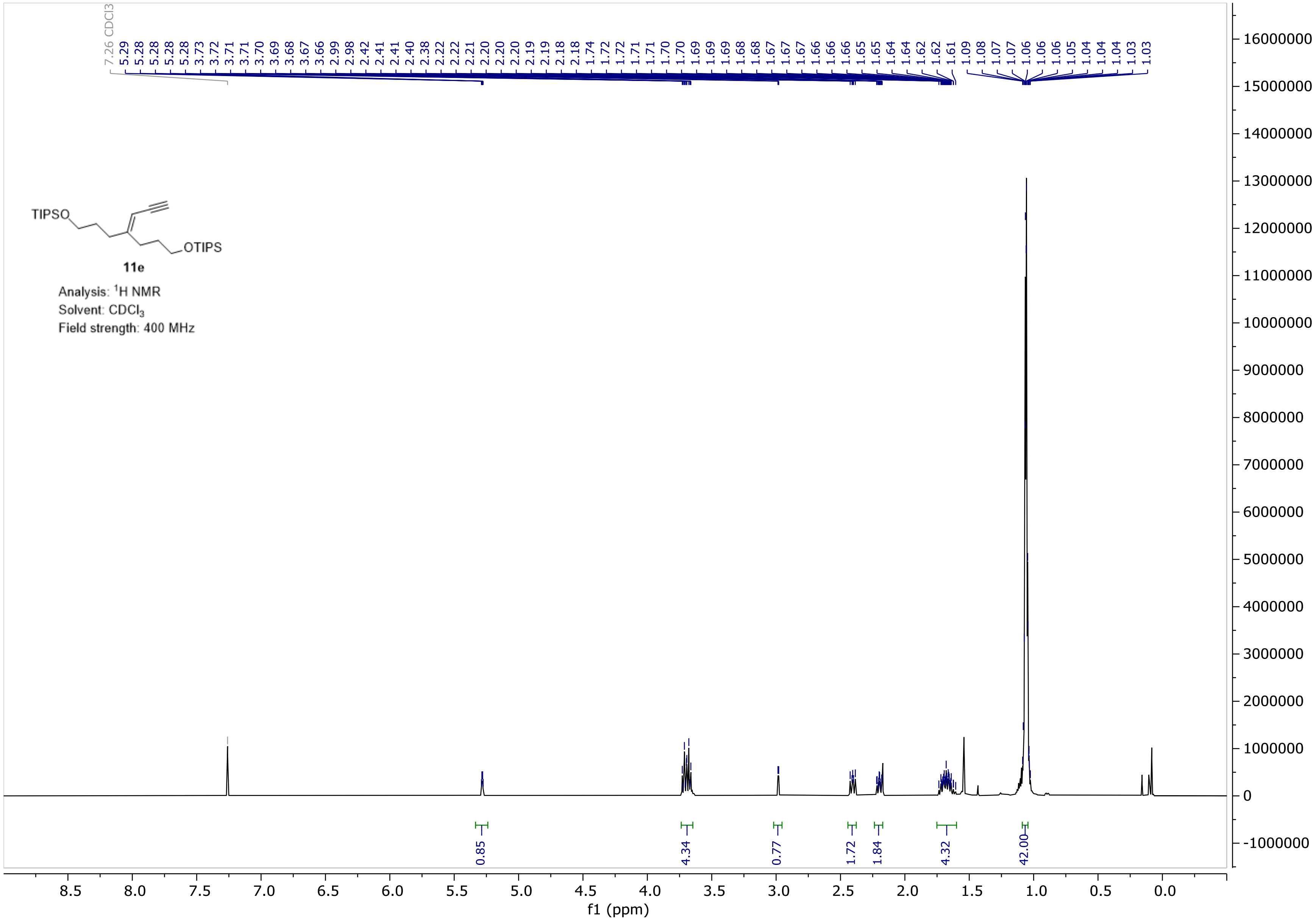
TIPSO
OTIPS
11e
Analysis: ¹H NMR
Solvent: CDCl3
Field strength: 400 MHz
7.26 CDCl3
f1 (ppm)
0.85
4.34
0.77
1.72
1.84
4.32
42.00

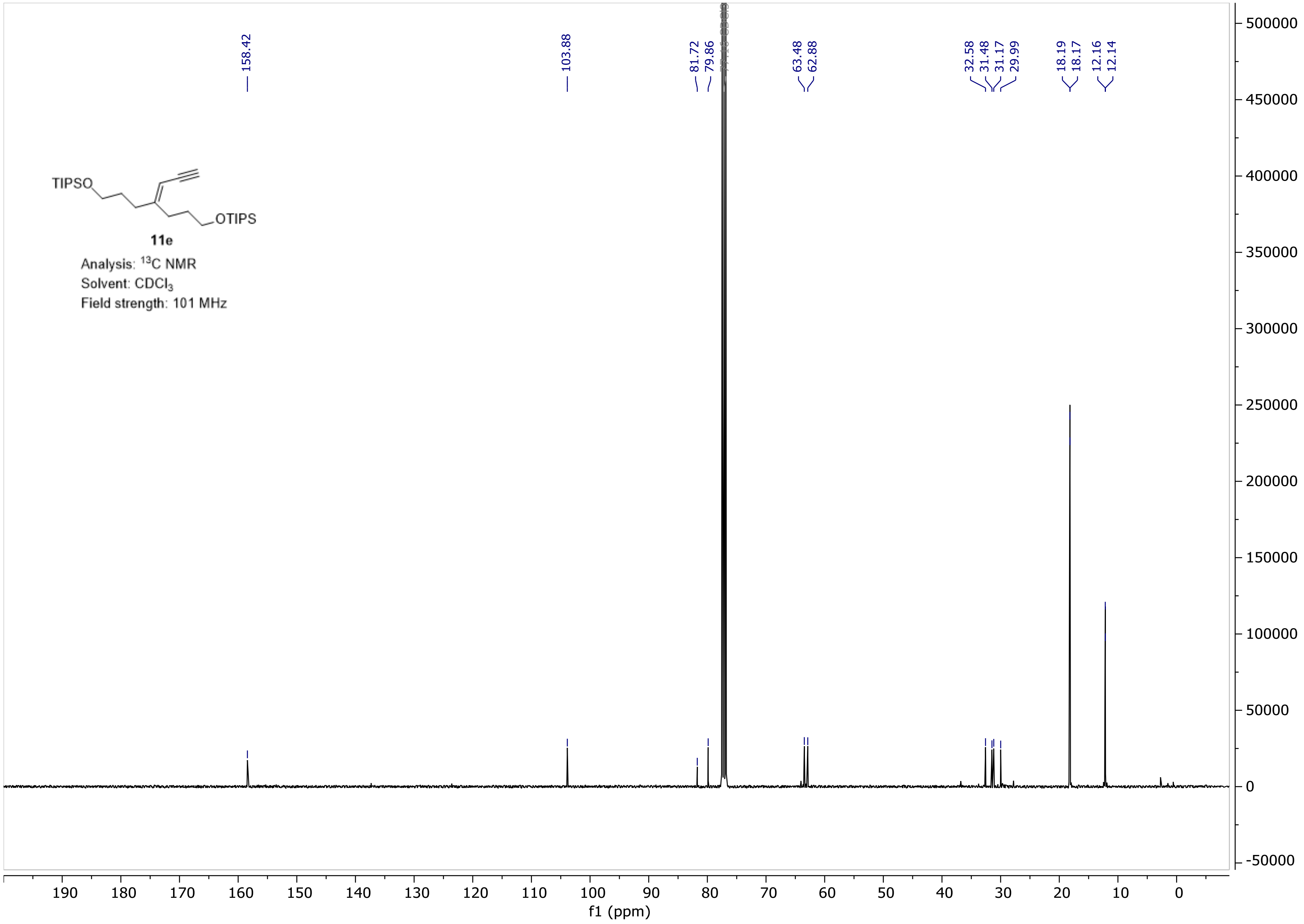

158.42
103.88
81.72
79.86
63.48
62.88
32.58
31.48
31.17
29.99
18.19
18.17
12.16
12.14
TIPSO
OTIPS
11e
Analysis: ^{13}C NMR
Solvent: $CDCl_3$
Field strength: 101 MHz
f1 (ppm)

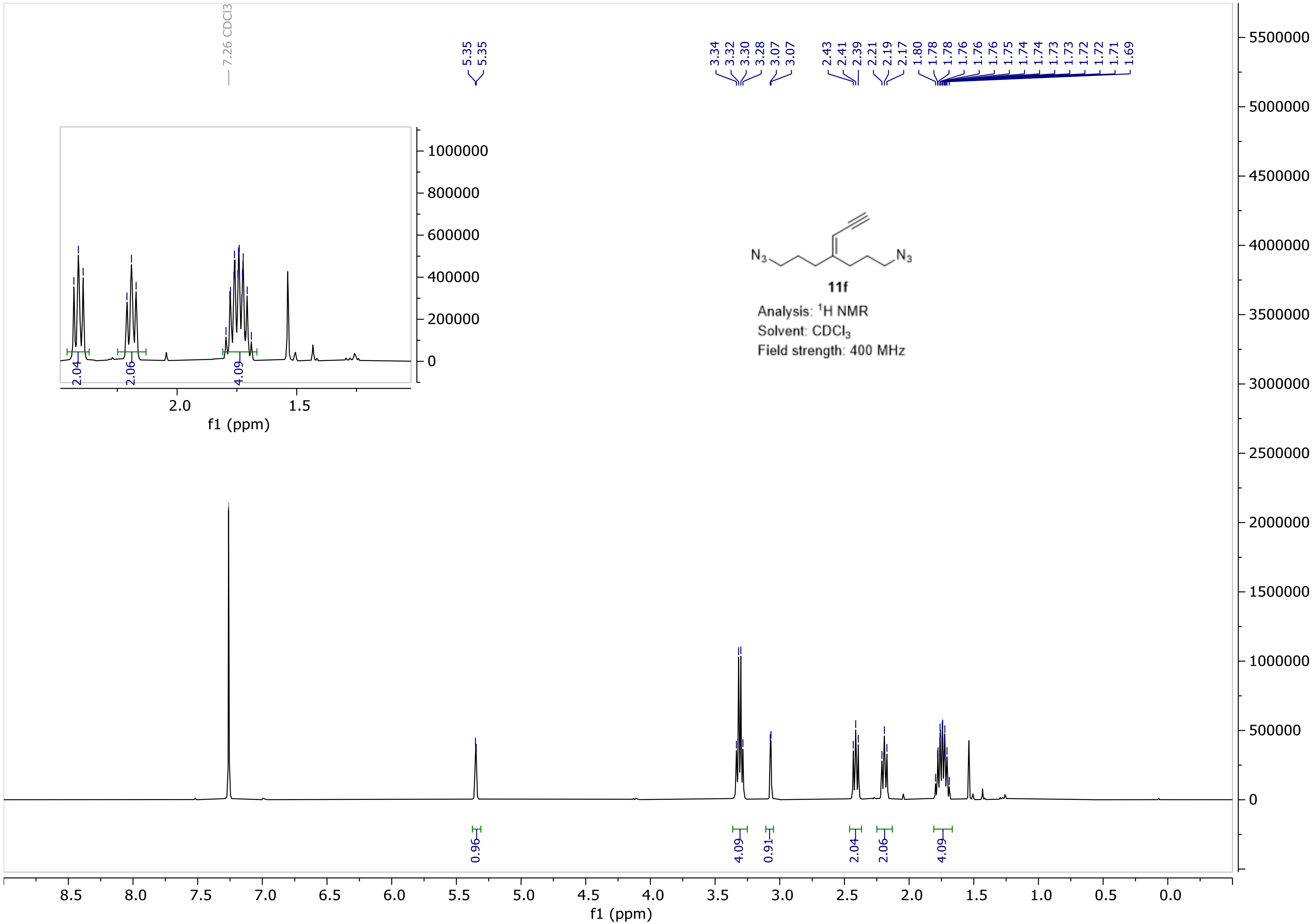

11f
Analysis: ¹H NMR
Solvent: CDCl3
Field strength: 400 MHz
f1 (ppm)

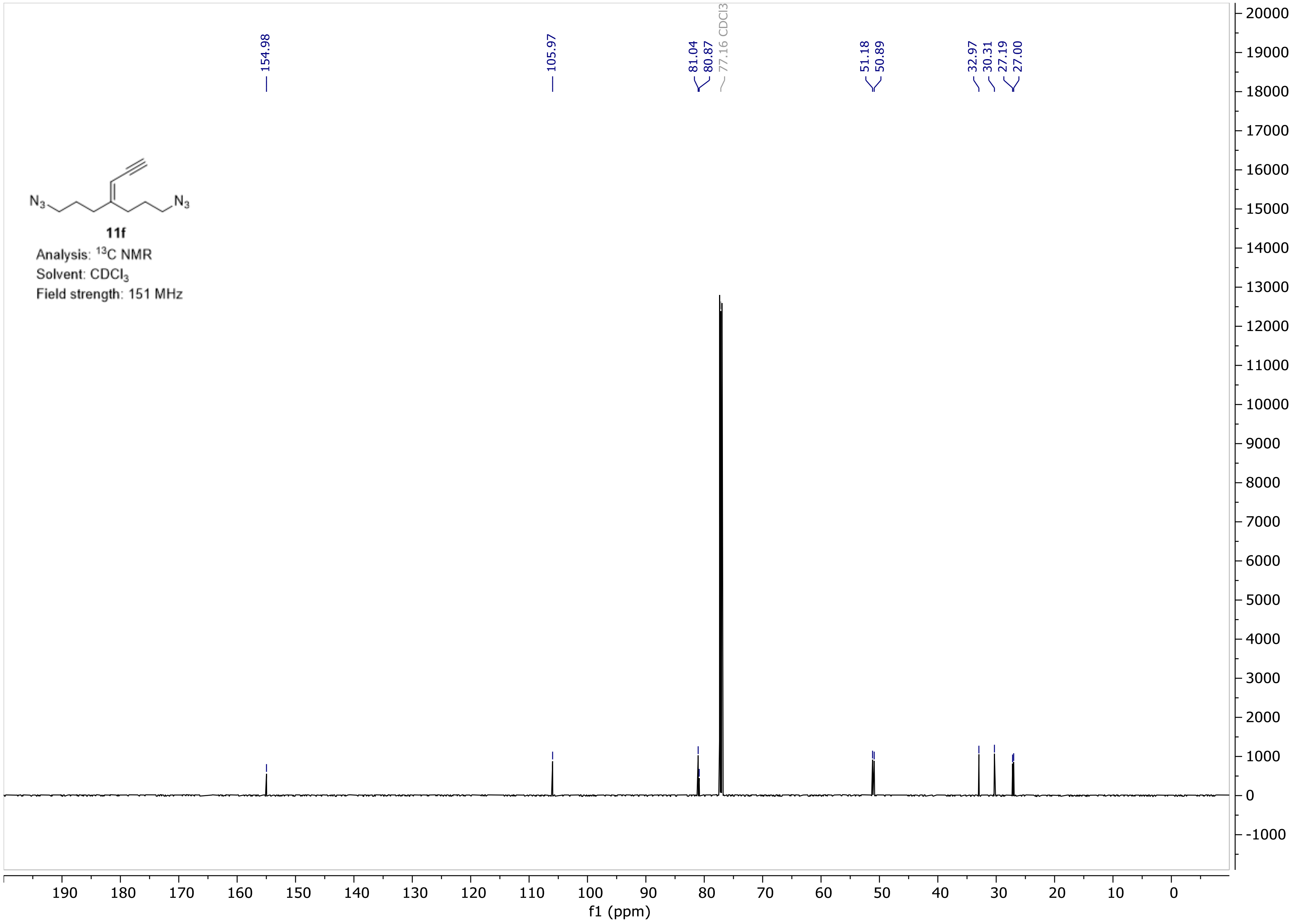
154.98
105.97
81.04
80.87
77.16 CDCl3
51.18
50.89
32.97
30.31
27.19
27.00
N3
N3
11f
Analysis: 13C NMR
Solvent: CDCl3
Field strength: 151 MHz
f1 (ppm)

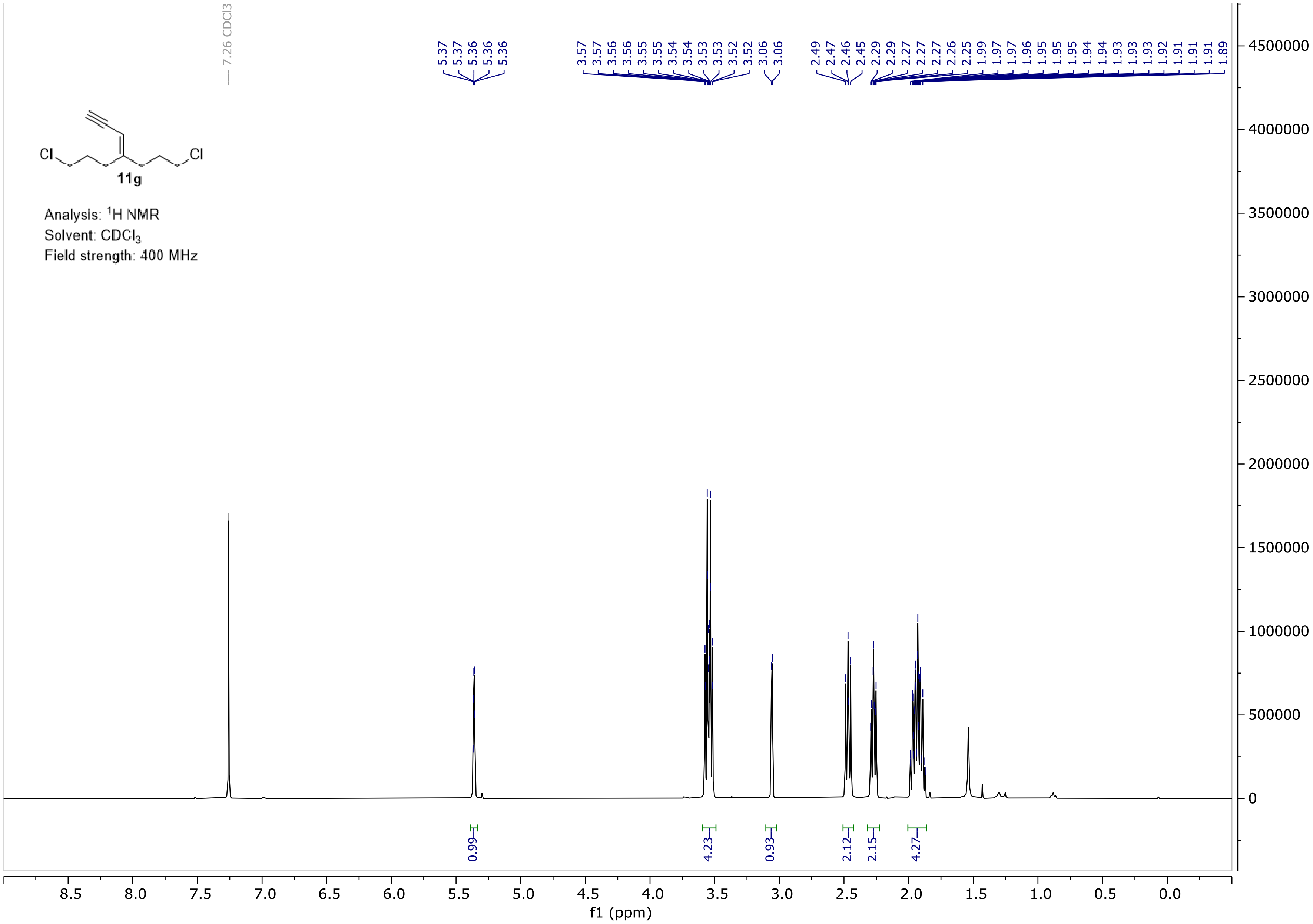

11g
Analysis: 1H NMR
Solvent: CDCl3
Field strength: 400 MHz
7.26 CDCl3
5.37
5.37
5.36
5.36
5.36
3.57
3.57
3.56
3.56
3.55
3.55
3.54
3.54
3.53
3.53
3.52
3.52
3.06
3.06
2.49
2.47
2.46
2.45
2.29
2.29
2.27
2.27
2.27
2.26
2.25
1.99
1.97
1.97
1.96
1.95
1.95
1.95
1.94
1.94
1.93
1.93
1.93
1.92
1.91
1.91
1.91
1.89
0.99
4.23
0.93
2.12
2.15
4.27
f1 (ppm)

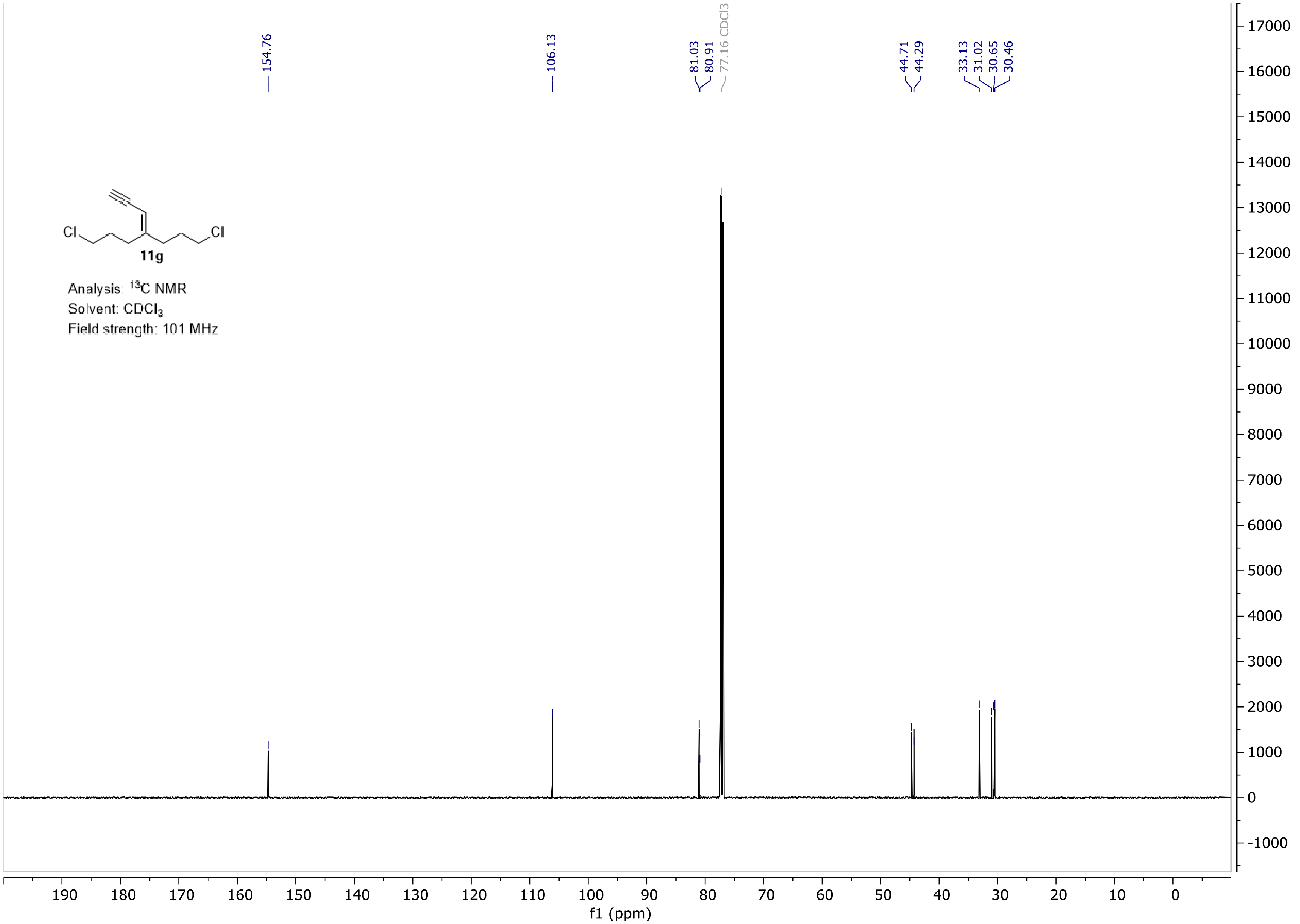
154.76
106.13
81.03
80.91
77.16 CDCl3
44.71
44.29
33.13
31.02
30.65
30.46
Cl
Cl
11g
Analysis: 13C NMR
Solvent: CDCl3
Field strength: 101 MHz
f1 (ppm)

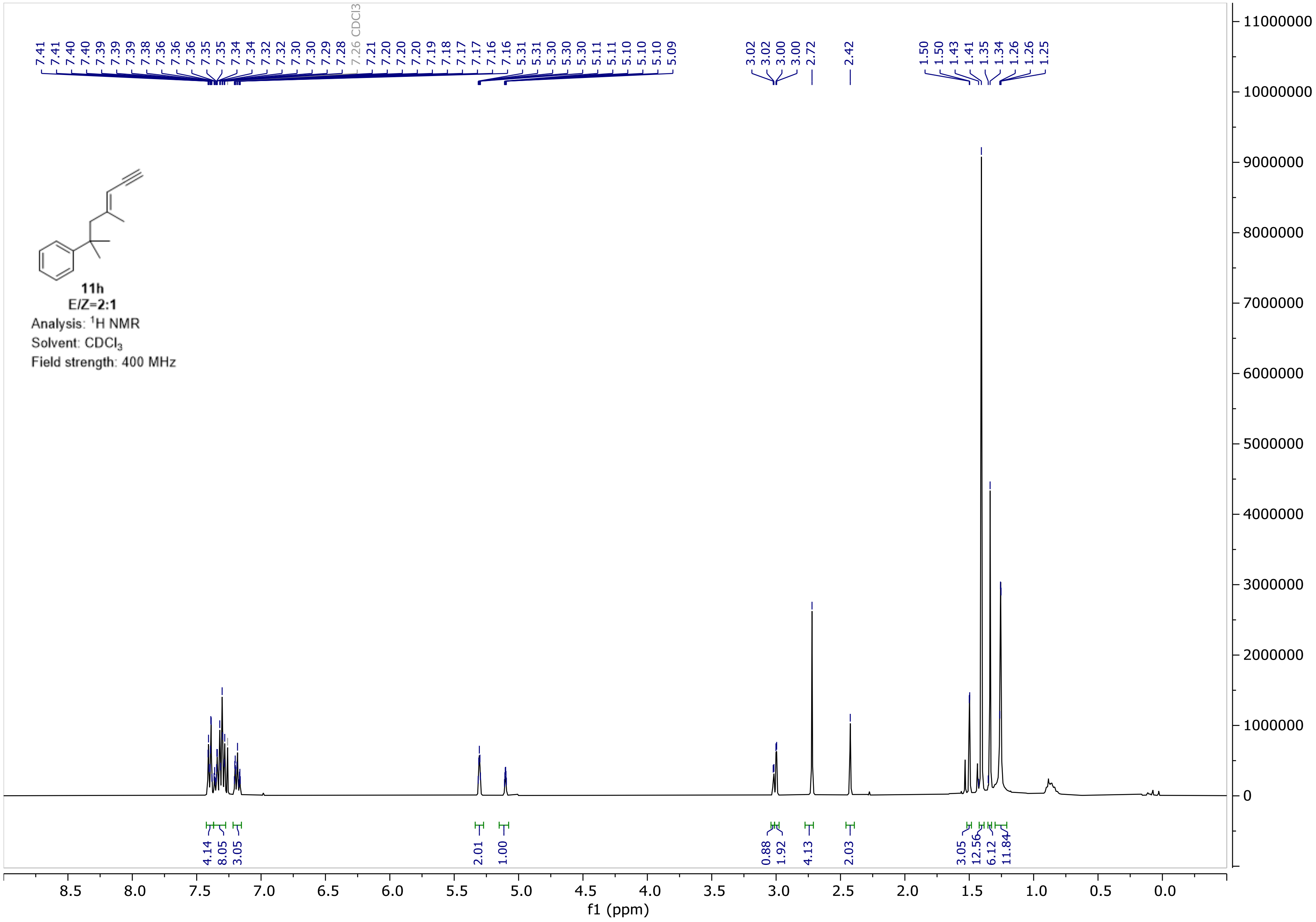

11h
E/Z=2:1
Analysis: 1H NMR
Solvent: CDCl3
Field strength: 400 MHz
f1 (ppm)

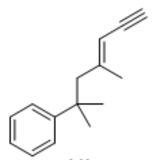

11h
E/Z=2:1

Analysis: ^{13}C NMR
Solvent: $CDCl_3$
Field strength: 151 MHz

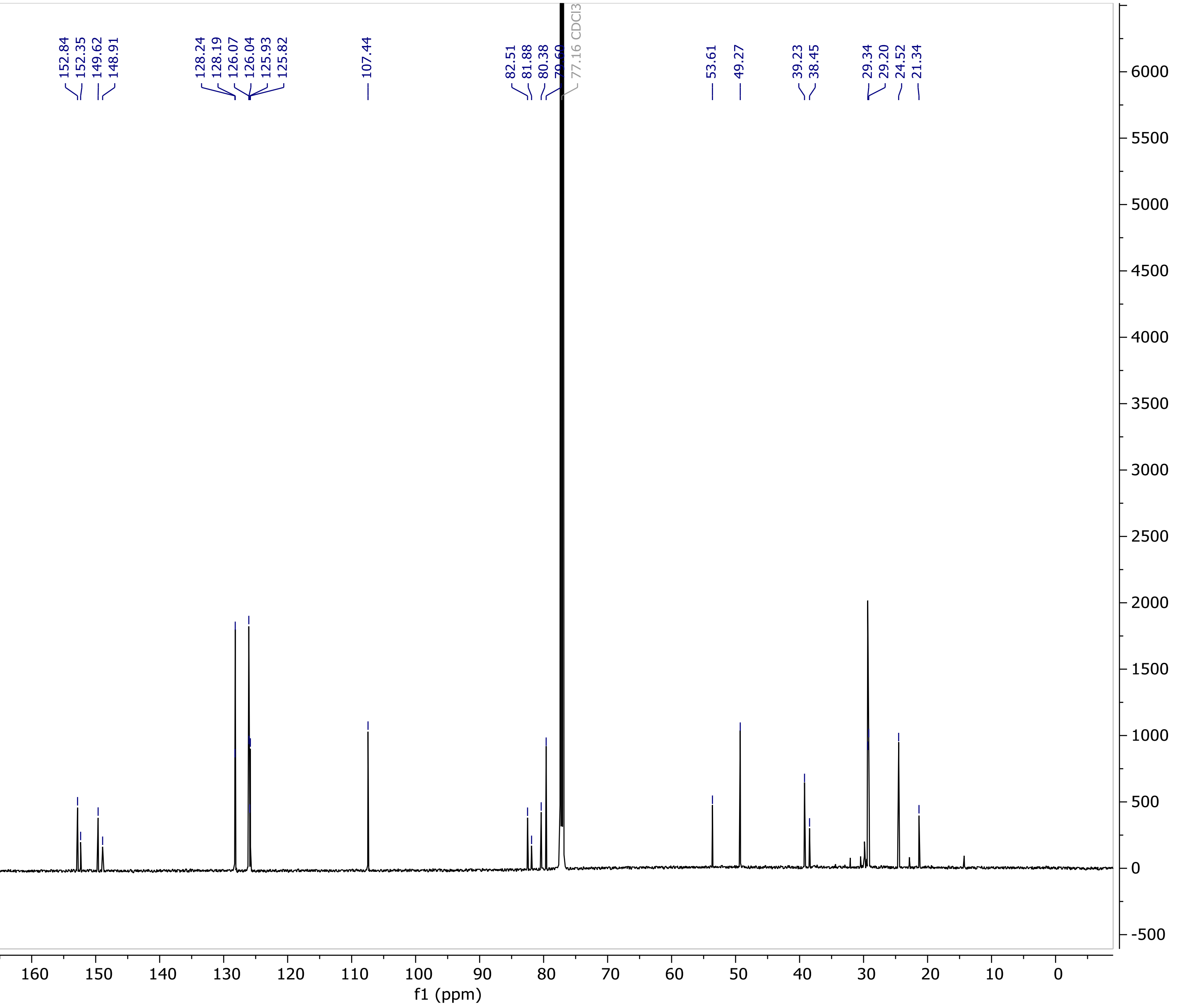

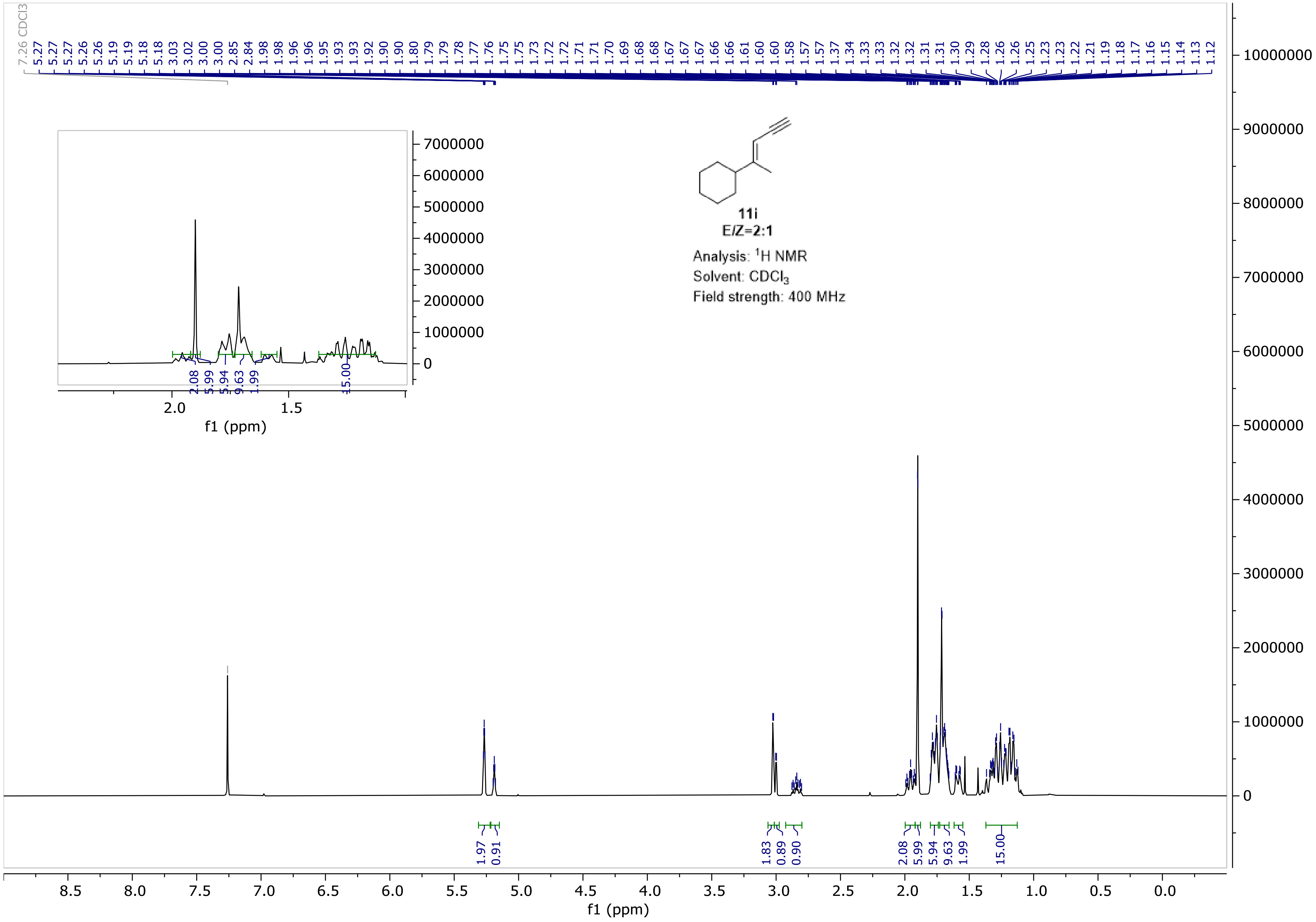
7.26 CDCl3
5.27 5.27 5.27 5.26 5.26 5.19 5.19 5.18 5.18 3.03 3.02 3.00 3.00 2.85 2.84 1.98 1.98 1.96 1.96 1.95 1.93 1.93 1.92 1.90 1.90 1.80 1.79 1.79 1.78 1.77 1.76 1.75 1.75 1.73 1.72 1.72 1.71 1.71 1.70 1.69 1.68 1.67 1.67 1.67 1.66 1.66 1.61 1.60 1.60 1.58 1.57 1.57 1.37 1.34 1.33 1.33 1.32 1.32 1.31 1.31 1.30 1.29 1.28 1.26 1.26 1.25 1.23 1.23 1.22 1.21 1.19 1.18 1.17 1.16 1.15 1.14 1.13 1.12
11i
E/Z=2:1
Analysis: ¹H NMR
Solvent: CDCl3
Field strength: 400 MHz
2.08 5.99 5.94 9.63 1.99 15.00
1.97 0.91 1.83 0.89 0.90 2.08 5.99 5.94 9.63 1.99 15.00
f1 (ppm)

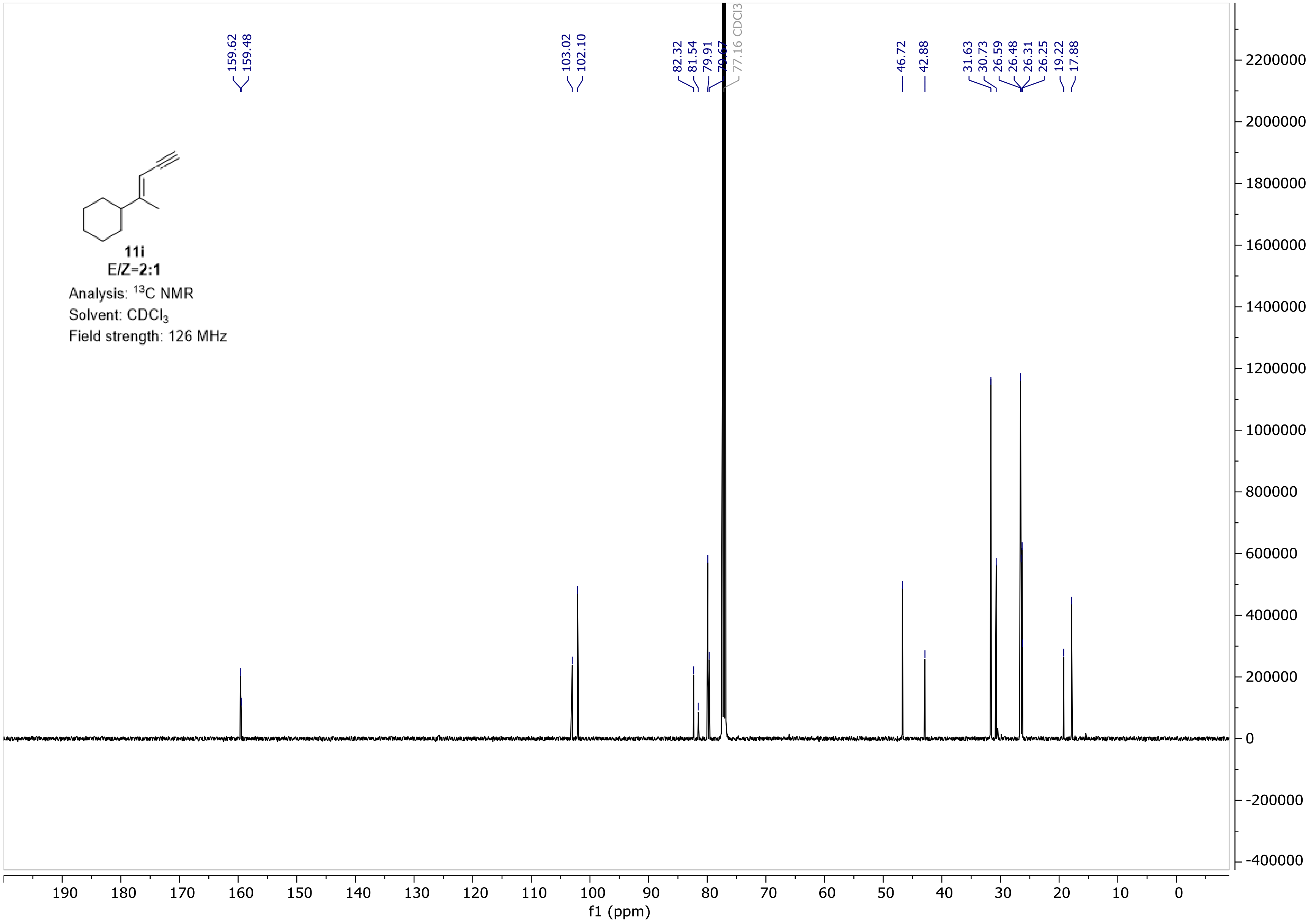

11i
E/Z=**2:1**
Analysis: ^{13}C NMR
Solvent: $CDCl_3$
Field strength: 126 MHz

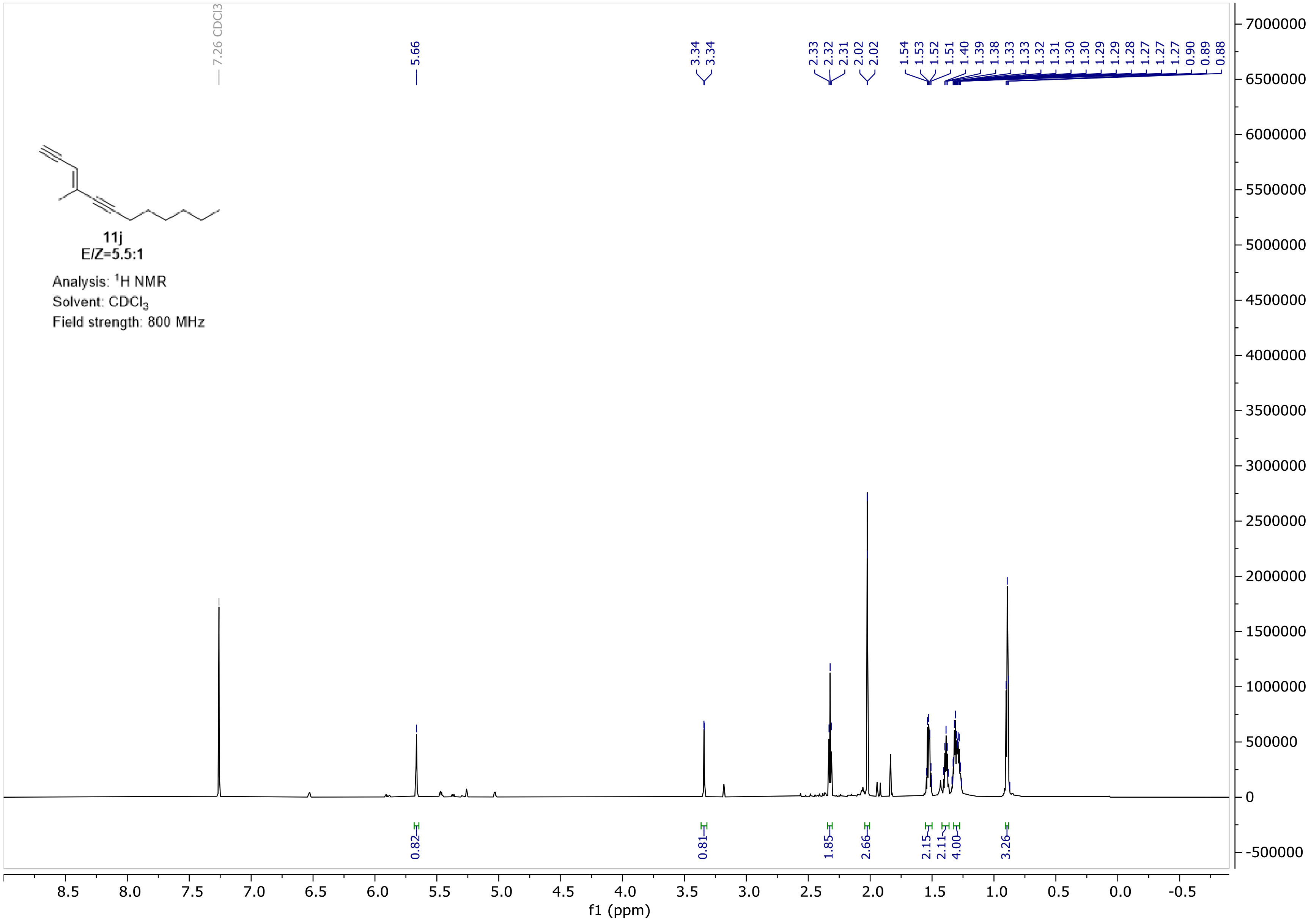

7.26 CDCl3
5.66
3.34
3.34
2.33
2.32
2.31
2.02
2.02
1.54
1.53
1.52
1.51
1.40
1.39
1.38
1.33
1.33
1.32
1.31
1.30
1.30
1.29
1.29
1.28
1.27
1.27
1.27
0.90
0.89
0.88
11j
E/Z=5.5:1
Analysis: ¹H NMR
Solvent: CDCl3
Field strength: 800 MHz
0.82
0.81
1.85
2.66
2.15
2.11
4.00
3.26
f1 (ppm)

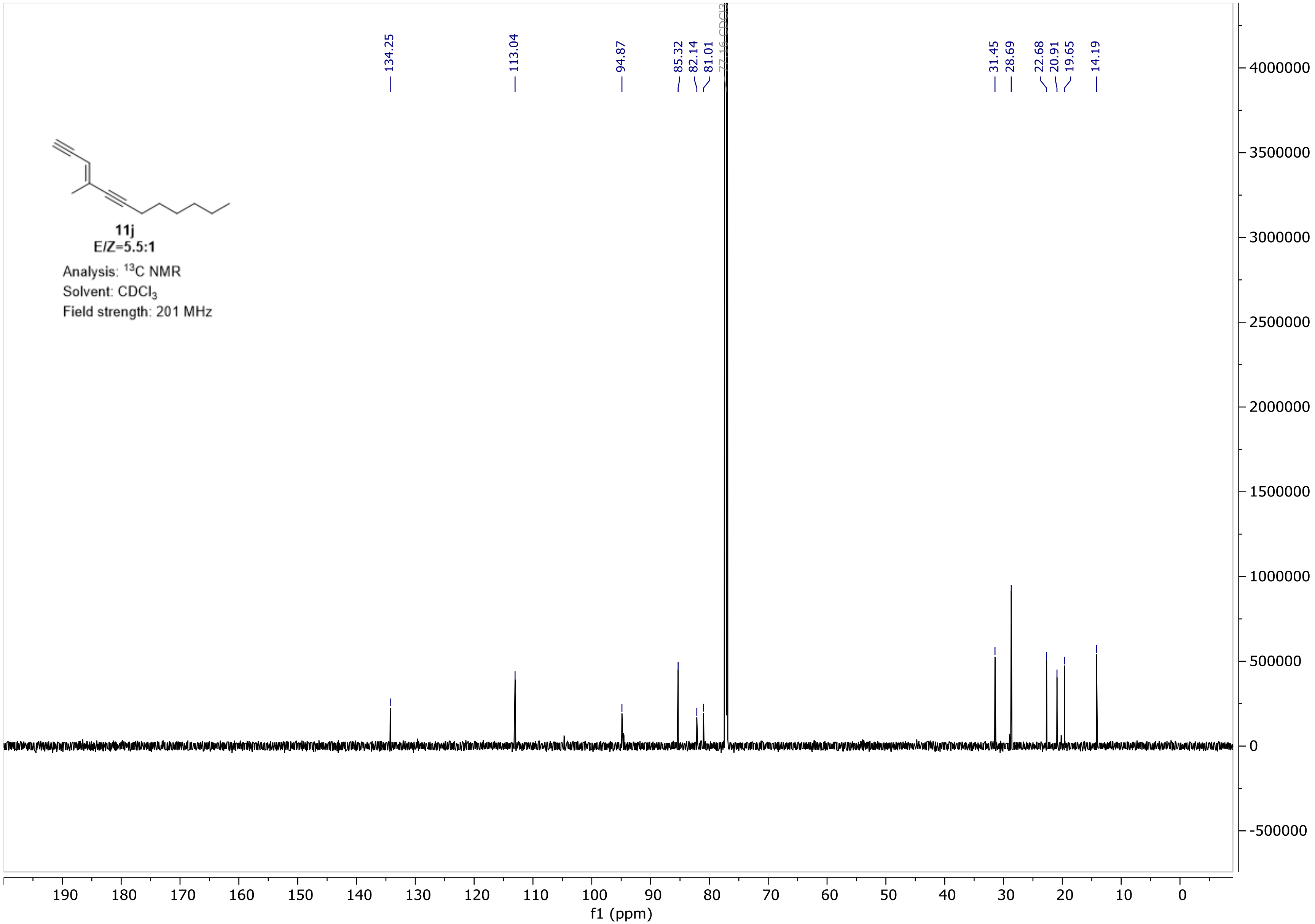
11j
E/Z=5.5:1
Analysis: 13C NMR
Solvent: CDCl3
Field strength: 201 MHz
134.25
113.04
94.87
85.32
82.14
81.01
77.16 CDCl3
31.45
28.69
22.68
20.91
19.65
14.19
4000000
3500000
3000000
2500000
2000000
1500000
1000000
500000
0
-500000
190
180
170
160
150
140
130
120
110
100
90
80
70
60
50
40
30
20
10
0
f1 (ppm)

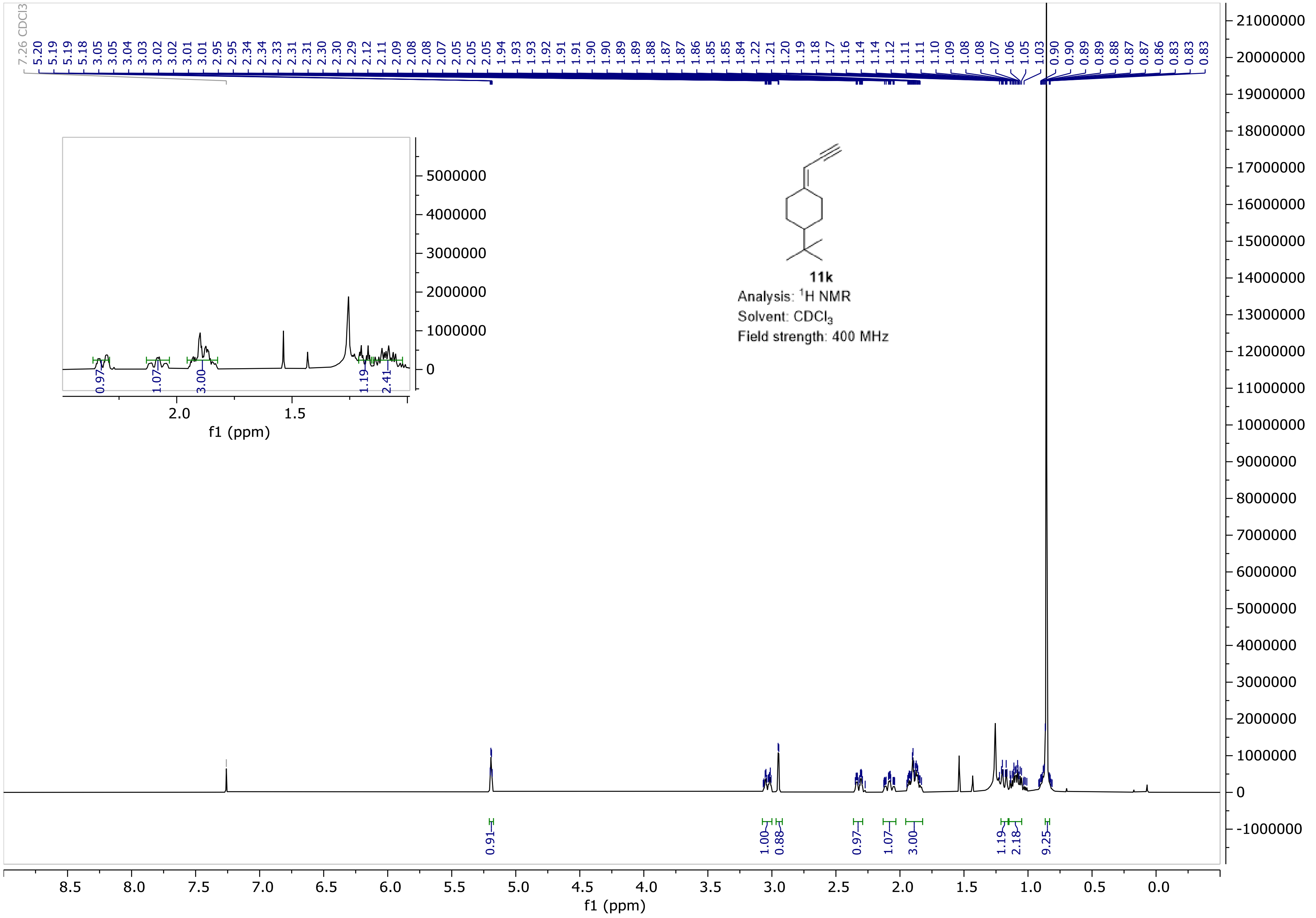
11k
Analysis: 1H NMR
Solvent: CDCl3
Field strength: 400 MHz
f1 (ppm)

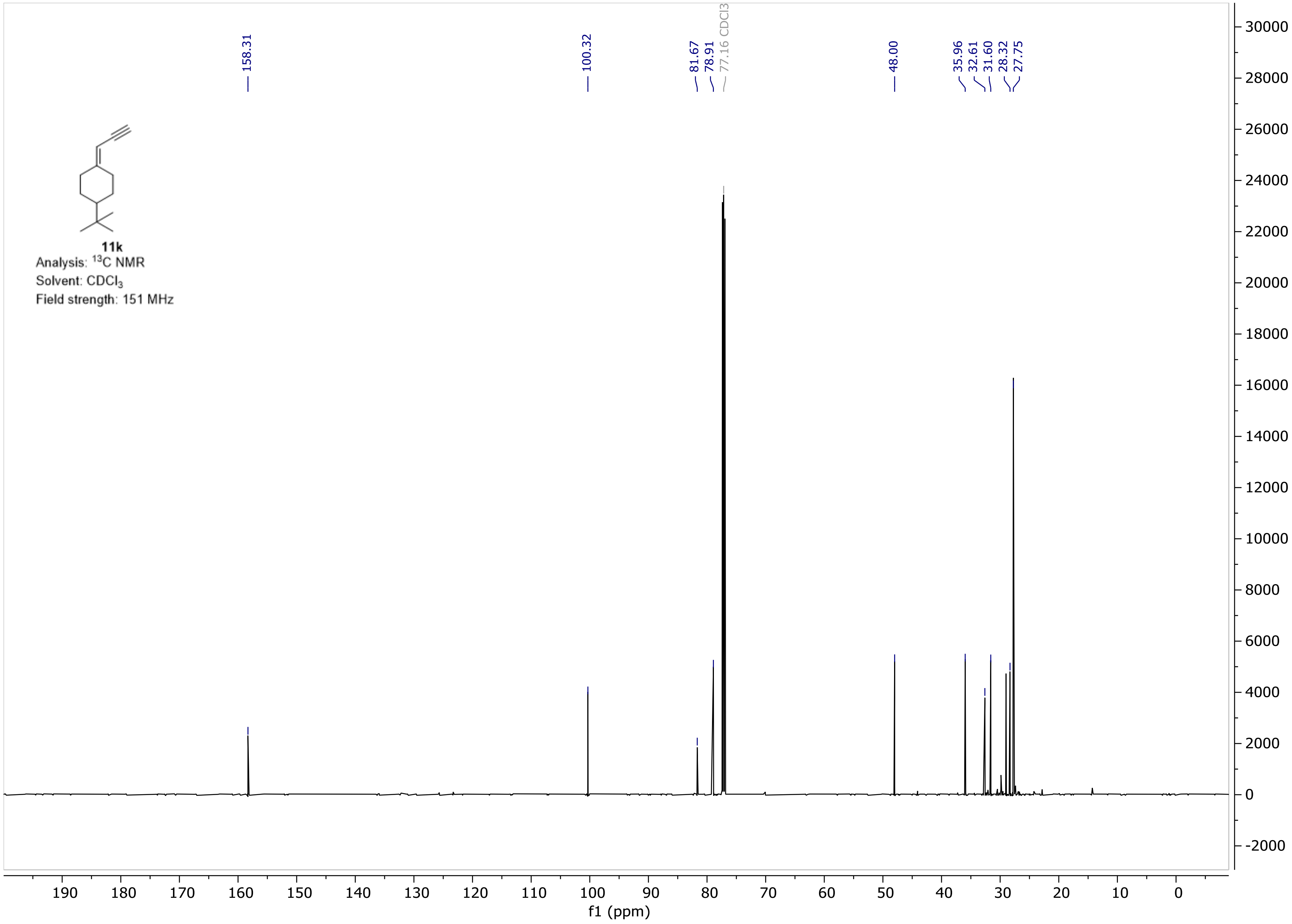

11k
Analysis: ^{13}C NMR
Solvent: $CDCl_3$
Field strength: 151 MHz

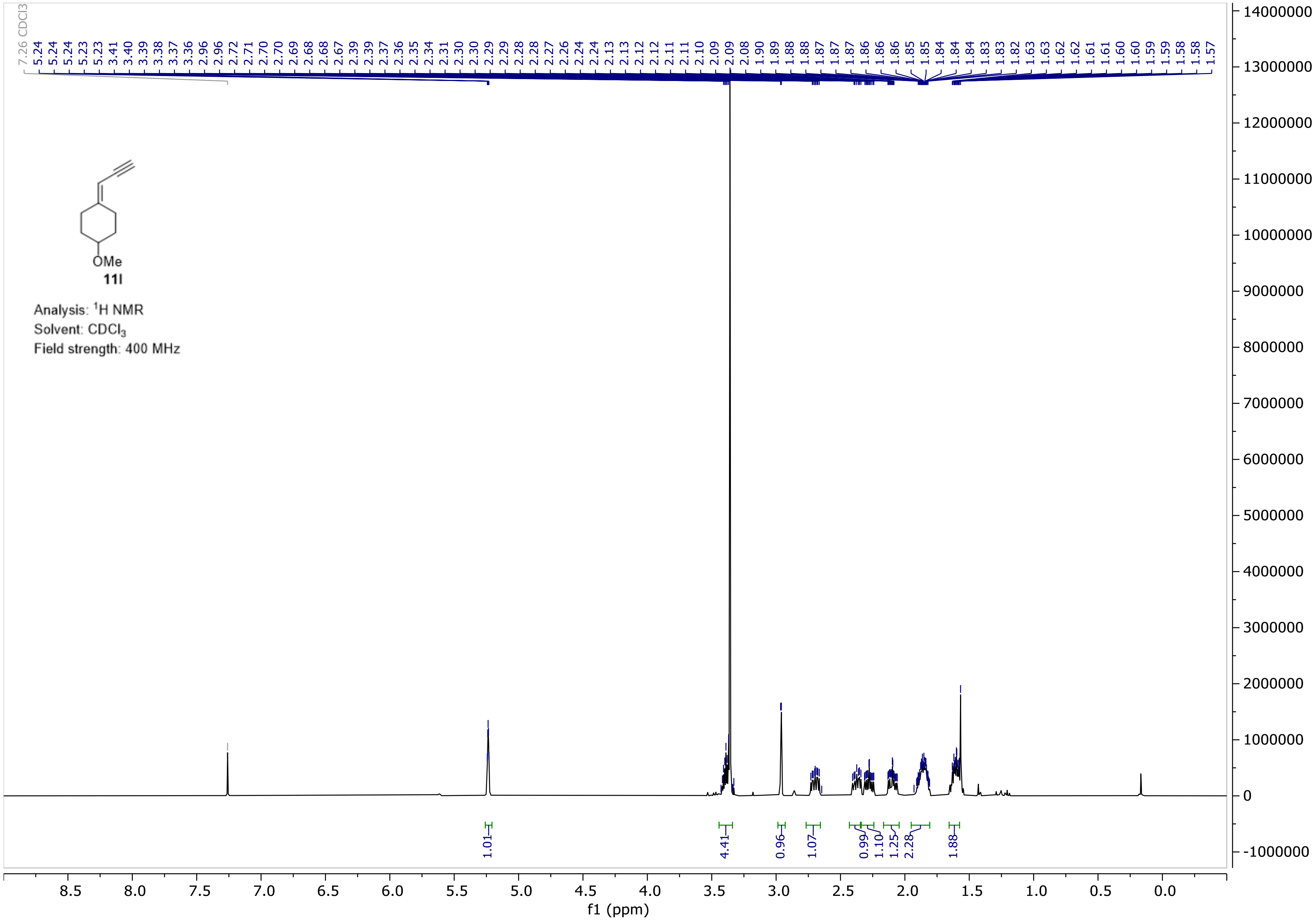

Analysis: ^{1}H NMR
Solvent: $CDCl_3$
Field strength: 400 MHz

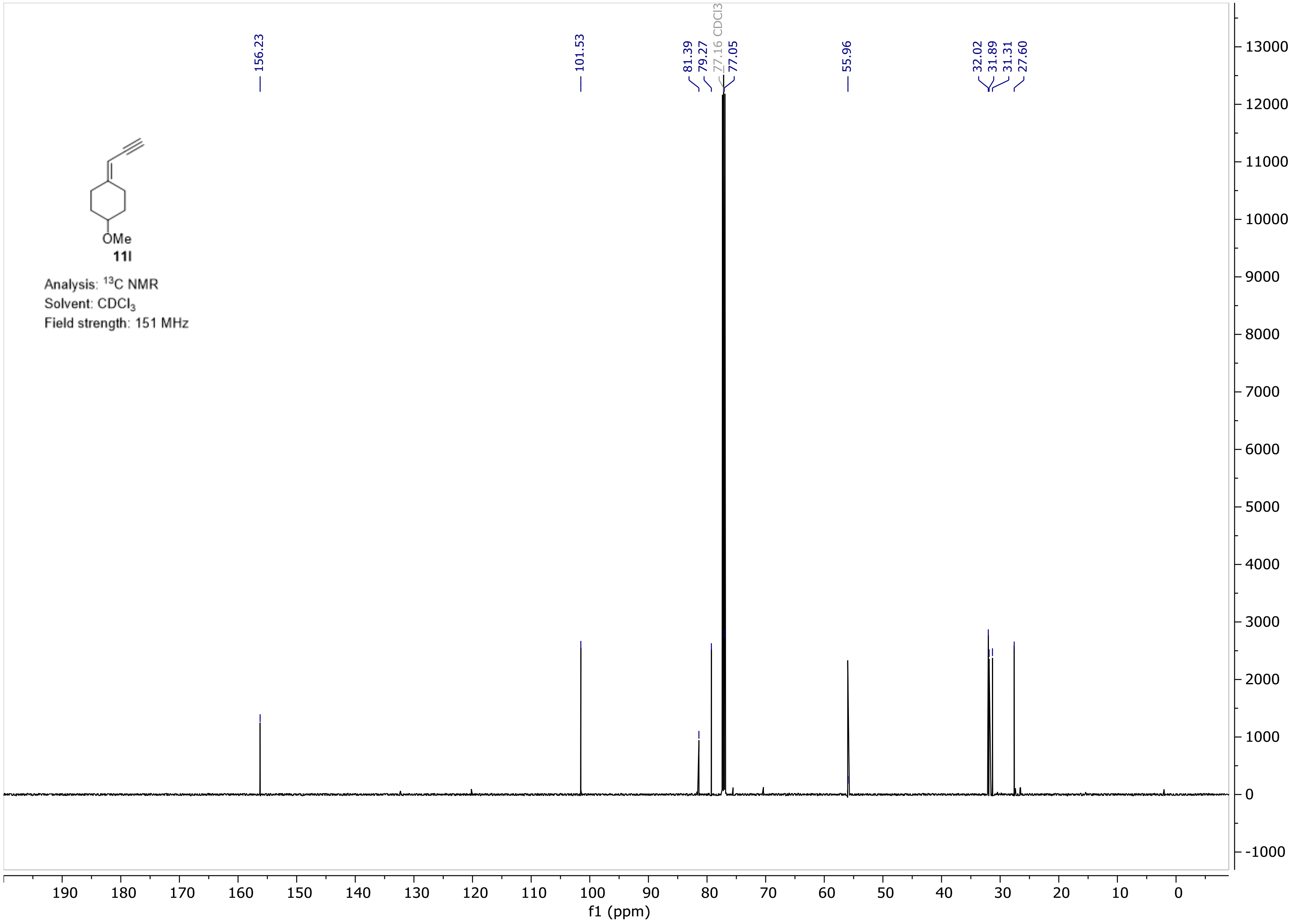

156.23
101.53
81.39
79.27
77.16 CDCl3
77.05
55.96
32.02
31.89
31.31
27.60
OMe
11l
Analysis: 13C NMR
Solvent: CDCl3
Field strength: 151 MHz
13000
12000
11000
10000
9000
8000
7000
6000
5000
4000
3000
2000
1000
0
-1000
190
180
170
160
150
140
130
120
110
100
90
80
70
60
50
40
30
20
10
0
f1 (ppm)

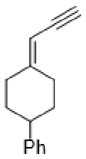

11m

Analysis: ^{1}H NMR
Solvent: $CDCl_3$
Field strength: 400 MHz

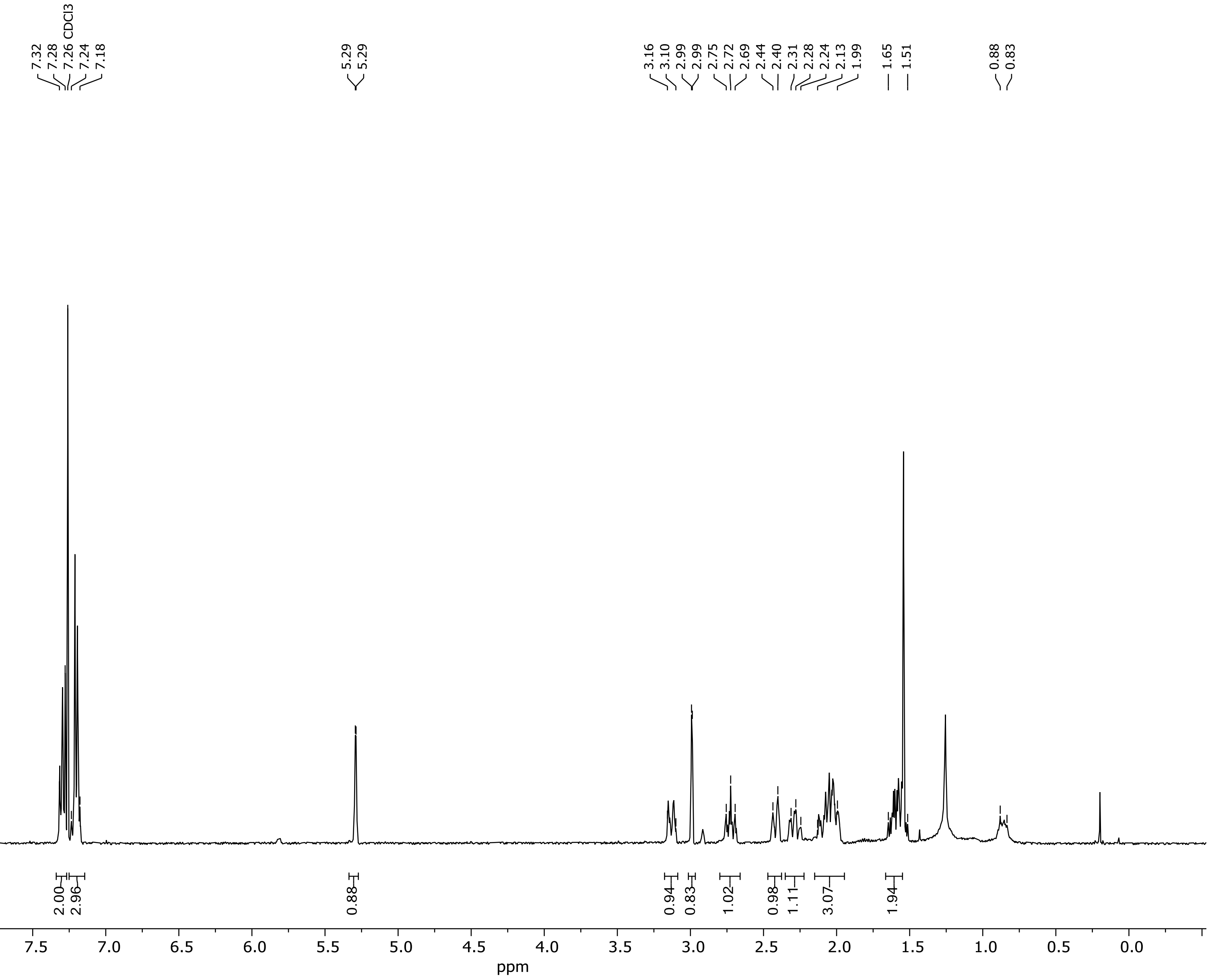

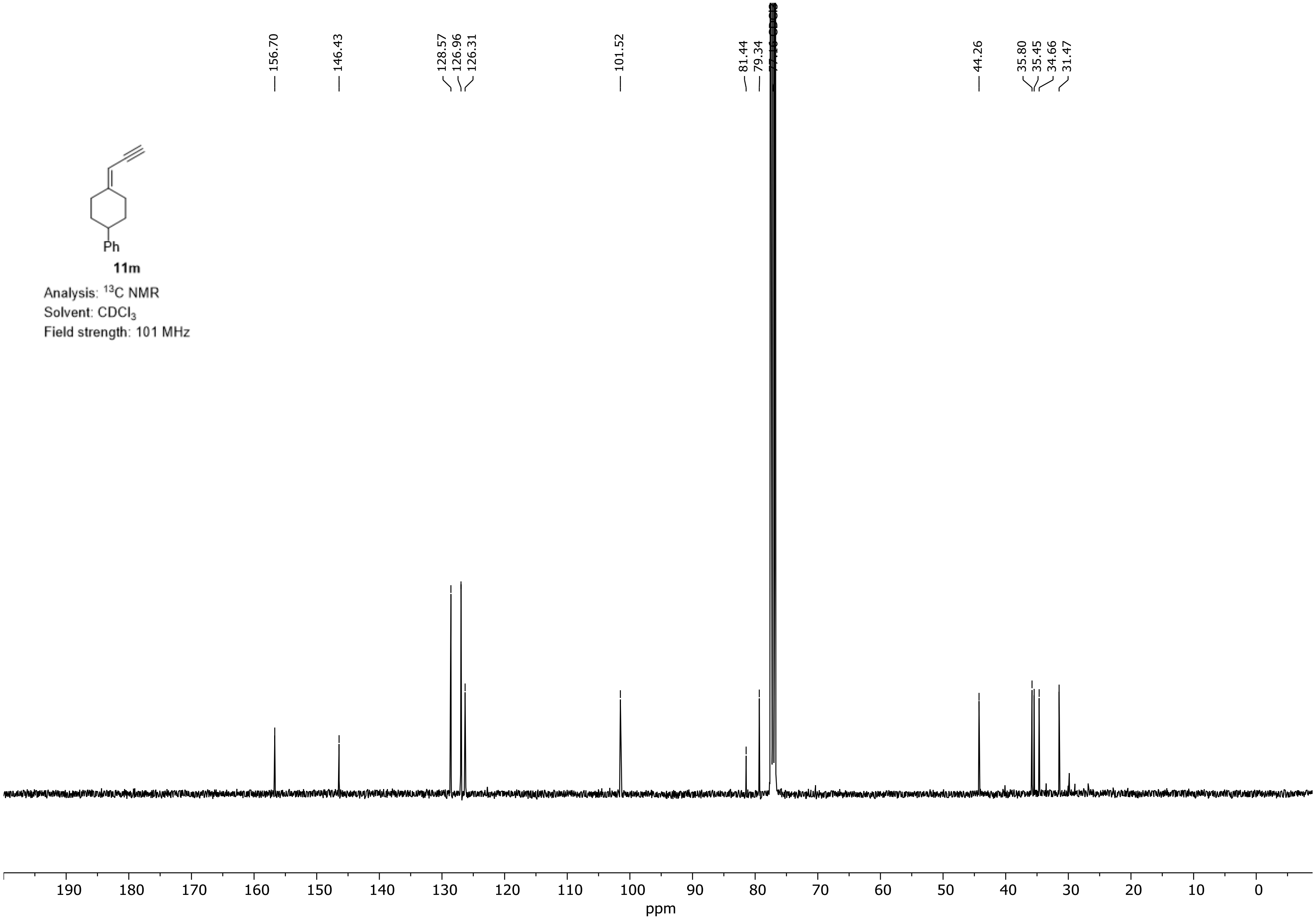

156.70
146.43
128.57
126.96
126.31
101.52
81.44
79.34
44.26
35.80
35.45
34.66
31.47
Ph
11m
Analysis: 13C NMR
Solvent: CDCl3
Field strength: 101 MHz
190 180 170 160 150 140 130 120 110 100 90 80 70 60 50 40 30 20 10 0
ppm

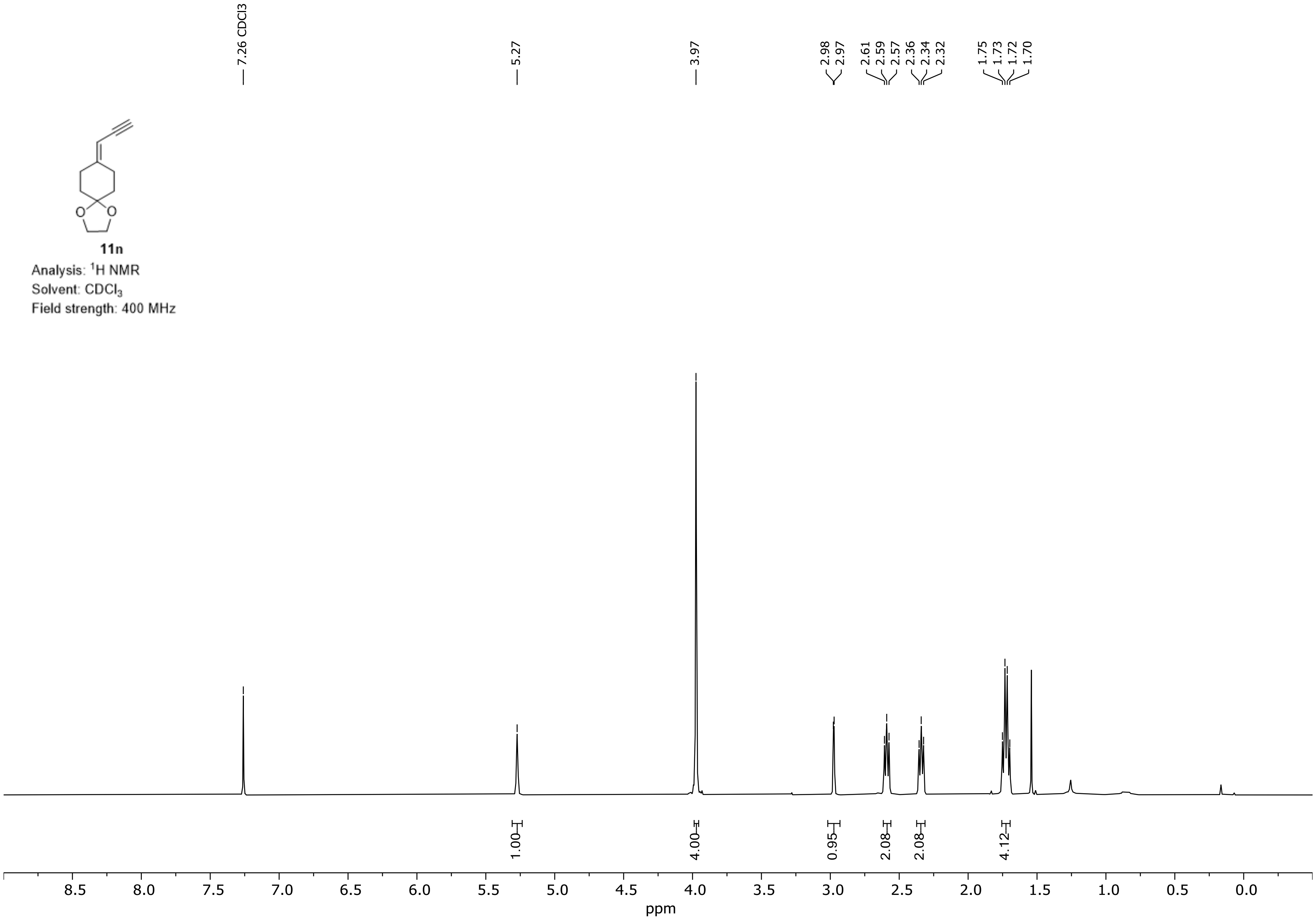

7.26 CDCl3
5.27
3.97
2.98
2.97
2.61
2.59
2.57
2.36
2.34
2.32
1.75
1.73
1.72
1.70
11n
Analysis: ^1H NMR
Solvent: $CDCl_3$
Field strength: 400 MHz
1.00
4.00
0.95
2.08
2.08
4.12
8.5
8.0
7.5
7.0
6.5
6.0
5.5
5.0
4.5
4.0
3.5
3.0
2.5
2.0
1.5
1.0
0.5
0.0
ppm

11n

Analysis: ^{13}C NMR

Solvent: $CDCl_3$

Field strength: 101 MHz

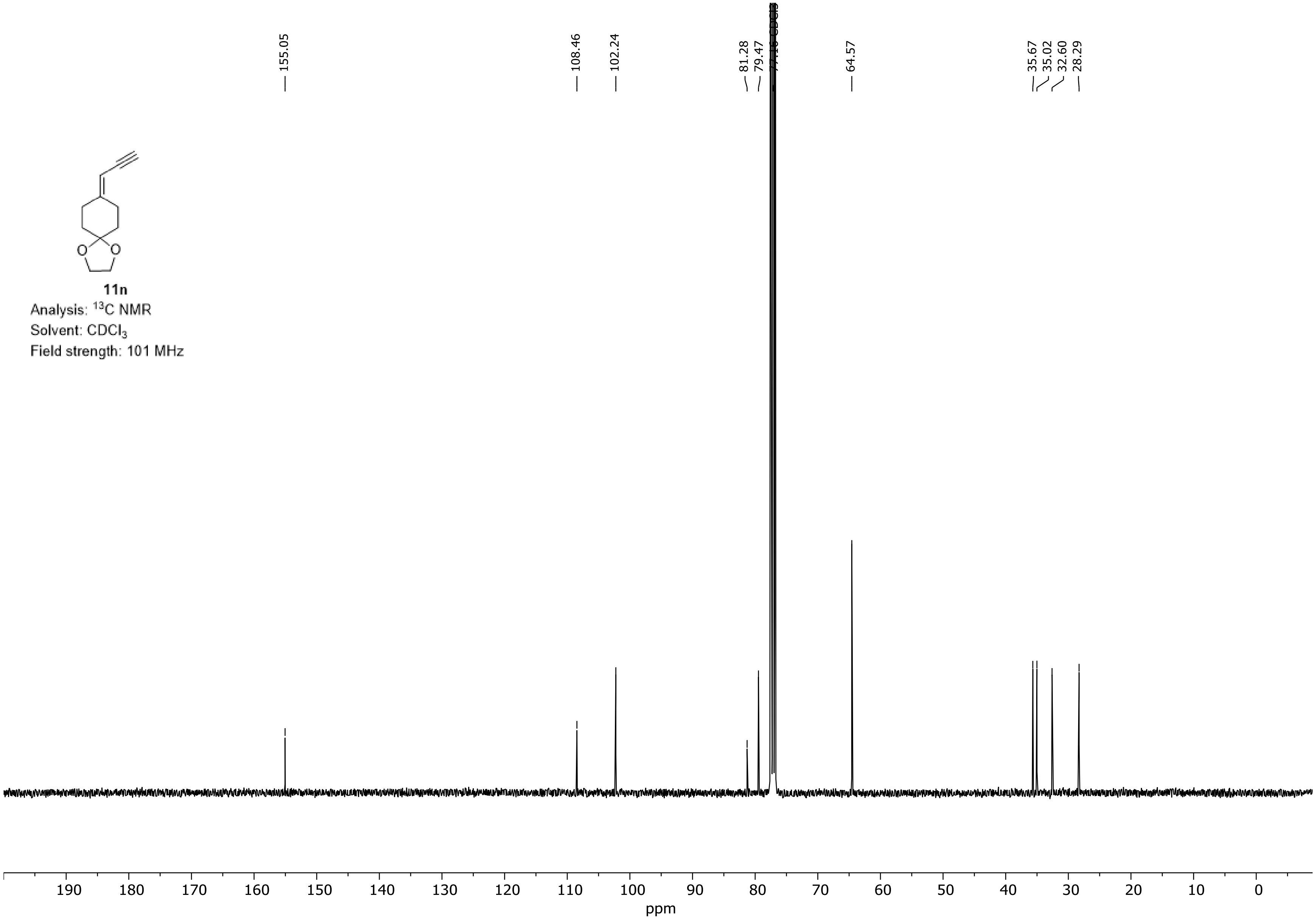

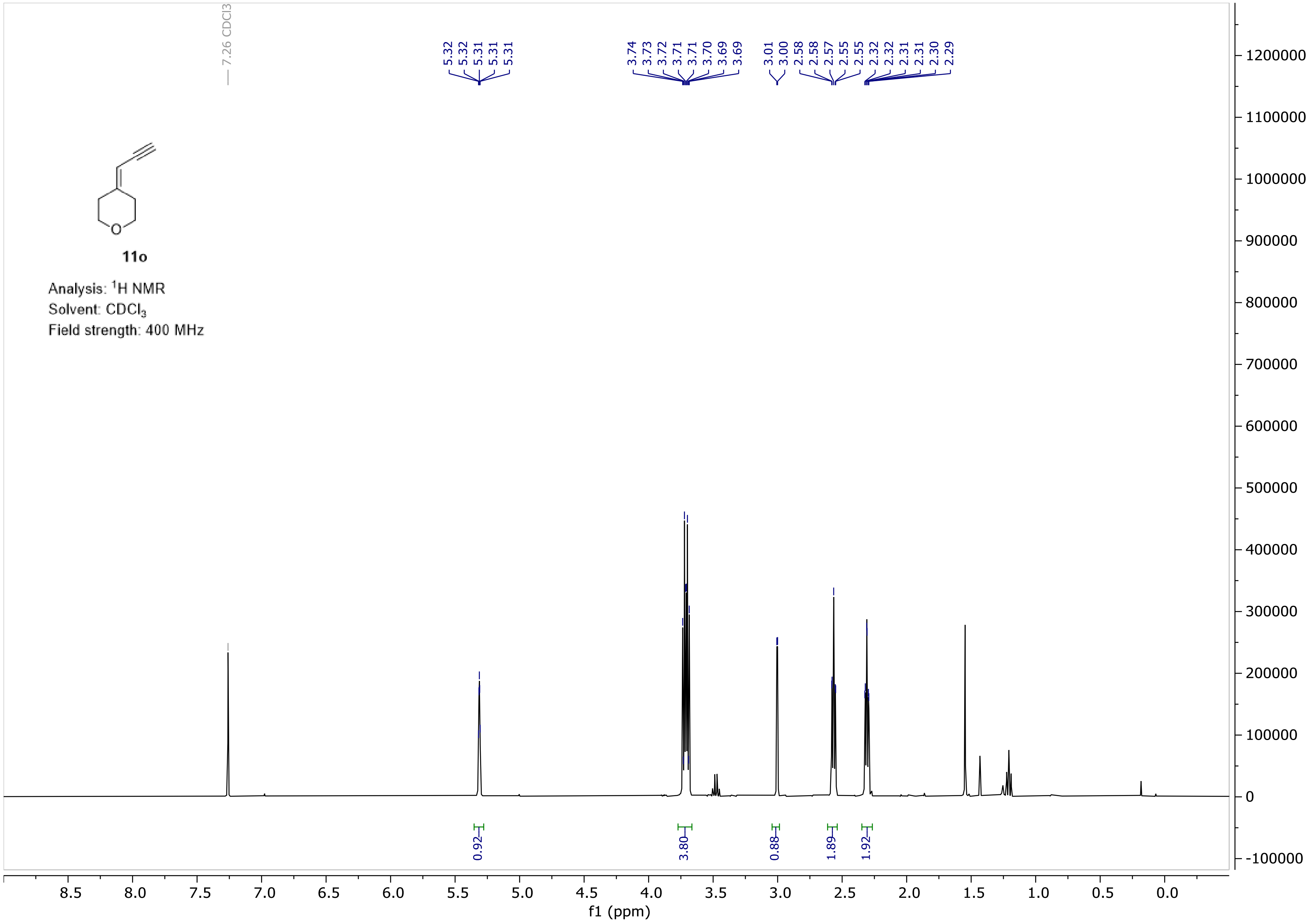
11o
Analysis: ¹H NMR
Solvent: CDCl3
Field strength: 400 MHz
7.26 CDCl3
5.32
5.32
5.31
5.31
5.31
3.74
3.73
3.72
3.71
3.71
3.70
3.69
3.69
3.01
3.00
2.58
2.58
2.57
2.55
2.55
2.32
2.32
2.32
2.31
2.31
2.30
2.29
0.92
3.80
0.88
1.89
1.92
f1 (ppm)

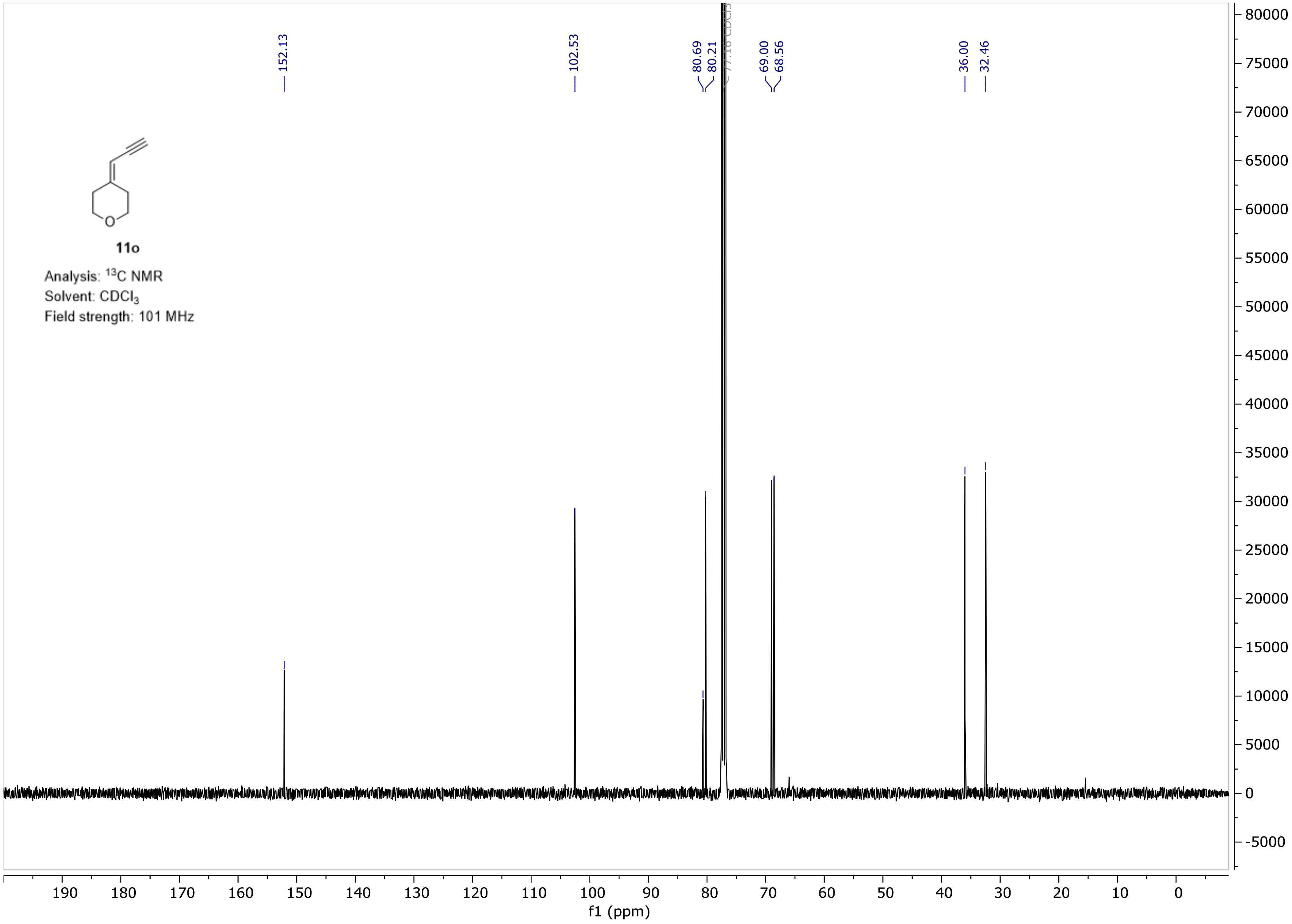

152.13
102.53
80.69
80.21
77.16 CDCl3
69.00
68.56
36.00
32.46
11o
Analysis: 13C NMR
Solvent: CDCl3
Field strength: 101 MHz
O
f1 (ppm)

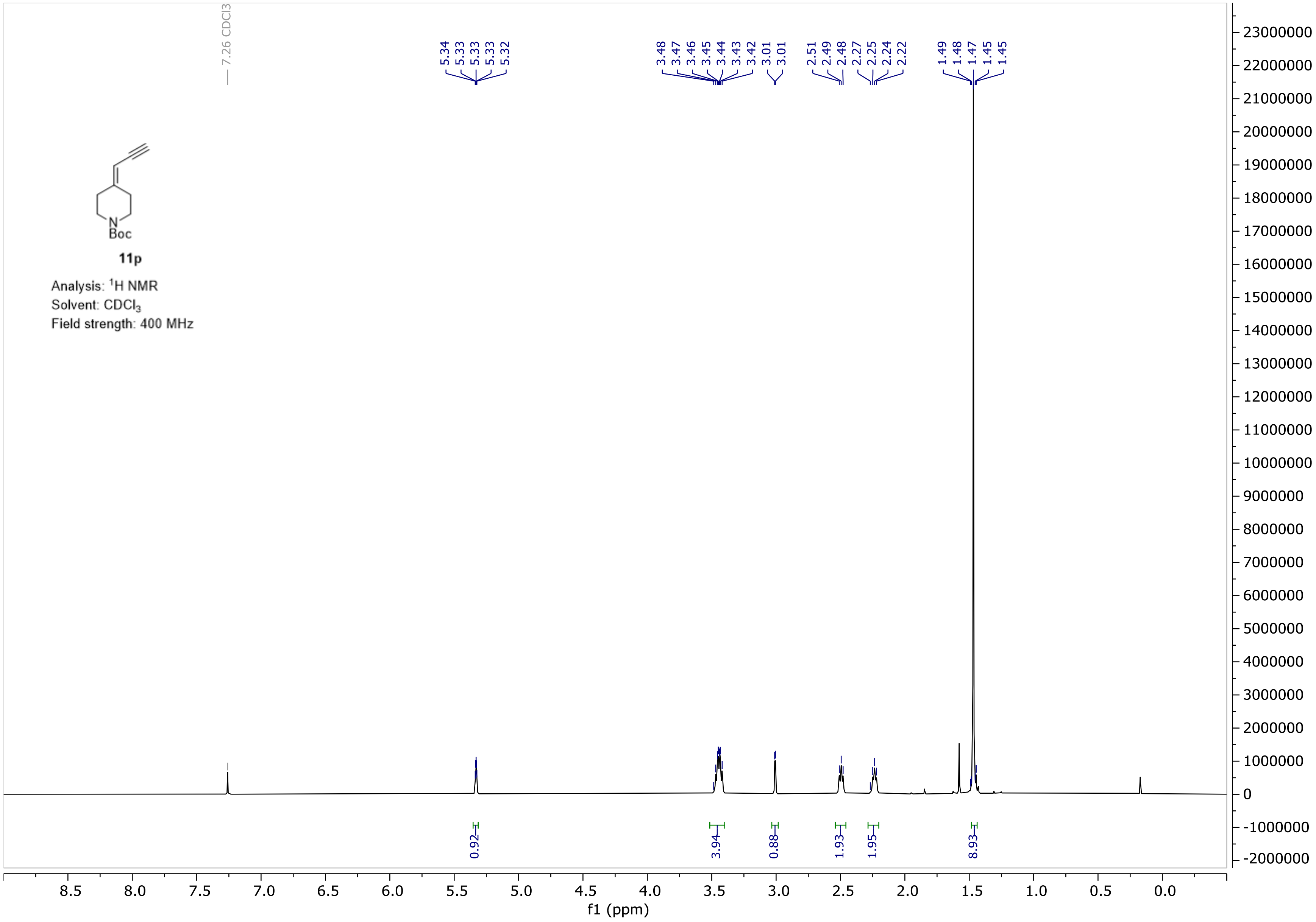
7.26 CDCl3
5.34 5.33 5.33 5.33 5.32
3.48 3.47 3.46 3.45 3.44 3.43 3.42 3.01 3.01
2.51 2.49 2.48 2.27 2.25 2.24 2.22
1.49 1.48 1.47 1.45 1.45
N
Boc
11p
Analysis: ¹H NMR
Solvent: CDCl₃
Field strength: 400 MHz
0.92 3.94 0.88 1.93 1.95 8.93
f1 (ppm)

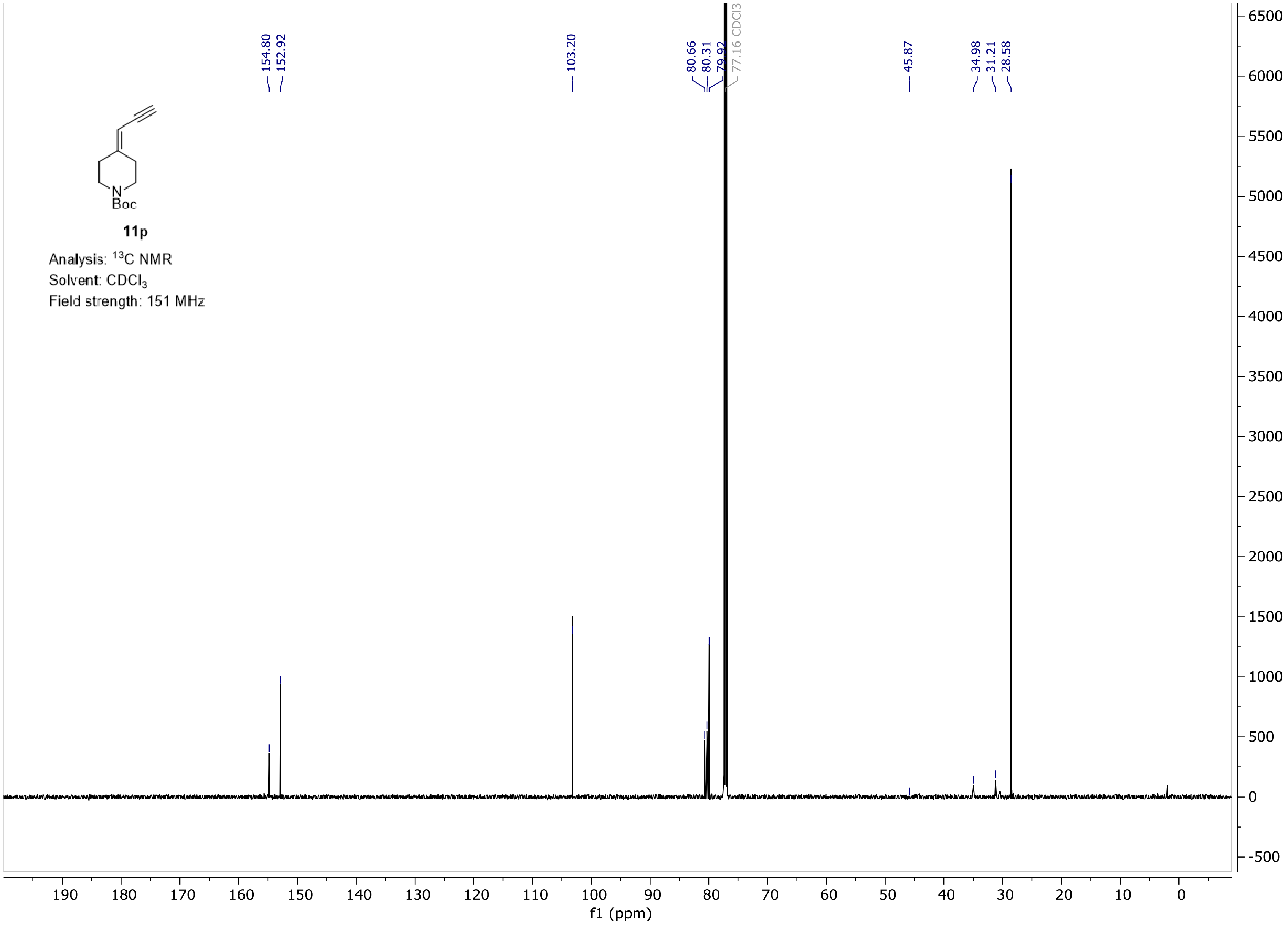

154.80
152.92
103.20
80.66
80.31
79.92
77.16 CDCl3
45.87
34.98
31.21
28.58
Boc
11p
Analysis: 13C NMR
Solvent: CDCl3
Field strength: 151 MHz
f1 (ppm)

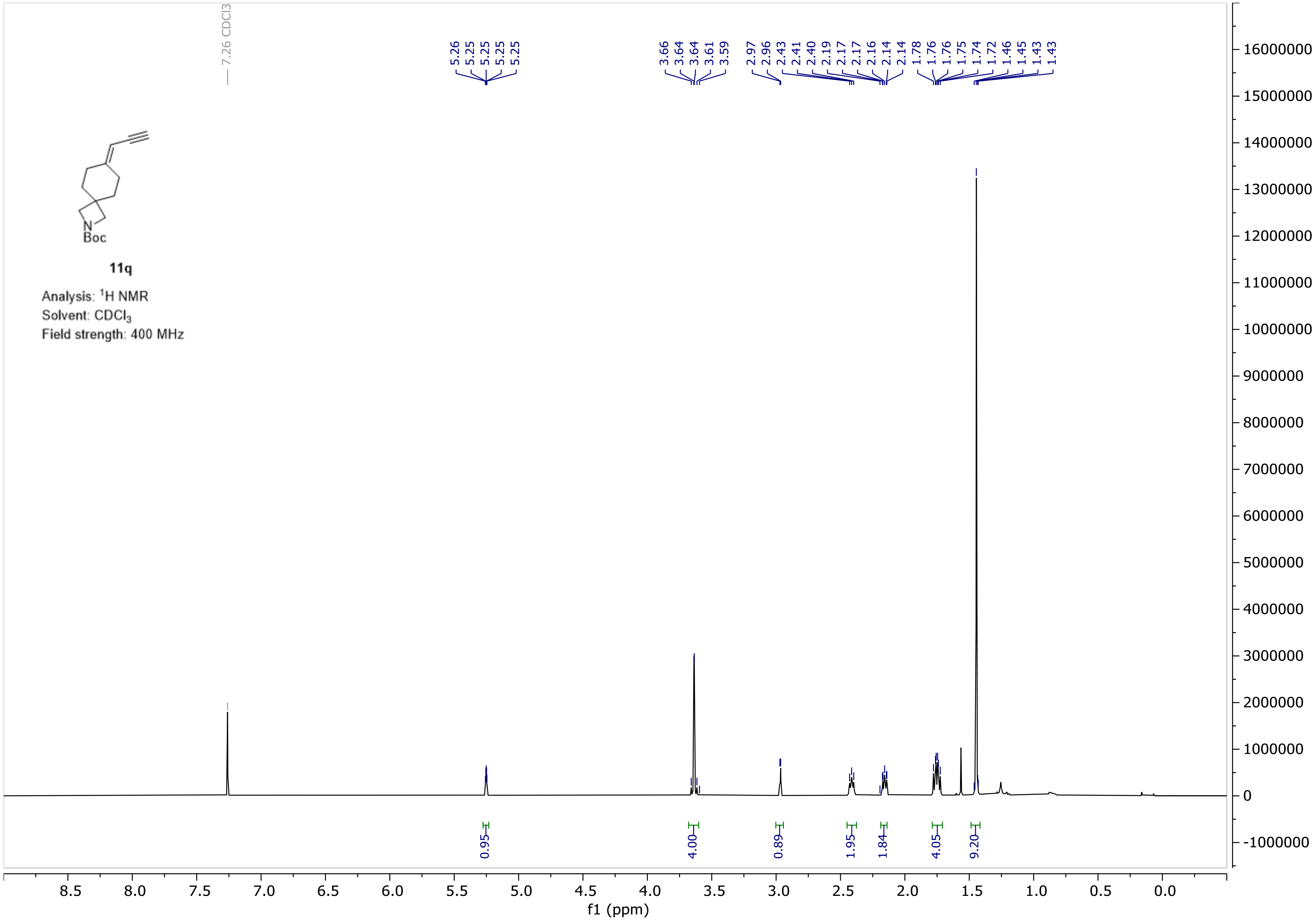

7.26 CDCl3
5.26 5.25 5.25 5.25 5.25 5.25
3.66 3.64 3.64 3.61 3.59
2.97 2.96 2.43 2.41 2.40 2.19 2.17 2.17 2.16 2.14 2.14 1.78 1.76 1.76 1.75 1.74 1.72 1.46 1.45 1.43 1.43
Boc
11q
Analysis: ¹H NMR
Solvent: CDCl3
Field strength: 400 MHz
0.95
4.00
0.89
1.95
1.84
4.05
9.20
f1 (ppm)

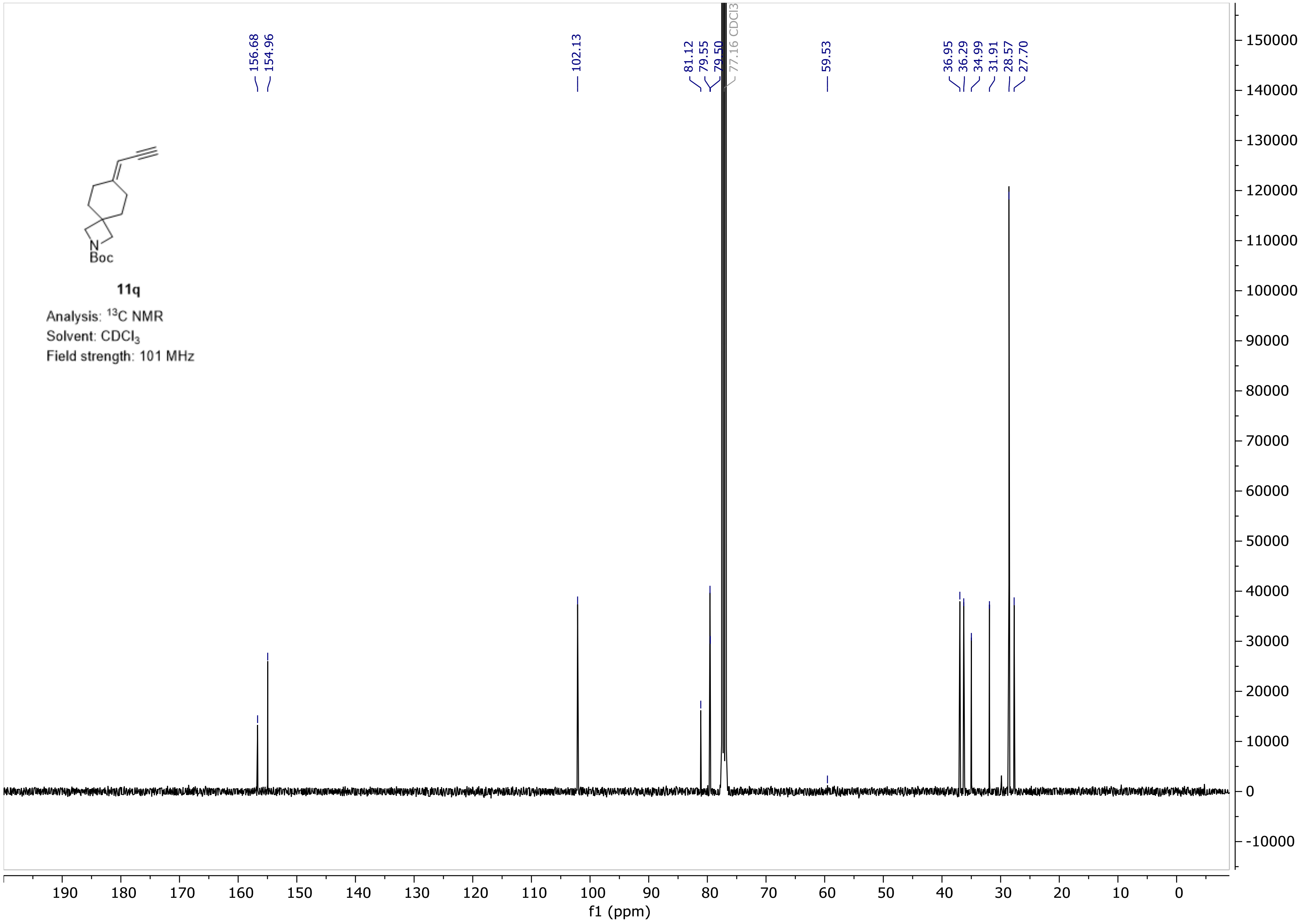

156.68
154.96
102.13
81.12
79.55
79.50
77.16 CDCl3
59.53
36.95
36.29
34.99
31.91
28.57
27.70
N
Boc
11q
Analysis: 13C NMR
Solvent: CDCl3
Field strength: 101 MHz
f1 (ppm)

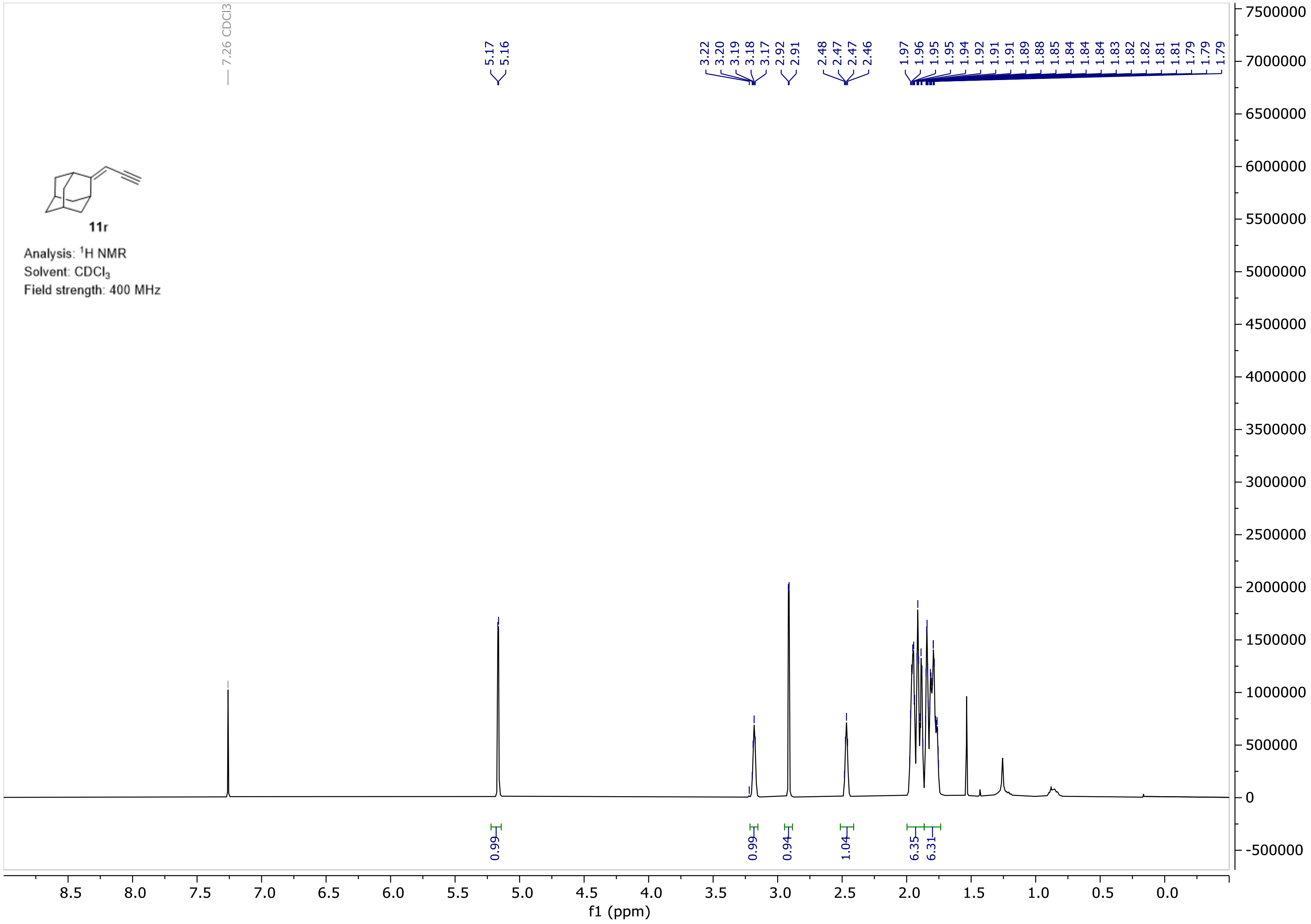
7.26 CDCl3
5.17
5.16
3.22
3.20
3.19
3.18
3.17
2.92
2.91
2.48
2.47
2.47
2.46
1.97
1.96
1.95
1.95
1.94
1.92
1.91
1.91
1.89
1.88
1.85
1.84
1.84
1.84
1.83
1.82
1.82
1.81
1.81
1.79
1.79
1.79
11r
Analysis: 1H NMR
Solvent: CDCl3
Field strength: 400 MHz
0.99
0.99
0.94
1.04
6.35
6.31
f1 (ppm)

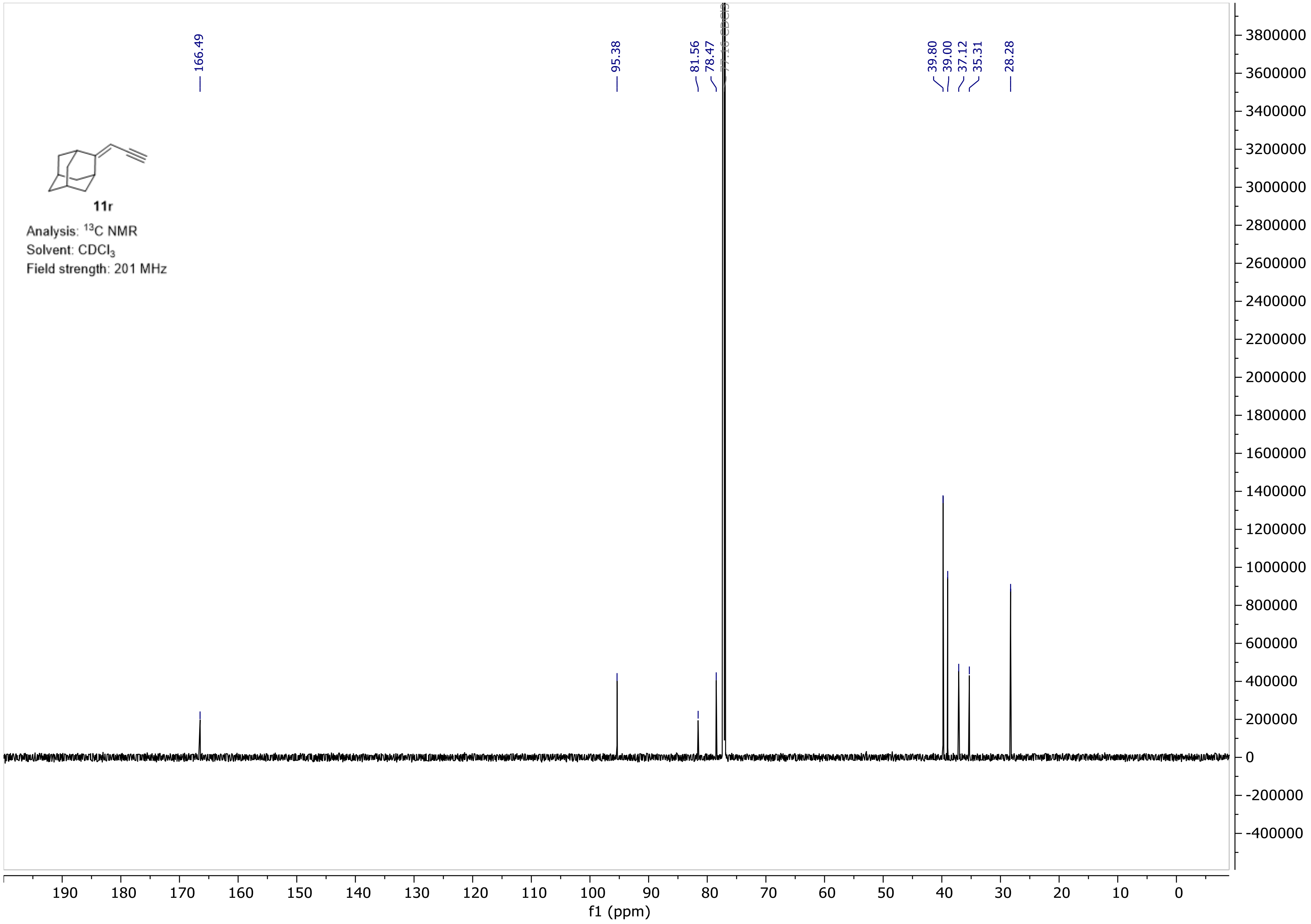
166.49
95.38
81.56
78.47
39.80
39.00
37.12
35.31
28.28
11r
Analysis: ^{13}C NMR
Solvent: $CDCl_3$
Field strength: 201 MHz
f1 (ppm)

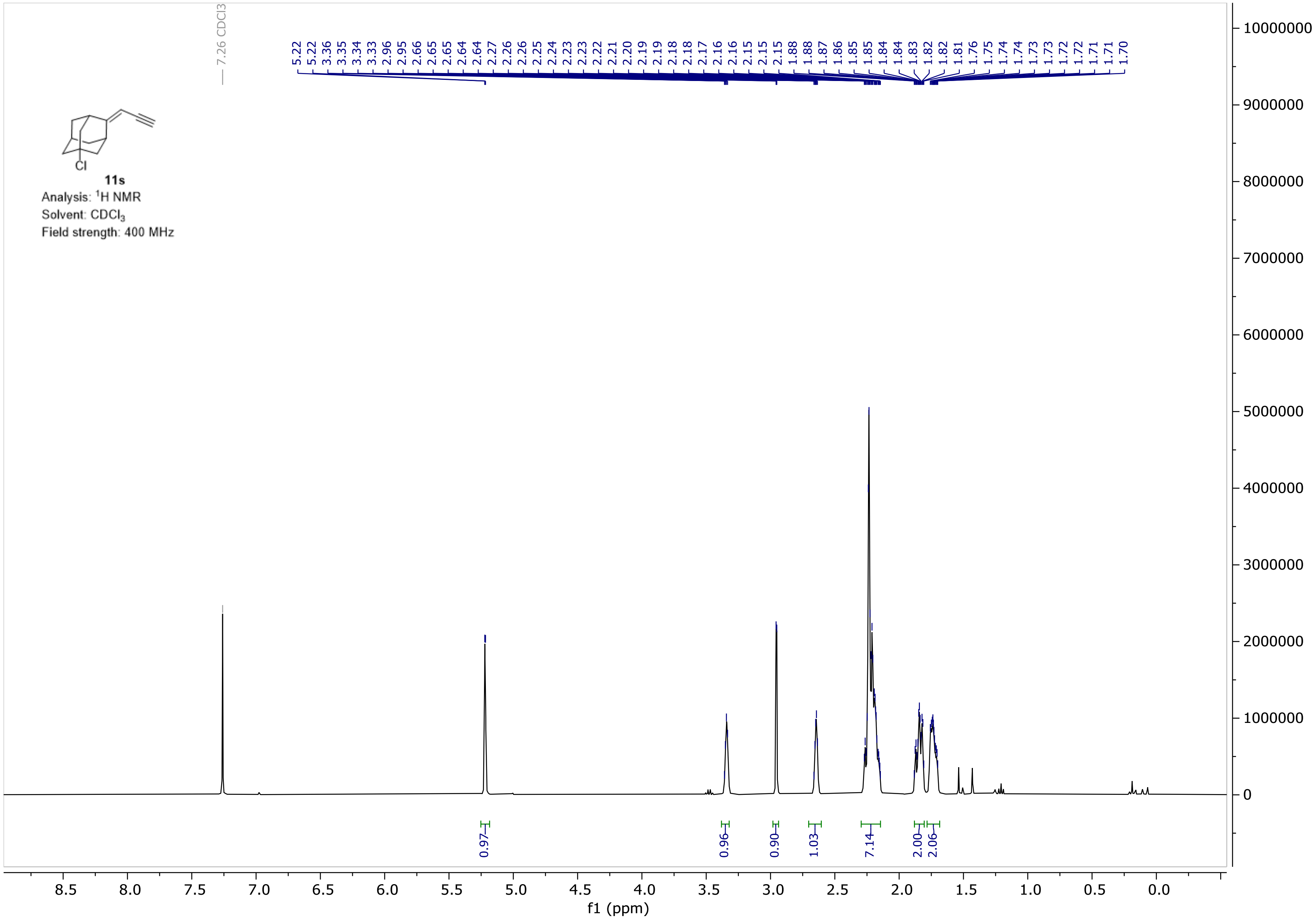
11s
Analysis: ^{1}H NMR
Solvent: $CDCl_3$
Field strength: 400 MHz
7.26 CDCl3
5.22 5.22 3.36 3.35 3.34 3.33 2.96 2.95 2.66 2.65 2.65 2.64 2.64 2.27 2.26 2.26 2.25 2.24 2.23 2.23 2.22 2.21 2.20 2.19 2.19 2.18 2.18 2.17 2.16 2.16 2.15 2.15 2.15 1.88 1.88 1.87 1.86 1.85 1.85 1.84 1.84 1.83 1.82 1.82 1.81 1.76 1.75 1.74 1.74 1.73 1.73 1.72 1.72 1.71 1.71 1.70
0.97 0.96 0.90 1.03 7.14 2.00 2.06
f1 (ppm)

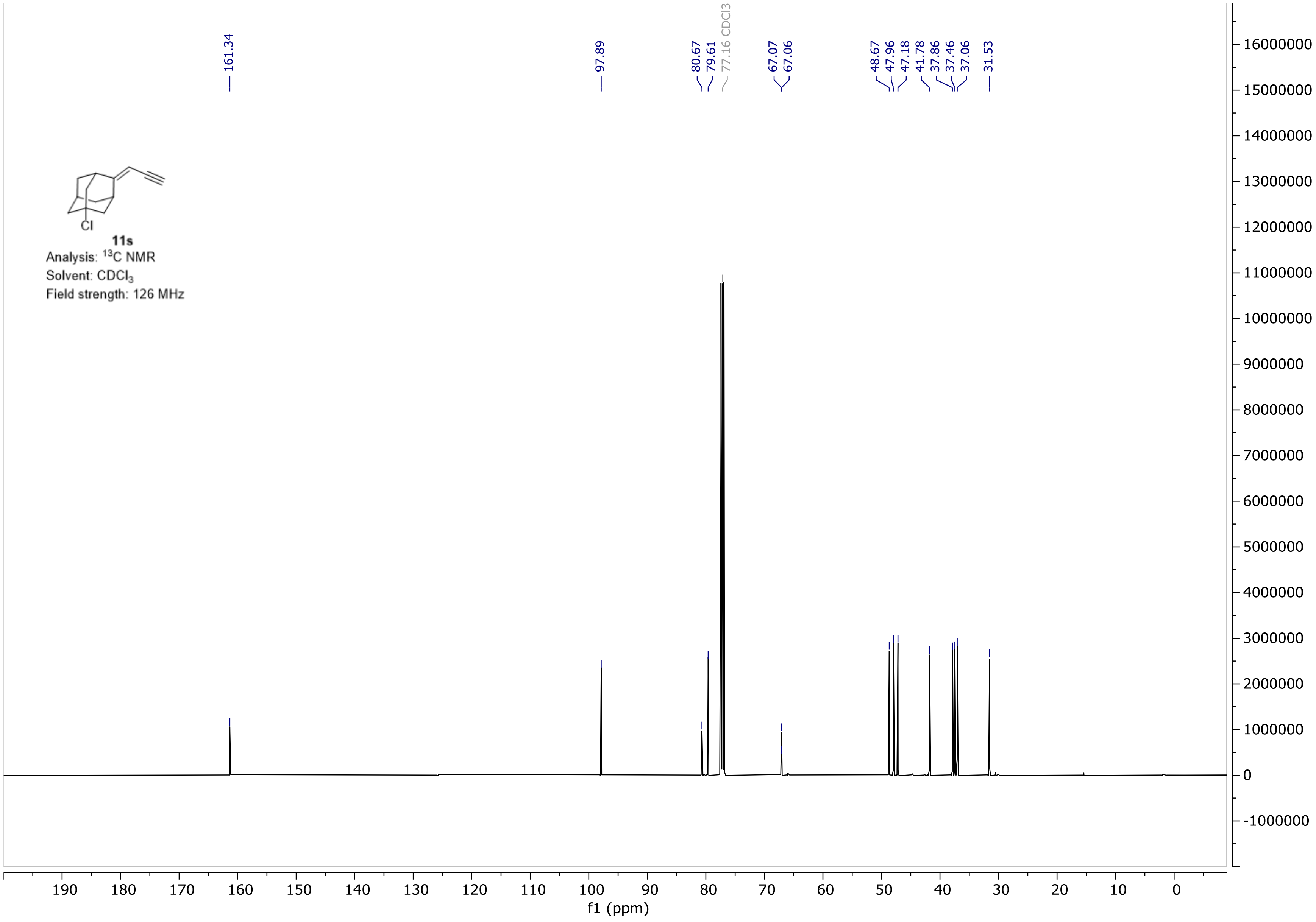

161.34
97.89
80.67
79.61
77.16 CDCl3
67.07
67.06
48.67
47.96
47.18
41.78
37.86
37.46
37.06
31.53
Cl
11s
Analysis: ^{13}C NMR
Solvent: $CDCl_3$
Field strength: 126 MHz
f1 (ppm)

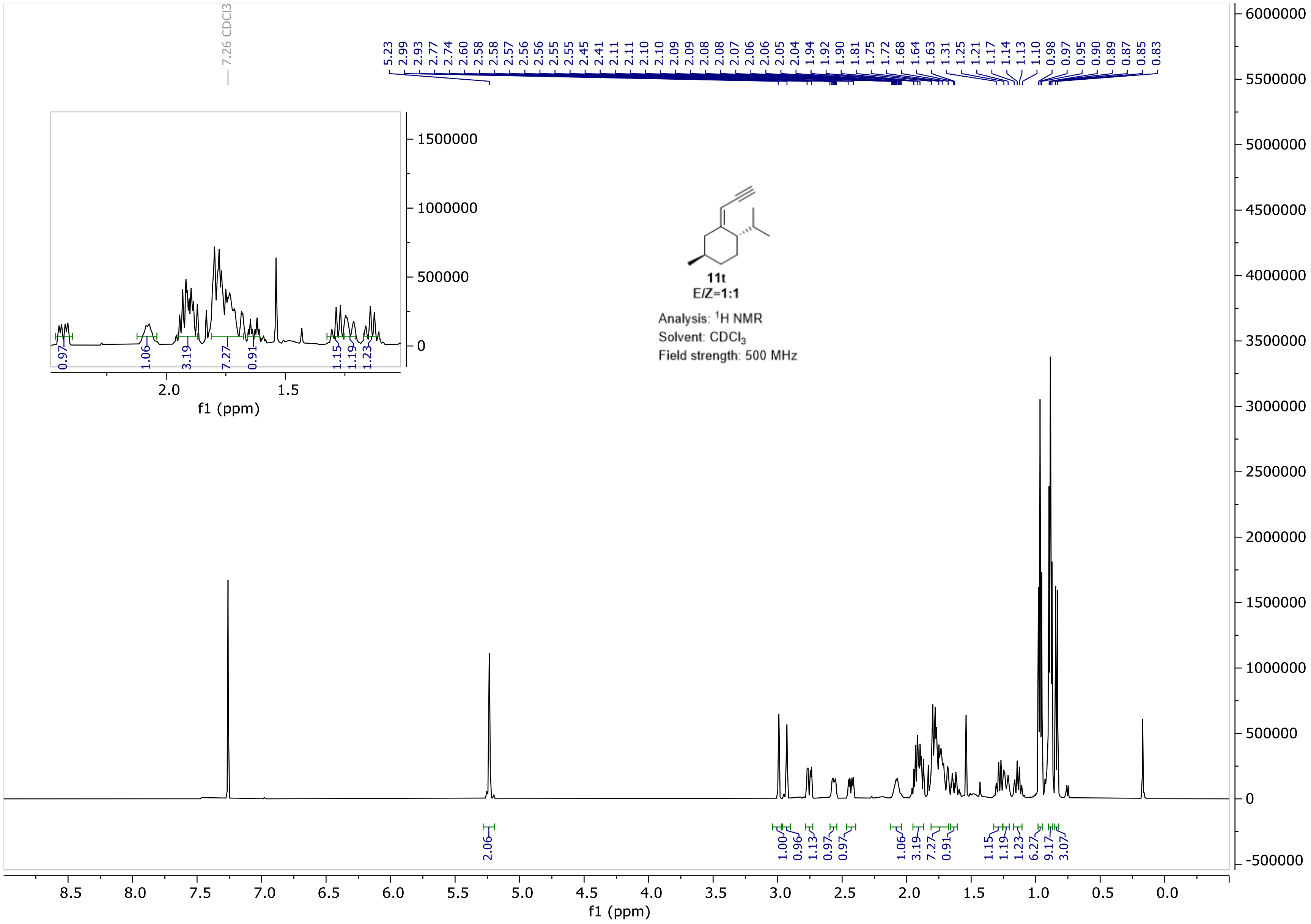

11t
E/Z=1:1
Analysis: ¹H NMR
Solvent: CDCl3
Field strength: 500 MHz
7.26 CDCl3
5.23
2.99
2.93
2.77
2.74
2.60
2.58
2.58
2.57
2.56
2.56
2.55
2.55
2.45
2.41
2.11
2.11
2.10
2.10
2.09
2.09
2.08
2.08
2.07
2.06
2.06
2.05
2.04
1.94
1.92
1.90
1.81
1.75
1.72
1.68
1.64
1.63
1.31
1.25
1.21
1.17
1.14
1.13
1.10
0.98
0.97
0.95
0.90
0.89
0.87
0.85
0.83
2.06
1.00
0.96
1.13
0.97
0.97
1.06
3.19
7.27
0.91
1.15
1.19
1.23
6.27
9.17
3.07
f1 (ppm)

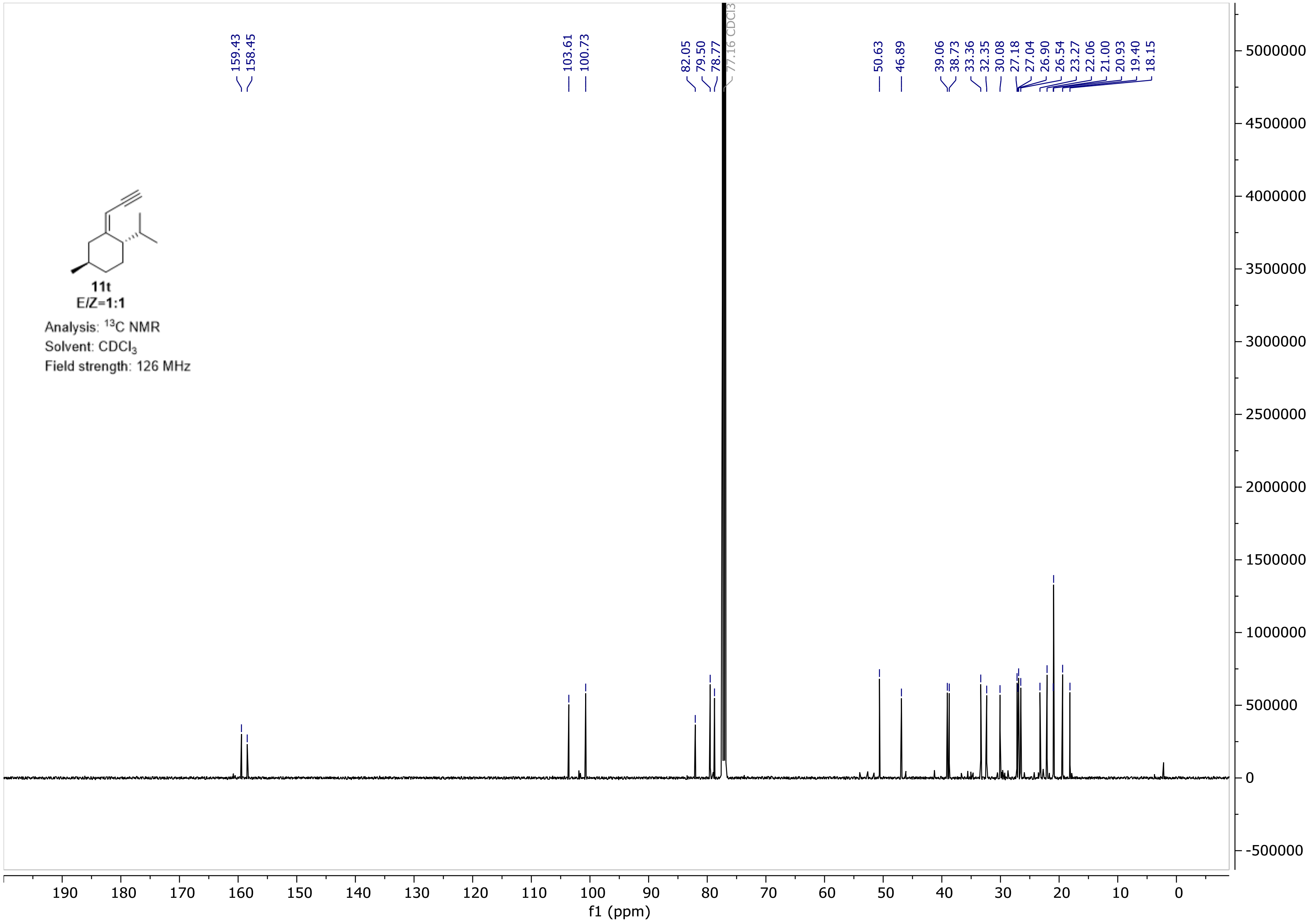

11t
E/Z=**1:1**

Analysis: ^{13}C NMR
Solvent: $CDCl_3$
Field strength: 126 MHz

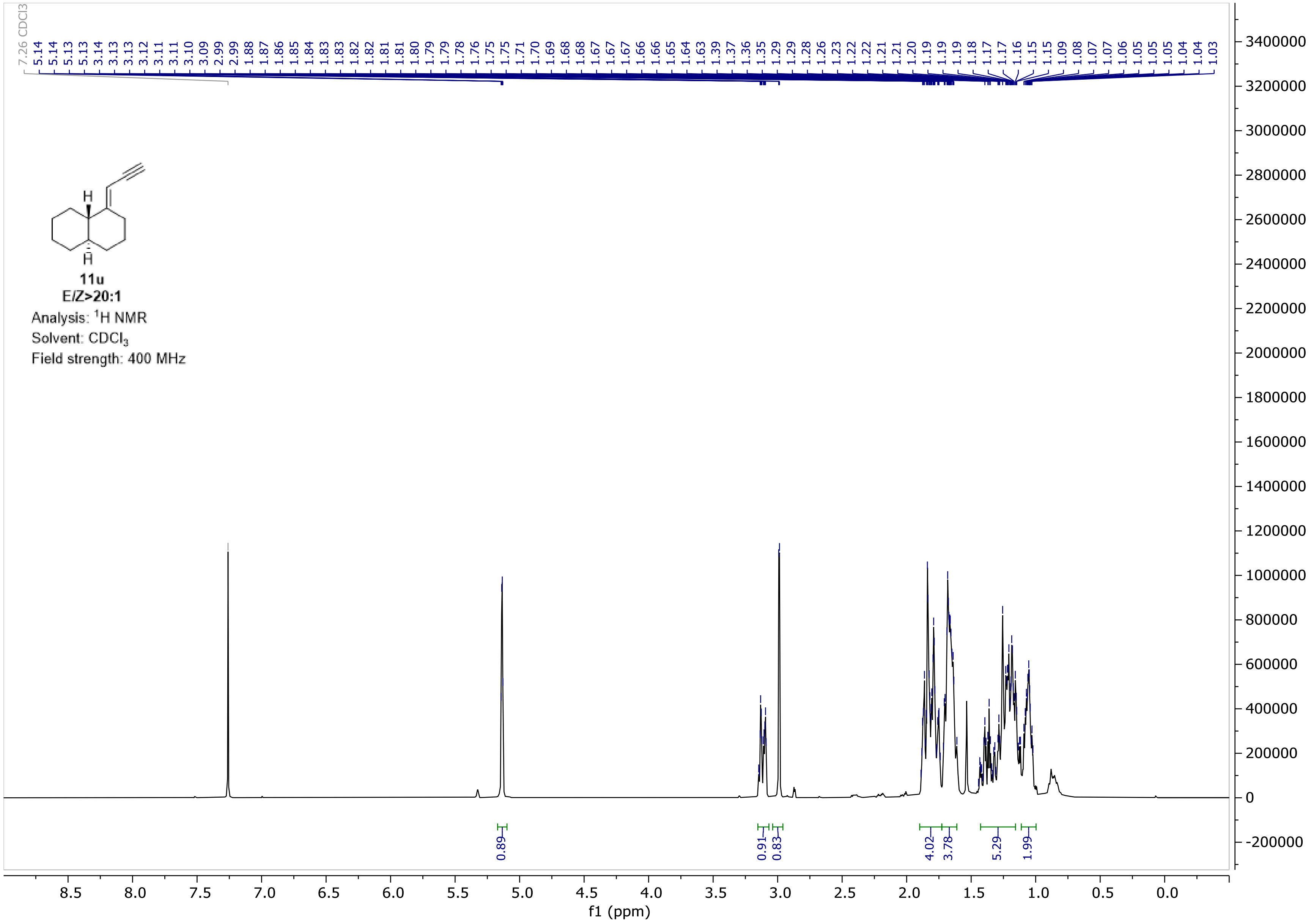
11u
E/Z>20:1
Analysis: ^{1}H NMR
Solvent: $CDCl_3$
Field strength: 400 MHz
f1 (ppm)

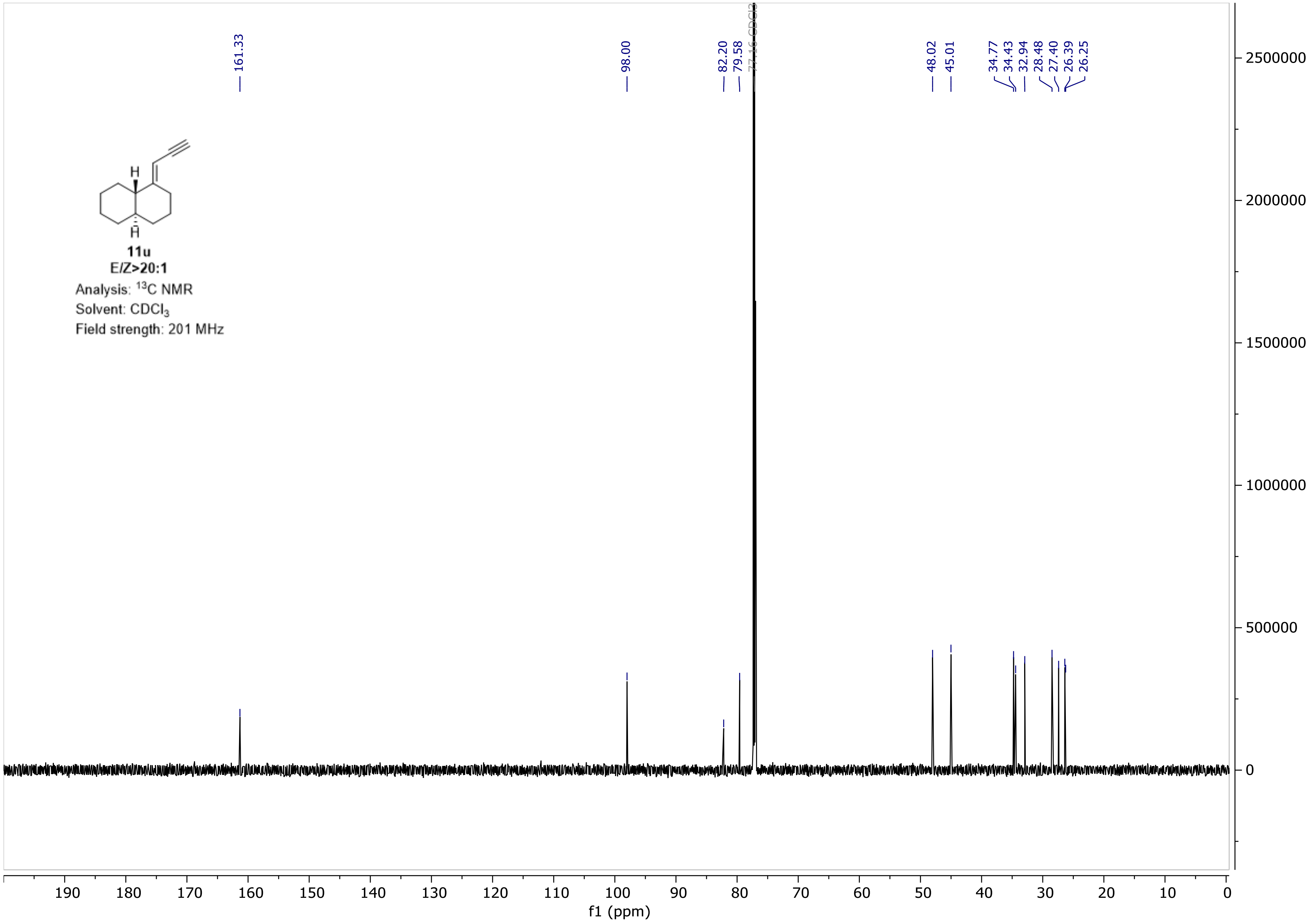

161.33
98.00
82.20
79.58
77.16 CDCl3
48.02
45.01
34.77
34.43
32.94
28.48
27.40
26.39
26.25
11u
E/Z>20:1
Analysis: 13C NMR
Solvent: CDCl3
Field strength: 201 MHz
f1 (ppm)

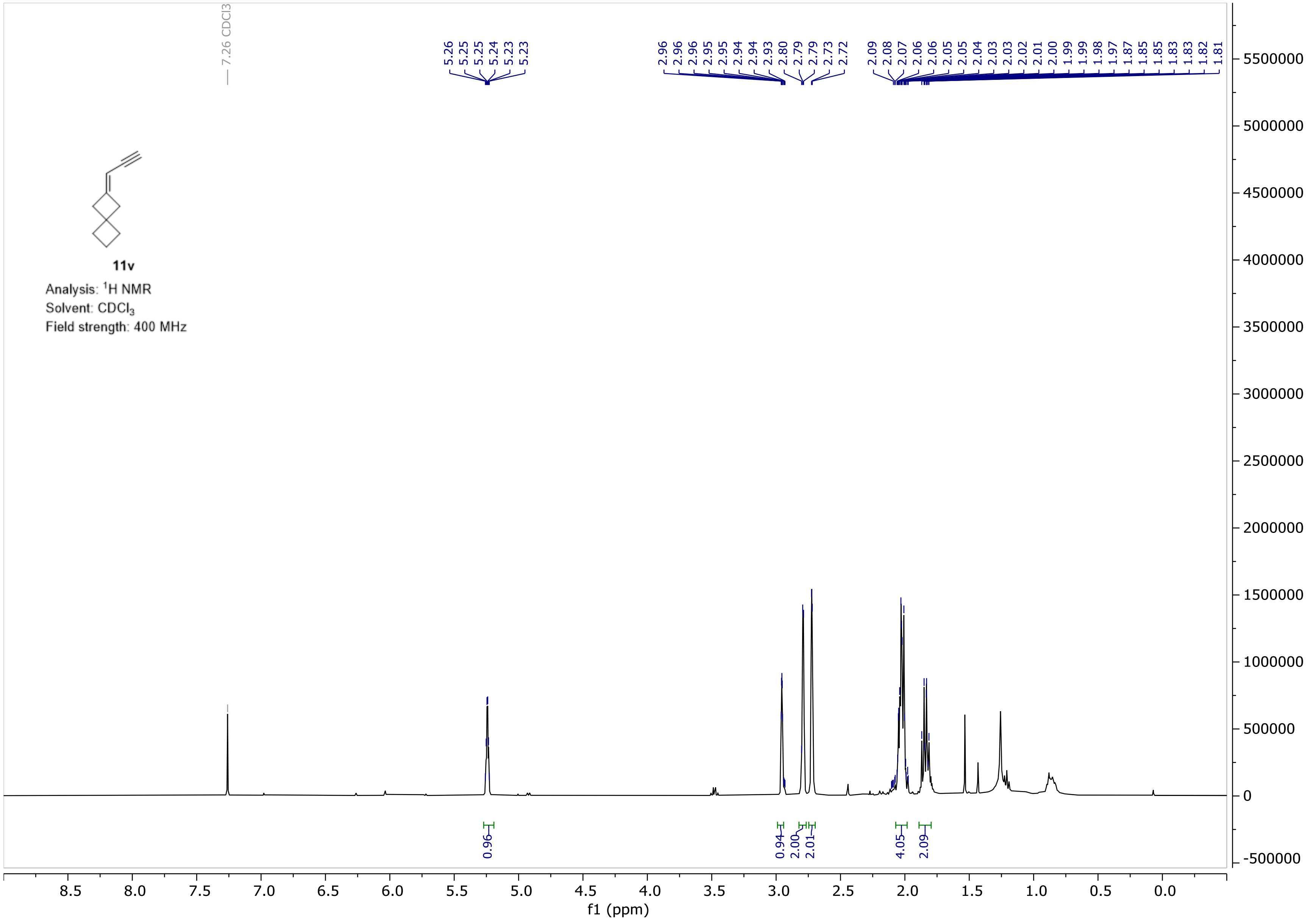
7.26 CDCl3
5.26
5.25
5.25
5.24
5.23
5.23
2.96
2.96
2.96
2.95
2.95
2.94
2.94
2.93
2.80
2.79
2.79
2.73
2.72
2.09
2.08
2.07
2.06
2.06
2.05
2.05
2.04
2.03
2.03
2.02
2.01
2.00
1.99
1.99
1.98
1.97
1.87
1.85
1.85
1.83
1.83
1.82
1.81
11v
Analysis: ^{1}H NMR
Solvent: $CDCl_3$
Field strength: 400 MHz
0.96
0.94
2.00
2.01
4.05
2.09
f1 (ppm)

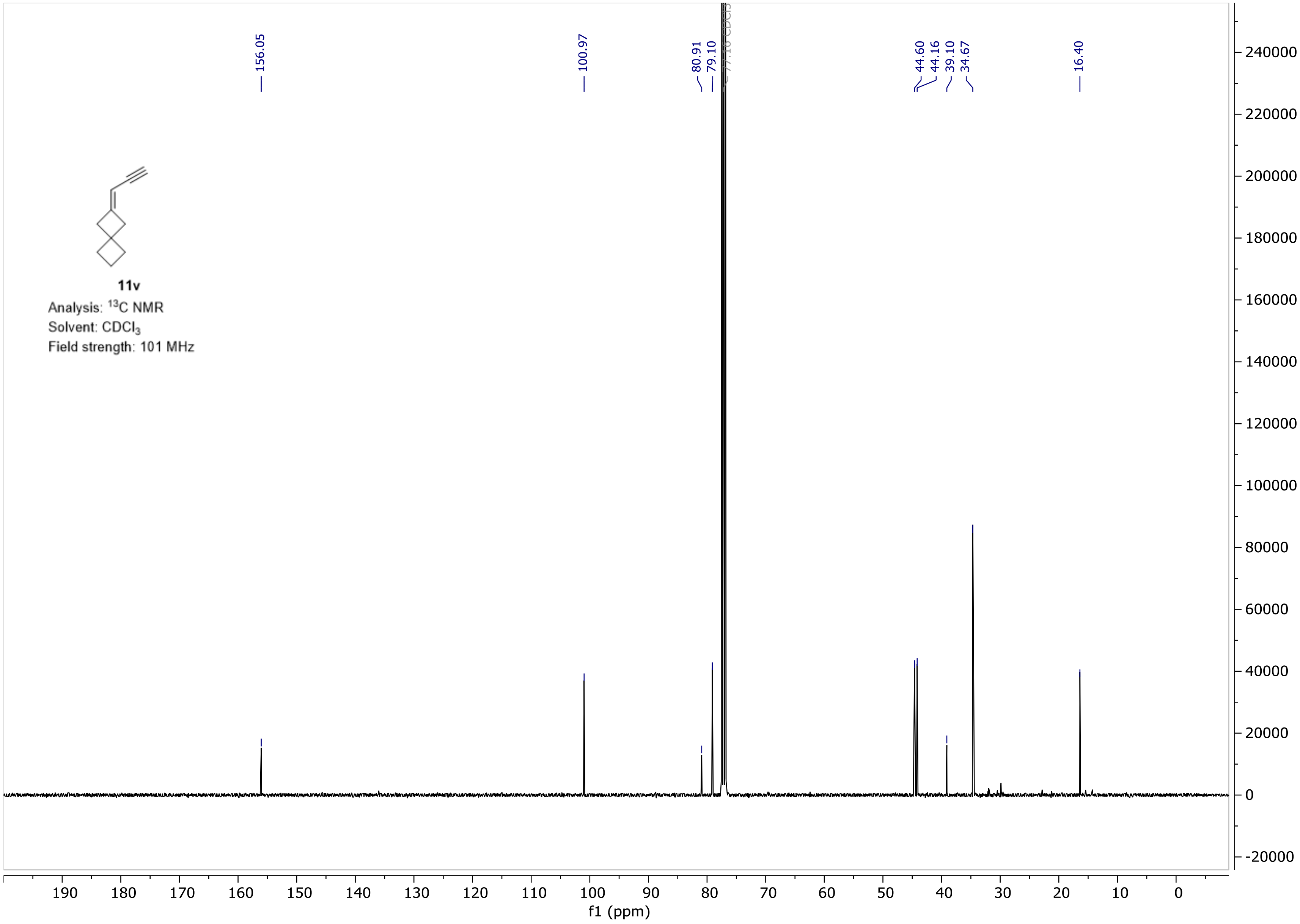

156.05
100.97
80.91
79.10
44.60
44.16
39.10
34.67
16.40
11v
Analysis: ^{13}C NMR
Solvent: $CDCl_3$
Field strength: 101 MHz
240000
220000
200000
180000
160000
140000
120000
100000
80000
60000
40000
20000
0
-20000
190 180 170 160 150 140 130 120 110 100 90 80 70 60 50 40 30 20 10 0
f1 (ppm)

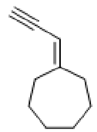

11w

Analysis: ^{1}H NMR

Solvent: $CDCl_3$

Field strength: 400 MHz

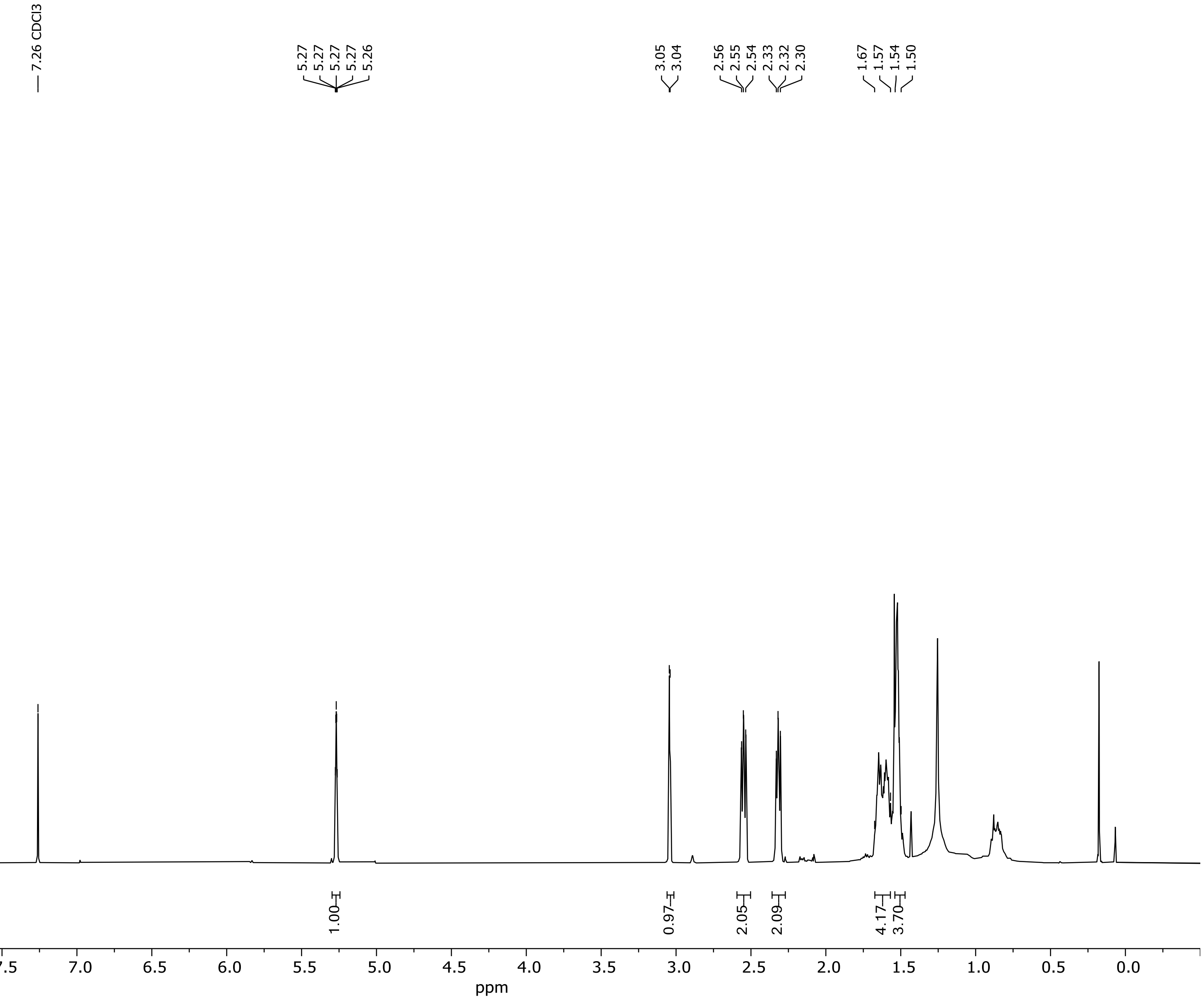

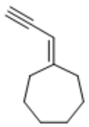

11w

Analysis: ^{13}C NMR

Solvent: $CDCl_3$

Field strength: 101 MHz

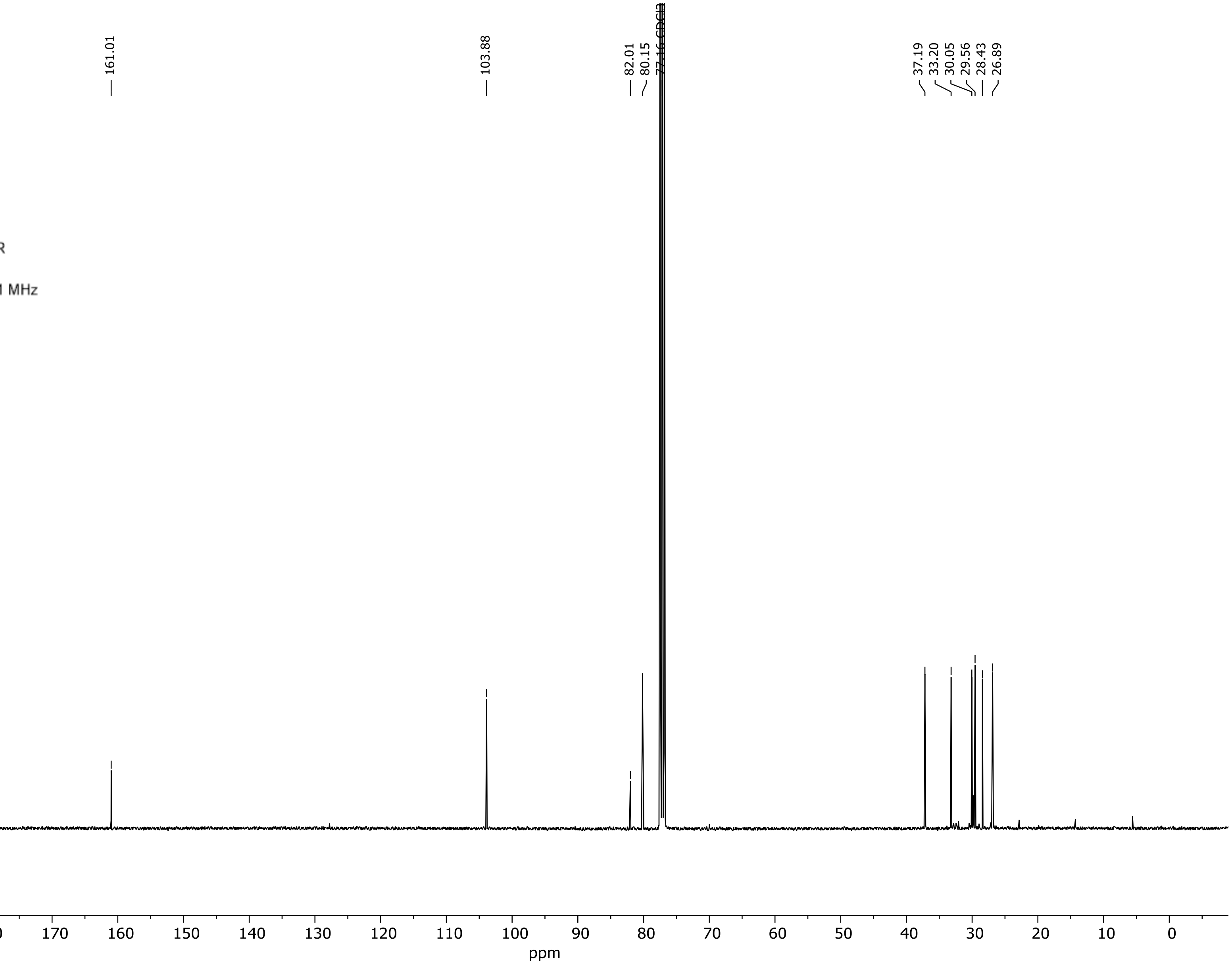

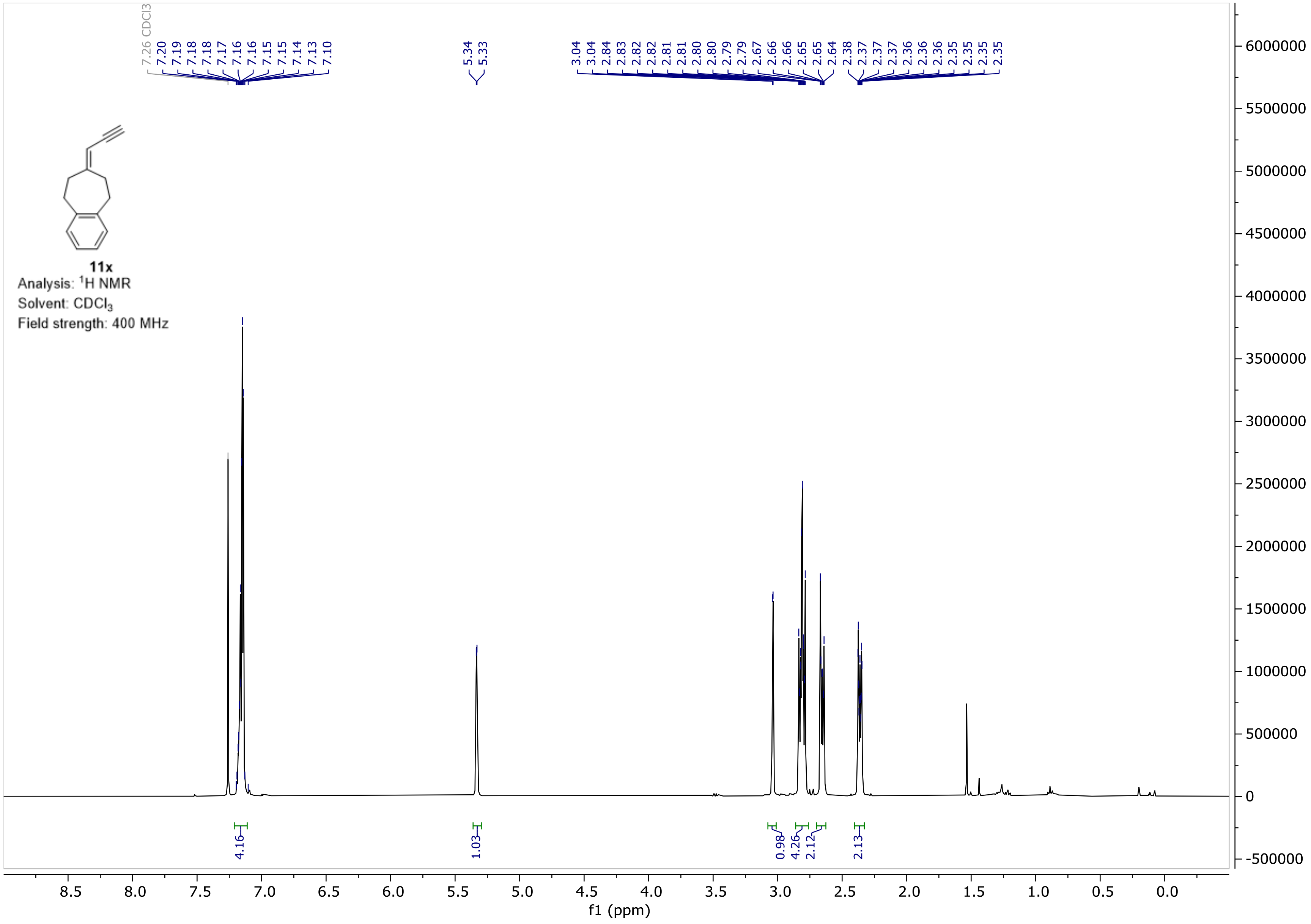

7.26 CDCl3
7.20 7.19 7.18 7.18 7.17 7.16 7.16 7.15 7.15 7.14 7.13 7.10
5.34 5.33
3.04 3.04 2.84 2.83 2.82 2.82 2.81 2.81 2.80 2.80 2.79 2.79 2.67 2.66 2.66 2.65 2.65 2.64 2.38 2.37 2.37 2.37 2.36 2.36 2.36 2.35 2.35 2.35 2.35
11x
Analysis: ¹H NMR
Solvent: CDCl3
Field strength: 400 MHz
4.16
1.03
0.98
4.26
2.12
2.13
f1 (ppm)

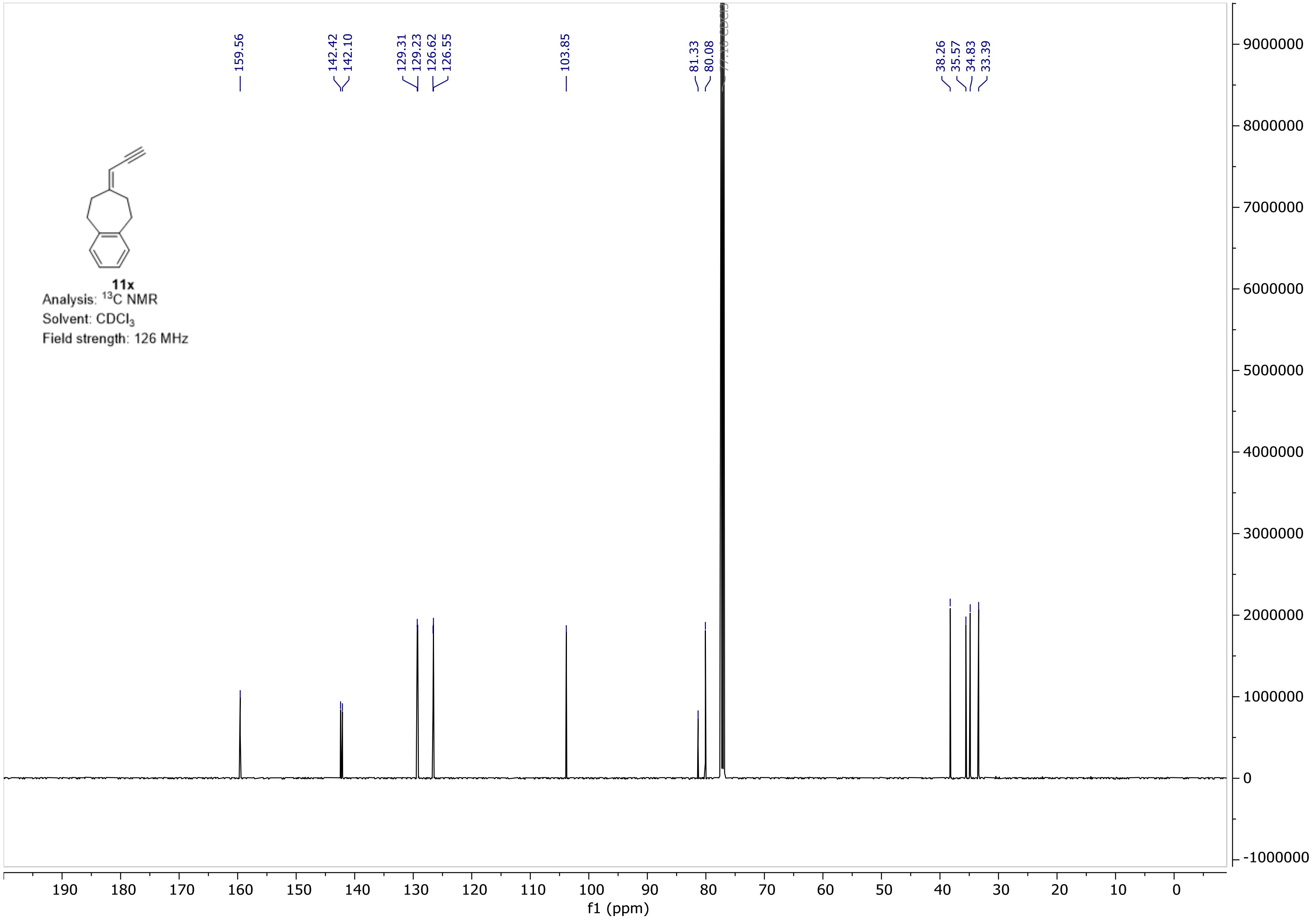
159.56
142.42
142.10
129.31
129.23
126.62
126.55
103.85
81.33
80.08
38.26
35.57
34.83
33.39
11x
Analysis: ^{13}C NMR
Solvent: $CDCl_3$
Field strength: 126 MHz
f1 (ppm)

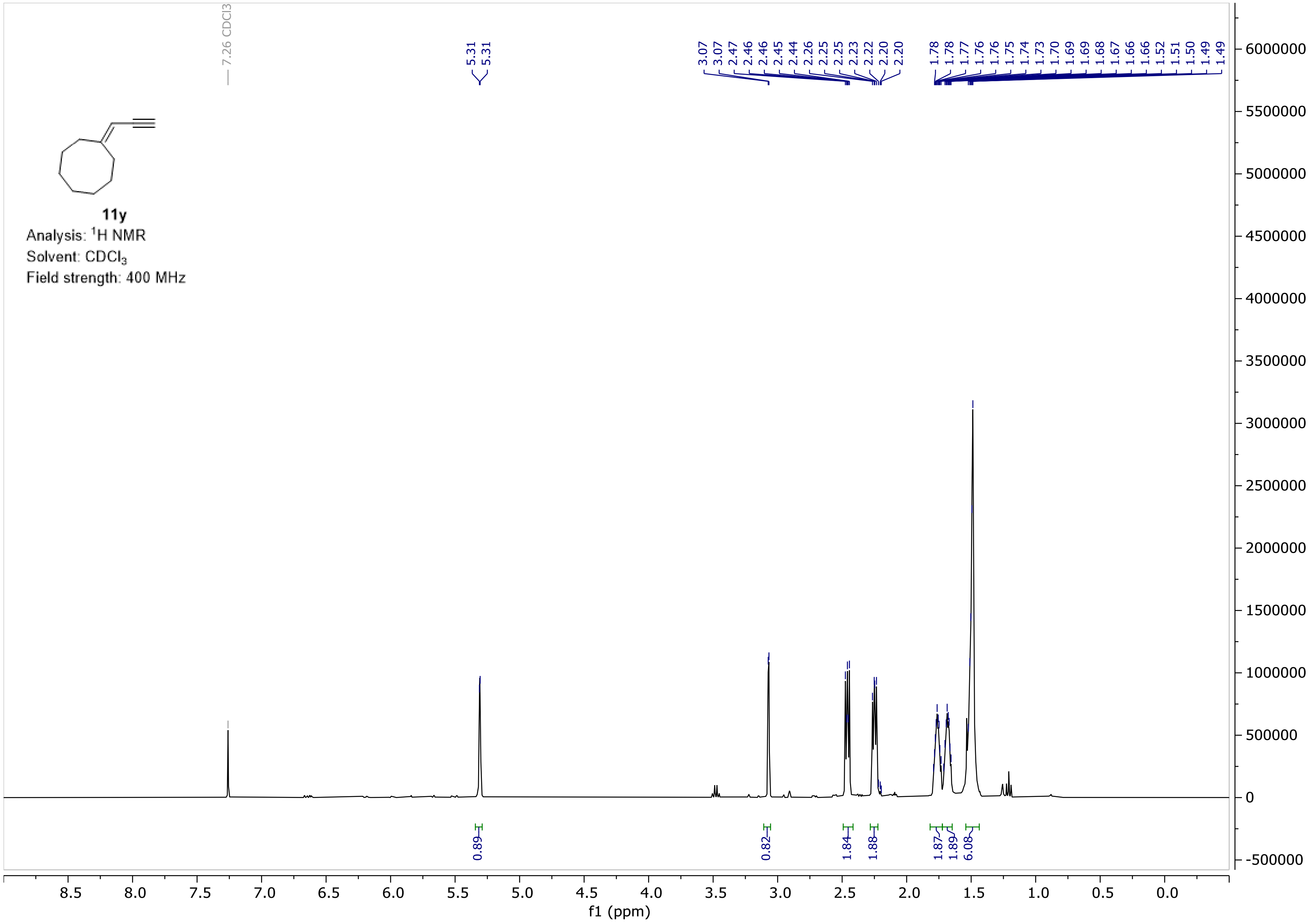
7.26 CDCl3
5.31
5.31
3.07
3.07
2.47
2.46
2.46
2.45
2.44
2.26
2.25
2.25
2.23
2.22
2.20
2.20
1.78
1.78
1.77
1.76
1.76
1.75
1.74
1.73
1.70
1.69
1.69
1.68
1.67
1.66
1.66
1.52
1.51
1.50
1.49
1.49
11y
Analysis: ^{1}H NMR
Solvent: $CDCl_3$
Field strength: 400 MHz
0.89
0.82
1.84
1.88
1.87
1.89
6.08
f1 (ppm)

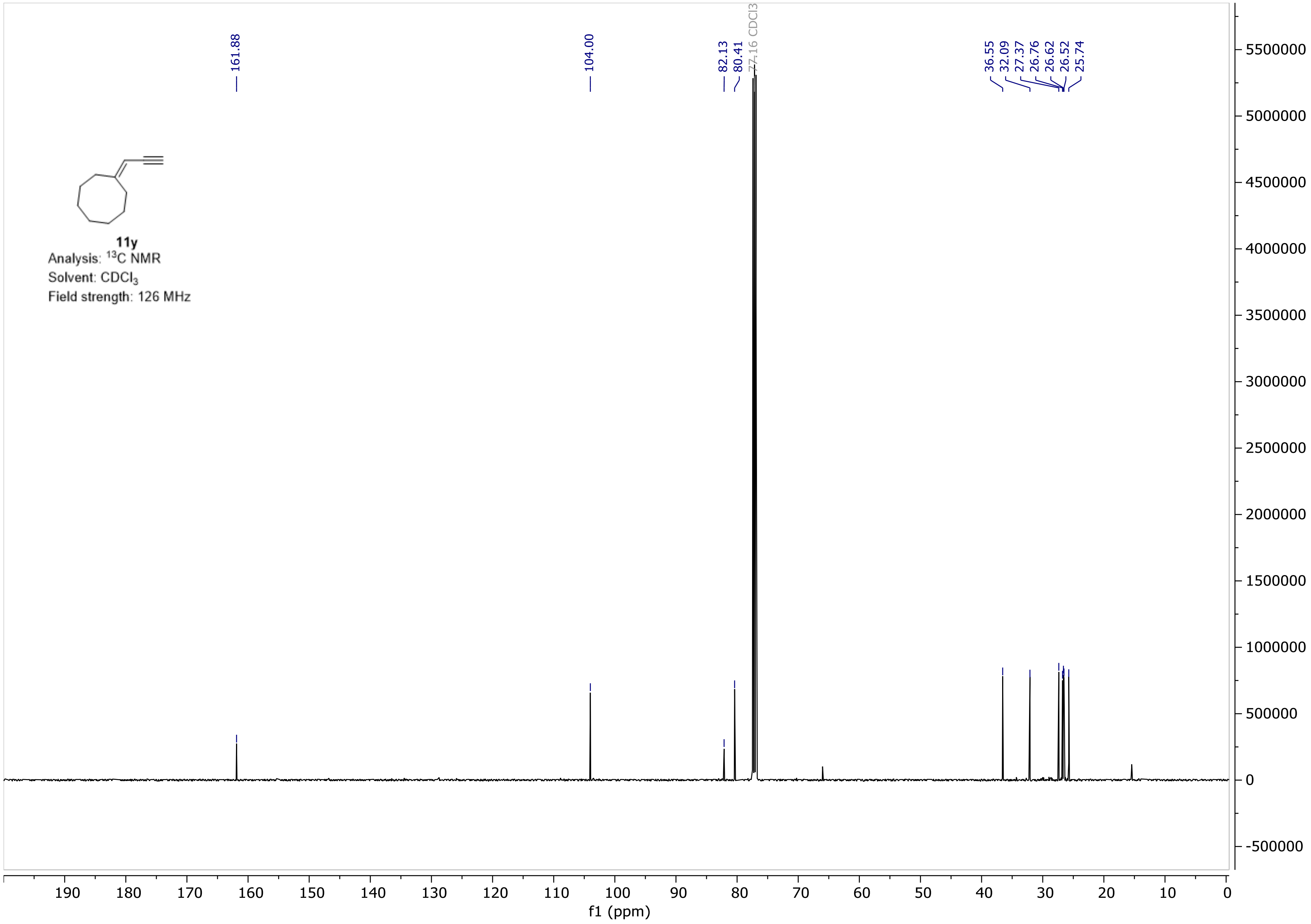

161.88
104.00
82.13
80.41
77.16 CDCl3
36.55
32.09
27.37
26.76
26.62
26.52
25.74
11y
Analysis: ^{13}C NMR
Solvent: $CDCl_3$
Field strength: 126 MHz
f1 (ppm)

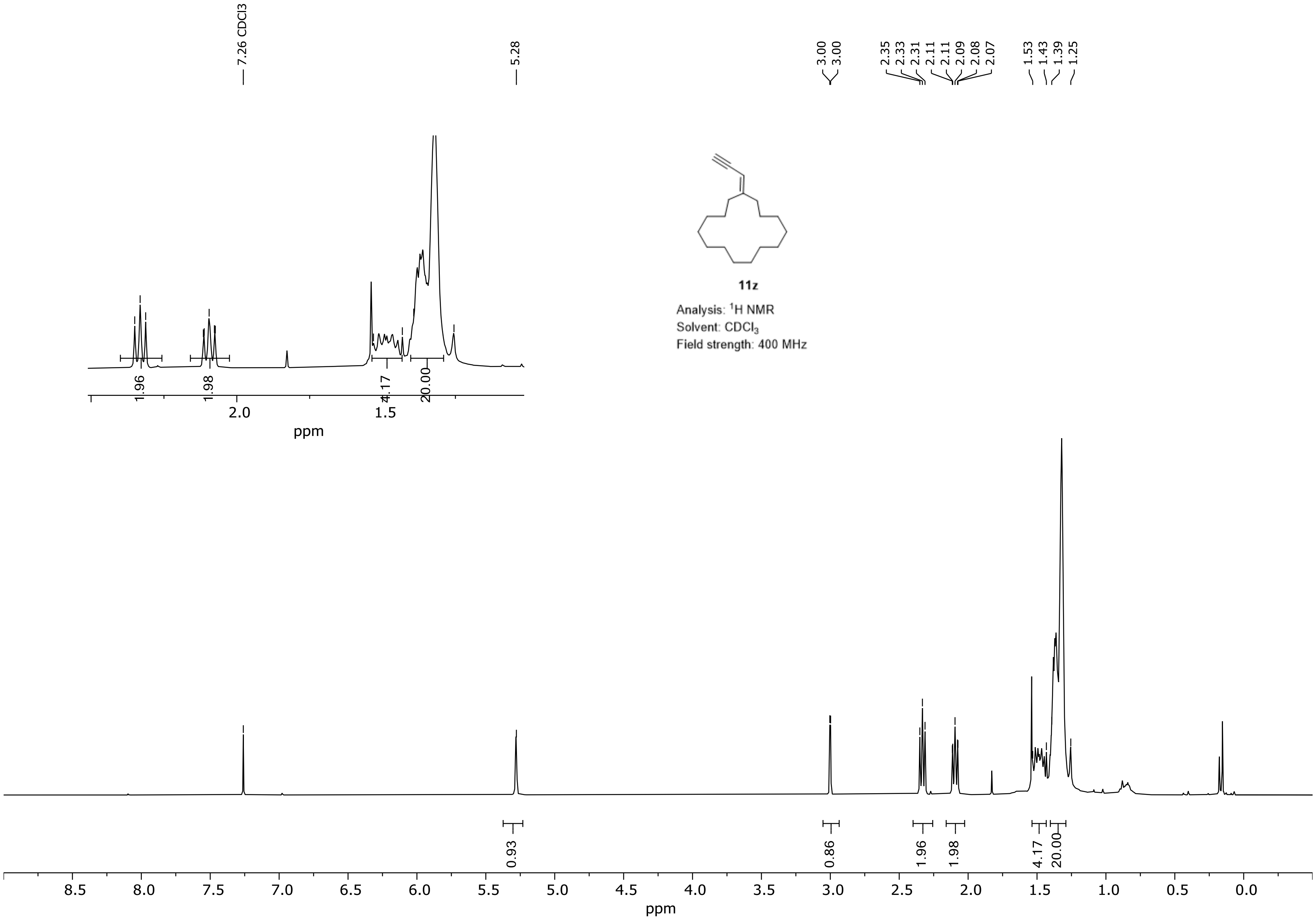

7.26 CDCl3
5.28
3.00
3.00
2.35
2.33
2.31
2.11
2.11
2.09
2.08
2.07
1.53
1.43
1.39
1.25
11z
Analysis: 1H NMR
Solvent: CDCl3
Field strength: 400 MHz
1.96
1.98
4.17
20.00
0.93
0.86
ppm

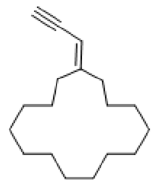

11z

Analysis: ^{13}C NMR
Solvent: $CDCl_3$
Field strength: 101 MHz

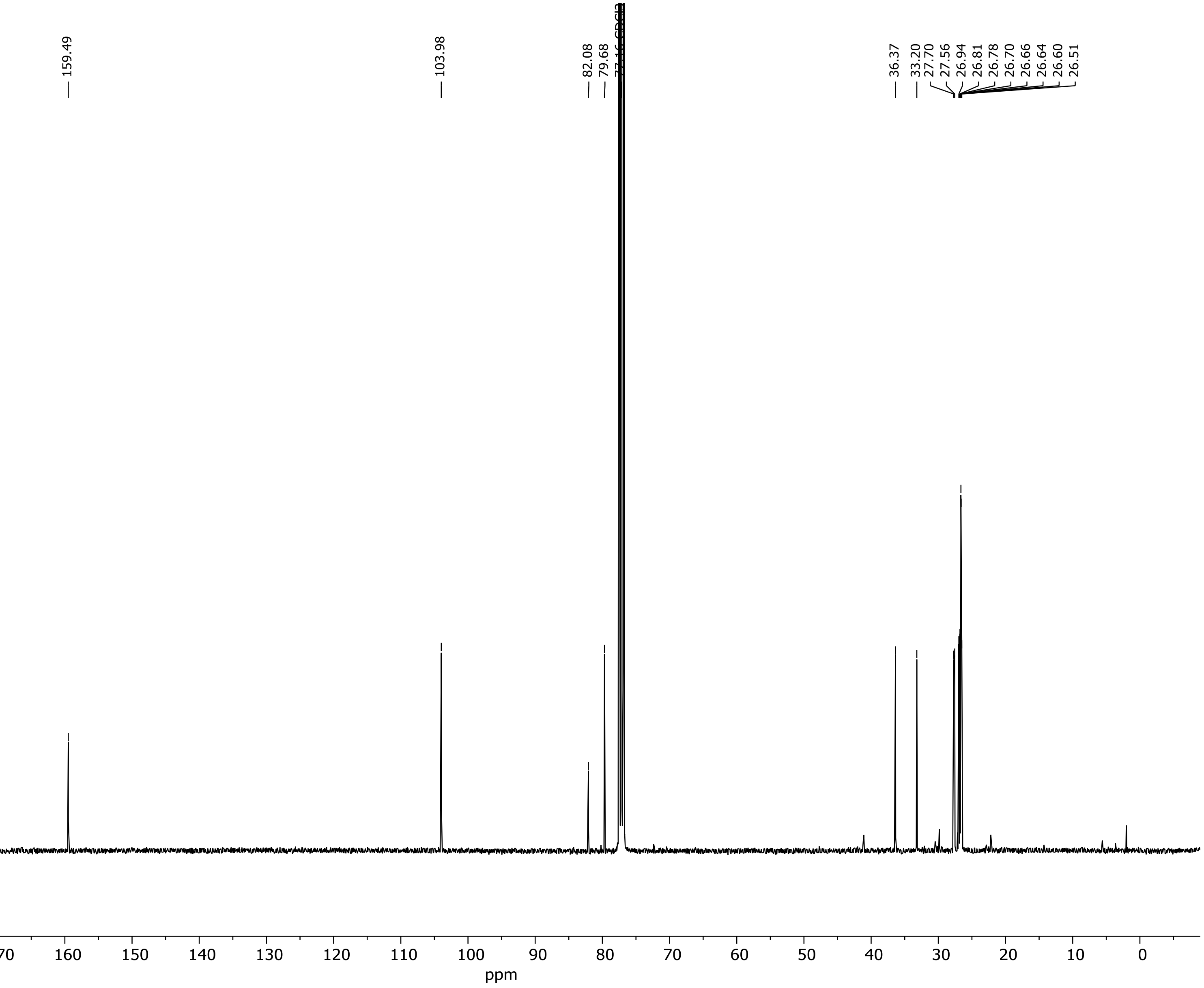

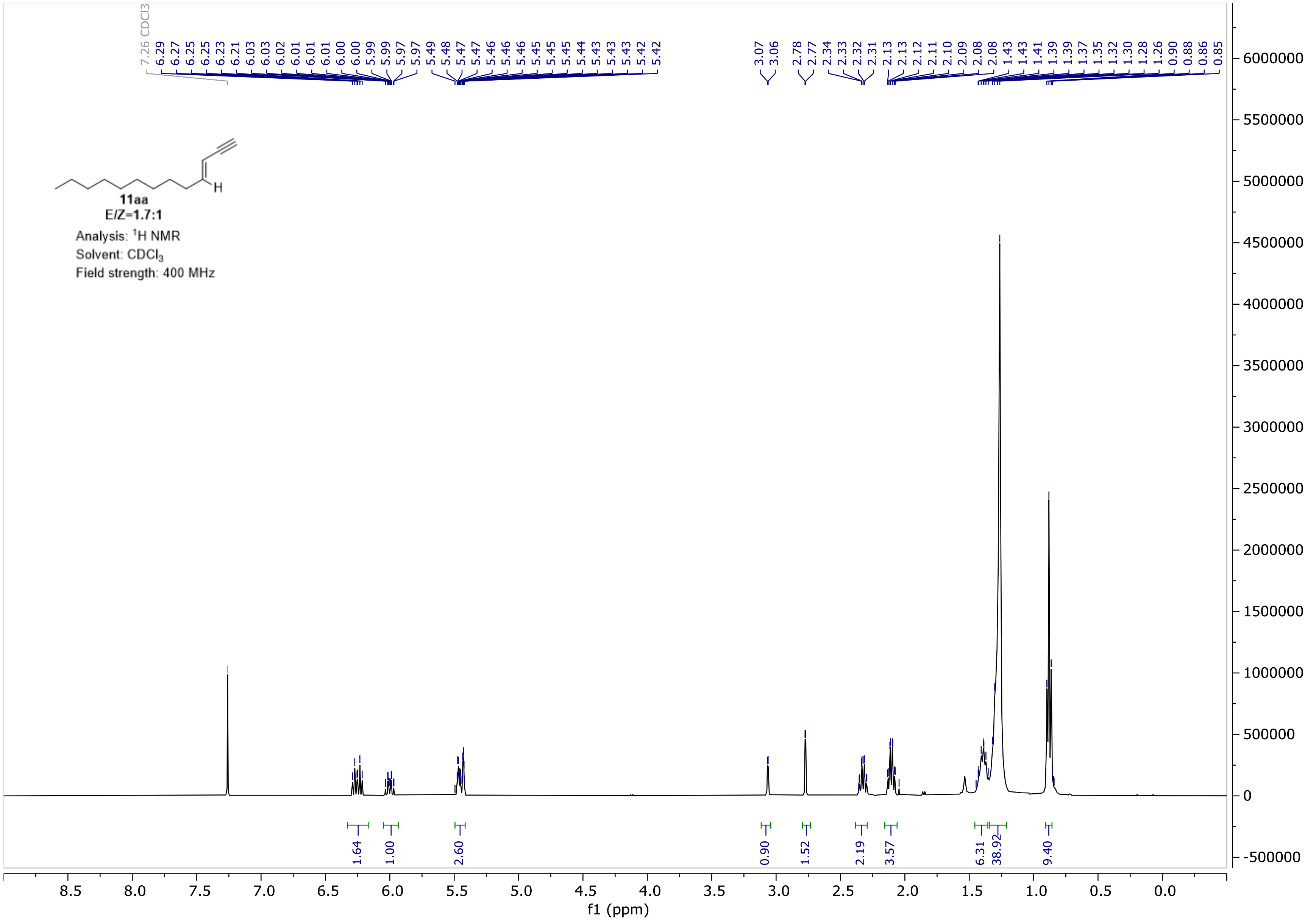
11aa
E/Z=1.7:1
Analysis: 1H NMR
Solvent: CDCl3
Field strength: 400 MHz
f1 (ppm)

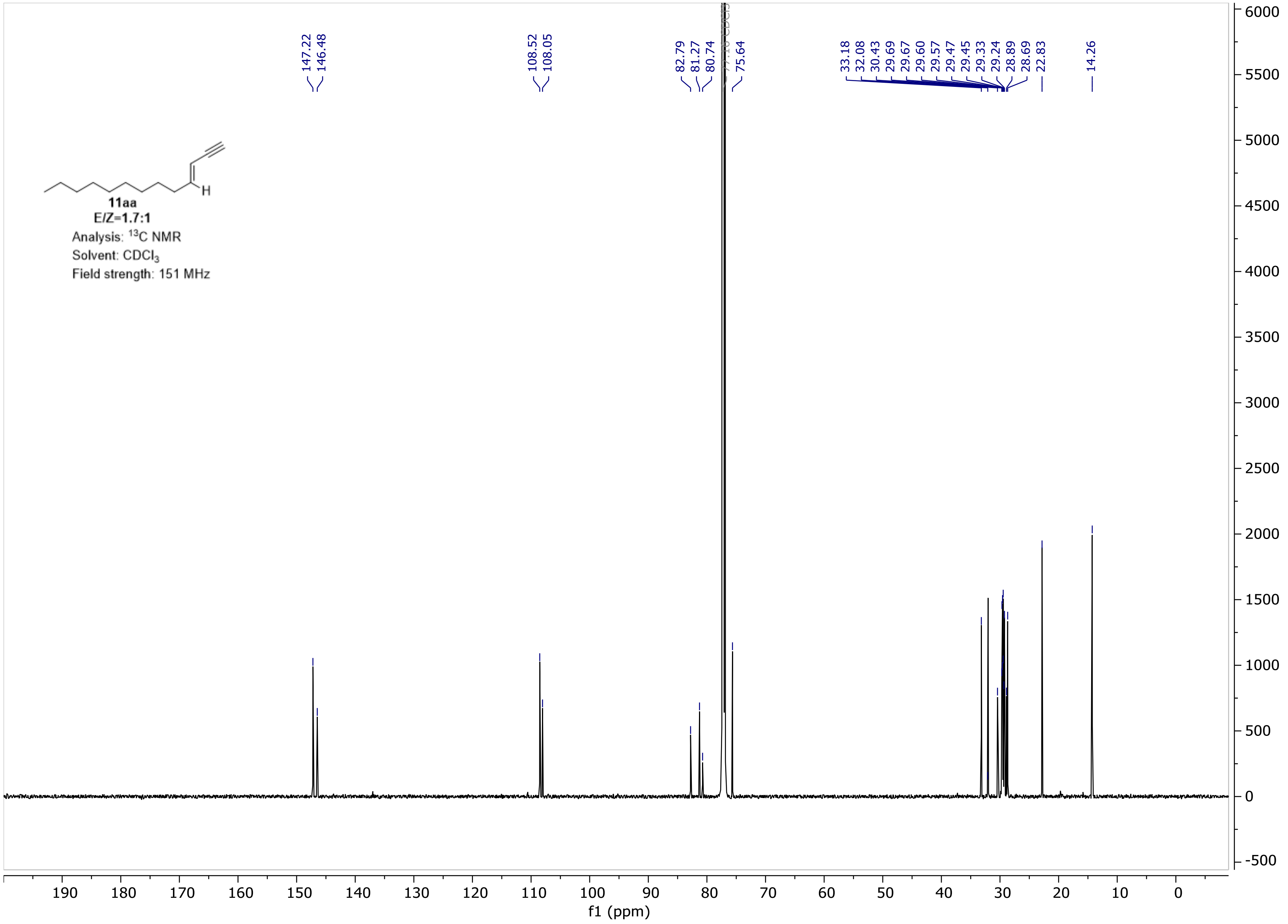

147.22
146.48
108.52
108.05
82.79
81.27
80.74
75.64
33.18
32.08
30.43
29.69
29.67
29.60
29.57
29.47
29.45
29.33
29.24
28.89
28.69
22.83
14.26
H
11aa
E/Z=1.7:1
Analysis: 13C NMR
Solvent: CDCl3
Field strength: 151 MHz
f1 (ppm)

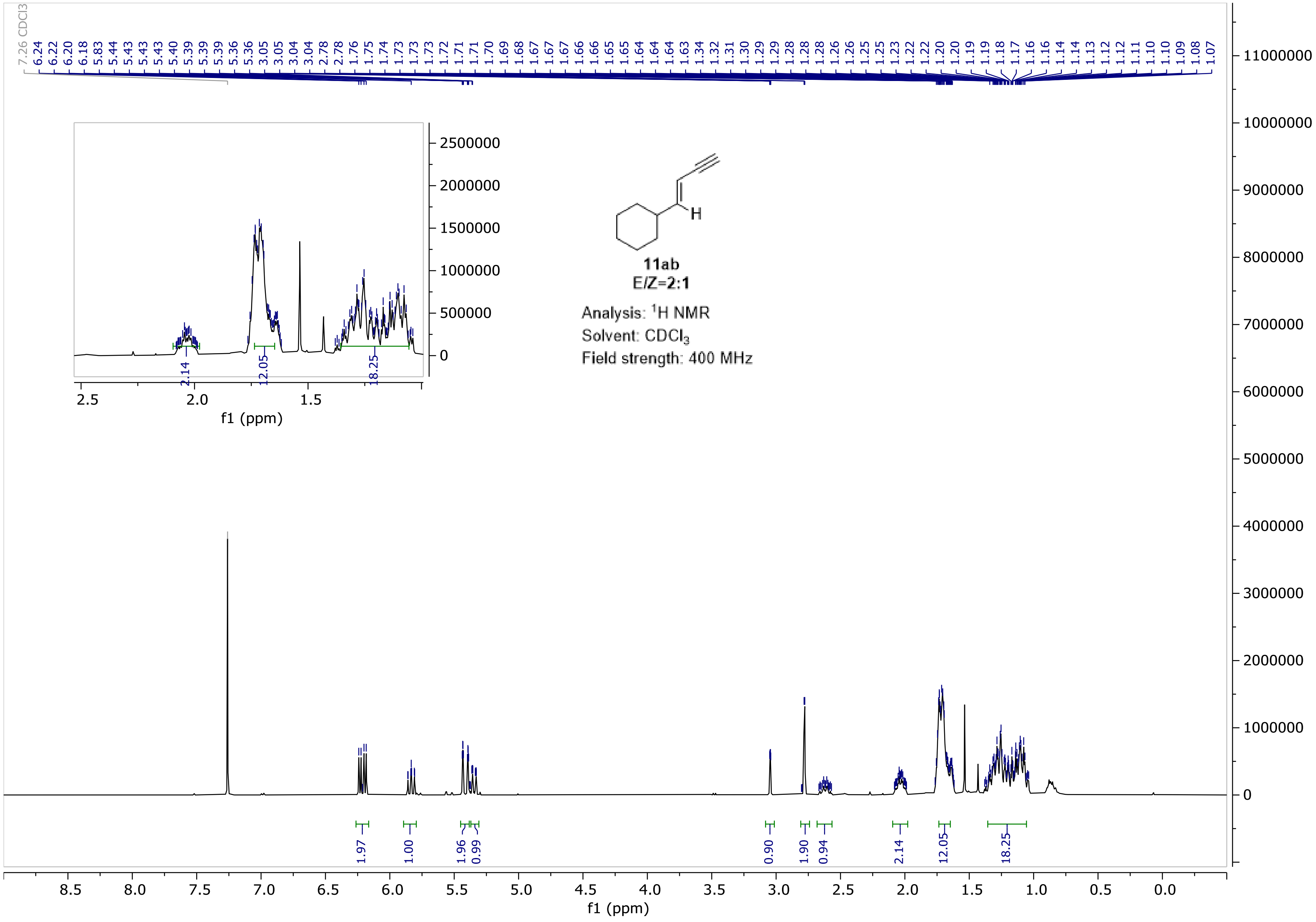

7.26 CDCl3
6.24 6.22 6.20 6.18 5.83 5.44 5.43 5.43 5.40 5.39 5.39 5.39 5.36 5.36 3.05 3.05 3.04 3.04 2.78 2.78 1.76 1.75 1.74 1.73 1.73 1.73 1.72 1.71 1.71 1.70 1.69 1.68 1.67 1.67 1.67 1.66 1.65 1.65 1.64 1.64 1.64 1.63 1.34 1.32 1.31 1.30 1.29 1.29 1.28 1.28 1.26 1.26 1.25 1.25 1.23 1.22 1.22 1.20 1.20 1.19 1.19 1.18 1.17 1.16 1.16 1.14 1.14 1.13 1.12 1.12 1.11 1.10 1.10 1.09 1.08 1.07
H
11ab
E/Z=2:1
Analysis: ^{1}H NMR
Solvent: $CDCl_3$
Field strength: 400 MHz
2.14
12.05
18.25
f1 (ppm)
1.97
1.00
1.96
0.99
0.90
1.90
0.94
2.14
12.05
18.25
f1 (ppm)

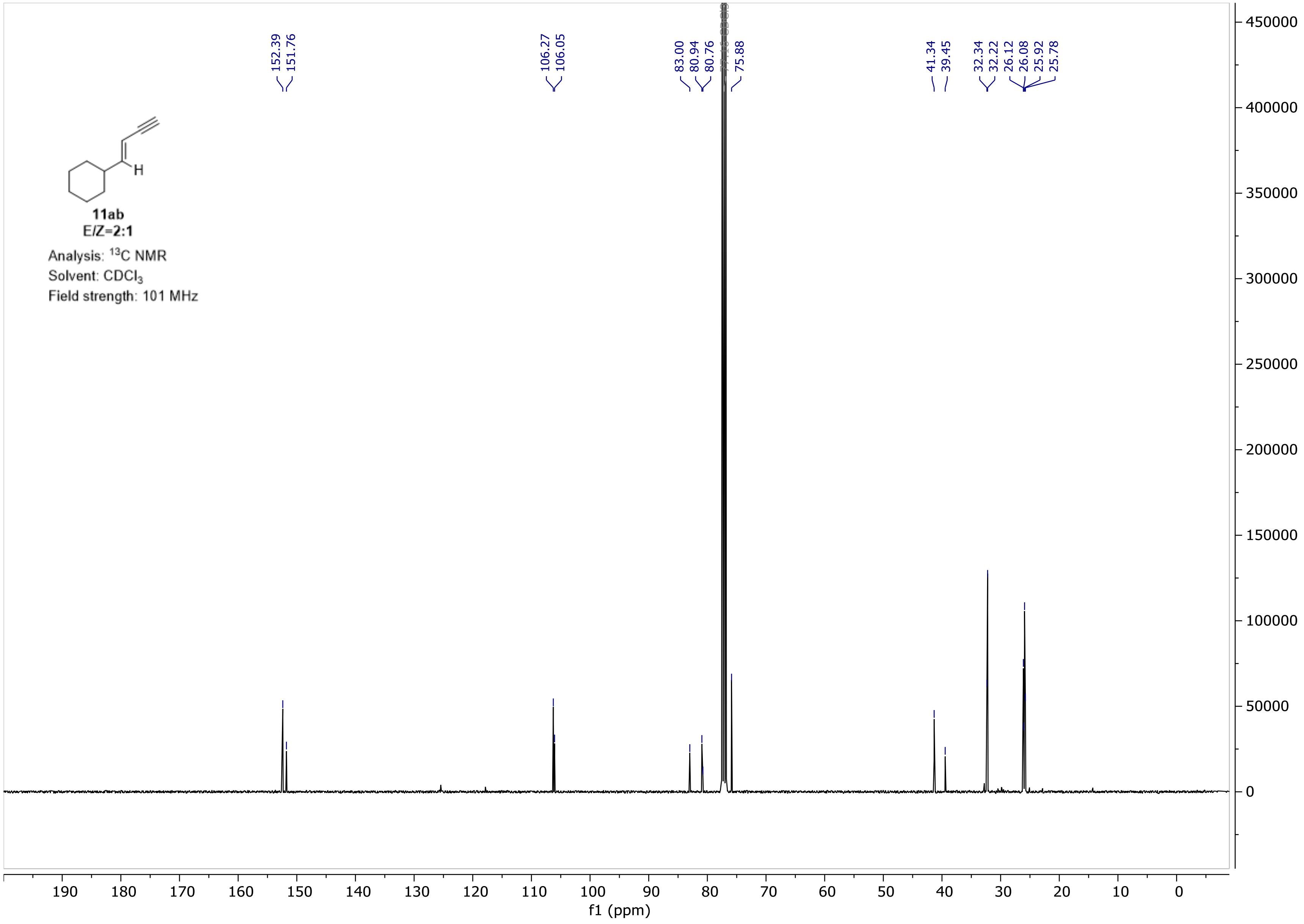

11ab
E/Z=2:1
Analysis: ¹³C NMR
Solvent: CDCl3
Field strength: 101 MHz
152.39
151.76
106.27
106.05
83.00
80.94
80.76
75.88
41.34
39.45
32.34
32.22
26.12
26.08
25.92
25.78
f1 (ppm)

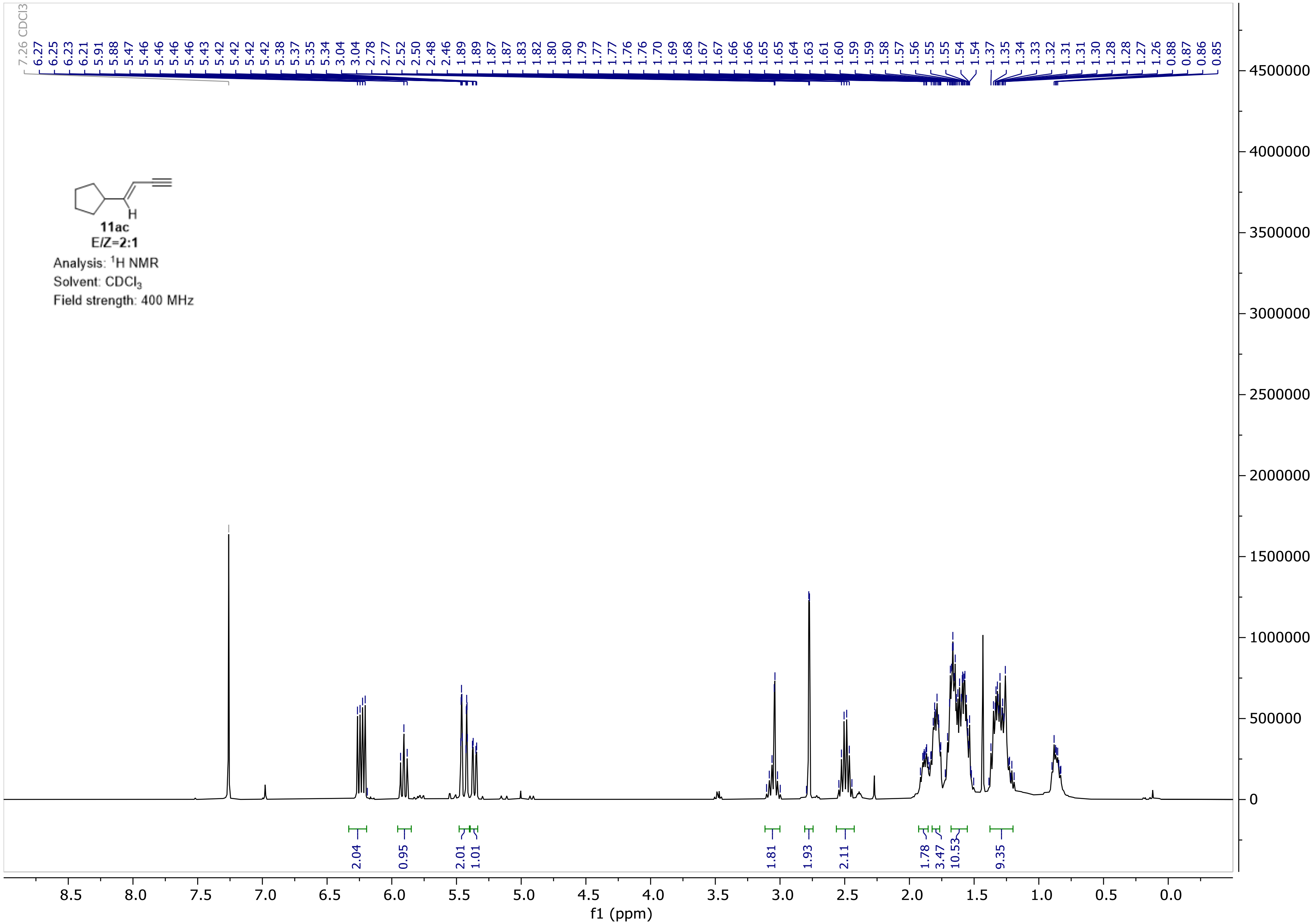
11ac
E/Z=2:1
Analysis: ¹H NMR
Solvent: CDCl3
Field strength: 400 MHz
f1 (ppm)

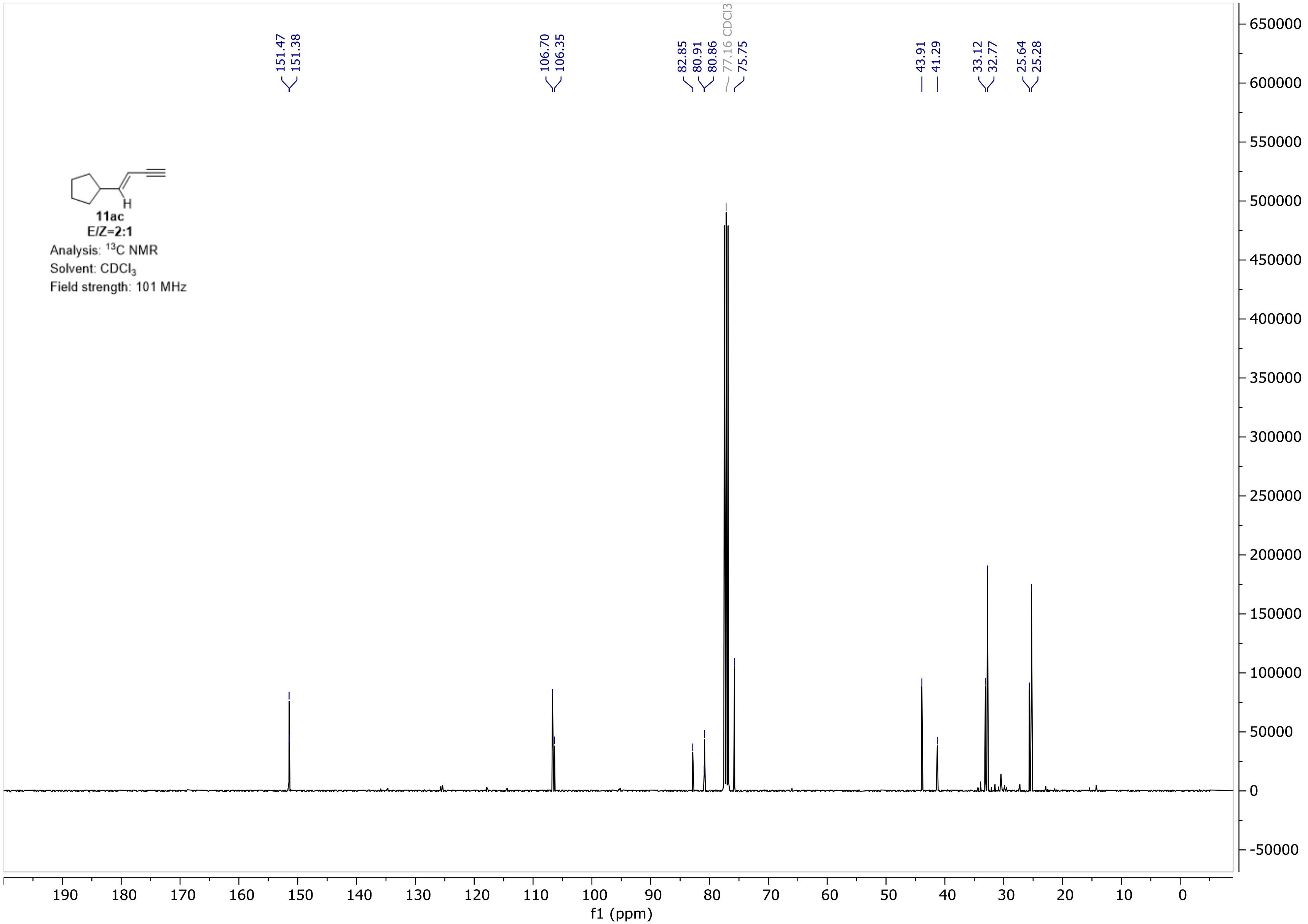

151.47
151.38
106.70
106.35
82.85
80.91
80.86
77.16 CDCl3
75.75
43.91
41.29
33.12
32.77
25.64
25.28
H
11ac
E/Z=2:1
Analysis: 13C NMR
Solvent: CDCl3
Field strength: 101 MHz
f1 (ppm)

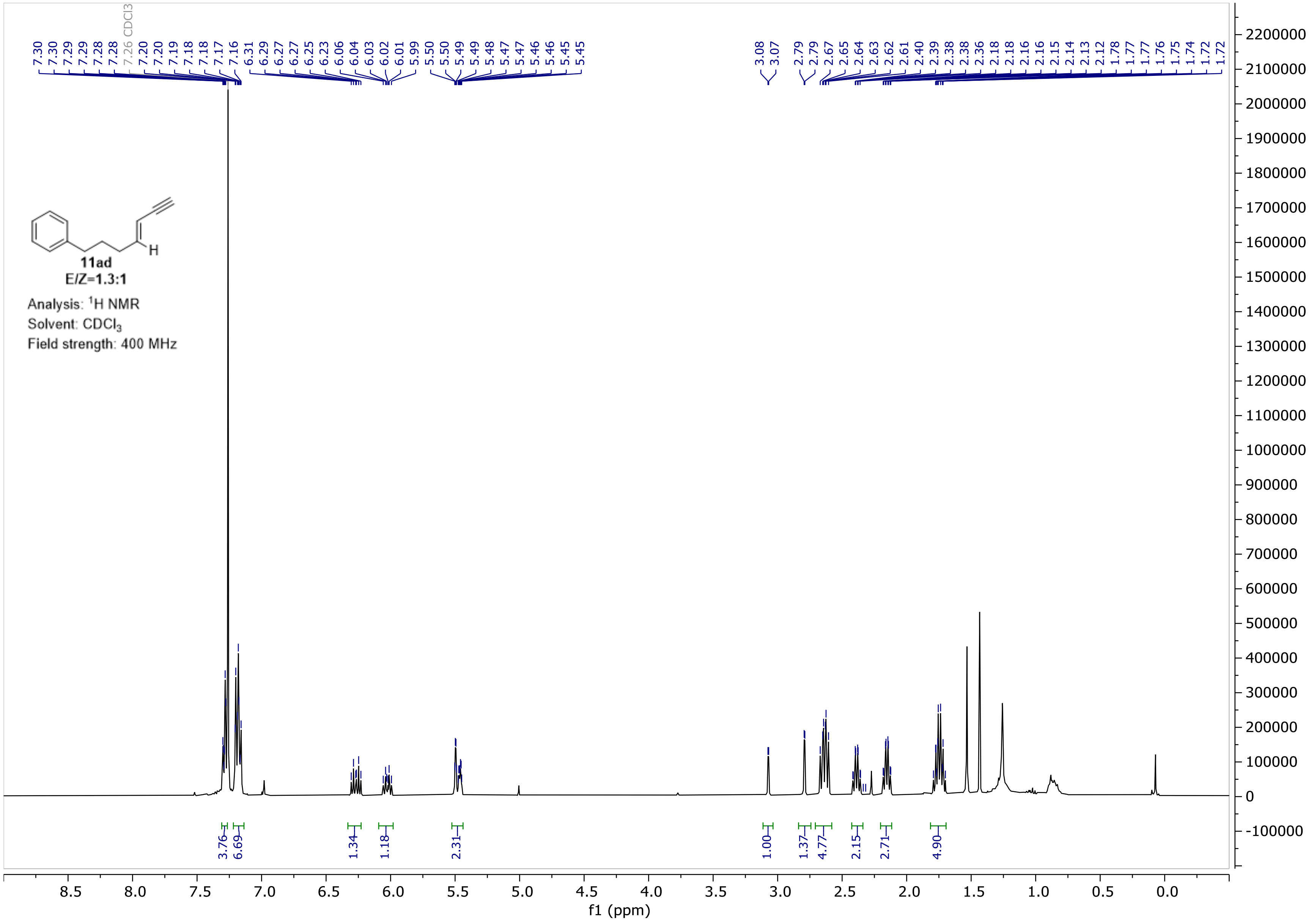

11ad
E/Z=1.3:1
Analysis: ¹H NMR
Solvent: CDCl3
Field strength: 400 MHz
f1 (ppm)

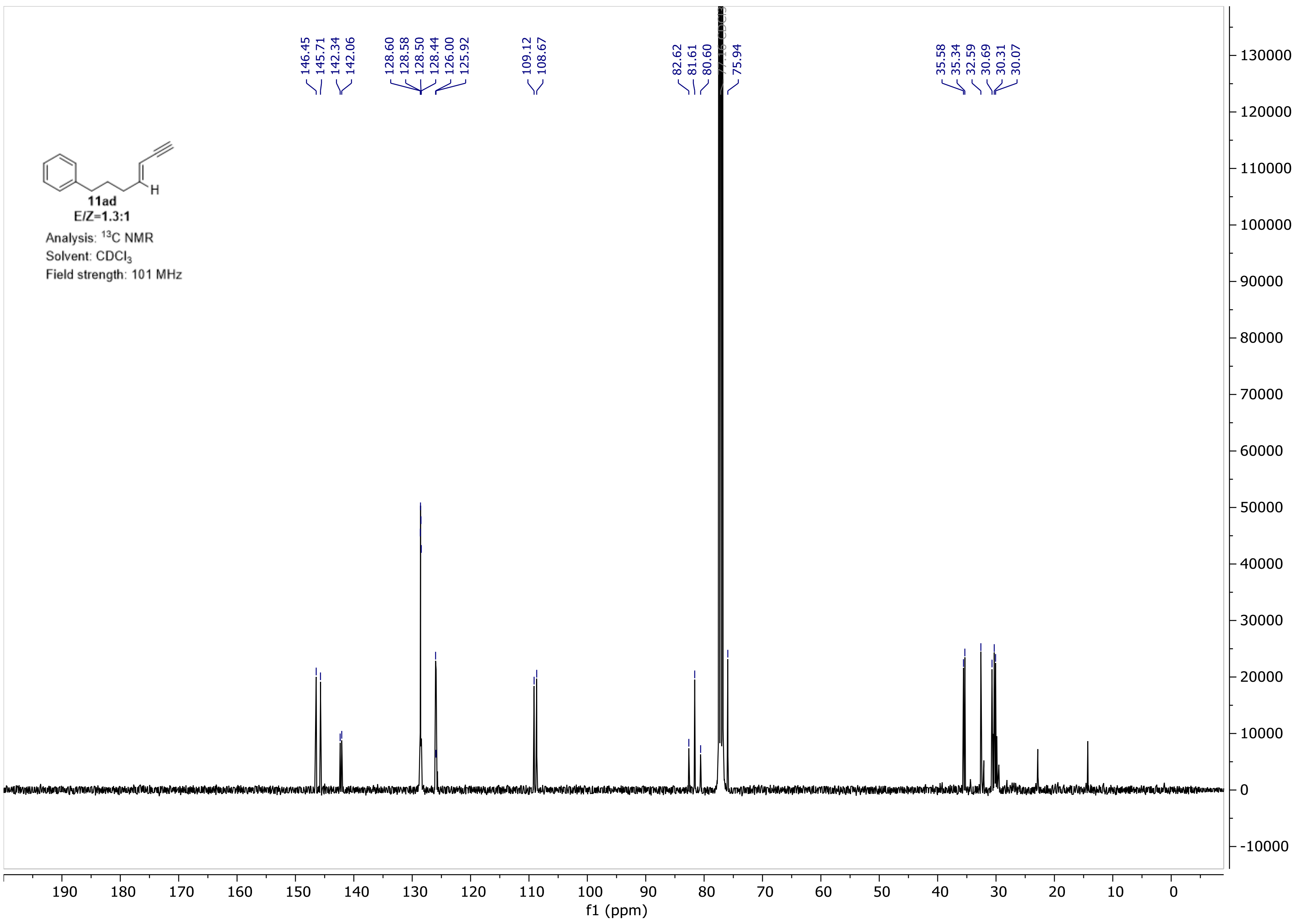

11ad
E/Z=1.3:1
Analysis: 13C NMR
Solvent: CDCl3
Field strength: 101 MHz
146.45
145.71
142.34
142.06
128.60
128.58
128.50
128.44
126.00
125.92
109.12
108.67
82.62
81.61
80.60
75.94
35.58
35.34
32.59
30.69
30.31
30.07
f1 (ppm)

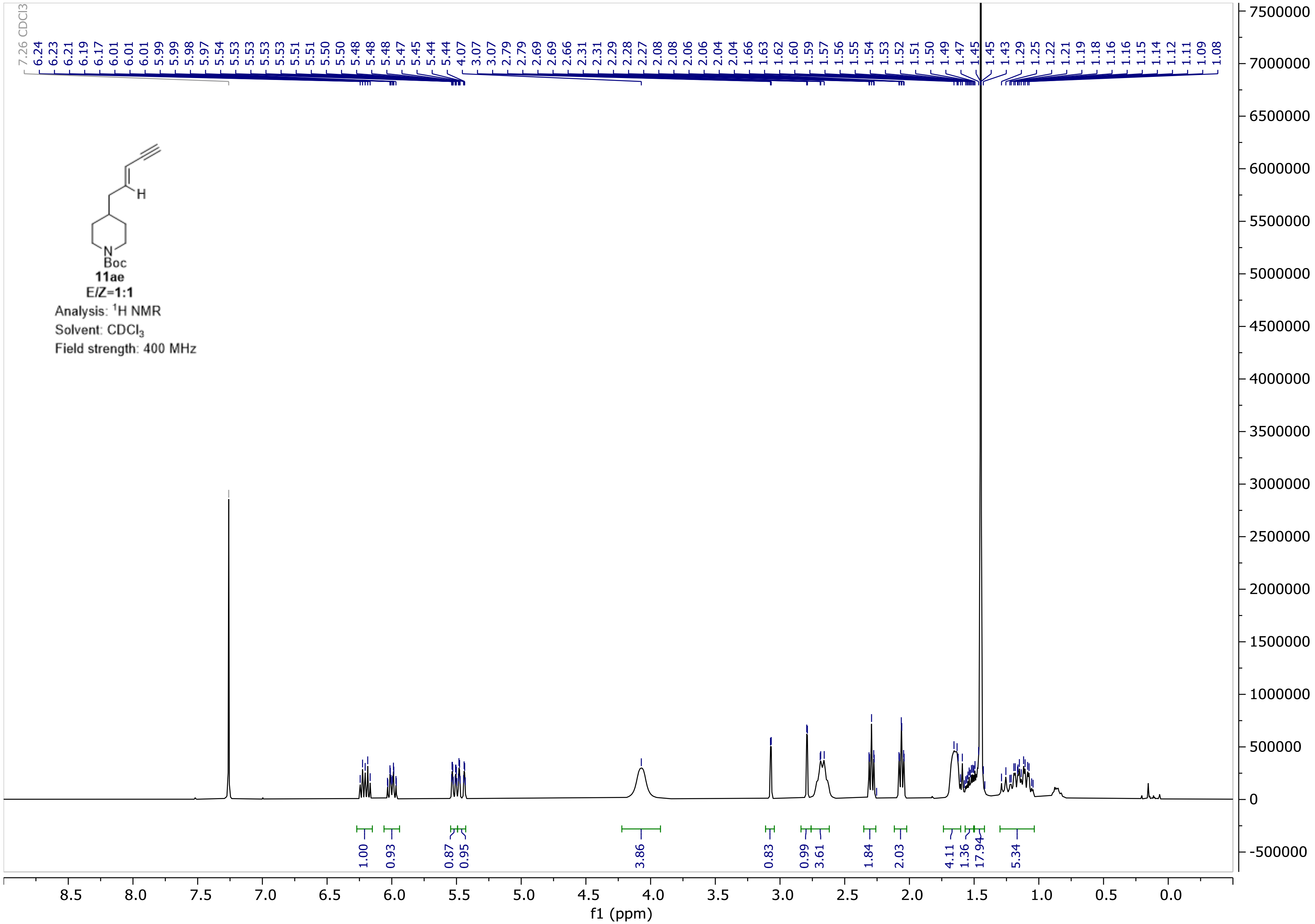
7.26 CDCl3
6.24 6.23 6.21 6.19 6.17 6.01 6.01 5.99 5.99 5.98 5.97 5.54 5.53 5.53 5.53 5.53 5.51 5.51 5.50 5.48 5.48 5.48 5.47 5.45 5.44 5.44 4.07 3.07 3.07 2.79 2.79 2.69 2.69 2.66 2.31 2.31 2.29 2.28 2.27 2.08 2.08 2.06 2.06 2.04 2.04 1.66 1.63 1.62 1.60 1.59 1.57 1.56 1.55 1.54 1.53 1.52 1.51 1.50 1.49 1.47 1.45 1.43 1.29 1.25 1.22 1.21 1.19 1.18 1.16 1.16 1.15 1.14 1.12 1.11 1.09 1.08
H
N
Boc
11ae
E/Z=1:1
Analysis: 1H NMR
Solvent: CDCl3
Field strength: 400 MHz
1.00 0.93 0.87 0.95 3.86 0.83 0.99 3.61 1.84 2.03 4.11 1.36 17.94 5.34
8.5 8.0 7.5 7.0 6.5 6.0 5.5 5.0 4.5 4.0 3.5 3.0 2.5 2.0 1.5 1.0 0.5 0.0
f1 (ppm)
7500000 7000000 6500000 6000000 5500000 5000000 4500000 4000000 3500000 3000000 2500000 2000000 1500000 1000000 500000 0 -500000

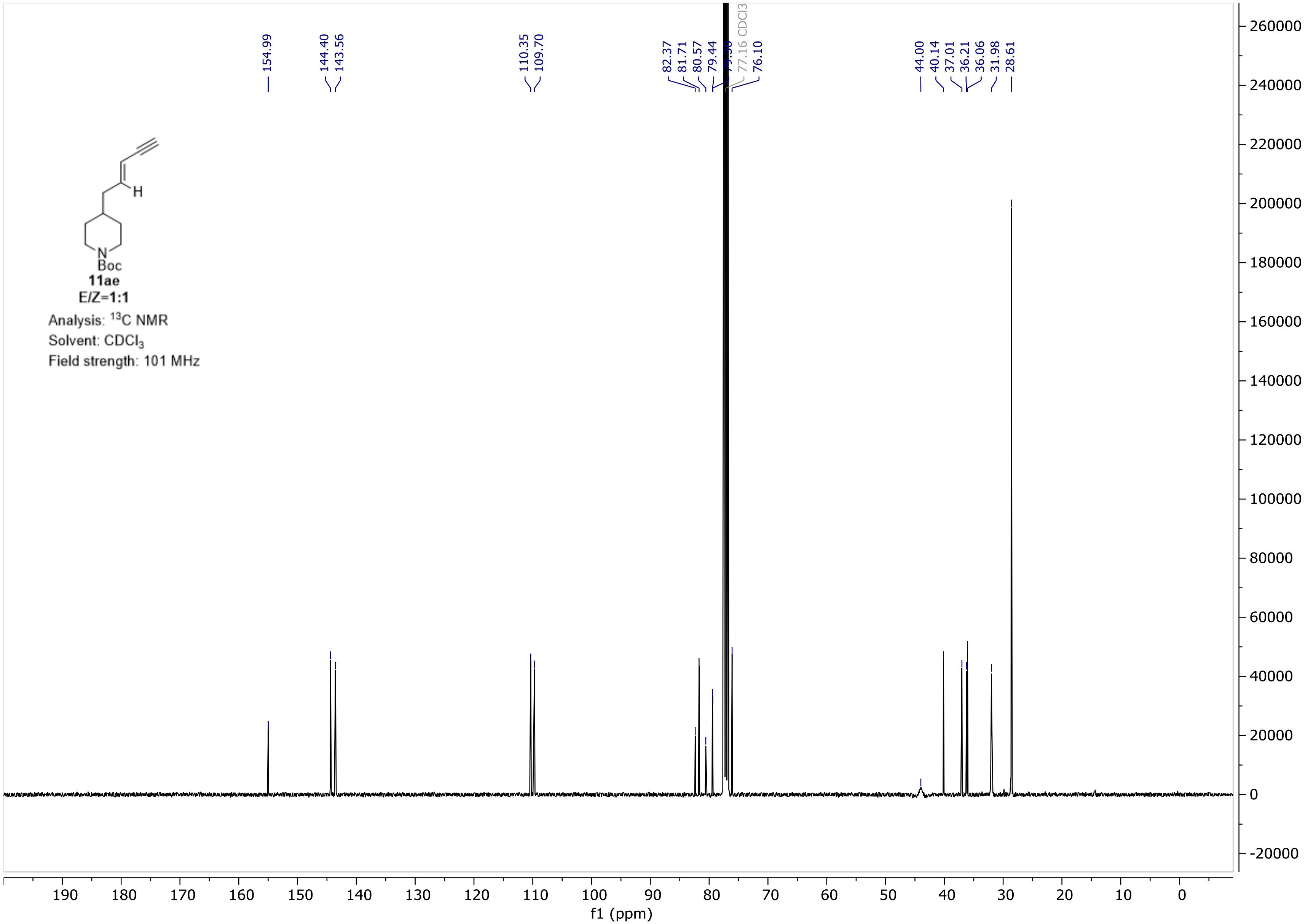

154.99
144.40
143.56
110.35
109.70
82.37
81.71
80.57
79.44
77.16 CDCl3
76.10
44.00
40.14
37.01
36.21
36.06
31.98
28.61
H
N
Boc
11ae
E/Z=1:1
Analysis: 13C NMR
Solvent: CDCl3
Field strength: 101 MHz
f1 (ppm)

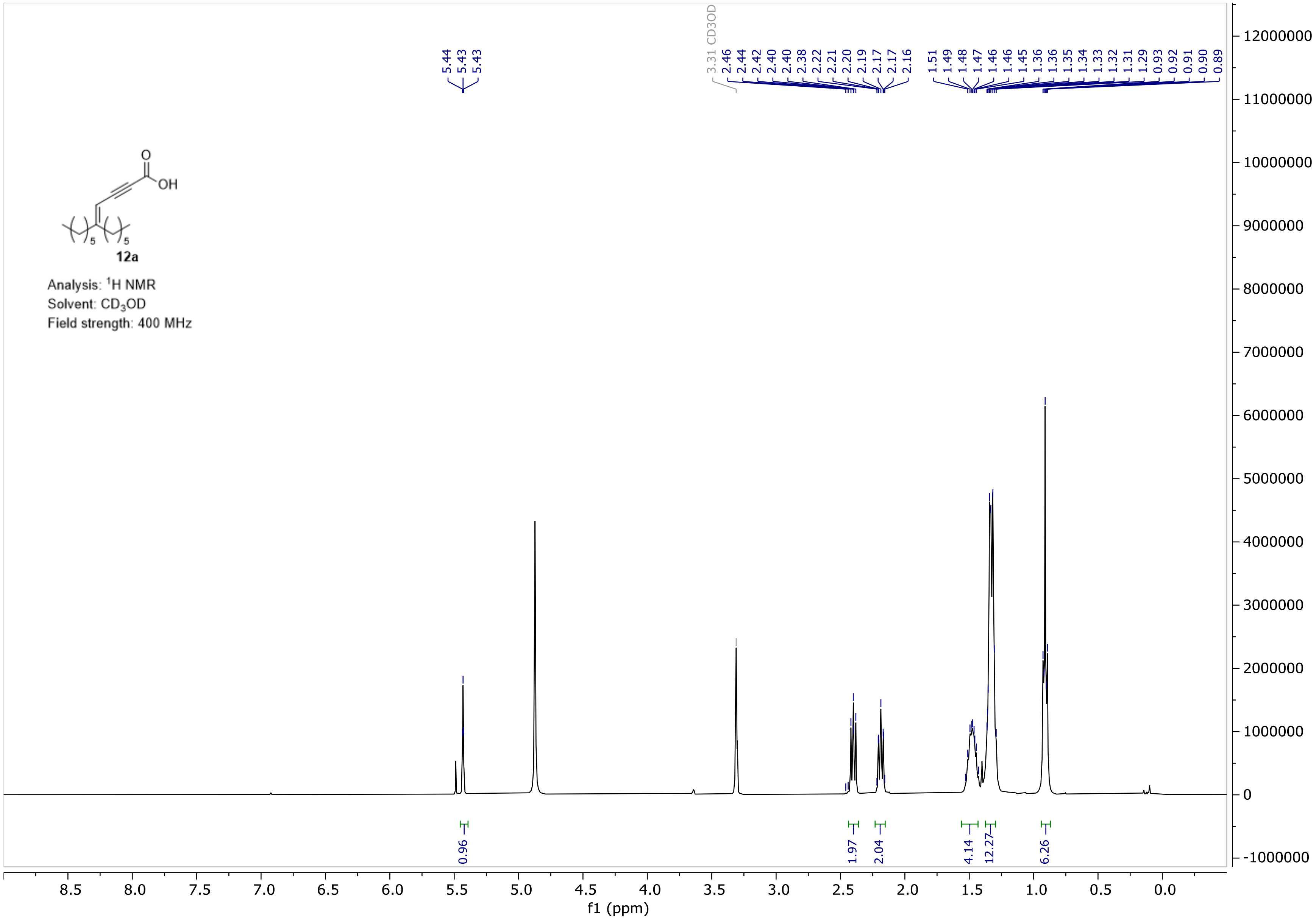

O
OH
5
5
12a
Analysis: ¹H NMR
Solvent: CD₃OD
Field strength: 400 MHz
5.44 5.43 5.43
3.31 CD3OD
2.46 2.44 2.42 2.40 2.40 2.38 2.22 2.21 2.20 2.19 2.17 2.17 2.16
1.51 1.49 1.48 1.47 1.46 1.46 1.45 1.36 1.36 1.35 1.34 1.33 1.32 1.31 1.29 0.93 0.92 0.91 0.90 0.89
0.96
1.97
2.04
4.14
12.27
6.26
f1 (ppm)

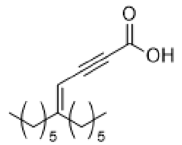

12a

Analysis: ^{13}C NMR
Solvent: CD_3OD
Field strength: 151 MHz

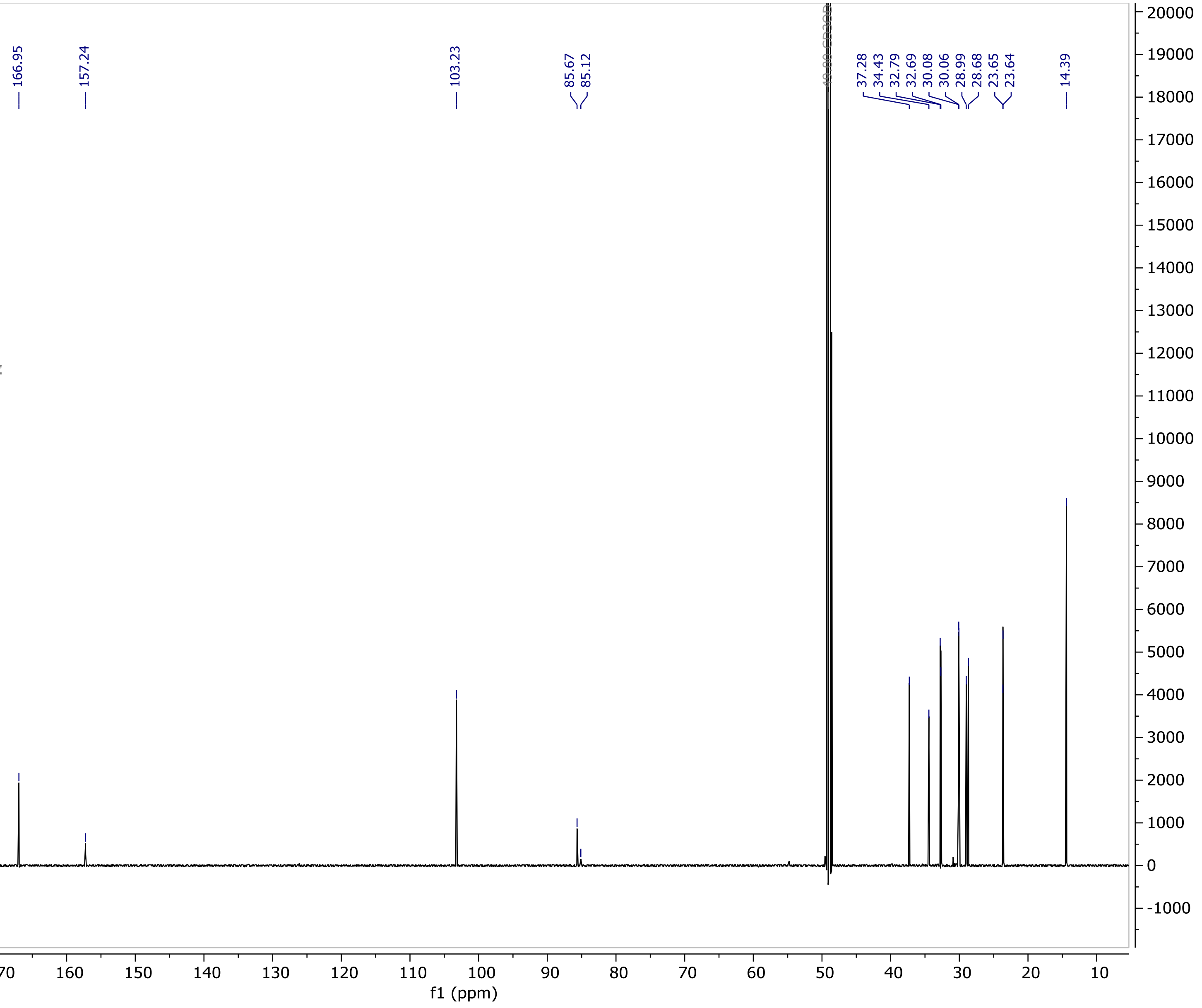

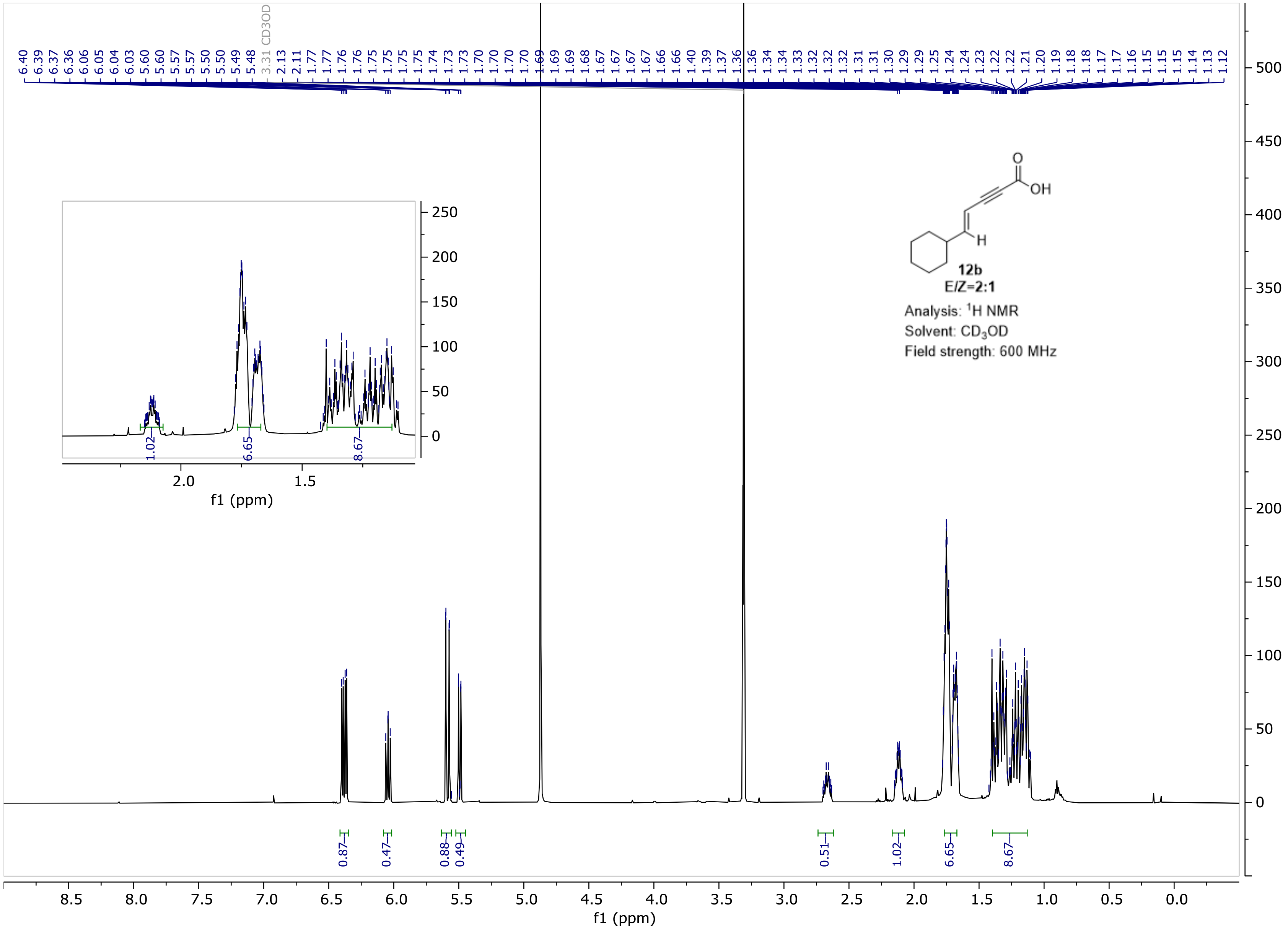
12b
E/Z=2:1
Analysis: ¹H NMR
Solvent: CD₃OD
Field strength: 600 MHz
f1 (ppm)

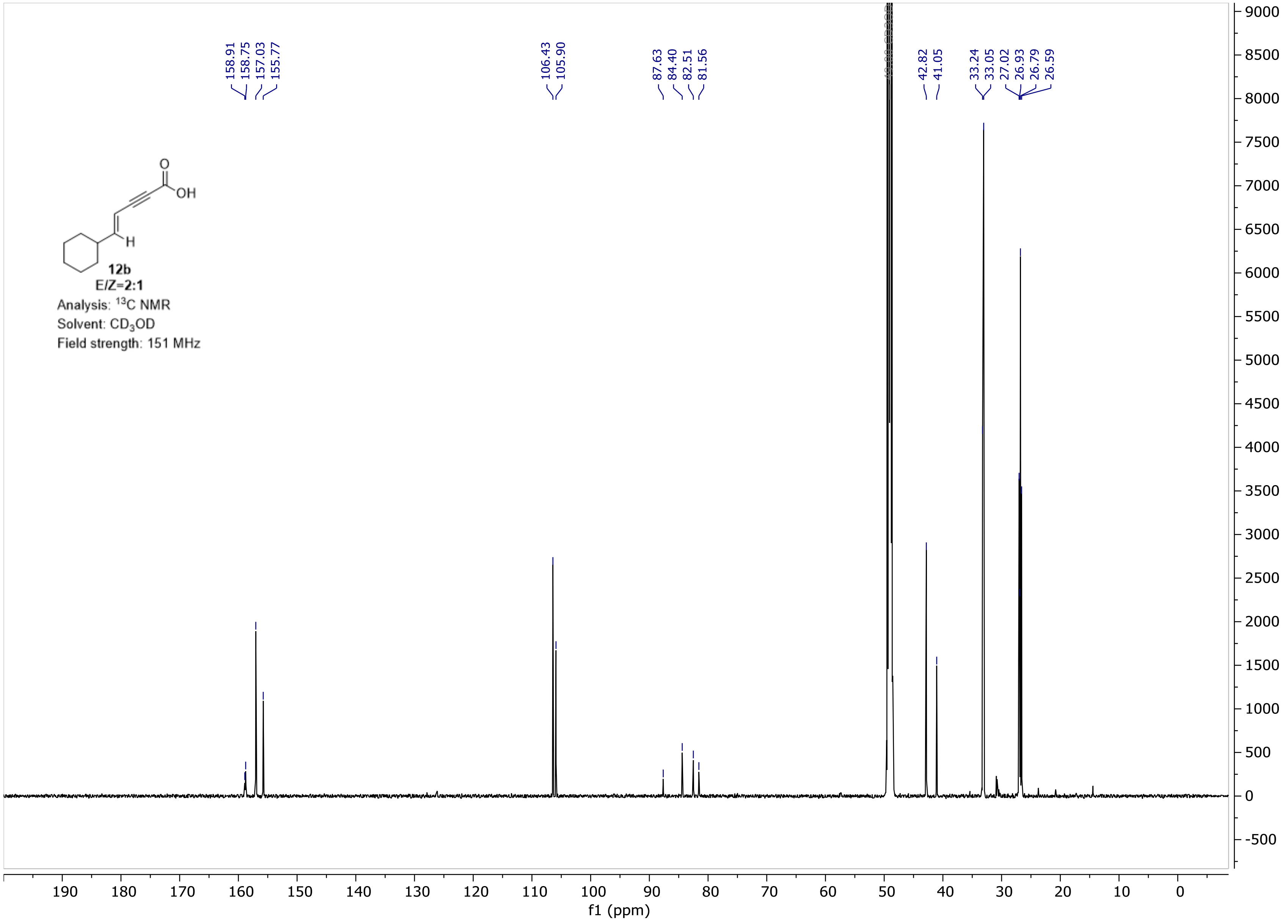
158.91
158.75
157.03
155.77
106.43
105.90
87.63
84.40
82.51
81.56
42.82
41.05
33.24
33.05
27.02
26.93
26.79
26.59
O
OH
H
12b
E/Z=2:1
Analysis: 13C NMR
Solvent: CD3OD
Field strength: 151 MHz
f1 (ppm)

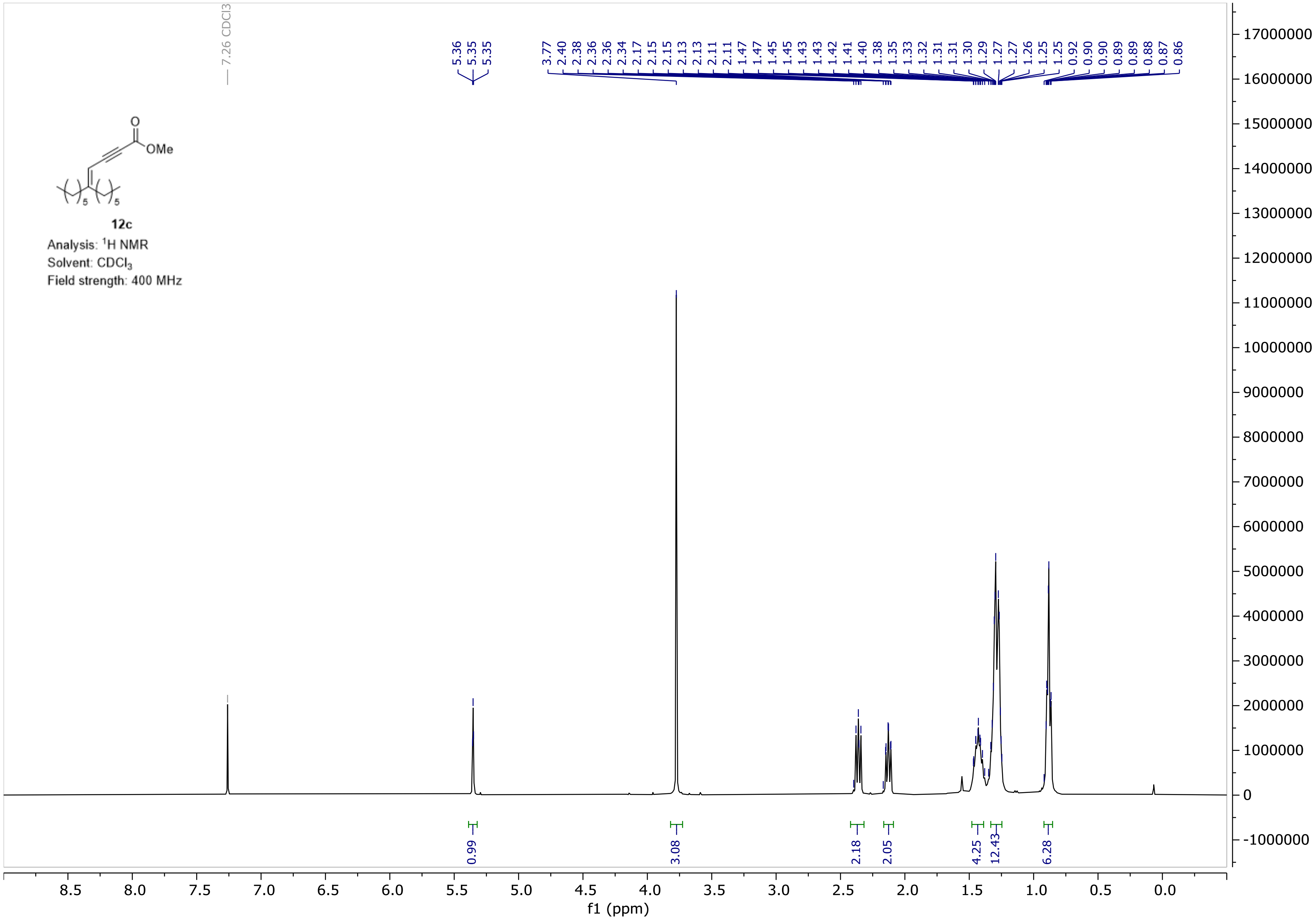

12c

Analysis: ^{1}H NMR

Solvent: $CDCl_3$

Field strength: 400 MHz

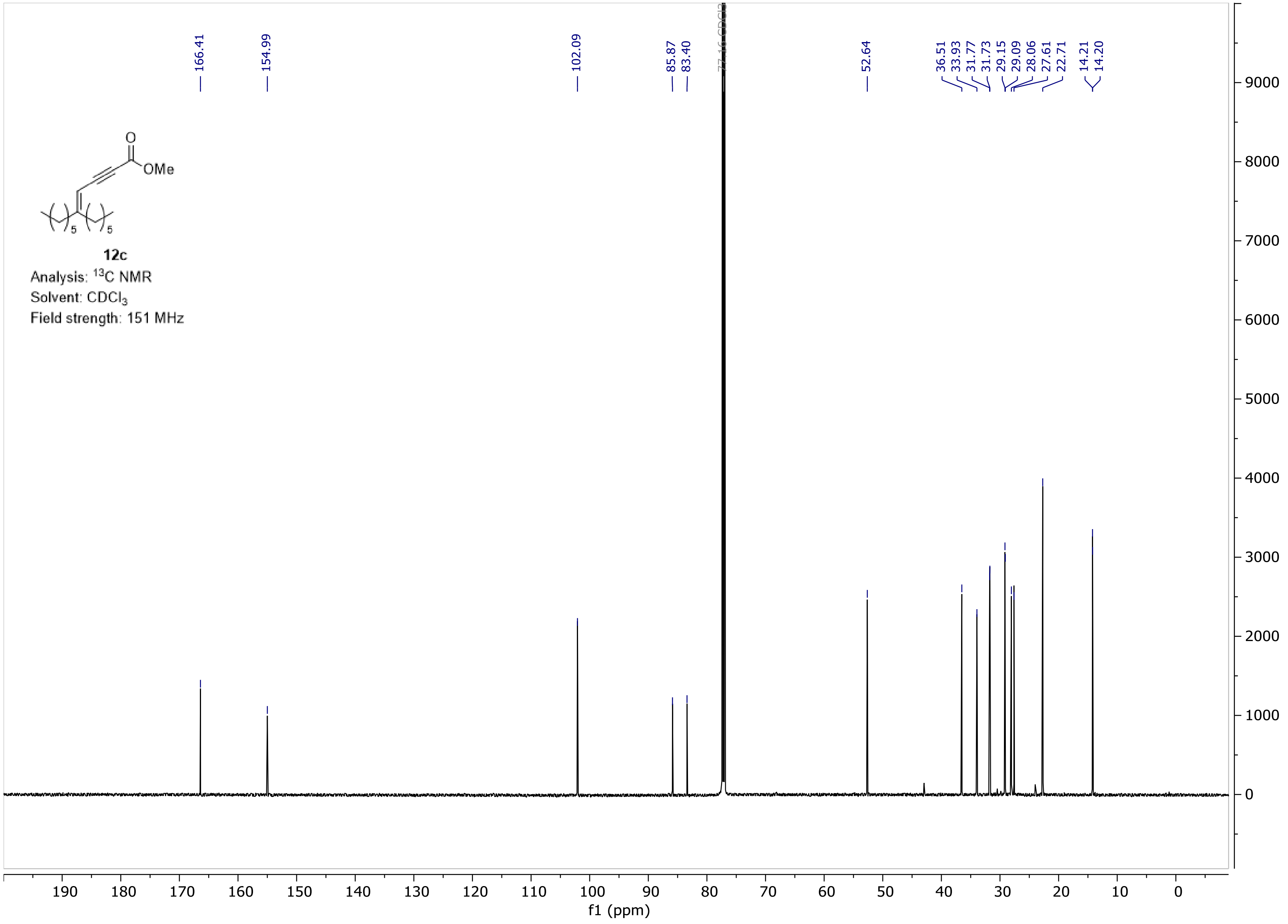

166.41
154.99
102.09
85.87
83.40
77.16 CDCl3
52.64
36.51
33.93
31.77
31.73
29.15
29.09
28.06
27.61
22.71
14.21
14.20
OMe
12c
Analysis: 13C NMR
Solvent: CDCl3
Field strength: 151 MHz
f1 (ppm)

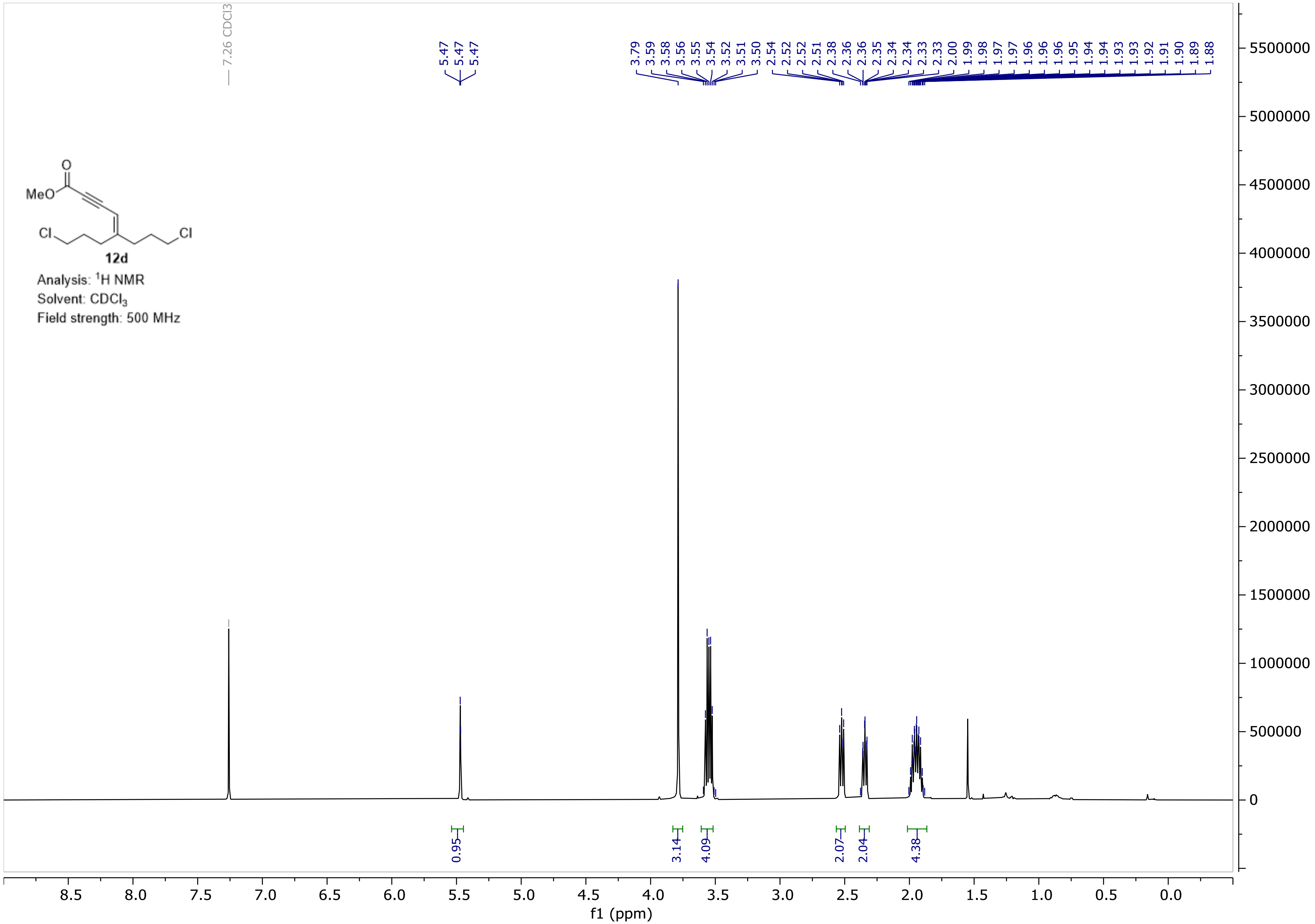
MeO
O
Cl
Cl
12d
Analysis: 1H NMR
Solvent: CDCl3
Field strength: 500 MHz
7.26 CDCl3
5.47
5.47
5.47
3.79
3.59
3.58
3.56
3.55
3.54
3.52
3.51
3.50
2.54
2.52
2.52
2.51
2.38
2.36
2.36
2.35
2.34
2.34
2.33
2.33
2.00
1.99
1.98
1.97
1.97
1.96
1.96
1.95
1.94
1.94
1.93
1.93
1.92
1.91
1.90
1.89
1.88
0.95
3.14
4.09
2.07
2.04
4.38
f1 (ppm)

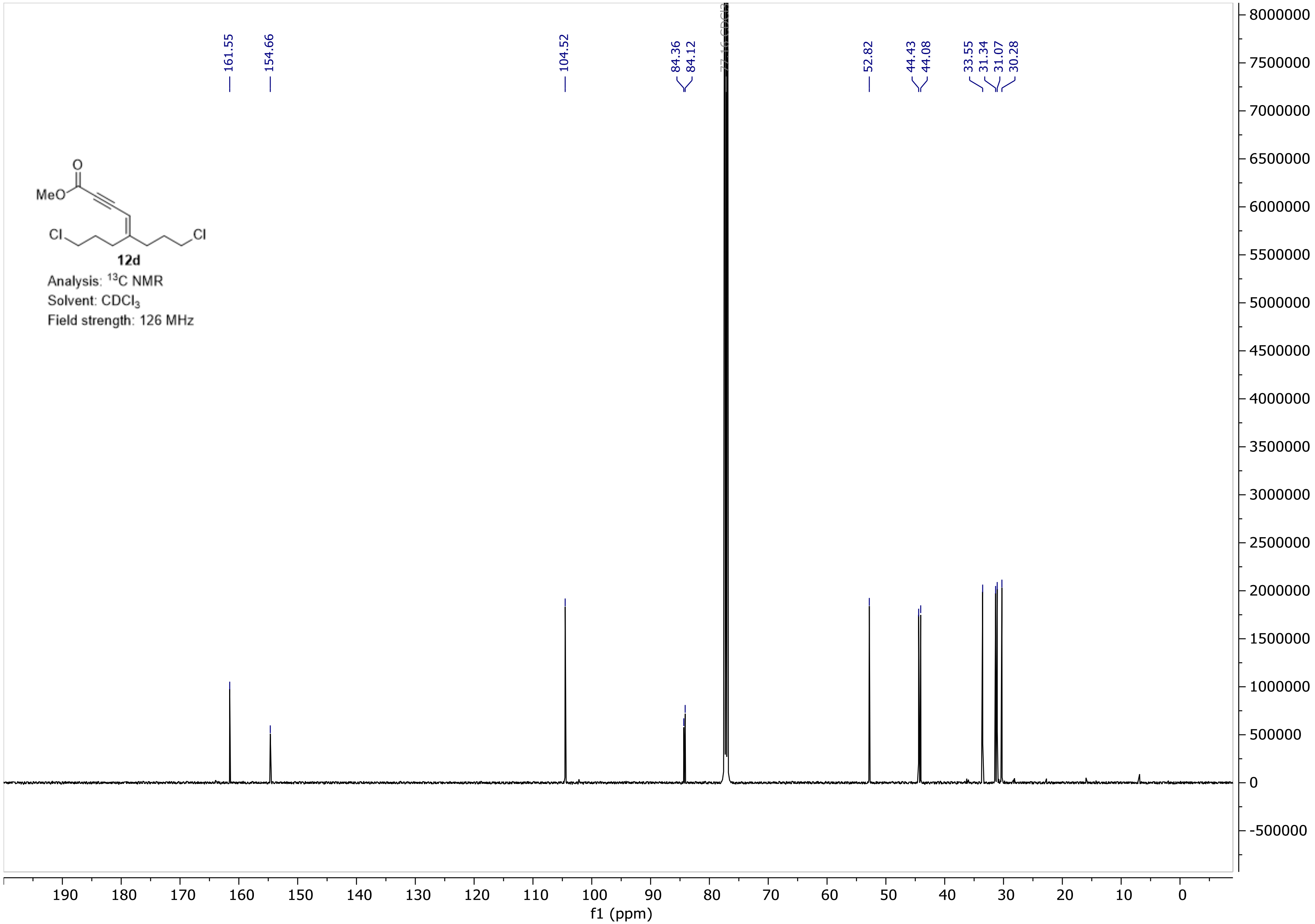

161.55
154.66
104.52
84.36
84.12
52.82
44.43
44.08
33.55
31.34
31.07
30.28
MeO
O
Cl
Cl
12d
Analysis: 13C NMR
Solvent: CDCl3
Field strength: 126 MHz
f1 (ppm)

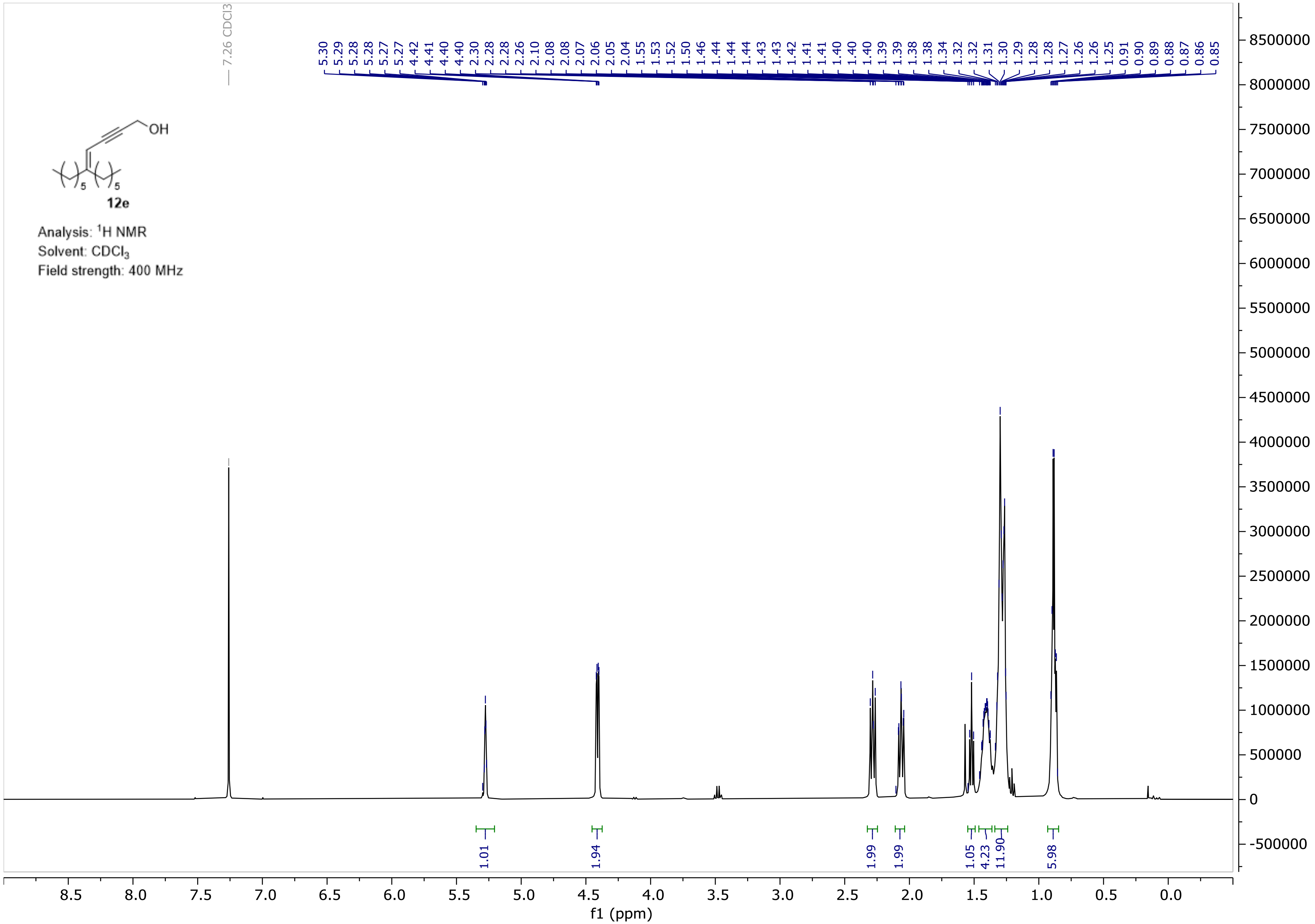

OH
12e
Analysis: ^{1}H NMR
Solvent: $CDCl_3$
Field strength: 400 MHz
7.26 CDCl3
f1 (ppm)

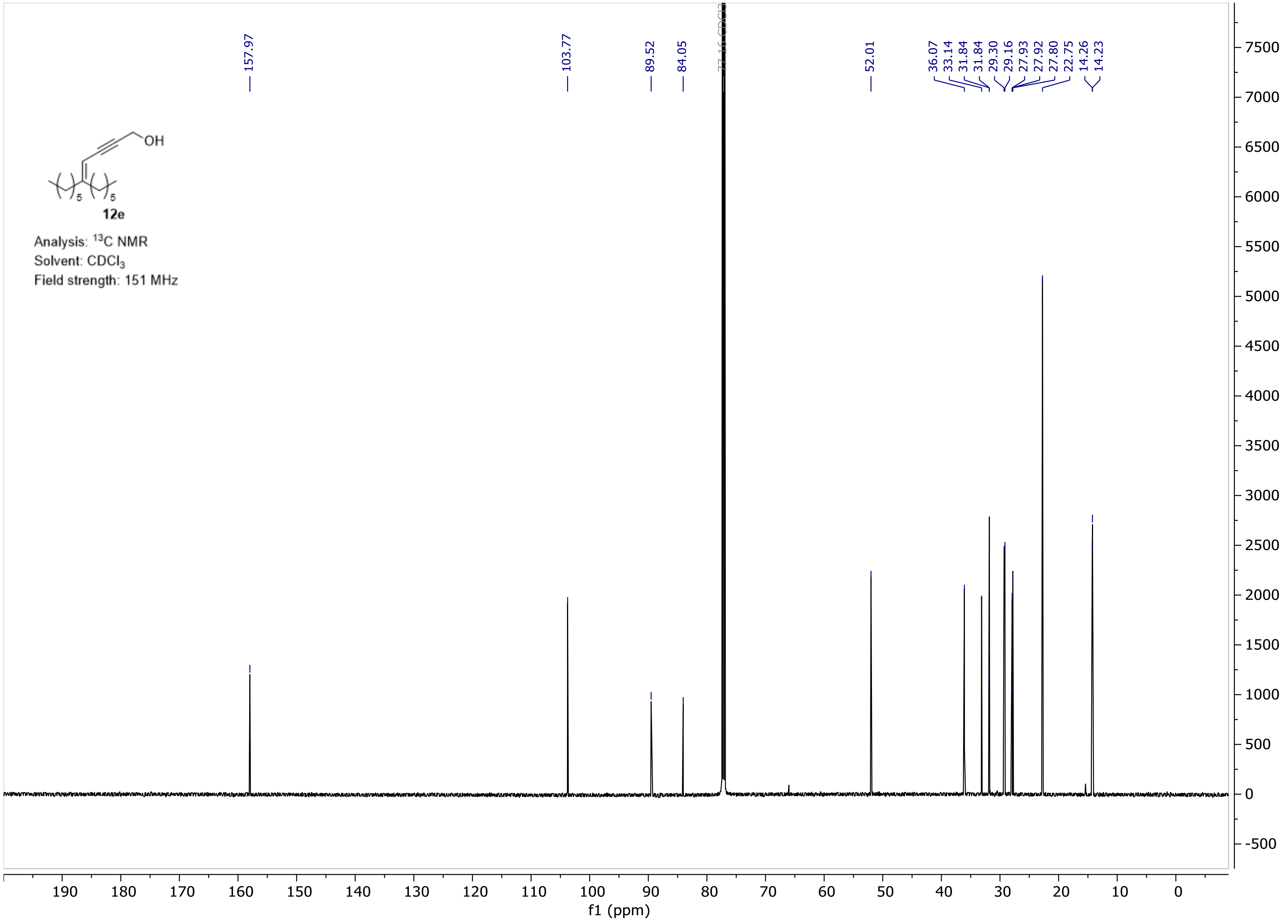
157.97
103.77
89.52
84.05
77.16 CDCl3
52.01
36.07
33.14
31.84
31.84
29.30
29.16
27.93
27.92
27.80
22.75
14.26
14.23
OH
12e
Analysis: 13C NMR
Solvent: CDCl3
Field strength: 151 MHz
f1 (ppm)

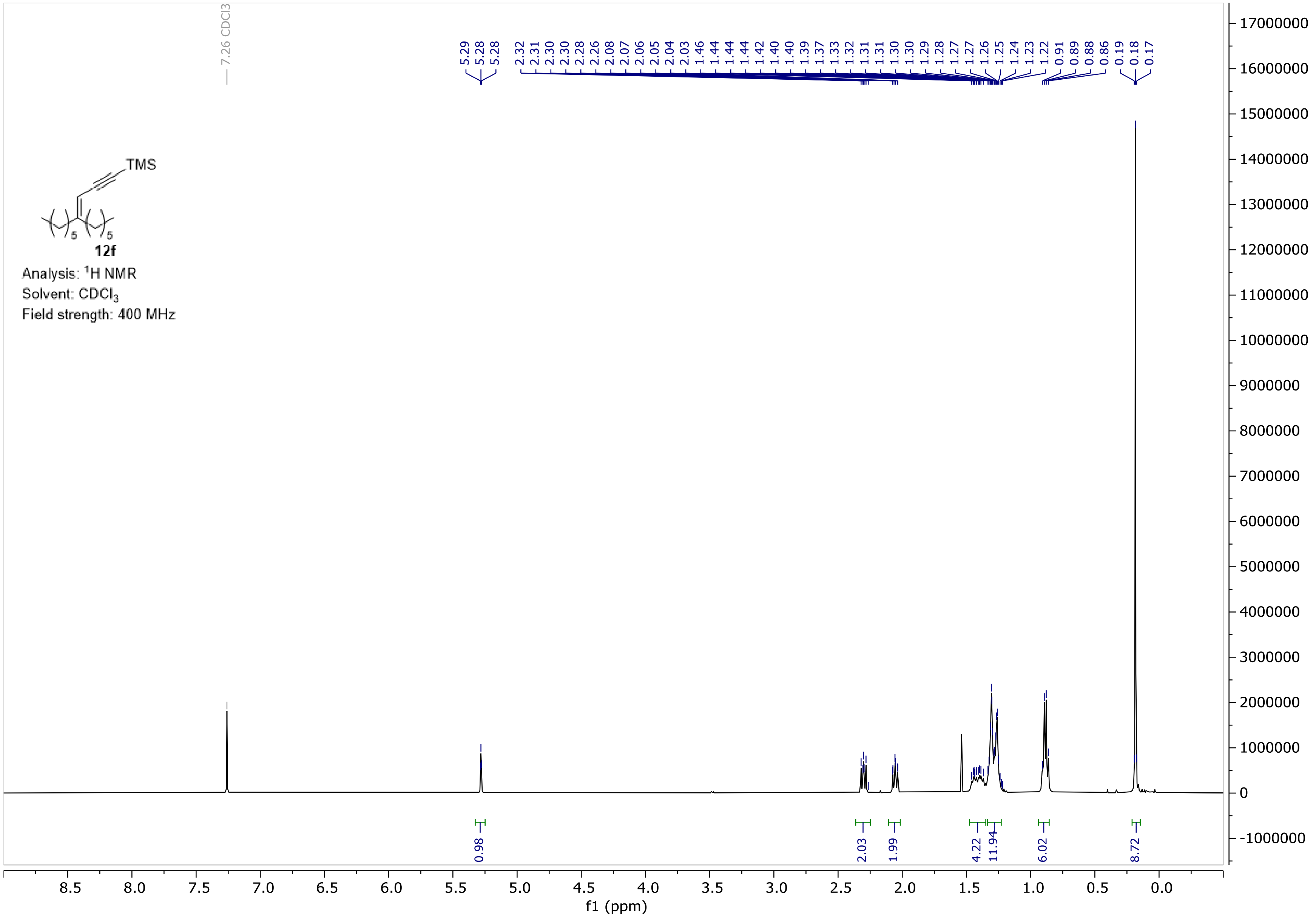
TMS
12f
Analysis: ^{1}H NMR
Solvent: $CDCl_3$
Field strength: 400 MHz
7.26 CDCl3
f1 (ppm)

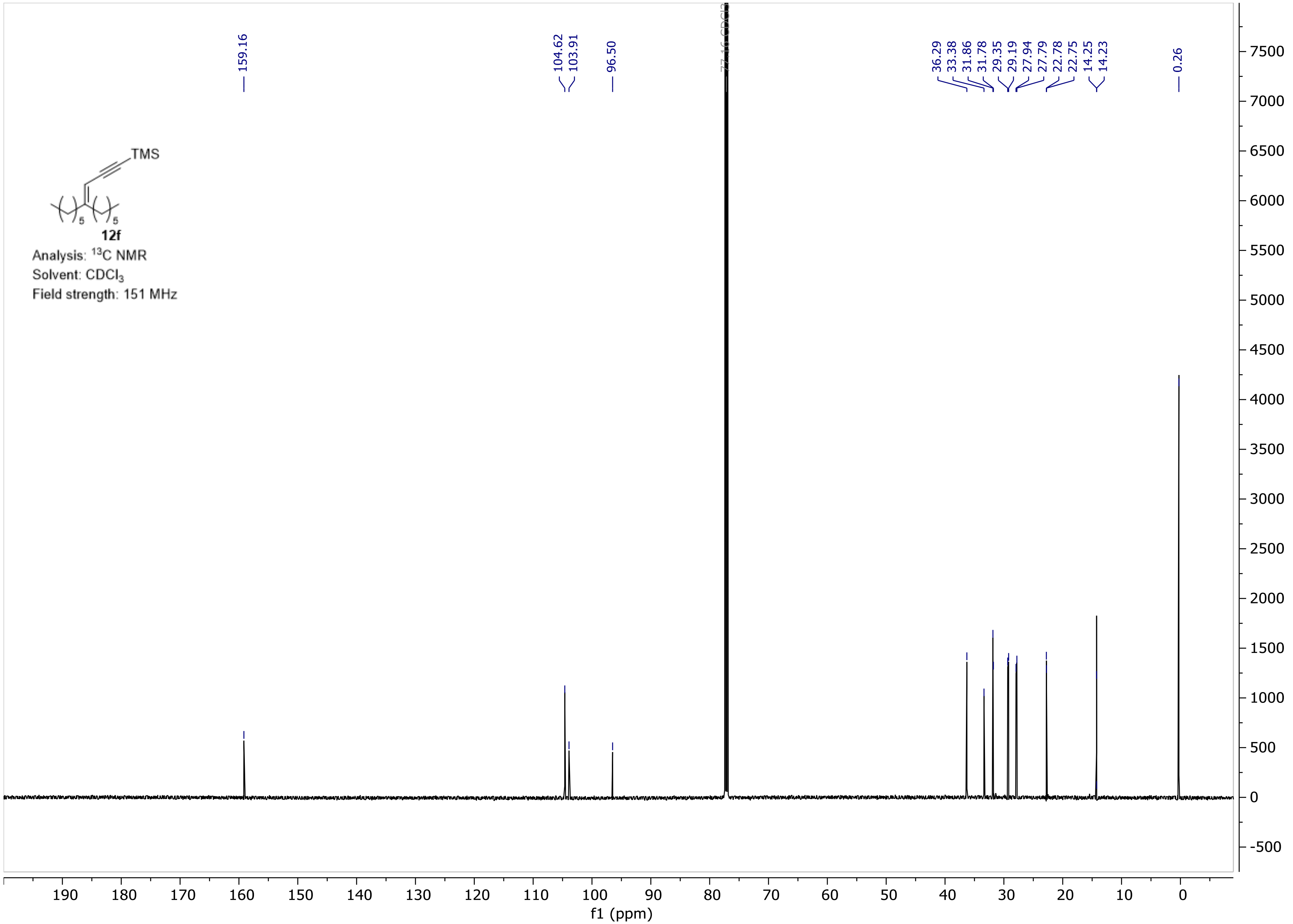
TMS
5
5
12f
Analysis: ^{13}C NMR
Solvent: $CDCl_3$
Field strength: 151 MHz
159.16
104.62
103.91
96.50
77.16 CDCl3
36.29
33.38
31.86
31.78
29.35
29.19
27.94
27.79
22.78
22.75
14.25
14.23
0.26
f1 (ppm)

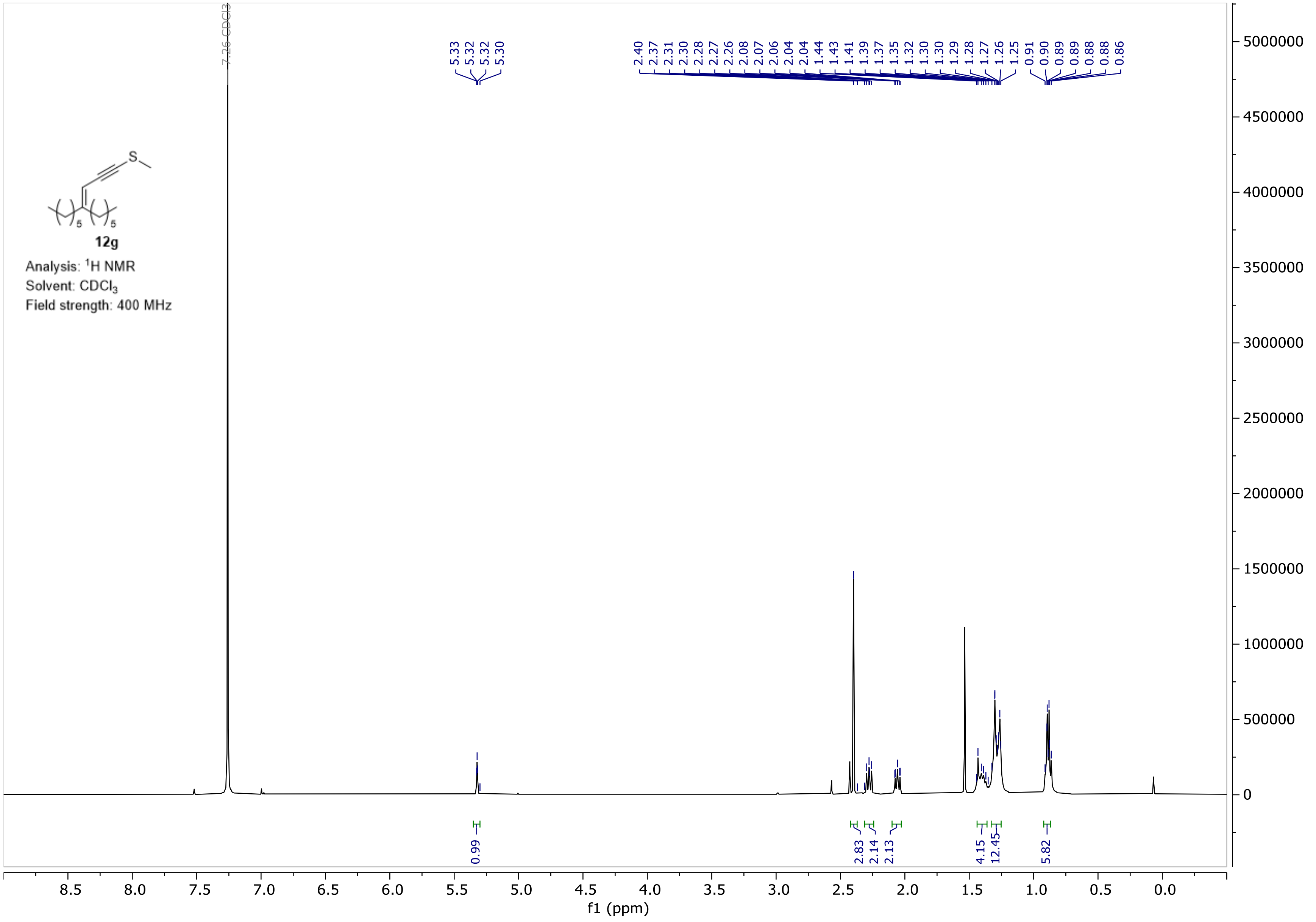
12g
Analysis: 1H NMR
Solvent: CDCl3
Field strength: 400 MHz
7.26 CDCl3
5.33 5.32 5.32 5.30
2.40 2.37 2.31 2.30 2.28 2.27 2.26 2.08 2.07 2.06 2.04 2.04 1.44 1.43 1.41 1.39 1.37 1.35 1.32 1.30 1.30 1.29 1.28 1.27 1.26 1.25 0.91 0.90 0.89 0.89 0.88 0.88 0.86
0.99
2.83
2.14
2.13
4.15
12.45
5.82
f1 (ppm)

12g

Analysis: ^{13}C NMR

Solvent: $CDCl_3$

Field strength: 151 MHz

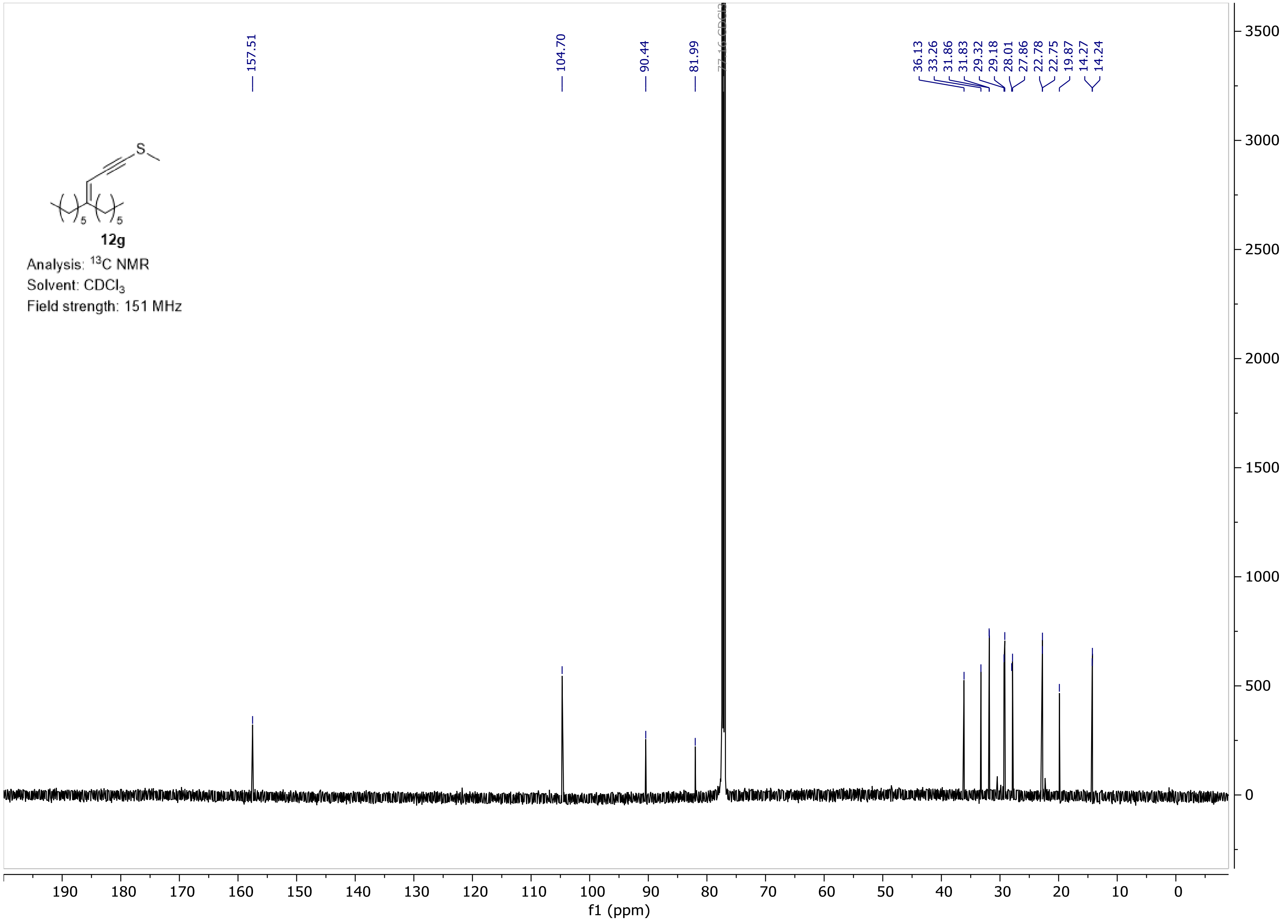

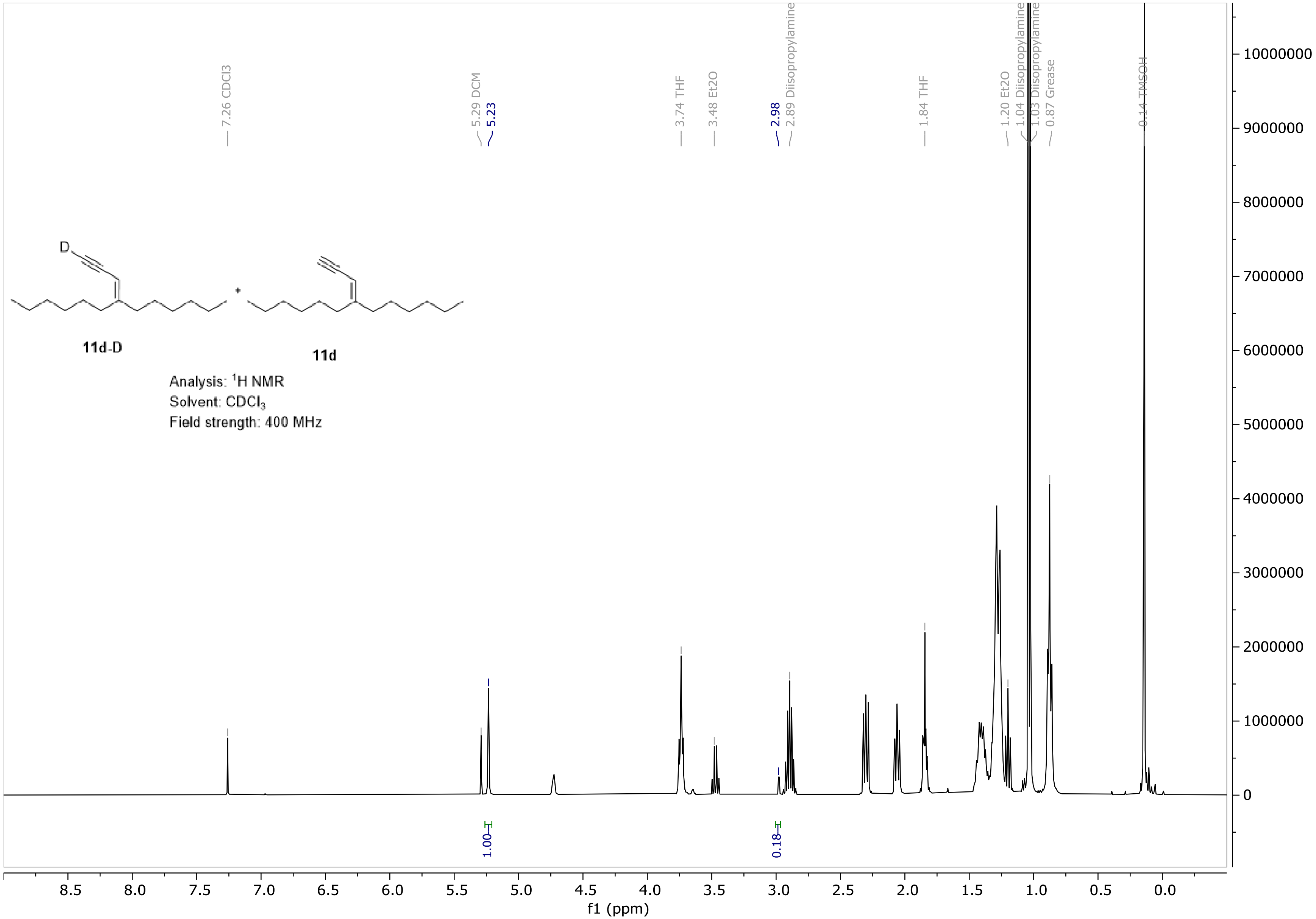

7.26 CDCl3
5.29 DCM
5.23
3.74 THF
3.48 Et2O
2.98
2.89 Diisopropylamine
1.84 THF
1.20 Et2O
1.04 Diisopropylamine
1.03 Diisopropylamine
0.87 Grease
0.14 TMSOH
D
11d-D
11d
Analysis: 1H NMR
Solvent: CDCl3
Field strength: 400 MHz
1.00
0.18
f1 (ppm)

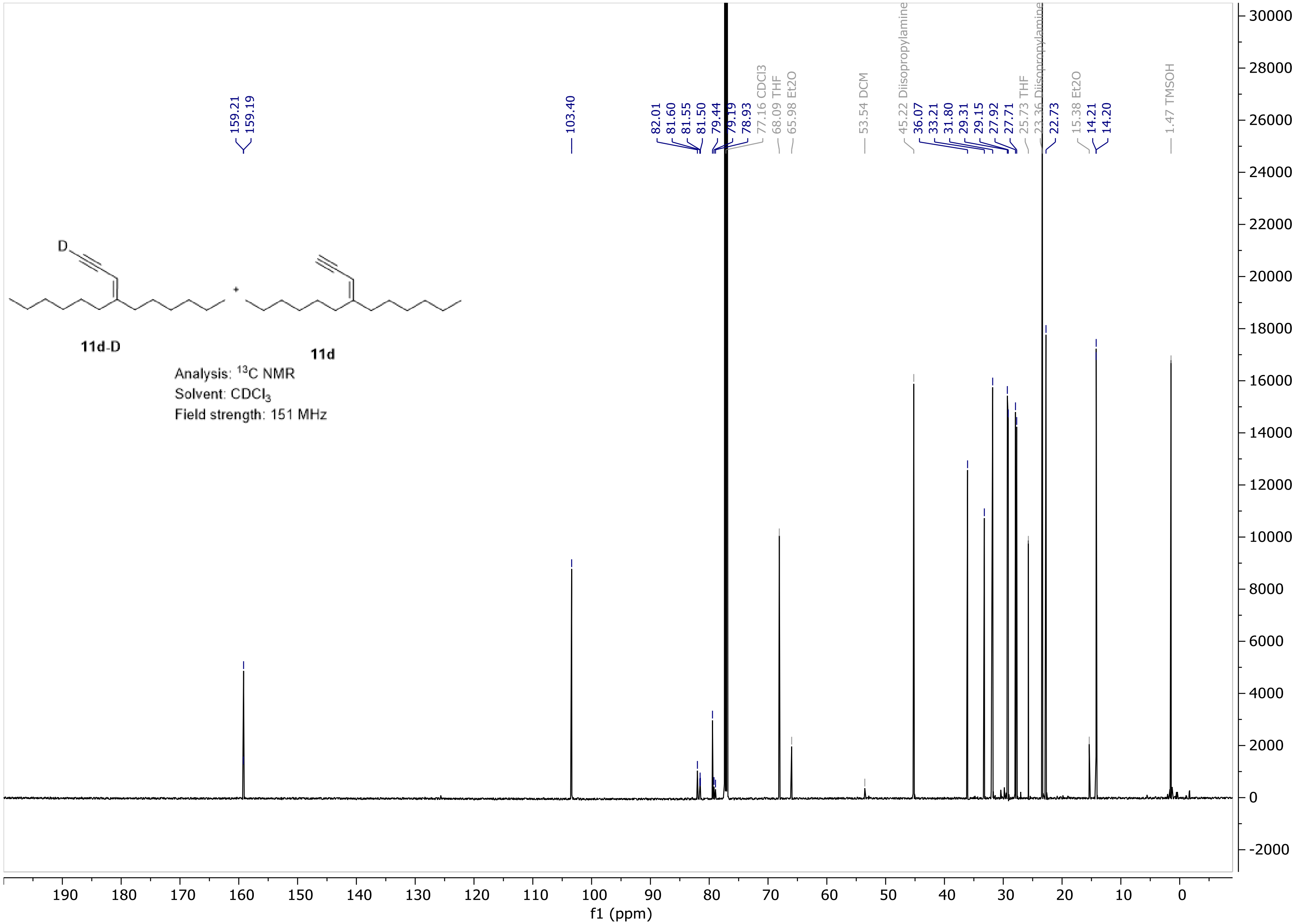

159.21
159.19
103.40
82.01
81.60
81.55
81.50
79.44
79.19
78.93
77.16 CDCl3
68.09 THF
65.98 Et2O
53.54 DCM
45.22 Diisopropylamine
36.07
33.21
31.80
29.31
29.15
27.92
27.71
25.73 THF
23.36 Diisopropylamine
22.73
15.38 Et2O
14.21
14.20
1.47 TMSOH
D
11d-D
11d
Analysis: ¹³C NMR
Solvent: CDCl₃
Field strength: 151 MHz
f1 (ppm)

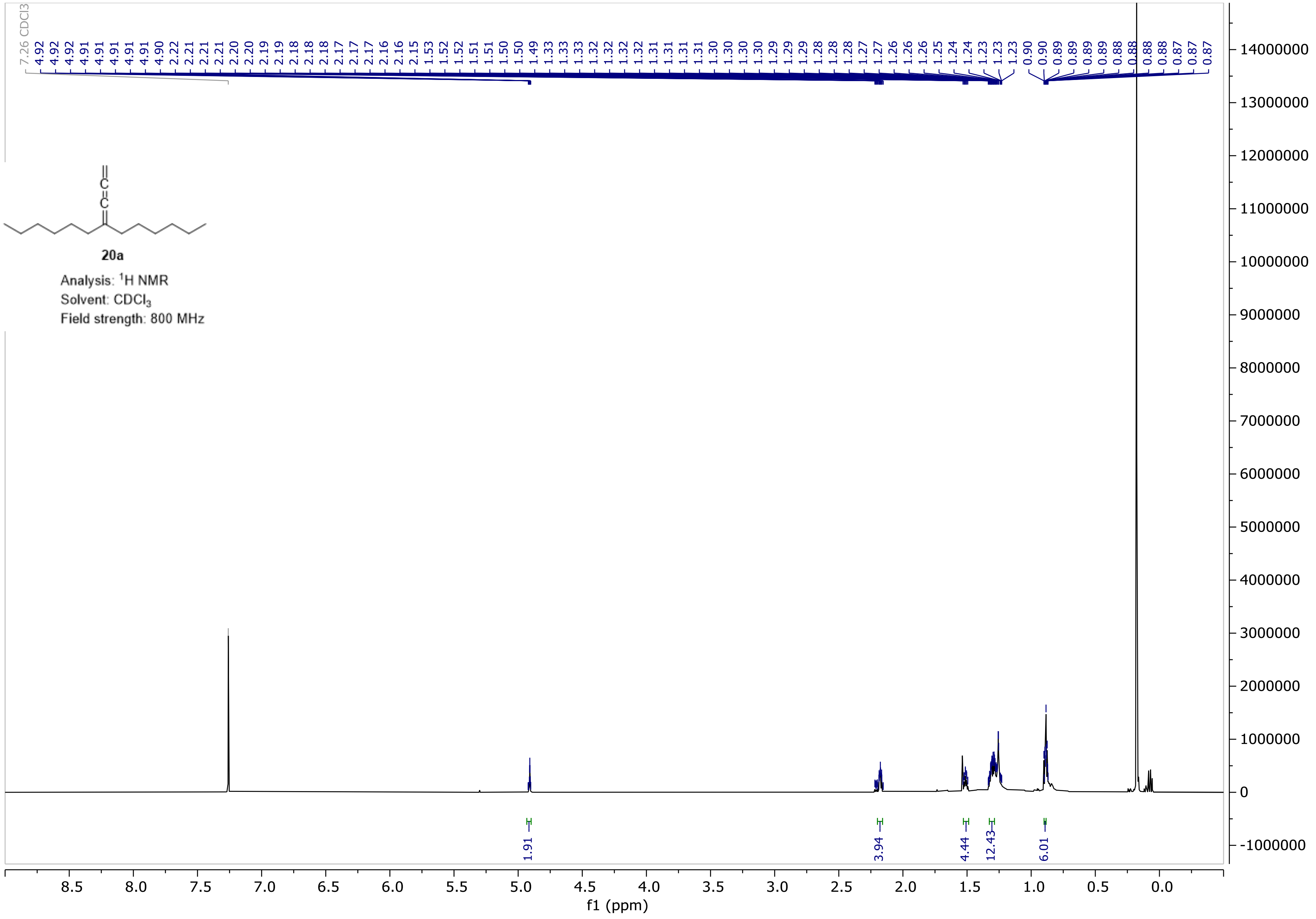

20a
Analysis: ¹H NMR
Solvent: CDCl3
Field strength: 800 MHz
7.26 CDCl3
1.91
3.94
4.44
12.43
6.01
f1 (ppm)

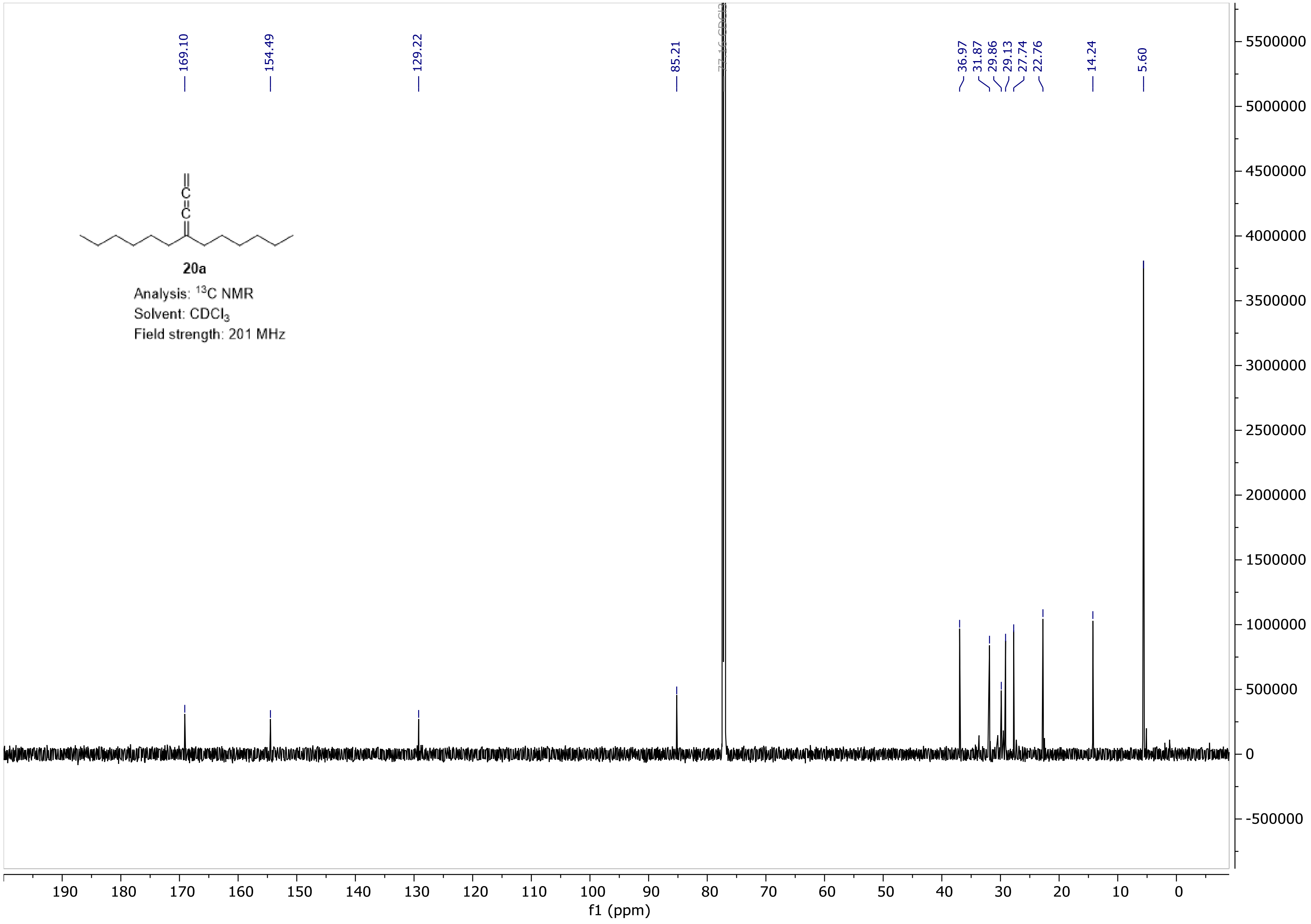
169.10
154.49
129.22
85.21
77.16 CDCl3
36.97
31.87
29.86
29.13
27.74
22.76
14.24
5.60
20a
Analysis: 13C NMR
Solvent: CDCl3
Field strength: 201 MHz
f1 (ppm)

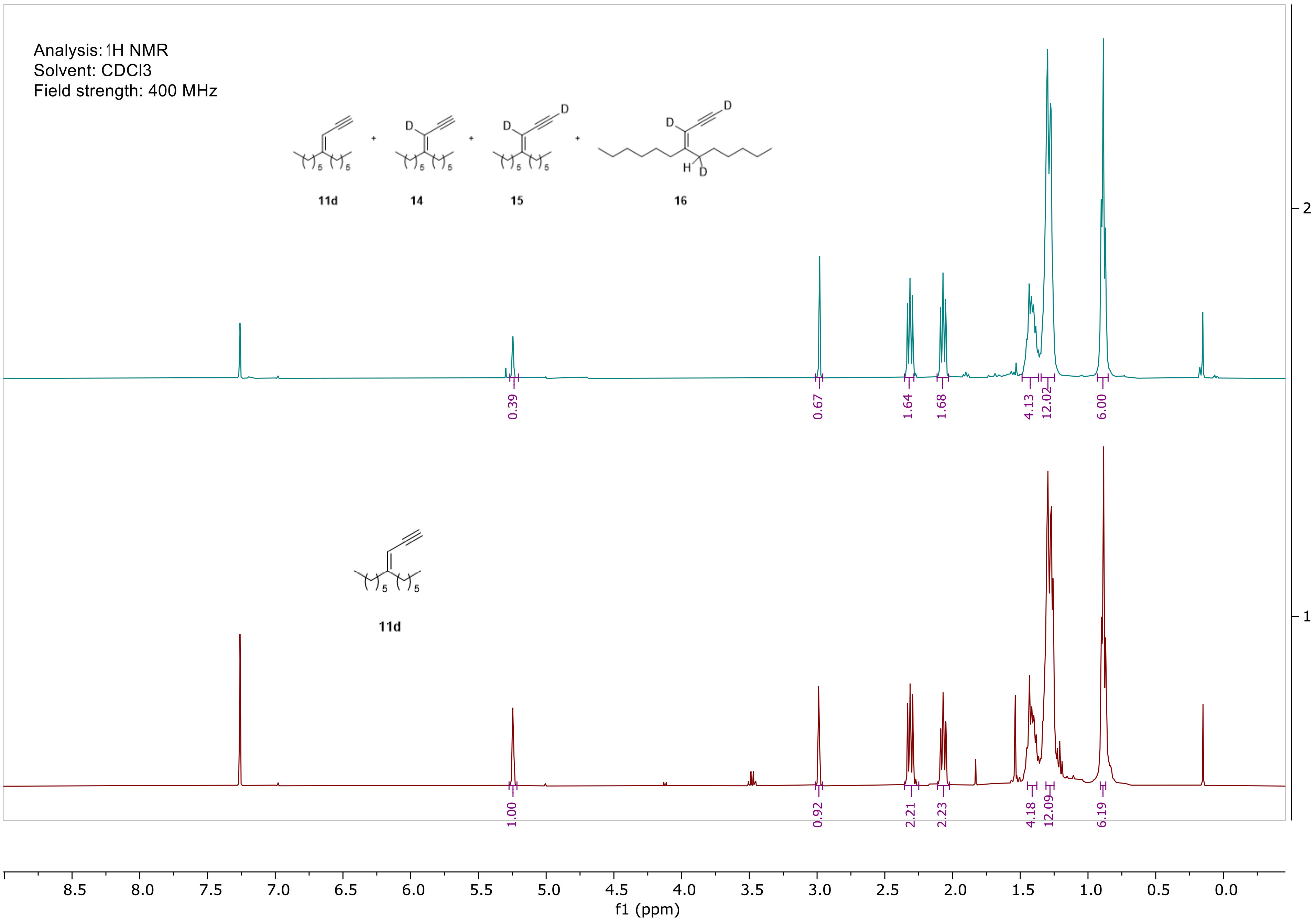

Analysis: 1H NMR
Solvent: CDCl3
Field strength: 400 MHz
11d
14
15
16
0.39
0.67
1.64
1.68
4.13
12.02
6.00
11d
1.00
0.92
2.21
2.23
4.18
12.09
6.19
2
1
8.5 8.0 7.5 7.0 6.5 6.0 5.5 5.0 4.5 4.0 3.5 3.0 2.5 2.0 1.5 1.0 0.5 0.0
f1 (ppm)

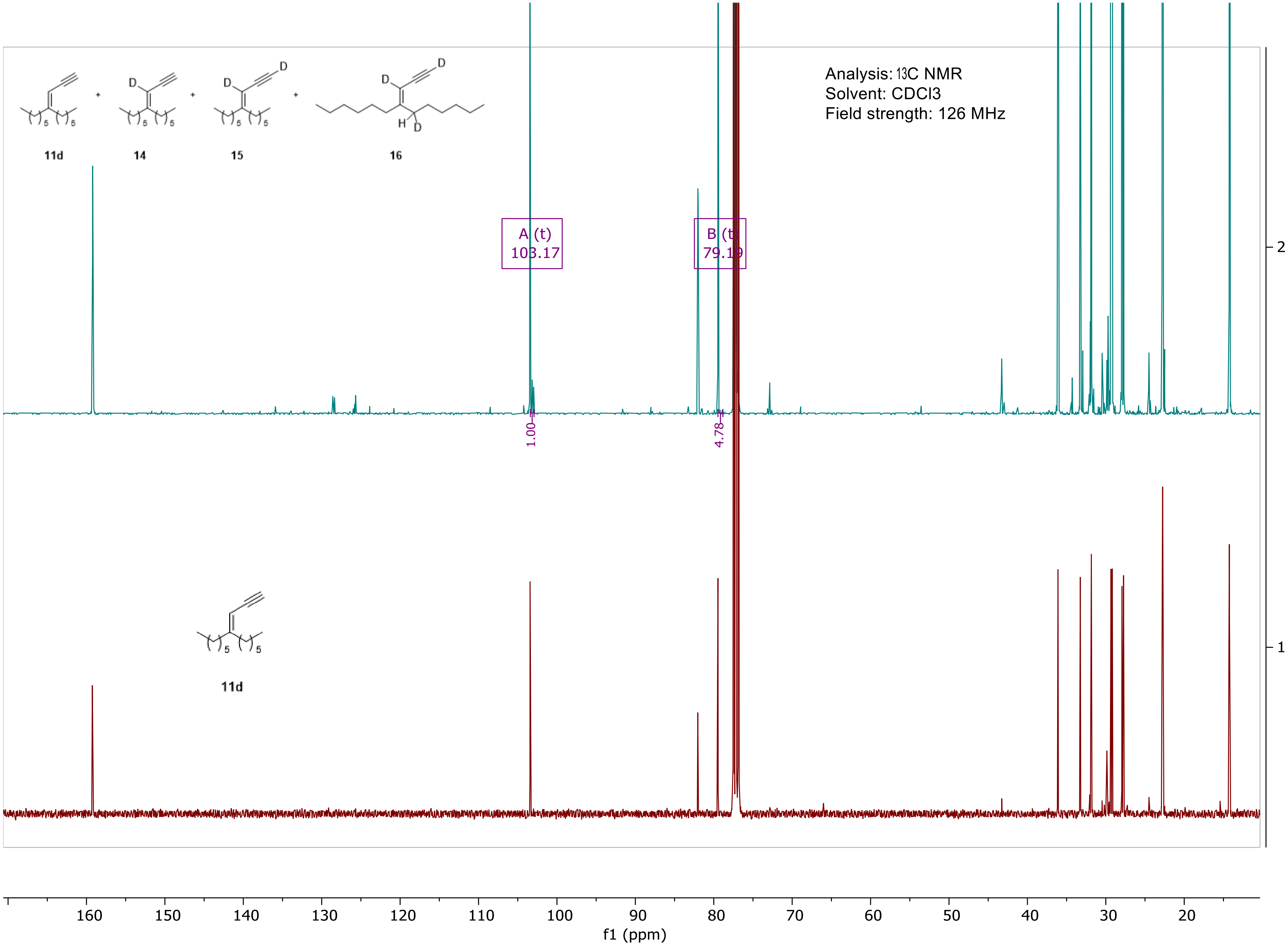

11d
14
15
16
Analysis: 13C NMR
Solvent: CDCl3
Field strength: 126 MHz
A (t)
103.17
B (t)
79.19
1.00
4.78
11d
160
150
140
130
120
110
100
90
80
70
60
50
40
30
20
f1 (ppm)
2
1

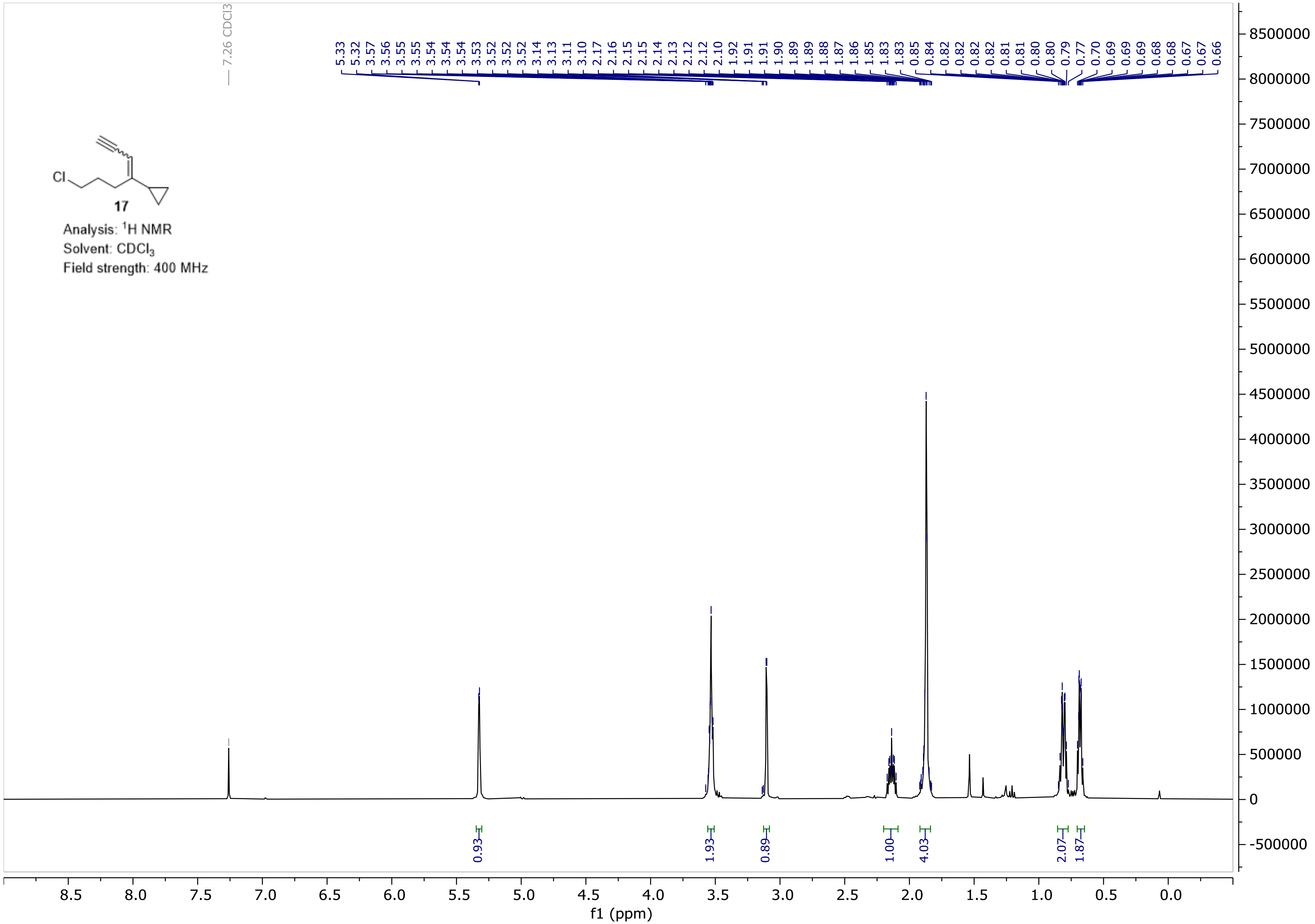

7.26 CDCl3
5.33 5.32 3.57 3.56 3.55 3.55 3.54 3.54 3.54 3.53 3.52 3.52 3.52 3.14 3.13 3.11 3.10 2.17 2.16 2.15 2.15 2.14 2.13 2.12 2.12 2.10 1.92 1.91 1.91 1.90 1.89 1.89 1.88 1.87 1.86 1.85 1.83 1.83 0.85 0.84 0.82 0.82 0.82 0.82 0.81 0.81 0.80 0.80 0.79 0.77 0.70 0.69 0.69 0.69 0.68 0.67 0.67 0.66
Cl
17
Analysis: 1H NMR
Solvent: CDCl3
Field strength: 400 MHz
0.93
1.93
0.89
1.00
4.03
2.07
1.87
f1 (ppm)

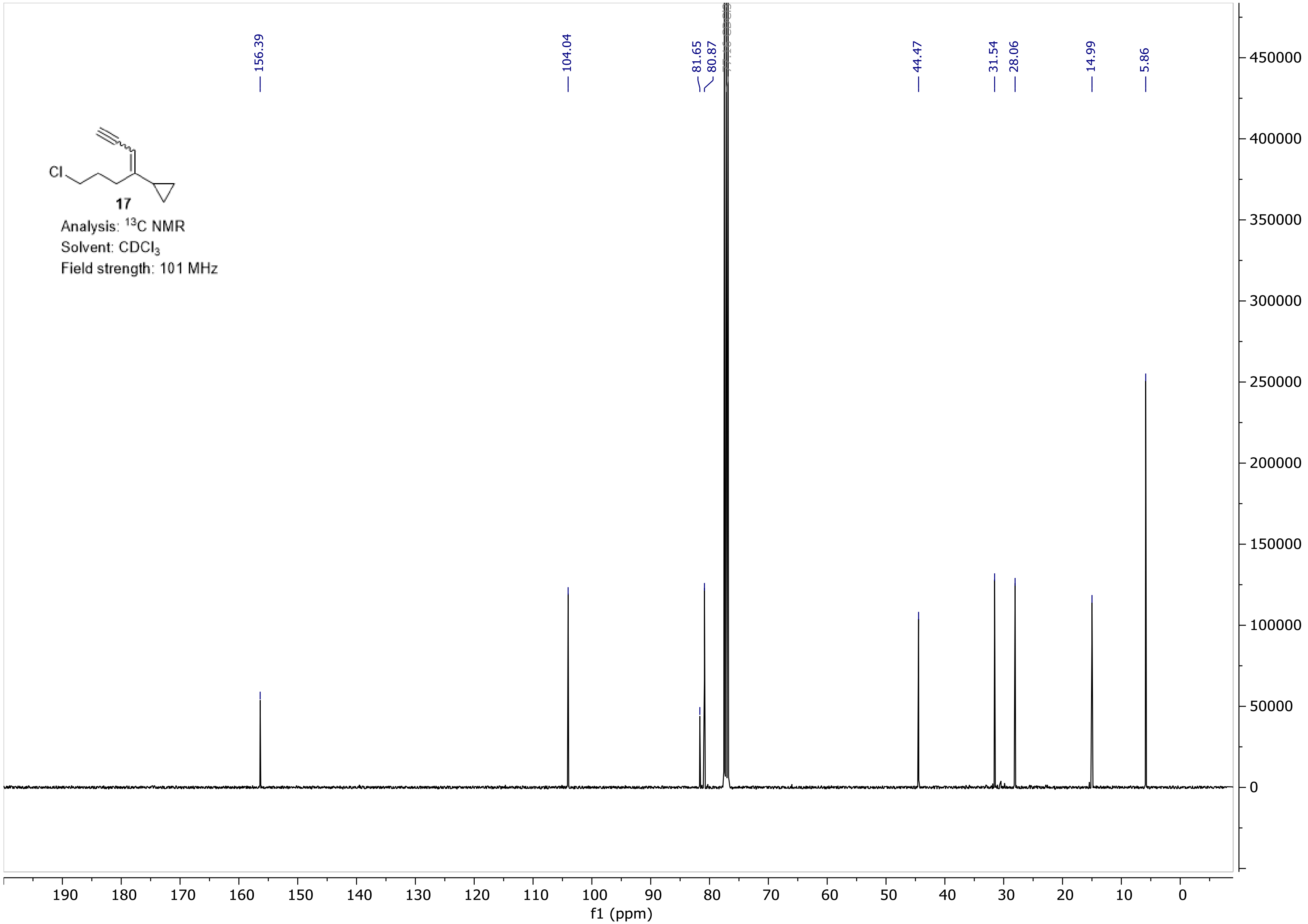
Cl
17
Analysis: ^{13}C NMR
Solvent: $CDCl_3$
Field strength: 101 MHz
156.39
104.04
81.65
80.87
44.47
31.54
28.06
14.99
5.86
f1 (ppm)

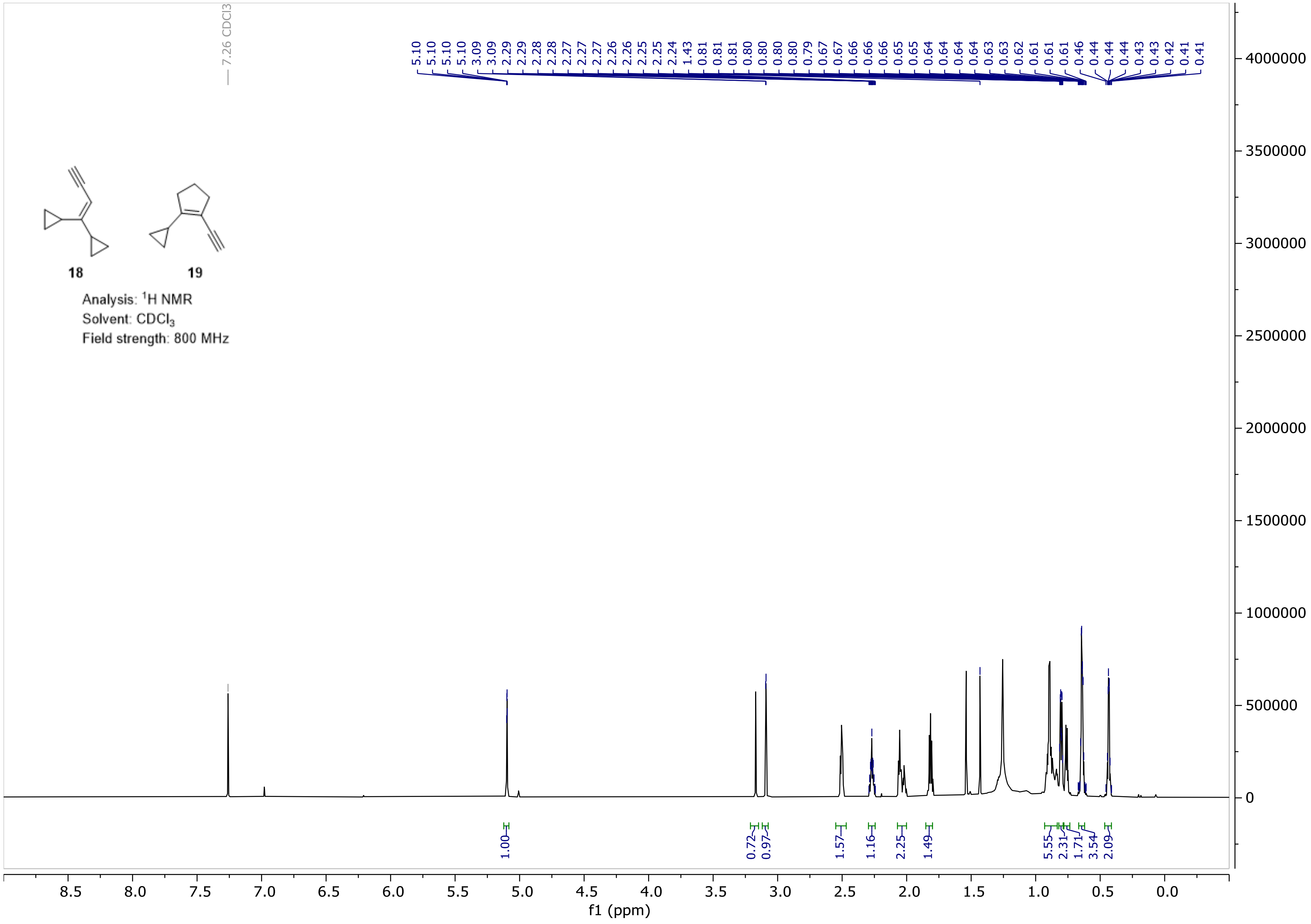
18
19
Analysis: 1H NMR
Solvent: CDCl3
Field strength: 800 MHz
7.26 CDCl3
f1 (ppm)

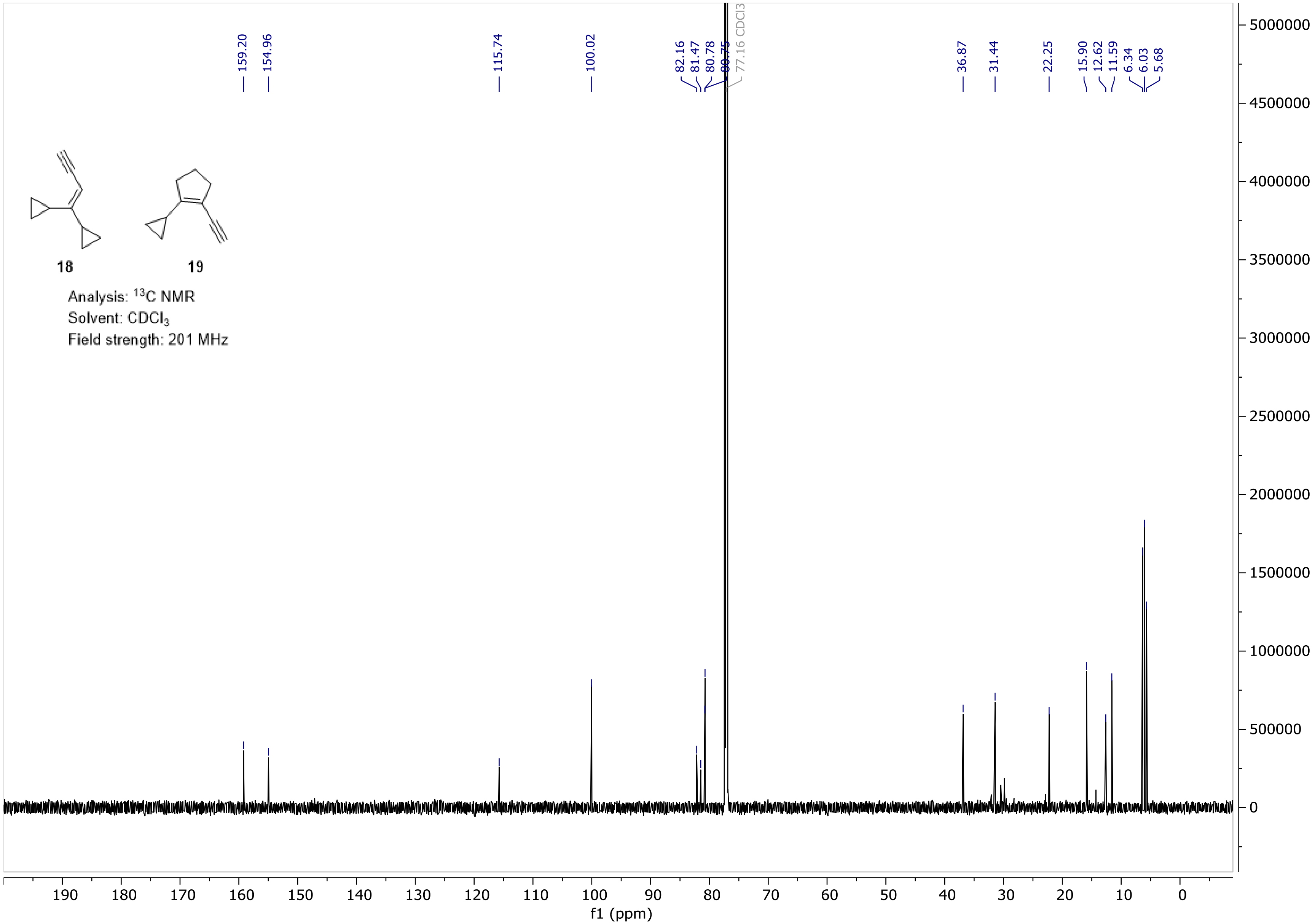

159.20
154.96
115.74
100.02
82.16
81.47
80.78
80.75
77.16 CDCl3
36.87
31.44
22.25
15.90
12.62
11.59
6.34
6.03
5.68
18
19
Analysis: 13C NMR
Solvent: CDCl3
Field strength: 201 MHz
f1 (ppm)
